# Supplementary material for: A Multi-Pronged Technique for Identifying Equisetum palustre and Equisetum arvense—Combining HPTLC, HPLC-ESI-MS/MS and Optimized DNA Barcoding Techniques
Source: Plants (Basel). 2022 Sep 28;11(19):2562. doi: 10.3390/plants11192562 (PMC9573248; doi:10.3390/plants11192562)
Supplement: Supplementary file 1 [file plants-11-02562-s001.zip › plants-1932149 supplementary/Horsetail Supplementary Figures/Figure S1.pdf]

BLAST Results

Questions/comments

Job title: OL304248.1

**RID**	FD53FYMN013 (Expires on 08-14 01:57 am)
**Query ID**	lcl|Query_47981
**Description**	None
**Molecule type**	dna
**Query Length**	333
**Database Name**	nt
**Description**	Nucleotide collection (nt)
**Program**	BLASTN 2.13.0+

Graphic Summary

Distribution of the top 108 Blast Hits on 100 subject sequences

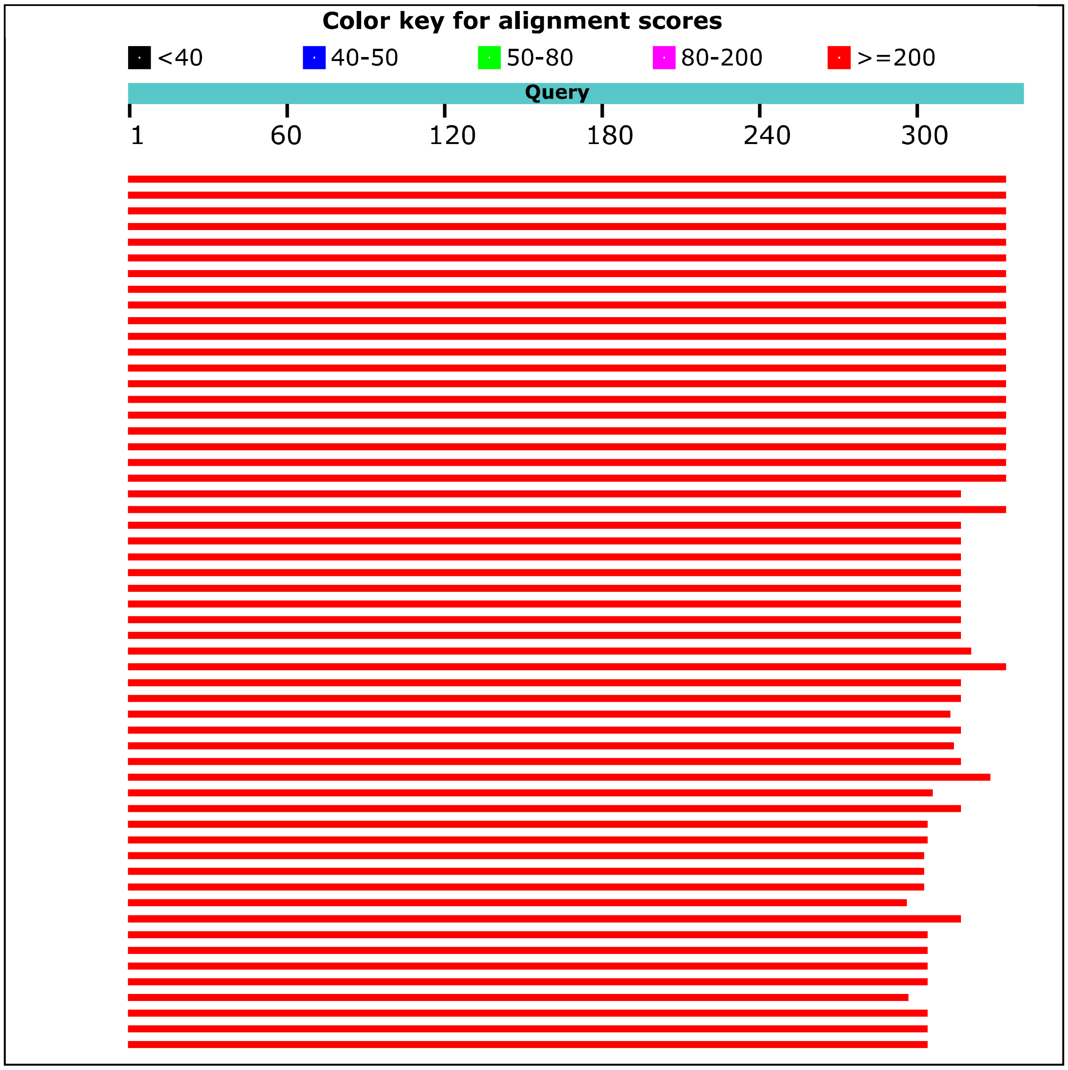

Descriptions

Sequences producing significant alignments:

Description	Max Score	Total Score	Query Cover	E value	Per. Ident	Accession
Equisetum arvense voucher MN01 5.8S ribosomal RNA gene, partial sequence; internal transcribed spacer 2, complete sequence; and large subunit ribosomal RNA gene, partial sequence	616	616	100%	9e-172	100.00%	OL304248.1
Equisetum arvense voucher MN05 5.8S ribosomal RNA gene, partial sequence; internal transcribed spacer 2, complete sequence; and large subunit ribosomal RNA gene, partial sequence	606	606	100%	5e-169	99.40%	OL304249.1
uncultured fungus genomic DNA sequence contains ITS1	601	601	100%	2e-167	99.10%	OW836430.1
Equisetum arvense voucher MN06 5.8S ribosomal RNA gene, partial sequence; internal transcribed spacer 2, complete sequence; and large subunit ribosomal RNA gene, partial sequence	601	601	100%	2e-167	99.10%	OL304250.1
Chloroidium sp. FACHB-2422 small subunit ribosomal RNA gene, partial sequence; internal transcribed spacer 1, 5.8S ribosomal RNA gene, and internal transcribed spacer 2, complete sequence; and large subunit ribosomal RNA gene, partial sequence	601	601	100%	2e-167	99.10%	MT006211.1
Chloroidium saccharophilum genomic DNA containing 18S rRNA gene, ITS1, 5.8S rRNA gene, ITS2, 28S rRNA gene, culture collection CCAP 211/31	595	595	100%	1e-165	98.81%	FR865664.1
Equisetum arvense voucher Equisetum_arvense_ITS.fasta_2 5.8S ribosomal RNA gene, partial sequence; internal transcribed spacer 2, complete sequence; and large subunit ribosomal RNA gene, partial sequence	593	593	100%	4e-165	98.51%	MW040323.1
Equisetum arvense voucher 34008 5.8S ribosomal RNA gene, partial sequence; internal transcribed spacer 2, complete sequence; and large subunit ribosomal RNA gene, partial sequence	593	593	100%	4e-165	98.51%	MW040322.1
Chloroidium saccharophilum strain FACHB-1796 18S ribosomal RNA gene, partial sequence; internal transcribed spacer 1, 5.8S ribosomal RNA gene, and internal transcribed spacer 2, complete sequence; and 28S ribosomal RNA gene, partial sequence	590	590	100%	5e-164	98.51%	KX024689.1
uncultured Pezizaceae genomic DNA sequence contains ITS1	590	590	100%	5e-164	98.51%	OU496020.1
Equisetum arvense voucher Equisetum_arvense_ITS.fasta_1 5.8S ribosomal RNA gene, partial sequence; internal transcribed spacer 2, complete sequence; and large subunit ribosomal RNA gene, partial sequence	590	590	100%	5e-164	98.21%	MW040324.1

Description	Max Score	Total Score	Query Cover	E value	Per. Ident	Accession
Unclassified Viridiplantae clone 1537 5.8S ribosomal RNA gene, partial sequence; internal transcribed spacer 2, complete sequence; and large subunit ribosomal RNA gene, partial sequence	584	584	100%	3e-162	98.21%	MF570051.1
Chloroidium ellipsoideum isolate FG2/4.5E 18S ribosomal RNA gene, partial sequence; internal transcribed spacer 1, 5.8S ribosomal RNA gene, and internal transcribed spacer 2, complete sequence; and 26S ribosomal RNA gene, partial sequence	584	584	100%	3e-162	98.21%	KM020071.1
Chloroidium saccharophilum isolate FG2/1.3 18S ribosomal RNA gene, partial sequence; internal transcribed spacer 1, 5.8S ribosomal RNA gene, and internal transcribed spacer 2, complete sequence; and 26S ribosomal RNA gene, partial sequence	584	584	100%	3e-162	98.21%	KM020070.1
Chloroidium saccharophilum var. saccharophilum strain UTEX247 18S ribosomal RNA gene, partial sequence; internal transcribed spacer 1, 5.8S ribosomal RNA gene, and internal transcribed spacer 2, complete sequence; and 28S ribosomal RNA gene, partial sequence	584	584	100%	3e-162	98.21%	KJ676130.1
Chloroidium saccharophilum strain OD-1-1-C small subunit ribosomal RNA gene, partial sequence; internal transcribed spacer 1, 5.8S ribosomal RNA gene, and internal transcribed spacer 2, complete sequence; and large subunit ribosomal RNA gene, partial sequence	584	584	100%	3e-162	98.21%	MT991539.1
Chloroidium ellipsoideum genomic DNA containing 18S rRNA gene, ITS1, 5.8S rRNA gene, ITS2, 28S rRNA gene, culture collection CCAP 211/40	584	584	100%	3e-162	98.21%	FR865669.1
Chloroidium saccharophilum genomic DNA containing 18S rRNA gene, ITS1, 5.8S rRNA gene, ITS2, 28S rRNA gene, culture collection CCAP 211/32	584	584	100%	3e-162	98.21%	FR865665.1
Chloroidium saccharophilum var. saccharophilum strain UTEX2469 18S ribosomal RNA gene, partial sequence; internal transcribed spacer 1, 5.8S ribosomal RNA gene, and internal transcribed spacer 2, complete sequence; and 28S ribosomal RNA gene, partial sequence	573	573	100%	5e-159	97.61%	KJ676131.1
Chloroidium sp. FACHB-2420 small subunit ribosomal RNA gene, partial sequence; internal transcribed spacer 1, 5.8S ribosomal RNA gene, and internal transcribed spacer 2, complete sequence; and large subunit ribosomal RNA gene, partial sequence	573	573	100%	5e-159	97.61%	MT006209.1
Chloroidium saccharophilum strain ISBAL-007 18S ribosomal RNA gene, internal transcribed spacer 1, 5.8S ribosomal RNA gene, internal transcribed spacer 2, and 28S ribosomal RNA gene, region	569	569	94%	7e-158	99.06%	MH551492.1

Description	Max Score	Total Score	Query Cover	E value	Per. Ident	Accession
'Chlorella' ellipsoidea strain Ce 18S ribosomal RNA gene, partial sequence; internal transcribed spacer 1, 5.8S ribosomal RNA gene, and internal transcribed spacer 2, complete sequence; and 26S ribosomal RNA gene, partial sequence	568	568	100%	3e-157	97.31%	EU038292.1
Chlorella saccharophila partial 18S rRNA gene, ITS1, 5.8S rRNA, ITS2 and partial 28S rRNA gene, strain SAG 2120	558	558	94%	2e-154	98.43%	FM946008.1
Chlorella saccharophila partial 18S rRNA gene, ITS1, 5.8S rRNA, ITS2 and partial 28S rRNA gene, strain CCAP 211/42	558	558	94%	2e-154	98.43%	FM946004.1
Chlorella saccharophila partial 18S rRNA gene, ITS1, 5.8S rRNA, ITS2 and partial 28S rRNA gene, strain CCAP 211/36	558	558	94%	2e-154	98.43%	FM946003.1
Chloroidium saccharophilum strain CCAP 211/27 18S ribosomal RNA gene, internal transcribed spacer 1, 5.8S ribosomal RNA gene, internal transcribed spacer 2, and 28S ribosomal RNA gene, region	553	553	94%	7e-153	98.11%	MH551491.1
Chloroidium saccharophilum strain CCAP 211/40 small subunit ribosomal RNA gene, partial sequence; internal transcribed spacer 1 and 5.8S ribosomal RNA gene, complete sequence; and internal transcribed spacer 2, partial sequence	553	553	94%	7e-153	98.11%	MH551490.1
Chlorella saccharophila partial 18S rRNA gene, ITS1, 5.8S rRNA, ITS2 and partial 28S rRNA gene, strain SAG 2197	553	553	94%	7e-153	98.11%	FM946010.1
Chlorella saccharophila partial 18S rRNA gene, ITS1, 5.8S rRNA, ITS2 and partial 28S rRNA gene, strain SAG 211-1c	553	553	94%	7e-153	98.11%	FM946007.1
Chlorella saccharophila partial 18S rRNA gene, ITS1, 5.8S rRNA, ITS2 and partial 28S rRNA gene, strain CCAP 211/32	553	553	94%	7e-153	98.11%	FM946001.1
Chlorella sp. UTEX 2068 18S ribosomal RNA gene, partial sequence; internal transcribed spacer 1, 5.8S ribosomal RNA gene, and internal transcribed spacer 2, complete sequence; and 28S ribosomal RNA gene, partial sequence	549	549	96%	9e-152	97.52%	KJ676132.1
Chloroidium saccharophilum genomic DNA containing 18S rRNA gene, ITS1, 5.8S rRNA gene, ITS2, 28S rRNA gene, culture collection CCAP 211/58	549	549	100%	9e-152	96.42%	FR865677.1
Chloroidium saccharophilum strain FaII/B-1b small subunit ribosomal RNA gene, partial sequence; internal transcribed spacer 1 and 5.8S ribosomal RNA gene, complete sequence; and internal transcribed spacer 2, partial sequence	547	547	94%	3e-151	97.80%	MH551489.1
Chloroidium saccharophilum strain SAG 2055 small subunit ribosomal RNA gene, partial sequence; internal transcribed spacer 1 and 5.8S ribosomal RNA gene, complete sequence; and internal transcribed spacer 2, partial sequence	547	547	94%	3e-151	97.80%	MH551488.1

Description	Max Score	Total Score	Query Cover	E value	Per. Ident	Accession
Chloroidium saccharophilum strain KMMCC 192 18S ribosomal RNA gene, internal transcribed spacer 1, 5.8S ribosomal RNA gene, internal transcribed spacer 2, and 28S ribosomal RNA gene, region	545	545	93%	1e-150	98.09%	JQ315766.1
Chlorella saccharophila partial 18S rRNA gene, ITS1, 5.8S rRNA, ITS2 and partial 28S rRNA gene, strain SAG 211-1b	545	545	94%	1e-150	97.80%	FM946005.1
Uncultured alga genes for 5.8S rRNA, ITS2, 28S rRNA, partial and complete sequence, note: OTU163	542	542	93%	2e-149	97.78%	LC381753.1
Chlorella saccharophila partial 18S rRNA gene, ITS1, 5.8S rRNA, ITS2 and partial 28S rRNA gene, strain SAG 211-9a	542	542	94%	2e-149	97.48%	FM946000.1
Chloroidium saccharophilum strain KMMCC 158 18S ribosomal RNA gene, internal transcribed spacer 1, 5.8S ribosomal RNA gene, internal transcribed spacer 2, and 28S ribosomal RNA gene, region	531	531	98%	3e-146	95.47%	JQ315771.1
Chloroidium saccharophilum strain KMMCC 195 18S ribosomal RNA gene, internal transcribed spacer 1, 5.8S ribosomal RNA gene, internal transcribed spacer 2, and 28S ribosomal RNA gene, region	527	527	91%	4e-145	97.72%	JQ315769.1
Chloroidium saccharophilum strain CCAP 264/2 small subunit ribosomal RNA gene, partial sequence; internal transcribed spacer 1 and 5.8S ribosomal RNA gene, complete sequence; and internal transcribed spacer 2, partial sequence	523	523	94%	6e-144	96.54%	MH551493.1
Uncultured Chloroidium isolate A207 internal transcribed spacer 1, partial sequence; 5.8S ribosomal RNA gene, complete sequence; and internal transcribed spacer 2, partial sequence	523	523	90%	6e-144	97.70%	KP314644.1
Uncultured Chloroidium isolate A103 internal transcribed spacer 1, partial sequence; 5.8S ribosomal RNA gene, complete sequence; and internal transcribed spacer 2, partial sequence	523	523	90%	6e-144	97.70%	KP314563.1
Chloroidium saccharophilum strain St6 small subunit ribosomal RNA gene, partial sequence; internal transcribed spacer 1 and 5.8S ribosomal RNA gene, complete sequence; and internal transcribed spacer 2, partial sequence	521	521	90%	2e-143	97.70%	OM472016.1
Chloroidium saccharophilum strain ACSSI 340 small subunit ribosomal RNA gene, partial sequence; internal transcribed spacer 1 and 5.8S ribosomal RNA gene, complete sequence; and internal transcribed spacer 2, partial sequence	521	521	90%	2e-143	97.70%	MT827198.1
Chloroidium saccharophilum strain ACSSI 339 small subunit ribosomal RNA gene, partial sequence; internal transcribed spacer 1 and 5.8S ribosomal RNA gene, complete sequence; and internal transcribed spacer 2, partial sequence	521	521	90%	2e-143	97.70%	MT827197.1

Description	Max Score	Total Score	Query Cover	E value	Per. Ident	Accession
Chloroidium saccharophilum strain CCM-UDEC 143 18S ribosomal RNA gene, partial sequence; and internal transcribed spacer 1, 5S ribosomal RNA gene, and internal transcribed spacer 2, complete sequence	520	520	88%	7e-143	98.32%	KC517116.1
Chloroidium saccharophilum strain CCAP 211/58 small subunit ribosomal RNA gene, partial sequence; internal transcribed spacer 1 and 5.8S ribosomal RNA gene, complete sequence; and internal transcribed spacer 2, partial sequence	518	518	94%	3e-142	96.23%	MH551494.1
Uncultured Chloroidium isolate A214 internal transcribed spacer 1, partial sequence; 5.8S ribosomal RNA gene, complete sequence; and internal transcribed spacer 2, partial sequence	518	518	90%	3e-142	97.38%	KP314651.1
Uncultured Chloroidium isolate A032 internal transcribed spacer 1, partial sequence; 5.8S ribosomal RNA gene, complete sequence; and internal transcribed spacer 2, partial sequence	518	518	90%	3e-142	97.38%	KP314515.1
Uncultured Chloroidium isolate A029 internal transcribed spacer 1, partial sequence; 5.8S ribosomal RNA gene, complete sequence; and internal transcribed spacer 2, partial sequence	518	518	90%	3e-142	97.38%	KP314513.1
Uncultured Chloroidium isolate A011 internal transcribed spacer 1, partial sequence; 5.8S ribosomal RNA gene, complete sequence; and internal transcribed spacer 2, partial sequence	518	518	90%	3e-142	97.38%	KP314497.1
Chloroidium saccharophilum genomic DNA sequence contains 5.8S rRNA gene, ITS2, strain Chlorella saccharophila RNY	516	516	88%	9e-142	97.99%	LR215745.1
Uncultured Chloroidium isolate A023 internal transcribed spacer 1, partial sequence; 5.8S ribosomal RNA gene, complete sequence; and internal transcribed spacer 2, partial sequence	510	510	90%	4e-140	97.05%	KP314508.1
Uncultured Chloroidium isolate A141 internal transcribed spacer 1, partial sequence; 5.8S ribosomal RNA gene, complete sequence; and internal transcribed spacer 2, partial sequence	505	505	90%	2e-138	96.72%	KP314596.1
Uncultured Chloroidium isolate A217 internal transcribed spacer 1, partial sequence; 5.8S ribosomal RNA gene, complete sequence; and internal transcribed spacer 2, partial sequence	499	499	90%	9e-137	96.39%	KP314654.1
'Chlorella' saccharophila voucher UTEX 247 internal transcribed spacer 1, partial sequence; 5.8S ribosomal RNA gene, complete sequence; and internal transcribed spacer 2, partial sequence	492	492	91%	2e-134	95.48%	EF120783.1

Description	Max Score	Total Score	Query Cover	E value	Per. Ident	Accession
Chloroidium saccharophilum voucher Herbarium Tuerk51488 internal transcribed spacer 1, partial sequence; 5.8S ribosomal RNA gene, complete sequence; and internal transcribed spacer 2, partial sequence	490	490	84%	6e-134	97.89%	KF907674.1
Chloroidium saccharophilum voucher Herbarium Tuerk51481 internal transcribed spacer 1, partial sequence; 5.8S ribosomal RNA gene, complete sequence; and internal transcribed spacer 2, partial sequence	490	490	84%	6e-134	97.89%	KF907673.1
Chloroidium saccharophilum voucher Herbarium Tuerk51492 internal transcribed spacer 1, partial sequence; 5.8S ribosomal RNA gene, complete sequence; and internal transcribed spacer 2, partial sequence	484	484	84%	3e-132	97.54%	KF907675.1
Chloroidium saccharophilum voucher Herbarium Tuerk51480 internal transcribed spacer 1, partial sequence; 5.8S ribosomal RNA gene, complete sequence; and internal transcribed spacer 2, partial sequence	484	484	84%	3e-132	97.54%	KF907672.1
'Chlorella' saccharophila voucher UTEX 2911 internal transcribed spacer 1, partial sequence; 5.8S ribosomal RNA gene, complete sequence; and internal transcribed spacer 2, partial sequence	464	464	81%	3e-126	97.45%	EF120782.1
Chloroidium sp. S4MWC-51 internal transcribed spacer 1, 5.8S ribosomal RNA gene, and internal transcribed spacer 2, complete sequence	455	455	78%	2e-123	97.74%	MK005103.1
Uncultured Chloroidium isolate A026 internal transcribed spacer 1, partial sequence; 5.8S ribosomal RNA gene, complete sequence; and internal transcribed spacer 2, partial sequence	418	418	90%	3e-112	91.50%	KP314510.1
'Chlorella' saccharophila voucher UTEX 2469 internal transcribed spacer 1, partial sequence; 5.8S ribosomal RNA gene, complete sequence; and internal transcribed spacer 2, partial sequence	403	403	70%	8e-108	97.47%	EF120784.1
Chloroidium saccharophilum strain KMMCC 192 18S ribosomal RNA gene, internal transcribed spacer 1, 5.8S ribosomal RNA gene, internal transcribed spacer 2, and 28S ribosomal RNA gene, region	346	346	58%	1e-90	98.48%	JQ315767.1
Uncultured alga isolate ASV_573_A 5.8S ribosomal RNA gene, partial sequence; internal transcribed spacer 2, complete sequence; and large subunit ribosomal RNA gene, partial sequence	276	276	100%	2e-69	81.84%	ON119385.1
Chloroidium viscosum strain MG-2 small subunit ribosomal RNA gene, partial sequence; internal transcribed spacer 1 and 5.8S ribosomal RNA gene, complete sequence; and internal transcribed spacer 2, partial sequence	244	244	94%	5e-60	80.94%	MH551496.1

Description	Max Score	Total Score	Query Cover	E value	Per. Ident	Accession
Chloroidium angustoellipsoideum voucher VancurovaA356 internal transcribed spacer 1, partial sequence; 5.8S ribosomal RNA gene and internal transcribed spacer 2, complete sequence; and large subunit ribosomal RNA gene, partial sequence	244	244	100%	5e-60	81.45%	MH415249.1
uncultured fungus genomic DNA sequence contains ITS1	239	239	100%	2e-58	79.94%	OW836154.1
Chloroidium lichenum strain BII/C-1 small subunit ribosomal RNA gene, partial sequence; internal transcribed spacer 1 and 5.8S ribosomal RNA gene, complete sequence; and internal transcribed spacer 2, partial sequence	235	235	76%	3e-57	83.96%	MH551507.1
Chloroidium lichenum strain MG-4 small subunit ribosomal RNA gene, partial sequence; internal transcribed spacer 1 and 5.8S ribosomal RNA gene, complete sequence; and internal transcribed spacer 2, partial sequence	235	235	76%	3e-57	83.96%	MH551506.1
Chloroidium lichenum strain BII/B-9 small subunit ribosomal RNA gene, partial sequence; internal transcribed spacer 1 and 5.8S ribosomal RNA gene, complete sequence; and internal transcribed spacer 2, partial sequence	235	235	76%	3e-57	83.96%	MH551505.1
Uncultured Chlorella clone CM-E-A25 internal transcribed spacer 1, partial sequence; 5.8S ribosomal RNA gene, complete sequence; and internal transcribed spacer 2, partial sequence	235	235	76%	3e-57	83.96%	KJ489091.1
Chlorella angustoellipsoidea partial 18S rRNA gene, ITS1, 5.8S rRNA, ITS2 and partial 28S rRNA gene, strain SAG 2115	235	235	76%	3e-57	83.96%	FM946019.1
Chlorella angustoellipsoidea partial 18S rRNA gene, ITS1, 5.8S rRNA, ITS2 and partial 28S rRNA gene, strain SAG 2144	235	235	76%	3e-57	83.96%	FM946020.1
Chloroidium lichenum strain SAG 2293 18S ribosomal RNA gene, internal transcribed spacer 1, 5.8S ribosomal RNA gene, internal transcribed spacer 2, and 28S ribosomal RNA gene, region	231	231	53%	4e-56	90.50%	MH551510.1
Chloroidium lichenum strain SAG 2294 18S ribosomal RNA gene, internal transcribed spacer 1, 5.8S ribosomal RNA gene, internal transcribed spacer 2, and 28S ribosomal RNA gene, region	231	231	53%	4e-56	90.50%	MH551509.1
Chloroidium sp. LV-2018 voucher VancurovaA503 small subunit ribosomal RNA gene, partial sequence; internal transcribed spacer 1, 5.8S ribosomal RNA gene, and internal transcribed spacer 2, complete sequence; and large subunit ribosomal RNA gene, partial sequence	231	305	65%	4e-56	90.50%	MH415295.1

Description	Max Score	Total Score	Query Cover	E value	Per. Ident	Accession
Chloroidium sp. LV-2018 voucher VancurovaA318 small subunit ribosomal RNA gene, partial sequence; internal transcribed spacer 1 and 5.8S ribosomal RNA gene, complete sequence; and internal transcribed spacer 2, partial sequence	231	231	53%	4e-56	90.50%	MH415228.1
Chloroidium angustoellipsoideum voucher VancurovaA421.2 internal transcribed spacer 1, partial sequence; 5.8S ribosomal RNA gene and internal transcribed spacer 2, complete sequence; and large subunit ribosomal RNA gene, partial sequence	228	310	67%	5e-55	90.45%	MH415277.1
Chloroidium ellipsoideum voucher RidkaNZ23 internal transcribed spacer 1, partial sequence; 5.8S ribosomal RNA gene, complete sequence; and internal transcribed spacer 2, partial sequence	228	228	53%	5e-55	90.45%	MH415207.1
Uncultured alga genes for 5.8S rRNA, ITS2, 28S rRNA, partial and complete sequence, note: OTU71	228	228	53%	5e-55	89.62%	LC381752.1
Chloroidium lichenum strain SAG 2141 18S ribosomal RNA gene, internal transcribed spacer 1, 5.8S ribosomal RNA gene, internal transcribed spacer 2, and 28S ribosomal RNA gene, region	226	226	53%	2e-54	90.00%	MH551512.1
Chloroidium orientale strain VCA-45 (Ce2E) small subunit ribosomal RNA gene, partial sequence; internal transcribed spacer 1, 5.8S ribosomal RNA gene, and internal transcribed spacer 2, complete sequence; and large subunit ribosomal RNA gene, partial sequence	226	226	94%	2e-54	80.29%	MZ558750.1
Chloroidium lichenum strain QII/C-5b small subunit ribosomal RNA gene, partial sequence; internal transcribed spacer 1 and 5.8S ribosomal RNA gene, complete sequence; and internal transcribed spacer 2, partial sequence	224	224	53%	6e-54	89.44%	MH551508.1
Chloroidium sp. LV-2018 voucher VancurovaO29 small subunit ribosomal RNA gene, partial sequence; internal transcribed spacer 1, 5.8S ribosomal RNA gene, and internal transcribed spacer 2, complete sequence; and large subunit ribosomal RNA gene, partial sequence	224	301	90%	6e-54	83.59%	MH415404.1
Chloroidium lichenum voucher A114 internal transcribed spacer 1, partial sequence; 5.8S ribosomal RNA gene, complete sequence; and internal transcribed spacer 2, partial sequence	224	224	53%	6e-54	89.89%	OL625124.1
Chloroidium sp. LV-2018 voucher VancurovaO27 internal transcribed spacer 1, partial sequence; 5.8S ribosomal RNA gene and internal transcribed spacer 2, complete sequence; and large subunit ribosomal RNA gene, partial sequence	222	305	67%	2e-53	89.89%	MH415402.1

Description	Max Score	Total Score	Query Cover	E value	Per. Ident	Accession
Chloroidium lichenum strain CCAP 211/33 18S ribosomal RNA gene, internal transcribed spacer 1, 5.8S ribosomal RNA gene, internal transcribed spacer 2, and 28S ribosomal RNA gene, region	219	219	53%	3e-52	89.39%	MH551518.1
Chloroidium lichenum strain SAG 2295 small subunit ribosomal RNA gene, partial sequence; internal transcribed spacer 1 and 5.8S ribosomal RNA gene, complete sequence; and internal transcribed spacer 2, partial sequence	219	219	75%	3e-52	83.21%	MH551517.1
Chloroidium sp. Ru-6-6 small subunit ribosomal RNA gene, partial sequence; internal transcribed spacer 1, 5.8S ribosomal RNA gene, and internal transcribed spacer 2, complete sequence; and large subunit ribosomal RNA gene, partial sequence	219	295	67%	3e-52	89.39%	MH703747.1
Chloroidium sp. SY-1-2-B small subunit ribosomal RNA gene, partial sequence; internal transcribed spacer 1, 5.8S ribosomal RNA gene, and internal transcribed spacer 2, complete sequence; and large subunit ribosomal RNA gene, partial sequence	219	219	53%	3e-52	89.39%	MT991543.1
Chloroidium sp. NN-2-O small subunit ribosomal RNA gene, partial sequence; internal transcribed spacer 1, 5.8S ribosomal RNA gene, and internal transcribed spacer 2, complete sequence; and large subunit ribosomal RNA gene, partial sequence	219	295	67%	3e-52	89.39%	MT991540.1
Chloroidium ellipsoideum genomic DNA containing 18S rRNA gene, ITS1, 5.8S rRNA gene, ITS2, 28S rRNA gene, culture collection CCAP 211/33	219	295	67%	3e-52	89.39%	FR865666.1
Chloroidium ellipsoideum voucher Z7 internal transcribed spacer 1, partial sequence; 5.8S ribosomal RNA gene, complete sequence; and internal transcribed spacer 2, partial sequence	217	217	53%	1e-51	89.33%	OL625378.1
Chloroidium ellipsoideum voucher A689 internal transcribed spacer 1, partial sequence; 5.8S ribosomal RNA gene, complete sequence; and internal transcribed spacer 2, partial sequence	217	217	53%	1e-51	89.33%	OL625330.1
Chloroidium lichenum voucher A67_6 internal transcribed spacer 1, partial sequence; 5.8S ribosomal RNA gene, complete sequence; and internal transcribed spacer 2, partial sequence	217	217	53%	1e-51	89.33%	OL625329.1
Chloroidium lichenum voucher A67_5 internal transcribed spacer 1, partial sequence; 5.8S ribosomal RNA gene and internal transcribed spacer 2, complete sequence; and large subunit ribosomal RNA gene, partial sequence	217	294	68%	1e-51	89.33%	OL625328.1
Chloroidium ellipsoideum voucher A67_4 internal transcribed spacer 1, partial sequence; 5.8S ribosomal RNA gene, complete sequence; and internal transcribed spacer 2, partial sequence	217	217	53%	1e-51	89.33%	OL625327.1

Alignments

Equisetum arvense voucher MN01 5.8S ribosomal RNA gene, partial sequence; internal transcribed spacer 2, complete sequence; and large subunit ribosomal RNA gene, partial sequence

Sequence ID: **OL304248.1** Length: 333 Number of Matches: 1

Range 1: 1 to 333

Score	Expect	Identities	Gaps	Strand	Frame
616 bits(333)	9e-172()	333/333(100%)	0/333(0%)	Plus/Plus	

Features:

```
Query  1    TGCAGAATTCCGTGAATCATCGAGTCTTTGAACGCATATTGCGCCGGAGGCTTCGGTCAA  60
            ||||||||||||||||||||||||||||||||||||||||||||||||||||||||||||
Sbjct  1    TGCAGAATTCCGTGAATCATCGAGTCTTTGAACGCATATTGCGCCGGAGGCTTCGGTCAA  60

Query  61   GGGCATGTCTGCCTCAGCGTCGGTTGACCCCTCTCGTCCAATTCCCTCGGGAAGCAGACG  120
            ||||||||||||||||||||||||||||||||||||||||||||||||||||||||||||
Sbjct  61   GGGCATGTCTGCCTCAGCGTCGGTTGACCCCTCTCGTCCAATTCCCTCGGGAAGCAGACG  120

Query  121  GACCTGGCAGTCCCGCTGGCGACAGCGGGTCTGCTGCAGAACAGACGACGAGCCAGCCGC  180
            ||||||||||||||||||||||||||||||||||||||||||||||||||||||||||||
Sbjct  121  GACCTGGCAGTCCCGCTGGCGACAGCGGGTCTGCTGCAGAACAGACGACGAGCCAGCCGC  180

Query  181  AGCGCCGCTCGATTGGAGGTTTTCTCCGGAAGACATGCCGTCGAGTAGCCTGGGGACCAA  240
            ||||||||||||||||||||||||||||||||||||||||||||||||||||||||||||
Sbjct  181  AGCGCCGCTCGATTGGAGGTTTTCTCCGGAAGACATGCCGTCGAGTAGCCTGGGGACCAA  240

Query  241  CTGCCCGTCCTACCAACGACGGGGGAGCGCGCAAGCCATCCCCCTGACTCTCATTTCGAC  300
            ||||||||||||||||||||||||||||||||||||||||||||||||||||||||||||
Sbjct  241  CTGCCCGTCCTACCAACGACGGGGGAGCGCGCAAGCCATCCCCCTGACTCTCATTTCGAC  300

Query  301  CTGAGTTCAGGCAAGACCACCCGCTCAACTTAA  333
            |||||||||||||||||||||||||||||||||
Sbjct  301  CTGAGTTCAGGCAAGACCACCCGCTCAACTTAA  333
```

Equisetum arvense voucher MN05 5.8S ribosomal RNA gene, partial sequence; internal transcribed spacer 2, complete sequence; and large subunit ribosomal RNA gene, partial sequence

Sequence ID: **OL304249.1** Length: 335 Number of Matches: 1

Range 1: 1 to 335

Score	Expect	Identities	Gaps	Strand	Frame
606 bits(328)	5e-169()	333/335(99%)	2/335(0%)	Plus/Plus	

Features:

```
Query  1    TGCAGAATTCCGTGAATCATCGAGTCTTTGAACGCATATTGCGCCGGAGGCTTCGGTCAA  60
            ||||||||||||||||||||||||||||||||||||||||||||||||||||||||||||
Sbjct  1    TGCAGAATTCCGTGAATCATCGAGTCTTTGAACGCATATTGCGCCGGAGGCTTCGGTCAA  60

Query  61   GGGCATGTCTGCCTCAGCGTCGGTTGACCCCTCTCGTCCAATTCCCTCG-GGAAGCAGAC  119
            ||||||||||||||||||||||||||||||||||||||||||||||||| ||||||||||
Sbjct  61   GGGCATGTCTGCCTCAGCGTCGGTTGACCCCTCTCGTCCAATTCCCTCGAGGAAGCAGAC  120

Query  120  GGACCTGGCAGTCCCGCTGGCGACAGCGGGTCTGCTGCAGAACAGACGACGAGCCAGCCG  179
            ||||||||||||||||||||||||||||||||||||||||||||||||||||||||||||
Sbjct  121  GGACCTGGCAGTCCCGCTGGCGACAGCGGGTCTGCTGCAGAACAGACGACGAGCCAGCCG  180

Query  180  CAGCGCCGCTCGATTGG-AGGTTTTCTCCGGAAGACATGCCGTCGAGTAGCCTGGGGACC  238
            ||||||||||||||||| ||||||||||||||||||||||||||||||||||||||||||
Sbjct  181  CAGCGCCGCTCGATTGGTAGGTTTTCTCCGGAAGACATGCCGTCGAGTAGCCTGGGGACC  240

Query  239  AACTGCCCGTCCTACCAACGACGGGGGAGCGCGCAAGCCATCCCCCTGACTCTCATTTCG  298
            ||||||||||||||||||||||||||||||||||||||||||||||||||||||||||||
Sbjct  241  AACTGCCCGTCCTACCAACGACGGGGGAGCGCGCAAGCCATCCCCCTGACTCTCATTTCG  300

Query  299  ACCTGAGTTCAGGCAAGACCACCCGCTCAACTTAA  333
            |||||||||||||||||||||||||||||||||||
Sbjct  301  ACCTGAGTTCAGGCAAGACCACCCGCTCAACTTAA  335
```

uncultured fungus genomic DNA sequence contains ITS1

Sequence ID: **OW836430.1** Length: 358 Number of Matches: 1

Range 1: 24 to 358

Score	Expect	Identities	Gaps	Strand	Frame

601 bits(325)	2e-167()	332/335(99%)	2/335(0%)	Plus/Plus	

Features:

```
Query  1    TGCAGAATTCCGTGAATCATCGAGTCTTTGAACGCATATTGCGCCGGAGGCTTCGGTCAA  60
            ||||||||||||||||||||||||||||||||||||||||||||| ||||||||||||||
Sbjct  24   TGCAGAATTCCGTGAATCATCGAGTCTTTGAACGCATATTGCGCCCGAGGCTTCGGTCAA  83

Query  61   GGGCATGTCTGCCTCAGCGTCGGTTGACCCCTCTCGTCCAATTCCCTCG-GGAAGCAGAC  119
            ||||||||||||||||||||||||||||||||||||||||||||||||| ||||||||||
Sbjct  84   GGGCATGTCTGCCTCAGCGTCGGTTGACCCCTCTCGTCCAATTCCCTCGTGGAAGCAGAC  143

Query  120  GGACCTGGCAGTCCCGCTGGCGACAGCGGGTCTGCTGCAGAACAGACGACGAGCCAGCCG  179
            ||||||||||||||||||||||||||||||||||||||||||||||||||||||||||||
Sbjct  144  GGACCTGGCAGTCCCGCTGGCGACAGCGGGTCTGCTGCAGAACAGACGACGAGCCAGCCG  203

Query  180  CAGCGCCGCTCGATTGG-AGGTTTTCTCCGGAAGACATGCCGTCGAGTAGCCTGGGGACC  238
            ||||||||||||||||| ||||||||||||||||||||||||||||||||||||||||||
Sbjct  204  CAGCGCCGCTCGATTGGTAGGTTTTCTCCGGAAGACATGCCGTCGAGTAGCCTGGGGACC  263

Query  239  AACTGCCCGTCCTACCAACGACGGGGGAGCGCGCAAGCCATCCCCCTGACTCTCATTTCG  298
            ||||||||||||||||||||||||||||||||||||||||||||||||||||||||||||
Sbjct  264  AACTGCCCGTCCTACCAACGACGGGGGAGCGCGCAAGCCATCCCCCTGACTCTCATTTCG  323

Query  299  ACCTGAGTTCAGGCAAGACCACCCGCTCAACTTAA  333
            |||||||||||||||||||||||||||||||||||
Sbjct  324  ACCTGAGTTCAGGCAAGACCACCCGCTCAACTTAA  358
```

Equisetum arvense voucher MN06 5.8S ribosomal RNA gene, partial sequence; internal transcribed spacer 2, complete sequence; and large subunit ribosomal RNA gene, partial sequence

Sequence ID: **OL304250.1** Length: 335 Number of Matches: 1

Range 1: 1 to 335

Score	Expect	Identities	Gaps	Strand	Frame
601 bits(325)	2e-167()	332/335(99%)	2/335(0%)	Plus/Plus	

Features:

```
Query  1    TGCAGAATTCCGTGAATCATCGAGTCTTTGAACGCATATTGCGCCGGAGGCTTCGGTCAA  60
            ||||||||||||||||||||||||||||||||||||||||||||| ||||||||||||||
Sbjct  1    TGCAGAATTCCGTGAATCATCGAGTCTTTGAACGCATATTGCGCCCGAGGCTTCGGTCAA  60

Query  61   GGGCATGTCTGCCTCAGCGTCGGTTGACCCCTCTCGTCCAATTCCCTCG-GGAAGCAGAC  119
            ||||||||||||||||||||||||||||||||||||||||||||||||| ||||||||||
Sbjct  61   GGGCATGTCTGCCTCAGCGTCGGTTGACCCCTCTCGTCCAATTCCCTCGTGGAAGCAGAC  120

Query  120  GGACCTGGCAGTCCCGCTGGCGACAGCGGGTCTGCTGCAGAACAGACGACGAGCCAGCCG  179
            ||||||||||||||||||||||||||||||||||||||||||||||||||||||||||||
Sbjct  121  GGACCTGGCAGTCCCGCTGGCGACAGCGGGTCTGCTGCAGAACAGACGACGAGCCAGCCG  180

Query  180  CAGCGCCGCTCGATTGG-AGGTTTTCTCCGGAAGACATGCCGTCGAGTAGCCTGGGGACC  238
            ||||||||||||||||| ||||||||||||||||||||||||||||||||||||||||||
Sbjct  181  CAGCGCCGCTCGATTGGAAGGTTTTCTCCGGAAGACATGCCGTCGAGTAGCCTGGGGACC  240

Query  239  AACTGCCCGTCCTACCAACGACGGGGGAGCGCGCAAGCCATCCCCCTGACTCTCATTTCG  298
            ||||||||||||||||||||||||||||||||||||||||||||||||||||||||||||
Sbjct  241  AACTGCCCGTCCTACCAACGACGGGGGAGCGCGCAAGCCATCCCCCTGACTCTCATTTCG  300

Query  299  ACCTGAGTTCAGGCAAGACCACCCGCTCAACTTAA  333
            |||||||||||||||||||||||||||||||||||
Sbjct  301  ACCTGAGTTCAGGCAAGACCACCCGCTCAACTTAA  335
```

Chloroidium sp. FACHB-2422 small subunit ribosomal RNA gene, partial sequence; internal transcribed spacer 1, 5.8S ribosomal RNA gene, and internal transcribed spacer 2, complete sequence; and large subunit ribosomal RNA gene, partial sequence

Sequence ID: **MT006211.1** Length: 3787 Number of Matches: 1

Range 1: 2888 to 3222

Score	Expect	Identities	Gaps	Strand	Frame
601 bits(325)	2e-167()	332/335(99%)	2/335(0%)	Plus/Plus	

Features:

```
Query  1     TGCAGAATTCCGTGAATCATCGAGTCTTTGAACGCATATTGCGCCGGAGGCTTCGGTCAA  60
             ||||||||||||||||||||||||||||||||||||||||||||| ||||||||||||||
Sbjct  2888  TGCAGAATTCCGTGAATCATCGAGTCTTTGAACGCATATTGCGCCCGAGGCTTCGGTCAA  2947

Query  61    GGGCATGTCTGCCTCAGCGTCGGTTGACCCCTCTCGTCCAATTCCCTCG-GGAAGCAGAC  119
             ||||||||||||||||||||||||||||||||||||||||||||||||| ||||||||||
```

```
Sbjct  2948  GGGCATGTCTGCCTCAGCGTCGGTTGACCCCTCTCGTCCAATTCCCTCGTGGAAGCAGAC  3007

Query  120   GGACCTGGCAGTCCCGCTGGCGACAGCGGGTCTGCTGCAGAACAGACGACGAGCCAGCCG  179
             ||||||||||||||||||||||||||||||||||||||||||||||||||||||||||||
Sbjct  3008  GGACCTGGCAGTCCCGCTGGCGACAGCGGGTCTGCTGCAGAACAGACGACGAGCCAGCCG  3067

Query  180   CAGCGCCGCTCGATTGG-AGGTTTTCTCCGGAAGACATGCCGTCGAGTAGCCTGGGGACC  238
             ||||||||||||||||| ||||||||||||||||||||||||||||||||||||||||||
Sbjct  3068  CAGCGCCGCTCGATTGGTAGGTTTTCTCCGGAAGACATGCCGTCGAGTAGCCTGGGGACC  3127

Query  239   AACTGCCCGTCCTACCAACGACGGGGGAGCGCGCAAGCCATCCCCCTGACTCTCATTTCG  298
             ||||||||||||||||||||||||||||||||||||||||||||||||||||||||||||
Sbjct  3128  AACTGCCCGTCCTACCAACGACGGGGGAGCGCGCAAGCCATCCCCCTGACTCTCATTTCG  3187

Query  299   ACCTGAGTTCAGGCAAGACCACCCGCTCAACTTAA  333
             |||||||||||||||||||||||||||||||||||
Sbjct  3188  ACCTGAGTTCAGGCAAGACCACCCGCTCAACTTAA  3222
```


BLAST is a registered trademark of the National Library of Medicine

Support center Mailing list YouTube

- National Library Of Medicine
- National Institutes Of Health
- U.S. Department of Health & Human Services
- USA.gov

NCBI

National Center for Biotechnology Information, U.S. National Library of Medicine 8600 Rockville Pike, Bethesda MD, 20894 USA

Policies and Guidelines | Contact

HHS Vulnerability Disclosure
