# Supplementary material for: A Multi-Pronged Technique for Identifying Equisetum palustre and Equisetum arvense—Combining HPTLC, HPLC-ESI-MS/MS and Optimized DNA Barcoding Techniques
Source: Plants (Basel). 2022 Sep 28;11(19):2562. doi: 10.3390/plants11192562 (PMC9573248; doi:10.3390/plants11192562)
Supplement: Supplementary file 1 [file plants-11-02562-s001.zip › plants-1932149 supplementary/Horsetail Supplementary Figures/Figure S10.pdf]

BLAST Results

Questions/comments

Job title: DQ377155.1

**RID**	FD4CKFVU016 (Expires on 08-14 01:44 am)		
**Query ID**	lcl|Query_53701	**Database Name**	nt
**Description**	None	**Description**	Nucleotide collection (nt)
**Molecule type**	dna	**Program**	BLASTN 2.13.0+
**Query Length**	294		

Graphic Summary

Distribution of the top 100 Blast Hits on 100 subject sequences

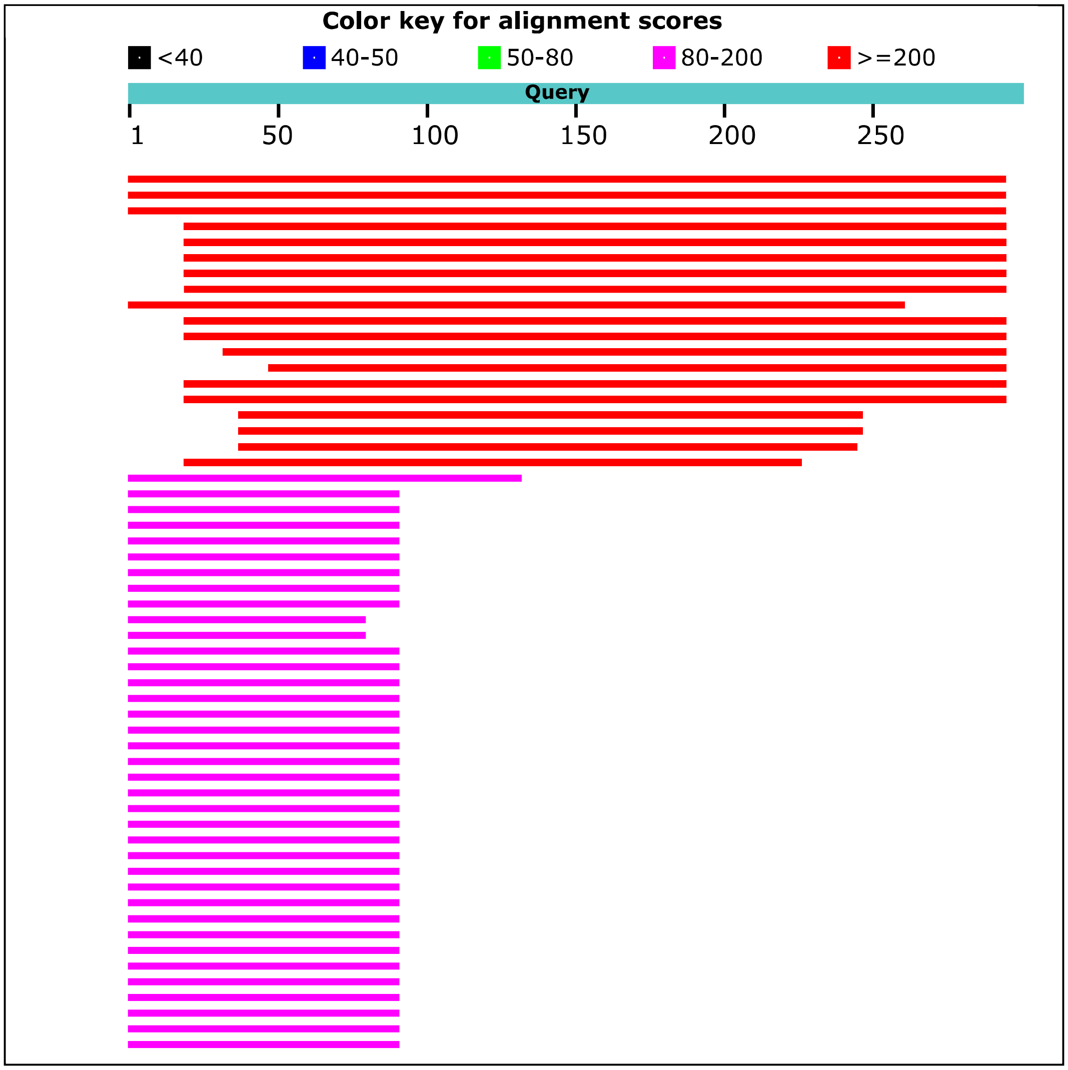

Descriptions

Sequences producing significant alignments:

Description	Max Score	Total Score	Query Cover	E value	Per. Ident	Accession
Equisetum variegatum subsp. variegatum internal transcribed spacer 1, partial sequence	544	544	100%	4e-150	100.00%	DQ377155.1
Equisetum ramosissimum isolate BPTPS123 small subunit ribosomal RNA gene, partial sequence; internal transcribed spacer 1, 5.8S ribosomal RNA gene, and internal transcribed spacer 2, complete sequence; and large subunit ribosomal RNA gene, partial sequence	510	510	100%	4e-140	97.96%	ON685433.1
Equisetum ramosissimum isolate BPTPS056 small subunit ribosomal RNA gene, partial sequence; internal transcribed spacer 1, 5.8S ribosomal RNA gene, and internal transcribed spacer 2, complete sequence; and large subunit ribosomal RNA gene, partial sequence	494	494	100%	4e-135	96.95%	OM670239.1
Equisetum variegatum 18S ribosomal RNA gene, partial sequence; internal transcribed spacer 1, complete sequence; and 5.8S ribosomal RNA gene, partial sequence	488	488	93%	2e-133	98.55%	EU328340.1
Equisetum variegatum voucher B. Hoshizaki 592 18S ribosomal RNA gene, partial sequence; internal transcribed spacer 1, complete sequence; and 5.8S ribosomal RNA gene, partial sequence	484	484	93%	2e-132	98.18%	EU372664.1
Equisetum x ferrissii 18S ribosomal RNA gene, partial sequence; internal transcribed spacer 1, complete sequence; and 5.8S ribosomal RNA gene, partial sequence	466	466	93%	8e-127	96.73%	EU328338.1
Equisetum laevigatum 18S ribosomal RNA gene, partial sequence; internal transcribed spacer 1, complete sequence; and 5.8S ribosomal RNA gene, partial sequence	460	460	93%	4e-125	96.00%	EU328341.1
Equisetum hyemale subsp. affine 18S ribosomal RNA gene, partial sequence; internal transcribed spacer 1, complete sequence; and 5.8S ribosomal RNA gene, partial sequence	444	444	93%	4e-120	95.27%	EU328339.1
Equisetum hyemale subsp. hyemale internal transcribed spacer 1, partial sequence	431	431	88%	3e-116	96.54%	DQ377151.1
Equisetum hyemale subsp. affine 18S ribosomal RNA gene, partial sequence; internal transcribed spacer 1, complete sequence; and 5.8S ribosomal RNA gene, partial sequence	424	424	93%	5e-114	93.82%	EU328336.1
Equisetum variegatum voucher B. Hoshizaki 203 18S ribosomal RNA gene, partial sequence; internal transcribed spacer 1, complete sequence; and 5.8S ribosomal RNA gene, partial sequence	422	422	93%	2e-113	93.82%	EU372665.1

Description	Max Score	Total Score	Query Cover	E value	Per. Ident	Accession
Equisetum ramosissimum 18S ribosomal RNA gene, partial sequence; internal transcribed spacer 1, 5.8S ribosomal RNA gene and internal transcribed spacer 2, complete sequence; and 26S ribosomal RNA gene, partial sequence	418	418	89%	2e-112	95.42%	AF448794.1
Equisetum hyemale genes for ITS1, 5.8S rRNA, ITS2, partial sequence, bio_material: KB:NIBR0000051239	401	401	84%	2e-107	95.95%	AB817880.1
Equisetum scirpoides voucher P. Hammond s.n 18S ribosomal RNA gene, partial sequence; internal transcribed spacer 1, complete sequence; and 5.8S ribosomal RNA gene, partial sequence	385	385	93%	2e-102	90.55%	EU372663.1
Equisetum myriochaetum 18S ribosomal RNA gene, partial sequence; internal transcribed spacer 1, complete sequence; and 5.8S ribosomal RNA gene, partial sequence	372	372	93%	2e-98	90.55%	EU328337.1
Equisetum x meridionale internal transcribed spacer 1, partial sequence	350	350	71%	9e-92	96.68%	DQ377156.1
Equisetum x moorei internal transcribed spacer 1, partial sequence	342	342	71%	1e-89	96.17%	DQ377157.1
Equisetum ramosissimum subsp. ramosissimum internal transcribed spacer 1, partial sequence	331	331	70%	3e-86	95.65%	DQ377153.1
Equisetum ramosissimum 18S ribosomal RNA gene, partial sequence; internal transcribed spacer 1, complete sequence; and 5.8S ribosomal RNA gene, partial sequence	292	292	70%	1e-74	91.75%	EU328343.1
Equisetum telmateia isolate DM233 small subunit ribosomal RNA gene, internal transcribed spacer 1, 5.8S ribosomal RNA gene, internal transcribed spacer 2, and large subunit ribosomal RNA gene, region	193	193	44%	2e-44	93.13%	MT784095.1
Pleopeltis macrocarpa voucher H. Shang SG1191 (PE) small subunit ribosomal RNA gene, internal transcribed spacer 1, 5.8S ribosomal RNA gene, internal transcribed spacer 2, and large subunit ribosomal RNA gene, complete sequence	121	121	30%	7e-23	91.21%	MZ361029.1
Microsorum steerei voucher R. Wei WR0620 (PE) small subunit ribosomal RNA gene, internal transcribed spacer 1, 5.8S ribosomal RNA gene, internal transcribed spacer 2, and large subunit ribosomal RNA gene, complete sequence	121	121	30%	7e-23	91.21%	MZ361020.1
Leptochilus pteropus voucher R. Wei WR0281 (PE) small subunit ribosomal RNA gene, internal transcribed spacer 1, 5.8S ribosomal RNA gene, internal transcribed spacer 2, and large subunit ribosomal RNA gene, complete sequence	121	121	30%	7e-23	91.21%	MZ361018.1

Description	Max Score	Total Score	Query Cover	E value	Per. Ident	Accession
Leptochilus insignis voucher R. Wei & al., 440 (PE) small subunit ribosomal RNA gene, internal transcribed spacer 1, 5.8S ribosomal RNA gene, internal transcribed spacer 2, and large subunit ribosomal RNA gene, complete sequence	121	121	30%	7e-23	91.21%	MZ361017.1
Campyloneurum sphenodes voucher P.C. Standley 34246 (PE) small subunit ribosomal RNA gene, internal transcribed spacer 1, 5.8S ribosomal RNA gene, internal transcribed spacer 2, and large subunit ribosomal RNA gene, complete sequence	121	121	30%	7e-23	91.21%	MZ360986.1
Bosmania membranacea subsp. carinata voucher R. Wei WR0410 (PE) small subunit ribosomal RNA gene, internal transcribed spacer 1, 5.8S ribosomal RNA gene, internal transcribed spacer 2, and large subunit ribosomal RNA gene, complete sequence	121	121	30%	7e-23	91.21%	MZ360985.1
Bosmania membranacea var. membranacea voucher R. Wei WR0368 (PE) small subunit ribosomal RNA gene, internal transcribed spacer 1, 5.8S ribosomal RNA gene, internal transcribed spacer 2, and large subunit ribosomal RNA gene, complete sequence	121	121	30%	7e-23	91.21%	MZ360984.1
Pellaea breweri isolate CBP242 18S ribosomal RNA gene, partial sequence; and internal transcribed spacer 1, 5.8S ribosomal RNA gene, and internal transcribed spacer 2, complete sequence	117	117	30%	1e-21	90.11%	MF963964.1
Hovenkampia schimperiana voucher B. Liu CPG27233 (PE) small subunit ribosomal RNA gene, internal transcribed spacer 1, 5.8S ribosomal RNA gene, internal transcribed spacer 2, and large subunit ribosomal RNA gene, complete sequence	117	117	26%	1e-21	93.75%	MZ361042.1
Microsorum punctatum voucher Mt. Shiwan Exp. 148 (PE) small subunit ribosomal RNA gene, internal transcribed spacer 1, 5.8S ribosomal RNA gene, internal transcribed spacer 2, and large subunit ribosomal RNA gene, complete sequence	117	117	26%	1e-21	93.75%	MZ361019.1
Lellingeria brevistipes voucher P. Labiak 2007 (PE) small subunit ribosomal RNA gene, internal transcribed spacer 1, 5.8S ribosomal RNA gene, internal transcribed spacer 2, and large subunit ribosomal RNA gene, complete sequence	117	117	30%	1e-21	90.11%	MZ361001.1
Oreogrammitis congener voucher R. Wei & al. 523 (PE) small subunit ribosomal RNA gene, internal transcribed spacer 1, 5.8S ribosomal RNA gene, internal transcribed spacer 2, and large subunit ribosomal RNA gene, complete sequence	117	117	30%	1e-21	90.11%	MZ360999.1

Description	Max Score	Total Score	Query Cover	E value	Per. Ident	Accession
Oreogrammitis sp. RW-2021b voucher H.M. Liu B294 (PE) small subunit ribosomal RNA gene, internal transcribed spacer 1, 5.8S ribosomal RNA gene, internal transcribed spacer 2, and large subunit ribosomal RNA gene, complete sequence	117	117	30%	1e-21	90.11%	MZ360998.1
Oreogrammitis dorsipila voucher J.S. Kang 1201-11 (PE) small subunit ribosomal RNA gene, internal transcribed spacer 1, 5.8S ribosomal RNA gene, internal transcribed spacer 2, and large subunit ribosomal RNA gene, complete sequence	117	117	30%	1e-21	90.11%	MZ360997.1
Prosaptia venulosa voucher R. Wei 246 (PE) small subunit ribosomal RNA gene, internal transcribed spacer 1, 5.8S ribosomal RNA gene, internal transcribed spacer 2, and large subunit ribosomal RNA gene, complete sequence	117	117	30%	1e-21	90.11%	MZ360989.1
Thylacopteris papillosa voucher R. Wei 345 (PE) small subunit ribosomal RNA gene, internal transcribed spacer 1, 5.8S ribosomal RNA gene, internal transcribed spacer 2, and large subunit ribosomal RNA gene, complete sequence	115	115	30%	3e-21	90.11%	MZ361053.1
Serpocaulon loriceum voucher R.M. Rueda & al. 14546 (PE) small subunit ribosomal RNA gene, partial sequence; and internal transcribed spacer 1, 5.8S ribosomal RNA gene, internal transcribed spacer 2, and large subunit ribosomal RNA gene, complete sequence	115	115	30%	3e-21	90.11%	MZ361048.1
Selliguea metacoela voucher R. Wei 2925 (PE) small subunit ribosomal RNA gene, internal transcribed spacer 1, 5.8S ribosomal RNA gene, internal transcribed spacer 2, and large subunit ribosomal RNA gene, complete sequence	115	115	30%	3e-21	90.11%	MZ361045.1
Selliguea albidoglauca voucher X.C. Zhang 7609 (PE) small subunit ribosomal RNA gene, internal transcribed spacer 1, 5.8S ribosomal RNA gene, internal transcribed spacer 2, and large subunit ribosomal RNA gene, complete sequence	115	115	30%	3e-21	90.11%	MZ361043.1
Pyrrosia penangiana voucher R. Wei 3014 (PE) small subunit ribosomal RNA gene, internal transcribed spacer 1, 5.8S ribosomal RNA gene, internal transcribed spacer 2, and large subunit ribosomal RNA gene, complete sequence	115	115	30%	3e-21	90.11%	MZ361041.1
Pyrrosia angustata voucher Q. Wei wq004 (PE) small subunit ribosomal RNA gene, internal transcribed spacer 1, 5.8S ribosomal RNA gene, internal transcribed spacer 2, and large subunit ribosomal RNA gene, complete sequence	115	115	30%	3e-21	90.11%	MZ361040.1

Description	Max Score	Total Score	Query Cover	E value	Per. Ident	Accession
Polypodium vulgare voucher B. Liu 28227 (PE) small subunit ribosomal RNA gene, internal transcribed spacer 1, 5.8S ribosomal RNA gene, internal transcribed spacer 2, and large subunit ribosomal RNA gene, complete sequence	115	115	30%	3e-21	90.11%	MZ361039.1
Polypodium sibiricum voucher Zhang 7312 (PE) small subunit ribosomal RNA gene, internal transcribed spacer 1, 5.8S ribosomal RNA gene, internal transcribed spacer 2, and large subunit ribosomal RNA gene, complete sequence	115	115	30%	3e-21	90.11%	MZ361038.1
Polypodium rhodopleuron voucher A.M. Evans & al. 2871 (PE) small subunit ribosomal RNA gene, internal transcribed spacer 1, 5.8S ribosomal RNA gene, internal transcribed spacer 2, and large subunit ribosomal RNA gene, complete sequence	115	115	30%	3e-21	90.11%	MZ361037.1
Polypodium arcanum voucher M. Sundue & al. 3554 (PE) small subunit ribosomal RNA gene, internal transcribed spacer 1, 5.8S ribosomal RNA gene, internal transcribed spacer 2, and large subunit ribosomal RNA gene, complete sequence	115	115	30%	3e-21	90.11%	MZ361036.1
Goniophlebium niponicum voucher H.M. Liu A305 (PE) small subunit ribosomal RNA gene, internal transcribed spacer 1, 5.8S ribosomal RNA gene, internal transcribed spacer 2, and large subunit ribosomal RNA gene, complete sequence	115	115	30%	3e-21	90.11%	MZ361035.1
Selliguea griffithiana voucher X.C. Zhang 9580 (PE) small subunit ribosomal RNA gene, internal transcribed spacer 1, 5.8S ribosomal RNA gene, internal transcribed spacer 2, and large subunit ribosomal RNA gene, complete sequence	115	115	30%	3e-21	90.11%	MZ361025.1
Selliguea crenatopinnata voucher X.C. Zhang 6242 (PE) small subunit ribosomal RNA gene, internal transcribed spacer 1, 5.8S ribosomal RNA gene, internal transcribed spacer 2, and large subunit ribosomal RNA gene, complete sequence	115	115	30%	3e-21	90.11%	MZ361024.1
Selliguea chenopus voucher X.C. Zhang 6259 (PE) small subunit ribosomal RNA gene, internal transcribed spacer 1, 5.8S ribosomal RNA gene, internal transcribed spacer 2, and large subunit ribosomal RNA gene, complete sequence	115	115	30%	3e-21	90.11%	MZ361023.1
Microgramma vacciniifolia voucher C.X. Li RS281 small subunit ribosomal RNA gene, internal transcribed spacer 1, 5.8S ribosomal RNA gene, internal transcribed spacer 2, and large subunit ribosomal RNA gene, complete sequence	115	115	30%	3e-21	90.11%	MZ361014.1

Description	Max Score	Total Score	Query Cover	E value	Per. Ident	Accession
Leptochilus macrophyllus voucher R. Wei & al., 441 (PE) small subunit ribosomal RNA gene, internal transcribed spacer 1, 5.8S ribosomal RNA gene, internal transcribed spacer 2, and large subunit ribosomal RNA gene, complete sequence	115	115	30%	3e-21	90.11%	MZ361009.1
Goniophlebium formosanum voucher X.C. Zhang 9432 (PE) small subunit ribosomal RNA gene, internal transcribed spacer 1, 5.8S ribosomal RNA gene, internal transcribed spacer 2, and large subunit ribosomal RNA gene, complete sequence	115	115	30%	3e-21	90.11%	MZ360995.1
Aglaomorpha willdenowii voucher X.C. Zhang 9062 (PE) small subunit ribosomal RNA gene, internal transcribed spacer 1, 5.8S ribosomal RNA gene, internal transcribed spacer 2, and large subunit ribosomal RNA gene, complete sequence	115	115	30%	3e-21	90.11%	MZ360993.1
Leptochilus ellipticus voucher R. Wei WR0211 (PE) small subunit ribosomal RNA gene, internal transcribed spacer 1, 5.8S ribosomal RNA gene, internal transcribed spacer 2, and large subunit ribosomal RNA gene, complete sequence	115	115	30%	3e-21	90.11%	MZ360987.1
Selliguea capitellata voucher X.C. Zhang 6341 (PE) small subunit ribosomal RNA gene, internal transcribed spacer 1, 5.8S ribosomal RNA gene, internal transcribed spacer 2, and large subunit ribosomal RNA gene, complete sequence	115	115	30%	3e-21	90.11%	MZ360982.1
Selliguea mairei voucher X.C. Zhang 4609 (PE) small subunit ribosomal RNA gene, internal transcribed spacer 1, 5.8S ribosomal RNA gene, internal transcribed spacer 2, and large subunit ribosomal RNA gene, complete sequence	115	115	30%	3e-21	90.11%	MZ360981.1
Selliguea lehmannii voucher X.C. Zhang 5963 (PE) small subunit ribosomal RNA gene, internal transcribed spacer 1, 5.8S ribosomal RNA gene, internal transcribed spacer 2, and large subunit ribosomal RNA gene, complete sequence	115	115	30%	3e-21	90.11%	MZ360980.1
Drynaria heraclea voucher R. Wei 470 (PE) small subunit ribosomal RNA gene, internal transcribed spacer 1, 5.8S ribosomal RNA gene, internal transcribed spacer 2, and large subunit ribosomal RNA gene, complete sequence	115	115	30%	3e-21	90.11%	MZ360979.1
Equisetum sylvaticum 18S ribosomal RNA gene, partial sequence; internal transcribed spacer 1, complete sequence; and 5.8S ribosomal RNA gene, partial sequence	115	115	22%	3e-21	98.46%	EU328342.1

Description	Max Score	Total Score	Query Cover	E value	Per. Ident	Accession
Drepanolejeunea angustifolia voucher Zhu & Wei 20110418-65A small subunit ribosomal RNA gene, partial sequence; internal transcribed spacer 1, 5.8S ribosomal RNA gene, and internal transcribed spacer 2, complete sequence; and large subunit ribosomal RNA gene, partial sequence	113	113	25%	1e-20	94.67%	MH680792.1
Pleopeltis polypodioides voucher H. Shang 28B (PE) small subunit ribosomal RNA gene, internal transcribed spacer 1, 5.8S ribosomal RNA gene, internal transcribed spacer 2, and large subunit ribosomal RNA gene, complete sequence	113	113	30%	1e-20	89.01%	MZ361030.1
Platycerium bifurcatum voucher X.P. Wei 41503 (PE) small subunit ribosomal RNA gene, internal transcribed spacer 1, 5.8S ribosomal RNA gene, internal transcribed spacer 2, and large subunit ribosomal RNA gene, complete sequence	111	111	26%	4e-20	92.50%	MZ361028.1
Niphidium crassifolium voucher WR0621 (PE) small subunit ribosomal RNA gene, internal transcribed spacer 1, 5.8S ribosomal RNA gene, internal transcribed spacer 2, and large subunit ribosomal RNA gene, complete sequence	111	111	30%	4e-20	89.01%	MZ361021.1
Drynaria parishii voucher X.C. Zhang 7946 (PE) small subunit ribosomal RNA gene, internal transcribed spacer 1, 5.8S ribosomal RNA gene, internal transcribed spacer 2, and large subunit ribosomal RNA gene, complete sequence	111	111	26%	4e-20	92.50%	MZ360992.1
Drynaria speciosa voucher R. Wei 508 (PE) small subunit ribosomal RNA gene, internal transcribed spacer 1, 5.8S ribosomal RNA gene, internal transcribed spacer 2, and large subunit ribosomal RNA gene, complete sequence	111	111	26%	4e-20	92.50%	MZ360978.1
Asplenium scolopendrium isolate Copy2 voucher Vogel SCOL-175 Italy internal transcribed spacer 1, partial sequence; 5.8S ribosomal RNA gene, complete sequence; and internal transcribed spacer 2, partial sequence	111	111	30%	4e-20	89.01%	JX475163.1
Asplenium adulterinum isolate Copy1 voucher Vogel ADU-32 Norway internal transcribed spacer 1, partial sequence; 5.8S ribosomal RNA gene, complete sequence; and internal transcribed spacer 2, partial sequence	111	111	30%	4e-20	89.01%	JX475149.1
Scapania undulata Shevock et al. 29009 (GOET) 18S ribosomal RNA gene, partial sequence; internal transcribed spacer 1, 5.8S ribosomal RNA gene, and internal transcribed spacer 2, complete sequence; and 26S ribosomal RNA gene, partial sequence	111	111	30%	4e-20	89.13%	JN631489.1

Description	Max Score	Total Score	Query Cover	E value	Per. Ident	Accession
Scapania undulata Schaefer-Verwimp & Verwimp 25913 (GOET) 18S ribosomal RNA gene, partial sequence; internal transcribed spacer 1, 5.8S ribosomal RNA gene, and internal transcribed spacer 2, complete sequence; and 26S ribosomal RNA gene, partial sequence	111	111	30%	4e-20	89.13%	JN631485.1
Scapania undulata Schaefer-Verwimp & Verwimp 27551 (GOET) 18S ribosomal RNA gene, partial sequence; internal transcribed spacer 1, 5.8S ribosomal RNA gene, and internal transcribed spacer 2, complete sequence; and 26S ribosomal RNA gene, partial sequence	111	111	30%	4e-20	89.13%	JN631484.1
Scapania cuspiduligera Gradstein 7828 (GOET) 18S ribosomal RNA gene, partial sequence; internal transcribed spacer 1, 5.8S ribosomal RNA gene, and internal transcribed spacer 2, complete sequence; and 26S ribosomal RNA gene, partial sequence	111	111	30%	4e-20	89.13%	JN631408.1
Serpocaulon levigatum voucher R. Rojas & G. Ortiz 5315 (PE) small subunit ribosomal RNA gene, internal transcribed spacer 1, 5.8S ribosomal RNA gene, internal transcribed spacer 2, and large subunit ribosomal RNA gene, complete sequence	110	110	30%	2e-19	89.01%	MZ361047.1
Serpocaulon fraxinifolium voucher G. Hatschbach & al. 73838 (PE) small subunit ribosomal RNA gene, internal transcribed spacer 1, 5.8S ribosomal RNA gene, internal transcribed spacer 2, and large subunit ribosomal RNA gene, complete sequence	110	110	30%	2e-19	89.01%	MZ361046.1
Goniophlebium amoenum voucher Z.Y. Li 1897 (PE) small subunit ribosomal RNA gene, internal transcribed spacer 1, 5.8S ribosomal RNA gene, internal transcribed spacer 2, and large subunit ribosomal RNA gene, complete sequence	110	110	30%	2e-19	89.01%	MZ361034.1
Goniophlebium mengtzeense voucher H.M. Liu GX211 (PE) small subunit ribosomal RNA gene, internal transcribed spacer 1, 5.8S ribosomal RNA gene, internal transcribed spacer 2, and large subunit ribosomal RNA gene, complete sequence	110	110	30%	2e-19	89.01%	MZ361033.1
Goniophlebium argutum voucher R. Wei ST465 (PE) small subunit ribosomal RNA gene, internal transcribed spacer 1, 5.8S ribosomal RNA gene, internal transcribed spacer 2, and large subunit ribosomal RNA gene, complete sequence	110	110	30%	2e-19	89.01%	MZ361032.1
Pleurosoriopsis makinoi voucher X.C. Zhang 9478 (PE) small subunit ribosomal RNA gene, internal transcribed spacer 1, 5.8S ribosomal RNA gene, internal transcribed spacer 2, and large subunit ribosomal RNA gene, complete sequence	110	110	30%	2e-19	89.01%	MZ361031.1

Description	Max Score	Total Score	Query Cover	E value	Per. Ident	Accession
Microsorum cuspidatum voucher X.C. Zhang 4918 (PE) small subunit ribosomal RNA gene, internal transcribed spacer 1, 5.8S ribosomal RNA gene, internal transcribed spacer 2, and large subunit ribosomal RNA gene, complete sequence	110	110	30%	2e-19	89.01%	MZ361027.1
Goniophlebium manmeiense voucher X.P. Qi Q061 (PE) small subunit ribosomal RNA gene, internal transcribed spacer 1, 5.8S ribosomal RNA gene, internal transcribed spacer 2, and large subunit ribosomal RNA gene, complete sequence	110	110	30%	2e-19	89.01%	MZ361013.1
Leptochilus decurrens voucher R. Wei WR0267 (PE) small subunit ribosomal RNA gene, internal transcribed spacer 1, 5.8S ribosomal RNA gene, internal transcribed spacer 2, and large subunit ribosomal RNA gene, complete sequence	110	110	30%	2e-19	89.01%	MZ361008.1
Lepisorus schraderi voucher X.C. Zhang 9057 (PE) small subunit ribosomal RNA gene, internal transcribed spacer 1, 5.8S ribosomal RNA gene, internal transcribed spacer 2, and large subunit ribosomal RNA gene, complete sequence	110	110	30%	2e-19	89.01%	MZ361007.1
Lepisorus longifolius voucher R. Wei SIWI4 (PE) small subunit ribosomal RNA gene, internal transcribed spacer 1, 5.8S ribosomal RNA gene, internal transcribed spacer 2, and large subunit ribosomal RNA gene, complete sequence	110	110	30%	2e-19	89.01%	MZ361006.1
Lepisorus clathratus voucher Voucher unknown small subunit ribosomal RNA gene, internal transcribed spacer 1, 5.8S ribosomal RNA gene, internal transcribed spacer 2, and large subunit ribosomal RNA gene, complete sequence	110	110	30%	2e-19	89.01%	MZ361004.1
Lepisorus hederaceus voucher Voucher unknown small subunit ribosomal RNA gene, internal transcribed spacer 1, 5.8S ribosomal RNA gene, internal transcribed spacer 2, and large subunit ribosomal RNA gene, complete sequence	110	110	30%	2e-19	89.01%	MZ361003.1
Selliguea dareiformis voucher R. Wei ST1108 (PE) small subunit ribosomal RNA gene, internal transcribed spacer 1, 5.8S ribosomal RNA gene, internal transcribed spacer 2, and large subunit ribosomal RNA gene, complete sequence	110	110	30%	2e-19	89.01%	MZ361000.1
Goniophlebium persicifolium voucher X.C. Zhang 433 (PE) small subunit ribosomal RNA gene, internal transcribed spacer 1, 5.8S ribosomal RNA gene, internal transcribed spacer 2, and large subunit ribosomal RNA gene, complete sequence	110	110	30%	2e-19	89.01%	MZ360996.1

Description	Max Score	Total Score	Query Cover	E value	Per. Ident	Accession
Lepisorus miyoshianus voucher X.C. Zhang 4511 (PE) small subunit ribosomal RNA gene, internal transcribed spacer 1, 5.8S ribosomal RNA gene, internal transcribed spacer 2, and large subunit ribosomal RNA gene, complete sequence	110	110	30%	2e-19	89.01%	MZ360991.1
Leptochilus henryi voucher X.C. Zhang 7487 (PE) small subunit ribosomal RNA gene, internal transcribed spacer 1, 5.8S ribosomal RNA gene, internal transcribed spacer 2, and large subunit ribosomal RNA gene, complete sequence	110	110	30%	2e-19	89.01%	MZ360988.1
Lepisorus spicatus voucher R. Wei 29 (PE) small subunit ribosomal RNA gene, internal transcribed spacer 1, 5.8S ribosomal RNA gene, internal transcribed spacer 2, and large subunit ribosomal RNA gene, complete sequence	110	110	30%	2e-19	89.01%	MZ360983.1
Adetogramma chrysolepis voucher Alfredo Tupayachi H. 758 (A) small subunit ribosomal RNA gene, internal transcribed spacer 1, 5.8S ribosomal RNA gene, internal transcribed spacer 2, and large subunit ribosomal RNA gene, complete sequence	110	110	30%	2e-19	89.01%	MZ360977.1
Asplenium trichomanes isolate Copy2 voucher Wood s.n. Hawaii internal transcribed spacer 1, partial sequence; 5.8S ribosomal RNA gene, complete sequence; and internal transcribed spacer 2, partial sequence	110	110	32%	2e-19	87.63%	JX475165.1
Dictymia brownii voucher R. Coveny 10858 (PE) small subunit ribosomal RNA gene, internal transcribed spacer 1, 5.8S ribosomal RNA gene, internal transcribed spacer 2, and large subunit ribosomal RNA gene, complete sequence	108	108	26%	6e-19	91.25%	MZ360990.1
Terpsichore aspleniifolia voucher R.M. Rueda & al. 14728 (PE) small subunit ribosomal RNA gene, internal transcribed spacer 1, 5.8S ribosomal RNA gene, internal transcribed spacer 2, and large subunit ribosomal RNA gene, complete sequence	106	106	30%	2e-18	87.91%	MZ361052.1
Selliguea taeniata voucher B.Y. Sun & al. 36 (PE) small subunit ribosomal RNA gene, internal transcribed spacer 1, 5.8S ribosomal RNA gene, internal transcribed spacer 2, and large subunit ribosomal RNA gene, complete sequence	106	106	30%	2e-18	88.17%	MZ361026.1
Micropolypodium sikkimense voucher Z.Y. Li 2763 (PE) small subunit ribosomal RNA gene, internal transcribed spacer 1, 5.8S ribosomal RNA gene, internal transcribed spacer 2, and large subunit ribosomal RNA gene, complete sequence	106	106	30%	2e-18	87.91%	MZ361016.1

Description	Max Score	Total Score	Query Cover	E value	Per. Ident	Accession
Micropolypodium okuboi voucher X.C. Zhang 6000 (PE) small subunit ribosomal RNA gene, internal transcribed spacer 1, 5.8S ribosomal RNA gene, internal transcribed spacer 2, and large subunit ribosomal RNA gene, complete sequence	106	106	30%	2e-18	87.91%	MZ361015.1
Melpomene flabelliformis voucher M. Sundue 3920 (PE) small subunit ribosomal RNA gene, internal transcribed spacer 1, 5.8S ribosomal RNA gene, internal transcribed spacer 2, and large subunit ribosomal RNA gene, complete sequence	106	106	30%	2e-18	88.04%	MZ361012.1
Loxogramme lankokiensis voucher H.M. Liu LHM1938 (PE) small subunit ribosomal RNA gene, internal transcribed spacer 1, 5.8S ribosomal RNA gene, internal transcribed spacer 2, and large subunit ribosomal RNA gene, complete sequence	106	106	26%	2e-18	91.25%	MZ361011.1
Loxogramme avenia voucher R. Wei 2761 (PE) small subunit ribosomal RNA gene, internal transcribed spacer 1, 5.8S ribosomal RNA gene, internal transcribed spacer 2, and large subunit ribosomal RNA gene, complete sequence	106	106	26%	2e-18	91.25%	MZ361010.1
Isoetes echinospora isolate ITV11767 small subunit ribosomal RNA gene, partial sequence; internal transcribed spacer 1, 5.8S ribosomal RNA gene, and internal transcribed spacer 2, complete sequence; and large subunit ribosomal RNA gene, partial sequence	106	106	24%	2e-18	93.15%	MW254978.1

Alignments

Equisetum variegatum subsp. variegatum internal transcribed spacer 1, partial sequence

Sequence ID: **DQ377155.1** Length: 294 Number of Matches: 1

Range 1: 1 to 294

Score	Expect	Identities	Gaps	Strand	Frame
544 bits(294)	4e-150()	294/294(100%)	0/294(0%)	Plus/Plus	

Features:

```
Query  1    TCGTAACAAGGTTTCCGTAGGTGAACCTGCGGAAGGATCATTGTCACACATCCGTGCAAA  60
            ||||||||||||||||||||||||||||||||||||||||||||||||||||||||||||
Sbjct  1    TCGTAACAAGGTTTCCGTAGGTGAACCTGCGGAAGGATCATTGTCACACATCCGTGCAAA  60

Query  61   CCCTGCGAACCGTGGACGTCCCCCAAGGGGGCGGTGGTTGTGGTGCCCGCTTGCGCCCTC  120
            ||||||||||||||||||||||||||||||||||||||||||||||||||||||||||||
Sbjct  61   CCCTGCGAACCGTGGACGTCCCCCAAGGGGGCGGTGGTTGTGGTGCCCGCTTGCGCCCTC  120

Query  121  GCGGGCGTGGGTGCGGTGCGGCGCCCCGTCCCCTCCTCTCGGTCGCTCCGGCGGCCgggg  180
            ||||||||||||||||||||||||||||||||||||||||||||||||||||||||||||
Sbjct  121  GCGGGCGTGGGTGCGGTGCGGCGCCCCGTCCCCTCCTCTCGGTCGCTCCGGCGGCCGGGG  180

Query  181  gggTCGAACCTTCCGCCCCTCCGGGGGCGTGGAGCACTCTTGGAACGCGCACCGCCTGAG  240
            ||||||||||||||||||||||||||||||||||||||||||||||||||||||||||||
Sbjct  181  GGGTCGAACCTTCCGCCCCTCCGGGGGCGTGGAGCACTCTTGGAACGCGCACCGCCTGAG  240

Query  241  ACTCCCCCGCAAGGGGGAACACGAAAAAACGAGAACGACTCTCGGCAACGGATA  294
            ||||||||||||||||||||||||||||||||||||||||||||||||||||||
Sbjct  241  ACTCCCCCGCAAGGGGGAACACGAAAAAACGAGAACGACTCTCGGCAACGGATA  294
```

Equisetum ramosissimum isolate BPTPS123 small subunit ribosomal RNA gene, partial sequence; internal transcribed spacer 1, 5.8S ribosomal RNA gene, and internal transcribed spacer 2, complete sequence; and large subunit ribosomal RNA gene, partial sequence
Sequence ID: **ON685433.1** Length: 3702 Number of Matches: 1
Range 1: 1502 to 1795

Score	Expect	Identities	Gaps	Strand	Frame
510 bits(276)	4e-140()	288/294(98%)	0/294(0%)	Plus/Plus	

Features:

```
Query  1     TCGTAACAAGGTTTCCGTAGGTGAACCTGCGGAAGGATCATTGTCACACATCCGTGCAAA  60
             ||||||||||||||||||||||||||||||||||||||||||||||||||||||||||||
Sbjct  1502  TCGTAACAAGGTTTCCGTAGGTGAACCTGCGGAAGGATCATTGTCACACATCCGTGCAAA  1561

Query  61    CCCTGCGAACCGTGGACGTCCCCCAAGGGGGCGGTGGTTGTGGTGCCCGCTTGCGCCCTC  120
             || ||||||||||||||||||| |||| ||||||||||||||||||||||||||||||| 
Sbjct  1562  CCATGCGAACCGTGGACGTCCCTCAAGAGGGCGGTGGTTGTGGTGCCCGCTTGCGCCCTT  1621

Query  121   GCGGGCGTGGGTGCGGTGCGGCGCCCCGTCCCCTCCTCTCGGTCGCTCCGGCGGCCgggg  180
             |||||||||||||||||||||||||||||||||||||||||| |||||||||||||||||
Sbjct  1622  GCGGGCGTGGGTGCGGTGCGGCGCCCCGTCCCCTCCTCTCGGCCGCTCCGGCGGCCGGGG  1681

Query  181   gggTCGAACCTTCCGCCCCTCCGGGGGCGTGGAGCACTCTTGGAACGCGCACCGCCTGAG  240
             ||||||||||||||||||| ||||||||||||||||||||||||||||||||||||||||
Sbjct  1682  GGGTCGAACCTTCCGCCCCCCCGGGGGCGTGGAGCACTCTTGGAACGCGCACCGCCTGAG  1741

Query  241   ACTCCCCCGCAAGGGGGAACACGAAAAAACGAGAACGACTCTCGGCAACGGATA  294
             ||||||||||||||||||||||||||||||||||||||||||||||||||||||
Sbjct  1742  ACTCCCCCGCAAGGGGGAACACGAAAAAACGAGAACGACTCTCGGCAACGGATA  1795
```

Equisetum ramosissimum isolate BPTPS056 small subunit ribosomal RNA gene, partial sequence; internal transcribed spacer 1, 5.8S ribosomal RNA gene, and internal transcribed spacer 2, complete sequence; and large subunit ribosomal RNA gene, partial sequence
Sequence ID: **OM670239.1** Length: 3631 Number of Matches: 1
Range 1: 1458 to 1752

Score	Expect	Identities	Gaps	Strand	Frame
494 bits(267)	4e-135()	286/295(97%)	1/295(0%)	Plus/Plus	

Features:

```
Query  1     TCGTAACAAGGTTTCCGTAGGTGAACCTGCGGAAGGATCATTGTCACACATCCGTGCAAA  60
             ||||||||||||||||||||||||||||||||||||||||||||||||||||||||||||
Sbjct  1458  TCGTAACAAGGTTTCCGTAGGTGAACCTGCGGAAGGATCATTGTCACACATCCGTGCAAA  1517

Query  61    CCCTGCGAACCGTGGACGTCCCCCAAGGGGGCGGTGGTTGTGGTGCCCGCTTGCGCCCTC  120
             ||||| |||||||||| ||||||||| |||||||||||||||||||||||||| ||||||
Sbjct  1518  CCCTGTGAACCGTGGATGTCCCCCAAAGGGGCGGTGGTTGTGGTGCCCGCTTGTGCCCTC  1577

Query  121   GCGGGCGTGGGTGCGGTGCGGCG-CCCCGTCCCCTCCTCTCGGTCGCTCCGGCGGCCggg  179
             ||||||||||||| ||||||||| |||| ||||||||| |||||||||||||||||||||
Sbjct  1578  GCGGGCGTGGGTGTGGTGCGGCGCCCCCTTCCCCTCCTTTCGGTCGCTCCGGCGGCCGGG  1637

Query  180   ggggTCGAACCTTCCGCCCCTCCGGGGGCGTGGAGCACTCTTGGAACGCGCACCGCCTGA  239
             ||| ||||||||||||||||||||||||||||||||||||||||||||||||||||||||
Sbjct  1638  GGGTTCGAACCTTCCGCCCCTCCGGGGGCGTGGAGCACTCTTGGAACGCGCACCGCCTGA  1697

Query  240   GACTCCCCCGCAAGGGGGAACACGAAAAAACGAGAACGACTCTCGGCAACGGATA  294
             |||||||||||||||||||||||||||||||||||||||||||||||||||||||
Sbjct  1698  GACTCCCCCGCAAGGGGGAACACGAAAAAACGAGAACGACTCTCGGCAACGGATA  1752
```

Equisetum variegatum 18S ribosomal RNA gene, partial sequence; internal transcribed spacer 1, complete sequence; and 5.8S ribosomal RNA gene, partial sequence
Sequence ID: **EU328340.1** Length: 305 Number of Matches: 1
Range 1: 1 to 275

Score	Expect	Identities	Gaps	Strand	Frame
488 bits(264)	2e-133()	271/275(99%)	0/275(0%)	Plus/Plus	

Features:

```
Query  20   GGTGAACCTGCGGAAGGATCATTGTCACACATCCGTGCAAACCCTGCGAACCGTGGACGT  79
            ||||||||||||||||||||||||||||||||||||||||||||||||||||||||||||
Sbjct  1    GGTGAACCTGCGGAAGGATCATTGTCACACATCCGTGCAAACCCTGCGAACCGTGGACGT  60
```

```
Query  80    CCCCCAAGGGGGCGGTGGTTGTGGTGCCCGCTTGCGCCCTCGCGGGCGTGGGTGCGGTGC  139
             ||||||||||||||||||||||||||||||||||||||||||||||||||||||||||||
Sbjct  61    CCCCCAAGGGGGCGGTGGTTGTGGTGCCCGCTTGCGCCCTCGCGGGCGTGGGTGCGGTGC  120

Query  140   GGCGCCCCGTCCCCTCCTCTCGGTCGCTCCGGCGGCCgggggggTCGAACCTTCCGCCCC  199
             |||||||||||||||||||||||||||| ||||||||||||| |||||||||||||||||
Sbjct  121   GGCGCCCCGTCCCCTCCTCTCGGTCGCTTCGGCGGCCGGGGGNGTCGAACCTTCCGCCCC  180

Query  200   TCCGGGGGCGTGGAGCACTCTTGGAACGCGCACCGCCTGAGACTCCCCCGCAAGGGGGAA  259
             ||||||||||||||||||||||| ||| ||||||||||||||||||||||||||||||||
Sbjct  181   TCCGGGGGCGTGGAGCACTCTTGAAACACGCACCGCCTGAGACTCCCCCGCAAGGGGGAA  240

Query  260   CACGAAAAAACGAGAACGACTCTCGGCAACGGATA  294
             |||||||||||||||||||||||||||||||||||
Sbjct  241   CACGAAAAAACGAGAACGACTCTCGGCAACGGATA  275
```

Equisetum variegatum voucher B. Hoshizaki 592 18S ribosomal RNA gene, partial sequence; internal transcribed spacer 1, complete sequence; and 5.8S ribosomal RNA gene, partial sequence

Sequence ID: **EU372664.1** Length: 305 Number of Matches: 1

Range 1: 1 to 275

Score	Expect	Identities	Gaps	Strand	Frame
484 bits(262)	2e-132()	270/275(98%)	0/275(0%)	Plus/Plus	

Features:

```
Query  20    GGTGAACCTGCGGAAGGATCATTGTCACACATCCGTGCAAACCCTGCGAACCGTGGACGT  79
             ||||||||||||||||||||||||||||||||||||||||||||||||||||||||||||
Sbjct  1     GGTGAACCTGCGGAAGGATCATTGTCACACATCCGTGCAAACCCTGCGAACCGTGGACGT  60

Query  80    CCCCCAAGGGGGCGGTGGTTGTGGTGCCCGCTTGCGCCCTCGCGGGCGTGGGTGCGGTGC  139
             ||||||||||||||||||||||||||||||||||||||||||||||||||||||||||||
Sbjct  61    CCCCCAAGGGGGCGGTGGTTGTGGTGCCCGCTTGCGCCCTCGCGGGCGTGGGTGCGGTGC  120

Query  140   GGCGCCCCGTCCCCTCCTCTCGGTCGCTCCGGCGGCCgggggggTCGAACCTTCCGCCCC  199
             |||||||||||||||||||||||||||| ||||||||||||| |||||||||||||||||
Sbjct  121   GGCGCCCCGTCCCCTCCTCTCGGTCGCTTCGGCGGCCGGGGGNGTCGAACCTTCCGCCCC  180

Query  200   TCCGGGGGCGTGGAGCACTCTTGGAACGCGCACCGCCTGAGACTCCCCCGCAAGGGGGAA  259
             ||||||||||||||||||||||| ||| |||||||||||| |||||||||||||||||||
Sbjct  181   TCCGGGGGCGTGGAGCACTCTTGAAACACGCACCGCCTGANACTCCCCCGCAAGGGGGAA  240

Query  260   CACGAAAAAACGAGAACGACTCTCGGCAACGGATA  294
             |||||||||||||||||||||||||||||||||||
Sbjct  241   CACGAAAAAACGAGAACGACTCTCGGCAACGGATA  275
```


BLAST is a registered trademark of the National Library of Medicine

Support center Mailing list YouTube

- National Library Of Medicine
- National Institutes Of Health
- U.S. Department of Health & Human Services
- USA.gov

NCBI

National Center for Biotechnology Information, U.S. National Library of Medicine 8600 Rockville Pike, Bethesda MD, 20894 USA

Policies and Guidelines | Contact

HHS Vulnerability Disclosure
