# Supplementary material for: A Multi-Pronged Technique for Identifying Equisetum palustre and Equisetum arvense—Combining HPTLC, HPLC-ESI-MS/MS and Optimized DNA Barcoding Techniques
Source: Plants (Basel). 2022 Sep 28;11(19):2562. doi: 10.3390/plants11192562 (PMC9573248; doi:10.3390/plants11192562)
Supplement: Supplementary file 1 [file plants-11-02562-s001.zip › plants-1932149 supplementary/Horsetail Supplementary Figures/Figure S11.pdf]

BLAST Results

Questions/comments

Job title: DQ377156.1

**RID**	FD48SC2U013 (Expires on 08-14 01:42 am)		
**Query ID**	lcl|Query_2959	**Database Name**	nt
**Description**	None	**Description**	Nucleotide collection (nt)
**Molecule type**	dna	**Program**	BLASTN 2.13.0+
**Query Length**	211		

Graphic Summary

Distribution of the top 20 Blast Hits on 20 subject sequences

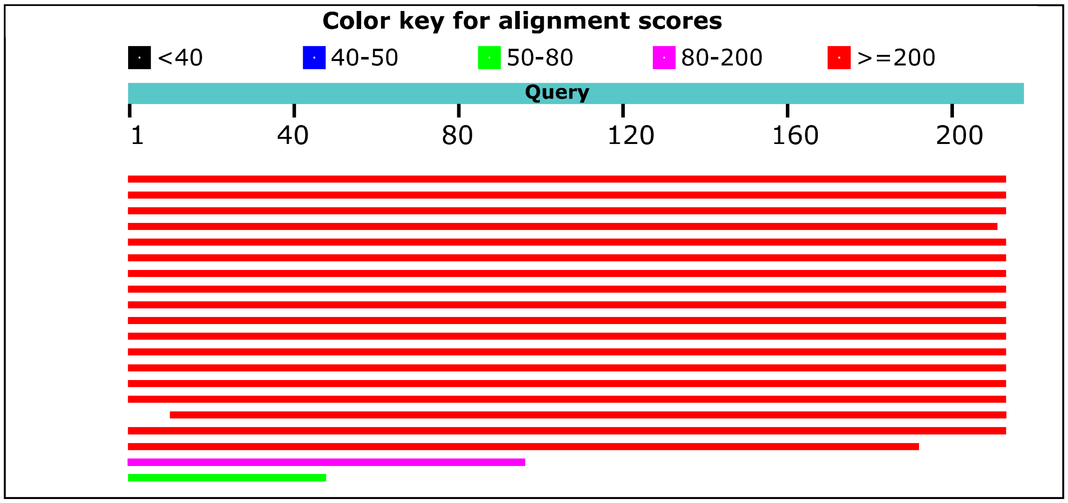

Descriptions

Sequences producing significant alignments:

Description	Max Score	Total Score	Query Cover	E value	Per. Ident	Accession
Equisetum x meridionale internal transcribed spacer 1, partial sequence	390	390	100%	3e-104	100.00%	DQ377156.1
Equisetum ramosissimum isolate BPTPS056 small subunit ribosomal RNA gene, partial sequence; internal transcribed spacer 1, 5.8S ribosomal RNA gene, and internal transcribed spacer 2, complete sequence; and large subunit ribosomal RNA gene, partial sequence	372	372	100%	1e-98	98.58%	OM670239.1
Equisetum variegatum subsp. variegatum internal transcribed spacer 1, partial sequence	350	350	100%	6e-92	96.68%	DQ377155.1
Equisetum ramosissimum subsp. ramosissimum internal transcribed spacer 1, partial sequence	344	344	99%	3e-90	96.65%	DQ377153.1
Equisetum x moorei internal transcribed spacer 1, partial sequence	333	333	100%	6e-87	95.28%	DQ377157.1
Equisetum variegatum 18S ribosomal RNA gene, partial sequence; internal transcribed spacer 1, complete sequence; and 5.8S ribosomal RNA gene, partial sequence	329	329	100%	8e-86	94.79%	EU328340.1
Equisetum variegatum voucher B. Hoshizaki 592 18S ribosomal RNA gene, partial sequence; internal transcribed spacer 1, complete sequence; and 5.8S ribosomal RNA gene, partial sequence	326	326	100%	1e-84	94.31%	EU372664.1
Equisetum laevigatum 18S ribosomal RNA gene, partial sequence; internal transcribed spacer 1, complete sequence; and 5.8S ribosomal RNA gene, partial sequence	322	322	100%	1e-83	93.84%	EU328341.1
Equisetum ramosissimum isolate BPTPS123 small subunit ribosomal RNA gene, partial sequence; internal transcribed spacer 1, 5.8S ribosomal RNA gene, and internal transcribed spacer 2, complete sequence; and large subunit ribosomal RNA gene, partial sequence	316	316	100%	6e-82	93.84%	ON685433.1
Equisetum ramosissimum 18S ribosomal RNA gene, partial sequence; internal transcribed spacer 1, 5.8S ribosomal RNA gene and internal transcribed spacer 2, complete sequence; and 26S ribosomal RNA gene, partial sequence	316	316	100%	6e-82	93.84%	AF448794.1
Equisetum x ferrissii 18S ribosomal RNA gene, partial sequence; internal transcribed spacer 1, complete sequence; and 5.8S ribosomal RNA gene, partial sequence	311	311	100%	3e-80	92.89%	EU328338.1
Equisetum hyemale subsp. affine 18S ribosomal RNA gene, partial sequence; internal transcribed spacer 1, complete sequence; and 5.8S ribosomal RNA gene, partial sequence	303	303	100%	5e-78	92.42%	EU328339.1

Description	Max Score	Total Score	Query Cover	E value	Per. Ident	Accession
Equisetum variegatum voucher B. Hoshizaki 203 18S ribosomal RNA gene, partial sequence; internal transcribed spacer 1, complete sequence; and 5.8S ribosomal RNA gene, partial sequence	302	302	100%	2e-77	92.42%	EU372665.1
Equisetum hyemale subsp. hyemale internal transcribed spacer 1, partial sequence	300	300	100%	6e-77	92.42%	DQ377151.1
Equisetum hyemale subsp. affine 18S ribosomal RNA gene, partial sequence; internal transcribed spacer 1, complete sequence; and 5.8S ribosomal RNA gene, partial sequence	298	298	100%	2e-76	91.94%	EU328336.1
Equisetum hyemale genes for ITS1, 5.8S rRNA, ITS2, partial sequence, bio_material: KB:NIBR0000051239	281	281	95%	2e-71	92.04%	AB817880.1
Equisetum scirpoides voucher P. Hammond s.n 18S ribosomal RNA gene, partial sequence; internal transcribed spacer 1, complete sequence; and 5.8S ribosomal RNA gene, partial sequence	274	274	100%	4e-69	89.10%	EU372663.1
Equisetum ramosissimum 18S ribosomal RNA gene, partial sequence; internal transcribed spacer 1, complete sequence; and 5.8S ribosomal RNA gene, partial sequence	239	239	90%	1e-58	88.95%	EU328343.1
Equisetum telmateia isolate DM233 small subunit ribosomal RNA gene, internal transcribed spacer 1, 5.8S ribosomal RNA gene, internal transcribed spacer 2, and large subunit ribosomal RNA gene, region	102	102	45%	2e-17	86.32%	MT784095.1
Equisetum sylvaticum 18S ribosomal RNA gene, partial sequence; internal transcribed spacer 1, complete sequence; and 5.8S ribosomal RNA gene, partial sequence	71.3	71.3	22%	5e-08	93.62%	EU328342.1

Alignments

Equisetum x meridionale internal transcribed spacer 1, partial sequence

Sequence ID: **DQ377156.1** Length: 211 Number of Matches: 1

Range 1: 1 to 211

Score	Expect	Identities	Gaps	Strand	Frame
390 bits(211)	3e-104()	211/211(100%)	0/211(0%)	Plus/Plus	

Features:

```
Query  1    TCATTGTCACACATCCGTGCAAACCCTGTGAACCGTGGATGTCCCCCAAAGGGGGCGGTG  60
            ||||||||||||||||||||||||||||||||||||||||||||||||||||||||||||
Sbjct  1    TCATTGTCACACATCCGTGCAAACCCTGTGAACCGTGGATGTCCCCCAAAGGGGGCGGTG  60

Query  61   GTTGTGGTGCCCGCTTGTGCCCTCGCGGGCGTGGGTGTGGTGCGGCGCCCCCGTCCCCTC  120
            ||||||||||||||||||||||||||||||||||||||||||||||||||||||||||||
Sbjct  61   GTTGTGGTGCCCGCTTGTGCCCTCGCGGGCGTGGGTGTGGTGCGGCGCCCCCGTCCCCTC  120

Query  121  CTTTCGGTCGCTCCGGCGGCCgggggggTCGAACCTTCCGCCCCTCCGGGGGCGTGGAGC  180
            ||||||||||||||||||||||||||||||||||||||||||||||||||||||||||||
Sbjct  121  CTTTCGGTCGCTCCGGCGGCCGGGGGGGTCGAACCTTCCGCCCCTCCGGGGGCGTGGAGC  180

Query  181  ACTCTTGGAACGCGCACCGCCTGAGACTCCC  211
            |||||||||||||||||||||||||||||||
Sbjct  181  ACTCTTGGAACGCGCACCGCCTGAGACTCCC  211
```

Equisetum ramosissimum isolate BPTPS056 small subunit ribosomal RNA gene, partial sequence; internal transcribed spacer 1, 5.8S ribosomal RNA gene, and internal transcribed spacer 2, complete sequence; and large subunit ribosomal RNA gene, partial sequence
Sequence ID: **OM670239.1** Length: 3631 Number of Matches: 1
Range 1: 1495 to 1704

Score	Expect	Identities	Gaps	Strand	Frame
372 bits(201)	1e-98()	208/211(99%)	1/211(0%)	Plus/Plus	

Features:

```
Query  1     TCATTGTCACACATCCGTGCAAACCCTGTGAACCGTGGATGTCCCCCAAAGGGGGCGGTG  60
             |||||||||||||||||||||||||||||||||||||||||||||||||| |||||||||
Sbjct  1495  TCATTGTCACACATCCGTGCAAACCCTGTGAACCGTGGATGTCCCCCAAA-GGGGCGGTG  1553

Query  61    GTTGTGGTGCCCGCTTGTGCCCTCGCGGGCGTGGGTGTGGTGCGGCGCCCCCGTCCCCTC  120
             |||||||||||||||||||||||||||||||||||||||||||||||||||| |||||||
Sbjct  1554  GTTGTGGTGCCCGCTTGTGCCCTCGCGGGCGTGGGTGTGGTGCGGCGCCCCCTTCCCCTC  1613

Query  121   CTTTCGGTCGCTCCGGCGGCCgggggggTCGAACCTTCCGCCCCTCCGGGGGCGTGGAGC  180
             ||||||||||||||||||||||||||| ||||||||||||||||||||||||||||||||
Sbjct  1614  CTTTCGGTCGCTCCGGCGGCCGGGGGGTTCGAACCTTCCGCCCCTCCGGGGGCGTGGAGC  1673

Query  181   ACTCTTGGAACGCGCACCGCCTGAGACTCCC  211
             |||||||||||||||||||||||||||||||
Sbjct  1674  ACTCTTGGAACGCGCACCGCCTGAGACTCCC  1704
```

Equisetum variegatum subsp. variegatum internal transcribed spacer 1, partial sequence
Sequence ID: **DQ377155.1** Length: 294 Number of Matches: 1
Range 1: 38 to 246

Score	Expect	Identities	Gaps	Strand	Frame
350 bits(189)	6e-92()	204/211(97%)	2/211(0%)	Plus/Plus	

Features:

```
Query  1    TCATTGTCACACATCCGTGCAAACCCTGTGAACCGTGGATGTCCCCCAAAGGGGGCGGTG  60
            |||||||||||||||||||||||||||| |||||||||| ||||||| ||||||||||||
Sbjct  38   TCATTGTCACACATCCGTGCAAACCCTGCGAACCGTGGACGTCCCCC-AAGGGGGCGGTG  96

Query  61   GTTGTGGTGCCCGCTTGTGCCCTCGCGGGCGTGGGTGTGGTGCGGCGCCCCCGTCCCCTC  120
            ||||||||||||||||| ||||||||||||||||||| ||||||||| ||||||||||||
Sbjct  97   GTTGTGGTGCCCGCTTGCGCCCTCGCGGGCGTGGGTGCGGTGCGGCG-CCCCGTCCCCTC  155

Query  121  CTTTCGGTCGCTCCGGCGGCCgggggggTCGAACCTTCCGCCCCTCCGGGGGCGTGGAGC  180
            || |||||||||||||||||||||||||||||||||||||||||||||||||||||||||
Sbjct  156  CTCTCGGTCGCTCCGGCGGCCGGGGGGGTCGAACCTTCCGCCCCTCCGGGGGCGTGGAGC  215

Query  181  ACTCTTGGAACGCGCACCGCCTGAGACTCCC  211
            |||||||||||||||||||||||||||||||
Sbjct  216  ACTCTTGGAACGCGCACCGCCTGAGACTCCC  246
```

Equisetum ramosissimum subsp. ramosissimum internal transcribed spacer 1, partial sequence
Sequence ID: **DQ377153.1** Length: 206 Number of Matches: 1
Range 1: 1 to 206

Score	Expect	Identities	Gaps	Strand	Frame
344 bits(186)	3e-90()	202/209(97%)	3/209(1%)	Plus/Plus	

Features:

```
Query  1    TCATTGTCACACATCCGTGCAAACCCTGTGAACCGTGGATGTCCCCCAAAGGGGGCGGTG  60
            |||||||||||||||||||||||||||||| |||||||||||||| |||| |||||||||
Sbjct  1    TCATTGTCACACATCCGTGCAAACCCTGTG-ACCGTGGATGTCCCTCAAA-GGGGCGGTG  58

Query  61   GTTGTGGTGCCCGCTTGTGCCCTCGCGGGCGTGGGTGTGGTGCGGCGCCCCCGTCCCCTC  120
            ||||||||||||||||||||||||||||||||| ||| ||||||||| ||||||||||||
Sbjct  59   GTTGTGGTGCCCGCTTGTGCCCTCGCGGGCGTGAGTGCGGTGCGGCG-CCCCGTCCCCTC  117

Query  121  CTTTCGGTCGCTCCGGCGGCCgggggggTCGAACCTTCCGCCCCTCCGGGGGCGTGGAGC  180
            ||||||||||||||||||||||||||| ||||||||||||||||||||||||||||||||
Sbjct  118  CTTTCGGTCGCTCCGGCGGCCGGGGGGTTCGAACCTTCCGCCCCTCCGGGGGCGTGGAGC  177
```

```
Query  181   ACTCTTGGAACGCGCACCGCCTGAGACTC  209
             |||||||||||||||||||||||||||||
Sbjct  178   ACTCTTGGAACGCGCACCGCCTGAGACTC  206
```

Equisetum x moorei internal transcribed spacer 1, partial sequence

Sequence ID: **DQ377157.1** Length: 209 Number of Matches: 1

Range 1: 1 to 209

Score	Expect	Identities	Gaps	Strand	Frame
333 bits(180)	6e-87()	202/212(95%)	4/212(1%)	Plus/Plus	

Features:

```
Query  1     TCATTGTCACACATCCGTGCAAACCCTG-TGAACCGTGGATGTCCCCCAAAGGGGGCGGT  59
             |||||||||||||||||||||||||||| || |||||||||||||| | ||| |||||||
Sbjct  1     TCATTGTCACACATCCGTGCAAACCCTGCTG-ACCGTGGATGTCCCTC-AAGAGGGCGGT  58

Query  60    GGTTGTGGTGCCCGCTTGTGCCCTCGCGGGCGTGGGTGTGGTGCGGCGCCCCCGTCCCCT  119
             |||||||||||||||||||||||||||||||||| ||| ||||||||| |||||||||||
Sbjct  59    GGTTGTGGTGCCCGCTTGTGCCCTCGCGGGCGTGAGTGCGGTGCGGCG-CCCCGTCCCCT  117

Query  120   CCTTTCGGTCGCTCCGGCGGCCgggggggTCGAACCTTCCGCCCCTCCGGGGGCGTGGAG  179
             ||| |||||||||||||||||||||||| |||||||||||||||||||||||||||||||
Sbjct  118   CCTCTCGGTCGCTCCGGCGGCCGGGGGGTTCGAACCTTCCGCCCCTCCGGGGGCGTGGAG  177

Query  180   CACTCTTGGAACGCGCACCGCCTGAGACTCCC  211
             ||||||||||||||||||||||||||||||||
Sbjct  178   CACTCTTGGAACGCGCACCGCCTGAGACTCCC  209
```

BLAST is a registered trademark of the National Library of Medicine

Support center Mailing list YouTube

- National Library Of Medicine
- National Institutes Of Health
- U.S. Department of Health & Human Services
- USA.gov

NCBI

National Center for Biotechnology Information, U.S. National Library of Medicine 8600 Rockville Pike, Bethesda MD, 20894 USA

Policies and Guidelines | Contact

HHS Vulnerability Disclosure
