# Supplementary material for: A Multi-Pronged Technique for Identifying Equisetum palustre and Equisetum arvense—Combining HPTLC, HPLC-ESI-MS/MS and Optimized DNA Barcoding Techniques
Source: Plants (Basel). 2022 Sep 28;11(19):2562. doi: 10.3390/plants11192562 (PMC9573248; doi:10.3390/plants11192562)
Supplement: Supplementary file 1 [file plants-11-02562-s001.zip › plants-1932149 supplementary/Horsetail Supplementary Figures/Figure S12.pdf]

BLAST Results

Questions/comments

Job title: DQ377157.1

**RID**	FD3U2VX801R (Expires on 08-14 01:34 am)
**Query ID**	lcl|Query_521929
**Description**	None
**Molecule type**	dna
**Query Length**	209
**Database Name**	nt
**Description**	Nucleotide collection (nt)
**Program**	BLASTN 2.13.0+

Graphic Summary

Distribution of the top 20 Blast Hits on 20 subject sequences

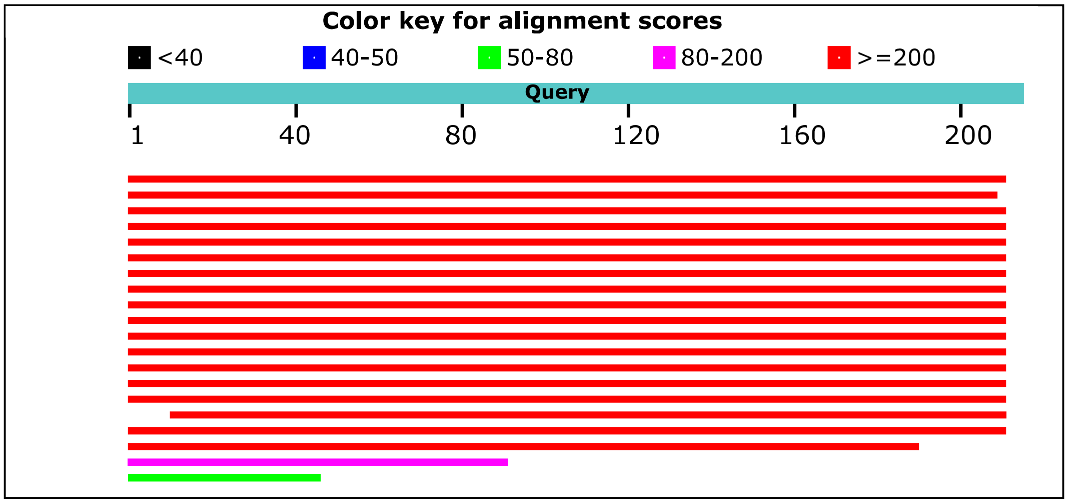

Descriptions

Sequences producing significant alignments:

Description	Max Score	Total Score	Query Cover	E value	Per. Ident	Accession
Equisetum x moorei internal transcribed spacer 1, partial sequence	387	387	100%	4e-103	100.00%	DQ377157.1
Equisetum ramosissimum subsp. ramosissimum internal transcribed spacer 1, partial sequence	359	359	99%	1e-94	98.07%	DQ377153.1
Equisetum variegatum subsp. variegatum internal transcribed spacer 1, partial sequence	342	342	100%	1e-89	96.17%	DQ377155.1
Equisetum x meridionale internal transcribed spacer 1, partial sequence	333	333	100%	6e-87	95.28%	DQ377156.1
Equisetum ramosissimum isolate BPTPS123 small subunit ribosomal RNA gene, partial sequence; internal transcribed spacer 1, 5.8S ribosomal RNA gene, and internal transcribed spacer 2, complete sequence; and large subunit ribosomal RNA gene, partial sequence	331	331	100%	2e-86	95.22%	ON685433.1
Equisetum ramosissimum isolate BPTPS056 small subunit ribosomal RNA gene, partial sequence; internal transcribed spacer 1, 5.8S ribosomal RNA gene, and internal transcribed spacer 2, complete sequence; and large subunit ribosomal RNA gene, partial sequence	331	331	100%	2e-86	95.26%	OM670239.1
Equisetum variegatum 18S ribosomal RNA gene, partial sequence; internal transcribed spacer 1, complete sequence; and 5.8S ribosomal RNA gene, partial sequence	322	322	100%	1e-83	94.26%	EU328340.1
Equisetum variegatum voucher B. Hoshizaki 592 18S ribosomal RNA gene, partial sequence; internal transcribed spacer 1, complete sequence; and 5.8S ribosomal RNA gene, partial sequence	318	318	100%	2e-82	93.78%	EU372664.1
Equisetum laevigatum 18S ribosomal RNA gene, partial sequence; internal transcribed spacer 1, complete sequence; and 5.8S ribosomal RNA gene, partial sequence	315	315	100%	2e-81	93.30%	EU328341.1
Equisetum ramosissimum 18S ribosomal RNA gene, partial sequence; internal transcribed spacer 1, 5.8S ribosomal RNA gene and internal transcribed spacer 2, complete sequence; and 26S ribosomal RNA gene, partial sequence	309	309	100%	1e-79	93.30%	AF448794.1
Equisetum x ferrissii 18S ribosomal RNA gene, partial sequence; internal transcribed spacer 1, complete sequence; and 5.8S ribosomal RNA gene, partial sequence	303	303	100%	5e-78	92.34%	EU328338.1
Equisetum hyemale subsp. affine 18S ribosomal RNA gene, partial sequence; internal transcribed spacer 1, complete sequence; and 5.8S ribosomal RNA gene, partial sequence	296	296	100%	8e-76	91.87%	EU328339.1

Description	Max Score	Total Score	Query Cover	E value	Per. Ident	Accession
Equisetum variegatum voucher B. Hoshizaki 203 18S ribosomal RNA gene, partial sequence; internal transcribed spacer 1, complete sequence; and 5.8S ribosomal RNA gene, partial sequence	294	294	100%	3e-75	91.87%	EU372665.1
Equisetum hyemale subsp. hyemale internal transcribed spacer 1, partial sequence	292	292	100%	1e-74	91.87%	DQ377151.1
Equisetum hyemale subsp. affine 18S ribosomal RNA gene, partial sequence; internal transcribed spacer 1, complete sequence; and 5.8S ribosomal RNA gene, partial sequence	291	291	100%	4e-74	91.39%	EU328336.1
Equisetum hyemale genes for ITS1, 5.8S rRNA, ITS2, partial sequence, bio_material: KB:NIBR0000051239	274	274	95%	4e-69	91.46%	AB817880.1
Equisetum scirpoides voucher P. Hammond s.n 18S ribosomal RNA gene, partial sequence; internal transcribed spacer 1, complete sequence; and 5.8S ribosomal RNA gene, partial sequence	267	267	100%	6e-67	88.52%	EU372663.1
Equisetum ramosissimum 18S ribosomal RNA gene, partial sequence; internal transcribed spacer 1, complete sequence; and 5.8S ribosomal RNA gene, partial sequence	226	226	89%	1e-54	87.77%	EU328343.1
Equisetum telmateia isolate DM233 small subunit ribosomal RNA gene, internal transcribed spacer 1, 5.8S ribosomal RNA gene, internal transcribed spacer 2, and large subunit ribosomal RNA gene, region	89.8	89.8	43%	1e-13	84.44%	MT784095.1
Equisetum sylvaticum 18S ribosomal RNA gene, partial sequence; internal transcribed spacer 1, complete sequence; and 5.8S ribosomal RNA gene, partial sequence	62.1	62.1	21%	3e-05	91.11%	EU328342.1

Alignments

Equisetum x moorei internal transcribed spacer 1, partial sequence

Sequence ID: **DQ377157.1** Length: 209 Number of Matches: 1

Range 1: 1 to 209

Score	Expect	Identities	Gaps	Strand	Frame
387 bits(209)	4e-103()	209/209(100%)	0/209(0%)	Plus/Plus	

Features:

```
Query  1    TCATTGTCACACATCCGTGCAAACCCTGCTGACCGTGGATGTCCCTCAAGAGGGCGGTGG  60
            ||||||||||||||||||||||||||||||||||||||||||||||||||||||||||||
Sbjct  1    TCATTGTCACACATCCGTGCAAACCCTGCTGACCGTGGATGTCCCTCAAGAGGGCGGTGG  60

Query  61   TTGTGGTGCCCGCTTGTGCCCTCGCGGGCGTGAGTGCGGTGCGGCGCCCCGTCCCCTCCT  120
            ||||||||||||||||||||||||||||||||||||||||||||||||||||||||||||
Sbjct  61   TTGTGGTGCCCGCTTGTGCCCTCGCGGGCGTGAGTGCGGTGCGGCGCCCCGTCCCCTCCT  120

Query  121  CTCGGTCGCTCCGGCGGCCGGGGGGTTCGAACCTTCCGCCCCTCCGGGGGCGTGGAGCAC  180
            ||||||||||||||||||||||||||||||||||||||||||||||||||||||||||||
Sbjct  121  CTCGGTCGCTCCGGCGGCCGGGGGGTTCGAACCTTCCGCCCCTCCGGGGGCGTGGAGCAC  180

Query  181  TCTTGGAACGCGCACCGCCTGAGACTCCC  209
            |||||||||||||||||||||||||||||
Sbjct  181  TCTTGGAACGCGCACCGCCTGAGACTCCC  209
```

Equisetum ramosissimum subsp. ramosissimum internal transcribed spacer 1, partial sequence

Sequence ID: **DQ377153.1** Length: 206 Number of Matches: 1

Range 1: 1 to 206

**Score**	**Expect**	**Identities**	**Gaps**	**Strand**	**Frame**
359 bits(194)	1e-94()	203/207(98%)	1/207(0%)	Plus/Plus	

Features:

```
Query  1    TCATTGTCACACATCCGTGCAAACCCTGCTGACCGTGGATGTCCCTCAAGAGGGCGGTGG  60
            |||||||||||||||||||||||||||| ||||||||||||||||||||  |||||||||
Sbjct  1    TCATTGTCACACATCCGTGCAAACCCTG-TGACCGTGGATGTCCCTCAAAGGGGCGGTGG  59

Query  61   TTGTGGTGCCCGCTTGTGCCCTCGCGGGCGTGAGTGCGGTGCGGCGCCCCGTCCCCTCCT  120
            ||||||||||||||||||||||||||||||||||||||||||||||||||||||||||||
Sbjct  60   TTGTGGTGCCCGCTTGTGCCCTCGCGGGCGTGAGTGCGGTGCGGCGCCCCGTCCCCTCCT  119

Query  121  CTCGGTCGCTCCGGCGGCCGGGGGGTTCGAACCTTCCGCCCCTCCGGGGGCGTGGAGCAC  180
             |||||||||||||||||||||||||||||||||||||||||||||||||||||||||||
Sbjct  120  TTCGGTCGCTCCGGCGGCCGGGGGGTTCGAACCTTCCGCCCCTCCGGGGGCGTGGAGCAC  179

Query  181  TCTTGGAACGCGCACCGCCTGAGACTC  207
            |||||||||||||||||||||||||||
Sbjct  180  TCTTGGAACGCGCACCGCCTGAGACTC  206
```

Equisetum variegatum subsp. variegatum internal transcribed spacer 1, partial sequence

Sequence ID: **DQ377155.1** Length: 294 Number of Matches: 1

Range 1: 38 to 246

**Score**	**Expect**	**Identities**	**Gaps**	**Strand**	**Frame**
342 bits(185)	1e-89()	201/209(96%)	0/209(0%)	Plus/Plus	

Features:

```
Query  1    TCATTGTCACACATCCGTGCAAACCCTGCTGACCGTGGATGTCCCTCAAGAGGGCGGTGG  60
            |||||||||||||||||||||||||||||  |||||||| ||||| |||| |||||||||
Sbjct  38   TCATTGTCACACATCCGTGCAAACCCTGCGAACCGTGGACGTCCCCCAAGGGGGCGGTGG  97

Query  61   TTGTGGTGCCCGCTTGTGCCCTCGCGGGCGTGAGTGCGGTGCGGCGCCCCGTCCCCTCCT  120
            |||||||||||||||| ||||||||||||||| |||||||||||||||||||||||||||
Sbjct  98   TTGTGGTGCCCGCTTGCGCCCTCGCGGGCGTGGGTGCGGTGCGGCGCCCCGTCCCCTCCT  157

Query  121  CTCGGTCGCTCCGGCGGCCGGGGGGTTCGAACCTTCCGCCCCTCCGGGGGCGTGGAGCAC  180
            ||||||||||||||||||||||||| ||||||||||||||||||||||||||||||||||
Sbjct  158  CTCGGTCGCTCCGGCGGCCGGGGGGGTCGAACCTTCCGCCCCTCCGGGGGCGTGGAGCAC  217

Query  181  TCTTGGAACGCGCACCGCCTGAGACTCCC  209
            |||||||||||||||||||||||||||||
Sbjct  218  TCTTGGAACGCGCACCGCCTGAGACTCCC  246
```

Equisetum x meridionale internal transcribed spacer 1, partial sequence

Sequence ID: **DQ377156.1** Length: 211 Number of Matches: 1

Range 1: 1 to 211

**Score**	**Expect**	**Identities**	**Gaps**	**Strand**	**Frame**
333 bits(180)	6e-87()	202/212(95%)	4/212(1%)	Plus/Plus	

Features:

```
Query  1    TCATTGTCACACATCCGTGCAAACCCTGCTG-ACCGTGGATGTCCCTC-AAGAGGGCGGT  58
            |||||||||||||||||||||||||||| || |||||||||||||| | ||| |||||||
Sbjct  1    TCATTGTCACACATCCGTGCAAACCCTG-TGAACCGTGGATGTCCCCCAAAGGGGGCGGT  59

Query  59   GGTTGTGGTGCCCGCTTGTGCCCTCGCGGGCGTGAGTGCGGTGCGGCG-CCCCGTCCCCT  117
            |||||||||||||||||||||||||||||||||| ||| ||||||||| |||||||||||
Sbjct  60   GGTTGTGGTGCCCGCTTGTGCCCTCGCGGGCGTGGGTGTGGTGCGGCGCCCCCGTCCCCT  119

Query  118  CCTCTCGGTCGCTCCGGCGGCCGGGGGGTTCGAACCTTCCGCCCCTCCGGGGGCGTGGAG  177
            ||| |||||||||||||||||||||||| |||||||||||||||||||||||||||||||
Sbjct  120  CCTTTCGGTCGCTCCGGCGGCCGGGGGGGTCGAACCTTCCGCCCCTCCGGGGGCGTGGAG  179

Query  178  CACTCTTGGAACGCGCACCGCCTGAGACTCCC  209
            ||||||||||||||||||||||||||||||||
```

```
Sbjct  180  CACTCTTGGAACGCGCACCGCCTGAGACTCCC  211
```

Equisetum ramosissimum isolate BPTPS123 small subunit ribosomal RNA gene, partial sequence; internal transcribed spacer 1, 5.8S ribosomal RNA gene, and internal transcribed spacer 2, complete sequence; and large subunit ribosomal RNA gene, partial sequence
Sequence ID: **ON685433.1** Length: 3702 Number of Matches: 1
Range 1: 1539 to 1747

Score	Expect	Identities	Gaps	Strand	Frame
331 bits(179)	2e-86()	199/209(95%)	0/209(0%)	Plus/Plus	

Features:

```
Query  1     TCATTGTCACACATCCGTGCAAACCCTGCTGACCGTGGATGTCCCTCAAGAGGGCGGTGG  60
             ||||||||||||||||||||||||| |||  |||||||| ||||||||||||||||||||
Sbjct  1539  TCATTGTCACACATCCGTGCAAACCATGCGAACCGTGGACGTCCCTCAAGAGGGCGGTGG  1598

Query  61    TTGTGGTGCCCGCTTGTGCCCTCGCGGGCGTGAGTGCGGTGCGGCGCCCCGTCCCCTCCT  120
             |||||||||||||||| ||||| ||||||||| |||||||||||||||||||||||||||
Sbjct  1599  TTGTGGTGCCCGCTTGCGCCCTTGCGGGCGTGGGTGCGGTGCGGCGCCCCGTCCCCTCCT  1658

Query  121   CTCGGTCGCTCCGGCGGCCGGGGGGTTCGAACCTTCCGCCCCTCCGGGGGCGTGGAGCAC  180
             ||||| ||||||||||||||||||| |||||||||||||||| |||||||||||||||||
Sbjct  1659  CTCGGCCGCTCCGGCGGCCGGGGGGGTCGAACCTTCCGCCCCCCCGGGGGCGTGGAGCAC  1718

Query  181   TCTTGGAACGCGCACCGCCTGAGACTCCC  209
             |||||||||||||||||||||||||||||
Sbjct  1719  TCTTGGAACGCGCACCGCCTGAGACTCCC  1747
```

BLAST is a registered trademark of the National Library of Medicine

Support center Mailing list YouTube

- National Library Of Medicine
- National Institutes Of Health
- U.S. Department of Health & Human Services
- USA.gov

NCBI
National Center for Biotechnology Information, U.S. National Library of Medicine 8600 Rockville Pike, Bethesda MD, 20894 USA
Policies and Guidelines | Contact
HHS Vulnerability Disclosure
