# Supplementary material for: A Multi-Pronged Technique for Identifying Equisetum palustre and Equisetum arvense—Combining HPTLC, HPLC-ESI-MS/MS and Optimized DNA Barcoding Techniques
Source: Plants (Basel). 2022 Sep 28;11(19):2562. doi: 10.3390/plants11192562 (PMC9573248; doi:10.3390/plants11192562)
Supplement: Supplementary file 1 [file plants-11-02562-s001.zip › plants-1932149 supplementary/Horsetail Supplementary Figures/Figure S13.pdf]

BLAST Results

Questions/comments

Job title: AF448794.1

**RID**	FD4KVT9701R (Expires on 08-14 01:48 am)
**Query ID**	lcl|Query_232813
**Description**	None
**Molecule type**	dna
**Query Length**	652
**Database Name**	nt
**Description**	Nucleotide collection (nt)
**Program**	BLASTN 2.13.0+

Graphic Summary

Distribution of the top 100 Blast Hits on 100 subject sequences

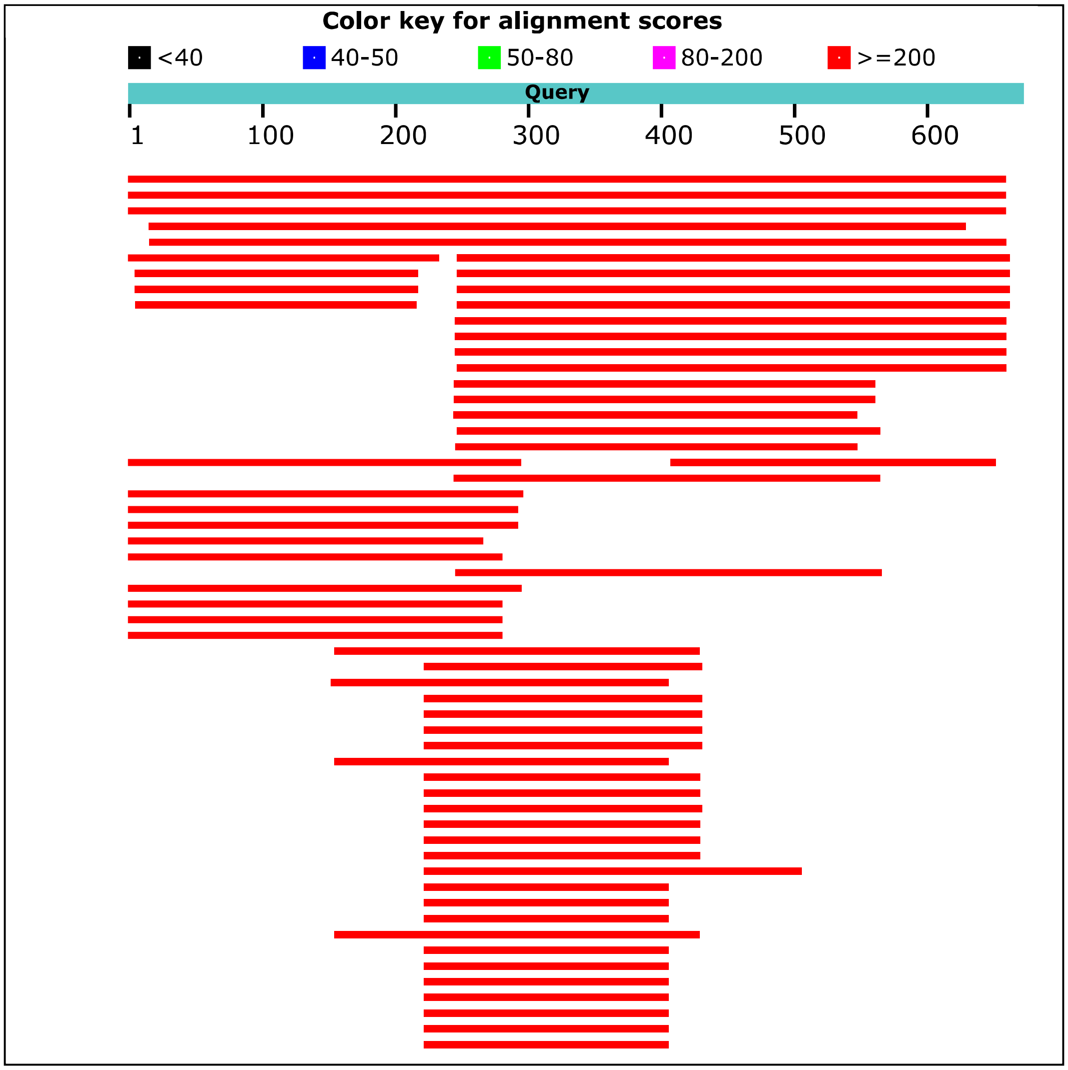

Descriptions

Sequences producing significant alignments:

Description	Max Score	Total Score	Query Cover	E value	Per. Ident	Accession
Equisetum ramosissimum 18S ribosomal RNA gene, partial sequence; internal transcribed spacer 1, 5.8S ribosomal RNA gene and internal transcribed spacer 2, complete sequence; and 26S ribosomal RNA gene, partial sequence	1205	1205	100%	0.0	100.00%	AF448794.1
Equisetum ramosissimum isolate BPTPS123 small subunit ribosomal RNA gene, partial sequence; internal transcribed spacer 1, 5.8S ribosomal RNA gene, and internal transcribed spacer 2, complete sequence; and large subunit ribosomal RNA gene, partial sequence	1037	1037	100%	0.0	95.40%	ON685433.1
Equisetum ramosissimum isolate BPTPS056 small subunit ribosomal RNA gene, partial sequence; internal transcribed spacer 1, 5.8S ribosomal RNA gene, and internal transcribed spacer 2, complete sequence; and large subunit ribosomal RNA gene, partial sequence	1027	1027	100%	0.0	95.11%	OM670239.1
Equisetum hyemale genes for ITS1, 5.8S rRNA, ITS2, partial sequence, bio_material: KB:NIBR0000051239	1003	1003	93%	0.0	96.54%	AB817880.1
Equisetum environmental sample clone EDNA16-0043490 internal transcribed spacer 1, partial sequence; 5.8S ribosomal RNA gene and internal transcribed spacer 2, complete sequence; and large subunit ribosomal RNA gene, partial sequence	780	780	97%	0.0	88.92%	MT198943.1
Equisetum palustre isolate 82716220323 5.8S ribosomal RNA gene, partial sequence; internal transcribed spacer 2, complete sequence; and large subunit ribosomal RNA gene, partial sequence	558	558	62%	3e-154	91.53%	ON713477.1
Equisetum palustre isolate 82714220323 5.8S ribosomal RNA gene, partial sequence; internal transcribed spacer 2, complete sequence; and large subunit ribosomal RNA gene, partial sequence	558	558	62%	3e-154	91.53%	ON713475.1
Equisetum palustre isolate 82713220323 5.8S ribosomal RNA gene, partial sequence; internal transcribed spacer 2, complete sequence; and large subunit ribosomal RNA gene, partial sequence	558	558	62%	3e-154	91.53%	ON713474.1
Equisetum palustre isolate 82712220323 5.8S ribosomal RNA gene, partial sequence; internal transcribed spacer 2, complete sequence; and large subunit ribosomal RNA gene, partial sequence	558	558	62%	3e-154	91.53%	ON713473.1
Equisetum palustre isolate 82715220323 5.8S ribosomal RNA gene, partial sequence; internal transcribed spacer 2, complete sequence; and large subunit ribosomal RNA gene, partial sequence	556	556	62%	1e-153	91.50%	ON713476.1

Description	Max Score	Total Score	Query Cover	E value	Per. Ident	Accession
Equisetum palustre isolate 82711220323 5.8S ribosomal RNA gene, partial sequence; internal transcribed spacer 2, complete sequence; and large subunit ribosomal RNA gene, partial sequence	556	556	62%	1e-153	91.50%	ON713472.1
Equisetum palustre isolate 82710220323 5.8S ribosomal RNA gene, partial sequence; internal transcribed spacer 2, complete sequence; and large subunit ribosomal RNA gene, partial sequence	556	556	62%	1e-153	91.50%	ON713471.1
Equisetum palustre isolate 82709220323 5.8S ribosomal RNA gene, partial sequence; internal transcribed spacer 2, complete sequence; and large subunit ribosomal RNA gene, partial sequence	555	555	62%	4e-153	91.48%	ON713470.1
Equisetum arvense isolate 82704220323 5.8S ribosomal RNA gene and internal transcribed spacer 2, partial sequence	484	484	47%	5e-132	94.89%	ON713479.1
Equisetum arvense isolate 82703220323 5.8S ribosomal RNA gene and internal transcribed spacer 2, partial sequence	484	484	47%	5e-132	94.87%	ON713478.1
Equisetum arvense isolate 82701220323 5.8S ribosomal RNA gene and internal transcribed spacer 2, partial sequence	483	483	45%	2e-131	95.99%	ON876507.1
Equisetum arvense isolate 83919220502 5.8S ribosomal RNA gene and internal transcribed spacer 2, partial sequence	483	483	48%	2e-131	94.60%	ON713484.1
Equisetum arvense isolate 82702220323 5.8S ribosomal RNA gene and internal transcribed spacer 2, partial sequence	470	470	45%	2e-127	95.30%	ON713480.1
Equisetum x ferrissii 18S ribosomal RNA gene, partial sequence; internal transcribed spacer 1, complete sequence; and 5.8S ribosomal RNA gene, partial sequence	468	468	44%	5e-127	95.19%	EU328338.1
Equisetum arvense isolate 84087220504 5.8S ribosomal RNA gene and internal transcribed spacer 2, partial sequence	464	464	48%	7e-126	93.38%	ON713485.1
Equisetum variegatum 18S ribosomal RNA gene, partial sequence; internal transcribed spacer 1, complete sequence; and 5.8S ribosomal RNA gene, partial sequence	453	453	44%	2e-122	94.52%	EU328340.1
Equisetum variegatum voucher B. Hoshizaki 592 18S ribosomal RNA gene, partial sequence; internal transcribed spacer 1, complete sequence; and 5.8S ribosomal RNA gene, partial sequence	438	438	44%	4e-118	93.75%	EU372664.1
Equisetum hyemale subsp. affine 18S ribosomal RNA gene, partial sequence; internal transcribed spacer 1, complete sequence; and 5.8S ribosomal RNA gene, partial sequence	435	435	44%	6e-117	93.40%	EU328339.1
Equisetum variegatum subsp. variegatum internal transcribed spacer 1, partial sequence	418	418	40%	6e-112	95.42%	DQ377155.1

Description	Max Score	Total Score	Query Cover	E value	Per. Ident	Accession
Equisetum hyemale subsp. affine 18S ribosomal RNA gene, partial sequence; internal transcribed spacer 1, complete sequence; and 5.8S ribosomal RNA gene, partial sequence	405	405	42%	4e-108	92.39%	EU328336.1
Equisetum arvense isolate 82708220323 5.8S ribosomal RNA gene and internal transcribed spacer 2, partial sequence	403	403	48%	2e-107	89.91%	ON876508.1
Equisetum laevigatum 18S ribosomal RNA gene, partial sequence; internal transcribed spacer 1, complete sequence; and 5.8S ribosomal RNA gene, partial sequence	398	398	44%	7e-106	90.38%	EU328341.1
Equisetum hyemale subsp. hyemale internal transcribed spacer 1, partial sequence	370	370	35%	2e-97	95.65%	DQ377151.1
Equisetum variegatum voucher B. Hoshizaki 203 18S ribosomal RNA gene, partial sequence; internal transcribed spacer 1, complete sequence; and 5.8S ribosomal RNA gene, partial sequence	368	368	42%	6e-97	90.22%	EU372665.1
Equisetum scirpoides voucher P. Hammond s.n 18S ribosomal RNA gene, partial sequence; internal transcribed spacer 1, complete sequence; and 5.8S ribosomal RNA gene, partial sequence	326	326	42%	3e-84	86.59%	EU372663.1
Equisetum myriochaetum 18S ribosomal RNA gene, partial sequence; internal transcribed spacer 1, complete sequence; and 5.8S ribosomal RNA gene, partial sequence	318	318	42%	6e-82	86.96%	EU328337.1
Equisetum x meridionale internal transcribed spacer 1, partial sequence	316	316	32%	2e-81	93.84%	DQ377156.1
Equisetum x moorei internal transcribed spacer 1, partial sequence	309	309	32%	3e-79	93.30%	DQ377157.1
Equisetum ramosissimum subsp. ramosissimum internal transcribed spacer 1, partial sequence	298	298	31%	8e-76	92.75%	DQ377153.1
Equisetum arvense isolate L internal transcribed spacer 2, partial sequence	268	268	36%	6e-67	87.19%	ON568505.1
Selliguea metacoela voucher R. Wei 2925 (PE) small subunit ribosomal RNA gene, internal transcribed spacer 1, 5.8S ribosomal RNA gene, internal transcribed spacer 2, and large subunit ribosomal RNA gene, complete sequence	267	267	41%	2e-66	84.75%	MZ361045.1
Niphidium crassifolium voucher WR0621 (PE) small subunit ribosomal RNA gene, internal transcribed spacer 1, 5.8S ribosomal RNA gene, internal transcribed spacer 2, and large subunit ribosomal RNA gene, complete sequence	267	267	31%	2e-66	90.34%	MZ361021.1
Microgramma vacciniifolia voucher C.X. Li RS281 small subunit ribosomal RNA gene, internal transcribed spacer 1, 5.8S ribosomal RNA gene, internal transcribed spacer 2, and large subunit ribosomal RNA gene, complete sequence	265	265	38%	8e-66	86.15%	MZ361014.1

Description	Max Score	Total Score	Query Cover	E value	Per. Ident	Accession
Goniophlebium niponicum voucher H.M. Liu A305 (PE) small subunit ribosomal RNA gene, internal transcribed spacer 1, 5.8S ribosomal RNA gene, internal transcribed spacer 2, and large subunit ribosomal RNA gene, complete sequence	263	263	31%	3e-65	89.95%	MZ361035.1
Selliguea chenopus voucher X.C. Zhang 6259 (PE) small subunit ribosomal RNA gene, internal transcribed spacer 1, 5.8S ribosomal RNA gene, internal transcribed spacer 2, and large subunit ribosomal RNA gene, complete sequence	263	263	31%	3e-65	89.95%	MZ361023.1
Lepisorus hederaceus voucher Voucher unknown small subunit ribosomal RNA gene, internal transcribed spacer 1, 5.8S ribosomal RNA gene, internal transcribed spacer 2, and large subunit ribosomal RNA gene, complete sequence	263	263	31%	3e-65	90.00%	MZ361003.1
Goniophlebium persicifolium voucher X.C. Zhang 433 (PE) small subunit ribosomal RNA gene, internal transcribed spacer 1, 5.8S ribosomal RNA gene, internal transcribed spacer 2, and large subunit ribosomal RNA gene, complete sequence	263	263	31%	3e-65	89.95%	MZ360996.1
Adetogramma chrysolepis voucher Alfredo Tupayachi H. 758 (A) small subunit ribosomal RNA gene, internal transcribed spacer 1, 5.8S ribosomal RNA gene, internal transcribed spacer 2, and large subunit ribosomal RNA gene, complete sequence	263	263	37%	3e-65	86.22%	MZ360977.1
Goniophlebium mengtzeense voucher H.M. Liu GX211 (PE) small subunit ribosomal RNA gene, internal transcribed spacer 1, 5.8S ribosomal RNA gene, internal transcribed spacer 2, and large subunit ribosomal RNA gene, complete sequence	261	261	31%	1e-64	89.90%	MZ361033.1
Selliguea griffithiana voucher X.C. Zhang 9580 (PE) small subunit ribosomal RNA gene, internal transcribed spacer 1, 5.8S ribosomal RNA gene, internal transcribed spacer 2, and large subunit ribosomal RNA gene, complete sequence	261	261	31%	1e-64	89.90%	MZ361025.1
Phlebodium pseudoaureum voucher WR0622 (PE) small subunit ribosomal RNA gene, internal transcribed spacer 1, 5.8S ribosomal RNA gene, internal transcribed spacer 2, and large subunit ribosomal RNA gene, complete sequence	261	261	31%	1e-64	89.90%	MZ361022.1
Microsorum steerei voucher R. Wei WR0620 (PE) small subunit ribosomal RNA gene, internal transcribed spacer 1, 5.8S ribosomal RNA gene, internal transcribed spacer 2, and large subunit ribosomal RNA gene, complete sequence	261	261	31%	1e-64	89.95%	MZ361020.1

Description	Max Score	Total Score	Query Cover	E value	Per. Ident	Accession
Goniophlebium formosanum voucher X.C. Zhang 9432 (PE) small subunit ribosomal RNA gene, internal transcribed spacer 1, 5.8S ribosomal RNA gene, internal transcribed spacer 2, and large subunit ribosomal RNA gene, complete sequence	261	261	31%	1e-64	89.90%	MZ360995.1
Selliguea capitellata voucher X.C. Zhang 6341 (PE) small subunit ribosomal RNA gene, internal transcribed spacer 1, 5.8S ribosomal RNA gene, internal transcribed spacer 2, and large subunit ribosomal RNA gene, complete sequence	261	261	31%	1e-64	89.95%	MZ360982.1
Pilularia americana isolate CBP331 18S ribosomal RNA gene, partial sequence; internal transcribed spacer 1, 5.8S ribosomal RNA gene, and internal transcribed spacer 2, complete sequence; and 26S ribosomal RNA gene, partial sequence	259	259	42%	4e-64	83.80%	MF964017.1
Polypodium vulgare voucher B. Liu 28227 (PE) small subunit ribosomal RNA gene, internal transcribed spacer 1, 5.8S ribosomal RNA gene, internal transcribed spacer 2, and large subunit ribosomal RNA gene, complete sequence	259	259	27%	4e-64	92.86%	MZ361039.1
Polypodium sibiricum voucher Zhang 7312 (PE) small subunit ribosomal RNA gene, internal transcribed spacer 1, 5.8S ribosomal RNA gene, internal transcribed spacer 2, and large subunit ribosomal RNA gene, complete sequence	259	259	27%	4e-64	92.86%	MZ361038.1
Goniophlebium argutum voucher R. Wei ST465 (PE) small subunit ribosomal RNA gene, internal transcribed spacer 1, 5.8S ribosomal RNA gene, internal transcribed spacer 2, and large subunit ribosomal RNA gene, complete sequence	259	259	27%	4e-64	92.86%	MZ361032.1
Selliguea taeniata voucher B.Y. Sun & al. 36 (PE) small subunit ribosomal RNA gene, internal transcribed spacer 1, 5.8S ribosomal RNA gene, internal transcribed spacer 2, and large subunit ribosomal RNA gene, complete sequence	259	259	41%	4e-64	84.29%	MZ361026.1
Selliguea crenatopinnata voucher X.C. Zhang 6242 (PE) small subunit ribosomal RNA gene, internal transcribed spacer 1, 5.8S ribosomal RNA gene, internal transcribed spacer 2, and large subunit ribosomal RNA gene, complete sequence	259	259	27%	4e-64	92.86%	MZ361024.1
Loxogramme avenia voucher R. Wei 2761 (PE) small subunit ribosomal RNA gene, internal transcribed spacer 1, 5.8S ribosomal RNA gene, internal transcribed spacer 2, and large subunit ribosomal RNA gene, complete sequence	259	259	27%	4e-64	92.86%	MZ361010.1

Description	Max Score	Total Score	Query Cover	E value	Per. Ident	Accession
Leptochilus macrophyllus voucher R. Wei & al., 441 (PE) small subunit ribosomal RNA gene, internal transcribed spacer 1, 5.8S ribosomal RNA gene, internal transcribed spacer 2, and large subunit ribosomal RNA gene, complete sequence	259	259	27%	4e-64	92.90%	MZ361009.1
Leptochilus decurrens voucher R. Wei WR0267 (PE) small subunit ribosomal RNA gene, internal transcribed spacer 1, 5.8S ribosomal RNA gene, internal transcribed spacer 2, and large subunit ribosomal RNA gene, complete sequence	259	259	27%	4e-64	92.90%	MZ361008.1
Lepisorus schraderi voucher X.C. Zhang 9057 (PE) small subunit ribosomal RNA gene, internal transcribed spacer 1, 5.8S ribosomal RNA gene, internal transcribed spacer 2, and large subunit ribosomal RNA gene, complete sequence	259	259	27%	4e-64	92.90%	MZ361007.1
Lepisorus clathratus voucher Voucher unknown small subunit ribosomal RNA gene, internal transcribed spacer 1, 5.8S ribosomal RNA gene, internal transcribed spacer 2, and large subunit ribosomal RNA gene, complete sequence	259	259	27%	4e-64	92.90%	MZ361004.1
Lepisorus miyoshianus voucher X.C. Zhang 4511 (PE) small subunit ribosomal RNA gene, internal transcribed spacer 1, 5.8S ribosomal RNA gene, internal transcribed spacer 2, and large subunit ribosomal RNA gene, complete sequence	259	259	27%	4e-64	92.90%	MZ360991.1
Leptochilus ellipticus voucher R. Wei WR0211 (PE) small subunit ribosomal RNA gene, internal transcribed spacer 1, 5.8S ribosomal RNA gene, internal transcribed spacer 2, and large subunit ribosomal RNA gene, complete sequence	259	259	27%	4e-64	92.90%	MZ360987.1
Bosmania membranacea subsp. carinata voucher R. Wei WR0410 (PE) small subunit ribosomal RNA gene, internal transcribed spacer 1, 5.8S ribosomal RNA gene, internal transcribed spacer 2, and large subunit ribosomal RNA gene, complete sequence	259	259	27%	4e-64	92.86%	MZ360985.1
Bosmania membranacea var. membranacea voucher R. Wei WR0368 (PE) small subunit ribosomal RNA gene, internal transcribed spacer 1, 5.8S ribosomal RNA gene, internal transcribed spacer 2, and large subunit ribosomal RNA gene, complete sequence	259	259	27%	4e-64	92.86%	MZ360984.1
Selliguea mairei voucher X.C. Zhang 4609 (PE) small subunit ribosomal RNA gene, internal transcribed spacer 1, 5.8S ribosomal RNA gene, internal transcribed spacer 2, and large subunit ribosomal RNA gene, complete sequence	259	259	27%	4e-64	92.86%	MZ360981.1

Description	Max Score	Total Score	Query Cover	E value	Per. Ident	Accession
Drynaria heraclea voucher R. Wei 470 (PE) small subunit ribosomal RNA gene, internal transcribed spacer 1, 5.8S ribosomal RNA gene, internal transcribed spacer 2, and large subunit ribosomal RNA gene, complete sequence	259	259	27%	4e-64	92.86%	MZ360979.1
Drynaria speciosa voucher R. Wei 508 (PE) small subunit ribosomal RNA gene, internal transcribed spacer 1, 5.8S ribosomal RNA gene, internal transcribed spacer 2, and large subunit ribosomal RNA gene, complete sequence	259	259	27%	4e-64	92.86%	MZ360978.1
Azolla filiculoides isolate BG-PG small subunit ribosomal RNA gene, partial sequence; internal transcribed spacer 1, 5.8S ribosomal RNA gene, and internal transcribed spacer 2, complete sequence; and large subunit ribosomal RNA gene, partial sequence	259	259	38%	4e-64	85.34%	MW872256.1
Azolla filiculoides voucher ApSA22 (48) 18S ribosomal RNA gene, partial sequence; internal transcribed spacer 1 and 5.8S ribosomal RNA gene, complete sequence; and internal transcribed spacer 2, partial sequence	259	259	38%	4e-64	85.34%	JX297314.1
Azolla filiculoides voucher Reid & Peters 68 internal transcribed spacer 1, partial sequence; 5.8S ribosomal RNA gene, complete sequence; and internal transcribed spacer 2, partial sequence	259	259	38%	4e-64	85.34%	DQ066494.1
Thylacopteris papillosa voucher R. Wei 345 (PE) small subunit ribosomal RNA gene, internal transcribed spacer 1, 5.8S ribosomal RNA gene, internal transcribed spacer 2, and large subunit ribosomal RNA gene, complete sequence	257	257	32%	1e-63	88.58%	MZ361053.1
Synammia espinosae voucher I.M. Johnston 5306 (NY) small subunit ribosomal RNA gene, internal transcribed spacer 1, 5.8S ribosomal RNA gene, internal transcribed spacer 2, and large subunit ribosomal RNA gene, complete sequence	257	257	31%	1e-63	89.47%	MZ361049.1
Selliguea heterocarpa voucher R. Wei & al. 353 (PE) small subunit ribosomal RNA gene, internal transcribed spacer 1, 5.8S ribosomal RNA gene, internal transcribed spacer 2, and large subunit ribosomal RNA gene, complete sequence	257	257	31%	1e-63	89.42%	MZ361044.1
Pyrrosia penangiana voucher R. Wei 3014 (PE) small subunit ribosomal RNA gene, internal transcribed spacer 1, 5.8S ribosomal RNA gene, internal transcribed spacer 2, and large subunit ribosomal RNA gene, complete sequence	257	257	31%	1e-63	89.52%	MZ361041.1
Goniophlebium amoenum voucher Z.Y. Li 1897 (PE) small subunit ribosomal RNA gene, internal transcribed spacer 1, 5.8S ribosomal RNA gene, internal transcribed spacer 2, and large subunit ribosomal RNA gene, complete sequence	257	257	31%	1e-63	89.47%	MZ361034.1

Description	Max Score	Total Score	Query Cover	E value	Per. Ident	Accession
Selliguea dareiformis voucher R. Wei ST1108 (PE) small subunit ribosomal RNA gene, internal transcribed spacer 1, 5.8S ribosomal RNA gene, internal transcribed spacer 2, and large subunit ribosomal RNA gene, complete sequence	257	257	37%	1e-63	85.88%	MZ361000.1
Aglaomorpha willdenowii voucher X.C. Zhang 9062 (PE) small subunit ribosomal RNA gene, internal transcribed spacer 1, 5.8S ribosomal RNA gene, internal transcribed spacer 2, and large subunit ribosomal RNA gene, complete sequence	257	257	31%	1e-63	89.47%	MZ360993.1
Asplenium scolopendrium isolate Copy2 voucher Vogel SCOL-175 Italy internal transcribed spacer 1, partial sequence; 5.8S ribosomal RNA gene, complete sequence; and internal transcribed spacer 2, partial sequence	257	257	24%	1e-63	96.23%	JX475163.1
Phaeoceros sp. 'Spessart' isolate Spessart internal transcribed spacer 1, partial sequence; 5.8S ribosomal RNA gene and internal transcribed spacer 2, complete sequence; and large subunit ribosomal RNA gene, partial sequence	255	255	24%	5e-63	95.62%	MN585760.1
Pellaea breweri isolate CBP242 18S ribosomal RNA gene, partial sequence; and internal transcribed spacer 1, 5.8S ribosomal RNA gene, and internal transcribed spacer 2, complete sequence	255	255	28%	5e-63	91.94%	MF963964.1
Synammia intermedia voucher C. Scottsberg 432 (NY) small subunit ribosomal RNA gene, internal transcribed spacer 1, 5.8S ribosomal RNA gene, internal transcribed spacer 2, and large subunit ribosomal RNA gene, complete sequence	255	255	31%	5e-63	89.42%	MZ361051.1
Synammia feuillei voucher Lendemer 16329 (NY) small subunit ribosomal RNA gene, internal transcribed spacer 1, 5.8S ribosomal RNA gene, internal transcribed spacer 2, and large subunit ribosomal RNA gene, complete sequence	255	255	31%	5e-63	89.42%	MZ361050.1
Dryopteris decipiens voucher X.C. Zhang 7333 (PE) small subunit ribosomal RNA gene, internal transcribed spacer 1, 5.8S ribosomal RNA gene, internal transcribed spacer 2, and large subunit ribosomal RNA gene, complete sequence	255	255	28%	5e-63	91.94%	MZ360994.1
Drynaria parishii voucher X.C. Zhang 7946 (PE) small subunit ribosomal RNA gene, internal transcribed spacer 1, 5.8S ribosomal RNA gene, internal transcribed spacer 2, and large subunit ribosomal RNA gene, complete sequence	255	255	27%	5e-63	92.31%	MZ360992.1
Leptochilus henryi voucher X.C. Zhang 7487 (PE) small subunit ribosomal RNA gene, internal transcribed spacer 1, 5.8S ribosomal RNA gene, internal transcribed spacer 2, and large subunit ribosomal RNA gene, complete sequence	255	255	26%	5e-63	93.68%	MZ360988.1

Description	Max Score	Total Score	Query Cover	E value	Per. Ident	Accession
Selliguea lehmannii voucher X.C. Zhang 5963 (PE) small subunit ribosomal RNA gene, internal transcribed spacer 1, 5.8S ribosomal RNA gene, internal transcribed spacer 2, and large subunit ribosomal RNA gene, complete sequence	255	255	29%	5e-63	90.45%	MZ360980.1
Dryopteris intermedia internal transcribed spacer 1, partial sequence; 5.8S ribosomal RNA gene, complete sequence; and internal transcribed spacer 2, partial sequence	255	255	28%	5e-63	91.94%	KF977439.1
Asplenium normale isolate Copy1 voucher Hemp 3605 Tanzania internal transcribed spacer 1, partial sequence; 5.8S ribosomal RNA gene, complete sequence; and internal transcribed spacer 2, partial sequence	255	255	28%	5e-63	91.94%	JX475160.1
Asplenium normale isolate Copy1 voucher Ranker 1799 Hawaii internal transcribed spacer 1, partial sequence; 5.8S ribosomal RNA gene, complete sequence; and internal transcribed spacer 2, partial sequence	255	255	28%	5e-63	91.94%	JX475159.1
Selliguea albidoglauca voucher X.C. Zhang 7609 (PE) small subunit ribosomal RNA gene, internal transcribed spacer 1, 5.8S ribosomal RNA gene, internal transcribed spacer 2, and large subunit ribosomal RNA gene, complete sequence	254	254	28%	2e-62	91.10%	MZ361043.1
Pyrrosia angustata voucher Q. Wei wq004 (PE) small subunit ribosomal RNA gene, internal transcribed spacer 1, 5.8S ribosomal RNA gene, internal transcribed spacer 2, and large subunit ribosomal RNA gene, complete sequence	254	254	31%	2e-62	89.37%	MZ361040.1
Polypodium rhodopleuron voucher A.M. Evans & al. 2871 (PE) small subunit ribosomal RNA gene, internal transcribed spacer 1, 5.8S ribosomal RNA gene, internal transcribed spacer 2, and large subunit ribosomal RNA gene, complete sequence	254	254	27%	2e-62	92.31%	MZ361037.1
Polypodium arcanum voucher M. Sundue & al. 3554 (PE) small subunit ribosomal RNA gene, internal transcribed spacer 1, 5.8S ribosomal RNA gene, internal transcribed spacer 2, and large subunit ribosomal RNA gene, complete sequence	254	254	27%	2e-62	92.31%	MZ361036.1
Pleopeltis polypodioides voucher H. Shang 28B (PE) small subunit ribosomal RNA gene, internal transcribed spacer 1, 5.8S ribosomal RNA gene, internal transcribed spacer 2, and large subunit ribosomal RNA gene, complete sequence	254	254	26%	2e-62	93.64%	MZ361030.1
Platycerium bifurcatum voucher X.P. Wei 41503 (PE) small subunit ribosomal RNA gene, internal transcribed spacer 1, 5.8S ribosomal RNA gene, internal transcribed spacer 2, and large subunit ribosomal RNA gene, complete sequence	254	254	28%	2e-62	91.15%	MZ361028.1

Description	Max Score	Total Score	Query Cover	E value	Per. Ident	Accession
Microsorum punctatum voucher Mt. Shiwan Exp. 148 (PE) small subunit ribosomal RNA gene, internal transcribed spacer 1, 5.8S ribosomal RNA gene, internal transcribed spacer 2, and large subunit ribosomal RNA gene, complete sequence	254	254	28%	2e-62	91.85%	MZ361019.1
Leptochilus pteropus voucher R. Wei WR0281 (PE) small subunit ribosomal RNA gene, internal transcribed spacer 1, 5.8S ribosomal RNA gene, internal transcribed spacer 2, and large subunit ribosomal RNA gene, complete sequence	254	254	27%	2e-62	92.35%	MZ361018.1
Goniophlebium manmeiense voucher X.P. Qi Q061 (PE) small subunit ribosomal RNA gene, internal transcribed spacer 1, 5.8S ribosomal RNA gene, internal transcribed spacer 2, and large subunit ribosomal RNA gene, complete sequence	254	254	27%	2e-62	92.35%	MZ361013.1
Lepisorus carnosus voucher Voucher unknown small subunit ribosomal RNA gene, internal transcribed spacer 1, 5.8S ribosomal RNA gene, internal transcribed spacer 2, and large subunit ribosomal RNA gene, complete sequence	254	254	27%	2e-62	92.35%	MZ361002.1
Campyloneurum sphenodes voucher P.C. Standley 34246 (PE) small subunit ribosomal RNA gene, internal transcribed spacer 1, 5.8S ribosomal RNA gene, internal transcribed spacer 2, and large subunit ribosomal RNA gene, complete sequence	254	254	27%	2e-62	92.35%	MZ360986.1

Alignments

Equisetum ramosissimum 18S ribosomal RNA gene, partial sequence; internal transcribed spacer 1, 5.8S ribosomal RNA gene and internal transcribed spacer 2, complete sequence; and 26S ribosomal RNA gene, partial sequence

Sequence ID: **AF448794.1** Length: 652 Number of Matches: 1

Range 1: 1 to 652

Score	Expect	Identities	Gaps	Strand	Frame
1205 bits(652)	0.0()	652/652(100%)	0/652(0%)	Plus/Plus	

Features:

```
Query  1    AAGGATCATTGTCACACATCCGTGCAAACCCTGCGAATCGTGGACGTCCCCCAAGGGGGC  60
            ||||||||||||||||||||||||||||||||||||||||||||||||||||||||||||
Sbjct  1    AAGGATCATTGTCACACATCCGTGCAAACCCTGCGAATCGTGGACGTCCCCCAAGGGGGC  60

Query  61   GGTGGTTGCGGTGCCCGCTTGCGCCCTCGCGTGCGTGGGTGTGGTGCGGCGCCCCGTCCC  120
            ||||||||||||||||||||||||||||||||||||||||||||||||||||||||||||
Sbjct  61   GGTGGTTGCGGTGCCCGCTTGCGCCCTCGCGTGCGTGGGTGTGGTGCGGCGCCCCGTCCC  120

Query  121  CTCCTCTCGGTTGCTTCGGCGGCCGGGGGGTTCGAACCTTCCGCCCCTCCGGGGGCGTGG  180
            ||||||||||||||||||||||||||||||||||||||||||||||||||||||||||||
Sbjct  121  CTCCTCTCGGTTGCTTCGGCGGCCGGGGGGTTCGAACCTTCCGCCCCTCCGGGGGCGTGG  180

Query  181  AGCACTATTGGAACGCGCACCGCCTGAGACTCCCTCGCAAGGGGGAACGCGAAAGAATGA  240
            ||||||||||||||||||||||||||||||||||||||||||||||||||||||||||||
Sbjct  181  AGCACTATTGGAACGCGCACCGCCTGAGACTCCCTCGCAAGGGGGAACGCGAAAGAATGA  240

Query  241  GAACGACTCTCGGCAACGGATATCTTGGCTCTTGCAACGATGAAGAACGCAGCGAAATGC  300
            ||||||||||||||||||||||||||||||||||||||||||||||||||||||||||||
Sbjct  241  GAACGACTCTCGGCAACGGATATCTTGGCTCTTGCAACGATGAAGAACGCAGCGAAATGC  300
```

```
Query  301   GATACGTAGTGTGAATTGCAGAATTCCGCGAATCATCGAGTCTTTGAACGCAACTTGCGC  360
             ||||||||||||||||||||||||||||||||||||||||||||||||||||||||||||
Sbjct  301   GATACGTAGTGTGAATTGCAGAATTCCGCGAATCATCGAGTCTTTGAACGCAACTTGCGC  360

Query  361   CCGAGGGCTCGCGCCCGAGGGCATGTCTGCCTGAGCGTCCTTACAAATCCTACGCCGccc  420
             ||||||||||||||||||||||||||||||||||||||||||||||||||||||||||||
Sbjct  361   CCGAGGGCTCGCGCCCGAGGGCATGTCTGCCTGAGCGTCCTTACAAATCCTACGCCGCCC  420

Query  421   cccccACGGGAGGAGCGGCGGACGTGGCCATCCGTGAGCTCCTTTGGAGCGTGCGGTCGG  480
             ||||||||||||||||||||||||||||||||||||||||||||||||||||||||||||
Sbjct  421   CCCCCACGGGAGGAGCGGCGGACGTGGCCATCCGTGAGCTCCTTTGGAGCGTGCGGTCGG  480

Query  481   CTGAAATGCATCGGCGGCACCGAGCGGCGAGACGAGTCGCGGGGGTGGTCCTTCAACCGT  540
             ||||||||||||||||||||||||||||||||||||||||||||||||||||||||||||
Sbjct  481   CTGAAATGCATCGGCGGCACCGAGCGGCGAGACGAGTCGCGGGGGTGGTCCTTCAACCGT  540

Query  541   TCGCGTCCCCCTCGTGGGGGCGTGCGACGGTCGCGGCTGTCCGCCACGGTGCGAAGCTAG  600
             ||||||||||||||||||||||||||||||||||||||||||||||||||||||||||||
Sbjct  541   TCGCGTCCCCCTCGTGGGGGCGTGCGACGGTCGCGGCTGTCCGCCACGGTGCGAAGCTAG  600

Query  601   AGAGGGCTATCGGGGCGCGCGCCTTCGCGTGCGCCCTCTTACACTGGACCTC  652
             ||||||||||||||||||||||||||||||||||||||||||||||||||||
Sbjct  601   AGAGGGCTATCGGGGCGCGCGCCTTCGCGTGCGCCCTCTTACACTGGACCTC  652
```

Equisetum ramosissimum isolate BPTPS123 small subunit ribosomal RNA gene, partial sequence; internal transcribed spacer 1, 5.8S ribosomal RNA gene, and internal transcribed spacer 2, complete sequence; and large subunit ribosomal RNA gene, partial sequence
Sequence ID: **ON685433.1** Length: 3702 Number of Matches: 1
Range 1: 1534 to 2183

Score	Expect	Identities	Gaps	Strand	Frame
1037 bits(561)	0.0()	622/652(95%)	2/652(0%)	Plus/Plus	

Features:

```
Query  1      AAGGATCATTGTCACACATCCGTGCAAACCCTGCGAATCGTGGACGTCCCCCAAGGGGGC  60
              |||||||||||||||||||||||||||||| |||||| |||||||||||| |||| ||||
Sbjct  1534   AAGGATCATTGTCACACATCCGTGCAAACCATGCGAACCGTGGACGTCCCTCAAGAGGGC  1593

Query  61     GGTGGTTGCGGTGCCCGCTTGCGCCCTCGCGTGCGTGGGTGTGGTGCGGCGCCCCGTCCC  120
              |||||||| |||||||||||||||||| ||| ||||||||| ||||||||||||||||||
Sbjct  1594   GGTGGTTGTGGTGCCCGCTTGCGCCCTTGCGGGCGTGGGTGCGGTGCGGCGCCCCGTCCC  1653

Query  121    CTCCTCTCGGTTGCTTCGGCGGCCGGGGGGTTCGAACCTTCCGCCCCTCCGGGGGCGTGG  180
              ||||||||||  ||| |||||||||||||| |||||||||||||||| ||||||||||||
Sbjct  1654   CTCCTCTCGGCCGCTCCGGCGGCCGGGGGGGTCGAACCTTCCGCCCCCCCGGGGGCGTGG  1713

Query  181    AGCACTATTGGAACGCGCACCGCCTGAGACTCCCTCGCAAGGGGGAACGCGAAAGAATGA  240
              |||||| ||||||||||||||||||||||||||| ||||||||||||| ||||| || ||
Sbjct  1714   AGCACTCTTGGAACGCGCACCGCCTGAGACTCCCCCGCAAGGGGGAACACGAAAAAACGA  1773

Query  241    GAACGACTCTCGGCAACGGATATCTTGGCTCTTGCAACGATGAAGAACGCAGCGAAATGC  300
              ||||||||||||||||||||||||||||||||||||||||||||||||||||||||||||
Sbjct  1774   GAACGACTCTCGGCAACGGATATCTTGGCTCTTGCAACGATGAAGAACGCAGCGAAATGC  1833

Query  301    GATACGTAGTGTGAATTGCAGAATTCCGCGAATCATCGAGTCTTTGAACGCAACTTGCGC  360
              ||||||||||||||||||||||||||||||||||||||||||||||||||||||||||||
Sbjct  1834   GATACGTAGTGTGAATTGCAGAATTCCGCGAATCATCGAGTCTTTGAACGCAACTTGCGC  1893

Query  361    CCGAGGGCTCGCGCCCGAGGGCATGTCTGCCTGAGCGTCCTTACAAATCCTACGCCGccc  420
              ||||||||||||||||||||||||||||||||||||||||||||||||||||||||||| 
Sbjct  1894   CCGAGGGCTCGCGCCCGAGGGCATGTCTGCCTGAGCGTCCTTACAAATCCTACGCCGCCT  1953

Query  421    cccccACGGGAGGAGCGGCGGACGTGGCCATCCGTGAGCTCCTTTGGAGCGTGCGGTCGG  480
              |||||||||| || |||||||||||||||||||| |||||||||||||||||||||||||
Sbjct  1954   CCCCCACGGGGGGTGCGGCGGACGTGGCCATCCGCGAGCTCCTTTGGAGCGTGCGGTCGG  2013

Query  481    CTGAAATGCATCGGCGGCACCGAGCGGCGAGACGAGTCGCGGGGGTGGTCCTTCAACCGT  540
              ||||||||||||||||||||||||||| ||||||||||||||||||||||||||||||| 
Sbjct  2014   CTGAAATGCATCGGCGGCACCGAGCGGTGAGACGAGTCGCGGGGGTGGTCCTTCAACCGC  2073

Query  541    TCGCGTCCCCCTCGTGGGGGCGTGCGACGGTCGCGGCTGTCCGCCACGGTGCGAAGCTAG  600
              |||||||||||||||||||||||||| |||||||||| ||||||||||||||||||||||
Sbjct  2074   TCGCGTCCCCCTCGTGGGGGCGTGCGGCGGTCGCGGCCGTCCGCCACGGTGCGAAGCTAG  2133

Query  601    AGAGGGCTATCGGGGCGCGCGCCTTCGCGTGCGCCCTCTTACACTGGACCTC  652
              |||||||||||||| || ||||| |||||||||||||| |||||||||||||
Sbjct  2134   AGAGGGCTATCGGG-CGTGCGCC-TCGCGTGCGCCCTCCTACACTGGACCTC  2183
```

Equisetum ramosissimum isolate BPTPS056 small subunit ribosomal RNA gene, partial sequence; internal transcribed spacer 1, 5.8S ribosomal RNA gene, and internal transcribed spacer 2, complete sequence; and large subunit ribosomal RNA gene, partial sequence
Sequence ID: **OM670239.1** Length: 3631 Number of Matches: 1

Range 1: 1490 to 2141

Score	Expect	Identities	Gaps	Strand	Frame
1027 bits(556)	0.0()	622/654(95%)	4/654(0%)	Plus/Plus	

Features:

```
Query  1     AAGGATCATTGTCACACATCCGTGCAAACCCTGCGAATCGTGGACGTCCCCCAAGGGGGC  60
             ||||||||||||||||||||||||||||||||| ||| |||||| ||||||||| |||||
Sbjct  1490  AAGGATCATTGTCACACATCCGTGCAAACCCTGTGAACCGTGGATGTCCCCCAAAGGGGC  1549

Query  61    GGTGGTTGCGGTGCCCGCTTGCGCCCTCGCGTGCGTGGGTGTGGTGCGGCG-CCCCGTCC  119
             |||||||| |||||||||||| ||||||||| ||||||||||||||||||| |||| |||
Sbjct  1550  GGTGGTTGTGGTGCCCGCTTGTGCCCTCGCGGGCGTGGGTGTGGTGCGGCGCCCCCTTCC  1609

Query  120   CCTCCTCTCGGTTGCTTCGGCGGCCGGGGGGTTCGAACCTTCCGCCCCTCCGGGGGCGTG  179
             |||||| ||||| ||| |||||||||||||||||||||||||||||||||||||||||||
Sbjct  1610  CCTCCTTTCGGTCGCTCCGGCGGCCGGGGGGTTCGAACCTTCCGCCCCTCCGGGGGCGTG  1669

Query  180   GAGCACTATTGGAACGCGCACCGCCTGAGACTCCCTCGCAAGGGGGAACGCGAAAGAATG  239
             ||||||| ||||||||||||||||||||||||||| ||||||||||||| ||||| || |
Sbjct  1670  GAGCACTCTTGGAACGCGCACCGCCTGAGACTCCCCCGCAAGGGGGAACACGAAAAAACG  1729

Query  240   AGAACGACTCTCGGCAACGGATATCTTGGCTCTTGCAACGATGAAGAACGCAGCGAAATG  299
             ||||||||||||||||||||||||||||||||||||||||||||||||||||||||||||
Sbjct  1730  AGAACGACTCTCGGCAACGGATATCTTGGCTCTTGCAACGATGAAGAACGCAGCGAAATG  1789

Query  300   CGATACGTAGTGTGAATTGCAGAATTCCGCGAATCATCGAGTCTTTGAACGCAACTTGCG  359
             ||||||||||||||||||||||||||||||||||||||||||||||||||||||||||||
Sbjct  1790  CGATACGTAGTGTGAATTGCAGAATTCCGCGAATCATCGAGTCTTTGAACGCAACTTGCG  1849

Query  360   CCCGAGGGCTCGCGCCCGAGGGCATGTCTGCCTGAGCGTCCTTACAAATCCTACGCCGcc  419
             ||||||||||||||||||||||||||||||||||||||||||||||||||||||||||||
Sbjct  1850  CCCGAGGGCTCGCGCCCGAGGGCATGTCTGCCTGAGCGTCCTTACAAATCCTACGCCGCC  1909

Query  420   ccccccACGGGAGGA-GCGGCGGACGTGGCCATCCGTGAGCTCCTTTGGAGCGTGCGGTC  478
              |||||||||| ||  |||||||| |||||||||||||||||||||||||||||||||||
Sbjct  1910  TCCCCCACGGGGGGGTGCGGCGGATGTGGCCATCCGTGAGCTCCTTTGGAGCGTGCGGTC  1969

Query  479   GGCTGAAATGCATCGGCGGCACCGAGCGGCGAGACGAGTCGCGGGGGTGGTCCTTCAACC  538
             ||||||||||||||||||||||||||||| ||||||||||||||||||||||||||||||
Sbjct  1970  GGCTGAAATGCATCGGCGGCACCGAGCGGTGAGACGAGTCGCGGGGGTGGTCCTTCAACC  2029

Query  539   GTTCGCGTCCCCCTCGTGGGGGCGTGCGACGGTCGCGGCTGTCCGCCACGGTGCGAAGCT  598
             | |||||||||||||||||||||||||| |||||||||| || |||||||||||||||||
Sbjct  2030  GCTCGCGTCCCCCTCGTGGGGGCGTGCGGCGGTCGCGGCCGTTCGCCACGGTGCGAAGCT  2089

Query  599   AGAGAGGGCTATCGGGGCGCGCGCCTTCGCGTGCGCCCTCTTACACTGGACCTC  652
             |||||||||||||||| || ||||||  |||||||||||| |||||||||||||
Sbjct  2090  AGAGAGGGCTATCGGG-CGTGCGCCTA-GCGTGCGCCCTCCTACACTGGACCTC  2141
```

Equisetum hyemale genes for ITS1, 5.8S rRNA, ITS2, partial sequence, bio_material: KB:NIBR0000051239

Sequence ID: **AB817880.1** Length: 606 Number of Matches: 1

See 9 more title(s)

Range 1: 1 to 606

Score	Expect	Identities	Gaps	Strand	Frame
1003 bits(543)	0.0()	586/607(97%)	1/607(0%)	Plus/Plus	

Features:

```
Query  16    ACATCCGTGCAAACCCTGCGAATCGTGGACGTCCCCCAAGGGGGCGGTGGTTGCGGTGCC  75
             ||||||||||||||||||||||||||||||||||||||||||||||||||||||||||| 
Sbjct  1     ACATCCGTGCAAACCCTGCGAATCGTGGACGTCCCCCAAGGGGGCGGTGGTTGCGGTGCT  60

Query  76    CGCTTGCGCCCTCGCGTGCGTGGGTGTGGTGCGGCGCCCCGTCCCCTCCTCTCGGTTGCT  135
             |||||||||||||||| ||||||||| |||||||||||||| |||||||||||||  |||
Sbjct  61    CGCTTGCGCCCTCGCGGGCGTGGGTGCGGTGCGGCGCCCCGCCCCCTCCTCTCGGCGGCT  120

Query  136   TCGGCGGCCGGGGGGTTCGAACCTTCCGCCCCTCCGGGGGCGTGGAGCACTATTGGAACG  195
              |||||||||||||| ||||||||||||||||||||||||||||||||||||||||||||
Sbjct  121   CCGGCGGCCGGGGGGGTCGAACCTTCCGCCCCTCCGGGGGCGTGGAGCACTATTGGAACG  180

Query  196   CGCACCGCCTGAGACTCCCTCGCAAGGGGGAACGCGAAAGAATGAGAACGACTCTCGGCA  255
             ||||  ||||||||||||| ||||||||||||||||||| || |||||||||||||||||
Sbjct  181   CGCATTGCCTGAGACTCCCCCGCAAGGGGGAACGCGAAAAAACGAGAACGACTCTCGGCA  240

Query  256   ACGGATATCTTGGCTCTTGCAACGATGAAGAACGCAGCGAAATGCGATACGTAGTGTGAA  315
             ||||||||||||||||||||||||||||||||||||||||||||||||||||||||||||
Sbjct  241   ACGGATATCTTGGCTCTTGCAACGATGAAGAACGCAGCGAAATGCGATACGTAGTGTGAA  300

Query  316   TTGCAGAATTCCGCGAATCATCGAGTCTTTGAACGCAACTTGCGCCCGAGGGCTCGCGCC  375
             ||||||||||||||||||||||||||||||||||||||||||||||||||||||||||||
Sbjct  301   TTGCAGAATTCCGCGAATCATCGAGTCTTTGAACGCAACTTGCGCCCGAGGGCTCGCGCC  360
```

```
Query  376   CGAGGGCATGTCTGCCTGAGCGTCCTTACAAATCCTACGCCGccccccccACGGGAGGAG  435
             |||||||||||||||||||||||||||||||||||||||||||| |||||||||| ||||
Sbjct  361   CGAGGGCATGTCTGCCTGAGCGTCCTTACAAATCCTACGCCGCCTCCCCCACGGGGGGAG  420

Query  436   CGGCGGACGTGGCCATCCGTGAGCTCCTTTGGAGCGTGCGGTCGGCTGAAATGCATCGGC  495
             ||||||||||||||||||| ||||||||||||||||||||||||||||||||||||||||
Sbjct  421   CGGCGGACGTGGCCATCCGCGAGCTCCTTTGGAGCGTGCGGTCGGCTGAAATGCATCGGC  480

Query  496   GGCACCGAGCGGCGAGACGAGTCGCGGGGGTGGTCCTTCAACCGTTCGCGTCCCCCTCGT  555
             ||||||||||||||||||||||||||||||||||||||||||||||||||||||||||| 
Sbjct  481   GGCACCGAGCGGCGAGACGAGTCGCGGGGGTGGTCCTTCAACCGTTCGCGTCCCCCTCGC  540

Query  556   GGGGGCGTGCGACGGTCGCGGCTGTCCGCCACGGTGCGAAGCTAGAGAGGGCTATCGGGG  615
             ||||||||||| |||||||||| |||||||||||||||||||||||||||||||||||| 
Sbjct  541   GGGGGCGTGCGGCGGTCGCGGCCGTCCGCCACGGTGCGAAGCTAGAGAGGGCTATCGGG-  599

Query  616   CGCGCGC  622
             || ||||
Sbjct  600   CGTGCGC  606
```

Equisetum environmental sample clone EDNA16-0043490 internal transcribed spacer 1, partial sequence; 5.8S ribosomal RNA gene and internal transcribed spacer 2, complete sequence; and large subunit ribosomal RNA gene, partial sequence

Sequence ID: **MT198943.1** Length: 739 Number of Matches: 1

Range 1: 1 to 633

Score	Expect	Identities	Gaps	Strand	Frame
780 bits(422)	0.0()	570/641(89%)	13/641(2%)	Plus/Plus	

Features:

```
Query  17    CATCCGTGCAAACCCTGCGAATCGTGGACGTCCCCCAAGGGGGCGGTGGTTGCGGTGCCC  76
             |||||||||||||||||||||  ||||||||||||||||||||||| || | || |||||
Sbjct  1     CATCCGTGCAAACCCTGCGAACTGTGGACGTCCCCCAAGGGGGCGGCGGCTCCGTTGCCC  60

Query  77    GCTTGCGCCCTCGCGTGCGTGGGTGTGGTGCGGCGCCCCGTCCCCTCCTCTCGGTTGCTT  136
              |  ||||||||||| ||| ||| | || ||||||||||||||||||||| |||  ||| 
Sbjct  61    TCCCGCGCCCTCGCGGGCGCGGGCGCGGCGCGGCGCCCCGTCCCCTCCTCCCGGCCGCTC  120

Query  137   CGGCGGCCGGGGGGTTCGAACCTTCCGCCCCTCCGGGGGCGTGGAGCACTATTGGAACGC  196
             ||||| |||||||| |||||||||||||||||||||||||| |||||||| || |||| |
Sbjct  121   CGGCGTCCGGGGGGGTCGAACCTTCCGCCCCTCCGGGGGCGCGGAGCACTCTTCGAACTC  180

Query  197   -GCACCGCCTGAGACTCCCTCGCAAGGGGGAACGCGAAAGAAT-GAGAACGACTCTCGGC  254
                |||| ||||||||||| |||| |||||||| ||||| ||| ||||||||||||||||
Sbjct  181   TAAACCGTCTGAGACTCCCCCGCACGGGGGAACACGAAA-AATCGAGAACGACTCTCGGC  239

Query  255   AACGGATATCTTGGCTCTTGCAACGATGAAGAACGCAGCGAAATGCGATACGTAGTGTGA  314
             ||||||||||||||||||||||||||||||||||||||||||||||||||||||||||||
Sbjct  240   AACGGATATCTTGGCTCTTGCAACGATGAAGAACGCAGCGAAATGCGATACGTAGTGTGA  299

Query  315   ATTGCAGAATTCCGCGAATCATCGAGTCTTTGAACGCAACTTGCGCCCGAGGGCTCGCGC  374
             ||||||||||||||||||||||||| ||||||||||||||| |||||||||||||  |||
Sbjct  300   ATTGCAGAATTCCGCGAATCATCGAATCTTTGAACGCAACTCGCGCCCGAGGGCT--CGC  357

Query  375   CCGAGGGCATGTCTGCCTGAGCGTCCTTACAAATCCTACGCCGcc-ccccccACGGGAGG  433
             ||||||||||||||||||||||||||||||||||||||||||||| |||||| |||| ||
Sbjct  358   CCGAGGGCATGTCTGCCTGAGCGTCCTTACAAATCCTACGCCGCCTCCCCCCGCGGGGGG  417

Query  434   AGCGGCGGACGTGGCCATCCGTGAGCTCCTTTGGAGCGTGCGGTCGGCTGAAATGCATCG  493
             |||||||||| ||||| |||| || |||||  |||||| |||||||||||||||||||||
Sbjct  418   AGCGGCGGACCTGGCCGTCCGCGAACTCCTCCGGAGCGCGCGGTCGGCTGAAATGCATCG  477

Query  494   GCGGCACCGAGCGGCGAGACGAGTCGCGGGGGTGGTCCTTCAACCGTTCGC-GTCCCCCT  552
             |||||||||||||||||||||||||||||||||||||||||| ||||| ||   ||||||
Sbjct  478   GCGGCACCGAGCGGCGAGACGAGTCGCGGGGGTGGTCCTTCACCCGTT-GCAAGCCCCCT  536

Query  553   CGTGGGGGCGTGCGACGGTCGCGGCTGTCCGCCACGGTGCGAAGCTAGAGAGGGCTATCG  612
             || |||||| |||| |||||||||| ||||||||||||||||| |  || |||||| | |
Sbjct  537   CGCGGGGGCTTGCGGCGGTCGCGGCCGTCCGCCACGGTGCGAARCCGGAAAGGGCTTTAG  596

Query  613   GGGCGCGCGCCTTCGCGTGCG-CCCTCTTACACTGGACCTC  652
             ||  |||| | | |||| ||  ||||| |||||||||||||
Sbjct  597   GGT-GCGC-C-T-CGCGCGCTCCCCTCCTACACTGGACCTC  633
```

BLAST is a registered trademark of the National Library of Medicine

Support center Mailing list YouTube

- National Library Of Medicine
- National Institutes Of Health
- U.S. Department of Health & Human Services
- USA.gov

NCBI

National Center for Biotechnology Information, U.S. National Library of Medicine 8600 Rockville Pike, Bethesda MD, 20894 USA

Policies and Guidelines | Contact

HHS Vulnerability Disclosure
