# Supplementary material for: A Multi-Pronged Technique for Identifying Equisetum palustre and Equisetum arvense—Combining HPTLC, HPLC-ESI-MS/MS and Optimized DNA Barcoding Techniques
Source: Plants (Basel). 2022 Sep 28;11(19):2562. doi: 10.3390/plants11192562 (PMC9573248; doi:10.3390/plants11192562)
Supplement: Supplementary file 1 [file plants-11-02562-s001.zip › plants-1932149 supplementary/Horsetail Supplementary Figures/Figure S14.pdf]

**BLAST Results**

Questions/comments

**Job title: KT960212.1**

| | | | |
|---|---|---|---|
| **RID** | FD4RGWE0016 (Expires on 08-14 01:50 am) | | |
| **Query ID** | lcl\|Query_41523 | **Database Name** | nt |
| **Description** | None | **Description** | Nucleotide collection (nt) |
| **Molecule type** | dna | **Program** | BLASTN 2.13.0+ |
| **Query Length** | 343 | | |

## Graphic Summary

**Distribution of the top 100 Blast Hits on 100 subject sequences**

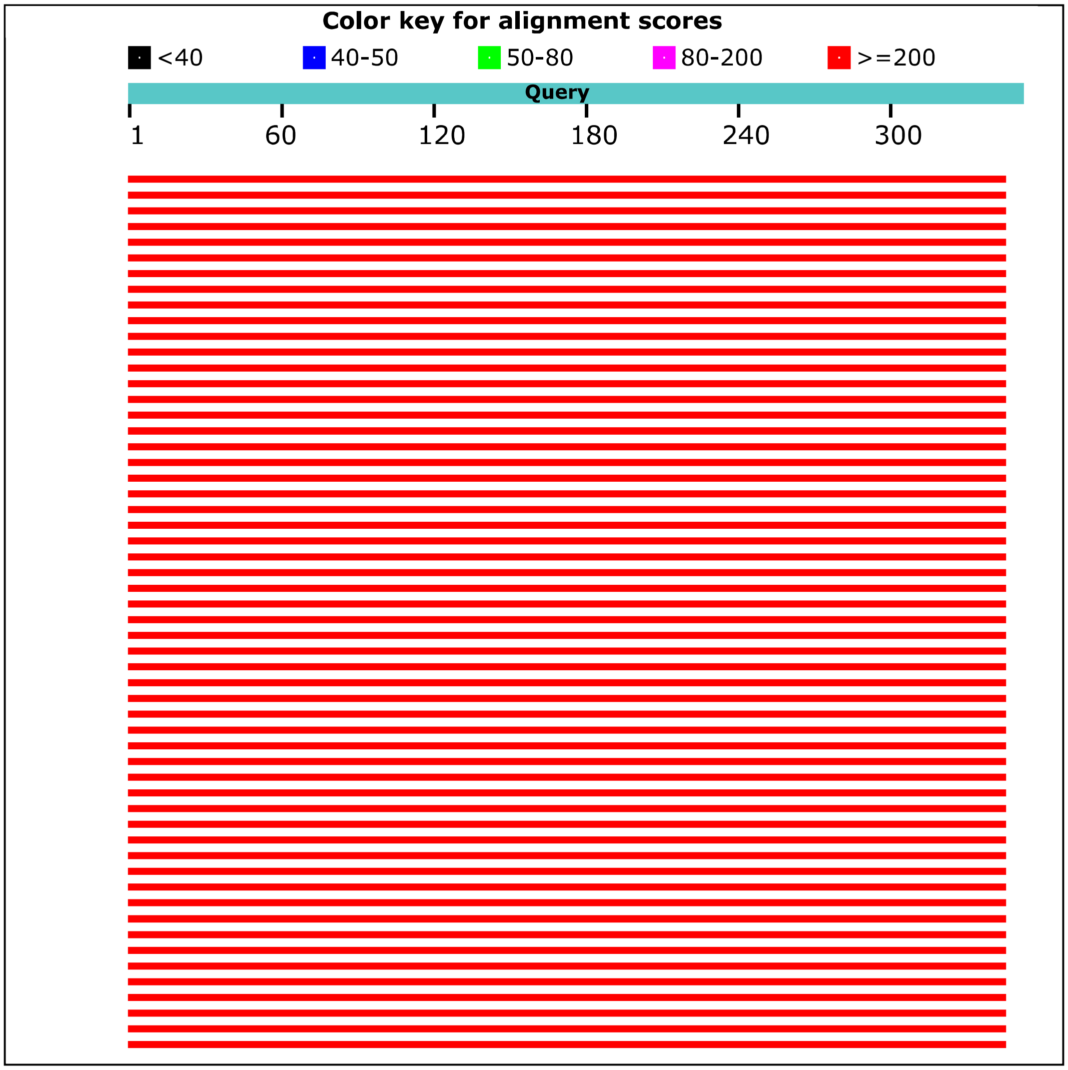

## Descriptions

Sequences producing significant alignments:

| Description | Max Score | Total Score | Query Cover | E value | Per. Ident | Accession |
|---|---|---|---|---|---|---|
| Equisetum variegatum voucher ZA2012-2553 internal transcribed spacer 2, partial sequence | 619 | 619 | 100% | 7e-173 | 100.00% | KT960212.1 |
| Trebouxia sp. Hap09 internal transcribed spacer 1, partial sequence; 5.8S ribosomal RNA gene and internal transcribed spacer 2, complete sequence; and large subunit ribosomal RNA gene, partial sequence | 608 | 608 | 100% | 2e-169 | 98.25% | MN832493.1 |
| Trebouxia sp. Hap07 internal transcribed spacer 1, partial sequence; 5.8S ribosomal RNA gene and internal transcribed spacer 2, complete sequence; and large subunit ribosomal RNA gene, partial sequence | 608 | 608 | 100% | 2e-169 | 98.25% | MN832491.1 |
| Trebouxia sp. Hap06 internal transcribed spacer 1, partial sequence; 5.8S ribosomal RNA gene and internal transcribed spacer 2, complete sequence; and large subunit ribosomal RNA gene, partial sequence | 608 | 608 | 100% | 2e-169 | 98.25% | MN832490.1 |
| Trebouxia impressa isolate L2239p internal transcribed spacer 1, partial sequence; 5.8S ribosomal RNA gene, complete sequence; and internal transcribed spacer 2, partial sequence | 608 | 608 | 100% | 2e-169 | 98.25% | KX181276.1 |
| Trebouxia impressa isolate L2238p internal transcribed spacer 1, partial sequence; 5.8S ribosomal RNA gene, complete sequence; and internal transcribed spacer 2, partial sequence | 608 | 608 | 100% | 2e-169 | 98.25% | KX181275.1 |
| Trebouxia flava isolate L3229 internal transcribed spacer 1, partial sequence; 5.8S ribosomal RNA gene and internal transcribed spacer 2, complete sequence; and large subunit ribosomal RNA gene, partial sequence | 608 | 608 | 100% | 2e-169 | 98.25% | OM275696.1 |
| Trebouxia flava isolate L3228 internal transcribed spacer 1, partial sequence; 5.8S ribosomal RNA gene and internal transcribed spacer 2, complete sequence; and large subunit ribosomal RNA gene, partial sequence | 608 | 608 | 100% | 2e-169 | 98.25% | OM275695.1 |
| Trebouxia flava isolate L3227 internal transcribed spacer 1, partial sequence; 5.8S ribosomal RNA gene and internal transcribed spacer 2, complete sequence; and large subunit ribosomal RNA gene, partial sequence | 608 | 608 | 100% | 2e-169 | 98.25% | OM275694.1 |
| Trebouxia flava isolate L3012 internal transcribed spacer 1, partial sequence; 5.8S ribosomal RNA gene and internal transcribed spacer 2, complete sequence; and large subunit ribosomal RNA gene, partial sequence | 608 | 608 | 100% | 2e-169 | 98.25% | OM275632.1 |

| Description | Max Score | Total Score | Query Cover | E value | Per. Ident | Accession |
|---|---|---|---|---|---|---|
| Trebouxia flava isolate L2725 internal transcribed spacer 1, partial sequence; 5.8S ribosomal RNA gene and internal transcribed spacer 2, complete sequence; and large subunit ribosomal RNA gene, partial sequence | 608 | 608 | 100% | 2e-169 | 98.25% | OM275516.1 |
| Uncultured Trebouxia photobiont partial 18S rRNA gene, ITS1, 5.8S rRNA gene, ITS2 and partial 26S rRNA gene, clone L-475-t2 | 608 | 608 | 100% | 2e-169 | 98.25% | AM159209.1 |
| Trebouxia impressa isolate L4130 internal transcribed spacer 1, partial sequence; 5.8S ribosomal RNA gene and internal transcribed spacer 2, complete sequence; and large subunit ribosomal RNA gene, partial sequence | 603 | 603 | 100% | 7e-168 | 97.96% | OM275768.1 |
| Trebouxia impressa isolate L4129 internal transcribed spacer 1, partial sequence; 5.8S ribosomal RNA gene and internal transcribed spacer 2, complete sequence; and large subunit ribosomal RNA gene, partial sequence | 603 | 603 | 100% | 7e-168 | 97.96% | OM275767.1 |
| Trebouxia impressa isolate L3336 internal transcribed spacer 1, partial sequence; 5.8S ribosomal RNA gene and internal transcribed spacer 2, complete sequence; and large subunit ribosomal RNA gene, partial sequence | 603 | 603 | 100% | 7e-168 | 97.96% | OM275707.1 |
| Trebouxia impressa isolate L3317 internal transcribed spacer 1, partial sequence; 5.8S ribosomal RNA gene and internal transcribed spacer 2, complete sequence; and large subunit ribosomal RNA gene, partial sequence | 603 | 603 | 100% | 7e-168 | 97.96% | OM275705.1 |
| Trebouxia flava isolate L2513 internal transcribed spacer 1, partial sequence; 5.8S ribosomal RNA gene and internal transcribed spacer 2, complete sequence; and large subunit ribosomal RNA gene, partial sequence | 603 | 603 | 100% | 7e-168 | 97.96% | OM275494.1 |
| Trebouxia flava isolate L2567 internal transcribed spacer 1, partial sequence; 5.8S ribosomal RNA gene and internal transcribed spacer 2, complete sequence; and large subunit ribosomal RNA gene, partial sequence | 599 | 599 | 100% | 9e-167 | 97.67% | OM275498.1 |
| Trebouxia flava isolate L4196 internal transcribed spacer 1, partial sequence; 5.8S ribosomal RNA gene and internal transcribed spacer 2, complete sequence; and large subunit ribosomal RNA gene, partial sequence | 597 | 597 | 100% | 3e-166 | 97.67% | OM275791.1 |
| Trebouxia flava isolate L4164 internal transcribed spacer 1, partial sequence; 5.8S ribosomal RNA gene and internal transcribed spacer 2, complete sequence; and large subunit ribosomal RNA gene, partial sequence | 597 | 597 | 100% | 3e-166 | 97.67% | OM275781.1 |

| Description | Max Score | Total Score | Query Cover | E value | Per. Ident | Accession |
|---|---|---|---|---|---|---|
| Trebouxia flava isolate L4154 internal transcribed spacer 1, partial sequence; 5.8S ribosomal RNA gene and internal transcribed spacer 2, complete sequence; and large subunit ribosomal RNA gene, partial sequence | 597 | 597 | 100% | 3e-166 | 97.67% | OM275774.1 |
| Trebouxia flava isolate L4153 internal transcribed spacer 1, partial sequence; 5.8S ribosomal RNA gene and internal transcribed spacer 2, complete sequence; and large subunit ribosomal RNA gene, partial sequence | 597 | 597 | 100% | 3e-166 | 97.67% | OM275773.1 |
| Trebouxia flava isolate L4119 internal transcribed spacer 1, partial sequence; 5.8S ribosomal RNA gene and internal transcribed spacer 2, complete sequence; and large subunit ribosomal RNA gene, partial sequence | 597 | 597 | 100% | 3e-166 | 97.67% | OM275763.1 |
| Trebouxia flava isolate L4118 internal transcribed spacer 1, partial sequence; 5.8S ribosomal RNA gene and internal transcribed spacer 2, complete sequence; and large subunit ribosomal RNA gene, partial sequence | 597 | 597 | 100% | 3e-166 | 97.67% | OM275762.1 |
| Trebouxia flava isolate L3955 internal transcribed spacer 1, partial sequence; 5.8S ribosomal RNA gene and internal transcribed spacer 2, complete sequence; and large subunit ribosomal RNA gene, partial sequence | 597 | 597 | 100% | 3e-166 | 97.67% | OM275761.1 |
| Trebouxia flava isolate L3915 internal transcribed spacer 1, partial sequence; 5.8S ribosomal RNA gene and internal transcribed spacer 2, complete sequence; and large subunit ribosomal RNA gene, partial sequence | 597 | 597 | 100% | 3e-166 | 97.67% | OM275757.1 |
| Trebouxia flava isolate L3913 internal transcribed spacer 1, partial sequence; 5.8S ribosomal RNA gene and internal transcribed spacer 2, complete sequence; and large subunit ribosomal RNA gene, partial sequence | 597 | 597 | 100% | 3e-166 | 97.67% | OM275756.1 |
| Trebouxia flava isolate L3910 internal transcribed spacer 1, partial sequence; 5.8S ribosomal RNA gene and internal transcribed spacer 2, complete sequence; and large subunit ribosomal RNA gene, partial sequence | 597 | 597 | 100% | 3e-166 | 97.67% | OM275755.1 |
| Trebouxia flava isolate L3904 internal transcribed spacer 1, partial sequence; 5.8S ribosomal RNA gene and internal transcribed spacer 2, complete sequence; and large subunit ribosomal RNA gene, partial sequence | 597 | 597 | 100% | 3e-166 | 97.67% | OM275754.1 |
| Trebouxia flava isolate L3902 internal transcribed spacer 1, partial sequence; 5.8S ribosomal RNA gene and internal transcribed spacer 2, complete sequence; and large subunit ribosomal RNA gene, partial sequence | 597 | 597 | 100% | 3e-166 | 97.67% | OM275753.1 |

| Description | Max Score | Total Score | Query Cover | E value | Per. Ident | Accession |
|---|---|---|---|---|---|---|
| Trebouxia flava isolate L3725 internal transcribed spacer 1, partial sequence; 5.8S ribosomal RNA gene and internal transcribed spacer 2, complete sequence; and large subunit ribosomal RNA gene, partial sequence | 597 | 597 | 100% | 3e-166 | 97.67% | OM275748.1 |
| Trebouxia flava isolate L3722 internal transcribed spacer 1, partial sequence; 5.8S ribosomal RNA gene and internal transcribed spacer 2, complete sequence; and large subunit ribosomal RNA gene, partial sequence | 597 | 597 | 100% | 3e-166 | 97.67% | OM275746.1 |
| Trebouxia flava isolate L3538 internal transcribed spacer 1, partial sequence; 5.8S ribosomal RNA gene and internal transcribed spacer 2, complete sequence; and large subunit ribosomal RNA gene, partial sequence | 597 | 597 | 100% | 3e-166 | 97.67% | OM275730.1 |
| Trebouxia flava isolate L3484 internal transcribed spacer 1, partial sequence; 5.8S ribosomal RNA gene and internal transcribed spacer 2, complete sequence; and large subunit ribosomal RNA gene, partial sequence | 597 | 597 | 100% | 3e-166 | 97.67% | OM275725.1 |
| Trebouxia flava isolate L3481 internal transcribed spacer 1, partial sequence; 5.8S ribosomal RNA gene and internal transcribed spacer 2, complete sequence; and large subunit ribosomal RNA gene, partial sequence | 597 | 597 | 100% | 3e-166 | 97.67% | OM275724.1 |
| Trebouxia flava isolate L3439 internal transcribed spacer 1, partial sequence; 5.8S ribosomal RNA gene and internal transcribed spacer 2, complete sequence; and large subunit ribosomal RNA gene, partial sequence | 597 | 597 | 100% | 3e-166 | 97.67% | OM275718.1 |
| Trebouxia flava isolate L3422 internal transcribed spacer 1, partial sequence; 5.8S ribosomal RNA gene and internal transcribed spacer 2, complete sequence; and large subunit ribosomal RNA gene, partial sequence | 597 | 597 | 100% | 3e-166 | 97.67% | OM275717.1 |
| Trebouxia flava isolate L3419 internal transcribed spacer 1, partial sequence; 5.8S ribosomal RNA gene and internal transcribed spacer 2, complete sequence; and large subunit ribosomal RNA gene, partial sequence | 597 | 597 | 100% | 3e-166 | 97.67% | OM275716.1 |
| Trebouxia flava isolate L3358 internal transcribed spacer 1, partial sequence; 5.8S ribosomal RNA gene and internal transcribed spacer 2, complete sequence; and large subunit ribosomal RNA gene, partial sequence | 597 | 597 | 100% | 3e-166 | 97.67% | OM275710.1 |
| Trebouxia flava isolate L3340 internal transcribed spacer 1, partial sequence; 5.8S ribosomal RNA gene and internal transcribed spacer 2, complete sequence; and large subunit ribosomal RNA gene, partial sequence | 597 | 597 | 100% | 3e-166 | 97.67% | OM275708.1 |

| Description | Max Score | Total Score | Query Cover | E value | Per. Ident | Accession |
|---|---|---|---|---|---|---|
| Trebouxia flava isolate L3293 internal transcribed spacer 1, partial sequence; 5.8S ribosomal RNA gene and internal transcribed spacer 2, complete sequence; and large subunit ribosomal RNA gene, partial sequence | 597 | 597 | 100% | 3e-166 | 97.67% | OM275703.1 |
| Trebouxia sp. phot1688 internal transcribed spacer 1, partial sequence; 5.8S ribosomal RNA gene and internal transcribed spacer 2, complete sequence; and 28S ribosomal RNA gene, partial sequence | 597 | 597 | 100% | 3e-166 | 97.67% | KC757601.1 |
| Trebouxia sp. phot1686 internal transcribed spacer 1, partial sequence; 5.8S ribosomal RNA gene and internal transcribed spacer 2, complete sequence; and 28S ribosomal RNA gene, partial sequence | 597 | 597 | 100% | 3e-166 | 97.67% | KC757600.1 |
| Trebouxia sp. phot1685 internal transcribed spacer 1, partial sequence; 5.8S ribosomal RNA gene and internal transcribed spacer 2, complete sequence; and 28S ribosomal RNA gene, partial sequence | 597 | 597 | 100% | 3e-166 | 97.67% | KC757599.1 |
| Trebouxia sp. phot1682 internal transcribed spacer 1, partial sequence; 5.8S ribosomal RNA gene and internal transcribed spacer 2, complete sequence; and 28S ribosomal RNA gene, partial sequence | 597 | 597 | 100% | 3e-166 | 97.67% | KC757598.1 |
| Trebouxia sp. phot1302 internal transcribed spacer 1, partial sequence; 5.8S ribosomal RNA gene and internal transcribed spacer 2, complete sequence; and 28S ribosomal RNA gene, partial sequence | 597 | 597 | 100% | 3e-166 | 97.67% | KC757555.1 |
| Trebouxia sp. phot1301 internal transcribed spacer 1, partial sequence; 5.8S ribosomal RNA gene and internal transcribed spacer 2, complete sequence; and 28S ribosomal RNA gene, partial sequence | 597 | 597 | 100% | 3e-166 | 97.67% | KC757554.1 |
| Trebouxia sp. TAE1 internal transcribed spacer 1, partial sequence; 5.8S ribosomal RNA gene and internal transcribed spacer 2, complete sequence; and 28S ribosomal RNA gene, partial sequence | 597 | 597 | 100% | 3e-166 | 97.67% | FJ792798.1 |
| Uncultured Trebouxia photobiont partial 18S rRNA gene, ITS1, 5.8S rRNA gene, ITS2 and partial 26S rRNA gene, clone L-329-t1 | 597 | 597 | 100% | 3e-166 | 97.67% | AM159203.1 |
| Uncultured Trebouxia photobiont voucher J. Blaha b348 (GZU) internal transcribed spacer 1, partial sequence; and 5.8S ribosomal RNA gene and internal transcribed spacer 2, complete sequence | 597 | 597 | 100% | 3e-166 | 97.67% | DQ166597.1 |

| Description | Max Score | Total Score | Query Cover | E value | Per. Ident | Accession |
|---|---|---|---|---|---|---|
| Trebouxia impressa isolate L1428 internal transcribed spacer 1, partial sequence; 5.8S ribosomal RNA gene and internal transcribed spacer 2, complete sequence; and 28S ribosomal RNA gene, partial sequence | 595 | 595 | 100% | 1e-165 | 97.67% | KJ754210.1 |
| Trebouxia sp. Hap02 internal transcribed spacer 1, partial sequence; 5.8S ribosomal RNA gene and internal transcribed spacer 2, complete sequence; and large subunit ribosomal RNA gene, partial sequence | 592 | 592 | 100% | 2e-164 | 97.38% | MN832486.1 |
| Trebouxia impressa isolate L2252p internal transcribed spacer 1, partial sequence; 5.8S ribosomal RNA gene, complete sequence; and internal transcribed spacer 2, partial sequence | 592 | 592 | 100% | 2e-164 | 97.38% | KX181283.1 |
| Trebouxia impressa isolate L2251p internal transcribed spacer 1, partial sequence; 5.8S ribosomal RNA gene, complete sequence; and internal transcribed spacer 2, partial sequence | 592 | 592 | 100% | 2e-164 | 97.38% | KX181282.1 |
| Trebouxia impressa isolate L2249p internal transcribed spacer 1, partial sequence; 5.8S ribosomal RNA gene, complete sequence; and internal transcribed spacer 2, partial sequence | 592 | 592 | 100% | 2e-164 | 97.38% | KX181281.1 |
| Trebouxia impressa isolate L2248p internal transcribed spacer 1, partial sequence; 5.8S ribosomal RNA gene, complete sequence; and internal transcribed spacer 2, partial sequence | 592 | 592 | 100% | 2e-164 | 97.38% | KX181280.1 |
| Trebouxia impressa isolate L2246p internal transcribed spacer 1, partial sequence; 5.8S ribosomal RNA gene, complete sequence; and internal transcribed spacer 2, partial sequence | 592 | 592 | 100% | 2e-164 | 97.38% | KX181278.1 |
| Trebouxia impressa isolate L4194 internal transcribed spacer 1, partial sequence; 5.8S ribosomal RNA gene and internal transcribed spacer 2, complete sequence; and large subunit ribosomal RNA gene, partial sequence | 592 | 592 | 100% | 2e-164 | 97.38% | OM275790.1 |
| Trebouxia impressa isolate L4193 internal transcribed spacer 1, partial sequence; 5.8S ribosomal RNA gene and internal transcribed spacer 2, complete sequence; and large subunit ribosomal RNA gene, partial sequence | 592 | 592 | 100% | 2e-164 | 97.38% | OM275789.1 |
| Trebouxia impressa isolate L4180 internal transcribed spacer 1, partial sequence; 5.8S ribosomal RNA gene and internal transcribed spacer 2, complete sequence; and large subunit ribosomal RNA gene, partial sequence | 592 | 592 | 100% | 2e-164 | 97.38% | OM275785.1 |
| Trebouxia impressa isolate L4178 internal transcribed spacer 1, partial sequence; 5.8S ribosomal RNA gene and internal transcribed spacer 2, complete sequence; and large subunit ribosomal RNA gene, partial sequence | 592 | 592 | 100% | 2e-164 | 97.38% | OM275784.1 |

| Description | Max Score | Total Score | Query Cover | E value | Per. Ident | Accession |
|---|---|---|---|---|---|---|
| Trebouxia impressa isolate L4176 internal transcribed spacer 1, partial sequence; 5.8S ribosomal RNA gene and internal transcribed spacer 2, complete sequence; and large subunit ribosomal RNA gene, partial sequence | 592 | 592 | 100% | 2e-164 | 97.38% | OM275783.1 |
| Trebouxia flava isolate L4172 internal transcribed spacer 1, partial sequence; 5.8S ribosomal RNA gene and internal transcribed spacer 2, complete sequence; and large subunit ribosomal RNA gene, partial sequence | 592 | 592 | 100% | 2e-164 | 97.38% | OM275782.1 |
| Trebouxia flava isolate L4163 internal transcribed spacer 1, partial sequence; 5.8S ribosomal RNA gene and internal transcribed spacer 2, complete sequence; and large subunit ribosomal RNA gene, partial sequence | 592 | 592 | 100% | 2e-164 | 97.38% | OM275780.1 |
| Trebouxia impressa isolate L4156 internal transcribed spacer 1, partial sequence; 5.8S ribosomal RNA gene and internal transcribed spacer 2, complete sequence; and large subunit ribosomal RNA gene, partial sequence | 592 | 592 | 100% | 2e-164 | 97.38% | OM275776.1 |
| Trebouxia impressa isolate L4155 internal transcribed spacer 1, partial sequence; 5.8S ribosomal RNA gene and internal transcribed spacer 2, complete sequence; and large subunit ribosomal RNA gene, partial sequence | 592 | 592 | 100% | 2e-164 | 97.38% | OM275775.1 |
| Trebouxia impressa isolate L4152 internal transcribed spacer 1, partial sequence; 5.8S ribosomal RNA gene and internal transcribed spacer 2, complete sequence; and large subunit ribosomal RNA gene, partial sequence | 592 | 592 | 100% | 2e-164 | 97.38% | OM275772.1 |
| Trebouxia flava isolate L3953 internal transcribed spacer 1, partial sequence; 5.8S ribosomal RNA gene and internal transcribed spacer 2, complete sequence; and large subunit ribosomal RNA gene, partial sequence | 592 | 592 | 100% | 2e-164 | 97.38% | OM275760.1 |
| Trebouxia impressa isolate L3952 internal transcribed spacer 1, partial sequence; 5.8S ribosomal RNA gene and internal transcribed spacer 2, complete sequence; and large subunit ribosomal RNA gene, partial sequence | 592 | 592 | 100% | 2e-164 | 97.38% | OM275759.1 |
| Trebouxia impressa isolate L3888 internal transcribed spacer 1, partial sequence; 5.8S ribosomal RNA gene and internal transcribed spacer 2, complete sequence; and large subunit ribosomal RNA gene, partial sequence | 592 | 592 | 100% | 2e-164 | 97.38% | OM275752.1 |
| Trebouxia impressa isolate L3887 internal transcribed spacer 1, partial sequence; 5.8S ribosomal RNA gene and internal transcribed spacer 2, complete sequence; and large subunit ribosomal RNA gene, partial sequence | 592 | 592 | 100% | 2e-164 | 97.38% | OM275751.1 |

| Description | Max Score | Total Score | Query Cover | E value | Per. Ident | Accession |
|---|---|---|---|---|---|---|
| Trebouxia impressa isolate L3724 internal transcribed spacer 1, partial sequence; 5.8S ribosomal RNA gene and internal transcribed spacer 2, complete sequence; and large subunit ribosomal RNA gene, partial sequence | 592 | 592 | 100% | 2e-164 | 97.38% | OM275747.1 |
| Trebouxia impressa isolate L3692 internal transcribed spacer 1, partial sequence; 5.8S ribosomal RNA gene and internal transcribed spacer 2, complete sequence; and large subunit ribosomal RNA gene, partial sequence | 592 | 592 | 100% | 2e-164 | 97.38% | OM275742.1 |
| Trebouxia impressa isolate L3681 internal transcribed spacer 1, partial sequence; 5.8S ribosomal RNA gene and internal transcribed spacer 2, complete sequence; and large subunit ribosomal RNA gene, partial sequence | 592 | 592 | 100% | 2e-164 | 97.38% | OM275741.1 |
| Trebouxia impressa isolate L3653 internal transcribed spacer 1, partial sequence; 5.8S ribosomal RNA gene and internal transcribed spacer 2, complete sequence; and large subunit ribosomal RNA gene, partial sequence | 592 | 592 | 100% | 2e-164 | 97.38% | OM275740.1 |
| Trebouxia impressa isolate L3577 internal transcribed spacer 1, partial sequence; 5.8S ribosomal RNA gene and internal transcribed spacer 2, complete sequence; and large subunit ribosomal RNA gene, partial sequence | 592 | 592 | 100% | 2e-164 | 97.38% | OM275737.1 |
| Trebouxia impressa isolate L3576 internal transcribed spacer 1, partial sequence; 5.8S ribosomal RNA gene and internal transcribed spacer 2, complete sequence; and large subunit ribosomal RNA gene, partial sequence | 592 | 592 | 100% | 2e-164 | 97.38% | OM275736.1 |
| Trebouxia flava isolate L3572 internal transcribed spacer 1, partial sequence; 5.8S ribosomal RNA gene and internal transcribed spacer 2, complete sequence; and large subunit ribosomal RNA gene, partial sequence | 592 | 592 | 100% | 2e-164 | 97.38% | OM275735.1 |
| Trebouxia impressa isolate L3569 internal transcribed spacer 1, partial sequence; 5.8S ribosomal RNA gene and internal transcribed spacer 2, complete sequence; and large subunit ribosomal RNA gene, partial sequence | 592 | 592 | 100% | 2e-164 | 97.38% | OM275734.1 |
| Trebouxia flava isolate L3564 internal transcribed spacer 1, partial sequence; 5.8S ribosomal RNA gene and internal transcribed spacer 2, complete sequence; and large subunit ribosomal RNA gene, partial sequence | 592 | 592 | 100% | 2e-164 | 97.38% | OM275733.1 |
| Trebouxia impressa isolate L3559 internal transcribed spacer 1, partial sequence; 5.8S ribosomal RNA gene and internal transcribed spacer 2, complete sequence; and large subunit ribosomal RNA gene, partial sequence | 592 | 592 | 100% | 2e-164 | 97.38% | OM275732.1 |

| Description | Max Score | Total Score | Query Cover | E value | Per. Ident | Accession |
|---|---|---|---|---|---|---|
| Trebouxia flava isolate L3523 internal transcribed spacer 1, partial sequence; 5.8S ribosomal RNA gene and internal transcribed spacer 2, complete sequence; and large subunit ribosomal RNA gene, partial sequence | 592 | 592 | 100% | 2e-164 | 97.38% | OM275728.1 |
| Trebouxia impressa isolate L3497 internal transcribed spacer 1, partial sequence; 5.8S ribosomal RNA gene and internal transcribed spacer 2, complete sequence; and large subunit ribosomal RNA gene, partial sequence | 592 | 592 | 100% | 2e-164 | 97.38% | OM275727.1 |
| Trebouxia impressa isolate L3440 internal transcribed spacer 1, partial sequence; 5.8S ribosomal RNA gene and internal transcribed spacer 2, complete sequence; and large subunit ribosomal RNA gene, partial sequence | 592 | 592 | 100% | 2e-164 | 97.38% | OM275719.1 |
| Trebouxia impressa isolate L3365 internal transcribed spacer 1, partial sequence; 5.8S ribosomal RNA gene and internal transcribed spacer 2, complete sequence; and large subunit ribosomal RNA gene, partial sequence | 592 | 592 | 100% | 2e-164 | 97.38% | OM275711.1 |
| Trebouxia flava isolate L3314 internal transcribed spacer 1, partial sequence; 5.8S ribosomal RNA gene and internal transcribed spacer 2, complete sequence; and large subunit ribosomal RNA gene, partial sequence | 592 | 592 | 100% | 2e-164 | 97.38% | OM275704.1 |
| Trebouxia impressa isolate L3183 internal transcribed spacer 1, partial sequence; 5.8S ribosomal RNA gene and internal transcribed spacer 2, complete sequence; and large subunit ribosomal RNA gene, partial sequence | 592 | 592 | 100% | 2e-164 | 97.38% | OM275654.1 |
| Trebouxia impressa isolate L3182 internal transcribed spacer 1, partial sequence; 5.8S ribosomal RNA gene and internal transcribed spacer 2, complete sequence; and large subunit ribosomal RNA gene, partial sequence | 592 | 592 | 100% | 2e-164 | 97.38% | OM275653.1 |
| Trebouxia flava isolate L3171 internal transcribed spacer 1, partial sequence; 5.8S ribosomal RNA gene and internal transcribed spacer 2, complete sequence; and large subunit ribosomal RNA gene, partial sequence | 592 | 592 | 100% | 2e-164 | 97.38% | OM275644.1 |
| Trebouxia flava isolate L3167 internal transcribed spacer 1, partial sequence; 5.8S ribosomal RNA gene and internal transcribed spacer 2, complete sequence; and large subunit ribosomal RNA gene, partial sequence | 592 | 592 | 100% | 2e-164 | 97.38% | OM275641.1 |
| Trebouxia impressa isolate L2962 internal transcribed spacer 1, partial sequence; 5.8S ribosomal RNA gene and internal transcribed spacer 2, complete sequence; and large subunit ribosomal RNA gene, partial sequence | 592 | 592 | 100% | 2e-164 | 97.38% | OM275584.1 |

| Description | Max Score | Total Score | Query Cover | E value | Per. Ident | Accession |
|---|---|---|---|---|---|---|
| Trebouxia impressa isolate L2961 internal transcribed spacer 1, partial sequence; 5.8S ribosomal RNA gene and internal transcribed spacer 2, complete sequence; and large subunit ribosomal RNA gene, partial sequence | 592 | 592 | 100% | 2e-164 | 97.38% | OM275583.1 |
| Trebouxia flava isolate L2960 internal transcribed spacer 1, partial sequence; 5.8S ribosomal RNA gene and internal transcribed spacer 2, complete sequence; and large subunit ribosomal RNA gene, partial sequence | 592 | 592 | 100% | 2e-164 | 97.38% | OM275582.1 |
| Trebouxia flava isolate L2955 internal transcribed spacer 1, partial sequence; 5.8S ribosomal RNA gene and internal transcribed spacer 2, complete sequence; and large subunit ribosomal RNA gene, partial sequence | 592 | 592 | 100% | 2e-164 | 97.38% | OM275578.1 |
| Trebouxia flava isolate L2954 internal transcribed spacer 1, partial sequence; 5.8S ribosomal RNA gene and internal transcribed spacer 2, complete sequence; and large subunit ribosomal RNA gene, partial sequence | 592 | 592 | 100% | 2e-164 | 97.38% | OM275577.1 |
| Trebouxia flava isolate L2952 internal transcribed spacer 1, partial sequence; 5.8S ribosomal RNA gene and internal transcribed spacer 2, complete sequence; and large subunit ribosomal RNA gene, partial sequence | 592 | 592 | 100% | 2e-164 | 97.38% | OM275575.1 |
| Trebouxia flava isolate L2723 internal transcribed spacer 1, partial sequence; 5.8S ribosomal RNA gene and internal transcribed spacer 2, complete sequence; and large subunit ribosomal RNA gene, partial sequence | 592 | 592 | 100% | 2e-164 | 97.38% | OM275514.1 |
| Trebouxia impressa isolate L2635 internal transcribed spacer 1, partial sequence; 5.8S ribosomal RNA gene and internal transcribed spacer 2, complete sequence; and large subunit ribosomal RNA gene, partial sequence | 592 | 592 | 100% | 2e-164 | 97.38% | OM275507.1 |
| Trebouxia impressa isolate 823-1-P/L internal transcribed spacer 1, partial sequence; 5.8S ribosomal RNA gene and internal transcribed spacer 2, complete sequence; and large subunit ribosomal RNA gene, partial sequence | 592 | 592 | 100% | 2e-164 | 97.38% | MZ919275.1 |
| Trebouxia impressa isolate L2920 internal transcribed spacer 1, partial sequence; 5.8S ribosomal RNA gene and internal transcribed spacer 2, complete sequence; and large subunit ribosomal RNA gene, partial sequence | 588 | 588 | 100% | 2e-163 | 97.08% | OM275546.1 |

## Alignments

Equisetum variegatum voucher ZA2012-2553 internal transcribed spacer 2, partial sequence

Sequence ID: **KT960212.1** Length: 343 Number of Matches: 1
Range 1: 1 to 343

| Score | Expect | Identities | Gaps | Strand | Frame |
|---|---|---|---|---|---|
| 619 bits(335) | 7e-173() | 343/343(100%) | 0/343(0%) | Plus/Plus | |

Features:

```
Query  1    TGCAGAATTCCGTGAACCATCGAATCTTTGAACGCAAATTGCNNTTGAGGCTTCGGCCAA  60
            ||||||||||||||||||||||||||||||||||||||||||||||||||||||||||||
Sbjct  1    TGCAGAATTCCGTGAACCATCGAATCTTTGAACGCAAATTGCNNTTGAGGCTTCGGCCAA  60

Query  61   GAGCATGTCTGGCTCAGCGTCGGCTTACTACCCTCAACCAATTCCCATGTGGAAATTGAT  120
            ||||||||||||||||||||||||||||||||||||||||||||||||||||||||||||
Sbjct  61   GAGCATGTCTGGCTCAGCGTCGGCTTACTACCCTCAACCAATTCCCATGTGGAAATTGAT  120

Query  121  TGGAGCTGGCTTTCCGGTTTTCCGACTAGGTTGCCGGTCAGCTGAAAAACATTCGCGCTG  180
            ||||||||||||||||||||||||||||||||||||||||||||||||||||||||||||
Sbjct  121  TGGAGCTGGCTTTCCGGTTTTCCGACTAGGTTGCCGGTCAGCTGAAAAACATTCGCGCTG  180

Query  181  TGGGAGCTGCTTTCCCTTCAAAAAGGAAAGCCGAGTTCCGATTTGGTAGGTGACCTTTGC  240
            ||||||||||||||||||||||||||||||||||||||||||||||||||||||||||||
Sbjct  181  TGGGAGCTGCTTTCCCTTCAAAAAGGAAAGCCGAGTTCCGATTTGGTAGGTGACCTTTGC  240

Query  241  CGGTTGCATGCCTTCGTTGCTCGCAATGGCTTCCATGAAAGCGTGTGCAGGACGCCNTTN  300
            ||||||||||||||||||||||||||||||||||||||||||||||||||||||||||||
Sbjct  241  CGGTTGCATGCCTTCGTTGCTCGCAATGGCTTCCATGAAAGCGTGTGCAGGACGCCNTTN  300

Query  301  GGCTTTCGACCTGAGCTCAGGCAAGAACACCCGCTGAACTTAA  343
            |||||||||||||||||||||||||||||||||||||||||||
Sbjct  301  GGCTTTCGACCTGAGCTCAGGCAAGAACACCCGCTGAACTTAA  343
```

Trebouxia sp. Hap09 internal transcribed spacer 1, partial sequence; 5.8S ribosomal RNA gene and internal transcribed spacer 2, complete sequence; and large subunit ribosomal RNA gene, partial sequence

Sequence ID: **MN832493.1** Length: 697 Number of Matches: 1

Range 1: 300 to 642

| Score | Expect | Identities | Gaps | Strand | Frame |
|---|---|---|---|---|---|
| 608 bits(329) | 2e-169() | 337/343(98%) | 0/343(0%) | Plus/Plus | |

Features:

```
Query  1    TGCAGAATTCCGTGAACCATCGAATCTTTGAACGCAAATTGCNNTTGAGGCTTCGGCCAA  60
            ||||||||||||||||||||||||||||||||||||||||||  ||||||||||||||||
Sbjct  300  TGCAGAATTCCGTGAACCATCGAATCTTTGAACGCAAATTGCGCTTGAGGCTTCGGCCAA  359

Query  61   GAGCATGTCTGGCTCAGCGTCGGCTTACTACCCTCAACCAATTCCCATGTGGAAATTGAT  120
            |||||||||||||||||||||||||||||||||||||||||||||| |||||||||||||
Sbjct  360  GAGCATGTCTGGCTCAGCGTCGGCTTACTACCCTCAACCAATTCCCTTGTGGAAATTGAT  419

Query  121  TGGAGCTGGCTTTCCGGTTTTCCGACTAGGTTGCCGGTCAGCTGAAAAACATTCGCGCTG  180
            ||||||||||||||||||||||||||||||||||||||||||||||||| ||||||||||
Sbjct  420  TGGAGCTGGCTTTCCGGTTTTCCGACTAGGTTGCCGGTCAGCTGAAAAATATTCGCGCTG  479

Query  181  TGGGAGCTGCTTTCCCTTCAAAAAGGAAAGCCGAGTTCCGATTTGGTAGGTGACCTTTGC  240
            ||||||||||||||||||||||||||||||||||||||||||||||||||||||||||||
Sbjct  480  TGGGAGCTGCTTTCCCTTCAAAAAGGAAAGCCGAGTTCCGATTTGGTAGGTGACCTTTGC  539

Query  241  CGGTTGCATGCCTTCGTTGCTCGCAATGGCTTCCATGAAAGCGTGTGCAGGACGCCNTTN  300
            |||||||||||||||||||||||||||||||||||||||||||||||||||||||| || 
Sbjct  540  CGGTTGCATGCCTTCGTTGCTCGCAATGGCTTCCATGAAAGCGTGTGCAGGACGCCTTTT  599

Query  301  GGCTTTCGACCTGAGCTCAGGCAAGAACACCCGCTGAACTTAA  343
            |||||||||||||||||||||||||||||||||||||||||||
Sbjct  600  GGCTTTCGACCTGAGCTCAGGCAAGAACACCCGCTGAACTTAA  642
```

Trebouxia sp. Hap07 internal transcribed spacer 1, partial sequence; 5.8S ribosomal RNA gene and internal transcribed spacer 2, complete sequence; and large subunit ribosomal RNA gene, partial sequence

Sequence ID: **MN832491.1** Length: 697 Number of Matches: 1

Range 1: 300 to 642

| Score | Expect | Identities | Gaps | Strand | Frame |
|---|---|---|---|---|---|
| 608 bits(329) | 2e-169() | 337/343(98%) | 0/343(0%) | Plus/Plus | |

Features:

```
Query  1    TGCAGAATTCCGTGAACCATCGAATCTTTGAACGCAAATTGCNNTTGAGGCTTCGGCCAA  60
            ||||||||||||||||||||||||||||||||||||||||||  ||||||||||||||||
Sbjct  300  TGCAGAATTCCGTGAACCATCGAATCTTTGAACGCAAATTGCGCTTGAGGCTTCGGCCAA  359
```

```
Query  61    GAGCATGTCTGGCTCAGCGTCGGCTTACTACCCTCAACCAATTCCCATGTGGAAATTGAT  120
             |||||||||||||||||||||||||||||||||||||||||||||| |||||||||||||
Sbjct  360   GAGCATGTCTGGCTCAGCGTCGGCTTACTACCCTCAACCAATTCCCTTGTGGAAATTGAT  419

Query  121   TGGAGCTGGCTTTCCGGTTTTCCGACTAGGTTGCCGGTCAGCTGAAAAACATTCGCGCTG  180
             ||||||||||||||||||||||||||||||||||||||||||||||||| ||||||||||
Sbjct  420   TGGAGCTGGCTTTCCGGTTTTCCGACTAGGTTGCCGGTCAGCTGAAAAATATTCGCGCTG  479

Query  181   TGGGAGCTGCTTTCCCTTCAAAAAGGAAAGCCGAGTTCCGATTTGGTAGGTGACCTTTGC  240
             ||||||||||||||||||||||||||||||||||||||||||||||||||||||||||||
Sbjct  480   TGGGAGCTGCTTTCCCTTCAAAAAGGAAAGCCGAGTTCCGATTTGGTAGGTGACCTTTGC  539

Query  241   CGGTTGCATGCCTTCGTTGCTCGCAATGGCTTCCATGAAAGCGTGTGCAGGACGCCNTTN  300
             |||||||||||||||||||||||||||||||||||||||||||||||||||||||| || 
Sbjct  540   CGGTTGCATGCCTTCGTTGCTCGCAATGGCTTCCATGAAAGCGTGTGCAGGACGCCTTTT  599

Query  301   GGCTTTCGACCTGAGCTCAGGCAAGAACACCCGCTGAACTTAA  343
             |||||||||||||||||||||||||||||||||||||||||||
Sbjct  600   GGCTTTCGACCTGAGCTCAGGCAAGAACACCCGCTGAACTTAA  642
```

Trebouxia sp. Hap06 internal transcribed spacer 1, partial sequence; 5.8S ribosomal RNA gene and internal transcribed spacer 2, complete sequence; and large subunit ribosomal RNA gene, partial sequence

Sequence ID: **MN832490.1** Length: 697 Number of Matches: 1

Range 1: 300 to 642

| Score | Expect | Identities | Gaps | Strand | Frame |
|---|---|---|---|---|---|
| 608 bits(329) | 2e-169() | 337/343(98%) | 0/343(0%) | Plus/Plus | |

Features:

```
Query  1     TGCAGAATTCCGTGAACCATCGAATCTTTGAACGCAAATTGCNNTTGAGGCTTCGGCCAA  60
             ||||||||||||||||||||||||||||||||||||||||||  ||||||||||||||||
Sbjct  300   TGCAGAATTCCGTGAACCATCGAATCTTTGAACGCAAATTGCGCTTGAGGCTTCGGCCAA  359

Query  61    GAGCATGTCTGGCTCAGCGTCGGCTTACTACCCTCAACCAATTCCCATGTGGAAATTGAT  120
             |||||||||||||||||||||||||||||||||||||||||||||| |||||||||||||
Sbjct  360   GAGCATGTCTGGCTCAGCGTCGGCTTACTACCCTCAACCAATTCCCTTGTGGAAATTGAT  419

Query  121   TGGAGCTGGCTTTCCGGTTTTCCGACTAGGTTGCCGGTCAGCTGAAAAACATTCGCGCTG  180
             ||||||||||||||||||||||||||||||||||||||||||||||||| ||||||||||
Sbjct  420   TGGAGCTGGCTTTCCGGTTTTCCGACTAGGTTGCCGGTCAGCTGAAAAATATTCGCGCTG  479

Query  181   TGGGAGCTGCTTTCCCTTCAAAAAGGAAAGCCGAGTTCCGATTTGGTAGGTGACCTTTGC  240
             ||||||||||||||||||||||||||||||||||||||||||||||||||||||||||||
Sbjct  480   TGGGAGCTGCTTTCCCTTCAAAAAGGAAAGCCGAGTTCCGATTTGGTAGGTGACCTTTGC  539

Query  241   CGGTTGCATGCCTTCGTTGCTCGCAATGGCTTCCATGAAAGCGTGTGCAGGACGCCNTTN  300
             |||||||||||||||||||||||||||||||||||||||||||||||||||||||| || 
Sbjct  540   CGGTTGCATGCCTTCGTTGCTCGCAATGGCTTCCATGAAAGCGTGTGCAGGACGCCTTTT  599

Query  301   GGCTTTCGACCTGAGCTCAGGCAAGAACACCCGCTGAACTTAA  343
             |||||||||||||||||||||||||||||||||||||||||||
Sbjct  600   GGCTTTCGACCTGAGCTCAGGCAAGAACACCCGCTGAACTTAA  642
```

Trebouxia impressa isolate L2239p internal transcribed spacer 1, partial sequence; 5.8S ribosomal RNA gene, complete sequence; and internal transcribed spacer 2, partial sequence

Sequence ID: **KX181276.1** Length: 692 Number of Matches: 1

Range 1: 289 to 631

| Score | Expect | Identities | Gaps | Strand | Frame |
|---|---|---|---|---|---|
| 608 bits(329) | 2e-169() | 337/343(98%) | 0/343(0%) | Plus/Plus | |

Features:

```
Query  1     TGCAGAATTCCGTGAACCATCGAATCTTTGAACGCAAATTGCNNTTGAGGCTTCGGCCAA  60
             ||||||||||||||||||||||||||||||||||||||||||  ||||||||||||||||
Sbjct  289   TGCAGAATTCCGTGAACCATCGAATCTTTGAACGCAAATTGCGCTTGAGGCTTCGGCCAA  348

Query  61    GAGCATGTCTGGCTCAGCGTCGGCTTACTACCCTCAACCAATTCCCATGTGGAAATTGAT  120
             |||||||||||||||||||||||||||||||||||||||||||||| |||||||||||||
Sbjct  349   GAGCATGTCTGGCTCAGCGTCGGCTTACTACCCTCAACCAATTCCCTTGTGGAAATTGAT  408

Query  121   TGGAGCTGGCTTTCCGGTTTTCCGACTAGGTTGCCGGTCAGCTGAAAAACATTCGCGCTG  180
             ||||||||||||||||||||||||||||||||||||||||||||||||| ||||||||||
Sbjct  409   TGGAGCTGGCTTTCCGGTTTTCCGACTAGGTTGCCGGTCAGCTGAAAAATATTCGCGCTG  468

Query  181   TGGGAGCTGCTTTCCCTTCAAAAAGGAAAGCCGAGTTCCGATTTGGTAGGTGACCTTTGC  240
             ||||||||||||||||||||||||||||||||||||||||||||||||||||||||||||
Sbjct  469   TGGGAGCTGCTTTCCCTTCAAAAAGGAAAGCCGAGTTCCGATTTGGTAGGTGACCTTTGC  528
```

```
Query  241   CGGTTGCATGCCTTCGTTGCTCGCAATGGCTTCCATGAAAGCGTGTGCAGGACGCCNTTN  300
             |||||||||||||||||||||||||||||||||||||||||||||||||||||||| ||
Sbjct  529   CGGTTGCATGCCTTCGTTGCTCGCAATGGCTTCCATGAAAGCGTGTGCAGGACGCCTTTT  588

Query  301   GGCTTTCGACCTGAGCTCAGGCAAGAACACCCGCTGAACTTAA  343
             |||||||||||||||||||||||||||||||||||||||||||
Sbjct  589   GGCTTTCGACCTGAGCTCAGGCAAGAACACCCGCTGAACTTAA  631
```

BLAST is a registered trademark of the National Library of Medicine
