# Supplementary material for: A Multi-Pronged Technique for Identifying Equisetum palustre and Equisetum arvense—Combining HPTLC, HPLC-ESI-MS/MS and Optimized DNA Barcoding Techniques
Source: Plants (Basel). 2022 Sep 28;11(19):2562. doi: 10.3390/plants11192562 (PMC9573248; doi:10.3390/plants11192562)
Supplement: Supplementary file 1 [file plants-11-02562-s001.zip › plants-1932149 supplementary/Horsetail Supplementary Figures/Figure S15.pdf]

BLAST Results

Questions/comments

Job title: EU328339.1

**RID**	FD4UJ9FS013 (Expires on 08-14 01:52 am)
**Query ID**	lcl|Query_16675
**Description**	None
**Molecule type**	dna
**Query Length**	305
**Database Name**	nt
**Description**	Nucleotide collection (nt)
**Program**	BLASTN 2.13.0+

Graphic Summary

Distribution of the top 101 Blast Hits on 100 subject sequences

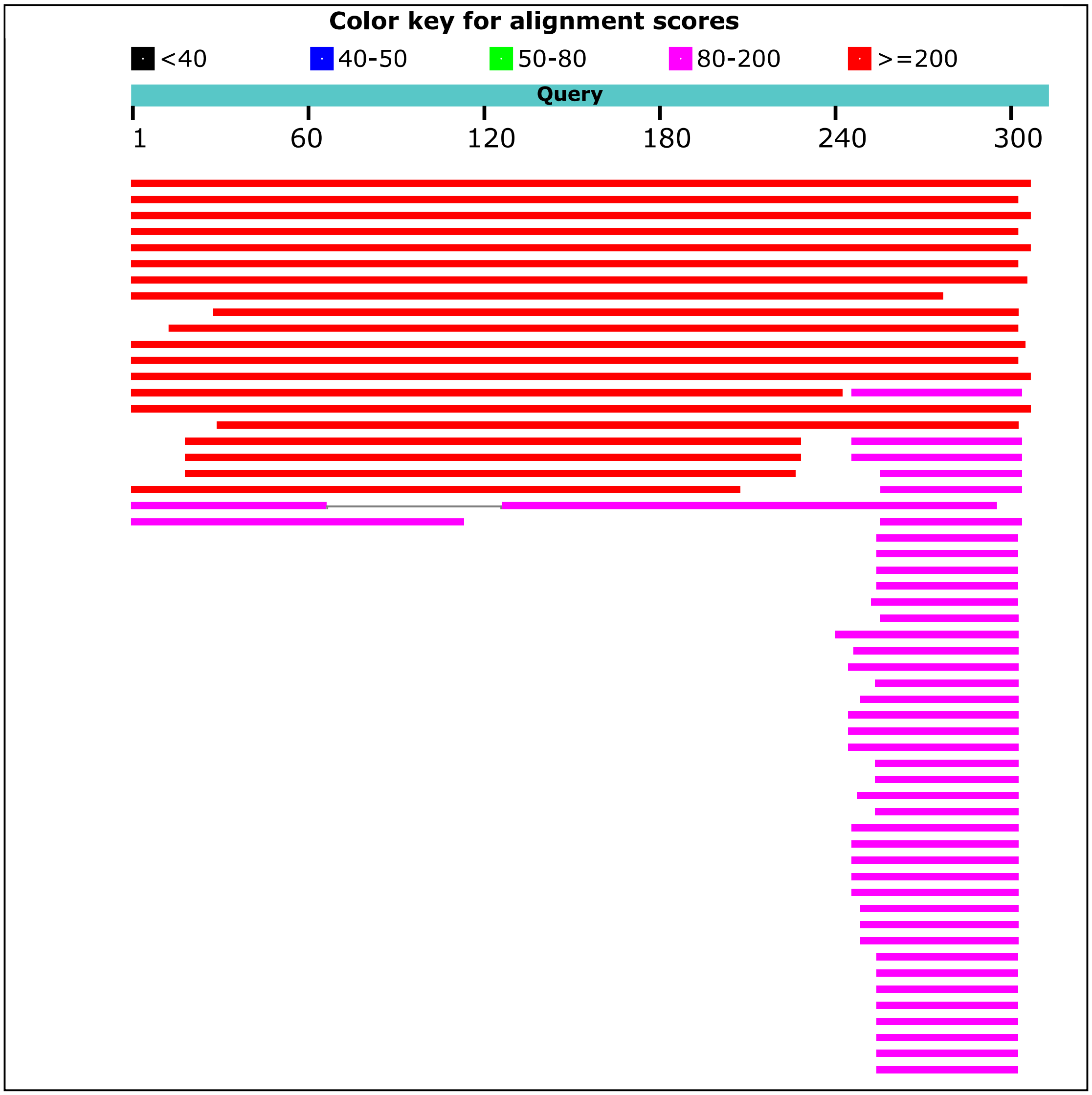

Descriptions

Sequences producing significant alignments:

Description	Max Score	Total Score	Query Cover	E value	Per. Ident	Accession
Equisetum hyemale subsp. affine 18S ribosomal RNA gene, partial sequence; internal transcribed spacer 1, complete sequence; and 5.8S ribosomal RNA gene, partial sequence	549	549	100%	8e-152	100.00%	EU328339.1
Equisetum x ferrissii 18S ribosomal RNA gene, partial sequence; internal transcribed spacer 1, complete sequence; and 5.8S ribosomal RNA gene, partial sequence	510	510	98%	4e-140	96.35%	EU328338.1
Equisetum variegatum voucher B. Hoshizaki 592 18S ribosomal RNA gene, partial sequence; internal transcribed spacer 1, complete sequence; and 5.8S ribosomal RNA gene, partial sequence	483	483	100%	9e-132	94.43%	EU372664.1
Equisetum variegatum 18S ribosomal RNA gene, partial sequence; internal transcribed spacer 1, complete sequence; and 5.8S ribosomal RNA gene, partial sequence	477	477	98%	4e-130	94.68%	EU328340.1
Equisetum hyemale subsp. affine 18S ribosomal RNA gene, partial sequence; internal transcribed spacer 1, complete sequence; and 5.8S ribosomal RNA gene, partial sequence	475	475	100%	1e-129	94.10%	EU328336.1
Equisetum ramosissimum isolate BPTPS123 small subunit ribosomal RNA gene, partial sequence; internal transcribed spacer 1, 5.8S ribosomal RNA gene, and internal transcribed spacer 2, complete sequence; and large subunit ribosomal RNA gene, partial sequence	459	459	98%	1e-124	93.69%	ON685433.1
Equisetum laevigatum 18S ribosomal RNA gene, partial sequence; internal transcribed spacer 1, complete sequence; and 5.8S ribosomal RNA gene, partial sequence	449	449	99%	9e-122	92.43%	EU328341.1
Equisetum variegatum subsp. variegatum internal transcribed spacer 1, partial sequence	444	444	90%	4e-120	95.27%	DQ377155.1
Equisetum hyemale genes for ITS1, 5.8S rRNA, ITS2, partial sequence, bio_material: KB:NIBR0000051239	435	435	89%	2e-117	94.87%	AB817880.1

Description	Max Score	Total Score	Query Cover	E value	Per. Ident	Accession
Equisetum ramosissimum 18S ribosomal RNA gene, partial sequence; internal transcribed spacer 1, 5.8S ribosomal RNA gene and internal transcribed spacer 2, complete sequence; and 26S ribosomal RNA gene, partial sequence	435	435	94%	2e-117	93.40%	AF448794.1
Equisetum myriochaetum 18S ribosomal RNA gene, partial sequence; internal transcribed spacer 1, complete sequence; and 5.8S ribosomal RNA gene, partial sequence	433	433	99%	9e-117	92.08%	EU328337.1
Equisetum ramosissimum isolate BPTPS056 small subunit ribosomal RNA gene, partial sequence; internal transcribed spacer 1, 5.8S ribosomal RNA gene, and internal transcribed spacer 2, complete sequence; and large subunit ribosomal RNA gene, partial sequence	431	431	98%	3e-116	92.05%	OM670239.1
Equisetum variegatum voucher B. Hoshizaki 203 18S ribosomal RNA gene, partial sequence; internal transcribed spacer 1, complete sequence; and 5.8S ribosomal RNA gene, partial sequence	429	429	100%	1e-115	91.15%	EU372665.1
Equisetum hyemale subsp. hyemale internal transcribed spacer 1, partial sequence	390	390	79%	5e-104	95.44%	DQ377151.1
Equisetum scirpoides voucher P. Hammond s.n 18S ribosomal RNA gene, partial sequence; internal transcribed spacer 1, complete sequence; and 5.8S ribosomal RNA gene, partial sequence	387	387	100%	7e-103	87.87%	EU372663.1
Equisetum environmental sample clone EDNA16-0043490 internal transcribed spacer 1, partial sequence; 5.8S ribosomal RNA gene and internal transcribed spacer 2, complete sequence; and large subunit ribosomal RNA gene, partial sequence	322	322	89%	2e-83	87.55%	MT198943.1
Equisetum x meridionale internal transcribed spacer 1, partial sequence	303	303	68%	7e-78	92.42%	DQ377156.1
Equisetum x moorei internal transcribed spacer 1, partial sequence	296	296	68%	1e-75	91.87%	DQ377157.1
Equisetum ramosissimum subsp. ramosissimum internal transcribed spacer 1, partial sequence	285	285	67%	3e-72	91.30%	DQ377153.1

Description	Max Score	Total Score	Query Cover	E value	Per. Ident	Accession
Equisetum ramosissimum 18S ribosomal RNA gene, partial sequence; internal transcribed spacer 1, complete sequence; and 5.8S ribosomal RNA gene, partial sequence	274	274	67%	6e-69	89.81%	EU328343.1
Equisetum sylvaticum 18S ribosomal RNA gene, partial sequence; internal transcribed spacer 1, complete sequence; and 5.8S ribosomal RNA gene, partial sequence	185	295	76%	3e-42	85.71%	EU328342.1
Equisetum telmateia isolate DM233 small subunit ribosomal RNA gene, internal transcribed spacer 1, 5.8S ribosomal RNA gene, internal transcribed spacer 2, and large subunit ribosomal RNA gene, region	158	158	36%	6e-34	91.96%	MT784095.1
Durio graveolens isolate S internal transcribed spacer 1, partial sequence; 5.8S ribosomal RNA gene, complete sequence; and internal transcribed spacer 2, partial sequence	87.9	87.9	18%	8e-13	94.74%	MF629768.1
Durio zibethinus cultivar Kinoso internal transcribed spacer 1, partial sequence; 5.8S ribosomal RNA gene, complete sequence; and internal transcribed spacer 2, partial sequence	87.9	87.9	18%	8e-13	94.74%	ON817274.1
Durio zibethinus cultivar Duriat internal transcribed spacer 1, partial sequence; 5.8S ribosomal RNA gene, complete sequence; and internal transcribed spacer 2, partial sequence	87.9	87.9	18%	8e-13	94.74%	ON815633.1
Monotropa uniflora isolate Freudenstein 2874 internal transcribed spacer 1, partial sequence; 5.8S ribosomal RNA gene and internal transcribed spacer 2, complete sequence; and large subunit ribosomal RNA gene, partial sequence	87.9	87.9	15%	8e-13	100.00%	MZ063657.1
Monotropa uniflora isolate Freudenstein 3054 small subunit ribosomal RNA gene, partial sequence; internal transcribed spacer 1, 5.8S ribosomal RNA gene, and internal transcribed spacer 2, complete sequence; and large subunit ribosomal RNA gene, partial sequence	87.9	87.9	15%	8e-13	100.00%	MZ063635.1

Description	Max Score	Total Score	Query Cover	E value	Per. Ident	Accession
Monotropa brittonii isolate Keesling 67 small subunit ribosomal RNA gene, partial sequence; internal transcribed spacer 1, 5.8S ribosomal RNA gene, and internal transcribed spacer 2, complete sequence; and large subunit ribosomal RNA gene, partial sequence	87.9	87.9	15%	8e-13	100.00%	MZ063621.1
Monotropa uniflora isolate Keesling 58 small subunit ribosomal RNA gene, partial sequence; internal transcribed spacer 1, 5.8S ribosomal RNA gene, and internal transcribed spacer 2, complete sequence; and large subunit ribosomal RNA gene, partial sequence	87.9	87.9	15%	8e-13	100.00%	MZ063612.1
Monotropa uniflora isolate Keesling 57 small subunit ribosomal RNA gene, partial sequence; internal transcribed spacer 1, 5.8S ribosomal RNA gene, and internal transcribed spacer 2, complete sequence; and large subunit ribosomal RNA gene, partial sequence	87.9	87.9	15%	8e-13	100.00%	MZ063611.1
Monotropa uniflora isolate Keesling 52 small subunit ribosomal RNA gene, partial sequence; internal transcribed spacer 1, 5.8S ribosomal RNA gene, and internal transcribed spacer 2, complete sequence; and large subunit ribosomal RNA gene, partial sequence	87.9	87.9	15%	8e-13	100.00%	MZ063607.1
Monotropastrum humile genes for ITS1, 5.8S rRNA, ITS2, isolate: YM-2007-Mie11	87.9	87.9	15%	8e-13	100.00%	AB594725.1
Lespedeza thunbergii subsp. formosa isolate BX16ITS-194 18S ribosomal RNA gene, partial sequence; internal transcribed spacer 1, 5.8S ribosomal RNA gene, and internal transcribed spacer 2, complete sequence; and 26S ribosomal RNA gene, partial sequence	86.1	86.1	16%	3e-12	97.96%	KY174637.1
Asarum kiusianum as36 genes for ITS1, 5.8S rRNA, ITS2, partial and complete sequence	86.1	86.1	15%	3e-12	100.00%	LC669867.1
Camellia sinensis f. formosensis clone Taq_4 internal transcribed spacer 1, partial sequence; 5.8S ribosomal RNA gene, complete sequence; and internal transcribed spacer 2, partial sequence	86.1	86.1	20%	3e-12	91.80%	EF544714.1

Description	Max Score	Total Score	Query Cover	E value	Per. Ident	Accession
Cleistanthus tomentosus voucher Y. Li lyq92 (IBSC, M) 18S ribosomal RNA gene, partial sequence; internal transcribed spacer 1, 5.8S ribosomal RNA gene, and internal transcribed spacer 2, complete sequence; and 26S ribosomal RNA gene, partial sequence	86.1	86.1	18%	3e-12	94.55%	FJ439932.1
Monotropa uniflora isolate Freudenstein 3062 small subunit ribosomal RNA gene, partial sequence; internal transcribed spacer 1, 5.8S ribosomal RNA gene, and internal transcribed spacer 2, complete sequence; and large subunit ribosomal RNA gene, partial sequence	84.2	84.2	18%	1e-11	92.98%	MZ063636.1
Croninia kingiana isolate VA02 18S ribosomal RNA gene, partial sequence; internal transcribed spacer 1, 5.8S ribosomal RNA gene, and internal transcribed spacer 2, complete sequence; and 28S ribosomal RNA gene, partial sequence	84.2	84.2	15%	1e-11	97.92%	KC197140.1
Acidoton microphyllus isolate WCM_317 18S ribosomal RNA gene, partial sequence; internal transcribed spacer 1, 5.8S ribosomal RNA gene, and internal transcribed spacer 2, complete sequence; and 26S ribosomal RNA gene, partial sequence	84.2	84.2	17%	1e-11	94.34%	MK780907.1
Campanula barbata voucher PAL 96204 small subunit ribosomal RNA gene, partial sequence; internal transcribed spacer 1, 5.8S ribosomal RNA gene, and internal transcribed spacer 2, complete sequence; and large subunit ribosomal RNA gene, partial sequence	84.2	84.2	18%	1e-11	92.98%	MT774696.1
Campanula tomentosa voucher EGE 43418 small subunit ribosomal RNA gene, partial sequence; internal transcribed spacer 1, 5.8S ribosomal RNA gene, and internal transcribed spacer 2, complete sequence; and large subunit ribosomal RNA gene, partial sequence	84.2	84.2	18%	1e-11	92.98%	MT774692.1
Campanula mugeana voucher EGE 43408 small subunit ribosomal RNA gene, partial sequence; internal transcribed spacer 1, 5.8S ribosomal RNA gene, and internal transcribed spacer 2, complete sequence; and large subunit ribosomal RNA gene, partial sequence	84.2	84.2	18%	1e-11	92.98%	MT774625.1

Description	Max Score	Total Score	Query Cover	E value	Per. Ident	Accession
Croninia kingiana 18S ribosomal RNA gene, partial sequence; internal transcribed spacer 1 and 5.8S ribosomal RNA gene, complete sequence; and internal transcribed spacer 2, partial sequence	84.2	84.2	15%	1e-11	97.92%	JF437568.1
Delphinium requienii voucher Erben (collect 6.6.1991) (M) clone Drequienii10 18S ribosomal RNA gene, internal transcribed spacer 1, 5.8S ribosomal RNA gene, internal transcribed spacer 2, and 28S ribosomal RNA gene, region	84.2	84.2	15%	1e-11	97.92%	JF332018.1
Krameria argentea isolate 10 clone 11 18S ribosomal RNA gene, partial sequence; internal transcribed spacer 1, 5.8S ribosomal RNA gene and internal transcribed spacer 2, complete sequence; and 25S ribosomal RNA gene, partial sequence	84.2	84.2	17%	1e-11	94.44%	AY260977.1
Helleborus foetidus internal transcribed spacer 2 (ITS2), 5.8S ribosomal RNA and internal transcribed spacer 1 (ITS1), specimen voucher McLewin K2, K	84.2	84.2	15%	1e-11	97.92%	AJ347892.1
Campanula yildirimlii voucher Campanula_yildirimlii_HY3060 internal transcribed spacer 1, partial sequence; 5.8S ribosomal RNA gene, complete sequence; and internal transcribed spacer 2, partial sequence	82.4	82.4	18%	4e-11	92.86%	OL764507.1
Campanula quercetorum voucher Campanula_quercetorum_HY3464b internal transcribed spacer 1, partial sequence; 5.8S ribosomal RNA gene, complete sequence; and internal transcribed spacer 2, partial sequence	82.4	82.4	18%	4e-11	92.86%	OL764506.1
Campanula quercetorum voucher Campanula_quercetorum_HY3464a internal transcribed spacer 1, partial sequence; 5.8S ribosomal RNA gene, complete sequence; and internal transcribed spacer 2, partial sequence	82.4	82.4	18%	4e-11	92.86%	OL764505.1
Campanula sp. BO-2021 voucher Campanula_dersimiana_Mfirat28888b internal transcribed spacer 1, partial sequence; 5.8S ribosomal RNA gene, complete sequence; and internal transcribed spacer 2, partial sequence	82.4	82.4	18%	4e-11	92.86%	OL764504.1

Description	Max Score	Total Score	Query Cover	E value	Per. Ident	Accession
Campanula sp. BO-2021 voucher Campanula_dersimiana_Mfirat28888a internal transcribed spacer 1, partial sequence; 5.8S ribosomal RNA gene, complete sequence; and internal transcribed spacer 2, partial sequence	82.4	82.4	18%	4e-11	92.86%	OL764503.1
Hypericum androsaemum isolate BPTPS187 small subunit ribosomal RNA gene, partial sequence; internal transcribed spacer 1, 5.8S ribosomal RNA gene, and internal transcribed spacer 2, complete sequence; and large subunit ribosomal RNA gene, partial sequence	82.4	82.4	17%	4e-11	94.34%	ON685486.1
Hypericum androsaemum isolate BPTPS150 small subunit ribosomal RNA gene, partial sequence; internal transcribed spacer 1, 5.8S ribosomal RNA gene, and internal transcribed spacer 2, complete sequence; and large subunit ribosomal RNA gene, partial sequence	82.4	82.4	17%	4e-11	94.34%	ON685450.1
Priogymnanthus hasslerianus voucher P T. Rojas 10694 (P03384284) external transcribed spacer, partial sequence; small subunit ribosomal RNA gene, internal transcribed spacer 1, 5.8S ribosomal RNA gene, internal transcribed spacer 2, and large subunit ribosomal RNA gene, complete sequence; and external transcribed spacer, partial sequence	82.4	82.4	17%	4e-11	94.44%	MW046253.1
Borago officinalis isolate BPTPS175 small subunit ribosomal RNA gene, partial sequence; internal transcribed spacer 1, 5.8S ribosomal RNA gene, and internal transcribed spacer 2, complete sequence; and large subunit ribosomal RNA gene, partial sequence	82.4	82.4	15%	4e-11	97.87%	OM670241.1
Monotropa uniflora isolate Broe 108 internal transcribed spacer 1, partial sequence; 5.8S ribosomal RNA gene and internal transcribed spacer 2, complete sequence; and large subunit ribosomal RNA gene, partial sequence	82.4	82.4	15%	4e-11	97.87%	MZ063683.1
Monotropa uniflora isolate Broe 104 internal transcribed spacer 1, partial sequence; 5.8S ribosomal RNA gene and internal transcribed spacer 2, complete sequence; and large subunit ribosomal RNA gene, partial sequence	82.4	82.4	15%	4e-11	97.87%	MZ063682.1

Description	Max Score	Total Score	Query Cover	E value	Per. Ident	Accession
Monotropa uniflora isolate Broe 103 internal transcribed spacer 1, partial sequence; 5.8S ribosomal RNA gene and internal transcribed spacer 2, complete sequence; and large subunit ribosomal RNA gene, partial sequence	82.4	82.4	15%	4e-11	97.87%	MZ063681.1
Monotropa uniflora isolate Broe 102 internal transcribed spacer 1, partial sequence; 5.8S ribosomal RNA gene and internal transcribed spacer 2, complete sequence; and large subunit ribosomal RNA gene, partial sequence	82.4	82.4	15%	4e-11	97.87%	MZ063680.1
Monotropa brittonii isolate Broe 100 internal transcribed spacer 1, partial sequence; 5.8S ribosomal RNA gene and internal transcribed spacer 2, complete sequence; and large subunit ribosomal RNA gene, partial sequence	82.4	82.4	15%	4e-11	97.87%	MZ063679.1
Monotropa brittonii isolate Broe 99 internal transcribed spacer 1, partial sequence; 5.8S ribosomal RNA gene and internal transcribed spacer 2, complete sequence; and large subunit ribosomal RNA gene, partial sequence	82.4	82.4	15%	4e-11	97.87%	MZ063678.1
Monotropa uniflora isolate Broe 91 internal transcribed spacer 1, partial sequence; 5.8S ribosomal RNA gene and internal transcribed spacer 2, complete sequence; and large subunit ribosomal RNA gene, partial sequence	82.4	82.4	15%	4e-11	97.87%	MZ063677.1
Monotropa brittonii isolate Broe 89 internal transcribed spacer 1, partial sequence; 5.8S ribosomal RNA gene and internal transcribed spacer 2, complete sequence; and large subunit ribosomal RNA gene, partial sequence	82.4	82.4	15%	4e-11	97.87%	MZ063676.1
Monotropa brittonii isolate Broe 88 internal transcribed spacer 1, partial sequence; 5.8S ribosomal RNA gene and internal transcribed spacer 2, complete sequence; and large subunit ribosomal RNA gene, partial sequence	82.4	82.4	15%	4e-11	97.87%	MZ063675.1
Monotropa brittonii isolate Broe 87 internal transcribed spacer 1, partial sequence; 5.8S ribosomal RNA gene and internal transcribed spacer 2, complete sequence; and large subunit ribosomal RNA gene, partial sequence	82.4	82.4	15%	4e-11	97.87%	MZ063674.1

Description	Max Score	Total Score	Query Cover	E value	Per. Ident	Accession
Monotropa brittonii isolate Broe 86 internal transcribed spacer 1, partial sequence; 5.8S ribosomal RNA gene and internal transcribed spacer 2, complete sequence; and large subunit ribosomal RNA gene, partial sequence	82.4	82.4	15%	4e-11	97.87%	MZ063673.1
Monotropa uniflora isolate Broe 71 internal transcribed spacer 1, partial sequence; 5.8S ribosomal RNA gene and internal transcribed spacer 2, complete sequence; and large subunit ribosomal RNA gene, partial sequence	82.4	82.4	15%	4e-11	97.87%	MZ063672.1
