# Supplementary material for: A Multi-Pronged Technique for Identifying Equisetum palustre and Equisetum arvense—Combining HPTLC, HPLC-ESI-MS/MS and Optimized DNA Barcoding Techniques
Source: Plants (Basel). 2022 Sep 28;11(19):2562. doi: 10.3390/plants11192562 (PMC9573248; doi:10.3390/plants11192562)
Supplement: Supplementary file 1 [file plants-11-02562-s001.zip › plants-1932149 supplementary/Horsetail Supplementary Figures/Figure S16.pdf]

BLAST ® » blastn suite » RID-FD4YEJAJ013

BLAST Results

Questions/comments

Job title: MT198943.1

**RID**	FD4YEJAJ013 (Expires on 08-14 01:54 am)
**Query ID**	lcl|Query_55687
**Description**	None
**Molecule type**	dna
**Query Length**	739
**Database Name**	nt
**Description**	Nucleotide collection (nt)
**Program**	BLASTN 2.13.0+

Graphic Summary

Distribution of the top 166 Blast Hits on 100 subject sequences

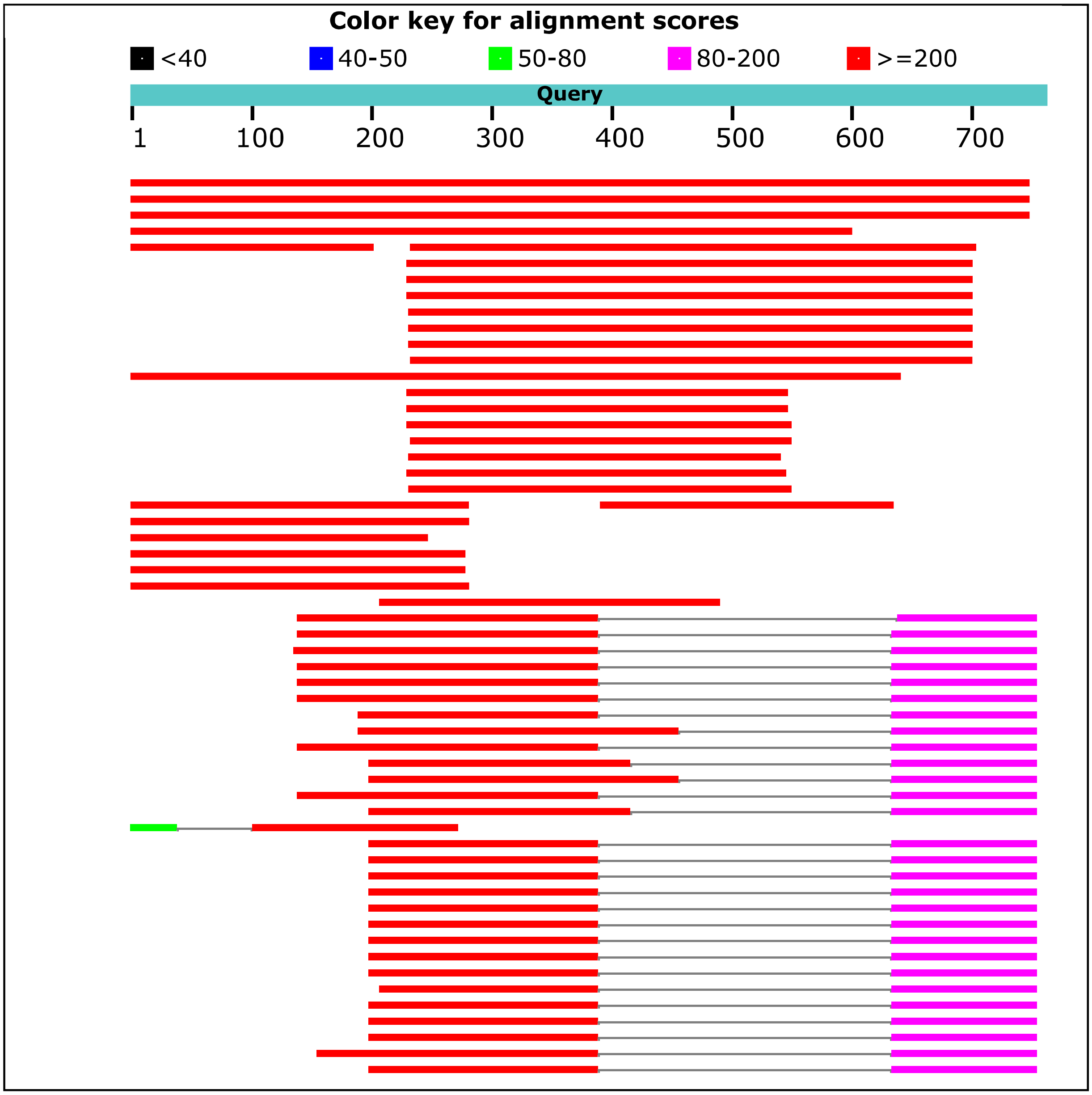

Descriptions

Sequences producing significant alignments:

Description	Max Score	Total Score	Query Cover	E value	Per. Ident	Accession
Equisetum environmental sample clone EDNA16-0043490 internal transcribed spacer 1, partial sequence; 5.8S ribosomal RNA gene and internal transcribed spacer 2, complete sequence; and large subunit ribosomal RNA gene, partial sequence	1358	1358	100%	0.0	100.00%	MT198943.1
Equisetum ramosissimum isolate BPTPS123 small subunit ribosomal RNA gene, partial sequence; internal transcribed spacer 1, 5.8S ribosomal RNA gene, and internal transcribed spacer 2, complete sequence; and large subunit ribosomal RNA gene, partial sequence	1018	1018	100%	0.0	91.53%	ON685433.1
Equisetum ramosissimum isolate BPTPS056 small subunit ribosomal RNA gene, partial sequence; internal transcribed spacer 1, 5.8S ribosomal RNA gene, and internal transcribed spacer 2, complete sequence; and large subunit ribosomal RNA gene, partial sequence	970	970	100%	0.0	90.35%	OM670239.1
Equisetum hyemale genes for ITS1, 5.8S rRNA, ITS2, partial sequence, bio_material: KB:NIBR0000051239	808	808	80%	0.0	91.26%	AB817880.1

Description	Max Score	Total Score	Query Cover	E value	Per. Ident	Accession
Equisetum palustre isolate 82713220323 5.8S ribosomal RNA gene, partial sequence; internal transcribed spacer 2, complete sequence; and large subunit ribosomal RNA gene, partial sequence	798	798	62%	0.0	97.64%	ON713474.1
Equisetum palustre isolate 82716220323 5.8S ribosomal RNA gene, partial sequence; internal transcribed spacer 2, complete sequence; and large subunit ribosomal RNA gene, partial sequence	797	797	62%	0.0	97.63%	ON713477.1
Equisetum palustre isolate 82714220323 5.8S ribosomal RNA gene, partial sequence; internal transcribed spacer 2, complete sequence; and large subunit ribosomal RNA gene, partial sequence	797	797	62%	0.0	97.63%	ON713475.1
Equisetum palustre isolate 82712220323 5.8S ribosomal RNA gene, partial sequence; internal transcribed spacer 2, complete sequence; and large subunit ribosomal RNA gene, partial sequence	797	797	62%	0.0	97.63%	ON713473.1
Equisetum palustre isolate 82715220323 5.8S ribosomal RNA gene, partial sequence; internal transcribed spacer 2, complete sequence; and large subunit ribosomal RNA gene, partial sequence	795	795	62%	0.0	97.63%	ON713476.1

Description	Max Score	Total Score	Query Cover	E value	Per. Ident	Accession
Equisetum palustre isolate 82711220323 5.8S ribosomal RNA gene, partial sequence; internal transcribed spacer 2, complete sequence; and large subunit ribosomal RNA gene, partial sequence	795	795	62%	0.0	97.63%	ON713472.1
Equisetum palustre isolate 82710220323 5.8S ribosomal RNA gene, partial sequence; internal transcribed spacer 2, complete sequence; and large subunit ribosomal RNA gene, partial sequence	795	795	62%	0.0	97.63%	ON713471.1
Equisetum palustre isolate 82709220323 5.8S ribosomal RNA gene, partial sequence; internal transcribed spacer 2, complete sequence; and large subunit ribosomal RNA gene, partial sequence	795	795	62%	0.0	97.63%	ON713470.1
Equisetum ramosissimum 18S ribosomal RNA gene, partial sequence; internal transcribed spacer 1, 5.8S ribosomal RNA gene and internal transcribed spacer 2, complete sequence; and 26S ribosomal RNA gene, partial sequence	780	780	85%	0.0	88.92%	AF448794.1
Equisetum arvense isolate 82704220323 5.8S ribosomal RNA gene and internal transcribed spacer 2, partial sequence	558	558	42%	4e-154	99.04%	ON713479.1
Equisetum arvense isolate 82701220323 5.8S ribosomal RNA gene and internal transcribed spacer 2, partial sequence	553	553	42%	2e-152	98.71%	ON876507.1

Description	Max Score	Total Score	Query Cover	E value	Per. Ident	Accession
Equisetum arvense isolate 84087220504 5.8S ribosomal RNA gene and internal transcribed spacer 2, partial sequence	553	553	42%	2e-152	98.41%	ON713485.1
Equisetum arvense isolate 83919220502 5.8S ribosomal RNA gene and internal transcribed spacer 2, partial sequence	553	553	42%	2e-152	98.72%	ON713484.1
Equisetum arvense isolate 82702220323 5.8S ribosomal RNA gene and internal transcribed spacer 2, partial sequence	553	553	41%	2e-152	99.34%	ON713480.1
Equisetum arvense isolate 82703220323 5.8S ribosomal RNA gene and internal transcribed spacer 2, partial sequence	551	551	41%	6e-152	98.71%	ON713478.1
Equisetum arvense isolate 82708220323 5.8S ribosomal RNA gene and internal transcribed spacer 2, partial sequence	492	492	42%	4e-134	94.90%	ON876508.1
Equisetum arvense isolate L internal transcribed spacer 2, partial sequence	412	412	32%	3e-110	97.90%	ON568505.1
Equisetum variegatum 18S ribosomal RNA gene, partial sequence; internal transcribed spacer 1, complete sequence; and 5.8S ribosomal RNA gene, partial sequence	351	351	37%	7e-92	89.53%	EU328340.1
Equisetum x ferrissii 18S ribosomal RNA gene, partial sequence; internal transcribed spacer 1, complete sequence; and 5.8S ribosomal RNA gene, partial sequence	344	344	37%	1e-89	88.77%	EU328338.1
Equisetum pratense internal transcribed spacer 1, partial sequence	344	344	32%	1e-89	92.24%	DQ377152.1

Description	Max Score	Total Score	Query Cover	E value	Per. Ident	Accession
Equisetum telmateia subsp. telmateia internal transcribed spacer 1, partial sequence	339	339	26%	5e-88	97.47%	DQ377154.1
Equisetum variegatum voucher B. Hoshizaki 592 18S ribosomal RNA gene, partial sequence; internal transcribed spacer 1, complete sequence; and 5.8S ribosomal RNA gene, partial sequence	337	337	36%	2e-87	88.64%	EU372664.1
Equisetum hyemale subsp. affine 18S ribosomal RNA gene, partial sequence; internal transcribed spacer 1, complete sequence; and 5.8S ribosomal RNA gene, partial sequence	322	322	36%	5e-83	87.55%	EU328339.1
Equisetum laevigatum 18S ribosomal RNA gene, partial sequence; internal transcribed spacer 1, complete sequence; and 5.8S ribosomal RNA gene, partial sequence	318	318	37%	7e-82	86.59%	EU328341.1
Pilularia americana isolate CBP331 18S ribosomal RNA gene, partial sequence; internal transcribed spacer 1, 5.8S ribosomal RNA gene, and internal transcribed spacer 2, complete sequence; and 26S ribosomal RNA gene, partial sequence	291	291	37%	1e-73	85.82%	MF964017.1

Description	Max Score	Total Score	Query Cover	E value	Per. Ident	Accession
Campyloneurum sphenodes voucher P.C. Standley 34246 (PE) small subunit ribosomal RNA gene, internal transcribed spacer 1, 5.8S ribosomal RNA gene, internal transcribed spacer 2, and large subunit ribosomal RNA gene, complete sequence	274	454	48%	1e-68	86.90%	MZ360986.1
Selliguea metacoela voucher R. Wei 2925 (PE) small subunit ribosomal RNA gene, internal transcribed spacer 1, 5.8S ribosomal RNA gene, internal transcribed spacer 2, and large subunit ribosomal RNA gene, complete sequence	268	456	48%	7e-67	86.45%	MZ361045.1
Microgramma vacciniifolia voucher C.X. Li RS281 small subunit ribosomal RNA gene, internal transcribed spacer 1, 5.8S ribosomal RNA gene, internal transcribed spacer 2, and large subunit ribosomal RNA gene, complete sequence	268	456	49%	7e-67	86.38%	MZ361014.1
Selliguea dareiformis voucher R. Wei ST1108 (PE) small subunit ribosomal RNA gene, internal transcribed spacer 1, 5.8S ribosomal RNA gene, internal transcribed spacer 2, and large subunit ribosomal RNA gene, complete sequence	268	452	48%	7e-67	86.45%	MZ361000.1

Description	Max Score	Total Score	Query Cover	E value	Per. Ident	Accession
Adetogramma chrysolepis voucher Alfredo Tupayachi H. 758 (A) small subunit ribosomal RNA gene, internal transcribed spacer 1, 5.8S ribosomal RNA gene, internal transcribed spacer 2, and large subunit ribosomal RNA gene, complete sequence	268	445	48%	7e-67	86.51%	MZ360977.1
Selliguea taeniata voucher B.Y. Sun & al. 36 (PE) small subunit ribosomal RNA gene, internal transcribed spacer 1, 5.8S ribosomal RNA gene, internal transcribed spacer 2, and large subunit ribosomal RNA gene, complete sequence	263	450	48%	3e-65	86.17%	MZ361026.1
Leptochilus pteropus voucher R. Wei WR0281 (PE) small subunit ribosomal RNA gene, internal transcribed spacer 1, 5.8S ribosomal RNA gene, internal transcribed spacer 2, and large subunit ribosomal RNA gene, complete sequence	263	439	42%	3e-65	90.95%	MZ361018.1
Leptochilus henryi voucher X.C. Zhang 7487 (PE) small subunit ribosomal RNA gene, internal transcribed spacer 1, 5.8S ribosomal RNA gene, internal transcribed spacer 2, and large subunit ribosomal RNA gene, complete sequence	263	439	51%	3e-65	84.73%	MZ360988.1

Description	Max Score	Total Score	Query Cover	E value	Per. Ident	Accession
Selliguea heterocarpa voucher R. Wei & al. 353 (PE) small subunit ribosomal RNA gene, internal transcribed spacer 1, 5.8S ribosomal RNA gene, internal transcribed spacer 2, and large subunit ribosomal RNA gene, complete sequence	261	449	48%	1e-64	85.66%	MZ361044.1
Polypodium rhodopleuron voucher A.M. Evans & al. 2871 (PE) small subunit ribosomal RNA gene, internal transcribed spacer 1, 5.8S ribosomal RNA gene, internal transcribed spacer 2, and large subunit ribosomal RNA gene, complete sequence	261	438	44%	1e-64	88.84%	MZ361037.1
Leptochilus ellipticus voucher R. Wei WR0211 (PE) small subunit ribosomal RNA gene, internal transcribed spacer 1, 5.8S ribosomal RNA gene, internal transcribed spacer 2, and large subunit ribosomal RNA gene, complete sequence	261	438	50%	1e-64	85.34%	MZ360987.1
Lepisorus spicatus voucher R. Wei 29 (PE) small subunit ribosomal RNA gene, internal transcribed spacer 1, 5.8S ribosomal RNA gene, internal transcribed spacer 2, and large subunit ribosomal RNA gene, complete sequence	261	443	48%	1e-64	85.99%	MZ360983.1

Description	Max Score	Total Score	Query Cover	E value	Per. Ident	Accession
Phlebodium pseudoaureum voucher WR0622 (PE) small subunit ribosomal RNA gene, internal transcribed spacer 1, 5.8S ribosomal RNA gene, internal transcribed spacer 2, and large subunit ribosomal RNA gene, complete sequence	259	447	44%	4e-64	88.84%	MZ361022.1
Equisetum sylvaticum 18S ribosomal RNA gene, partial sequence; internal transcribed spacer 1, complete sequence; and 5.8S ribosomal RNA gene, partial sequence	259	327	27%	4e-64	94.58%	EU328342.1
Selliguea albidoglauca voucher X.C. Zhang 7609 (PE) small subunit ribosomal RNA gene, internal transcribed spacer 1, 5.8S ribosomal RNA gene, internal transcribed spacer 2, and large subunit ribosomal RNA gene, complete sequence	257	441	41%	1e-63	91.53%	MZ361043.1
Polypodium vulgare voucher B. Liu 28227 (PE) small subunit ribosomal RNA gene, internal transcribed spacer 1, 5.8S ribosomal RNA gene, internal transcribed spacer 2, and large subunit ribosomal RNA gene, complete sequence	257	441	41%	1e-63	91.53%	MZ361039.1
Polypodium sibiricum voucher Zhang 7312 (PE) small subunit ribosomal RNA gene, internal transcribed spacer 1, 5.8S ribosomal RNA gene, internal transcribed spacer 2, and large subunit ribosomal RNA gene, complete sequence	257	441	41%	1e-63	91.53%	MZ361038.1

Description	Max Score	Total Score	Query Cover	E value	Per. Ident	Accession
Polypodium arcanum voucher M. Sundue & al. 3554 (PE) small subunit ribosomal RNA gene, internal transcribed spacer 1, 5.8S ribosomal RNA gene, internal transcribed spacer 2, and large subunit ribosomal RNA gene, complete sequence	257	434	41%	1e-63	91.53%	MZ361036.1
Goniophlebium niponicum voucher H.M. Liu A305 (PE) small subunit ribosomal RNA gene, internal transcribed spacer 1, 5.8S ribosomal RNA gene, internal transcribed spacer 2, and large subunit ribosomal RNA gene, complete sequence	257	441	41%	1e-63	91.49%	MZ361035.1
Goniophlebium amoenum voucher Z.Y. Li 1897 (PE) small subunit ribosomal RNA gene, internal transcribed spacer 1, 5.8S ribosomal RNA gene, internal transcribed spacer 2, and large subunit ribosomal RNA gene, complete sequence	257	436	41%	1e-63	91.53%	MZ361034.1
Goniophlebium mengtzeense voucher H.M. Liu GX211 (PE) small subunit ribosomal RNA gene, internal transcribed spacer 1, 5.8S ribosomal RNA gene, internal transcribed spacer 2, and large subunit ribosomal RNA gene, complete sequence	257	441	41%	1e-63	91.53%	MZ361033.1

Description	Max Score	Total Score	Query Cover	E value	Per. Ident	Accession
Goniophlebium argutum voucher R. Wei ST465 (PE) small subunit ribosomal RNA gene, internal transcribed spacer 1, 5.8S ribosomal RNA gene, internal transcribed spacer 2, and large subunit ribosomal RNA gene, complete sequence	257	441	41%	1e-63	91.53%	MZ361032.1
Selliguea griffithiana voucher X.C. Zhang 9580 (PE) small subunit ribosomal RNA gene, internal transcribed spacer 1, 5.8S ribosomal RNA gene, internal transcribed spacer 2, and large subunit ribosomal RNA gene, complete sequence	257	445	41%	1e-63	91.53%	MZ361025.1
Selliguea crenatopinnata voucher X.C. Zhang 6242 (PE) small subunit ribosomal RNA gene, internal transcribed spacer 1, 5.8S ribosomal RNA gene, internal transcribed spacer 2, and large subunit ribosomal RNA gene, complete sequence	257	441	39%	1e-63	92.78%	MZ361024.1
Selliguea chenopus voucher X.C. Zhang 6259 (PE) small subunit ribosomal RNA gene, internal transcribed spacer 1, 5.8S ribosomal RNA gene, internal transcribed spacer 2, and large subunit ribosomal RNA gene, complete sequence	257	445	41%	1e-63	91.53%	MZ361023.1

Description	Max Score	Total Score	Query Cover	E value	Per. Ident	Accession
Goniophlebium manmeiense voucher X.P. Qi Q061 (PE) small subunit ribosomal RNA gene, internal transcribed spacer 1, 5.8S ribosomal RNA gene, internal transcribed spacer 2, and large subunit ribosomal RNA gene, complete sequence	257	445	41%	1e-63	91.58%	MZ361013.1
Loxogramme avenia voucher R. Wei 2761 (PE) small subunit ribosomal RNA gene, internal transcribed spacer 1, 5.8S ribosomal RNA gene, internal transcribed spacer 2, and large subunit ribosomal RNA gene, complete sequence	257	445	41%	1e-63	91.53%	MZ361010.1
Lepisorus schraderi voucher X.C. Zhang 9057 (PE) small subunit ribosomal RNA gene, internal transcribed spacer 1, 5.8S ribosomal RNA gene, internal transcribed spacer 2, and large subunit ribosomal RNA gene, complete sequence	257	439	46%	1e-63	86.92%	MZ361007.1
Lepisorus carnosus voucher Voucher unknown small subunit ribosomal RNA gene, internal transcribed spacer 1, 5.8S ribosomal RNA gene, internal transcribed spacer 2, and large subunit ribosomal RNA gene, complete sequence	257	439	41%	1e-63	91.58%	MZ361002.1

Description	Max Score	Total Score	Query Cover	E value	Per. Ident	Accession
Oreogrammitis congener voucher R. Wei & al. 523 (PE) small subunit ribosomal RNA gene, internal transcribed spacer 1, 5.8S ribosomal RNA gene, internal transcribed spacer 2, and large subunit ribosomal RNA gene, complete sequence	257	443	48%	1e-63	85.77%	MZ360999.1
Goniophlebium formosanum voucher X.C. Zhang 9432 (PE) small subunit ribosomal RNA gene, internal transcribed spacer 1, 5.8S ribosomal RNA gene, internal transcribed spacer 2, and large subunit ribosomal RNA gene, complete sequence	257	441	41%	1e-63	91.49%	MZ360995.1
Aglaomorpha willdenowii voucher X.C. Zhang 9062 (PE) small subunit ribosomal RNA gene, internal transcribed spacer 1, 5.8S ribosomal RNA gene, internal transcribed spacer 2, and large subunit ribosomal RNA gene, complete sequence	257	445	41%	1e-63	91.53%	MZ360993.1
Bosmania membranacea subsp. carinata voucher R. Wei WR0410 (PE) small subunit ribosomal RNA gene, internal transcribed spacer 1, 5.8S ribosomal RNA gene, internal transcribed spacer 2, and large subunit ribosomal RNA gene, complete sequence	257	445	41%	1e-63	91.53%	MZ360985.1

Description	Max Score	Total Score	Query Cover	E value	Per. Ident	Accession
Bosmania membranacea var. membranacea voucher R. Wei WR0368 (PE) small subunit ribosomal RNA gene, internal transcribed spacer 1, 5.8S ribosomal RNA gene, internal transcribed spacer 2, and large subunit ribosomal RNA gene, complete sequence	257	445	41%	1e-63	91.53%	MZ360984.1
Selliguea mairei voucher X.C. Zhang 4609 (PE) small subunit ribosomal RNA gene, internal transcribed spacer 1, 5.8S ribosomal RNA gene, internal transcribed spacer 2, and large subunit ribosomal RNA gene, complete sequence	257	441	41%	1e-63	91.53%	MZ360981.1
Drynaria heraclea voucher R. Wei 470 (PE) small subunit ribosomal RNA gene, internal transcribed spacer 1, 5.8S ribosomal RNA gene, internal transcribed spacer 2, and large subunit ribosomal RNA gene, complete sequence	257	445	41%	1e-63	91.53%	MZ360979.1
Drynaria speciosa voucher R. Wei 508 (PE) small subunit ribosomal RNA gene, internal transcribed spacer 1, 5.8S ribosomal RNA gene, internal transcribed spacer 2, and large subunit ribosomal RNA gene, complete sequence	257	443	40%	1e-63	91.53%	MZ360978.1

Description	Max Score	Total Score	Query Cover	E value	Per. Ident	Accession
Asplenium scolopendrium isolate Copy2 voucher Vogel SCOL-175 Italy internal transcribed spacer 1, partial sequence; 5.8S ribosomal RNA gene, complete sequence; and internal transcribed spacer 2, partial sequence	257	366	33%	1e-63	92.78%	JX475163.1
Lygodium microphyllum isolate 2 voucher BOP017890 internal transcribed spacer 1, partial sequence; 5.8S ribosomal RNA gene, complete sequence; and internal transcribed spacer 2, partial sequence	255	255	41%	5e-63	82.11%	KJ562324.1
Lygodium microphyllum isolate 1 voucher BOP017890 internal transcribed spacer 1, partial sequence; 5.8S ribosomal RNA gene, complete sequence; and internal transcribed spacer 2, partial sequence	255	255	41%	5e-63	82.11%	KJ562323.1
Marsilea quadrifolia 18S ribosomal RNA gene, partial sequence; internal transcribed spacer 1, 5.8S ribosomal RNA gene and internal transcribed spacer 2, complete sequence; and 26S ribosomal RNA gene, partial sequence	255	255	21%	5e-63	96.18%	AF448792.1
Niphidium crassifolium voucher WR0621 (PE) small subunit ribosomal RNA gene, internal transcribed spacer 1, 5.8S ribosomal RNA gene, internal transcribed spacer 2, and large subunit ribosomal RNA gene, complete sequence	254	438	40%	2e-62	91.80%	MZ361021.1

Description	Max Score	Total Score	Query Cover	E value	Per. Ident	Accession
Microsorum steerei voucher R. Wei WR0620 (PE) small subunit ribosomal RNA gene, internal transcribed spacer 1, 5.8S ribosomal RNA gene, internal transcribed spacer 2, and large subunit ribosomal RNA gene, complete sequence	254	430	41%	2e-62	91.15%	MZ361020.1
Leptochilus macrophyllus voucher R. Wei & al., 441 (PE) small subunit ribosomal RNA gene, internal transcribed spacer 1, 5.8S ribosomal RNA gene, internal transcribed spacer 2, and large subunit ribosomal RNA gene, complete sequence	254	430	43%	2e-62	89.00%	MZ361009.1
Leptochilus decurrens voucher R. Wei WR0267 (PE) small subunit ribosomal RNA gene, internal transcribed spacer 1, 5.8S ribosomal RNA gene, internal transcribed spacer 2, and large subunit ribosomal RNA gene, complete sequence	254	430	44%	2e-62	88.13%	MZ361008.1
Lepisorus longifolius voucher R. Wei SIWI4 (PE) small subunit ribosomal RNA gene, internal transcribed spacer 1, 5.8S ribosomal RNA gene, internal transcribed spacer 2, and large subunit ribosomal RNA gene, complete sequence	254	436	46%	2e-62	86.67%	MZ361006.1

Description	Max Score	Total Score	Query Cover	E value	Per. Ident	Accession
Drynaria parishii voucher X.C. Zhang 7946 (PE) small subunit ribosomal RNA gene, internal transcribed spacer 1, 5.8S ribosomal RNA gene, internal transcribed spacer 2, and large subunit ribosomal RNA gene, complete sequence	254	441	41%	2e-62	91.01%	MZ360992.1
Thylacopteris papillosa voucher R. Wei 345 (PE) small subunit ribosomal RNA gene, internal transcribed spacer 1, 5.8S ribosomal RNA gene, internal transcribed spacer 2, and large subunit ribosomal RNA gene, complete sequence	252	432	38%	7e-62	93.57%	MZ361053.1
Synammia intermedia voucher C. Scottsberg 432 (NY) small subunit ribosomal RNA gene, internal transcribed spacer 1, 5.8S ribosomal RNA gene, internal transcribed spacer 2, and large subunit ribosomal RNA gene, complete sequence	252	432	41%	7e-62	91.01%	MZ361051.1
Synammia feuillei voucher Lendemer 16329 (NY) small subunit ribosomal RNA gene, internal transcribed spacer 1, 5.8S ribosomal RNA gene, internal transcribed spacer 2, and large subunit ribosomal RNA gene, complete sequence	252	430	41%	7e-62	91.01%	MZ361050.1

Description	Max Score	Total Score	Query Cover	E value	Per. Ident	Accession
Synammia espinosae voucher I.M. Johnston 5306 (NY) small subunit ribosomal RNA gene, internal transcribed spacer 1, 5.8S ribosomal RNA gene, internal transcribed spacer 2, and large subunit ribosomal RNA gene, complete sequence	252	434	41%	7e-62	91.01%	MZ361049.1
Serpocaulon levigatum voucher R. Rojas & G. Ortiz 5315 (PE) small subunit ribosomal RNA gene, internal transcribed spacer 1, 5.8S ribosomal RNA gene, internal transcribed spacer 2, and large subunit ribosomal RNA gene, complete sequence	252	439	41%	7e-62	91.01%	MZ361047.1
Serpocaulon fraxinifolium voucher G. Hatschbach & al. 73838 (PE) small subunit ribosomal RNA gene, internal transcribed spacer 1, 5.8S ribosomal RNA gene, internal transcribed spacer 2, and large subunit ribosomal RNA gene, complete sequence	252	439	41%	7e-62	91.01%	MZ361046.1
Pyrrosia penangiana voucher R. Wei 3014 (PE) small subunit ribosomal RNA gene, internal transcribed spacer 1, 5.8S ribosomal RNA gene, internal transcribed spacer 2, and large subunit ribosomal RNA gene, complete sequence	252	436	41%	7e-62	91.01%	MZ361041.1

Description	Max Score	Total Score	Query Cover	E value	Per. Ident	Accession
Pleurosoriopsis makinoi voucher X.C. Zhang 9478 (PE) small subunit ribosomal RNA gene, internal transcribed spacer 1, 5.8S ribosomal RNA gene, internal transcribed spacer 2, and large subunit ribosomal RNA gene, complete sequence	252	436	41%	7e-62	91.01%	MZ361031.1
Pleopeltis polypodioides voucher H. Shang 28B (PE) small subunit ribosomal RNA gene, internal transcribed spacer 1, 5.8S ribosomal RNA gene, internal transcribed spacer 2, and large subunit ribosomal RNA gene, complete sequence	252	439	38%	7e-62	93.57%	MZ361030.1
Microsorum cuspidatum voucher X.C. Zhang 4918 (PE) small subunit ribosomal RNA gene, internal transcribed spacer 1, 5.8S ribosomal RNA gene, internal transcribed spacer 2, and large subunit ribosomal RNA gene, complete sequence	252	434	41%	7e-62	91.05%	MZ361027.1
Loxogramme lankokiensis voucher H.M. Liu LHM1938 (PE) small subunit ribosomal RNA gene, internal transcribed spacer 1, 5.8S ribosomal RNA gene, internal transcribed spacer 2, and large subunit ribosomal RNA gene, complete sequence	252	436	41%	7e-62	91.01%	MZ361011.1

Description	Max Score	Total Score	Query Cover	E value	Per. Ident	Accession
Lepisorus clathratus voucher Voucher unknown small subunit ribosomal RNA gene, internal transcribed spacer 1, 5.8S ribosomal RNA gene, internal transcribed spacer 2, and large subunit ribosomal RNA gene, complete sequence	252	434	41%	7e-62	91.05%	MZ361004.1
Lepisorus hederaceus voucher Voucher unknown small subunit ribosomal RNA gene, internal transcribed spacer 1, 5.8S ribosomal RNA gene, internal transcribed spacer 2, and large subunit ribosomal RNA gene, complete sequence	252	434	41%	7e-62	91.05%	MZ361003.1
Goniophlebium persicifolium voucher X.C. Zhang 433 (PE) small subunit ribosomal RNA gene, internal transcribed spacer 1, 5.8S ribosomal RNA gene, internal transcribed spacer 2, and large subunit ribosomal RNA gene, complete sequence	252	439	41%	7e-62	91.05%	MZ360996.1
Lepisorus miyoshianus voucher X.C. Zhang 4511 (PE) small subunit ribosomal RNA gene, internal transcribed spacer 1, 5.8S ribosomal RNA gene, internal transcribed spacer 2, and large subunit ribosomal RNA gene, complete sequence	252	434	41%	7e-62	91.05%	MZ360991.1

Description	Max Score	Total Score	Query Cover	E value	Per. Ident	Accession
Dictymia brownii voucher R. Coveny 10858 (PE) small subunit ribosomal RNA gene, internal transcribed spacer 1, 5.8S ribosomal RNA gene, internal transcribed spacer 2, and large subunit ribosomal RNA gene, complete sequence	252	439	41%	7e-62	91.01%	MZ360990.1
Selliguea capitellata voucher X.C. Zhang 6341 (PE) small subunit ribosomal RNA gene, internal transcribed spacer 1, 5.8S ribosomal RNA gene, internal transcribed spacer 2, and large subunit ribosomal RNA gene, complete sequence	252	439	41%	7e-62	91.05%	MZ360982.1
Selliguea lehmannii voucher X.C. Zhang 5963 (PE) small subunit ribosomal RNA gene, internal transcribed spacer 1, 5.8S ribosomal RNA gene, internal transcribed spacer 2, and large subunit ribosomal RNA gene, complete sequence	252	439	38%	7e-62	93.57%	MZ360980.1
Asplenium normale isolate Copy1 voucher Ranker 1799 Hawaii internal transcribed spacer 1, partial sequence; 5.8S ribosomal RNA gene, complete sequence; and internal transcribed spacer 2, partial sequence	252	360	31%	7e-62	94.05%	JX475159.1
Jubula hutchinsiae subsp. javanica 18S rRNA gene (partial), ITS1, 5.8S rRNA gene, ITS2 and 28S rRNA gene (partial), specimen voucher HSNU:Zhu et al. 20050903-7a	252	252	23%	7e-62	93.10%	FN396801.1

Description	Max Score	Total Score	Query Cover	E value	Per. Ident	Accession
Azolla filiculoides voucher Reid & Peters 68 internal transcribed spacer 1, partial sequence; 5.8S ribosomal RNA gene, complete sequence; and internal transcribed spacer 2, partial sequence	252	252	24%	7e-62	91.76%	DQ066494.1
Serpocaulon loriceum voucher R.M. Rueda & al. 14546 (PE) small subunit ribosomal RNA gene, partial sequence; and internal transcribed spacer 1, 5.8S ribosomal RNA gene, internal transcribed spacer 2, and large subunit ribosomal RNA gene, complete sequence	250	434	41%	2e-61	91.01%	MZ361048.1
Pyrrosia angustata voucher Q. Wei wq004 (PE) small subunit ribosomal RNA gene, internal transcribed spacer 1, 5.8S ribosomal RNA gene, internal transcribed spacer 2, and large subunit ribosomal RNA gene, complete sequence	250	438	37%	2e-61	95.54%	MZ361040.1
Microsorum punctatum voucher Mt. Shiwan Exp. 148 (PE) small subunit ribosomal RNA gene, internal transcribed spacer 1, 5.8S ribosomal RNA gene, internal transcribed spacer 2, and large subunit ribosomal RNA gene, complete sequence	250	426	37%	2e-61	95.54%	MZ361019.1

Alignments

BLAST is a registered trademark of the National Library of Medicine

Support center Mailing list YouTube

- National Library Of Medicine
- National Institutes Of Health
- U.S. Department of Health & Human Services
- USA.gov

NCBI

National Center for Biotechnology Information, U.S. National Library of Medicine 8600 Rockville Pike, Bethesda MD, 20894 USA

Policies and Guidelines | Contact

HHS Vulnerability Disclosure
