# Supplementary material for: A Multi-Pronged Technique for Identifying Equisetum palustre and Equisetum arvense—Combining HPTLC, HPLC-ESI-MS/MS and Optimized DNA Barcoding Techniques
Source: Plants (Basel). 2022 Sep 28;11(19):2562. doi: 10.3390/plants11192562 (PMC9573248; doi:10.3390/plants11192562)
Supplement: Supplementary file 1 [file plants-11-02562-s001.zip › plants-1932149 supplementary/Horsetail Supplementary Figures/Figure S17.pdf]

```
Y11470.1               ------------------------------------------------------------
MW040324.1             ------------------------------------------------------------
MW040322.1             ------------------------------------------------------------
MW040323.1             ------------------------------------------------------------
OL304250.1             ------------------------------------------------------------
OL304248.1             ------------------------------------------------------------
OL304249.1             ------------------------------------------------------------
Y11471.1               ------------------------------------------------------------
A7_(82708220323)       -GACTCTCGGCAACGGATATCTTGGCTCTTGCAACGATGAAGAACGCAGCGAAATGCGAT
A9_(84087220504)       CGACTCTCGGCAACGGATATCTcGGCTCTcGCtACGATGAAGAACGCAGCGAAATGCGAT
A4_(82702220323)       aGACTCTCGGCAACGGATATCTTGGCTCTTGCAACGATGAAGAACGCAGCGAAATGCGAT
A3_(82701220323)       CGACTCTCGGCAACGGATATCTTGGCTCTTGCAACGATGAAGAACGCAGCGAAATGCGAT
A6_(82704220323)       CGACTCTCGGCAACGGATATCTTGGCTCTTGCAACGATGAAGAACGCAGCGAAATGCGAT
A8_(83919220502)       --ACTCTCGGCAACGGATATCTTGGCTCTTGCAACGATGAAGAACGCAGCGAAATGCGAT
A5_(82703220323)       CGACTCTCGGCAACGGATATCTTGGCTCTTGCAACGATGAAGAACGCAGCGAAATGCGAT

Y11470.1               --------------cCAcgcaTCCGtG------CaAAcCcT-----GCAAATTGtG----
MW040324.1             -------------TGCAGAATTCCGtGAATCATCGAgTCTTTGAACGCAtATTGCGCCCG
MW040322.1             -------------TGCAGAATTCCGtGAATCATCGAgTCTTTGAACGCAtATTGCGCnCG
MW040323.1             -------------TGCAGAATTCCGtGAATCATCGAgTCTTTGAACGCAtATTGCGCCCG
OL304250.1             -------------TGCAGAATTCCGtGAATCATCGAgTCTTTGAACGCAtATTGCGCCCG
OL304248.1             -------------TGCAGAATTCCGtGAATCATCGAgTCTTTGAACGCAtATTGCGCCgG
OL304249.1             -------------TGCAGAATTCCGtGAATCATCGAgTCTTTGAACGCAtATTGCGCCgG
Y11471.1               --------------------------------TacAATCT----------ATTGCGC---
A7_(82708220323)       ACGTAGTGTGAATTGCAGAATTCCGCGAATCATCGAATCTTTGAACGCAAcTcGCGCCCG
A9_(84087220504)       ACGTAGTGTGAATTGCAGAATTCCGCGAATCATCGAgTCTTTGAACGCAAcTcGCGCCCG
A4_(82702220323)       ACGTAGTGTGAATTGCAGAATTCCGCGAATCATCGAATCTTTGAACGCAAcTcGCGCCCG
A3_(82701220323)       ACGTAGTGTGAATTGCAGAATTCCGCGAATCATCGAATCTTTGAACGCAAcTcGCGCCCG
A6_(82704220323)       ACGTAGTGTGAATTGCAGAATTCCGCGAATCATCGAATCTTTGAACGCAAcTcGCGCCCG
A8_(83919220502)       ACGTAGTGTGAATTGCAGAATTCCGCGAATCATCGAATCTTTGAACGCAAcTcGCGCCCG
A5_(82703220323)       ACGTAGTGTGAATTGCAGAATTCCGCGAATCATCGAATCTTTGAACGCAAcTcGCGCCCG

Y11470.1               -GaCaTCcCCCaAGGaggcGaCT-------------TcaAtACCCTcCtgCGCCTgtCC-
MW040324.1             AGGCtTCGgtCaAGGGCATGTCTGCCTcAGCGTCggTtgAccCCtctCGtCcaaTtCCCt
MW040322.1             AGGCtTCGgtCaAGGGCATGTCTGCCTcAGCGTCggTtgAccCCtctCGtCcaaTtCCCt
MW040323.1             AGGCtTCGgtCnAGGGCATGTCTGCCTcAGCGTCggTtgAccCCtctCGtCcaaTtCCCt
OL304250.1             AGGCtTCGgtCaAGGGCATGTCTGCCTcAGCGTCggTtgAccCCtctCGtCcaaTtCCCt
OL304248.1             AGGCtTCGgtCaAGGGCATGTCTGCCTcAGCGTCggTtgAccCCtctCGtCcaaTtCCCt
OL304249.1             AGGCtTCGgtCaAGGGCATGTCTGCCTcAGCGTCggTtgAccCCtctCGtCcaaTtCCCt
Y11471.1               --------------------------------------------------------CtCC
A7_(82708220323)       AGGgCTCGCCtGAGGaCATGTtTGCCTGAGCGTCCTTACAAAtCCTACGCCGCCTCCCCC
A9_(84087220504)       AGGgCTCGCCCGAGGGCATGTCTGCCTGAGCGTCCTTACAAAtCCTACGCCGCCTCCCCC
A4_(82702220323)       AGGgCTCGCCCGAGGGCATGTCTGCCTGAGCGTCCTTACAAAtCCTACGCCGCCTCCCCC
A3_(82701220323)       AGGgCTCGCCCGAGGGCATGTCTGCCTGAGCGTCCTTACAAAtCCTACGCCGCCTCCCCC
A6_(82704220323)       AGGgCTCGCCCGAGGGCATGTCTGCCTGAGCGTCCTTACAAAtCCTACGCCGCCTCCCCC
A8_(83919220502)       AGGgCTCGCCCGAGGGCATGTCTGCCTGAGCGTCCTTACAAAtCCTACGCCGCCTCCCCC
A5_(82703220323)       AGGgCTCGCCCGAGGGCATGTCTGCCTGAGCGTCCTTACAAAtCCTACGCCGCCTCCCCC

Y11470.1               ------------GtCcGAC------aTCCaGggGtCaActtCtGccCTCCgatgGAGCAc
MW040324.1             CGnGGAaGcA--GACGGACcTGGnaGTCCCGCTGGCGAcAGCGGGTCTgCTGCAGAaCAG
MW040322.1             CGtGGAaGcA--GACGGACcTGGCaGTCCCGCTGGCGAcAGCGGGTCTgCTGCAGAaCAG
MW040323.1             CGtGGAaGcA--GACGGACcTGGCaGTCCCGCTGGCGAcAGCGGGTCTgCTGCAGAaCAG
OL304250.1             CGtGGAaGcA--GACGGACcTGGCaGTCCCGCTGGCGAcAGCGGGTCTgCTGCAGAaCAG
OL304248.1             CG-GGAaGcA--GACGGACcTGGCaGTCCCGCTGGCGAcAGCGGGTCTgCTGCAGAaCAG
OL304249.1             CGaGGAaGcA--GACGGACcTGGCaGTCCCGCTGGCGAcAGCGGGTCTgCTGCAGAaCAG
Y11471.1               CGtaGAGGGAGCGAC-aACGTGGtCGTC------GCaA--------CTCCTcCAGAG-gc
A7_(82708220323)       CaCcGgGGGAGCGgCGGAtGTGGtCGTCC-----GCGAA-------CTCCTcCAGAGCAc
A9_(84087220504)       CGCGGgGGGAGCGgCGGACGTGGCCGTCC-----GCGAA-------CTCCTcCgGAGCgc
A4_(82702220323)       CGCGGgGGGAGCGgCGGACGTGGCCGTCC-----GCGAA-------CTCCTcCgGAGCgc
A3_(82701220323)       CGCGGgGGGAGCGgCGGACGTGGCCGTCC-----GCGAA-------CTCCTtCgGAGCgt
A6_(82704220323)       CGCGGgGGGAGCGgCGGACGTGGCCGTCC-----GCGAA-------CTCCTtCgGAGCgt
A8_(83919220502)       CGCGGgGGGAGCGgCGGACGTGGCCGTCC-----GCGAA-------CTCCTtCgGAGCgt
A5_(82703220323)       CGCGGgGGGAGCGgCGGACGTGGCCGTCC-----GCGAA-------CTCCTtCgGAGCgt
```

```
Y11470.1             tCttC-AaTgcGCTGAgActC-------------------------------CcaCATaG
MW040324.1           ACGACGAncCAGCcGcAgcGCCGCTCGATtGGTAGGTTTTCTCCGGAAGACAtGcCgTCG
MW040322.1           ACGACGAGcCAGCcGcAgcGCCGCTCGATtGGnAGGTTTTCTCCGGAAGACAtGcCgTCG
MW040323.1           ACGACGAGcCAGCcGcAgcGCCGCTCGATtGGTAGGTTTTCTCCGGAAGACAtGcCgTCG
OL304250.1           ACGACGAGcCAGCcGcAgcGCCGCTCGATtGGaAGGTTTTCTCCGGAAGACAtGcCgTCG
OL304248.1           ACGACGAGcCAGCcGcAgcGCCGCTCGATtGG-AGGTTTTCTCCGGAAGACAtGcCgTCG
OL304249.1           ACGACGAGcCAGCcGcAgcGCCGCTCGATtGGTAGGTTTTCTCCGGAAGACAtGcCgTCG
Y11471.1             ACG----GTtAGtTGAAATaC------ATCGa--------------------CGaCActG
A7_(82708220323)     gCG----GTtgaCTGAAATGC------ATCGG--------------------CGGCATCG
A9_(84087220504)     gCG----GTCgGCTGAAATGC------ATCGG--------------------CGGCAcCG
A4_(82702220323)     gCG----GTCgGCTGAAATGC------ATCGG--------------------CGGCAcCG
A3_(82701220323)     gCG----GTCgGCTGAAATGC------ATCGG--------------------CGGCAcCG
A6_(82704220323)     gCG----GTCgGCTGAAATGC------ATCGG--------------------CGGCAcCG
A8_(83919220502)     gCG----GTCgGCTGAAATGC------ATCGG--------------------CGGCAcCG
A5_(82703220323)     gCG----GTCgGCTGAAATGC------ATCGG--------------------CGGCAcCG

Y11470.1             gaatat---GAaAtcA--------------------------------------------
MW040324.1           AGtaGCCTGGgGACcAactGCCCGTCCTACCAACGACGGGGGaGCGCGCAAGcCaTcCcC
MW040322.1           AGnaGCCTGGgGACcAactGCCCGTCCTACCAACGACGGGGGaGCGCGCAAGcCaTcCcC
MW040323.1           AGtaGCCTGGgGACcAactGCCCGTCCTACCAACGACGGGGGaGCGCGCAAGcnaTcCcC
OL304250.1           AGtaGCCTGGgGACcAactGCCCGTCCTACCAACGACGGGGGaGCGCGCAAGcCaTcCcC
OL304248.1           AGtaGCCTGGgGACcAactGCCCGTCCTACCAACGACGGGGGaGCGCGCAAGcCaTcCcC
OL304249.1           AGtaGCCTGGgGACcAactGCCCGTCCTACCAACGACGGGGGaGCGCGCAAGcCaTcCcC
Y11471.1             AGCGGC---aAGACGAGTtG----------------taGGGGTG-------cTCCTTCAC
A7_(82708220323)     AGCGGC---aAGACGAGTCG----------------CaGGGGTG-------GTCCTTCAC
A9_(84087220504)     AGCGGC---GAGACGAGTCG----------------CGGGGGTG-------GTCCTTCAC
A4_(82702220323)     AGCGGC---GAGACGAGTCG----------------CGGGGGTG-------GTCCTTCAC
A3_(82701220323)     AGCGGC---GAGACGAGTCG----------------CGGGGGTG-------GTCCTTCAC
A6_(82704220323)     AGCGGC---GAGACGAGTCG----------------CGGGGGTG-------GTCCTTCAC
A8_(83919220502)     AGCGGC---GAGACGAGTCG----------------CGGGGGTG-------GTCCTTCAC
A5_(82703220323)     AGCGGC---GAGACGAGTCG----------------CGGGGGTG-------GTCCTTCAC

Y11470.1             ------------------------------------------------------------
MW040324.1           CtGaCTCTCA--------------------------------------------------
MW040322.1           CtGaCTCTCA--------------------------------------------------
MW040323.1           CtGaCTCTCA--------------------------------------------------
OL304250.1           CtGaCTCTCA--------------------------------------------------
OL304248.1           CtGaCTCTCA--------------------------------------------------
OL304249.1           CtGaCTCTCA--------------------------------------------------
Y11471.1             CCGcCTtgagctcgcgggggcttacagtggtcacagtcatccgcctcagtgcgaagctag
A7_(82708220323)     CtGTt-------------------------------------------------------
A9_(84087220504)     CCGTt-------------------------------------------------------
A4_(82702220323)     CCGTt-------------------------------------------------------
A3_(82701220323)     CCGTt-------------------------------------------------------
A6_(82704220323)     CCGTt-------------------------------------------------------
A8_(83919220502)     CCGTt-------------------------------------------------------
A5_(82703220323)     CCGTt-------------------------------------------------------

Y11470.1             -----------------------------------------------
MW040324.1           -------TTTCGACCTGAGTTCAGGCAAnaCCACCCGCTCAACTTAA
MW040322.1           -------TTTCGACCTGAGTTCAGGCAAGaCCACCCGCTCAACTTAA
MW040323.1           -------TTTCGACCTGAGTTCAGGCAAGaCCACCCGCTCAACTTAA
OL304250.1           -------TTTCGACCTGAGTTCAGGCAAGaCCACCCGCTCAACTTAA
OL304248.1           -------TTTCGACCTGAGTTCAGGCAAGaCCACCCGCTCAACTTAA
OL304249.1           -------TTTCGACCTGAGTTCAGGCAAGaCCACCCGCTCAACTTAA
Y11471.1             agagggcTTTaGggtcGAccTtgtGCgctCCCACCtaCTC-------
A7_(82708220323)     ------------------------GCAAGCCCcCtCGCggggt----
A9_(84087220504)     ------------------------GCAAGCCCcCtCGCggg------
A4_(82702220323)     ------------------------GgAAGCCCc--------------
A3_(82701220323)     ------------------------GaAAGCCCcCtCGC---------
A6_(82704220323)     ------------------------GCAAGCCCcCtCGC---------
A8_(83919220502)     ------------------------GCAAGCCCcCtCGggg-------
A5_(82703220323)     ------------------------GgAAGCCCcCtCGgg--------
```
