# Supplementary material for: A Multi-Pronged Technique for Identifying Equisetum palustre and Equisetum arvense—Combining HPTLC, HPLC-ESI-MS/MS and Optimized DNA Barcoding Techniques
Source: Plants (Basel). 2022 Sep 28;11(19):2562. doi: 10.3390/plants11192562 (PMC9573248; doi:10.3390/plants11192562)
Supplement: Supplementary file 1 [file plants-11-02562-s001.zip › plants-1932149 supplementary/Horsetail Supplementary Figures/Figure S18.pdf]

```
A7_(82708220323)        -GACTCTCGGCAACGGATATCTTGGCTCTTGCAACGATGAAGAACGCAGCGAAATGCGAT
A9_(84087220504)        CGACTCTCGGCAACGGATATCTcGGCTCTcGCtACGATGAAGAACGCAGCGAAATGCGAT
A3_(82701220323)        CGACTCTCGGCAACGGATATCTTGGCTCTTGCAACGATGAAGAACGCAGCGAAATGCGAT
A6_(82704220323)        CGACTCTCGGCAACGGATATCTTGGCTCTTGCAACGATGAAGAACGCAGCGAAATGCGAT
A8_(83919220502)        --ACTCTCGGCAACGGATATCTTGGCTCTTGCAACGATGAAGAACGCAGCGAAATGCGAT
A5_(82703220323)        CGACTCTCGGCAACGGATATCTTGGCTCTTGCAACGATGAAGAACGCAGCGAAATGCGAT
A4_(82702220323)        aGACTCTCGGCAACGGATATCTTGGCTCTTGCAACGATGAAGAACGCAGCGAAATGCGAT
P10_(82716220323)       CGACTCTCGGCAACGGATATCTTGGCTCTTGCAACGATGAAGAACGCAGCGAAATGCGAT
P8_(82714220323)        CGACTCTCGGCAACGGATATCTTGGCTCTTGCAACGATGAAGAACGCAGCGAAATGCGAT
P5_(82711220323)        -GACTCTCGGCAACGGATATCTTGGCTCTTGCAACGATGAAGAACGCAGCGAAATGCGAT
P4_(82710220323)        -GACTCTCGGCAACGGATATCTTGGCTCTTGCAACGATGAAGAACGCAGCGAAATGCGAT
P9_(82715220323)        -GACTCTCGGCAACGGATATCTTGGCTCTTGCAACGATGAAGAACGCAGCGAAATGCGAT
P6_(82712220323)        CGACTCTCGGCAACGGATATCTTGGCTCTTGCAACGATGAAGAACGCAGCGAAATGCGAT
P3_(82709220323)        --ACTCTCGGCAACGGATATCTTGGCTCTTGCAACGATGAAGAACGCAGCGAAATGCGAT
P7_(82713220323)        CGACTCTCGGCAACGGATATCTTGGCTCTTGCAACGATGAAGAACGCAGCGAAATGCGAT

A7_(82708220323)        ACGTAGTGTGAATTGCAGAATTCCGCGAATCATCGAATCTTTGAACGCAACTCGCGCCCG
A9_(84087220504)        ACGTAGTGTGAATTGCAGAATTCCGCGAATCATCGAgTCTTTGAACGCAACTCGCGCCCG
A3_(82701220323)        ACGTAGTGTGAATTGCAGAATTCCGCGAATCATCGAATCTTTGAACGCAACTCGCGCCCG
A6_(82704220323)        ACGTAGTGTGAATTGCAGAATTCCGCGAATCATCGAATCTTTGAACGCAACTCGCGCCCG
A8_(83919220502)        ACGTAGTGTGAATTGCAGAATTCCGCGAATCATCGAATCTTTGAACGCAACTCGCGCCCG
A5_(82703220323)        ACGTAGTGTGAATTGCAGAATTCCGCGAATCATCGAATCTTTGAACGCAACTCGCGCCCG
A4_(82702220323)        ACGTAGTGTGAATTGCAGAATTCCGCGAATCATCGAATCTTTGAACGCAACTCGCGCCCG
P10_(82716220323)       ACGTAGTGTGAATTGCAGAATTCCGCGAATCATCGAATCTTTGAACGCAACTCGCGCCCG
P8_(82714220323)        ACGTAGTGTGAATTGCAGAATTCCGCGAATCATCGAATCTTTGAACGCAACTCGCGCCCG
P5_(82711220323)        ACGTAGTGTGAATTGCAGAATTCCGCGAATCATCGAATCTTTGAACGCAACTCGCGCCCG
P4_(82710220323)        ACGTAGTGTGAATTGCAGAATTCCGCGAATCATCGAATCTTTGAACGCAACTCGCGCCCG
P9_(82715220323)        ACGTAGTGTGAATTGCAGAATTCCGCGAATCATCGAATCTTTGAACGCAACTCGCGCCCG
P6_(82712220323)        ACGTAGTGTGAATTGCAGAATTCCGCGAATCATCGAATCTTTGAACGCAACTCGCGCCCG
P3_(82709220323)        ACGTAGTGTGAATTGCAGAATTCCGCGAATCATCGAATCTTTGAACGCAACTCGCGCCCG
P7_(82713220323)        ACGTAGTGTGAATTGCAGAATTCCGCGAATCATCGAATCTTTGAACGCAACTCGCGCCCG

A7_(82708220323)        AGGGCTCGCCtGAGGaCATGTtTGCCTGAGCGTCCTTACAAATCCTACGCCGCCTCCCCC
A9_(84087220504)        AGGGCTCGCCCGAGGGCATGTCTGCCTGAGCGTCCTTACAAATCCTACGCCGCCTCCCCC
A3_(82701220323)        AGGGCTCGCCCGAGGGCATGTCTGCCTGAGCGTCCTTACAAATCCTACGCCGCCTCCCCC
A6_(82704220323)        AGGGCTCGCCCGAGGGCATGTCTGCCTGAGCGTCCTTACAAATCCTACGCCGCCTCCCCC
A8_(83919220502)        AGGGCTCGCCCGAGGGCATGTCTGCCTGAGCGTCCTTACAAATCCTACGCCGCCTCCCCC
A5_(82703220323)        AGGGCTCGCCCGAGGGCATGTCTGCCTGAGCGTCCTTACAAATCCTACGCCGCCTCCCCC
A4_(82702220323)        AGGGCTCGCCCGAGGGCATGTCTGCCTGAGCGTCCTTACAAATCCTACGCCGCCTCCCCC
P10_(82716220323)       AGGGCTCGCCCGAGGGCATGTCTGCCTGAGCGTCCTTACAAATCCTACGCCGCCTCCCCC
P8_(82714220323)        AGGGCTCGCCCGAGGGCATGTCTGCCTGAGCGTCCTTACAAATCCTACGCCGCCTCCCCC
P5_(82711220323)        AGGGCTCGCCCGAGGGCATGTCTGCCTGAGCGTCCTTACAAATCCTACGCCGCCTCCCCC
P4_(82710220323)        AGGGCTCGCCCGAGGGCATGTCTGCCTGAGCGTCCTTACAAATCCTACGCCGCCTCCCCC
P9_(82715220323)        AGGGCTCGCCCGAGGGCATGTCTGCCTGAGCGTCCTTACAAATCCTACGCCGCCTCCCCC
P6_(82712220323)        AGGGCTCGCCCGAGGGCATGTCTGCCTGAGCGTCCTTACAAATCCTACGCCGCCTCCCCC
P3_(82709220323)        AGGGCTCGCCCGAGGGCATGTCTGCCTGAGCGTCCTTACAAATCCTACGCCGCCTCCCCC
P7_(82713220323)        AGGGCTCGCCCGAGGGCATGTCTGCCTGAGCGTCCTTACAAATCCTACGCCGCCTCCCCC

A7_(82708220323)        CaCcGGGGGAGCGGCGGAtGTGGtCGTCCGCGAACTCCTCCaGAGCaCGCGGTtGaCTGA
A9_(84087220504)        CGCGGGGGGAGCGGCGGACGTGGCCGTCCGCGAACTCCTCCGGAGCGCGCGGTCGGCTGA
A3_(82701220323)        CGCGGGGGGAGCGGCGGACGTGGCCGTCCGCGAACTCCTtCGGAGCGtGCGGTCGGCTGA
A6_(82704220323)        CGCGGGGGGAGCGGCGGACGTGGCCGTCCGCGAACTCCTtCGGAGCGtGCGGTCGGCTGA
A8_(83919220502)        CGCGGGGGGAGCGGCGGACGTGGCCGTCCGCGAACTCCTtCGGAGCGtGCGGTCGGCTGA
A5_(82703220323)        CGCGGGGGGAGCGGCGGACGTGGCCGTCCGCGAACTCCTtCGGAGCGtGCGGTCGGCTGA
A4_(82702220323)        CGCGGGGGGAGCGGCGGACGTGGCCGTCCGCGAACTCCTCCGGAGCGCGCGGTCGGCTGA
P10_(82716220323)       CGCGGGGGGAGCGGCGGACGTGGCCGTCCGCGAACTCCTCCGGAGCGCGCGGTCGGCTGA
P8_(82714220323)        CGCGGGGGGAGCGGCGGACGTGGCCGTCCGCGAACTCCTCCGGAGCGCGCGGTCGGCTGA
P5_(82711220323)        CGCGGGGGGAGCGGCGGACGTGGCCGTCCGCGAACTCCTCCGGAGCGCGCGGTCGGCTGA
P4_(82710220323)        CGCGGGGGGAGCGGCGGACGTGGCCGTCCGCGAACTCCTCCGGAGCGCGCGGTCGGCTGA
P9_(82715220323)        CGCGGGGGGAGCGGCGGACGTGGCCGTCCGCGAACTCCTCCGGAGCGCGCGGTCGGCTGA
P6_(82712220323)        CGCGGGGGGAGCGGCGGACGTGGCCGTCCGCGAACTCCTCCGGAGCGCGCGGTCGGCTGA
P3_(82709220323)        CGCGGGGGGAGCGGCGGACGTGGCCGTCCGCGAACTCCTCCGGAGCGCGCGGTCGGCTGA
P7_(82713220323)        CGCGGGGGGAGCGGCGGACGTGGCCGTCCGCGAACTCCTCCGGAGCGCGCGGTCGGCTGA
```

```
A7_(82708220323)      AATGCATCGGCGGCAtCGAGCGGCaAGACGAGTCGCaGGGGTGGTCCTTCACCtGtTGcA
A9_(84087220504)      AATGCATCGGCGGCACCGAGCGGCGAGACGAGTCGCGGGGGTGGTCCTTCACCCGtTGcA
A3_(82701220323)      AATGCATCGGCGGCACCGAGCGGCGAGACGAGTCGCGGGGGTGGTCCTTCACCCGtTGaA
A6_(82704220323)      AATGCATCGGCGGCACCGAGCGGCGAGACGAGTCGCGGGGGTGGTCCTTCACCCGtTGcA
A8_(83919220502)      AATGCATCGGCGGCACCGAGCGGCGAGACGAGTCGCGGGGGTGGTCCTTCACCCGtTGcA
A5_(82703220323)      AATGCATCGGCGGCACCGAGCGGCGAGACGAGTCGCGGGGGTGGTCCTTCACCCGtTGgA
A4_(82702220323)      AATGCATCGGCGGCACCGAGCGGCGAGACGAGTCGCGGGGGTGGTCCTTCACCCGtTGgA
P10_(82716220323)     AATGCATCGGCGGCACCGAGCGGCGAGACGAGTCGCGGGGGTGGTCCTTCACCCGCTGTA
P8_(82714220323)      AATGCATCGGCGGCACCGAGCGGCGAGACGAGTCGCGGGGGTGGTCCTTCACCCGCTGTA
P5_(82711220323)      AATGCATCGGCGGCACCGAGCGGCGAGACGAGTCGCGGGGGTGGTCCTTCACCCGCTGTA
P4_(82710220323)      AATGCATCGGCGGCACCGAGCGGCGAGACGAGTCGCGGGGGTGGTCCTTCACCCGCTGTA
P9_(82715220323)      AATGCATCGGCGGCACCGAGCGGCGAGACGAGTCGCGGGGGTGGTCCTTCACCCGCTGTA
P6_(82712220323)      AATGCATCGGCGGCACCGAGCGGCGAGACGAGTCGCGGGGGTGGTCCTTCACCCGCTGTA
P3_(82709220323)      AATGCATCGGCGGCACCGAGCGGCGAGACGAGTCGCGGGGGTGGTCCTTCACCCGCTGTA
P7_(82713220323)      AATGCATCGGCGGCACCGAGCGGCGAGACGAGTCGCGGGGGTGGTCCTTCACCCGCTGTA

A7_(82708220323)      AGCCCCCTCgCGgGGt--------------------------------------------
A9_(84087220504)      AGCCCCCTCgCGgG----------------------------------------------
A3_(82701220323)      AGCCCCCTCgC-------------------------------------------------
A6_(82704220323)      AGCCCCCTCgC-------------------------------------------------
A8_(83919220502)      AGCCCCCTCggGg-----------------------------------------------
A5_(82703220323)      AGCCCCCTCggG------------------------------------------------
A4_(82702220323)      AGCCCC------------------------------------------------------
P10_(82716220323)     AGCCCCCTCTCGCGGGGGCTTGCGGCGGTCGCGGCCGTCCGCCACGGTGCGAAGCTAGAG
P8_(82714220323)      AGCCCCCTCTCGCGGGGGCTTGCGGCGGTCGCGGCCGTCCGCCACGGTGCGAAGCTAGAG
P5_(82711220323)      AGCCCCCTCTCGCGGGGGCTTGCGGCGGTCGCGGCCGTCCGCCACGGTGCGAAGCTAGAG
P4_(82710220323)      AGCCCCCTCTCGCGGGGGCTTGCGGCGGTCGCGGCCGTCCGCCACGGTGCGAAGCTAGAG
P9_(82715220323)      AGCCCCCTCTCGCGGGGGCTTGCGGCGGTCGCGGCCGTCCGCCACGGTGCGAAGCTAGAG
P6_(82712220323)      AGCCCCCTCTCGCGGGGGCTTGCGGCGGTCGCGGCCGTCCGCCACGGTGCGAAGCTAGAG
P3_(82709220323)      AGCCCCCTCTCGCGGGGGCTTGCGGCGGTCGCGGCCGTCCGCCACGGTGCGAAGCTAGAG
P7_(82713220323)      AGCCCCCTCTCGCGGGGGCTTGCGGCGGTCGCGGCCGTCCGCCACGGTGCGAAGCTAGAG

A7_(82708220323)      ------------------------------------------------------------
A9_(84087220504)      ------------------------------------------------------------
A3_(82701220323)      ------------------------------------------------------------
A6_(82704220323)      ------------------------------------------------------------
A8_(83919220502)      ------------------------------------------------------------
A5_(82703220323)      ------------------------------------------------------------
A4_(82702220323)      ------------------------------------------------------------
P10_(82716220323)     AGGGCTTTAGGGTGCGCCTCGCGCGCTCCCTCCTACACTGGACCTCAGATCAGGCAAGGC
P8_(82714220323)      AGGGCTTTAGGGTGCGCCTCGCGCGCTCCCTCCTACACTGGACCTCAGATCAGGCAAGGC
P5_(82711220323)      AGGGCTTTAGGGTGCGCCTCGCGCGCTCCCTCCTACACTGGACCTCAGATCAGGCAAGGC
P4_(82710220323)      AGGGCTTTAGGGTGCGCCTCGCGCGCTCCCTCCTACACTGGACCTCAGATCAGGCAAGGC
P9_(82715220323)      AGGGCTTTAGGGTGCGCCTCGCGCGCTCCCTCCTACACTGGACCTCAGATCAGGCAAGGC
P6_(82712220323)      AGGGCTTTAGGGTGCGCCTCGCGCGCTCCCTCCTACACTGGACCTCAGATCAGGCAAGGC
P3_(82709220323)      AGGGCTTTAGGGTGCGCCTCGCGCGCTCCCTCCTACACTGGACCTCAGATCAGGCAAGGC
P7_(82713220323)      AGGGCTTTAGGGTGCGCCTCGCGCGCTCCCTCCTACACTGGACCTCAGATCAGGCAAGGC

A7_(82708220323)      -----------------------------------------------------
A9_(84087220504)      -----------------------------------------------------
A3_(82701220323)      -----------------------------------------------------
A6_(82704220323)      -----------------------------------------------------
A8_(83919220502)      -----------------------------------------------------
A5_(82703220323)      -----------------------------------------------------
A4_(82702220323)      -----------------------------------------------------
P10_(82716220323)     TACCCGCTGAGTTTAAGCATATAACTAAGCGGAGGAAAAGAAACCTGG-----
P8_(82714220323)      TACCCGCTGAGTTTAAGCATATAACTAAGCGGAGGAAAAGAAACCTGGTC---
P5_(82711220323)      TACCCGCTGAGTTTAAGCATATAACTAAGCGGAGGAAAAGAAACCTG------
P4_(82710220323)      TACCCGCTGAGTTTAAGCATATAACTAAGCGGAGGAAAAGAAACCTG------
P9_(82715220323)      TACCCGCTGAGTTTAAGCATATAACTAAGCGGAGGAAAAGAAACCTGGTCa--
P6_(82712220323)      TACCCGCTGAGTTTAAGCATATAACTAAGCGGAGGAAAAGAAAC---------
P3_(82709220323)      TACCCGCTGAGTTTAAGCATATAACTAAGCGGAGGAAAAGAAACtg-------
P7_(82713220323)      TACCCGCTGAGTTTAAGCATATAACTAAGCGGAGGAAAAGAAACtgGtcatag
```
