# Supplementary material for: A Multi-Pronged Technique for Identifying Equisetum palustre and Equisetum arvense—Combining HPTLC, HPLC-ESI-MS/MS and Optimized DNA Barcoding Techniques
Source: Plants (Basel). 2022 Sep 28;11(19):2562. doi: 10.3390/plants11192562 (PMC9573248; doi:10.3390/plants11192562)
Supplement: Supplementary file 1 [file plants-11-02562-s001.zip › plants-1932149 supplementary/Horsetail Supplementary Figures/Figure S19 (P1, 78075211104-1).pdf]

BLAST Results

Questions/comments

Job title: Nucleotide Sequence

**RID**	J1BFPYK9013 (Expires on 09-15 00:21 am)
**Query ID**	lcl|Query_56843
**Description**	None
**Molecule type**	dna
**Query Length**	674
**Database Name**	nt
**Description**	Nucleotide collection (nt)
**Program**	BLASTN 2.13.0+

Graphic Summary

Distribution of the top 100 Blast Hits on 100 subject sequences

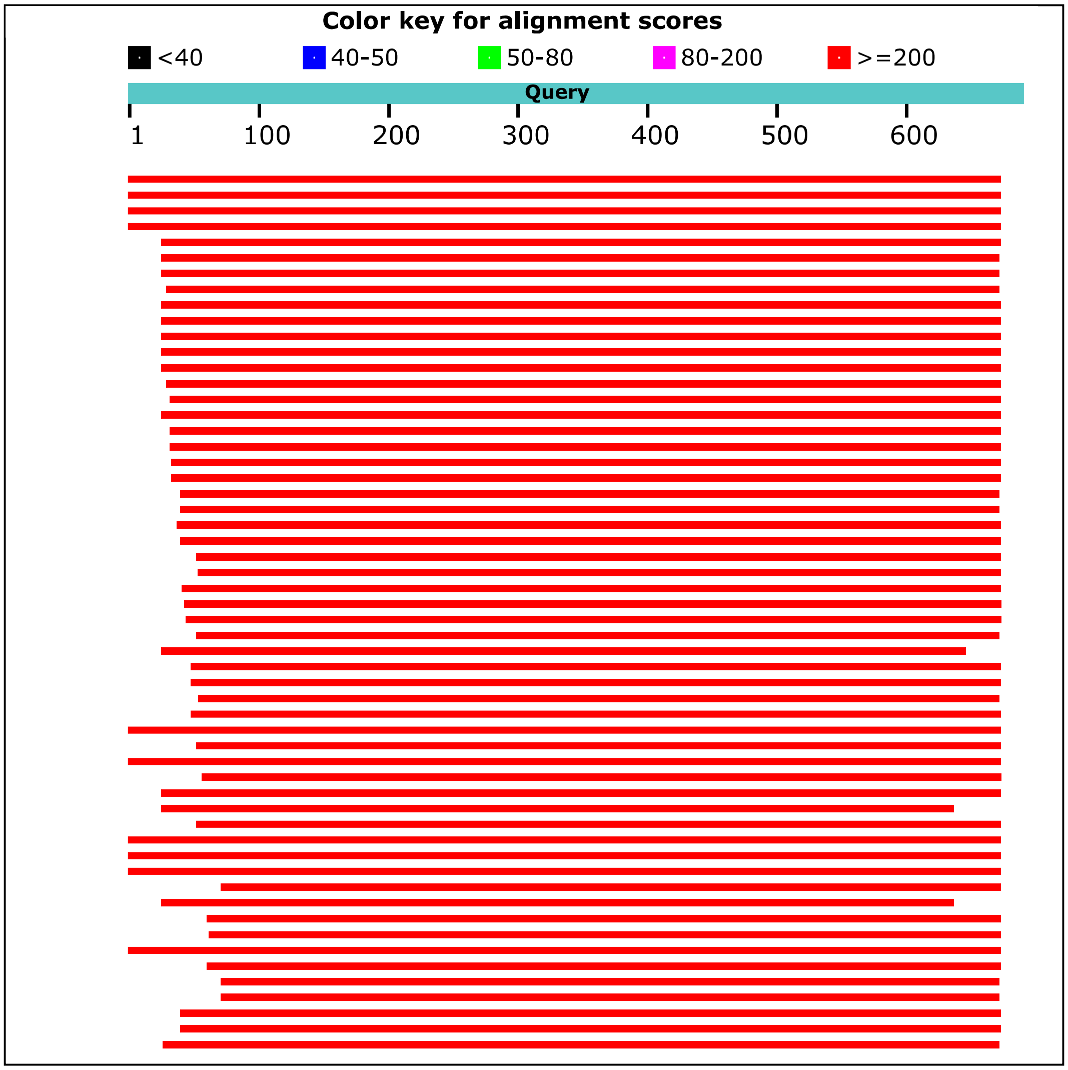

Descriptions

Sequences producing significant alignments:

Description	Max Score	Total Score	Query Cover	E value	Per. Ident	Accession
Equisetum palustre chloroplast, partial genome	1216	1216	99%	0.0	99.40%	MT984458.1
Equisetum arvense chloroplast, complete genome	1194	1194	99%	0.0	98.81%	GU191334.1
Equisetum arvense chloroplast ribulosebisphosphate carboxylase (rbcL) gene, complete cds	1194	1194	99%	0.0	98.81%	L11053.1
Equisetum arvense plastid, complete genome	1188	1188	99%	0.0	98.66%	JN968380.1
Equisetum palustre ribulose-1,5-bisphosphate carboxylase/oxygenase large subunit (rbcL) gene, partial cds; chloroplast gene for chloroplast product	1179	1179	95%	0.0	99.69%	AY226138.1
Equisetum pratense ribulose-1,5-bisphosphate carboxylase/oxygenase large subunit (rbcL) gene, partial cds; chloroplast gene for chloroplast product	1166	1166	95%	0.0	99.38%	AY226137.1
Equisetum sylvaticum ribulose-1,5-bisphosphate carboxylase/oxygenase large subunit (rbcL) gene, partial cds; chloroplast gene for chloroplast product	1166	1166	95%	0.0	99.38%	AY226136.1
Equisetum sylvaticum isolate 11640 ribulose-1,5-bisphosphate carboxylase/oxygenase large subunit (rbcL) gene, partial cds; plastid	1158	1158	94%	0.0	99.37%	MH750204.1
Equisetum environmental sample isolate EDNA16-0043490 ribulose-1,5-bisphosphate carboxylase/oxygenase large subunit (rbcL) gene, partial cds; chloroplast	1157	1157	95%	0.0	99.07%	MT197555.1
Equisetum fluviatile ribulose-1,5-bisphosphate carboxylase/oxygenase large subunit (rbcL) gene, partial cds; chloroplast gene for chloroplast product	1157	1157	95%	0.0	99.07%	AY226142.1
Equisetum arvense ribulose-1,5-bisphosphate carboxylase/oxygenase large subunit (rbcL) gene, partial cds; chloroplast gene for chloroplast product	1157	1157	95%	0.0	99.07%	AY226140.1
Equisetum telmateia subsp. braunii ribulose-1,5-bisphosphate carboxylase/oxygenase large subunit (rbcL) gene, partial cds; chloroplast gene for chloroplast product	1157	1157	95%	0.0	99.07%	AY226135.1
Equisetum environmental sample isolate EDNA16-0043491b ribulose-1,5-bisphosphate carboxylase/oxygenase large subunit (rbcL) gene, partial cds; chloroplast	1151	1151	95%	0.0	98.91%	MT197548.1
Equisetum telmateia subsp. telmateia isolate 11642 ribulose-1,5-bisphosphate carboxylase/oxygenase large subunit (rbcL) gene, partial cds; plastid	1149	1149	94%	0.0	99.06%	MH750210.1

Description	Max Score	Total Score	Query Cover	E value	Per. Ident	Accession
Equisetum arvense subsp. boreale isolate 41074 ribulose-1,5-bisphosphate carboxylase/oxygenase large subunit (rbcL) gene, partial cds; plastid	1146	1146	94%	0.0	99.06%	MH750160.1
Equisetum diffusum ribulose-1,5-bisphosphate carboxylase/oxygenase large subunit (rbcL) gene, partial cds; chloroplast gene for chloroplast product	1146	1146	95%	0.0	98.76%	AY226141.1
Equisetum x dycei isolate 26083 ribulose-1,5-bisphosphate carboxylase/oxygenase large subunit (rbcL) gene, partial cds; plastid	1144	1144	94%	0.0	99.06%	MH750220.1
Equisetum telmateia ribulose-1,5-bisphosphate carboxylase large subunit (rbcL) gene, partial cds; chloroplast gene for chloroplast product	1144	1144	94%	0.0	99.06%	AF313580.1
Equisetum fluviatile isolate 41076 ribulose-1,5-bisphosphate carboxylase/oxygenase large subunit (rbcL) gene, partial cds; plastid	1142	1142	94%	0.0	99.06%	MH750169.1
Equisetum fluviatile isolate 41075 ribulose-1,5-bisphosphate carboxylase/oxygenase large subunit (rbcL) gene, partial cds; plastid	1142	1142	94%	0.0	99.06%	MH750168.1
Equisetum sylvaticum isolate 41081 ribulose-1,5-bisphosphate carboxylase/oxygenase large subunit (rbcL) gene, partial cds; plastid	1138	1138	93%	0.0	99.36%	MH750205.1
Equisetum pratense isolate 41087 ribulose-1,5-bisphosphate carboxylase/oxygenase large subunit (rbcL) gene, partial cds; plastid	1138	1138	93%	0.0	99.36%	MH750195.1
Equisetum arvense subsp. boreale isolate 41073 ribulose-1,5-bisphosphate carboxylase/oxygenase large subunit (rbcL) gene, partial cds; plastid	1134	1134	93%	0.0	99.05%	MH750159.1
Equisetum telmateia subsp. braunii isolate 40828 ribulose-1,5-bisphosphate carboxylase/oxygenase large subunit (rbcL) gene, partial cds; plastid	1129	1129	93%	0.0	99.05%	MH750207.1
Equisetum palustre isolate 17671 ribulose-1,5-bisphosphate carboxylase/oxygenase large subunit (rbcL) gene, partial cds; plastid	1129	1129	91%	0.0	99.68%	MH750191.1
Equisetum palustre isolate 39349 ribulose-1,5-bisphosphate carboxylase/oxygenase large subunit (rbcL) gene, partial cds; plastid	1127	1127	91%	0.0	99.68%	MH750192.1
Equisetum arvense subsp. arvense isolate 41071 ribulose-1,5-bisphosphate carboxylase/oxygenase large subunit (rbcL) gene, partial cds; plastid	1127	1127	93%	0.0	99.04%	MH750158.1
Equisetum x fontqueri isolate 26093 ribulose-1,5-bisphosphate carboxylase/oxygenase large subunit (rbcL) gene, partial cds; plastid	1125	1125	93%	0.0	99.04%	MH750221.1
Equisetum x litorale isolate 41084 ribulose-1,5-bisphosphate carboxylase/oxygenase large subunit (rbcL) gene, partial cds; plastid	1122	1122	92%	0.0	99.04%	MH750222.1

Description	Max Score	Total Score	Query Cover	E value	Per. Ident	Accession
Equisetum pratense isolate 41086 ribulose-1,5-bisphosphate carboxylase/oxygenase large subunit (rbcL) gene, partial cds; plastid	1118	1118	91%	0.0	99.35%	MH750194.1
Equisetum arvense voucher JAG288 (OAC) ribulose-1,5-bisphosphate carboxylase/oxygenase large subunit (rbcL) gene, partial cds; chloroplast	1118	1118	91%	0.0	99.35%	EU677106.1
Equisetum telmateia subsp. braunii isolate 40832 ribulose-1,5-bisphosphate carboxylase/oxygenase large subunit (rbcL) gene, partial cds; plastid	1116	1116	92%	0.0	99.04%	MH750208.1
Equisetum arvense subsp. arvense isolate 26085 ribulose-1,5-bisphosphate carboxylase/oxygenase large subunit (rbcL) gene, partial cds; plastid	1116	1116	92%	0.0	99.04%	MH750156.1
Equisetum pratense isolate 39348 ribulose-1,5-bisphosphate carboxylase/oxygenase large subunit (rbcL) gene, partial cds; plastid	1114	1114	91%	0.0	99.35%	MH750193.1
Equisetum diffusum isolate 40689 ribulose-1,5-bisphosphate carboxylase/oxygenase large subunit (rbcL) gene, partial cds; plastid	1110	1110	92%	0.0	98.87%	MH750166.1
Equisetum xylochaetum chloroplast, complete genome	1110	1110	99%	0.0	96.57%	MW282958.1
Equisetum arvense subsp. arvense isolate 26084 ribulose-1,5-bisphosphate carboxylase/oxygenase large subunit (rbcL) gene, partial cds; plastid	1109	1109	91%	0.0	99.03%	MH750155.1
Equisetum hyemale chloroplast, complete genome	1105	1105	99%	0.0	96.42%	KC117177.1
Equisetum arvense subsp. arvense isolate 40833 ribulose-1,5-bisphosphate carboxylase/oxygenase large subunit (rbcL) gene, partial cds; plastid	1101	1101	91%	0.0	99.02%	MH750157.1
Equisetum scirpoides ribulose-1,5-bisphosphate carboxylase/oxygenase large subunit (rbcL) gene, partial cds; chloroplast gene for chloroplast product	1101	1101	95%	0.0	97.52%	AY226133.1
Equisetum arvense voucher AP400 (OAC) ribulose-1,5-bisphosphate carboxylase/oxygenase large subunit (rbcL) gene, partial cds; chloroplast	1099	1099	90%	0.0	99.34%	EU677105.1
Equisetum diffusum isolate 40804 ribulose-1,5-bisphosphate carboxylase/oxygenase large subunit (rbcL) gene, partial cds; plastid	1098	1098	91%	0.0	98.71%	MH750167.1
Equisetum ramosissimum isolate BPTPS123 voucher LISE:96444 chloroplast, complete genome	1094	1094	99%	0.0	96.12%	ON641349.1
Equisetum ramosissimum isolate BPTPS056 voucher LISE:96334 chloroplast, complete genome	1094	1094	99%	0.0	96.12%	NC_062377.1
Equisetum ramosissimum isolate BPTPS056 voucher LISE:96334 chloroplast, complete genome	1094	1094	99%	0.0	96.12%	OM691674.1

Description	Max Score	Total Score	Query Cover	E value	Per. Ident	Accession
Equisetum palustre chloroplast rbcL gene for ribulose-1,5-bisphosphate carboxylase/oxygenase large subunit, partial cds, specimen_voucher: TNS:776981	1094	1094	88%	0.0	99.67%	AB574686.1
Equisetum pratense voucher AP451 ribulose-1,5-bisphosphate carboxylase/oxygenase large subunit (rbcL) gene, partial cds; chloroplast	1092	1092	90%	0.0	99.01%	HQ590083.1
Equisetum fluviatile ribulose-1,5-bisphosphate carboxylase/oxygenase large subunit (rbcL) gene, partial cds; chloroplast	1092	1092	90%	0.0	99.01%	DQ463101.1
Equisetum arvense x Equisetum telmateia subsp. braunii isolate 40834 ribulose-1,5-bisphosphate carboxylase/oxygenase large subunit (rbcL) gene, partial cds; plastid	1090	1090	90%	0.0	99.01%	MH750161.1
Equisetum hyemale ribulose-1,5-bisphosphate carboxylase/oxygenase large subunit (rbcL) gene, partial cds; chloroplast	1090	1090	99%	0.0	95.97%	DQ646001.1
Equisetum arvense ribulose-1,5-bisphosphate carboxylase/oxygenase large subunit (rbcL) gene, partial cds; chloroplast	1088	1088	90%	0.0	98.85%	KM218345.1
Equisetum sylvaticum chloroplast rbcL gene for ribulose-1,5-bisphosphate carboxylase/oxygenase large subunit, partial cds, specimen_voucher: TNS:776974	1081	1081	88%	0.0	99.33%	AB574690.1
Equisetum pratense chloroplast rbcL gene for ribulose-1,5-bisphosphate carboxylase/oxygenase large subunit, partial cds, specimen_voucher: TNS:1107843	1081	1081	88%	0.0	99.33%	AB574687.1
Equisetum scirpoides isolate 41089 ribulose-1,5-bisphosphate carboxylase/oxygenase large subunit (rbcL) gene, partial cds; plastid	1079	1079	93%	0.0	97.62%	MH750203.1
Equisetum komarovii isolate 40830 ribulose-1,5-bisphosphate carboxylase/oxygenase large subunit (rbcL) gene, partial cds; plastid	1079	1079	93%	0.0	97.62%	MH750182.1
Equisetum bogotense ribulose-1,5-bisphosphate carboxylase/oxygenase large subunit (rbcL) gene, partial cds; chloroplast gene for chloroplast product	1075	1075	95%	0.0	96.88%	AY226139.1
Equisetum myriochaetum ribulose-1,5-bisphosphate carboxylase/oxygenase large subunit (rbcL) gene, partial cds; chloroplast gene for chloroplast product	1074	1074	95%	0.0	96.74%	AY226131.1
Equisetum giganteum ribulose-1,5-bisphosphate carboxylase/oxygenase large subunit (rbcL) gene, partial cds; chloroplast gene for chloroplast product	1074	1074	95%	0.0	96.74%	AY226127.1
Equisetum sp. Schuettpelz 1643 ribulose-1,5-bisphosphate carboxylase/oxygenase large subunit (rbcL) gene, partial cds; chloroplast	1072	1072	95%	0.0	96.73%	MW620286.1

Description	Max Score	Total Score	Query Cover	E value	Per. Ident	Accession
Equisetum fluviatile chloroplast rbcL gene for ribulose-1,5-bisphosphate carboxylase/oxygenase large subunit, partial cds, specimen_voucher: TNS:776384	1072	1072	88%	0.0	99.00%	AB574684.1
Equisetum laevigatum ribulose-1,5-bisphosphate carboxylase/oxygenase large subunit (rbcL) gene, partial cds; chloroplast gene for chloroplast product	1070	1070	95%	0.0	96.73%	AY226130.1
Equisetum scirpoides isolate 26090 ribulose-1,5-bisphosphate carboxylase/oxygenase large subunit (rbcL) gene, partial cds; plastid	1068	1068	93%	0.0	97.30%	MH750202.1
Equisetum hyemale subsp. affine voucher OAC 94916 ribulose-1,5-bisphosphate carboxylase/oxygenase large subunit (rbcL) gene, partial cds; chloroplast	1068	1068	95%	0.0	96.58%	KF186500.1
Equisetum hyemale subsp. affine ribulose-1,5-bisphosphate carboxylase/oxygenase large subunit (rbcL) gene, partial cds; chloroplast gene for chloroplast product	1068	1068	95%	0.0	96.58%	AY226128.1
Equisetum giganteum isolate 40806 ribulose-1,5-bisphosphate carboxylase/oxygenase large subunit (rbcL) gene, partial cds; plastid	1066	1066	94%	0.0	96.72%	MH750172.1
Equisetum giganteum isolate 26087 ribulose-1,5-bisphosphate carboxylase/oxygenase large subunit (rbcL) gene, partial cds; plastid	1066	1066	94%	0.0	96.72%	MH750170.1
Equisetum ramosissimum voucher R825 ribulose-1,5-bisphosphate carboxylase/oxygenase large subunit (rbcl) gene, partial cds; chloroplast	1066	1066	96%	0.0	96.30%	KY656709.1
Equisetum arvense chloroplast rbcL gene for ribulose-1,5-bisphosphate carboxylase/oxygenase large subunit, partial cds, specimen_voucher: TNS:763618	1066	1066	88%	0.0	98.83%	AB574683.1
Equisetum x ferrissii ribulose-1,5-bisphosphate carboxylase/oxygenase large subunit (rbcL) gene, partial cds; chloroplast	1066	1066	94%	0.0	96.72%	EU352292.1
Equisetum x ferrissii ribulose-1,5-bisphosphate carboxylase large subunit (rbcL) gene, partial cds; chloroplast gene for chloroplast product	1064	1064	94%	0.0	96.71%	AF313579.1
Equisetum scirpoides isolate 10933 ribulose-1,5-bisphosphate carboxylase/oxygenase large subunit (rbcL) gene, partial cds; plastid	1059	1059	91%	0.0	97.57%	MH750201.1
Equisetum ramosissimum subsp. debile voucher G.M. Plunkett 3642 (NYBG) ribulose-1,5-bisphosphate carboxylase/oxygenase large subunit (rbcL) gene, partial cds; chloroplast	1057	1057	95%	0.0	96.27%	MT657901.1
Equisetum ramosissimum subsp. debile ribulose-1,5-bisphosphate carboxylase/oxygenase large subunit (rbcL) gene, partial cds; chloroplast gene for chloroplast product	1057	1057	95%	0.0	96.27%	AY226132.1

Description	Max Score	Total Score	Query Cover	E value	Per. Ident	Accession
Equisetum myriochaetum isolate 40815 ribulose-1,5-bisphosphate carboxylase/oxygenase large subunit (rbcL) gene, partial cds; plastid	1053	1053	94%	0.0	96.55%	MH750185.1
Equisetum scirpoides voucher AP248 ribulose-1,5-bisphosphate carboxylase/oxygenase large subunit (rbcL) gene, partial cds; chloroplast	1050	1050	90%	0.0	97.86%	HQ590084.1
Equisetum xylochaetum isolate 40614 ribulose-1,5-bisphosphate carboxylase/oxygenase large subunit (rbcL) gene, partial cds; plastid	1046	1046	93%	0.0	96.66%	MH750228.1
Equisetum myriochaetum isolate 41080 ribulose-1,5-bisphosphate carboxylase/oxygenase large subunit (rbcL) gene, partial cds; plastid	1046	1046	93%	0.0	96.66%	MH750190.1
Equisetum myriochaetum isolate 40826 ribulose-1,5-bisphosphate carboxylase/oxygenase large subunit (rbcL) gene, partial cds; plastid	1046	1046	93%	0.0	96.66%	MH750188.1
Equisetum sylvaticum voucher TJD-502 ribulose-1,5-bisphosphate carboxylase/oxygenase large subunit (rbcL) gene, partial cds; chloroplast	1046	1046	84%	0.0	99.82%	KJ841305.1
Equisetum x schaffneri isolate 40813 ribulose-1,5-bisphosphate carboxylase/oxygenase large subunit (rbcL) gene, partial cds; plastid	1044	1044	93%	0.0	96.66%	MH750224.1
Equisetum giganteum isolate 40811 ribulose-1,5-bisphosphate carboxylase/oxygenase large subunit (rbcL) gene, partial cds; plastid	1042	1042	93%	0.0	96.65%	MH750176.1
Equisetum hyemale subsp. hyemale isolate 41088 ribulose-1,5-bisphosphate carboxylase/oxygenase large subunit (rbcL) gene, partial cds; plastid	1040	1040	93%	0.0	96.50%	MH750181.1
Equisetum variegatum ribulose-1,5-bisphosphate carboxylase/oxygenase large subunit (rbcL) gene, partial cds; chloroplast gene for chloroplast product	1040	1040	95%	0.0	95.81%	AY226134.1
Equisetum bogotense isolate 40800 ribulose-1,5-bisphosphate carboxylase/oxygenase large subunit (rbcL) gene, partial cds; plastid	1037	1037	92%	0.0	96.78%	MH750162.1
Equisetum arvense voucher TJD-508 ribulose-1,5-bisphosphate carboxylase/oxygenase large subunit (rbcL) gene, partial cds; chloroplast	1037	1037	84%	0.0	99.47%	KJ841301.1
Equisetum arvense voucher TJD-503 ribulose-1,5-bisphosphate carboxylase/oxygenase large subunit (rbcL) gene, partial cds; chloroplast	1035	1035	84%	0.0	99.47%	KJ841302.1
Equisetum giganteum isolate 40807 ribulose-1,5-bisphosphate carboxylase/oxygenase large subunit (rbcL) gene, partial cds; plastid	1033	1033	92%	0.0	96.62%	MH750173.1
Equisetum arvense voucher TJD-504 ribulose-1,5-bisphosphate carboxylase/oxygenase large subunit (rbcL) gene, partial cds; chloroplast	1033	1033	84%	0.0	99.47%	KJ841300.1

Description	Max Score	Total Score	Query Cover	E value	Per. Ident	Accession
Equisetum trachyodon isolate 41092 ribulose-1,5-bisphosphate carboxylase/oxygenase large subunit (rbcL) gene, partial cds; plastid	1029	1029	92%	0.0	96.33%	MH750227.1
Equisetum ramosissimum subsp. debile isolate 40829 ribulose-1,5-bisphosphate carboxylase/oxygenase large subunit (rbcL) gene, partial cds; plastid	1029	1029	93%	0.0	96.18%	MH750200.1
Equisetum myriochaetum isolate 40936 ribulose-1,5-bisphosphate carboxylase/oxygenase large subunit (rbcL) gene, partial cds; plastid	1029	1029	91%	0.0	96.61%	MH750189.1
Equisetum hyemale voucher AP344 (OAC) ribulose-1,5-bisphosphate carboxylase/oxygenase large subunit (rbcL) gene, partial cds; chloroplast	1029	1029	91%	0.0	96.76%	EU677107.1
Equisetum hyemale subsp. affine isolate 40831 ribulose-1,5-bisphosphate carboxylase/oxygenase large subunit (rbcL) gene, partial cds; plastid	1027	1027	92%	0.0	96.46%	MH750177.1
Equisetum sylvaticum plastid partial rbcL gene for ribulose-1,5-biphosphate carboxylase/oxygenase large subunit, specimen voucher H823764	1027	1027	82%	0.0	99.82%	HE574610.1
Equisetum ramosissimum subsp. debile isolate 36802 ribulose-1,5-bisphosphate carboxylase/oxygenase large subunit (rbcL) gene, partial cds; plastid	1026	1026	93%	0.0	96.03%	MH750199.1
Commelina diffusa voucher Q555 ribulose-1,5-bisphosphate carboxylase/oxygenase large subunit (rbcL) gene, partial cds; chloroplast	1026	1026	84%	0.0	99.29%	MH714073.1
Equisetum myriochaetum isolate 26088 ribulose-1,5-bisphosphate carboxylase/oxygenase large subunit (rbcL) gene, partial cds; plastid	1022	1022	91%	0.0	96.59%	MH750184.1
Equisetum scirpoides chloroplast rbcL gene for ribulose-1,5-bisphosphate carboxylase/oxygenase large subunit, partial cds, specimen_voucher: TNS:768168	1022	1022	88%	0.0	97.49%	AB574689.1
Equisetum palustre voucher Chase 11641 (K) ribulose-1,5-bisphosphate carboxylase/oxygenase large subunit (rbcL) gene, partial cds; chloroplast	1022	1022	82%	0.0	100.00%	GQ248601.1
Equisetum laevigatum isolate 40812 ribulose-1,5-bisphosphate carboxylase/oxygenase large subunit (rbcL) gene, partial cds; plastid	1020	1020	91%	0.0	96.59%	MH750183.1

Alignments

Equisetum palustre chloroplast, partial genome

Sequence ID: **MT984458.1** Length: 5501 Number of Matches: 1
Range 1: 2213 to 2882

Score	Expect	Identities	Gaps	Strand	Frame
1216 bits(658)	0.0()	666/670(99%)	0/670(0%)	Plus/Plus	

Features:

```
Query  1     ATGTCACCACAAACAGAGACTAAAGCAGGTGTTGGATTTAAAGCTGGTGTTAAAGATTAT  60
             |||||||||||||| ||||| |||||||||||||||||||||||||||||||||||||||
Sbjct  2213  ATGTCACCACAAACGGAGACCAAAGCAGGTGTTGGATTTAAAGCTGGTGTTAAAGATTAT  2272

Query  61    CGATTGACTTATTTTACTCCAGATTATGAAACCAAAGATACCGATATTTTAGCAGCATTC  120
             ||||||||||||||||||||||||||||||||||||||||||||||||||||||||||||
Sbjct  2273  CGATTGACTTATTTTACTCCAGATTATGAAACCAAAGATACCGATATTTTAGCAGCATTC  2332

Query  121   CGTATGACTCCTCAACCGGGAGTACCACCGGAAGAAGCAGGAGCAGCCGTAGCTGCTGAA  180
             ||||||||||||||||||||||||||||||||||||||||||||||||||||||||||||
Sbjct  2333  CGTATGACTCCTCAACCGGGAGTACCACCGGAAGAAGCAGGAGCAGCCGTAGCTGCTGAA  2392

Query  181   TCGTCCACGGGCACCTGGACTACCGTATGGACAGATGGACTTACTAGTCTTGATCGATAT  240
             ||||||||||||||||||||||||||||||||||||||||||||||||||||||||||||
Sbjct  2393  TCGTCCACGGGCACCTGGACTACCGTATGGACAGATGGACTTACTAGTCTTGATCGATAT  2452

Query  241   AAAGGTCGATGCTATAATATTGAACCTGTTGCTGGAGAAGATAATCAATTCATAGCTTAT  300
             ||||||||||||||||||||||||||||||||||||||||||||||||||||||||||||
Sbjct  2453  AAAGGTCGATGCTATAATATTGAACCTGTTGCTGGAGAAGATAATCAATTCATAGCTTAT  2512

Query  301   GTAGCCTATCCTTTAGATCTTTTTGAAGAAGGTTCTGTTACCAATCTTTTTACTTCAATT  360
             ||||||||||||||||||||||||||||||||||||||||||||||||||||||||||||
Sbjct  2513  GTAGCCTATCCTTTAGATCTTTTTGAAGAAGGTTCTGTTACCAATCTTTTTACTTCAATT  2572

Query  361   GTTGGTAATGTTTTCGGCTTCAAAGCTCTACGTGCTTTACGTTTAGAAGATTTAAGAATT  420
             ||||||||||||||||||||||||||||||||||||||||||||||||||||||||||||
Sbjct  2573  GTTGGTAATGTTTTCGGCTTCAAAGCTCTACGTGCTTTACGTTTAGAAGATTTAAGAATT  2632

Query  421   CCTCCTGCTTACTCCAAAACTTTTATAGGACCACCCCACGGTATCCAGGTTGAAAGAGAT  480
             ||||||||||||||||||||||||||||||||||||||||||||||||||||||||||||
Sbjct  2633  CCTCCTGCTTACTCCAAAACTTTTATAGGACCACCCCACGGTATCCAGGTTGAAAGAGAT  2692

Query  481   AAGTTAAACAAATATGGTCGTCCTTTATTAGGTTGTACAATTAAACCAAAATTGGGACTA  540
             ||||||||||||||||||||||||||||||||||||||||||||||||||||||||||||
Sbjct  2693  AAGTTAAACAAATATGGTCGTCCTTTATTAGGTTGTACAATTAAACCAAAATTGGGACTA  2752

Query  541   TCTGCTAAAAACTATGGTAGGGCTGTTTATGAATGTCTTCGTGGTGGACTTGATTTCACC  600
             ||||||||||||||||||||||||||||||||||||||||||||||||||||||||||||
Sbjct  2753  TCTGCTAAAAACTATGGTAGGGCTGTTTATGAATGTCTTCGTGGTGGACTTGATTTCACC  2812

Query  601   AAAGATGATGAGAATGTAAACTCTCAACCCTTTATGCGTTGGAGAGATCGATTCTTATTT  660
             |||||||||||||||||||||||||||||||||||||||||||||||||| |||||||| 
Sbjct  2813  AAAGATGATGAGAATGTAAACTCTCAACCCTTTATGCGTTGGAGAGATCGTTTCTTATTC  2872

Query  661   GTAGCAGAAG  670
             ||||||||||
Sbjct  2873  GTAGCAGAAG  2882
```

Equisetum arvense chloroplast, complete genome

Sequence ID: **GU191334.1** Length: 133309 Number of Matches: 1

Range 1: 27302 to 27971

Score	Expect	Identities	Gaps	Strand	Frame
1194 bits(646)	0.0()	662/670(99%)	0/670(0%)	Plus/Minus	

Features:

```
Query  1      ATGTCACCACAAACAGAGACTAAAGCAGGTGTTGGATTTAAAGCTGGTGTTAAAGATTAT  60
              |||||||||||||| ||||| |||||||||||||||||||||||||||||||||||||||
Sbjct  27971  ATGTCACCACAAACGGAGACCAAAGCAGGTGTTGGATTTAAAGCTGGTGTTAAAGATTAT  27912

Query  61     CGATTGACTTATTTTACTCCAGATTATGAAACCAAAGATACCGATATTTTAGCAGCATTC  120
              ||||||||||||||||||||||||||||||||||||||||||||||||||||||||||||
Sbjct  27911  CGATTGACTTATTTTACTCCAGATTATGAAACCAAAGATACCGATATTTTAGCAGCATTC  27852

Query  121    CGTATGACTCCTCAACCGGGAGTACCACCGGAAGAAGCAGGAGCAGCCGTAGCTGCTGAA  180
              ||||||||||||||||||||||||||||||||||||||||||||||||||||||||||||
Sbjct  27851  CGTATGACTCCTCAACCGGGAGTACCACCGGAAGAAGCAGGAGCAGCCGTAGCTGCTGAA  27792

Query  181    TCGTCCACGGGCACCTGGACTACCGTATGGACAGATGGACTTACTAGTCTTGATCGATAT  240
              ||||||||||||||||||||||||||||||||||||||||||||||||||||||||||||
Sbjct  27791  TCGTCCACGGGCACCTGGACTACCGTATGGACAGATGGACTTACTAGTCTTGATCGATAT  27732

Query  241    AAAGGTCGATGCTATAATATTGAACCTGTTGCTGGAGAAGATAATCAATTCATAGCTTAT  300
              ||||||||||||||||||||||||||||||||||||||||||||||||||||||||||||
Sbjct  27731  AAAGGTCGATGCTATAATATTGAACCTGTTGCTGGAGAAGATAATCAATTCATAGCTTAT  27672

Query  301    GTAGCCTATCCTTTAGATCTTTTTGAAGAAGGTTCTGTTACCAATCTTTTTACTTCAATT  360
              |||||||| |||||||||||||||||||||||||||||||||||||||||||||||||||
Sbjct  27671  GTAGCCTACCCTTTAGATCTTTTTGAAGAAGGTTCTGTTACCAATCTTTTTACTTCAATT  27612

Query  361    GTTGGTAATGTTTTCGGCTTCAAAGCTCTACGTGCTTTACGTTTAGAAGATTTAAGAATT  420
              ||||||||||||||||||||||||||||||||||||||||||||||||||||||||||||
Sbjct  27611  GTTGGTAATGTTTTCGGCTTCAAAGCTCTACGTGCTTTACGTTTAGAAGATTTAAGAATT  27552
```

```
Query  421    CCTCCTGCTTACTCCAAAACTTTTATAGGACCACCCCACGGTATCCAGGTTGAAAGAGAT  480
              ||||||||||||||||||||||||||||||||||||||||||||||||||||||||||||
Sbjct  27551  CCTCCTGCTTACTCCAAAACTTTTATAGGACCACCCCACGGTATCCAGGTTGAAAGAGAT  27492

Query  481    AAGTTAAACAAATATGGTCGTCCTTTATTAGGTTGTACAATTAAACCAAAATTGGGACTA  540
              ||||||||||||| ||||||||||||||||||||||||||||||||||||||||||||||
Sbjct  27491  AAGTTAAACAAATACGGTCGTCCTTTATTAGGTTGTACAATTAAACCAAAATTGGGACTA  27432

Query  541    TCTGCTAAAAACTATGGTAGGGCTGTTTATGAATGTCTTCGTGGTGGACTTGATTTCACC  600
              |||||||||||||||||||| |||||||||||||||||||||||||| ||||||||||||
Sbjct  27431  TCTGCTAAAAACTATGGTAGAGCTGTTTATGAATGTCTTCGTGGTGGGCTTGATTTCACC  27372

Query  601    AAAGATGATGAGAATGTAAACTCTCAACCCTTTATGCGTTGGAGAGATCGATTCTTATTT  660
              |||||||||||||||||||||||||||||||||||||||||||||||||| ||||||||
Sbjct  27371  AAAGATGATGAGAATGTAAACTCTCAACCCTTTATGCGTTGGAGAGATCGTTTCTTATTC  27312

Query  661    GTAGCAGAAG  670
              ||||||||||
Sbjct  27311  GTAGCAGAAG  27302
```

Equisetum arvense chloroplast ribulosebisphosphate carboxylase (rbcL) gene, complete cds

Sequence ID: **L11053.1** Length: 1587 Number of Matches: 1

Range 1: 73 to 742

Score	Expect	Identities	Gaps	Strand	Frame
1194 bits(646)	0.0()	662/670(99%)	0/670(0%)	Plus/Plus	

Features:

```
Query  1    ATGTCACCACAAACAGAGACTAAAGCAGGTGTTGGATTTAAAGCTGGTGTTAAAGATTAT  60
            |||||||||||||| ||||| |||||||||||||||||||||||||||||||||||||||
Sbjct  73   ATGTCACCACAAACGGAGACCAAAGCAGGTGTTGGATTTAAAGCTGGTGTTAAAGATTAT  132

Query  61   CGATTGACTTATTTTACTCCAGATTATGAAACCAAAGATACCGATATTTTAGCAGCATTC  120
            ||||||||||||||||||||||||||||||||||||||||||||||||||||||||||||
Sbjct  133  CGATTGACTTATTTTACTCCAGATTATGAAACCAAAGATACCGATATTTTAGCAGCATTC  192

Query  121  CGTATGACTCCTCAACCGGGAGTACCACCGGAAGAAGCAGGAGCAGCCGTAGCTGCTGAA  180
            ||||||||||||||||||||||||||||||||||||||||||||||||||||||||||||
Sbjct  193  CGTATGACTCCTCAACCGGGAGTACCACCGGAAGAAGCAGGAGCAGCCGTAGCTGCTGAA  252

Query  181  TCGTCCACGGGCACCTGGACTACCGTATGGACAGATGGACTTACTAGTCTTGATCGATAT  240
            ||||||||||||||||||||||||||||||||||||||||||||||||||||||||||||
Sbjct  253  TCGTCCACGGGCACCTGGACTACCGTATGGACAGATGGACTTACTAGTCTTGATCGATAT  312

Query  241  AAAGGTCGATGCTATAATATTGAACCTGTTGCTGGAGAAGATAATCAATTCATAGCTTAT  300
            ||||||||||||||||||||||||||||||||||||||||||||||||||||||||||||
Sbjct  313  AAAGGTCGATGCTATAATATTGAACCTGTTGCTGGAGAAGATAATCAATTCATAGCTTAT  372

Query  301  GTAGCCTATCCTTTAGATCTTTTTGAAGAAGGTTCTGTTACCAATCTTTTTACTTCAATT  360
            |||||||| |||||||||||||||||||||||||||||||||||||||||||||||||||
Sbjct  373  GTAGCCTACCCTTTAGATCTTTTTGAAGAAGGTTCTGTTACCAATCTTTTTACTTCAATT  432

Query  361  GTTGGTAATGTTTTCGGCTTCAAAGCTCTACGTGCTTTACGTTTAGAAGATTTAAGAATT  420
            ||||||||||||||||||||||||||||||||||||||||||||||||||||||||||||
Sbjct  433  GTTGGTAATGTTTTCGGCTTCAAAGCTCTACGTGCTTTACGTTTAGAAGATTTAAGAATT  492

Query  421  CCTCCTGCTTACTCCAAAACTTTTATAGGACCACCCCACGGTATCCAGGTTGAAAGAGAT  480
            ||||||||||||||||||||||||||||||||||||||||||||||||||||||||||||
Sbjct  493  CCTCCTGCTTACTCCAAAACTTTTATAGGACCACCCCACGGTATCCAGGTTGAAAGAGAT  552

Query  481  AAGTTAAACAAATATGGTCGTCCTTTATTAGGTTGTACAATTAAACCAAAATTGGGACTA  540
            ||||||||||||| ||||||||||||||||||||||||||||||||||||||||||||||
Sbjct  553  AAGTTAAACAAATACGGTCGTCCTTTATTAGGTTGTACAATTAAACCAAAATTGGGACTA  612

Query  541  TCTGCTAAAAACTATGGTAGGGCTGTTTATGAATGTCTTCGTGGTGGACTTGATTTCACC  600
            |||||||||||||||||||| |||||||||||||||||||||||||| ||||||||||||
Sbjct  613  TCTGCTAAAAACTATGGTAGAGCTGTTTATGAATGTCTTCGTGGTGGGCTTGATTTCACC  672

Query  601  AAAGATGATGAGAATGTAAACTCTCAACCCTTTATGCGTTGGAGAGATCGATTCTTATTT  660
            |||||||||||||||||||||||||||||||||||||||||||||||||| ||||||||
Sbjct  673  AAAGATGATGAGAATGTAAACTCTCAACCCTTTATGCGTTGGAGAGATCGTTTCTTATTC  732

Query  661  GTAGCAGAAG  670
            ||||||||||
Sbjct  733  GTAGCAGAAG  742
```

Equisetum arvense plastid, complete genome

Sequence ID: **JN968380.1** Length: 132726 Number of Matches: 1

Range 1: 64958 to 65627

Score	Expect	Identities	Gaps	Strand	Frame

1188 bits(643)	0.0()	661/670(99%)	0/670(0%)	Plus/Plus

Features:

```
Query  1      ATGTCACCACAAACAGAGACTAAAGCAGGTGTTGGATTTAAAGCTGGTGTTAAAGATTAT  60
              |||||||||||||| ||||| |||||||||||||||||||||||||||||||||||||||
Sbjct  64958  ATGTCACCACAAACGGAGACCAAAGCAGGTGTTGGATTTAAAGCTGGTGTTAAAGATTAT  65017

Query  61     CGATTGACTTATTTTACTCCAGATTATGAAACCAAAGATACCGATATTTTAGCAGCATTC  120
              ||||||||||||||||||||||||||||||||||||||||||||||||||||||||||||
Sbjct  65018  CGATTGACTTATTTTACTCCAGATTATGAAACCAAAGATACCGATATTTTAGCAGCATTC  65077

Query  121    CGTATGACTCCTCAACCGGGAGTACCACCGGAAGAAGCAGGAGCAGCCGTAGCTGCTGAA  180
              ||||||||||||||||||||||||||||||||||||||||||||||||||||||||||||
Sbjct  65078  CGTATGACTCCTCAACCGGGAGTACCACCGGAAGAAGCAGGAGCAGCCGTAGCTGCTGAA  65137

Query  181    TCGTCCACGGGCACCTGGACTACCGTATGGACAGATGGACTTACTAGTCTTGATCGATAT  240
              ||||||||||||||||||||||||||||||||||||||||||||||||||||||||||||
Sbjct  65138  TCGTCCACGGGCACCTGGACTACCGTATGGACAGATGGACTTACTAGTCTTGATCGATAT  65197

Query  241    AAAGGTCGATGCTATAATATTGAACCTGTTGCTGGAGAAGATAATCAATTCATAGCTTAT  300
              ||||||||||||||||||||||||||||||||||||||||||||||||||||||||||||
Sbjct  65198  AAAGGTCGATGCTATAATATTGAACCTGTTGCTGGAGAAGATAATCAATTCATAGCTTAT  65257

Query  301    GTAGCCTATCCTTTAGATCTTTTTGAAGAAGGTTCTGTTACCAATCTTTTTACTTCAATT  360
              |||||||| |||||||||||||||||||||||||||||||||||||||||||||||||||
Sbjct  65258  GTAGCCTACCCTTTAGATCTTTTTGAAGAAGGTTCTGTTACCAATCTTTTTACTTCAATT  65317

Query  361    GTTGGTAATGTTTTCGGCTTCAAAGCTCTACGTGCTTTACGTTTAGAAGATTTAAGAATT  420
              ||||||||||||||||| ||||||||||||||||||||||||||||||||||||||||||
Sbjct  65318  GTTGGTAATGTTTTCGGATTCAAAGCTCTACGTGCTTTACGTTTAGAAGATTTAAGAATT  65377

Query  421    CCTCCTGCTTACTCCAAAACTTTTATAGGACCACCCCACGGTATCCAGGTTGAAAGAGAT  480
              ||||||||||||||||||||||||||||||||||||||||||||||||||||||||||||
Sbjct  65378  CCTCCTGCTTACTCCAAAACTTTTATAGGACCACCCCACGGTATCCAGGTTGAAAGAGAT  65437

Query  481    AAGTTAAACAATATGGTCGTCCTTTATTAGGTTGTACAATTAAACCAAAATTGGGACTA  540
              ||||||||||||| ||||||||||||||||||||||||||||||||||||||||||||||
Sbjct  65438  AAGTTAAACAATACGGTCGTCCTTTATTAGGTTGTACAATTAAACCAAAATTGGGACTA  65497

Query  541    TCTGCTAAAAACTATGGTAGGGCTGTTTATGAATGTCTTCGTGGTGGACTTGATTTCACC  600
              |||||||||||||||||||| |||||||||||||||||||||||||| ||||||||||||
Sbjct  65498  TCTGCTAAAAACTATGGTAGAGCTGTTTATGAATGTCTTCGTGGTGGGCTTGATTTCACC  65557

Query  601    AAAGATGATGAGAATGTAAACTCTCAACCCTTTATGCGTTGGAGAGATCGATTCTTATTT  660
              |||||||||||||||||||||||||||||||||||||||||||||||||| ||||||||
Sbjct  65558  AAAGATGATGAGAATGTAAACTCTCAACCCTTTATGCGTTGGAGAGATCGTTTCTTATTC  65617

Query  661    GTAGCAGAAG  670
              ||||||||||
Sbjct  65618  GTAGCAGAAG  65627
```

Equisetum palustre ribulose-1,5-bisphosphate carboxylase/oxygenase large subunit (rbcL) gene, partial cds; chloroplast gene for chloroplast product

Sequence ID: **AY226138.1** Length: 1325 Number of Matches: 1

Range 1: 1 to 644

Score	Expect	Identities	Gaps	Strand	Frame
1179 bits(638)	0.0()	642/644(99%)	0/644(0%)	Plus/Plus	

Features:

```
Query  27     AGGTGTTGGATTTAAAGCTGGTGTTAAAGATTATCGATTGACTTATTTTACTCCAGATTA  86
              ||||||||||||||||||||||||||||||||||||||||||||||||||||||||||||
Sbjct  1      AGGTGTTGGATTTAAAGCTGGTGTTAAAGATTATCGATTGACTTATTTTACTCCAGATTA  60

Query  87     TGAAACCAAAGATACCGATATTTTAGCAGCATTCCGTATGACTCCTCAACCGGGAGTACC  146
              ||||||||||||||||||||||||||||||||||||||||||||||||||||||||||||
Sbjct  61     TGAAACCAAAGATACCGATATTTTAGCAGCATTCCGTATGACTCCTCAACCGGGAGTACC  120

Query  147    ACCGGAAGAAGCAGGAGCAGCCGTAGCTGCTGAATCGTCCACGGGCACCTGGACTACCGT  206
              ||||||||||||||||||||||||||||||||||||||||||||||||||||||||||||
Sbjct  121    ACCGGAAGAAGCAGGAGCAGCCGTAGCTGCTGAATCGTCCACGGGCACCTGGACTACCGT  180

Query  207    ATGGACAGATGGACTTACTAGTCTTGATCGATATAAAGGTCGATGCTATAATATTGAACC  266
              ||||||||||||||||||||||||||||||||||||||||||||||||||||||||||||
Sbjct  181    ATGGACAGATGGACTTACTAGTCTTGATCGATATAAAGGTCGATGCTATAATATTGAACC  240

Query  267    TGTTGCTGGAGAAGATAATCAATTCATAGCTTATGTAGCCTATCCTTTAGATCTTTTTGA  326
              ||||||||||||||||||||||||||||||||||||||||||||||||||||||||||||
Sbjct  241    TGTTGCTGGAGAAGATAATCAATTCATAGCTTATGTAGCCTATCCTTTAGATCTTTTTGA  300

Query  327    AGAAGGTTCTGTTACCAATCTTTTTACTTCAATTGTTGGTAATGTTTTCGGCTTCAAAGC  386
              ||||||||||||||||||||||||||||||||||||||||||||||||||||||||||||
Sbjct  301    AGAAGGTTCTGTTACCAATCTTTTTACTTCAATTGTTGGTAATGTTTTCGGCTTCAAAGC  360
```

```
Query  387   TCTACGTGCTTTACGTTTAGAAGATTTAAGAATTCCTCCTGCTTACTCCAAAACTTTTAT  446
             ||||||||||||||||||||||||||||||||||||||||||||||||||||||||||||
Sbjct  361   TCTACGTGCTTTACGTTTAGAAGATTTAAGAATTCCTCCTGCTTACTCCAAAACTTTTAT  420

Query  447   AGGACCACCCCACGGTATCCAGGTTGAAAGAGATAAGTTAAACAAATATGGTCGTCCTTT  506
             ||||||||||||||||||||||||||||||||||||||||||||||||||||||||||||
Sbjct  421   AGGACCACCCCACGGTATCCAGGTTGAAAGAGATAAGTTAAACAAATATGGTCGTCCTTT  480

Query  507   ATTAGGTTGTACAATTAAACCAAAATTGGGACTATCTGCTAAAAACTATGGTAGGGCTGT  566
             ||||||||||||||||||||||||||||||||||||||||||||||||||||||||||||
Sbjct  481   ATTAGGTTGTACAATTAAACCAAAATTGGGACTATCTGCTAAAAACTATGGTAGGGCTGT  540

Query  567   TTATGAATGTCTTCGTGGTGGACTTGATTTCACCAAAGATGATGAGAATGTAAACTCTCA  626
             ||||||||||||||||||||||||||||||||||||||||||||||||||||||||||||
Sbjct  541   TTATGAATGTCTTCGTGGTGGACTTGATTTCACCAAAGATGATGAGAATGTAAACTCTCA  600

Query  627   ACCCTTTATGCGTTGGAGAGATCGATTCTTATTTGTAGCAGAAG  670
             |||||||||||||||||||||||| |||||||| ||||||||||
Sbjct  601   ACCCTTTATGCGTTGGAGAGATCGTTTCTTATTCGTAGCAGAAG  644
```


BLAST is a registered trademark of the National Library of Medicine

Support center Mailing list YouTube

- National Library Of Medicine
- National Institutes Of Health
- U.S. Department of Health & Human Services
- USA.gov

NCBI

National Center for Biotechnology Information, U.S. National Library of Medicine 8600 Rockville Pike, Bethesda MD, 20894 USA

Policies and Guidelines | Contact

HHS Vulnerability Disclosure
