# Supplementary material for: A Multi-Pronged Technique for Identifying Equisetum palustre and Equisetum arvense—Combining HPTLC, HPLC-ESI-MS/MS and Optimized DNA Barcoding Techniques
Source: Plants (Basel). 2022 Sep 28;11(19):2562. doi: 10.3390/plants11192562 (PMC9573248; doi:10.3390/plants11192562)
Supplement: Supplementary file 1 [file plants-11-02562-s001.zip › plants-1932149 supplementary/Horsetail Supplementary Figures/Figure S20 (P2, 75890210901-1).pdf]

BLAST Results

Questions/comments

Job title: Nucleotide Sequence

**RID**	J1BT2DRF013 (Expires on 09-15 00:26 am)
**Query ID**	lcl|Query_19371
**Description**	None
**Molecule type**	dna
**Query Length**	669
**Database Name**	nt
**Description**	Nucleotide collection (nt)
**Program**	BLASTN 2.13.0+

Graphic Summary

Distribution of the top 100 Blast Hits on 100 subject sequences

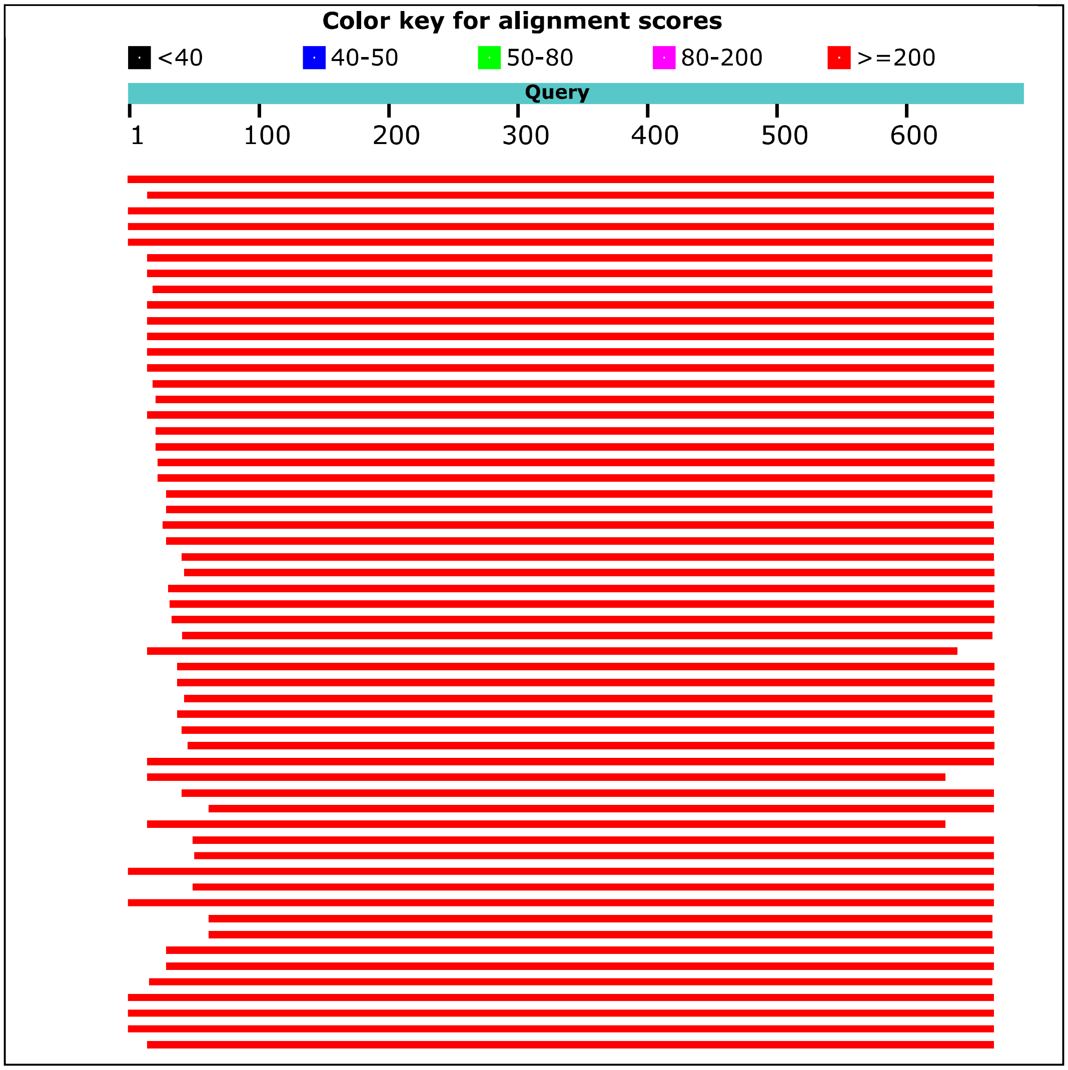

Descriptions

Sequences producing significant alignments:

Description	Max Score	Total Score	Query Cover	E value	Per. Ident	Accession
Equisetum palustre chloroplast, partial genome	1195	1195	98%	0.0	99.39%	MT984458.1
Equisetum palustre ribulose-1,5-bisphosphate carboxylase/oxygenase large subunit (rbcL) gene, partial cds; chloroplast gene for chloroplast product	1179	1179	96%	0.0	99.69%	AY226138.1
Equisetum arvense chloroplast, complete genome	1173	1173	98%	0.0	98.79%	GU191334.1
Equisetum arvense chloroplast ribulosebisphosphate carboxylase (rbcL) gene, complete cds	1173	1173	98%	0.0	98.79%	L11053.1
Equisetum arvense plastid, complete genome	1168	1168	98%	0.0	98.63%	JN968380.1
Equisetum pratense ribulose-1,5-bisphosphate carboxylase/oxygenase large subunit (rbcL) gene, partial cds; chloroplast gene for chloroplast product	1166	1166	96%	0.0	99.38%	AY226137.1
Equisetum sylvaticum ribulose-1,5-bisphosphate carboxylase/oxygenase large subunit (rbcL) gene, partial cds; chloroplast gene for chloroplast product	1166	1166	96%	0.0	99.38%	AY226136.1
Equisetum sylvaticum isolate 11640 ribulose-1,5-bisphosphate carboxylase/oxygenase large subunit (rbcL) gene, partial cds; plastid	1158	1158	95%	0.0	99.37%	MH750204.1
Equisetum environmental sample isolate EDNA16-0043490 ribulose-1,5-bisphosphate carboxylase/oxygenase large subunit (rbcL) gene, partial cds; chloroplast	1157	1157	96%	0.0	99.07%	MT197555.1
Equisetum fluviatile ribulose-1,5-bisphosphate carboxylase/oxygenase large subunit (rbcL) gene, partial cds; chloroplast gene for chloroplast product	1157	1157	96%	0.0	99.07%	AY226142.1
Equisetum arvense ribulose-1,5-bisphosphate carboxylase/oxygenase large subunit (rbcL) gene, partial cds; chloroplast gene for chloroplast product	1157	1157	96%	0.0	99.07%	AY226140.1
Equisetum telmateia subsp. braunii ribulose-1,5-bisphosphate carboxylase/oxygenase large subunit (rbcL) gene, partial cds; chloroplast gene for chloroplast product	1157	1157	96%	0.0	99.07%	AY226135.1
Equisetum environmental sample isolate EDNA16-0043491b ribulose-1,5-bisphosphate carboxylase/oxygenase large subunit (rbcL) gene, partial cds; chloroplast	1151	1151	96%	0.0	98.91%	MT197548.1
Equisetum telmateia subsp. telmateia isolate 11642 ribulose-1,5-bisphosphate carboxylase/oxygenase large subunit (rbcL) gene, partial cds; plastid	1149	1149	95%	0.0	99.06%	MH750210.1

Description	Max Score	Total Score	Query Cover	E value	Per. Ident	Accession
Equisetum arvense subsp. boreale isolate 41074 ribulose-1,5-bisphosphate carboxylase/oxygenase large subunit (rbcL) gene, partial cds; plastid	1146	1146	95%	0.0	99.06%	MH750160.1
Equisetum diffusum ribulose-1,5-bisphosphate carboxylase/oxygenase large subunit (rbcL) gene, partial cds; chloroplast gene for chloroplast product	1146	1146	96%	0.0	98.76%	AY226141.1
Equisetum x dycei isolate 26083 ribulose-1,5-bisphosphate carboxylase/oxygenase large subunit (rbcL) gene, partial cds; plastid	1144	1144	95%	0.0	99.06%	MH750220.1
Equisetum telmateia ribulose-1,5-bisphosphate carboxylase large subunit (rbcL) gene, partial cds; chloroplast gene for chloroplast product	1144	1144	95%	0.0	99.06%	AF313580.1
Equisetum fluviatile isolate 41076 ribulose-1,5-bisphosphate carboxylase/oxygenase large subunit (rbcL) gene, partial cds; plastid	1142	1142	95%	0.0	99.06%	MH750169.1
Equisetum fluviatile isolate 41075 ribulose-1,5-bisphosphate carboxylase/oxygenase large subunit (rbcL) gene, partial cds; plastid	1142	1142	95%	0.0	99.06%	MH750168.1
Equisetum sylvaticum isolate 41081 ribulose-1,5-bisphosphate carboxylase/oxygenase large subunit (rbcL) gene, partial cds; plastid	1138	1138	93%	0.0	99.36%	MH750205.1
Equisetum pratense isolate 41087 ribulose-1,5-bisphosphate carboxylase/oxygenase large subunit (rbcL) gene, partial cds; plastid	1138	1138	93%	0.0	99.36%	MH750195.1
Equisetum arvense subsp. boreale isolate 41073 ribulose-1,5-bisphosphate carboxylase/oxygenase large subunit (rbcL) gene, partial cds; plastid	1134	1134	94%	0.0	99.05%	MH750159.1
Equisetum telmateia subsp. braunii isolate 40828 ribulose-1,5-bisphosphate carboxylase/oxygenase large subunit (rbcL) gene, partial cds; plastid	1129	1129	94%	0.0	99.05%	MH750207.1
Equisetum palustre isolate 17671 ribulose-1,5-bisphosphate carboxylase/oxygenase large subunit (rbcL) gene, partial cds; plastid	1129	1129	92%	0.0	99.68%	MH750191.1
Equisetum palustre isolate 39349 ribulose-1,5-bisphosphate carboxylase/oxygenase large subunit (rbcL) gene, partial cds; plastid	1127	1127	92%	0.0	99.68%	MH750192.1
Equisetum arvense subsp. arvense isolate 41071 ribulose-1,5-bisphosphate carboxylase/oxygenase large subunit (rbcL) gene, partial cds; plastid	1127	1127	93%	0.0	99.04%	MH750158.1
Equisetum x fontqueri isolate 26093 ribulose-1,5-bisphosphate carboxylase/oxygenase large subunit (rbcL) gene, partial cds; plastid	1125	1125	93%	0.0	99.04%	MH750221.1
Equisetum x litorale isolate 41084 ribulose-1,5-bisphosphate carboxylase/oxygenase large subunit (rbcL) gene, partial cds; plastid	1122	1122	93%	0.0	99.04%	MH750222.1

Description	Max Score	Total Score	Query Cover	E value	Per. Ident	Accession
Equisetum pratense isolate 41086 ribulose-1,5-bisphosphate carboxylase/oxygenase large subunit (rbcL) gene, partial cds; plastid	1118	1118	92%	0.0	99.35%	MH750194.1
Equisetum arvense voucher JAG288 (OAC) ribulose-1,5-bisphosphate carboxylase/oxygenase large subunit (rbcL) gene, partial cds; chloroplast	1118	1118	92%	0.0	99.35%	EU677106.1
Equisetum telmateia subsp. braunii isolate 40832 ribulose-1,5-bisphosphate carboxylase/oxygenase large subunit (rbcL) gene, partial cds; plastid	1116	1116	92%	0.0	99.04%	MH750208.1
Equisetum arvense subsp. arvense isolate 26085 ribulose-1,5-bisphosphate carboxylase/oxygenase large subunit (rbcL) gene, partial cds; plastid	1116	1116	92%	0.0	99.04%	MH750156.1
Equisetum pratense isolate 39348 ribulose-1,5-bisphosphate carboxylase/oxygenase large subunit (rbcL) gene, partial cds; plastid	1114	1114	91%	0.0	99.35%	MH750193.1
Equisetum diffusum isolate 40689 ribulose-1,5-bisphosphate carboxylase/oxygenase large subunit (rbcL) gene, partial cds; plastid	1110	1110	92%	0.0	98.87%	MH750166.1
Equisetum arvense subsp. arvense isolate 26084 ribulose-1,5-bisphosphate carboxylase/oxygenase large subunit (rbcL) gene, partial cds; plastid	1109	1109	92%	0.0	99.03%	MH750155.1
Equisetum arvense subsp. arvense isolate 40833 ribulose-1,5-bisphosphate carboxylase/oxygenase large subunit (rbcL) gene, partial cds; plastid	1101	1101	91%	0.0	99.02%	MH750157.1
Equisetum scirpoides ribulose-1,5-bisphosphate carboxylase/oxygenase large subunit (rbcL) gene, partial cds; chloroplast gene for chloroplast product	1101	1101	96%	0.0	97.52%	AY226133.1
Equisetum arvense voucher AP400 (OAC) ribulose-1,5-bisphosphate carboxylase/oxygenase large subunit (rbcL) gene, partial cds; chloroplast	1099	1099	90%	0.0	99.34%	EU677105.1
Equisetum diffusum isolate 40804 ribulose-1,5-bisphosphate carboxylase/oxygenase large subunit (rbcL) gene, partial cds; plastid	1098	1098	92%	0.0	98.71%	MH750167.1
Equisetum palustre chloroplast rbcL gene for ribulose-1,5-bisphosphate carboxylase/oxygenase large subunit, partial cds, specimen_voucher: TNS:776981	1094	1094	89%	0.0	99.67%	AB574686.1
Equisetum pratense voucher AP451 ribulose-1,5-bisphosphate carboxylase/oxygenase large subunit (rbcL) gene, partial cds; chloroplast	1092	1092	90%	0.0	99.01%	HQ590083.1
Equisetum fluviatile ribulose-1,5-bisphosphate carboxylase/oxygenase large subunit (rbcL) gene, partial cds; chloroplast	1092	1092	91%	0.0	99.01%	DQ463101.1

Description	Max Score	Total Score	Query Cover	E value	Per. Ident	Accession
Equisetum arvense x Equisetum telmateia subsp. braunii isolate 40834 ribulose-1,5-bisphosphate carboxylase/oxygenase large subunit (rbcL) gene, partial cds; plastid	1090	1090	90%	0.0	99.01%	MH750161.1
Equisetum xylochaetum chloroplast, complete genome	1090	1090	98%	0.0	96.51%	MW282958.1
Equisetum arvense ribulose-1,5-bisphosphate carboxylase/oxygenase large subunit (rbcL) gene, partial cds; chloroplast	1088	1088	91%	0.0	98.85%	KM218345.1
Equisetum hyemale chloroplast, complete genome	1085	1085	98%	0.0	96.36%	KC117177.1
Equisetum sylvaticum chloroplast rbcL gene for ribulose-1,5-bisphosphate carboxylase/oxygenase large subunit, partial cds, specimen_voucher: TNS:776974	1081	1081	89%	0.0	99.33%	AB574690.1
Equisetum pratense chloroplast rbcL gene for ribulose-1,5-bisphosphate carboxylase/oxygenase large subunit, partial cds, specimen_voucher: TNS:1107843	1081	1081	89%	0.0	99.33%	AB574687.1
Equisetum scirpoides isolate 41089 ribulose-1,5-bisphosphate carboxylase/oxygenase large subunit (rbcL) gene, partial cds; plastid	1079	1079	94%	0.0	97.62%	MH750203.1
Equisetum komarovii isolate 40830 ribulose-1,5-bisphosphate carboxylase/oxygenase large subunit (rbcL) gene, partial cds; plastid	1079	1079	94%	0.0	97.62%	MH750182.1
Equisetum bogotense ribulose-1,5-bisphosphate carboxylase/oxygenase large subunit (rbcL) gene, partial cds; chloroplast gene for chloroplast product	1075	1075	95%	0.0	96.88%	AY226139.1
Equisetum ramosissimum isolate BPTPS123 voucher LISE:96444 chloroplast, complete genome	1074	1074	98%	0.0	96.05%	ON641349.1
Equisetum ramosissimum isolate BPTPS056 voucher LISE:96334 chloroplast, complete genome	1074	1074	98%	0.0	96.05%	NC_062377.1
Equisetum ramosissimum isolate BPTPS056 voucher LISE:96334 chloroplast, complete genome	1074	1074	98%	0.0	96.05%	OM691674.1
Equisetum myriochaetum ribulose-1,5-bisphosphate carboxylase/oxygenase large subunit (rbcL) gene, partial cds; chloroplast gene for chloroplast product	1074	1074	96%	0.0	96.74%	AY226131.1
Equisetum giganteum ribulose-1,5-bisphosphate carboxylase/oxygenase large subunit (rbcL) gene, partial cds; chloroplast gene for chloroplast product	1074	1074	96%	0.0	96.74%	AY226127.1
Equisetum sp. Schuettpelz 1643 ribulose-1,5-bisphosphate carboxylase/oxygenase large subunit (rbcL) gene, partial cds; chloroplast	1072	1072	96%	0.0	96.73%	MW620286.1

Description	Max Score	Total Score	Query Cover	E value	Per. Ident	Accession
Equisetum fluviatile chloroplast rbcL gene for ribulose-1,5-bisphosphate carboxylase/oxygenase large subunit, partial cds, specimen_voucher: TNS:776384	1072	1072	89%	0.0	99.00%	AB574684.1
Equisetum hyemale ribulose-1,5-bisphosphate carboxylase/oxygenase large subunit (rbcL) gene, partial cds; chloroplast	1070	1070	98%	0.0	95.90%	DQ646001.1
Equisetum laevigatum ribulose-1,5-bisphosphate carboxylase/oxygenase large subunit (rbcL) gene, partial cds; chloroplast gene for chloroplast product	1070	1070	95%	0.0	96.73%	AY226130.1
Equisetum scirpoides isolate 26090 ribulose-1,5-bisphosphate carboxylase/oxygenase large subunit (rbcL) gene, partial cds; plastid	1068	1068	94%	0.0	97.30%	MH750202.1
Equisetum hyemale subsp. affine voucher OAC 94916 ribulose-1,5-bisphosphate carboxylase/oxygenase large subunit (rbcL) gene, partial cds; chloroplast	1068	1068	96%	0.0	96.58%	KF186500.1
Equisetum hyemale subsp. affine ribulose-1,5-bisphosphate carboxylase/oxygenase large subunit (rbcL) gene, partial cds; chloroplast gene for chloroplast product	1068	1068	96%	0.0	96.58%	AY226128.1
Equisetum giganteum isolate 40806 ribulose-1,5-bisphosphate carboxylase/oxygenase large subunit (rbcL) gene, partial cds; plastid	1066	1066	95%	0.0	96.72%	MH750172.1
Equisetum giganteum isolate 26087 ribulose-1,5-bisphosphate carboxylase/oxygenase large subunit (rbcL) gene, partial cds; plastid	1066	1066	95%	0.0	96.72%	MH750170.1
Equisetum ramosissimum voucher R825 ribulose-1,5-bisphosphate carboxylase/oxygenase large subunit (rbcl) gene, partial cds; chloroplast	1066	1066	97%	0.0	96.30%	KY656709.1
Equisetum arvense chloroplast rbcL gene for ribulose-1,5-bisphosphate carboxylase/oxygenase large subunit, partial cds, specimen_voucher: TNS:763618	1066	1066	89%	0.0	98.83%	AB574683.1
Equisetum x ferrissii ribulose-1,5-bisphosphate carboxylase/oxygenase large subunit (rbcL) gene, partial cds; chloroplast	1066	1066	95%	0.0	96.72%	EU352292.1
Equisetum x ferrissii ribulose-1,5-bisphosphate carboxylase large subunit (rbcL) gene, partial cds; chloroplast gene for chloroplast product	1064	1064	95%	0.0	96.71%	AF313579.1
Equisetum scirpoides isolate 10933 ribulose-1,5-bisphosphate carboxylase/oxygenase large subunit (rbcL) gene, partial cds; plastid	1059	1059	92%	0.0	97.57%	MH750201.1
Equisetum ramosissimum subsp. debile voucher G.M. Plunkett 3642 (NYBG) ribulose-1,5-bisphosphate carboxylase/oxygenase large subunit (rbcL) gene, partial cds; chloroplast	1057	1057	96%	0.0	96.27%	MT657901.1

Description	Max Score	Total Score	Query Cover	E value	Per. Ident	Accession
Equisetum ramosissimum subsp. debile ribulose-1,5-bisphosphate carboxylase/oxygenase large subunit (rbcL) gene, partial cds; chloroplast gene for chloroplast product	1057	1057	96%	0.0	96.27%	AY226132.1
Equisetum myriochaetum isolate 40815 ribulose-1,5-bisphosphate carboxylase/oxygenase large subunit (rbcL) gene, partial cds; plastid	1053	1053	95%	0.0	96.55%	MH750185.1
Equisetum scirpoides voucher AP248 ribulose-1,5-bisphosphate carboxylase/oxygenase large subunit (rbcL) gene, partial cds; chloroplast	1050	1050	90%	0.0	97.86%	HQ590084.1
Equisetum xylochaetum isolate 40614 ribulose-1,5-bisphosphate carboxylase/oxygenase large subunit (rbcL) gene, partial cds; plastid	1046	1046	94%	0.0	96.66%	MH750228.1
Equisetum myriochaetum isolate 41080 ribulose-1,5-bisphosphate carboxylase/oxygenase large subunit (rbcL) gene, partial cds; plastid	1046	1046	94%	0.0	96.66%	MH750190.1
Equisetum myriochaetum isolate 40826 ribulose-1,5-bisphosphate carboxylase/oxygenase large subunit (rbcL) gene, partial cds; plastid	1046	1046	94%	0.0	96.66%	MH750188.1
Equisetum sylvaticum voucher TJD-502 ribulose-1,5-bisphosphate carboxylase/oxygenase large subunit (rbcL) gene, partial cds; chloroplast	1046	1046	85%	0.0	99.82%	KJ841305.1
Equisetum x schaffneri isolate 40813 ribulose-1,5-bisphosphate carboxylase/oxygenase large subunit (rbcL) gene, partial cds; plastid	1044	1044	93%	0.0	96.66%	MH750224.1
Equisetum giganteum isolate 40811 ribulose-1,5-bisphosphate carboxylase/oxygenase large subunit (rbcL) gene, partial cds; plastid	1042	1042	93%	0.0	96.65%	MH750176.1
Equisetum hyemale subsp. hyemale isolate 41088 ribulose-1,5-bisphosphate carboxylase/oxygenase large subunit (rbcL) gene, partial cds; plastid	1040	1040	94%	0.0	96.50%	MH750181.1
Equisetum variegatum ribulose-1,5-bisphosphate carboxylase/oxygenase large subunit (rbcL) gene, partial cds; chloroplast gene for chloroplast product	1040	1040	96%	0.0	95.81%	AY226134.1
Equisetum bogotense isolate 40800 ribulose-1,5-bisphosphate carboxylase/oxygenase large subunit (rbcL) gene, partial cds; plastid	1037	1037	92%	0.0	96.78%	MH750162.1
Equisetum arvense voucher TJD-508 ribulose-1,5-bisphosphate carboxylase/oxygenase large subunit (rbcL) gene, partial cds; chloroplast	1037	1037	85%	0.0	99.47%	KJ841301.1
Equisetum arvense voucher TJD-503 ribulose-1,5-bisphosphate carboxylase/oxygenase large subunit (rbcL) gene, partial cds; chloroplast	1035	1035	85%	0.0	99.47%	KJ841302.1
Equisetum giganteum isolate 40807 ribulose-1,5-bisphosphate carboxylase/oxygenase large subunit (rbcL) gene, partial cds; plastid	1033	1033	92%	0.0	96.62%	MH750173.1

Description	Max Score	Total Score	Query Cover	E value	Per. Ident	Accession
Equisetum arvense voucher TJD-504 ribulose-1,5-bisphosphate carboxylase/oxygenase large subunit (rbcL) gene, partial cds; chloroplast	1033	1033	84%	0.0	99.47%	KJ841300.1
Equisetum trachyodon isolate 41092 ribulose-1,5-bisphosphate carboxylase/oxygenase large subunit (rbcL) gene, partial cds; plastid	1029	1029	93%	0.0	96.33%	MH750227.1
Equisetum ramosissimum subsp. debile isolate 40829 ribulose-1,5-bisphosphate carboxylase/oxygenase large subunit (rbcL) gene, partial cds; plastid	1029	1029	94%	0.0	96.18%	MH750200.1
Equisetum myriochaetum isolate 40936 ribulose-1,5-bisphosphate carboxylase/oxygenase large subunit (rbcL) gene, partial cds; plastid	1029	1029	92%	0.0	96.61%	MH750189.1
Equisetum hyemale voucher AP344 (OAC) ribulose-1,5-bisphosphate carboxylase/oxygenase large subunit (rbcL) gene, partial cds; chloroplast	1029	1029	92%	0.0	96.76%	EU677107.1
Equisetum hyemale subsp. affine isolate 40831 ribulose-1,5-bisphosphate carboxylase/oxygenase large subunit (rbcL) gene, partial cds; plastid	1027	1027	92%	0.0	96.46%	MH750177.1
Equisetum sylvaticum plastid partial rbcL gene for ribulose-1,5-biphosphate carboxylase/oxygenase large subunit, specimen voucher H823764	1027	1027	83%	0.0	99.82%	HE574610.1
Equisetum ramosissimum subsp. debile isolate 36802 ribulose-1,5-bisphosphate carboxylase/oxygenase large subunit (rbcL) gene, partial cds; plastid	1026	1026	94%	0.0	96.03%	MH750199.1
Commelina diffusa voucher Q555 ribulose-1,5-bisphosphate carboxylase/oxygenase large subunit (rbcL) gene, partial cds; chloroplast	1026	1026	84%	0.0	99.29%	MH714073.1
Equisetum myriochaetum isolate 26088 ribulose-1,5-bisphosphate carboxylase/oxygenase large subunit (rbcL) gene, partial cds; plastid	1022	1022	92%	0.0	96.59%	MH750184.1
Equisetum scirpoides chloroplast rbcL gene for ribulose-1,5-bisphosphate carboxylase/oxygenase large subunit, partial cds, specimen_voucher: TNS:768168	1022	1022	89%	0.0	97.49%	AB574689.1
Equisetum palustre voucher Chase 11641 (K) ribulose-1,5-bisphosphate carboxylase/oxygenase large subunit (rbcL) gene, partial cds; chloroplast	1022	1022	82%	0.0	100.00%	GQ248601.1
Equisetum laevigatum isolate 40812 ribulose-1,5-bisphosphate carboxylase/oxygenase large subunit (rbcL) gene, partial cds; plastid	1020	1020	91%	0.0	96.59%	MH750183.1

Alignments

Equisetum palustre chloroplast, partial genome

Sequence ID: **MT984458.1** Length: 5501 Number of Matches: 1
Range 1: 2224 to 2882

Score	Expect	Identities	Gaps	Strand	Frame
1195 bits(647)	0.0()	655/659(99%)	0/659(0%)	Plus/Plus	

Features:

```
Query  1     AACAGAGACTAAAGCAGGTGTTGGATTTAAAGCTGGTGTTAAAGATTATCGATTGACTTA  60
             ||| ||||| ||||||||||||||||||||||||||||||||||||||||||||||||||
Sbjct  2224  AACGGAGACCAAAGCAGGTGTTGGATTTAAAGCTGGTGTTAAAGATTATCGATTGACTTA  2283

Query  61    TTTTACTCCAGATTATGAAACCAAAGATACCGATATTTTAGCAGCATTCCGTATGACTCC  120
             ||||||||||||||||||||||||||||||||||||||||||||||||||||||||||||
Sbjct  2284  TTTTACTCCAGATTATGAAACCAAAGATACCGATATTTTAGCAGCATTCCGTATGACTCC  2343

Query  121   TCAACCGGGAGTACCACCGGAAGAAGCAGGAGCAGCCGTAGCTGCTGAATCGTCCACGGG  180
             ||||||||||||||||||||||||||||||||||||||||||||||||||||||||||||
Sbjct  2344  TCAACCGGGAGTACCACCGGAAGAAGCAGGAGCAGCCGTAGCTGCTGAATCGTCCACGGG  2403

Query  181   CACCTGGACTACCGTATGGACAGATGGACTTACTAGTCTTGATCGATATAAAGGTCGATG  240
             ||||||||||||||||||||||||||||||||||||||||||||||||||||||||||||
Sbjct  2404  CACCTGGACTACCGTATGGACAGATGGACTTACTAGTCTTGATCGATATAAAGGTCGATG  2463

Query  241   CTATAATATTGAACCTGTTGCTGGAGAAGATAATCAATTCATAGCTTATGTAGCCTATCC  300
             ||||||||||||||||||||||||||||||||||||||||||||||||||||||||||||
Sbjct  2464  CTATAATATTGAACCTGTTGCTGGAGAAGATAATCAATTCATAGCTTATGTAGCCTATCC  2523

Query  301   TTTAGATCTTTTTGAAGAAGGTTCTGTTACCAATCTTTTTACTTCAATTGTTGGTAATGT  360
             ||||||||||||||||||||||||||||||||||||||||||||||||||||||||||||
Sbjct  2524  TTTAGATCTTTTTGAAGAAGGTTCTGTTACCAATCTTTTTACTTCAATTGTTGGTAATGT  2583

Query  361   TTTCGGCTTCAAAGCTCTACGTGCTTTACGTTTAGAAGATTTAAGAATTCCTCCTGCTTA  420
             ||||||||||||||||||||||||||||||||||||||||||||||||||||||||||||
Sbjct  2584  TTTCGGCTTCAAAGCTCTACGTGCTTTACGTTTAGAAGATTTAAGAATTCCTCCTGCTTA  2643

Query  421   CTCCAAAACTTTTATAGGACCACCCCACGGTATCCAGGTTGAAAGAGATAAGTTAAACAA  480
             ||||||||||||||||||||||||||||||||||||||||||||||||||||||||||||
Sbjct  2644  CTCCAAAACTTTTATAGGACCACCCCACGGTATCCAGGTTGAAAGAGATAAGTTAAACAA  2703

Query  481   ATATGGTCGTCCTTTATTAGGTTGTACAATTAAACCAAAATTGGGACTATCTGCTAAAAA  540
             ||||||||||||||||||||||||||||||||||||||||||||||||||||||||||||
Sbjct  2704  ATATGGTCGTCCTTTATTAGGTTGTACAATTAAACCAAAATTGGGACTATCTGCTAAAAA  2763

Query  541   CTATGGTAGGGCTGTTTATGAATGTCTTCGTGGTGGACTTGATTTCACCAAAGATGATGA  600
             ||||||||||||||||||||||||||||||||||||||||||||||||||||||||||||
Sbjct  2764  CTATGGTAGGGCTGTTTATGAATGTCTTCGTGGTGGACTTGATTTCACCAAAGATGATGA  2823

Query  601   GAATGTAAACTCTCAACCCTTTATGCGTTGGAGAGATCGATTCTTATTTGTAGCAGAAG  659
             ||||||||||||||||||||||||||||||||||||||| |||||||| ||||||||||
Sbjct  2824  GAATGTAAACTCTCAACCCTTTATGCGTTGGAGAGATCGTTTCTTATTCGTAGCAGAAG  2882
```

Equisetum palustre ribulose-1,5-bisphosphate carboxylase/oxygenase large subunit (rbcL) gene, partial cds; chloroplast gene for chloroplast product

Sequence ID: **AY226138.1** Length: 1325 Number of Matches: 1

Range 1: 1 to 644

Score	Expect	Identities	Gaps	Strand	Frame
1179 bits(638)	0.0()	642/644(99%)	0/644(0%)	Plus/Plus	

Features:

```
Query  16   AGGTGTTGGATTTAAAGCTGGTGTTAAAGATTATCGATTGACTTATTTTACTCCAGATTA  75
            ||||||||||||||||||||||||||||||||||||||||||||||||||||||||||||
Sbjct  1    AGGTGTTGGATTTAAAGCTGGTGTTAAAGATTATCGATTGACTTATTTTACTCCAGATTA  60

Query  76   TGAAACCAAAGATACCGATATTTTAGCAGCATTCCGTATGACTCCTCAACCGGGAGTACC  135
            ||||||||||||||||||||||||||||||||||||||||||||||||||||||||||||
Sbjct  61   TGAAACCAAAGATACCGATATTTTAGCAGCATTCCGTATGACTCCTCAACCGGGAGTACC  120

Query  136  ACCGGAAGAAGCAGGAGCAGCCGTAGCTGCTGAATCGTCCACGGGCACCTGGACTACCGT  195
            ||||||||||||||||||||||||||||||||||||||||||||||||||||||||||||
Sbjct  121  ACCGGAAGAAGCAGGAGCAGCCGTAGCTGCTGAATCGTCCACGGGCACCTGGACTACCGT  180

Query  196  ATGGACAGATGGACTTACTAGTCTTGATCGATATAAAGGTCGATGCTATAATATTGAACC  255
            ||||||||||||||||||||||||||||||||||||||||||||||||||||||||||||
Sbjct  181  ATGGACAGATGGACTTACTAGTCTTGATCGATATAAAGGTCGATGCTATAATATTGAACC  240

Query  256  TGTTGCTGGAGAAGATAATCAATTCATAGCTTATGTAGCCTATCCTTTAGATCTTTTTGA  315
            ||||||||||||||||||||||||||||||||||||||||||||||||||||||||||||
Sbjct  241  TGTTGCTGGAGAAGATAATCAATTCATAGCTTATGTAGCCTATCCTTTAGATCTTTTTGA  300

Query  316  AGAAGGTTCTGTTACCAATCTTTTTACTTCAATTGTTGGTAATGTTTTCGGCTTCAAAGC  375
            ||||||||||||||||||||||||||||||||||||||||||||||||||||||||||||
Sbjct  301  AGAAGGTTCTGTTACCAATCTTTTTACTTCAATTGTTGGTAATGTTTTCGGCTTCAAAGC  360
```

```
Query  376   TCTACGTGCTTTACGTTTAGAAGATTTAAGAATTCCTCCTGCTTACTCCAAAACTTTTAT  435
             ||||||||||||||||||||||||||||||||||||||||||||||||||||||||||||
Sbjct  361   TCTACGTGCTTTACGTTTAGAAGATTTAAGAATTCCTCCTGCTTACTCCAAAACTTTTAT  420

Query  436   AGGACCACCCCACGGTATCCAGGTTGAAAGAGATAAGTTAAACAAATATGGTCGTCCTTT  495
             ||||||||||||||||||||||||||||||||||||||||||||||||||||||||||||
Sbjct  421   AGGACCACCCCACGGTATCCAGGTTGAAAGAGATAAGTTAAACAAATATGGTCGTCCTTT  480

Query  496   ATTAGGTTGTACAATTAAACCAAAATTGGGACTATCTGCTAAAAACTATGGTAGGGCTGT  555
             ||||||||||||||||||||||||||||||||||||||||||||||||||||||||||||
Sbjct  481   ATTAGGTTGTACAATTAAACCAAAATTGGGACTATCTGCTAAAAACTATGGTAGGGCTGT  540

Query  556   TTATGAATGTCTTCGTGGTGGACTTGATTTCACCAAAGATGATGAGAATGTAAACTCTCA  615
             ||||||||||||||||||||||||||||||||||||||||||||||||||||||||||||
Sbjct  541   TTATGAATGTCTTCGTGGTGGACTTGATTTCACCAAAGATGATGAGAATGTAAACTCTCA  600

Query  616   ACCCTTTATGCGTTGGAGAGATCGATTCTTATTTGTAGCAGAAG  659
             |||||||||||||||||||||||| |||||||| ||||||||||
Sbjct  601   ACCCTTTATGCGTTGGAGAGATCGTTTCTTATTCGTAGCAGAAG  644
```

Equisetum arvense chloroplast, complete genome

Sequence ID: **GU191334.1** Length: 133309 Number of Matches: 1

Range 1: 27302 to 27960

Score	Expect	Identities	Gaps	Strand	Frame
1173 bits(635)	0.0()	651/659(99%)	0/659(0%)	Plus/Minus	

Features:

```
Query  1       AACAGAGACTAAAGCAGGTGTTGGATTTAAAGCTGGTGTTAAAGATTATCGATTGACTTA  60
               ||| ||||| ||||||||||||||||||||||||||||||||||||||||||||||||||
Sbjct  27960   AACGGAGACCAAAGCAGGTGTTGGATTTAAAGCTGGTGTTAAAGATTATCGATTGACTTA  27901

Query  61      TTTTACTCCAGATTATGAAACCAAAGATACCGATATTTTAGCAGCATTCCGTATGACTCC  120
               ||||||||||||||||||||||||||||||||||||||||||||||||||||||||||||
Sbjct  27900   TTTTACTCCAGATTATGAAACCAAAGATACCGATATTTTAGCAGCATTCCGTATGACTCC  27841

Query  121     TCAACCGGGAGTACCACCGGAAGAAGCAGGAGCAGCCGTAGCTGCTGAATCGTCCACGGG  180
               ||||||||||||||||||||||||||||||||||||||||||||||||||||||||||||
Sbjct  27840   TCAACCGGGAGTACCACCGGAAGAAGCAGGAGCAGCCGTAGCTGCTGAATCGTCCACGGG  27781

Query  181     CACCTGGACTACCGTATGGACAGATGGACTTACTAGTCTTGATCGATATAAAGGTCGATG  240
               ||||||||||||||||||||||||||||||||||||||||||||||||||||||||||||
Sbjct  27780   CACCTGGACTACCGTATGGACAGATGGACTTACTAGTCTTGATCGATATAAAGGTCGATG  27721

Query  241     CTATAATATTGAACCTGTTGCTGGAGAAGATAATCAATTCATAGCTTATGTAGCCTATCC  300
               ||||||||||||||||||||||||||||||||||||||||||||||||||||||||| ||
Sbjct  27720   CTATAATATTGAACCTGTTGCTGGAGAAGATAATCAATTCATAGCTTATGTAGCCTACCC  27661

Query  301     TTTAGATCTTTTTGAAGAAGGTTCTGTTACCAATCTTTTTACTTCAATTGTTGGTAATGT  360
               ||||||||||||||||||||||||||||||||||||||||||||||||||||||||||||
Sbjct  27660   TTTAGATCTTTTTGAAGAAGGTTCTGTTACCAATCTTTTTACTTCAATTGTTGGTAATGT  27601

Query  361     TTTCGGCTTCAAAGCTCTACGTGCTTTACGTTTAGAAGATTTAAGAATTCCTCCTGCTTA  420
               ||||||||||||||||||||||||||||||||||||||||||||||||||||||||||||
Sbjct  27600   TTTCGGCTTCAAAGCTCTACGTGCTTTACGTTTAGAAGATTTAAGAATTCCTCCTGCTTA  27541

Query  421     CTCCAAAACTTTTATAGGACCACCCCACGGTATCCAGGTTGAAAGAGATAAGTTAAACAA  480
               ||||||||||||||||||||||||||||||||||||||||||||||||||||||||||||
Sbjct  27540   CTCCAAAACTTTTATAGGACCACCCCACGGTATCCAGGTTGAAAGAGATAAGTTAAACAA  27481

Query  481     ATATGGTCGTCCTTTATTAGGTTGTACAATTAAACCAAAATTGGGACTATCTGCTAAAAA  540
               ||| ||||||||||||||||||||||||||||||||||||||||||||||||||||||||
Sbjct  27480   ATACGGTCGTCCTTTATTAGGTTGTACAATTAAACCAAAATTGGGACTATCTGCTAAAAA  27421

Query  541     CTATGGTAGGGCTGTTTATGAATGTCTTCGTGGTGGACTTGATTTCACCAAAGATGATGA  600
               ||||||||| |||||||||||||||||||||||||||| |||||||||||||||||||||||
Sbjct  27420   CTATGGTAGAGCTGTTTATGAATGTCTTCGTGGTGGGCTTGATTTCACCAAAGATGATGA  27361

Query  601     GAATGTAAACTCTCAACCCTTTATGCGTTGGAGAGATCGATTCTTATTTGTAGCAGAAG  659
               ||||||||||||||||||||||||||||||||||||||| |||||||| ||||||||||
Sbjct  27360   GAATGTAAACTCTCAACCCTTTATGCGTTGGAGAGATCGTTTCTTATTCGTAGCAGAAG  27302
```

Equisetum arvense chloroplast ribulosebisphosphate carboxylase (rbcL) gene, complete cds

Sequence ID: **L11053.1** Length: 1587 Number of Matches: 1

Range 1: 84 to 742

Score	Expect	Identities	Gaps	Strand	Frame
1173 bits(635)	0.0()	651/659(99%)	0/659(0%)	Plus/Plus	

Features:

```
Query  1    AACAGAGACTAAAGCAGGTGTTGGATTTAAAGCTGGTGTTAAAGATTATCGATTGACTTA  60
            ||| ||||| ||||||||||||||||||||||||||||||||||||||||||||||||||
Sbjct  84   AACGGAGACCAAAGCAGGTGTTGGATTTAAAGCTGGTGTTAAAGATTATCGATTGACTTA  143

Query  61   TTTTACTCCAGATTATGAAACCAAAGATACCGATATTTTAGCAGCATTCCGTATGACTCC  120
            ||||||||||||||||||||||||||||||||||||||||||||||||||||||||||||
Sbjct  144  TTTTACTCCAGATTATGAAACCAAAGATACCGATATTTTAGCAGCATTCCGTATGACTCC  203

Query  121  TCAACCGGGAGTACCACCGGAAGAAGCAGGAGCAGCCGTAGCTGCTGAATCGTCCACGGG  180
            ||||||||||||||||||||||||||||||||||||||||||||||||||||||||||||
Sbjct  204  TCAACCGGGAGTACCACCGGAAGAAGCAGGAGCAGCCGTAGCTGCTGAATCGTCCACGGG  263

Query  181  CACCTGGACTACCGTATGGACAGATGGACTTACTAGTCTTGATCGATATAAAGGTCGATG  240
            ||||||||||||||||||||||||||||||||||||||||||||||||||||||||||||
Sbjct  264  CACCTGGACTACCGTATGGACAGATGGACTTACTAGTCTTGATCGATATAAAGGTCGATG  323

Query  241  CTATAATATTGAACCTGTTGCTGGAGAAGATAATCAATTCATAGCTTATGTAGCCTATCC  300
            ||||||||||||||||||||||||||||||||||||||||||||||||||||||||| ||
Sbjct  324  CTATAATATTGAACCTGTTGCTGGAGAAGATAATCAATTCATAGCTTATGTAGCCTACCC  383

Query  301  TTTAGATCTTTTTGAAGAAGGTTCTGTTACCAATCTTTTTACTTCAATTGTTGGTAATGT  360
            ||||||||||||||||||||||||||||||||||||||||||||||||||||||||||||
Sbjct  384  TTTAGATCTTTTTGAAGAAGGTTCTGTTACCAATCTTTTTACTTCAATTGTTGGTAATGT  443

Query  361  TTTCGGCTTCAAAGCTCTACGTGCTTTACGTTTAGAAGATTTAAGAATTCCTCCTGCTTA  420
            ||||||||||||||||||||||||||||||||||||||||||||||||||||||||||||
Sbjct  444  TTTCGGCTTCAAAGCTCTACGTGCTTTACGTTTAGAAGATTTAAGAATTCCTCCTGCTTA  503

Query  421  CTCCAAAACTTTTATAGGACCACCCCACGGTATCCAGGTTGAAAGAGATAAGTTAAACAA  480
            ||||||||||||||||||||||||||||||||||||||||||||||||||||||||||||
Sbjct  504  CTCCAAAACTTTTATAGGACCACCCCACGGTATCCAGGTTGAAAGAGATAAGTTAAACAA  563

Query  481  ATATGGTCGTCCTTTATTAGGTTGTACAATTAAACCAAAATTGGGACTATCTGCTAAAAA  540
            ||| ||||||||||||||||||||||||||||||||||||||||||||||||||||||||
Sbjct  564  ATACGGTCGTCCTTTATTAGGTTGTACAATTAAACCAAAATTGGGACTATCTGCTAAAAA  623

Query  541  CTATGGTAGGGCTGTTTATGAATGTCTTCGTGGTGGACTTGATTTCACCAAAGATGATGA  600
            ||||||||| ||||||||||||||||||||||||| ||||||||||||||||||||||||
Sbjct  624  CTATGGTAGAGCTGTTTATGAATGTCTTCGTGGTGGGCTTGATTTCACCAAAGATGATGA  683

Query  601  GAATGTAAACTCTCAACCCTTTATGCGTTGGAGAGATCGATTCTTATTTGTAGCAGAAG  659
            ||||||||||||||||||||||||||||||||||||||| |||||||| ||||||||||
Sbjct  684  GAATGTAAACTCTCAACCCTTTATGCGTTGGAGAGATCGTTTCTTATTCGTAGCAGAAG  742
```

Equisetum arvense plastid, complete genome

Sequence ID: **JN968380.1** Length: 132726 Number of Matches: 1

Range 1: 64969 to 65627

Score	Expect	Identities	Gaps	Strand	Frame
1168 bits(632)	0.0()	650/659(99%)	0/659(0%)	Plus/Plus	

Features:

```
Query  1      AACAGAGACTAAAGCAGGTGTTGGATTTAAAGCTGGTGTTAAAGATTATCGATTGACTTA  60
              ||| ||||| ||||||||||||||||||||||||||||||||||||||||||||||||||
Sbjct  64969  AACGGAGACCAAAGCAGGTGTTGGATTTAAAGCTGGTGTTAAAGATTATCGATTGACTTA  65028

Query  61     TTTTACTCCAGATTATGAAACCAAAGATACCGATATTTTAGCAGCATTCCGTATGACTCC  120
              ||||||||||||||||||||||||||||||||||||||||||||||||||||||||||||
Sbjct  65029  TTTTACTCCAGATTATGAAACCAAAGATACCGATATTTTAGCAGCATTCCGTATGACTCC  65088

Query  121    TCAACCGGGAGTACCACCGGAAGAAGCAGGAGCAGCCGTAGCTGCTGAATCGTCCACGGG  180
              ||||||||||||||||||||||||||||||||||||||||||||||||||||||||||||
Sbjct  65089  TCAACCGGGAGTACCACCGGAAGAAGCAGGAGCAGCCGTAGCTGCTGAATCGTCCACGGG  65148

Query  181    CACCTGGACTACCGTATGGACAGATGGACTTACTAGTCTTGATCGATATAAAGGTCGATG  240
              ||||||||||||||||||||||||||||||||||||||||||||||||||||||||||||
Sbjct  65149  CACCTGGACTACCGTATGGACAGATGGACTTACTAGTCTTGATCGATATAAAGGTCGATG  65208

Query  241    CTATAATATTGAACCTGTTGCTGGAGAAGATAATCAATTCATAGCTTATGTAGCCTATCC  300
              ||||||||||||||||||||||||||||||||||||||||||||||||||||||||| ||
Sbjct  65209  CTATAATATTGAACCTGTTGCTGGAGAAGATAATCAATTCATAGCTTATGTAGCCTACCC  65268

Query  301    TTTAGATCTTTTTGAAGAAGGTTCTGTTACCAATCTTTTTACTTCAATTGTTGGTAATGT  360
              ||||||||||||||||||||||||||||||||||||||||||||||||||||||||||||
Sbjct  65269  TTTAGATCTTTTTGAAGAAGGTTCTGTTACCAATCTTTTTACTTCAATTGTTGGTAATGT  65328

Query  361    TTTCGGCTTCAAAGCTCTACGTGCTTTACGTTTAGAAGATTTAAGAATTCCTCCTGCTTA  420
              |||||| |||||||||||||||||||||||||||||||||||||||||||||||||||||
Sbjct  65329  TTTCGGATTCAAAGCTCTACGTGCTTTACGTTTAGAAGATTTAAGAATTCCTCCTGCTTA  65388

Query  421    CTCCAAAACTTTTATAGGACCACCCCACGGTATCCAGGTTGAAAGAGATAAGTTAAACAA  480
              ||||||||||||||||||||||||||||||||||||||||||||||||||||||||||||
Sbjct  65389  CTCCAAAACTTTTATAGGACCACCCCACGGTATCCAGGTTGAAAGAGATAAGTTAAACAA  65448
```

```
Query  481    ATATGGTCGTCCTTTATTAGGTTGTACAATTAAACCAAAATTGGGACTATCTGCTAAAAA  540
              ||| ||||||||||||||||||||||||||||||||||||||||||||||||||||||||
Sbjct  65449  ATACGGTCGTCCTTTATTAGGTTGTACAATTAAACCAAAATTGGGACTATCTGCTAAAAA  65508

Query  541    CTATGGTAGGGCTGTTTATGAATGTCTTCGTGGTGGACTTGATTTCACCAAAGATGATGA  600
              ||||||||| |||||||||||||||||||||||||| |||||||||||||||||||||||
Sbjct  65509  CTATGGTAGAGCTGTTTATGAATGTCTTCGTGGTGGGCTTGATTTCACCAAAGATGATGA  65568

Query  601    GAATGTAAACTCTCAACCCTTTATGCGTTGGAGAGATCGATTCTTATTTGTAGCAGAAG  659
              ||||||||||||||||||||||||||||||||||||||| |||||||| ||||||||||
Sbjct  65569  GAATGTAAACTCTCAACCCTTTATGCGTTGGAGAGATCGTTTCTTATTCGTAGCAGAAG  65627
```


BLAST is a registered trademark of the National Library of Medicine

Support center Mailing list YouTube

- National Library Of Medicine
- National Institutes Of Health
- U.S. Department of Health & Human Services
- USA.gov

NCBI

National Center for Biotechnology Information, U.S. National Library of Medicine 8600 Rockville Pike, Bethesda MD, 20894 USA
Policies and Guidelines | Contact

HHS Vulnerability Disclosure
