# Supplementary material for: A Multi-Pronged Technique for Identifying Equisetum palustre and Equisetum arvense—Combining HPTLC, HPLC-ESI-MS/MS and Optimized DNA Barcoding Techniques
Source: Plants (Basel). 2022 Sep 28;11(19):2562. doi: 10.3390/plants11192562 (PMC9573248; doi:10.3390/plants11192562)
Supplement: Supplementary file 1 [file plants-11-02562-s001.zip › plants-1932149 supplementary/Horsetail Supplementary Figures/Figure S21 (P3, 82709220323-1).pdf]

BLAST Results

Questions/comments

Job title: Nucleotide Sequence

**RID**	J1BXM44C013 (Expires on 09-15 00:28 am)
**Query ID**	lcl|Query_22561
**Description**	None
**Molecule type**	dna
**Query Length**	642
**Database Name**	nt
**Description**	Nucleotide collection (nt)
**Program**	BLASTN 2.13.0+

Graphic Summary

Distribution of the top 100 Blast Hits on 100 subject sequences

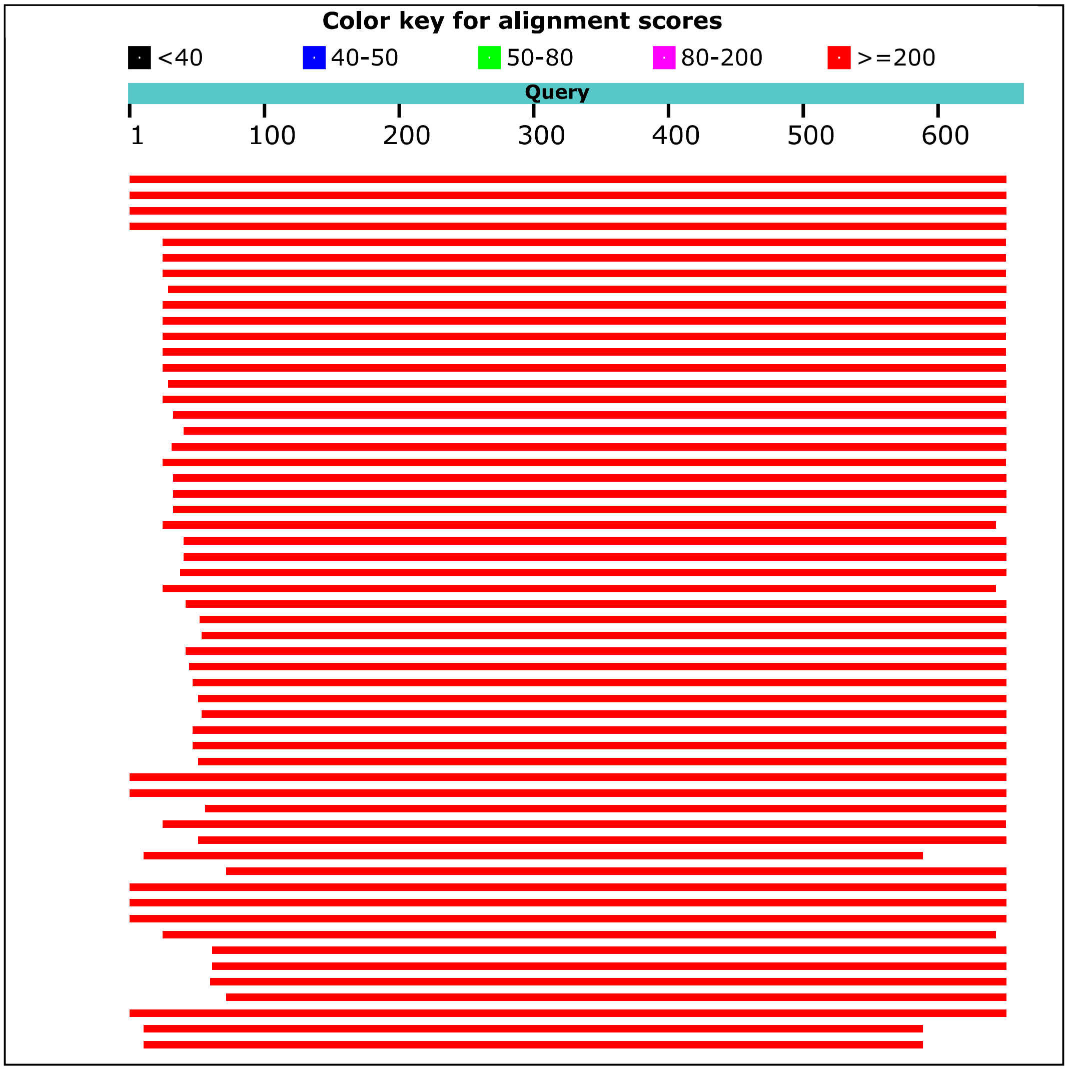

Descriptions

Sequences producing significant alignments:

Description	Max Score	Total Score	Query Cover	E value	Per. Ident	Accession
Equisetum palustre chloroplast, partial genome	1166	1166	99%	0.0	99.53%	MT984458.1
Equisetum arvense chloroplast, complete genome	1144	1144	99%	0.0	98.91%	GU191334.1
Equisetum arvense chloroplast ribulosebisphosphate carboxylase (rbcL) gene, complete cds	1144	1144	99%	0.0	98.91%	L11053.1
Equisetum arvense plastid, complete genome	1138	1138	99%	0.0	98.75%	JN968380.1
Equisetum palustre ribulose-1,5-bisphosphate carboxylase/oxygenase large subunit (rbcL) gene, partial cds; chloroplast gene for chloroplast product	1131	1131	95%	0.0	99.84%	AY226138.1
Equisetum sylvaticum ribulose-1,5-bisphosphate carboxylase/oxygenase large subunit (rbcL) gene, partial cds; chloroplast gene for chloroplast product	1125	1125	95%	0.0	99.68%	AY226136.1
Equisetum pratense ribulose-1,5-bisphosphate carboxylase/oxygenase large subunit (rbcL) gene, partial cds; chloroplast gene for chloroplast product	1120	1120	95%	0.0	99.51%	AY226137.1
Equisetum sylvaticum isolate 11640 ribulose-1,5-bisphosphate carboxylase/oxygenase large subunit (rbcL) gene, partial cds; plastid	1118	1118	95%	0.0	99.67%	MH750204.1
Equisetum telmateia subsp. braunii ribulose-1,5-bisphosphate carboxylase/oxygenase large subunit (rbcL) gene, partial cds; chloroplast gene for chloroplast product	1114	1114	95%	0.0	99.35%	AY226135.1
Equisetum environmental sample isolate EDNA16-0043490 ribulose-1,5-bisphosphate carboxylase/oxygenase large subunit (rbcL) gene, partial cds; chloroplast	1109	1109	95%	0.0	99.19%	MT197555.1
Equisetum arvense voucher JAG288 (OAC) ribulose-1,5-bisphosphate carboxylase/oxygenase large subunit (rbcL) gene, partial cds; chloroplast	1109	1109	95%	0.0	99.19%	EU677106.1
Equisetum fluviatile ribulose-1,5-bisphosphate carboxylase/oxygenase large subunit (rbcL) gene, partial cds; chloroplast gene for chloroplast product	1109	1109	95%	0.0	99.19%	AY226142.1
Equisetum arvense ribulose-1,5-bisphosphate carboxylase/oxygenase large subunit (rbcL) gene, partial cds; chloroplast gene for chloroplast product	1109	1109	95%	0.0	99.19%	AY226140.1
Equisetum telmateia subsp. telmateia isolate 11642 ribulose-1,5-bisphosphate carboxylase/oxygenase large subunit (rbcL) gene, partial cds; plastid	1107	1107	95%	0.0	99.35%	MH750210.1

Description	Max Score	Total Score	Query Cover	E value	Per. Ident	Accession
Equisetum environmental sample isolate EDNA16-0043491b ribulose-1,5-bisphosphate carboxylase/oxygenase large subunit (rbcL) gene, partial cds; chloroplast	1103	1103	95%	0.0	99.03%	MT197548.1
Equisetum telmateia ribulose-1,5-bisphosphate carboxylase large subunit (rbcL) gene, partial cds; chloroplast gene for chloroplast product	1101	1101	94%	0.0	99.34%	AF313580.1
Equisetum sylvaticum isolate 41081 ribulose-1,5-bisphosphate carboxylase/oxygenase large subunit (rbcL) gene, partial cds; plastid	1098	1098	93%	0.0	99.67%	MH750205.1
Equisetum arvense subsp. boreale isolate 41074 ribulose-1,5-bisphosphate carboxylase/oxygenase large subunit (rbcL) gene, partial cds; plastid	1098	1098	95%	0.0	99.18%	MH750160.1
Equisetum diffusum ribulose-1,5-bisphosphate carboxylase/oxygenase large subunit (rbcL) gene, partial cds; chloroplast gene for chloroplast product	1098	1098	95%	0.0	98.86%	AY226141.1
Equisetum x dycei isolate 26083 ribulose-1,5-bisphosphate carboxylase/oxygenase large subunit (rbcL) gene, partial cds; plastid	1096	1096	94%	0.0	99.18%	MH750220.1
Equisetum fluviatile isolate 41076 ribulose-1,5-bisphosphate carboxylase/oxygenase large subunit (rbcL) gene, partial cds; plastid	1094	1094	94%	0.0	99.18%	MH750169.1
Equisetum fluviatile isolate 41075 ribulose-1,5-bisphosphate carboxylase/oxygenase large subunit (rbcL) gene, partial cds; plastid	1094	1094	94%	0.0	99.18%	MH750168.1
Equisetum arvense voucher AP400 (OAC) ribulose-1,5-bisphosphate carboxylase/oxygenase large subunit (rbcL) gene, partial cds; chloroplast	1094	1094	94%	0.0	99.18%	EU677105.1
Equisetum pratense isolate 41087 ribulose-1,5-bisphosphate carboxylase/oxygenase large subunit (rbcL) gene, partial cds; plastid	1092	1092	93%	0.0	99.50%	MH750195.1
Equisetum telmateia subsp. braunii isolate 40828 ribulose-1,5-bisphosphate carboxylase/oxygenase large subunit (rbcL) gene, partial cds; plastid	1086	1086	93%	0.0	99.33%	MH750207.1
Equisetum arvense subsp. boreale isolate 41073 ribulose-1,5-bisphosphate carboxylase/oxygenase large subunit (rbcL) gene, partial cds; plastid	1086	1086	94%	0.0	99.17%	MH750159.1
Equisetum pratense voucher AP451 ribulose-1,5-bisphosphate carboxylase/oxygenase large subunit (rbcL) gene, partial cds; chloroplast	1086	1086	94%	0.0	98.85%	HQ590083.1
Equisetum x fontqueri isolate 26093 ribulose-1,5-bisphosphate carboxylase/oxygenase large subunit (rbcL) gene, partial cds; plastid	1083	1083	93%	0.0	99.33%	MH750221.1
Equisetum palustre isolate 17671 ribulose-1,5-bisphosphate carboxylase/oxygenase large subunit (rbcL) gene, partial cds; plastid	1081	1081	91%	0.0	99.83%	MH750191.1

Description	Max Score	Total Score	Query Cover	E value	Per. Ident	Accession
Equisetum palustre isolate 39349 ribulose-1,5-bisphosphate carboxylase/oxygenase large subunit (rbcL) gene, partial cds; plastid	1079	1079	91%	0.0	99.83%	MH750192.1
Equisetum arvense subsp. arvense isolate 41071 ribulose-1,5-bisphosphate carboxylase/oxygenase large subunit (rbcL) gene, partial cds; plastid	1079	1079	93%	0.0	99.17%	MH750158.1
Equisetum x litorale isolate 41084 ribulose-1,5-bisphosphate carboxylase/oxygenase large subunit (rbcL) gene, partial cds; plastid	1074	1074	92%	0.0	99.16%	MH750222.1
Equisetum telmateia subsp. braunii isolate 40832 ribulose-1,5-bisphosphate carboxylase/oxygenase large subunit (rbcL) gene, partial cds; plastid	1074	1074	92%	0.0	99.33%	MH750208.1
Equisetum pratense isolate 41086 ribulose-1,5-bisphosphate carboxylase/oxygenase large subunit (rbcL) gene, partial cds; plastid	1072	1072	91%	0.0	99.49%	MH750194.1
Equisetum pratense isolate 39348 ribulose-1,5-bisphosphate carboxylase/oxygenase large subunit (rbcL) gene, partial cds; plastid	1068	1068	91%	0.0	99.49%	MH750193.1
Equisetum arvense subsp. arvense isolate 26085 ribulose-1,5-bisphosphate carboxylase/oxygenase large subunit (rbcL) gene, partial cds; plastid	1068	1068	92%	0.0	99.16%	MH750156.1
Equisetum diffusum isolate 40689 ribulose-1,5-bisphosphate carboxylase/oxygenase large subunit (rbcL) gene, partial cds; plastid	1062	1062	92%	0.0	98.99%	MH750166.1
Equisetum arvense subsp. arvense isolate 26084 ribulose-1,5-bisphosphate carboxylase/oxygenase large subunit (rbcL) gene, partial cds; plastid	1061	1061	91%	0.0	99.15%	MH750155.1
Equisetum xylochaetum chloroplast, complete genome	1061	1061	99%	0.0	96.57%	MW282958.1
Equisetum hyemale chloroplast, complete genome	1055	1055	99%	0.0	96.41%	KC117177.1
Equisetum arvense subsp. arvense isolate 40833 ribulose-1,5-bisphosphate carboxylase/oxygenase large subunit (rbcL) gene, partial cds; plastid	1053	1053	91%	0.0	99.15%	MH750157.1
Equisetum scirpoides ribulose-1,5-bisphosphate carboxylase/oxygenase large subunit (rbcL) gene, partial cds; chloroplast gene for chloroplast product	1053	1053	95%	0.0	97.56%	AY226133.1
Equisetum diffusum isolate 40804 ribulose-1,5-bisphosphate carboxylase/oxygenase large subunit (rbcL) gene, partial cds; plastid	1050	1050	91%	0.0	98.81%	MH750167.1
Equisetum sylvaticum voucher TJD-502 ribulose-1,5-bisphosphate carboxylase/oxygenase large subunit (rbcL) gene, partial cds; chloroplast	1046	1046	88%	0.0	99.82%	KJ841305.1

Description	Max Score	Total Score	Query Cover	E value	Per. Ident	Accession
Equisetum palustre chloroplast rbcL gene for ribulose-1,5-bisphosphate carboxylase/oxygenase large subunit, partial cds, specimen_voucher: TNS:776981	1046	1046	88%	0.0	99.82%	AB574686.1
Equisetum ramosissimum isolate BPTPS123 voucher LISE:96444 chloroplast, complete genome	1044	1044	99%	0.0	96.10%	ON641349.1
Equisetum ramosissimum isolate BPTPS056 voucher LISE:96334 chloroplast, complete genome	1044	1044	99%	0.0	96.10%	NC_062377.1
Equisetum ramosissimum isolate BPTPS056 voucher LISE:96334 chloroplast, complete genome	1044	1044	99%	0.0	96.10%	OM691674.1
Equisetum scirpoides voucher AP248 ribulose-1,5-bisphosphate carboxylase/oxygenase large subunit (rbcL) gene, partial cds; chloroplast	1044	1044	94%	0.0	97.70%	HQ590084.1
Equisetum fluviatile ribulose-1,5-bisphosphate carboxylase/oxygenase large subunit (rbcL) gene, partial cds; chloroplast	1044	1044	90%	0.0	99.14%	DQ463101.1
Equisetum arvense x Equisetum telmateia subsp. braunii isolate 40834 ribulose-1,5-bisphosphate carboxylase/oxygenase large subunit (rbcL) gene, partial cds; plastid	1042	1042	90%	0.0	99.14%	MH750161.1
Equisetum arvense ribulose-1,5-bisphosphate carboxylase/oxygenase large subunit (rbcL) gene, partial cds; chloroplast	1040	1040	90%	0.0	98.97%	KM218345.1
Equisetum sylvaticum chloroplast rbcL gene for ribulose-1,5-bisphosphate carboxylase/oxygenase large subunit, partial cds, specimen_voucher: TNS:776974	1040	1040	88%	0.0	99.65%	AB574690.1
Equisetum hyemale ribulose-1,5-bisphosphate carboxylase/oxygenase large subunit (rbcL) gene, partial cds; chloroplast	1040	1040	99%	0.0	95.94%	DQ646001.1
Equisetum arvense voucher TJD-508 ribulose-1,5-bisphosphate carboxylase/oxygenase large subunit (rbcL) gene, partial cds; chloroplast	1037	1037	88%	0.0	99.47%	KJ841301.1
Equisetum arvense voucher TJD-503 ribulose-1,5-bisphosphate carboxylase/oxygenase large subunit (rbcL) gene, partial cds; chloroplast	1035	1035	88%	0.0	99.47%	KJ841302.1
Equisetum pratense chloroplast rbcL gene for ribulose-1,5-bisphosphate carboxylase/oxygenase large subunit, partial cds, specimen_voucher: TNS:1107843	1035	1035	88%	0.0	99.47%	AB574687.1
Equisetum arvense voucher TJD-504 ribulose-1,5-bisphosphate carboxylase/oxygenase large subunit (rbcL) gene, partial cds; chloroplast	1033	1033	88%	0.0	99.47%	KJ841300.1
Equisetum scirpoides isolate 41089 ribulose-1,5-bisphosphate carboxylase/oxygenase large subunit (rbcL) gene, partial cds; plastid	1031	1031	93%	0.0	97.67%	MH750203.1

Description	Max Score	Total Score	Query Cover	E value	Per. Ident	Accession
Equisetum komarovii isolate 40830 ribulose-1,5-bisphosphate carboxylase/oxygenase large subunit (rbcL) gene, partial cds; plastid	1031	1031	93%	0.0	97.67%	MH750182.1
Equisetum bogotense ribulose-1,5-bisphosphate carboxylase/oxygenase large subunit (rbcL) gene, partial cds; chloroplast gene for chloroplast product	1029	1029	95%	0.0	96.91%	AY226139.1
Equisetum sylvaticum plastid partial rbcL gene for ribulose-1,5-biphosphate carboxylase/oxygenase large subunit, specimen voucher H823764	1027	1027	87%	0.0	99.82%	HE574610.1
Commelina diffusa voucher Q555 ribulose-1,5-bisphosphate carboxylase/oxygenase large subunit (rbcL) gene, partial cds; chloroplast	1026	1026	88%	0.0	99.29%	MH714073.1
Equisetum myriochaetum ribulose-1,5-bisphosphate carboxylase/oxygenase large subunit (rbcL) gene, partial cds; chloroplast gene for chloroplast product	1026	1026	95%	0.0	96.75%	AY226131.1
Equisetum giganteum ribulose-1,5-bisphosphate carboxylase/oxygenase large subunit (rbcL) gene, partial cds; chloroplast gene for chloroplast product	1026	1026	95%	0.0	96.75%	AY226127.1
Equisetum sp. Schuettpelz 1643 ribulose-1,5-bisphosphate carboxylase/oxygenase large subunit (rbcL) gene, partial cds; chloroplast	1024	1024	95%	0.0	96.75%	MW620286.1
Equisetum fluviatile chloroplast rbcL gene for ribulose-1,5-bisphosphate carboxylase/oxygenase large subunit, partial cds, specimen_voucher: TNS:776384	1024	1024	88%	0.0	99.12%	AB574684.1
Equisetum palustre voucher Chase 11641 (K) ribulose-1,5-bisphosphate carboxylase/oxygenase large subunit (rbcL) gene, partial cds; chloroplast	1022	1022	86%	0.0	100.00%	GQ248601.1
Equisetum laevigatum ribulose-1,5-bisphosphate carboxylase/oxygenase large subunit (rbcL) gene, partial cds; chloroplast gene for chloroplast product	1022	1022	95%	0.0	96.74%	AY226130.1
Equisetum scirpoides isolate 26090 ribulose-1,5-bisphosphate carboxylase/oxygenase large subunit (rbcL) gene, partial cds; plastid	1020	1020	93%	0.0	97.34%	MH750202.1
Equisetum palustre voucher Gillespie_8830b ribulose-1,5-bisphosphate carboxylase/oxygenase large subunit (rbcL) gene, partial cds; chloroplast	1020	1020	85%	0.0	100.00%	KC482730.1
Equisetum hyemale subsp. affine voucher OAC 94916 ribulose-1,5-bisphosphate carboxylase/oxygenase large subunit (rbcL) gene, partial cds; chloroplast	1020	1020	95%	0.0	96.59%	KF186500.1
Equisetum hyemale voucher AP344 (OAC) ribulose-1,5-bisphosphate carboxylase/oxygenase large subunit (rbcL) gene, partial cds; chloroplast	1020	1020	95%	0.0	96.59%	EU677107.1

Description	Max Score	Total Score	Query Cover	E value	Per. Ident	Accession
Equisetum hyemale subsp. affine ribulose-1,5-bisphosphate carboxylase/oxygenase large subunit (rbcL) gene, partial cds; chloroplast gene for chloroplast product	1020	1020	95%	0.0	96.59%	AY226128.1
Equisetum giganteum isolate 40806 ribulose-1,5-bisphosphate carboxylase/oxygenase large subunit (rbcL) gene, partial cds; plastid	1018	1018	95%	0.0	96.73%	MH750172.1
Equisetum giganteum isolate 26087 ribulose-1,5-bisphosphate carboxylase/oxygenase large subunit (rbcL) gene, partial cds; plastid	1018	1018	95%	0.0	96.73%	MH750170.1
Equisetum ramosissimum voucher R825 ribulose-1,5-bisphosphate carboxylase/oxygenase large subunit (rbcl) gene, partial cds; chloroplast	1018	1018	96%	0.0	96.30%	KY656709.1
Equisetum arvense chloroplast rbcL gene for ribulose-1,5-bisphosphate carboxylase/oxygenase large subunit, partial cds, specimen_voucher: TNS:763618	1018	1018	88%	0.0	98.95%	AB574683.1
Equisetum x ferrissii ribulose-1,5-bisphosphate carboxylase/oxygenase large subunit (rbcL) gene, partial cds; chloroplast	1018	1018	95%	0.0	96.73%	EU352292.1
Equisetum sylvaticum voucher WAB_1079140180 ribulose-1,5-bisphosphate carboxylase/oxygenase large subunit (rbcL) gene, partial cds; chloroplast	1016	1016	86%	0.0	99.82%	KJ593395.1
Equisetum pratense voucher WAB_0132469185 ribulose-1,5-bisphosphate carboxylase/oxygenase large subunit (rbcL) gene, partial cds; chloroplast	1016	1016	86%	0.0	99.82%	KJ593391.1
Equisetum x ferrissii ribulose-1,5-bisphosphate carboxylase large subunit (rbcL) gene, partial cds; chloroplast gene for chloroplast product	1016	1016	95%	0.0	96.73%	AF313579.1
Equisetum sylvaticum voucher Gillespie_8524 ribulose-1,5-bisphosphate carboxylase/oxygenase large subunit (rbcL) gene, partial cds; chloroplast	1014	1014	85%	0.0	99.82%	KC482739.1
Equisetum scirpoides isolate 10933 ribulose-1,5-bisphosphate carboxylase/oxygenase large subunit (rbcL) gene, partial cds; plastid	1011	1011	91%	0.0	97.63%	MH750201.1
Equisetum palustre ribulose-1,5-bisphosphate carboxylase/oxygenase large subunit (rbcL) gene, partial cds; chloroplast	1009	1009	85%	0.0	100.00%	MK525785.1
Equisetum telmateia voucher CCDB-18287-A07 ribulose-1,5-bisphosphate carboxylase/oxygenase large subunit (rbcL) gene, partial cds; chloroplast	1009	1009	85%	0.0	99.64%	MG215015.1
Equisetum ramosissimum subsp. debile voucher G.M. Plunkett 3642 (NYBG) ribulose-1,5-bisphosphate carboxylase/oxygenase large subunit (rbcL) gene, partial cds; chloroplast	1009	1009	95%	0.0	96.27%	MT657901.1

Description	Max Score	Total Score	Query Cover	E value	Per. Ident	Accession
Equisetum ramosissimum subsp. debile ribulose-1,5-bisphosphate carboxylase/oxygenase large subunit (rbcL) gene, partial cds; chloroplast gene for chloroplast product	1009	1009	95%	0.0	96.27%	AY226132.1
Equisetum myriochaetum isolate 40815 ribulose-1,5-bisphosphate carboxylase/oxygenase large subunit (rbcL) gene, partial cds; plastid	1007	1007	94%	0.0	96.55%	MH750185.1
Equisetum arvense voucher WAB_1079140221 ribulose-1,5-bisphosphate carboxylase/oxygenase large subunit (rbcL) gene, partial cds; chloroplast	1005	1005	86%	0.0	99.46%	KJ593388.1
Equisetum arvense subsp. alpestre voucher Gillespie_10109_CAN ribulose-1,5-bisphosphate carboxylase/oxygenase large subunit (rbcL) gene, partial cds; chloroplast	1005	1005	86%	0.0	99.46%	KC482713.1
Equisetum sylvaticum ribulose-1,5-bisphosphate carboxylase/oxygenase large subunit (rbcL) gene, partial cds; chloroplast	1003	1003	85%	0.0	99.82%	MK525787.1
Equisetum pratense ribulose-1,5-bisphosphate carboxylase/oxygenase large subunit (rbcL) gene, partial cds; chloroplast	1003	1003	85%	0.0	99.82%	MK525786.1
Equisetum arvense ribulose-1,5-bisphosphate carboxylase/oxygenase large subunit (rbcL) gene, partial cds; chloroplast	1003	1003	85%	0.0	99.46%	MZ359289.1
Equisetum fluviatile voucher 09PROBE-05547 ribulose-1,5-bisphosphate carboxylase/oxygenase large subunit (rbcL) gene, partial cds; chloroplast	1003	1003	85%	0.0	99.46%	JN965528.1
Equisetum arvense voucher BIOUG24048-F01 ribulose-1,5-bisphosphate carboxylase/oxygenase large subunit (rbcL) gene, partial cds; chloroplast	1002	1002	85%	0.0	99.46%	KT695464.1
Equisetum xylochaetum isolate 40614 ribulose-1,5-bisphosphate carboxylase/oxygenase large subunit (rbcL) gene, partial cds; plastid	998	998	93%	0.0	96.67%	MH750228.1
Equisetum myriochaetum isolate 41080 ribulose-1,5-bisphosphate carboxylase/oxygenase large subunit (rbcL) gene, partial cds; plastid	998	998	93%	0.0	96.67%	MH750190.1
Equisetum myriochaetum isolate 40826 ribulose-1,5-bisphosphate carboxylase/oxygenase large subunit (rbcL) gene, partial cds; plastid	998	998	93%	0.0	96.67%	MH750188.1
Equisetum arvense voucher G00199177 ribulose-1,5-bisphosphate carboxylase/oxygenase large subunit (rbcL) gene, partial cds; chloroplast	998	998	85%	0.0	99.45%	KJ204336.1

Alignments

Equisetum palustre chloroplast, partial genome

Sequence ID: **MT984458.1** Length: 5501 Number of Matches: 1

Range 1: 2214 to 2853

Score	Expect	Identities	Gaps	Strand	Frame
1166 bits(631)	0.0()	638/641(99%)	1/641(0%)	Plus/Plus	

Features:

```
Query  2     TGTCACCACAAACAGAGACTAAAGCAGGTGTTGGATTTAAAGCTGGTGTTAAAGATTATC  61
             ||||||||||||| ||||| ||||||||||||||||||||||||||||||||||||||||
Sbjct  2214  TGTCACCACAAACGGAGACCAAAGCAGGTGTTGGATTTAAAGCTGGTGTTAAAGATTATC  2273

Query  62    GATTGACTTATTTTACTCCAGATTATGAAACCAAAGATACCGATATTTTAGCAGCATTCC  121
             ||||||||||||||||||||||||||||||||||||||||||||||||||||||||||||
Sbjct  2274  GATTGACTTATTTTACTCCAGATTATGAAACCAAAGATACCGATATTTTAGCAGCATTCC  2333

Query  122   GTATGACTCCTCAACCGGGAGTACCACCGGAAGAAGCAGGAGCAGCCGTAGCTGCTGAAT  181
             ||||||||||||||||||||||||||||||||||||||||||||||||||||||||||||
Sbjct  2334  GTATGACTCCTCAACCGGGAGTACCACCGGAAGAAGCAGGAGCAGCCGTAGCTGCTGAAT  2393

Query  182   CGTCCACGGGCACCTGGACTACCGTATGGACAGATGGACTTACTAGTCTTGATCGATATA  241
             ||||||||||||||||||||||||||||||||||||||||||||||||||||||||||||
Sbjct  2394  CGTCCACGGGCACCTGGACTACCGTATGGACAGATGGACTTACTAGTCTTGATCGATATA  2453

Query  242   AAGGTCGATGCTATAATATTGAACCTGTTGCTGGAGAAGATAATCAATTCATAGCTTATG  301
             ||||||||||||||||||||||||||||||||||||||||||||||||||||||||||||
Sbjct  2454  AAGGTCGATGCTATAATATTGAACCTGTTGCTGGAGAAGATAATCAATTCATAGCTTATG  2513

Query  302   TAGCCTATCCTTTAGATCTTTTTGAAGAAGGTTCTGTTACCAATCTTTTTACTTCAATTG  361
             ||||||||||||||||||||||||||||||||||||||||||||||||||||||||||||
Sbjct  2514  TAGCCTATCCTTTAGATCTTTTTGAAGAAGGTTCTGTTACCAATCTTTTTACTTCAATTG  2573

Query  362   TTGGTAATGTTTTCGGCTTCAAAGCTCTACGTGCTTTACGTTTAGAAGATTTAAGAATTC  421
             ||||||||||||||||||||||||||||||||||||||||||||||||||||||||||||
Sbjct  2574  TTGGTAATGTTTTCGGCTTCAAAGCTCTACGTGCTTTACGTTTAGAAGATTTAAGAATTC  2633

Query  422   CTCCTGCTTACTCCAAAACTTTTATAGGACCACCCCACGGTATCCAGGTTGAAAGAGATA  481
             ||||||||||||||||||||||||||||||||||||||||||||||||||||||||||||
Sbjct  2634  CTCCTGCTTACTCCAAAACTTTTATAGGACCACCCCACGGTATCCAGGTTGAAAGAGATA  2693

Query  482   AGTTAAACAAATATGGTCGTCCTTTATTAGGTTGTACAATTAAACCAAAATTGGGACTAT  541
             ||||||||||||||||||||||||||||||||||||||||||||||||||||||||||||
Sbjct  2694  AGTTAAACAAATATGGTCGTCCTTTATTAGGTTGTACAATTAAACCAAAATTGGGACTAT  2753

Query  542   CTGCTAAAAACTATGGTAGGGCTGTTTATGAATGTCTTCGTGGTGGACTTGATTTCACCC  601
             ||||||||||||||||||||||||||||||||||||||||||||||||||||||||| ||
Sbjct  2754  CTGCTAAAAACTATGGTAGGGCTGTTTATGAATGTCTTCGTGGTGGACTTGATTTCA-CC  2812

Query  602   AAAGATGATGAGAATGTAAACTCTCAACCCTTTATGCGTTG  642
             |||||||||||||||||||||||||||||||||||||||||
Sbjct  2813  AAAGATGATGAGAATGTAAACTCTCAACCCTTTATGCGTTG  2853
```

Equisetum arvense chloroplast, complete genome

Sequence ID: **GU191334.1** Length: 133309 Number of Matches: 1

Range 1: 27331 to 27970

Score	Expect	Identities	Gaps	Strand	Frame
1144 bits(619)	0.0()	634/641(99%)	1/641(0%)	Plus/Minus	

Features:

```
Query  2      TGTCACCACAAACAGAGACTAAAGCAGGTGTTGGATTTAAAGCTGGTGTTAAAGATTATC  61
              ||||||||||||| ||||| ||||||||||||||||||||||||||||||||||||||||
Sbjct  27970  TGTCACCACAAACGGAGACCAAAGCAGGTGTTGGATTTAAAGCTGGTGTTAAAGATTATC  27911

Query  62     GATTGACTTATTTTACTCCAGATTATGAAACCAAAGATACCGATATTTTAGCAGCATTCC  121
              ||||||||||||||||||||||||||||||||||||||||||||||||||||||||||||
Sbjct  27910  GATTGACTTATTTTACTCCAGATTATGAAACCAAAGATACCGATATTTTAGCAGCATTCC  27851

Query  122    GTATGACTCCTCAACCGGGAGTACCACCGGAAGAAGCAGGAGCAGCCGTAGCTGCTGAAT  181
              ||||||||||||||||||||||||||||||||||||||||||||||||||||||||||||
Sbjct  27850  GTATGACTCCTCAACCGGGAGTACCACCGGAAGAAGCAGGAGCAGCCGTAGCTGCTGAAT  27791

Query  182    CGTCCACGGGCACCTGGACTACCGTATGGACAGATGGACTTACTAGTCTTGATCGATATA  241
              ||||||||||||||||||||||||||||||||||||||||||||||||||||||||||||
Sbjct  27790  CGTCCACGGGCACCTGGACTACCGTATGGACAGATGGACTTACTAGTCTTGATCGATATA  27731

Query  242    AAGGTCGATGCTATAATATTGAACCTGTTGCTGGAGAAGATAATCAATTCATAGCTTATG  301
              ||||||||||||||||||||||||||||||||||||||||||||||||||||||||||||
Sbjct  27730  AAGGTCGATGCTATAATATTGAACCTGTTGCTGGAGAAGATAATCAATTCATAGCTTATG  27671
```

```
Query  302    TAGCCTATCCTTTAGATCTTTTTGAAGAAGGTTCTGTTACCAATCTTTTTACTTCAATTG  361
              ||||||| ||||||||||||||||||||||||||||||||||||||||||||||||||||
Sbjct  27670  TAGCCTACCCTTTAGATCTTTTTGAAGAAGGTTCTGTTACCAATCTTTTTACTTCAATTG  27611

Query  362    TTGGTAATGTTTTCGGCTTCAAAGCTCTACGTGCTTTACGTTTAGAAGATTTAAGAATTC  421
              ||||||||||||||||||||||||||||||||||||||||||||||||||||||||||||
Sbjct  27610  TTGGTAATGTTTTCGGCTTCAAAGCTCTACGTGCTTTACGTTTAGAAGATTTAAGAATTC  27551

Query  422    CTCCTGCTTACTCCAAAACTTTTATAGGACCACCCCACGGTATCCAGGTTGAAAGAGATA  481
              ||||||||||||||||||||||||||||||||||||||||||||||||||||||||||||
Sbjct  27550  CTCCTGCTTACTCCAAAACTTTTATAGGACCACCCCACGGTATCCAGGTTGAAAGAGATA  27491

Query  482    AGTTAAACAAATATGGTCGTCCTTTATTAGGTTGTACAATTAAACCAAAATTGGGACTAT  541
              ||||||||||||| ||||||||||||||||||||||||||||||||||||||||||||||
Sbjct  27490  AGTTAAACAAATACGGTCGTCCTTTATTAGGTTGTACAATTAAACCAAAATTGGGACTAT  27431

Query  542    CTGCTAAAAACTATGGTAGGGCTGTTTATGAATGTCTTCGTGGTGGACTTGATTTCACCC  601
              ||||||||||||||||||| |||||||||||||||||||||||||| |||||||||||| 
Sbjct  27430  CTGCTAAAAACTATGGTAGAGCTGTTTATGAATGTCTTCGTGGTGGGCTTGATTTCACC-  27372

Query  602    AAAGATGATGAGAATGTAAACTCTCAACCCTTTATGCGTTG  642
              |||||||||||||||||||||||||||||||||||||||||
Sbjct  27371  AAAGATGATGAGAATGTAAACTCTCAACCCTTTATGCGTTG  27331
```

Equisetum arvense chloroplast ribulosebisphosphate carboxylase (rbcL) gene, complete cds

Sequence ID: **L11053.1** Length: 1587 Number of Matches: 1

Range 1: 74 to 713

Score	Expect	Identities	Gaps	Strand	Frame
1144 bits(619)	0.0()	634/641(99%)	1/641(0%)	Plus/Plus	

Features:

```
Query  2      TGTCACCACAAACAGAGACTAAAGCAGGTGTTGGATTTAAAGCTGGTGTTAAAGATTATC  61
              ||||||||||||| ||||| ||||||||||||||||||||||||||||||||||||||||
Sbjct  74     TGTCACCACAAACGGAGACCAAAGCAGGTGTTGGATTTAAAGCTGGTGTTAAAGATTATC  133

Query  62     GATTGACTTATTTTACTCCAGATTATGAAACCAAAGATACCGATATTTTAGCAGCATTCC  121
              ||||||||||||||||||||||||||||||||||||||||||||||||||||||||||||
Sbjct  134    GATTGACTTATTTTACTCCAGATTATGAAACCAAAGATACCGATATTTTAGCAGCATTCC  193

Query  122    GTATGACTCCTCAACCGGGAGTACCACCGGAAGAAGCAGGAGCAGCCGTAGCTGCTGAAT  181
              ||||||||||||||||||||||||||||||||||||||||||||||||||||||||||||
Sbjct  194    GTATGACTCCTCAACCGGGAGTACCACCGGAAGAAGCAGGAGCAGCCGTAGCTGCTGAAT  253

Query  182    CGTCCACGGGCACCTGGACTACCGTATGGACAGATGGACTTACTAGTCTTGATCGATATA  241
              ||||||||||||||||||||||||||||||||||||||||||||||||||||||||||||
Sbjct  254    CGTCCACGGGCACCTGGACTACCGTATGGACAGATGGACTTACTAGTCTTGATCGATATA  313

Query  242    AAGGTCGATGCTATAATATTGAACCTGTTGCTGGAGAAGATAATCAATTCATAGCTTATG  301
              ||||||||||||||||||||||||||||||||||||||||||||||||||||||||||||
Sbjct  314    AAGGTCGATGCTATAATATTGAACCTGTTGCTGGAGAAGATAATCAATTCATAGCTTATG  373

Query  302    TAGCCTATCCTTTAGATCTTTTTGAAGAAGGTTCTGTTACCAATCTTTTTACTTCAATTG  361
              ||||||| ||||||||||||||||||||||||||||||||||||||||||||||||||||
Sbjct  374    TAGCCTACCCTTTAGATCTTTTTGAAGAAGGTTCTGTTACCAATCTTTTTACTTCAATTG  433

Query  362    TTGGTAATGTTTTCGGCTTCAAAGCTCTACGTGCTTTACGTTTAGAAGATTTAAGAATTC  421
              ||||||||||||||||||||||||||||||||||||||||||||||||||||||||||||
Sbjct  434    TTGGTAATGTTTTCGGCTTCAAAGCTCTACGTGCTTTACGTTTAGAAGATTTAAGAATTC  493

Query  422    CTCCTGCTTACTCCAAAACTTTTATAGGACCACCCCACGGTATCCAGGTTGAAAGAGATA  481
              ||||||||||||||||||||||||||||||||||||||||||||||||||||||||||||
Sbjct  494    CTCCTGCTTACTCCAAAACTTTTATAGGACCACCCCACGGTATCCAGGTTGAAAGAGATA  553

Query  482    AGTTAAACAAATATGGTCGTCCTTTATTAGGTTGTACAATTAAACCAAAATTGGGACTAT  541
              ||||||||||||| ||||||||||||||||||||||||||||||||||||||||||||||
Sbjct  554    AGTTAAACAAATACGGTCGTCCTTTATTAGGTTGTACAATTAAACCAAAATTGGGACTAT  613

Query  542    CTGCTAAAAACTATGGTAGGGCTGTTTATGAATGTCTTCGTGGTGGACTTGATTTCACCC  601
              ||||||||||||||||||| |||||||||||||||||||||||||| |||||||||| ||
Sbjct  614    CTGCTAAAAACTATGGTAGAGCTGTTTATGAATGTCTTCGTGGTGGGCTTGATTTCA-CC  672

Query  602    AAAGATGATGAGAATGTAAACTCTCAACCCTTTATGCGTTG  642
              |||||||||||||||||||||||||||||||||||||||||
Sbjct  673    AAAGATGATGAGAATGTAAACTCTCAACCCTTTATGCGTTG  713
```

Equisetum arvense plastid, complete genome

Sequence ID: **JN968380.1** Length: 132726 Number of Matches: 1

Range 1: 64959 to 65598

Score	Expect	Identities	Gaps	Strand	Frame

1138 bits(616)	0.0()	633/641(99%)	1/641(0%)	Plus/Plus	

Features:

```
Query  2      TGTCACCACAAACAGAGACTAAAGCAGGTGTTGGATTTAAAGCTGGTGTTAAAGATTATC  61
              ||||||||||||| ||||| ||||||||||||||||||||||||||||||||||||||||
Sbjct  64959  TGTCACCACAAACGGAGACCAAAGCAGGTGTTGGATTTAAAGCTGGTGTTAAAGATTATC  65018

Query  62     GATTGACTTATTTTACTCCAGATTATGAAACCAAAGATACCGATATTTTAGCAGCATTCC  121
              ||||||||||||||||||||||||||||||||||||||||||||||||||||||||||||
Sbjct  65019  GATTGACTTATTTTACTCCAGATTATGAAACCAAAGATACCGATATTTTAGCAGCATTCC  65078

Query  122    GTATGACTCCTCAACCGGGAGTACCACCGGAAGAAGCAGGAGCAGCCGTAGCTGCTGAAT  181
              ||||||||||||||||||||||||||||||||||||||||||||||||||||||||||||
Sbjct  65079  GTATGACTCCTCAACCGGGAGTACCACCGGAAGAAGCAGGAGCAGCCGTAGCTGCTGAAT  65138

Query  182    CGTCCACGGGCACCTGGACTACCGTATGGACAGATGGACTTACTAGTCTTGATCGATATA  241
              ||||||||||||||||||||||||||||||||||||||||||||||||||||||||||||
Sbjct  65139  CGTCCACGGGCACCTGGACTACCGTATGGACAGATGGACTTACTAGTCTTGATCGATATA  65198

Query  242    AAGGTCGATGCTATAATATTGAACCTGTTGCTGGAGAAGATAATCAATTCATAGCTTATG  301
              ||||||||||||||||||||||||||||||||||||||||||||||||||||||||||||
Sbjct  65199  AAGGTCGATGCTATAATATTGAACCTGTTGCTGGAGAAGATAATCAATTCATAGCTTATG  65258

Query  302    TAGCCTATCCTTTAGATCTTTTTGAAGAAGGTTCTGTTACCAATCTTTTTACTTCAATTG  361
              ||||||| ||||||||||||||||||||||||||||||||||||||||||||||||||||
Sbjct  65259  TAGCCTACCCTTTAGATCTTTTTGAAGAAGGTTCTGTTACCAATCTTTTTACTTCAATTG  65318

Query  362    TTGGTAATGTTTTCGGCTTCAAAGCTCTACGTGCTTTACGTTTAGAAGATTTAAGAATTC  421
              |||||||||||||||| |||||||||||||||||||||||||||||||||||||||||||
Sbjct  65319  TTGGTAATGTTTTCGGATTCAAAGCTCTACGTGCTTTACGTTTAGAAGATTTAAGAATTC  65378

Query  422    CTCCTGCTTACTCCAAAACTTTTATAGGACCACCCCACGGTATCCAGGTTGAAAGAGATA  481
              ||||||||||||||||||||||||||||||||||||||||||||||||||||||||||||
Sbjct  65379  CTCCTGCTTACTCCAAAACTTTTATAGGACCACCCCACGGTATCCAGGTTGAAAGAGATA  65438

Query  482    AGTTAAACAAATATGGTCGTCCTTTATTAGGTTGTACAATTAAACCAAAATTGGGACTAT  541
              ||||||||||||| ||||||||||||||||||||||||||||||||||||||||||||||
Sbjct  65439  AGTTAAACAAATACGGTCGTCCTTTATTAGGTTGTACAATTAAACCAAAATTGGGACTAT  65498

Query  542    CTGCTAAAAACTATGGTAGGGCTGTTTATGAATGTCTTCGTGGTGGACTTGATTTCACCC  601
              ||||||||||||||||||| |||||||||||||||||||||||||| |||||||||| ||
Sbjct  65499  CTGCTAAAAACTATGGTAGAGCTGTTTATGAATGTCTTCGTGGTGGGCTTGATTTCA-CC  65557

Query  602    AAAGATGATGAGAATGTAAACTCTCAACCCTTTATGCGTTG  642
              |||||||||||||||||||||||||||||||||||||||||
Sbjct  65558  AAAGATGATGAGAATGTAAACTCTCAACCCTTTATGCGTTG  65598
```

Equisetum palustre ribulose-1,5-bisphosphate carboxylase/oxygenase large subunit (rbcL) gene, partial cds; chloroplast gene for chloroplast product

Sequence ID: **AY226138.1** Length: 1325 Number of Matches: 1

Range 1: 1 to 615

Score	Expect	Identities	Gaps	Strand	Frame
1131 bits(612)	0.0()	615/616(99%)	1/616(0%)	Plus/Plus	

Features:

```
Query  27   AGGTGTTGGATTTAAAGCTGGTGTTAAAGATTATCGATTGACTTATTTTACTCCAGATTA  86
            ||||||||||||||||||||||||||||||||||||||||||||||||||||||||||||
Sbjct  1    AGGTGTTGGATTTAAAGCTGGTGTTAAAGATTATCGATTGACTTATTTTACTCCAGATTA  60

Query  87   TGAAACCAAAGATACCGATATTTTAGCAGCATTCCGTATGACTCCTCAACCGGGAGTACC  146
            ||||||||||||||||||||||||||||||||||||||||||||||||||||||||||||
Sbjct  61   TGAAACCAAAGATACCGATATTTTAGCAGCATTCCGTATGACTCCTCAACCGGGAGTACC  120

Query  147  ACCGGAAGAAGCAGGAGCAGCCGTAGCTGCTGAATCGTCCACGGGCACCTGGACTACCGT  206
            ||||||||||||||||||||||||||||||||||||||||||||||||||||||||||||
Sbjct  121  ACCGGAAGAAGCAGGAGCAGCCGTAGCTGCTGAATCGTCCACGGGCACCTGGACTACCGT  180

Query  207  ATGGACAGATGGACTTACTAGTCTTGATCGATATAAAGGTCGATGCTATAATATTGAACC  266
            ||||||||||||||||||||||||||||||||||||||||||||||||||||||||||||
Sbjct  181  ATGGACAGATGGACTTACTAGTCTTGATCGATATAAAGGTCGATGCTATAATATTGAACC  240

Query  267  TGTTGCTGGAGAAGATAATCAATTCATAGCTTATGTAGCCTATCCTTTAGATCTTTTTGA  326
            ||||||||||||||||||||||||||||||||||||||||||||||||||||||||||||
Sbjct  241  TGTTGCTGGAGAAGATAATCAATTCATAGCTTATGTAGCCTATCCTTTAGATCTTTTTGA  300

Query  327  AGAAGGTTCTGTTACCAATCTTTTTACTTCAATTGTTGGTAATGTTTTCGGCTTCAAAGC  386
            ||||||||||||||||||||||||||||||||||||||||||||||||||||||||||||
Sbjct  301  AGAAGGTTCTGTTACCAATCTTTTTACTTCAATTGTTGGTAATGTTTTCGGCTTCAAAGC  360

Query  387  TCTACGTGCTTTACGTTTAGAAGATTTAAGAATTCCTCCTGCTTACTCCAAAACTTTTAT  446
            ||||||||||||||||||||||||||||||||||||||||||||||||||||||||||||
Sbjct  361  TCTACGTGCTTTACGTTTAGAAGATTTAAGAATTCCTCCTGCTTACTCCAAAACTTTTAT  420
```

```
Query  447   AGGACCACCCCACGGTATCCAGGTTGAAAGAGATAAGTTAAACAAATATGGTCGTCCTTT  506
             ||||||||||||||||||||||||||||||||||||||||||||||||||||||||||||
Sbjct  421   AGGACCACCCCACGGTATCCAGGTTGAAAGAGATAAGTTAAACAAATATGGTCGTCCTTT  480

Query  507   ATTAGGTTGTACAATTAAACCAAAATTGGGACTATCTGCTAAAAACTATGGTAGGGCTGT  566
             ||||||||||||||||||||||||||||||||||||||||||||||||||||||||||||
Sbjct  481   ATTAGGTTGTACAATTAAACCAAAATTGGGACTATCTGCTAAAAACTATGGTAGGGCTGT  540

Query  567   TTATGAATGTCTTCGTGGTGGACTTGATTTCACCCAAAGATGATGAGAATGTAAACTCTC  626
             |||||||||||||||||||||||||||||||| |||||||||||||||||||||||||||
Sbjct  541   TTATGAATGTCTTCGTGGTGGACTTGATTTCA-CCAAAGATGATGAGAATGTAAACTCTC  599

Query  627   AACCCTTTATGCGTTG  642
             ||||||||||||||||
Sbjct  600   AACCCTTTATGCGTTG  615
```

BLAST is a registered trademark of the National Library of Medicine

Support center Mailing list YouTube

- National Library Of Medicine
- National Institutes Of Health
- U.S. Department of Health & Human Services
- USA.gov

NCBI

National Center for Biotechnology Information, U.S. National Library of Medicine 8600 Rockville Pike, Bethesda MD, 20894 USA

Policies and Guidelines | Contact

HHS Vulnerability Disclosure
