# Supplementary material for: A Multi-Pronged Technique for Identifying Equisetum palustre and Equisetum arvense—Combining HPTLC, HPLC-ESI-MS/MS and Optimized DNA Barcoding Techniques
Source: Plants (Basel). 2022 Sep 28;11(19):2562. doi: 10.3390/plants11192562 (PMC9573248; doi:10.3390/plants11192562)
Supplement: Supplementary file 1 [file plants-11-02562-s001.zip › plants-1932149 supplementary/Horsetail Supplementary Figures/Figure S22 (P4, 82710220323-1).pdf]

BLAST Results

Questions/comments

Job title: Nucleotide Sequence

**RID**	J1C0R3WB013 (Expires on 09-15 00:30 am)
**Query ID**	lcl|Query_58815
**Description**	None
**Molecule type**	dna
**Query Length**	671
**Database Name**	nt
**Description**	Nucleotide collection (nt)
**Program**	BLASTN 2.13.0+

Graphic Summary

Distribution of the top 100 Blast Hits on 100 subject sequences

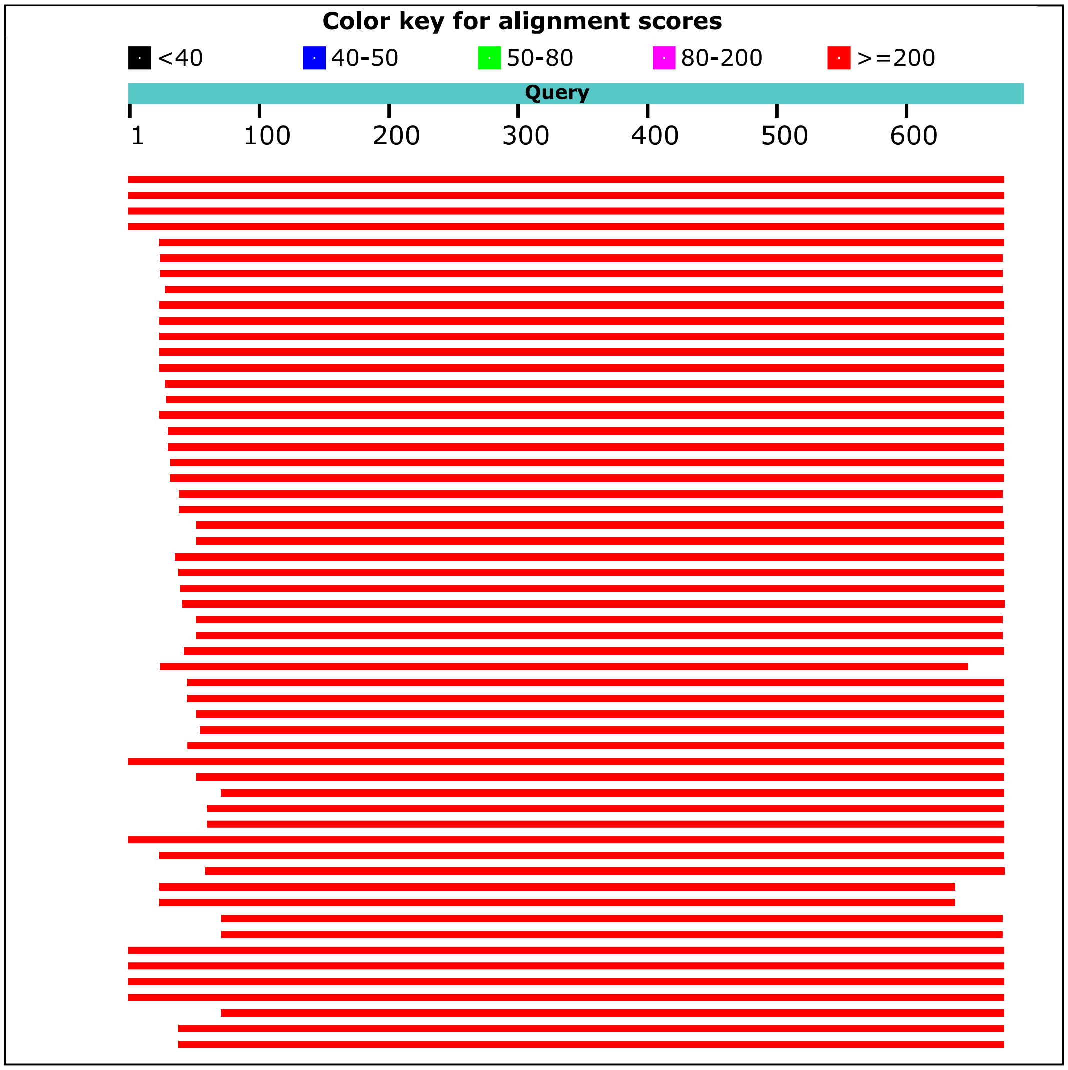

Descriptions

Sequences producing significant alignments:

Description	Max Score	Total Score	Query Cover	E value	Per. Ident	Accession
Equisetum palustre chloroplast, partial genome	1201	1201	99%	0.0	99.10%	MT984458.1
Equisetum arvense chloroplast, complete genome	1179	1179	99%	0.0	98.51%	GU191334.1
Equisetum arvense chloroplast ribulosebisphosphate carboxylase (rbcL) gene, complete cds	1179	1179	99%	0.0	98.51%	L11053.1
Equisetum arvense plastid, complete genome	1173	1173	99%	0.0	98.36%	JN968380.1
Equisetum palustre ribulose-1,5-bisphosphate carboxylase/oxygenase large subunit (rbcL) gene, partial cds; chloroplast gene for chloroplast product	1168	1168	96%	0.0	99.38%	AY226138.1
Equisetum pratense ribulose-1,5-bisphosphate carboxylase/oxygenase large subunit (rbcL) gene, partial cds; chloroplast gene for chloroplast product	1155	1155	95%	0.0	99.07%	AY226137.1
Equisetum sylvaticum ribulose-1,5-bisphosphate carboxylase/oxygenase large subunit (rbcL) gene, partial cds; chloroplast gene for chloroplast product	1155	1155	95%	0.0	99.07%	AY226136.1
Equisetum sylvaticum isolate 11640 ribulose-1,5-bisphosphate carboxylase/oxygenase large subunit (rbcL) gene, partial cds; plastid	1147	1147	95%	0.0	99.06%	MH750204.1
Equisetum environmental sample isolate EDNA16-0043490 ribulose-1,5-bisphosphate carboxylase/oxygenase large subunit (rbcL) gene, partial cds; chloroplast	1146	1146	96%	0.0	98.76%	MT197555.1
Equisetum fluviatile ribulose-1,5-bisphosphate carboxylase/oxygenase large subunit (rbcL) gene, partial cds; chloroplast gene for chloroplast product	1146	1146	96%	0.0	98.76%	AY226142.1
Equisetum arvense ribulose-1,5-bisphosphate carboxylase/oxygenase large subunit (rbcL) gene, partial cds; chloroplast gene for chloroplast product	1146	1146	96%	0.0	98.76%	AY226140.1
Equisetum telmateia subsp. braunii ribulose-1,5-bisphosphate carboxylase/oxygenase large subunit (rbcL) gene, partial cds; chloroplast gene for chloroplast product	1146	1146	96%	0.0	98.76%	AY226135.1
Equisetum environmental sample isolate EDNA16-0043491b ribulose-1,5-bisphosphate carboxylase/oxygenase large subunit (rbcL) gene, partial cds; chloroplast	1140	1140	96%	0.0	98.60%	MT197548.1
Equisetum telmateia subsp. telmateia isolate 11642 ribulose-1,5-bisphosphate carboxylase/oxygenase large subunit (rbcL) gene, partial cds; plastid	1138	1138	95%	0.0	98.75%	MH750210.1

Description	Max Score	Total Score	Query Cover	E value	Per. Ident	Accession
Equisetum arvense subsp. boreale isolate 41074 ribulose-1,5-bisphosphate carboxylase/oxygenase large subunit (rbcL) gene, partial cds; plastid	1134	1134	95%	0.0	98.75%	MH750160.1
Equisetum diffusum ribulose-1,5-bisphosphate carboxylase/oxygenase large subunit (rbcL) gene, partial cds; chloroplast gene for chloroplast product	1134	1134	96%	0.0	98.45%	AY226141.1
Equisetum x dycei isolate 26083 ribulose-1,5-bisphosphate carboxylase/oxygenase large subunit (rbcL) gene, partial cds; plastid	1133	1133	95%	0.0	98.75%	MH750220.1
Equisetum telmateia ribulose-1,5-bisphosphate carboxylase large subunit (rbcL) gene, partial cds; chloroplast gene for chloroplast product	1133	1133	95%	0.0	98.75%	AF313580.1
Equisetum fluviatile isolate 41076 ribulose-1,5-bisphosphate carboxylase/oxygenase large subunit (rbcL) gene, partial cds; plastid	1131	1131	94%	0.0	98.74%	MH750169.1
Equisetum fluviatile isolate 41075 ribulose-1,5-bisphosphate carboxylase/oxygenase large subunit (rbcL) gene, partial cds; plastid	1131	1131	94%	0.0	98.74%	MH750168.1
Equisetum sylvaticum isolate 41081 ribulose-1,5-bisphosphate carboxylase/oxygenase large subunit (rbcL) gene, partial cds; plastid	1127	1127	93%	0.0	99.05%	MH750205.1
Equisetum pratense isolate 41087 ribulose-1,5-bisphosphate carboxylase/oxygenase large subunit (rbcL) gene, partial cds; plastid	1127	1127	93%	0.0	99.05%	MH750195.1
Equisetum palustre isolate 39349 ribulose-1,5-bisphosphate carboxylase/oxygenase large subunit (rbcL) gene, partial cds; plastid	1127	1127	91%	0.0	99.68%	MH750192.1
Equisetum palustre isolate 17671 ribulose-1,5-bisphosphate carboxylase/oxygenase large subunit (rbcL) gene, partial cds; plastid	1127	1127	91%	0.0	99.68%	MH750191.1
Equisetum arvense subsp. boreale isolate 41073 ribulose-1,5-bisphosphate carboxylase/oxygenase large subunit (rbcL) gene, partial cds; plastid	1123	1123	94%	0.0	98.74%	MH750159.1
Equisetum telmateia subsp. braunii isolate 40828 ribulose-1,5-bisphosphate carboxylase/oxygenase large subunit (rbcL) gene, partial cds; plastid	1118	1118	93%	0.0	98.73%	MH750207.1
Equisetum arvense subsp. arvense isolate 41071 ribulose-1,5-bisphosphate carboxylase/oxygenase large subunit (rbcL) gene, partial cds; plastid	1116	1116	93%	0.0	98.73%	MH750158.1
Equisetum x fontqueri isolate 26093 ribulose-1,5-bisphosphate carboxylase/oxygenase large subunit (rbcL) gene, partial cds; plastid	1114	1114	93%	0.0	98.73%	MH750221.1
Equisetum pratense isolate 41086 ribulose-1,5-bisphosphate carboxylase/oxygenase large subunit (rbcL) gene, partial cds; plastid	1114	1114	91%	0.0	99.35%	MH750194.1

Description	Max Score	Total Score	Query Cover	E value	Per. Ident	Accession
Equisetum pratense isolate 39348 ribulose-1,5-bisphosphate carboxylase/oxygenase large subunit (rbcL) gene, partial cds; plastid	1114	1114	91%	0.0	99.35%	MH750193.1
Equisetum x litorale isolate 41084 ribulose-1,5-bisphosphate carboxylase/oxygenase large subunit (rbcL) gene, partial cds; plastid	1110	1110	93%	0.0	98.72%	MH750222.1
Equisetum arvense voucher JAG288 (OAC) ribulose-1,5-bisphosphate carboxylase/oxygenase large subunit (rbcL) gene, partial cds; chloroplast	1107	1107	92%	0.0	99.03%	EU677106.1
Equisetum telmateia subsp. braunii isolate 40832 ribulose-1,5-bisphosphate carboxylase/oxygenase large subunit (rbcL) gene, partial cds; plastid	1105	1105	92%	0.0	98.72%	MH750208.1
Equisetum arvense subsp. arvense isolate 26085 ribulose-1,5-bisphosphate carboxylase/oxygenase large subunit (rbcL) gene, partial cds; plastid	1105	1105	92%	0.0	98.72%	MH750156.1
Equisetum arvense subsp. arvense isolate 26084 ribulose-1,5-bisphosphate carboxylase/oxygenase large subunit (rbcL) gene, partial cds; plastid	1105	1105	91%	0.0	99.03%	MH750155.1
Equisetum arvense subsp. arvense isolate 40833 ribulose-1,5-bisphosphate carboxylase/oxygenase large subunit (rbcL) gene, partial cds; plastid	1101	1101	91%	0.0	99.02%	MH750157.1
Equisetum diffusum isolate 40689 ribulose-1,5-bisphosphate carboxylase/oxygenase large subunit (rbcL) gene, partial cds; plastid	1099	1099	92%	0.0	98.56%	MH750166.1
Equisetum xylochaetum chloroplast, complete genome	1096	1096	99%	0.0	96.26%	MW282958.1
Equisetum diffusum isolate 40804 ribulose-1,5-bisphosphate carboxylase/oxygenase large subunit (rbcL) gene, partial cds; plastid	1094	1094	91%	0.0	98.70%	MH750167.1
Equisetum palustre chloroplast rbcL gene for ribulose-1,5-bisphosphate carboxylase/oxygenase large subunit, partial cds, specimen_voucher: TNS:776981	1094	1094	89%	0.0	99.67%	AB574686.1
Equisetum fluviatile ribulose-1,5-bisphosphate carboxylase/oxygenase large subunit (rbcL) gene, partial cds; chloroplast	1092	1092	90%	0.0	99.01%	DQ463101.1
Equisetum arvense x Equisetum telmateia subsp. braunii isolate 40834 ribulose-1,5-bisphosphate carboxylase/oxygenase large subunit (rbcL) gene, partial cds; plastid	1090	1090	90%	0.0	99.01%	MH750161.1
Equisetum hyemale chloroplast, complete genome	1090	1090	99%	0.0	96.11%	KC117177.1
Equisetum scirpoides ribulose-1,5-bisphosphate carboxylase/oxygenase large subunit (rbcL) gene, partial cds; chloroplast gene for chloroplast product	1090	1090	96%	0.0	97.21%	AY226133.1

Description	Max Score	Total Score	Query Cover	E value	Per. Ident	Accession
Equisetum arvense ribulose-1,5-bisphosphate carboxylase/oxygenase large subunit (rbcL) gene, partial cds; chloroplast	1088	1088	90%	0.0	98.85%	KM218345.1
Equisetum arvense voucher AP400 (OAC) ribulose-1,5-bisphosphate carboxylase/oxygenase large subunit (rbcL) gene, partial cds; chloroplast	1088	1088	90%	0.0	99.01%	EU677105.1
Equisetum pratense voucher AP451 ribulose-1,5-bisphosphate carboxylase/oxygenase large subunit (rbcL) gene, partial cds; chloroplast	1081	1081	90%	0.0	98.68%	HQ590083.1
Equisetum sylvaticum chloroplast rbcL gene for ribulose-1,5-bisphosphate carboxylase/oxygenase large subunit, partial cds, specimen_voucher: TNS:776974	1081	1081	88%	0.0	99.33%	AB574690.1
Equisetum pratense chloroplast rbcL gene for ribulose-1,5-bisphosphate carboxylase/oxygenase large subunit, partial cds, specimen_voucher: TNS:1107843	1081	1081	88%	0.0	99.33%	AB574687.1
Equisetum ramosissimum isolate BPTPS123 voucher LISE:96444 chloroplast, complete genome	1079	1079	99%	0.0	95.81%	ON641349.1
Equisetum ramosissimum isolate BPTPS056 voucher LISE:96334 chloroplast, complete genome	1079	1079	99%	0.0	95.81%	NC_062377.1
Equisetum ramosissimum isolate BPTPS056 voucher LISE:96334 chloroplast, complete genome	1079	1079	99%	0.0	95.81%	OM691674.1
Equisetum hyemale ribulose-1,5-bisphosphate carboxylase/oxygenase large subunit (rbcL) gene, partial cds; chloroplast	1075	1075	99%	0.0	95.67%	DQ646001.1
Equisetum fluviatile chloroplast rbcL gene for ribulose-1,5-bisphosphate carboxylase/oxygenase large subunit, partial cds, specimen_voucher: TNS:776384	1072	1072	89%	0.0	99.00%	AB574684.1
Equisetum scirpoides isolate 41089 ribulose-1,5-bisphosphate carboxylase/oxygenase large subunit (rbcL) gene, partial cds; plastid	1068	1068	93%	0.0	97.30%	MH750203.1
Equisetum komarovii isolate 40830 ribulose-1,5-bisphosphate carboxylase/oxygenase large subunit (rbcL) gene, partial cds; plastid	1068	1068	93%	0.0	97.30%	MH750182.1
Equisetum arvense chloroplast rbcL gene for ribulose-1,5-bisphosphate carboxylase/oxygenase large subunit, partial cds, specimen_voucher: TNS:763618	1066	1066	89%	0.0	98.83%	AB574683.1
Equisetum bogotense ribulose-1,5-bisphosphate carboxylase/oxygenase large subunit (rbcL) gene, partial cds; chloroplast gene for chloroplast product	1064	1064	95%	0.0	96.58%	AY226139.1

Description	Max Score	Total Score	Query Cover	E value	Per. Ident	Accession
Equisetum myriochaetum ribulose-1,5-bisphosphate carboxylase/oxygenase large subunit (rbcL) gene, partial cds; chloroplast gene for chloroplast product	1062	1062	96%	0.0	96.43%	AY226131.1
Equisetum giganteum ribulose-1,5-bisphosphate carboxylase/oxygenase large subunit (rbcL) gene, partial cds; chloroplast gene for chloroplast product	1062	1062	96%	0.0	96.43%	AY226127.1
Equisetum sp. Schuettpelz 1643 ribulose-1,5-bisphosphate carboxylase/oxygenase large subunit (rbcL) gene, partial cds; chloroplast	1061	1061	95%	0.0	96.43%	MW620286.1
Equisetum laevigatum ribulose-1,5-bisphosphate carboxylase/oxygenase large subunit (rbcL) gene, partial cds; chloroplast gene for chloroplast product	1059	1059	95%	0.0	96.42%	AY226130.1
Equisetum scirpoides isolate 26090 ribulose-1,5-bisphosphate carboxylase/oxygenase large subunit (rbcL) gene, partial cds; plastid	1057	1057	93%	0.0	96.98%	MH750202.1
Equisetum hyemale subsp. affine voucher OAC 94916 ribulose-1,5-bisphosphate carboxylase/oxygenase large subunit (rbcL) gene, partial cds; chloroplast	1057	1057	96%	0.0	96.28%	KF186500.1
Equisetum hyemale subsp. affine ribulose-1,5-bisphosphate carboxylase/oxygenase large subunit (rbcL) gene, partial cds; chloroplast gene for chloroplast product	1057	1057	96%	0.0	96.28%	AY226128.1
Equisetum scirpoides isolate 10933 ribulose-1,5-bisphosphate carboxylase/oxygenase large subunit (rbcL) gene, partial cds; plastid	1055	1055	91%	0.0	97.56%	MH750201.1
Equisetum giganteum isolate 40806 ribulose-1,5-bisphosphate carboxylase/oxygenase large subunit (rbcL) gene, partial cds; plastid	1055	1055	95%	0.0	96.41%	MH750172.1
Equisetum giganteum isolate 26087 ribulose-1,5-bisphosphate carboxylase/oxygenase large subunit (rbcL) gene, partial cds; plastid	1055	1055	95%	0.0	96.41%	MH750170.1
Equisetum ramosissimum voucher R825 ribulose-1,5-bisphosphate carboxylase/oxygenase large subunit (rbcl) gene, partial cds; chloroplast	1055	1055	96%	0.0	96.00%	KY656709.1
Equisetum x ferrissii ribulose-1,5-bisphosphate carboxylase/oxygenase large subunit (rbcL) gene, partial cds; chloroplast	1055	1055	95%	0.0	96.41%	EU352292.1
Equisetum x ferrissii ribulose-1,5-bisphosphate carboxylase large subunit (rbcL) gene, partial cds; chloroplast gene for chloroplast product	1053	1053	95%	0.0	96.41%	AF313579.1
Equisetum ramosissimum subsp. debile voucher G.M. Plunkett 3642 (NYBG) ribulose-1,5-bisphosphate carboxylase/oxygenase large subunit (rbcL) gene, partial cds; chloroplast	1046	1046	96%	0.0	95.97%	MT657901.1

Description	Max Score	Total Score	Query Cover	E value	Per. Ident	Accession
Equisetum ramosissimum subsp. debile ribulose-1,5-bisphosphate carboxylase/oxygenase large subunit (rbcL) gene, partial cds; chloroplast gene for chloroplast product	1046	1046	96%	0.0	95.97%	AY226132.1
Equisetum myriochaetum isolate 40815 ribulose-1,5-bisphosphate carboxylase/oxygenase large subunit (rbcL) gene, partial cds; plastid	1044	1044	95%	0.0	96.24%	MH750185.1
Equisetum scirpoides voucher AP248 ribulose-1,5-bisphosphate carboxylase/oxygenase large subunit (rbcL) gene, partial cds; chloroplast	1038	1038	90%	0.0	97.53%	HQ590084.1
Equisetum xylochaetum isolate 40614 ribulose-1,5-bisphosphate carboxylase/oxygenase large subunit (rbcL) gene, partial cds; plastid	1035	1035	93%	0.0	96.35%	MH750228.1
Equisetum myriochaetum isolate 41080 ribulose-1,5-bisphosphate carboxylase/oxygenase large subunit (rbcL) gene, partial cds; plastid	1035	1035	93%	0.0	96.35%	MH750190.1
Equisetum myriochaetum isolate 40826 ribulose-1,5-bisphosphate carboxylase/oxygenase large subunit (rbcL) gene, partial cds; plastid	1035	1035	93%	0.0	96.35%	MH750188.1
Equisetum sylvaticum voucher TJD-502 ribulose-1,5-bisphosphate carboxylase/oxygenase large subunit (rbcL) gene, partial cds; chloroplast	1035	1035	84%	0.0	99.47%	KJ841305.1
Equisetum x schaffneri isolate 40813 ribulose-1,5-bisphosphate carboxylase/oxygenase large subunit (rbcL) gene, partial cds; plastid	1033	1033	93%	0.0	96.34%	MH750224.1
Equisetum giganteum isolate 40811 ribulose-1,5-bisphosphate carboxylase/oxygenase large subunit (rbcL) gene, partial cds; plastid	1031	1031	93%	0.0	96.34%	MH750176.1
Equisetum hyemale subsp. hyemale isolate 41088 ribulose-1,5-bisphosphate carboxylase/oxygenase large subunit (rbcL) gene, partial cds; plastid	1029	1029	93%	0.0	96.19%	MH750181.1
Equisetum variegatum ribulose-1,5-bisphosphate carboxylase/oxygenase large subunit (rbcL) gene, partial cds; chloroplast gene for chloroplast product	1029	1029	96%	0.0	95.50%	AY226134.1
Equisetum bogotense isolate 40800 ribulose-1,5-bisphosphate carboxylase/oxygenase large subunit (rbcL) gene, partial cds; plastid	1026	1026	92%	0.0	96.46%	MH750162.1
Equisetum arvense voucher TJD-508 ribulose-1,5-bisphosphate carboxylase/oxygenase large subunit (rbcL) gene, partial cds; chloroplast	1026	1026	85%	0.0	99.12%	KJ841301.1
Equisetum arvense voucher TJD-503 ribulose-1,5-bisphosphate carboxylase/oxygenase large subunit (rbcL) gene, partial cds; chloroplast	1024	1024	84%	0.0	99.12%	KJ841302.1
Equisetum myriochaetum isolate 40936 ribulose-1,5-bisphosphate carboxylase/oxygenase large subunit (rbcL) gene, partial cds; plastid	1022	1022	91%	0.0	96.59%	MH750189.1

Description	Max Score	Total Score	Query Cover	E value	Per. Ident	Accession
Equisetum myriochaetum isolate 26088 ribulose-1,5-bisphosphate carboxylase/oxygenase large subunit (rbcL) gene, partial cds; plastid	1022	1022	91%	0.0	96.59%	MH750184.1
Equisetum giganteum isolate 40807 ribulose-1,5-bisphosphate carboxylase/oxygenase large subunit (rbcL) gene, partial cds; plastid	1022	1022	92%	0.0	96.31%	MH750173.1
Equisetum arvense voucher TJD-504 ribulose-1,5-bisphosphate carboxylase/oxygenase large subunit (rbcL) gene, partial cds; chloroplast	1022	1022	84%	0.0	99.12%	KJ841300.1
Equisetum scirpoides chloroplast rbcL gene for ribulose-1,5-bisphosphate carboxylase/oxygenase large subunit, partial cds, specimen_voucher: TNS:768168	1022	1022	89%	0.0	97.49%	AB574689.1
Equisetum laevigatum isolate 40812 ribulose-1,5-bisphosphate carboxylase/oxygenase large subunit (rbcL) gene, partial cds; plastid	1020	1020	91%	0.0	96.59%	MH750183.1
Equisetum giganteum isolate 40809 ribulose-1,5-bisphosphate carboxylase/oxygenase large subunit (rbcL) gene, partial cds; plastid	1020	1020	91%	0.0	96.59%	MH750175.1
Equisetum bogotense isolate 40801 ribulose-1,5-bisphosphate carboxylase/oxygenase large subunit (rbcL) gene, partial cds; plastid	1020	1020	91%	0.0	96.73%	MH750163.1
Equisetum trachyodon isolate 41092 ribulose-1,5-bisphosphate carboxylase/oxygenase large subunit (rbcL) gene, partial cds; plastid	1018	1018	93%	0.0	96.01%	MH750227.1
Equisetum ramosissimum subsp. debile isolate 40829 ribulose-1,5-bisphosphate carboxylase/oxygenase large subunit (rbcL) gene, partial cds; plastid	1018	1018	93%	0.0	95.87%	MH750200.1
Equisetum hyemale voucher AP344 (OAC) ribulose-1,5-bisphosphate carboxylase/oxygenase large subunit (rbcL) gene, partial cds; chloroplast	1018	1018	92%	0.0	96.44%	EU677107.1
Equisetum hyemale subsp. hyemale isolate 20201 ribulose-1,5-bisphosphate carboxylase/oxygenase large subunit (rbcL) gene, partial cds; plastid	1016	1016	91%	0.0	96.43%	MH750179.1
Equisetum hyemale subsp. affine isolate 40831 ribulose-1,5-bisphosphate carboxylase/oxygenase large subunit (rbcL) gene, partial cds; plastid	1016	1016	92%	0.0	96.15%	MH750177.1
Equisetum sylvaticum plastid partial rbcL gene for ribulose-1,5-biphosphate carboxylase/oxygenase large subunit, specimen voucher H823764	1016	1016	83%	0.0	99.46%	HE574610.1

Alignments

Equisetum palustre chloroplast, partial genome

Sequence ID: **MT984458.1** Length: 5501 Number of Matches: 1
Range 1: 2215 to 2882

Score	Expect	Identities	Gaps	Strand	Frame
1201 bits(650)	0.0()	663/669(99%)	1/669(0%)	Plus/Plus	

Features:

```
Query  1     GTCACCACAAACAGAGACTAAAGCAGGTGTTGGATTTAAAGCTGGTGTTAACCGATTATC  60
             |||||||||||| ||||| ||||||||||||||||||||||||||||||||  |||||||
Sbjct  2215  GTCACCACAAACGGAGACCAAAGCAGGTGTTGGATTTAAAGCTGGTGTTAA-AGATTATC  2273

Query  61    GATTGACTTATTTTACTCCAGATTATGAAACCAAAGATACCGATATTTTAGCAGCATTCC  120
             ||||||||||||||||||||||||||||||||||||||||||||||||||||||||||||
Sbjct  2274  GATTGACTTATTTTACTCCAGATTATGAAACCAAAGATACCGATATTTTAGCAGCATTCC  2333

Query  121   GTATGACTCCTCAACCGGGAGTACCACCGGAAGAAGCAGGAGCAGCCGTAGCTGCTGAAT  180
             ||||||||||||||||||||||||||||||||||||||||||||||||||||||||||||
Sbjct  2334  GTATGACTCCTCAACCGGGAGTACCACCGGAAGAAGCAGGAGCAGCCGTAGCTGCTGAAT  2393

Query  181   CGTCCACGGGCACCTGGACTACCGTATGGACAGATGGACTTACTAGTCTTGATCGATATA  240
             ||||||||||||||||||||||||||||||||||||||||||||||||||||||||||||
Sbjct  2394  CGTCCACGGGCACCTGGACTACCGTATGGACAGATGGACTTACTAGTCTTGATCGATATA  2453

Query  241   AAGGTCGATGCTATAATATTGAACCTGTTGCTGGAGAAGATAATCAATTCATAGCTTATG  300
             ||||||||||||||||||||||||||||||||||||||||||||||||||||||||||||
Sbjct  2454  AAGGTCGATGCTATAATATTGAACCTGTTGCTGGAGAAGATAATCAATTCATAGCTTATG  2513

Query  301   TAGCCTATCCTTTAGATCTTTTTGAAGAAGGTTCTGTTACCAATCTTTTTACTTCAATTG  360
             ||||||||||||||||||||||||||||||||||||||||||||||||||||||||||||
Sbjct  2514  TAGCCTATCCTTTAGATCTTTTTGAAGAAGGTTCTGTTACCAATCTTTTTACTTCAATTG  2573

Query  361   TTGGTAATGTTTTCGGCTTCAAAGCTCTACGTGCTTTACGTTTAGAAGATTTAAGAATTC  420
             ||||||||||||||||||||||||||||||||||||||||||||||||||||||||||||
Sbjct  2574  TTGGTAATGTTTTCGGCTTCAAAGCTCTACGTGCTTTACGTTTAGAAGATTTAAGAATTC  2633

Query  421   CTCCTGCTTACTCCAAAACTTTTATAGGACCACCCCACGGTATCCAGGTTGAAAGAGATA  480
             ||||||||||||||||||||||||||||||||||||||||||||||||||||||||||||
Sbjct  2634  CTCCTGCTTACTCCAAAACTTTTATAGGACCACCCCACGGTATCCAGGTTGAAAGAGATA  2693

Query  481   AGTTAAACAAATATGGTCGTCCTTTATTAGGTTGTACAATTAAACCAAAATTGGGACTAT  540
             ||||||||||||||||||||||||||||||||||||||||||||||||||||||||||||
Sbjct  2694  AGTTAAACAAATATGGTCGTCCTTTATTAGGTTGTACAATTAAACCAAAATTGGGACTAT  2753

Query  541   CTGCTAAAAACTATGGTAGGGCTGTTTATGAATGTCTTCGTGGTGGACTTGATTTCACCA  600
             ||||||||||||||||||||||||||||||||||||||||||||||||||||||||||||
Sbjct  2754  CTGCTAAAAACTATGGTAGGGCTGTTTATGAATGTCTTCGTGGTGGACTTGATTTCACCA  2813

Query  601   AAGATGATGAGAATGTAAACTCTCAACCCTTTATGCGTTGGAGAGATCGATTCTTATTTG  660
             ||||||||||||||||||||||||||||||||||||||||||||||||| |||||||| |
Sbjct  2814  AAGATGATGAGAATGTAAACTCTCAACCCTTTATGCGTTGGAGAGATCGTTTCTTATTCG  2873

Query  661   TAGCAGAAG  669
             |||||||||
Sbjct  2874  TAGCAGAAG  2882
```

Equisetum arvense chloroplast, complete genome

Sequence ID: **GU191334.1** Length: 133309 Number of Matches: 1

Range 1: 27302 to 27969

Score	Expect	Identities	Gaps	Strand	Frame
1179 bits(638)	0.0()	659/669(99%)	1/669(0%)	Plus/Minus	

Features:

```
Query  1      GTCACCACAAACAGAGACTAAAGCAGGTGTTGGATTTAAAGCTGGTGTTAACCGATTATC  60
              |||||||||||| ||||| ||||||||||||||||||||||||||||||||  |||||||
Sbjct  27969  GTCACCACAAACGGAGACCAAAGCAGGTGTTGGATTTAAAGCTGGTGTTAA-AGATTATC  27911

Query  61     GATTGACTTATTTTACTCCAGATTATGAAACCAAAGATACCGATATTTTAGCAGCATTCC  120
              ||||||||||||||||||||||||||||||||||||||||||||||||||||||||||||
Sbjct  27910  GATTGACTTATTTTACTCCAGATTATGAAACCAAAGATACCGATATTTTAGCAGCATTCC  27851

Query  121    GTATGACTCCTCAACCGGGAGTACCACCGGAAGAAGCAGGAGCAGCCGTAGCTGCTGAAT  180
              ||||||||||||||||||||||||||||||||||||||||||||||||||||||||||||
Sbjct  27850  GTATGACTCCTCAACCGGGAGTACCACCGGAAGAAGCAGGAGCAGCCGTAGCTGCTGAAT  27791

Query  181    CGTCCACGGGCACCTGGACTACCGTATGGACAGATGGACTTACTAGTCTTGATCGATATA  240
              ||||||||||||||||||||||||||||||||||||||||||||||||||||||||||||
Sbjct  27790  CGTCCACGGGCACCTGGACTACCGTATGGACAGATGGACTTACTAGTCTTGATCGATATA  27731

Query  241    AAGGTCGATGCTATAATATTGAACCTGTTGCTGGAGAAGATAATCAATTCATAGCTTATG  300
              ||||||||||||||||||||||||||||||||||||||||||||||||||||||||||||
Sbjct  27730  AAGGTCGATGCTATAATATTGAACCTGTTGCTGGAGAAGATAATCAATTCATAGCTTATG  27671

Query  301    TAGCCTATCCTTTAGATCTTTTTGAAGAAGGTTCTGTTACCAATCTTTTTACTTCAATTG  360
              ||||||| ||||||||||||||||||||||||||||||||||||||||||||||||||||
```

```
Sbjct  27670  TAGCCTACCCTTTAGATCTTTTTGAAGAAGGTTCTGTTACCAATCTTTTTACTTCAATTG  27611

Query  361    TTGGTAATGTTTTCGGCTTCAAAGCTCTACGTGCTTTACGTTTAGAAGATTTAAGAATTC  420
              ||||||||||||||||||||||||||||||||||||||||||||||||||||||||||||
Sbjct  27610  TTGGTAATGTTTTCGGCTTCAAAGCTCTACGTGCTTTACGTTTAGAAGATTTAAGAATTC  27551

Query  421    CTCCTGCTTACTCCAAAACTTTTATAGGACCACCCCACGGTATCCAGGTTGAAAGAGATA  480
              ||||||||||||||||||||||||||||||||||||||||||||||||||||||||||||
Sbjct  27550  CTCCTGCTTACTCCAAAACTTTTATAGGACCACCCCACGGTATCCAGGTTGAAAGAGATA  27491

Query  481    AGTTAAACAAATATGGTCGTCCTTTATTAGGTTGTACAATTAAACCAAAATTGGGACTAT  540
              ||||||||||||| ||||||||||||||||||||||||||||||||||||||||||||||
Sbjct  27490  AGTTAAACAAATACGGTCGTCCTTTATTAGGTTGTACAATTAAACCAAAATTGGGACTAT  27431

Query  541    CTGCTAAAAACTATGGTAGGGCTGTTTATGAATGTCTTCGTGGTGGACTTGATTTCACCA  600
              ||||||||||||||||||| |||||||||||||||||||||||||| |||||||||||||
Sbjct  27430  CTGCTAAAAACTATGGTAGAGCTGTTTATGAATGTCTTCGTGGTGGGCTTGATTTCACCA  27371

Query  601    AAGATGATGAGAATGTAAACTCTCAACCCTTTATGCGTTGGAGAGATCGATTCTTATTTG  660
              ||||||||||||||||||||||||||||||||||||||||||||||||| |||||||| |
Sbjct  27370  AAGATGATGAGAATGTAAACTCTCAACCCTTTATGCGTTGGAGAGATCGTTTCTTATTCG  27311

Query  661    TAGCAGAAG  669
              |||||||||
Sbjct  27310  TAGCAGAAG  27302
```

Equisetum arvense chloroplast ribulosebisphosphate carboxylase (rbcL) gene, complete cds

Sequence ID: **L11053.1** Length: 1587 Number of Matches: 1

Range 1: 75 to 742

Score	Expect	Identities	Gaps	Strand	Frame
1179 bits(638)	0.0()	659/669(99%)	1/669(0%)	Plus/Plus	

Features:

```
Query  1    GTCACCACAAACAGAGACTAAAGCAGGTGTTGGATTTAAAGCTGGTGTTAACCGATTATC  60
            |||||||||||| ||||| ||||||||||||||||||||||||||||||||  |||||||
Sbjct  75   GTCACCACAAACGGAGACCAAAGCAGGTGTTGGATTTAAAGCTGGTGTTAA-AGATTATC  133

Query  61   GATTGACTTATTTTACTCCAGATTATGAAACCAAAGATACCGATATTTTAGCAGCATTCC  120
            ||||||||||||||||||||||||||||||||||||||||||||||||||||||||||||
Sbjct  134  GATTGACTTATTTTACTCCAGATTATGAAACCAAAGATACCGATATTTTAGCAGCATTCC  193

Query  121  GTATGACTCCTCAACCGGGAGTACCACCGGAAGAAGCAGGAGCAGCCGTAGCTGCTGAAT  180
            ||||||||||||||||||||||||||||||||||||||||||||||||||||||||||||
Sbjct  194  GTATGACTCCTCAACCGGGAGTACCACCGGAAGAAGCAGGAGCAGCCGTAGCTGCTGAAT  253

Query  181  CGTCCACGGGCACCTGGACTACCGTATGGACAGATGGACTTACTAGTCTTGATCGATATA  240
            ||||||||||||||||||||||||||||||||||||||||||||||||||||||||||||
Sbjct  254  CGTCCACGGGCACCTGGACTACCGTATGGACAGATGGACTTACTAGTCTTGATCGATATA  313

Query  241  AAGGTCGATGCTATAATATTGAACCTGTTGCTGGAGAAGATAATCAATTCATAGCTTATG  300
            ||||||||||||||||||||||||||||||||||||||||||||||||||||||||||||
Sbjct  314  AAGGTCGATGCTATAATATTGAACCTGTTGCTGGAGAAGATAATCAATTCATAGCTTATG  373

Query  301  TAGCCTATCCTTTAGATCTTTTTGAAGAAGGTTCTGTTACCAATCTTTTTACTTCAATTG  360
            ||||||| ||||||||||||||||||||||||||||||||||||||||||||||||||||
Sbjct  374  TAGCCTACCCTTTAGATCTTTTTGAAGAAGGTTCTGTTACCAATCTTTTTACTTCAATTG  433

Query  361  TTGGTAATGTTTTCGGCTTCAAAGCTCTACGTGCTTTACGTTTAGAAGATTTAAGAATTC  420
            ||||||||||||||||||||||||||||||||||||||||||||||||||||||||||||
Sbjct  434  TTGGTAATGTTTTCGGCTTCAAAGCTCTACGTGCTTTACGTTTAGAAGATTTAAGAATTC  493

Query  421  CTCCTGCTTACTCCAAAACTTTTATAGGACCACCCCACGGTATCCAGGTTGAAAGAGATA  480
            ||||||||||||||||||||||||||||||||||||||||||||||||||||||||||||
Sbjct  494  CTCCTGCTTACTCCAAAACTTTTATAGGACCACCCCACGGTATCCAGGTTGAAAGAGATA  553

Query  481  AGTTAAACAAATATGGTCGTCCTTTATTAGGTTGTACAATTAAACCAAAATTGGGACTAT  540
            ||||||||||||| ||||||||||||||||||||||||||||||||||||||||||||||
Sbjct  554  AGTTAAACAAATACGGTCGTCCTTTATTAGGTTGTACAATTAAACCAAAATTGGGACTAT  613

Query  541  CTGCTAAAAACTATGGTAGGGCTGTTTATGAATGTCTTCGTGGTGGACTTGATTTCACCA  600
            ||||||||||||||||||| |||||||||||||||||||||||||| |||||||||||||
Sbjct  614  CTGCTAAAAACTATGGTAGAGCTGTTTATGAATGTCTTCGTGGTGGGCTTGATTTCACCA  673

Query  601  AAGATGATGAGAATGTAAACTCTCAACCCTTTATGCGTTGGAGAGATCGATTCTTATTTG  660
            ||||||||||||||||||||||||||||||||||||||||||||||||| |||||||| |
Sbjct  674  AAGATGATGAGAATGTAAACTCTCAACCCTTTATGCGTTGGAGAGATCGTTTCTTATTCG  733

Query  661  TAGCAGAAG  669
            |||||||||
Sbjct  734  TAGCAGAAG  742
```

Equisetum arvense plastid, complete genome

Sequence ID: **JN968380.1** Length: 132726 Number of Matches: 1

Range 1: 64960 to 65627

Score	Expect	Identities	Gaps	Strand	Frame
1173 bits(635)	0.0()	658/669(98%)	1/669(0%)	Plus/Plus	

Features:

```
Query  1      GTCACCACAAACAGAGACTAAAGCAGGTGTTGGATTTAAAGCTGGTGTTAACCGATTATC  60
              |||||||||||| ||||| ||||||||||||||||||||||||||||||||  |||||||
Sbjct  64960  GTCACCACAAACGGAGACCAAAGCAGGTGTTGGATTTAAAGCTGGTGTTAA-AGATTATC  65018

Query  61     GATTGACTTATTTTACTCCAGATTATGAAACCAAAGATACCGATATTTTAGCAGCATTCC  120
              ||||||||||||||||||||||||||||||||||||||||||||||||||||||||||||
Sbjct  65019  GATTGACTTATTTTACTCCAGATTATGAAACCAAAGATACCGATATTTTAGCAGCATTCC  65078

Query  121    GTATGACTCCTCAACCGGGAGTACCACCGGAAGAAGCAGGAGCAGCCGTAGCTGCTGAAT  180
              ||||||||||||||||||||||||||||||||||||||||||||||||||||||||||||
Sbjct  65079  GTATGACTCCTCAACCGGGAGTACCACCGGAAGAAGCAGGAGCAGCCGTAGCTGCTGAAT  65138

Query  181    CGTCCACGGGCACCTGGACTACCGTATGGACAGATGGACTTACTAGTCTTGATCGATATA  240
              ||||||||||||||||||||||||||||||||||||||||||||||||||||||||||||
Sbjct  65139  CGTCCACGGGCACCTGGACTACCGTATGGACAGATGGACTTACTAGTCTTGATCGATATA  65198

Query  241    AAGGTCGATGCTATAATATTGAACCTGTTGCTGGAGAAGATAATCAATTCATAGCTTATG  300
              ||||||||||||||||||||||||||||||||||||||||||||||||||||||||||||
Sbjct  65199  AAGGTCGATGCTATAATATTGAACCTGTTGCTGGAGAAGATAATCAATTCATAGCTTATG  65258

Query  301    TAGCCTATCCTTTAGATCTTTTTGAAGAAGGTTCTGTTACCAATCTTTTTACTTCAATTG  360
              ||||||| ||||||||||||||||||||||||||||||||||||||||||||||||||||
Sbjct  65259  TAGCCTACCCTTTAGATCTTTTTGAAGAAGGTTCTGTTACCAATCTTTTTACTTCAATTG  65318

Query  361    TTGGTAATGTTTTCGGCTTCAAAGCTCTACGTGCTTTACGTTTAGAAGATTTAAGAATTC  420
              |||||||||||||||| |||||||||||||||||||||||||||||||||||||||||||
Sbjct  65319  TTGGTAATGTTTTCGGATTCAAAGCTCTACGTGCTTTACGTTTAGAAGATTTAAGAATTC  65378

Query  421    CTCCTGCTTACTCCAAAACTTTTATAGGACCACCCCACGGTATCCAGGTTGAAAGAGATA  480
              ||||||||||||||||||||||||||||||||||||||||||||||||||||||||||||
Sbjct  65379  CTCCTGCTTACTCCAAAACTTTTATAGGACCACCCCACGGTATCCAGGTTGAAAGAGATA  65438

Query  481    AGTTAAACAAATATGGTCGTCCTTTATTAGGTTGTACAATTAAACCAAAATTGGGACTAT  540
              ||||||||||||| ||||||||||||||||||||||||||||||||||||||||||||||
Sbjct  65439  AGTTAAACAAATACGGTCGTCCTTTATTAGGTTGTACAATTAAACCAAAATTGGGACTAT  65498

Query  541    CTGCTAAAAACTATGGTAGGGCTGTTTATGAATGTCTTCGTGGTGGACTTGATTTCACCA  600
              ||||||||||||||||||| |||||||||||||||||||||||||| |||||||||||||
Sbjct  65499  CTGCTAAAAACTATGGTAGAGCTGTTTATGAATGTCTTCGTGGTGGGCTTGATTTCACCA  65558

Query  601    AAGATGATGAGAATGTAAACTCTCAACCCTTTATGCGTTGGAGAGATCGATTCTTATTTG  660
              ||||||||||||||||||||||||||||||||||||||||||||||||| |||||||| |
Sbjct  65559  AAGATGATGAGAATGTAAACTCTCAACCCTTTATGCGTTGGAGAGATCGTTTCTTATTCG  65618

Query  661    TAGCAGAAG  669
              |||||||||
Sbjct  65619  TAGCAGAAG  65627
```

Equisetum palustre ribulose-1,5-bisphosphate carboxylase/oxygenase large subunit (rbcL) gene, partial cds; chloroplast gene for chloroplast product

Sequence ID: **AY226138.1** Length: 1325 Number of Matches: 1

Range 1: 1 to 644

Score	Expect	Identities	Gaps	Strand	Frame
1168 bits(632)	0.0()	641/645(99%)	1/645(0%)	Plus/Plus	

Features:

```
Query  25   AGGTGTTGGATTTAAAGCTGGTGTTAACCGATTATCGATTGACTTATTTTACTCCAGATT  84
            ||||||||||||||||||||||||||||  ||||||||||||||||||||||||||||||
Sbjct  1    AGGTGTTGGATTTAAAGCTGGTGTTAA-AGATTATCGATTGACTTATTTTACTCCAGATT  59

Query  85   ATGAAACCAAAGATACCGATATTTTAGCAGCATTCCGTATGACTCCTCAACCGGGAGTAC  144
            ||||||||||||||||||||||||||||||||||||||||||||||||||||||||||||
Sbjct  60   ATGAAACCAAAGATACCGATATTTTAGCAGCATTCCGTATGACTCCTCAACCGGGAGTAC  119

Query  145  CACCGGAAGAAGCAGGAGCAGCCGTAGCTGCTGAATCGTCCACGGGCACCTGGACTACCG  204
            ||||||||||||||||||||||||||||||||||||||||||||||||||||||||||||
Sbjct  120  CACCGGAAGAAGCAGGAGCAGCCGTAGCTGCTGAATCGTCCACGGGCACCTGGACTACCG  179

Query  205  TATGGACAGATGGACTTACTAGTCTTGATCGATATAAAGGTCGATGCTATAATATTGAAC  264
            ||||||||||||||||||||||||||||||||||||||||||||||||||||||||||||
Sbjct  180  TATGGACAGATGGACTTACTAGTCTTGATCGATATAAAGGTCGATGCTATAATATTGAAC  239
```

```
Query  265   CTGTTGCTGGAGAAGATAATCAATTCATAGCTTATGTAGCCTATCCTTTAGATCTTTTTG  324
             ||||||||||||||||||||||||||||||||||||||||||||||||||||||||||||
Sbjct  240   CTGTTGCTGGAGAAGATAATCAATTCATAGCTTATGTAGCCTATCCTTTAGATCTTTTTG  299

Query  325   AAGAAGGTTCTGTTACCAATCTTTTTACTTCAATTGTTGGTAATGTTTTCGGCTTCAAAG  384
             ||||||||||||||||||||||||||||||||||||||||||||||||||||||||||||
Sbjct  300   AAGAAGGTTCTGTTACCAATCTTTTTACTTCAATTGTTGGTAATGTTTTCGGCTTCAAAG  359

Query  385   CTCTACGTGCTTTACGTTTAGAAGATTTAAGAATTCCTCCTGCTTACTCCAAAACTTTTA  444
             ||||||||||||||||||||||||||||||||||||||||||||||||||||||||||||
Sbjct  360   CTCTACGTGCTTTACGTTTAGAAGATTTAAGAATTCCTCCTGCTTACTCCAAAACTTTTA  419

Query  445   TAGGACCACCCCACGGTATCCAGGTTGAAAGAGATAAGTTAAACAAATATGGTCGTCCTT  504
             ||||||||||||||||||||||||||||||||||||||||||||||||||||||||||||
Sbjct  420   TAGGACCACCCCACGGTATCCAGGTTGAAAGAGATAAGTTAAACAAATATGGTCGTCCTT  479

Query  505   TATTAGGTTGTACAATTAAACCAAAATTGGGACTATCTGCTAAAAACTATGGTAGGGCTG  564
             ||||||||||||||||||||||||||||||||||||||||||||||||||||||||||||
Sbjct  480   TATTAGGTTGTACAATTAAACCAAAATTGGGACTATCTGCTAAAAACTATGGTAGGGCTG  539

Query  565   TTTATGAATGTCTTCGTGGTGGACTTGATTTCACCAAAGATGATGAGAATGTAAACTCTC  624
             ||||||||||||||||||||||||||||||||||||||||||||||||||||||||||||
Sbjct  540   TTTATGAATGTCTTCGTGGTGGACTTGATTTCACCAAAGATGATGAGAATGTAAACTCTC  599

Query  625   AACCCTTTATGCGTTGGAGAGATCGATTCTTATTTGTAGCAGAAG  669
             ||||||||||||||||||||||||| |||||||| ||||||||||
Sbjct  600   AACCCTTTATGCGTTGGAGAGATCGTTTCTTATTCGTAGCAGAAG  644
```


BLAST is a registered trademark of the National Library of Medicine

Support center Mailing list YouTube

- National Library Of Medicine
- National Institutes Of Health
- U.S. Department of Health & Human Services
- USA.gov

NCBI

National Center for Biotechnology Information, U.S. National Library of Medicine 8600 Rockville Pike, Bethesda MD, 20894 USA

Policies and Guidelines | Contact

HHS Vulnerability Disclosure
