# Supplementary material for: A Multi-Pronged Technique for Identifying Equisetum palustre and Equisetum arvense—Combining HPTLC, HPLC-ESI-MS/MS and Optimized DNA Barcoding Techniques
Source: Plants (Basel). 2022 Sep 28;11(19):2562. doi: 10.3390/plants11192562 (PMC9573248; doi:10.3390/plants11192562)
Supplement: Supplementary file 1 [file plants-11-02562-s001.zip › plants-1932149 supplementary/Horsetail Supplementary Figures/Figure S23 (P5, 82711220323-1).pdf]

**BLAST Results**

Questions/comments

### Job title: Nucleotide Sequence

| | |
|---|---|
| **RID** | J1C4E0FZ013 (Expires on 09-15 00:32 am) |
| **Query ID** | lcl\|Query_49661 |
| **Description** | None |
| **Molecule type** | dna |
| **Query Length** | 656 |
| **Database Name** | nt |
| **Description** | Nucleotide collection (nt) |
| **Program** | BLASTN 2.13.0+ |

## Graphic Summary

Distribution of the top 100 Blast Hits on 100 subject sequences

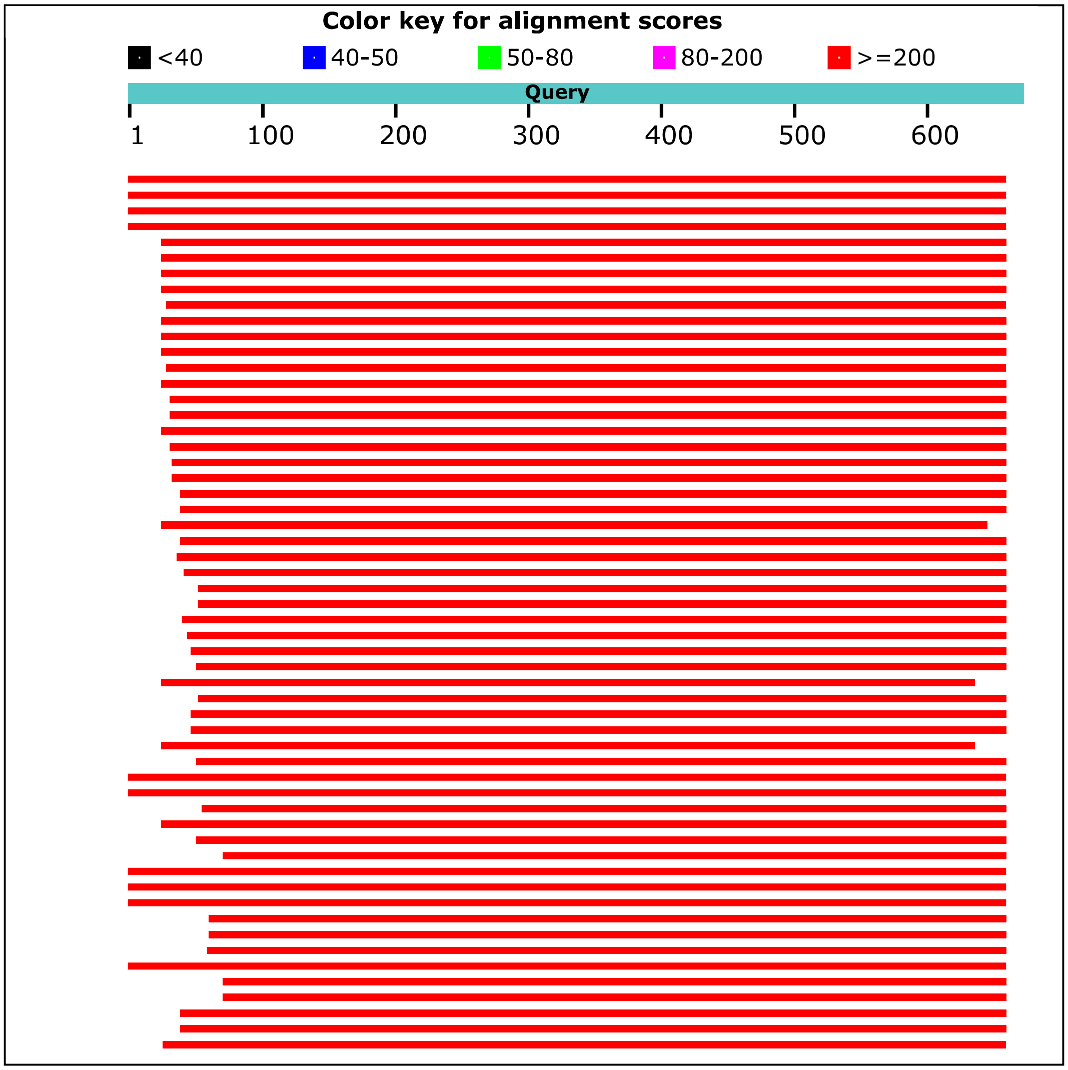

## Descriptions

Sequences producing significant alignments:

| Description | Max Score | Total Score | Query Cover | E value | Per. Ident | Accession |
|---|---|---|---|---|---|---|
| Equisetum palustre chloroplast, partial genome | 1195 | 1195 | 100% | 0.0 | 99.54% | MT984458.1 |
| Equisetum arvense chloroplast, complete genome | 1173 | 1173 | 100% | 0.0 | 98.93% | GU191334.1 |
| Equisetum arvense chloroplast ribulosebisphosphate carboxylase (rbcL) gene, complete cds | 1173 | 1173 | 100% | 0.0 | 98.93% | L11053.1 |
| Equisetum arvense plastid, complete genome | 1168 | 1168 | 100% | 0.0 | 98.78% | JN968380.1 |
| Equisetum palustre ribulose-1,5-bisphosphate carboxylase/oxygenase large subunit (rbcL) gene, partial cds; chloroplast gene for chloroplast product | 1160 | 1160 | 96% | 0.0 | 99.84% | AY226138.1 |
| Equisetum pratense ribulose-1,5-bisphosphate carboxylase/oxygenase large subunit (rbcL) gene, partial cds; chloroplast gene for chloroplast product | 1149 | 1149 | 96% | 0.0 | 99.52% | AY226137.1 |
| Equisetum sylvaticum ribulose-1,5-bisphosphate carboxylase/oxygenase large subunit (rbcL) gene, partial cds; chloroplast gene for chloroplast product | 1149 | 1149 | 96% | 0.0 | 99.52% | AY226136.1 |
| Equisetum telmateia subsp. braunii ribulose-1,5-bisphosphate carboxylase/oxygenase large subunit (rbcL) gene, partial cds; chloroplast gene for chloroplast product | 1144 | 1144 | 96% | 0.0 | 99.37% | AY226135.1 |
| Equisetum sylvaticum isolate 11640 ribulose-1,5-bisphosphate carboxylase/oxygenase large subunit (rbcL) gene, partial cds; plastid | 1142 | 1142 | 95% | 0.0 | 99.52% | MH750204.1 |
| Equisetum environmental sample isolate EDNA16-0043490 ribulose-1,5-bisphosphate carboxylase/oxygenase large subunit (rbcL) gene, partial cds; chloroplast | 1138 | 1138 | 96% | 0.0 | 99.21% | MT197555.1 |
| Equisetum fluviatile ribulose-1,5-bisphosphate carboxylase/oxygenase large subunit (rbcL) gene, partial cds; chloroplast gene for chloroplast product | 1138 | 1138 | 96% | 0.0 | 99.21% | AY226142.1 |
| Equisetum arvense ribulose-1,5-bisphosphate carboxylase/oxygenase large subunit (rbcL) gene, partial cds; chloroplast gene for chloroplast product | 1138 | 1138 | 96% | 0.0 | 99.21% | AY226140.1 |
| Equisetum telmateia subsp. telmateia isolate 11642 ribulose-1,5-bisphosphate carboxylase/oxygenase large subunit (rbcL) gene, partial cds; plastid | 1136 | 1136 | 95% | 0.0 | 99.36% | MH750210.1 |
| Equisetum environmental sample isolate EDNA16-0043491b ribulose-1,5-bisphosphate carboxylase/oxygenase large subunit (rbcL) gene, partial cds; chloroplast | 1133 | 1133 | 96% | 0.0 | 99.05% | MT197548.1 |

| Description | Max Score | Total Score | Query Cover | E value | Per. Ident | Accession |
|---|---|---|---|---|---|---|
| Equisetum telmateia ribulose-1,5-bisphosphate carboxylase large subunit (rbcL) gene, partial cds; chloroplast gene for chloroplast product | 1131 | 1131 | 95% | 0.0 | 99.36% | AF313580.1 |
| Equisetum arvense subsp. boreale isolate 41074 ribulose-1,5-bisphosphate carboxylase/oxygenase large subunit (rbcL) gene, partial cds; plastid | 1127 | 1127 | 95% | 0.0 | 99.20% | MH750160.1 |
| Equisetum diffusum ribulose-1,5-bisphosphate carboxylase/oxygenase large subunit (rbcL) gene, partial cds; chloroplast gene for chloroplast product | 1127 | 1127 | 96% | 0.0 | 98.89% | AY226141.1 |
| Equisetum x dycei isolate 26083 ribulose-1,5-bisphosphate carboxylase/oxygenase large subunit (rbcL) gene, partial cds; plastid | 1125 | 1125 | 95% | 0.0 | 99.20% | MH750220.1 |
| Equisetum fluviatile isolate 41076 ribulose-1,5-bisphosphate carboxylase/oxygenase large subunit (rbcL) gene, partial cds; plastid | 1123 | 1123 | 94% | 0.0 | 99.20% | MH750169.1 |
| Equisetum fluviatile isolate 41075 ribulose-1,5-bisphosphate carboxylase/oxygenase large subunit (rbcL) gene, partial cds; plastid | 1123 | 1123 | 94% | 0.0 | 99.20% | MH750168.1 |
| Equisetum sylvaticum isolate 41081 ribulose-1,5-bisphosphate carboxylase/oxygenase large subunit (rbcL) gene, partial cds; plastid | 1122 | 1122 | 93% | 0.0 | 99.51% | MH750205.1 |
| Equisetum pratense isolate 41087 ribulose-1,5-bisphosphate carboxylase/oxygenase large subunit (rbcL) gene, partial cds; plastid | 1122 | 1122 | 93% | 0.0 | 99.51% | MH750195.1 |
| Equisetum arvense voucher JAG288 (OAC) ribulose-1,5-bisphosphate carboxylase/oxygenase large subunit (rbcL) gene, partial cds; chloroplast | 1118 | 1118 | 94% | 0.0 | 99.35% | EU677106.1 |
| Equisetum telmateia subsp. braunii isolate 40828 ribulose-1,5-bisphosphate carboxylase/oxygenase large subunit (rbcL) gene, partial cds; plastid | 1116 | 1116 | 93% | 0.0 | 99.35% | MH750207.1 |
| Equisetum arvense subsp. boreale isolate 41073 ribulose-1,5-bisphosphate carboxylase/oxygenase large subunit (rbcL) gene, partial cds; plastid | 1116 | 1116 | 94% | 0.0 | 99.19% | MH750159.1 |
| Equisetum x fontqueri isolate 26093 ribulose-1,5-bisphosphate carboxylase/oxygenase large subunit (rbcL) gene, partial cds; plastid | 1112 | 1112 | 93% | 0.0 | 99.35% | MH750221.1 |
| Equisetum palustre isolate 17671 ribulose-1,5-bisphosphate carboxylase/oxygenase large subunit (rbcL) gene, partial cds; plastid | 1110 | 1110 | 92% | 0.0 | 99.83% | MH750191.1 |
| Equisetum palustre isolate 39349 ribulose-1,5-bisphosphate carboxylase/oxygenase large subunit (rbcL) gene, partial cds; plastid | 1109 | 1109 | 91% | 0.0 | 99.83% | MH750192.1 |
| Equisetum arvense subsp. arvense isolate 41071 ribulose-1,5-bisphosphate carboxylase/oxygenase large subunit (rbcL) gene, partial cds; plastid | 1109 | 1109 | 93% | 0.0 | 99.19% | MH750158.1 |

| Description | Max Score | Total Score | Query Cover | E value | Per. Ident | Accession |
|---|---|---|---|---|---|---|
| Equisetum x litorale isolate 41084 ribulose-1,5-bisphosphate carboxylase/oxygenase large subunit (rbcL) gene, partial cds; plastid | 1103 | 1103 | 93% | 0.0 | 99.18% | MH750222.1 |
| Equisetum telmateia subsp. braunii isolate 40832 ribulose-1,5-bisphosphate carboxylase/oxygenase large subunit (rbcL) gene, partial cds; plastid | 1103 | 1103 | 92% | 0.0 | 99.34% | MH750208.1 |
| Equisetum pratense isolate 41086 ribulose-1,5-bisphosphate carboxylase/oxygenase large subunit (rbcL) gene, partial cds; plastid | 1101 | 1101 | 92% | 0.0 | 99.50% | MH750194.1 |
| Equisetum arvense voucher AP400 (OAC) ribulose-1,5-bisphosphate carboxylase/oxygenase large subunit (rbcL) gene, partial cds; chloroplast | 1099 | 1099 | 92% | 0.0 | 99.34% | EU677105.1 |
| Equisetum pratense isolate 39348 ribulose-1,5-bisphosphate carboxylase/oxygenase large subunit (rbcL) gene, partial cds; plastid | 1098 | 1098 | 91% | 0.0 | 99.50% | MH750193.1 |
| Equisetum arvense subsp. arvense isolate 26085 ribulose-1,5-bisphosphate carboxylase/oxygenase large subunit (rbcL) gene, partial cds; plastid | 1098 | 1098 | 92% | 0.0 | 99.18% | MH750156.1 |
| Equisetum diffusum isolate 40689 ribulose-1,5-bisphosphate carboxylase/oxygenase large subunit (rbcL) gene, partial cds; plastid | 1092 | 1092 | 92% | 0.0 | 99.01% | MH750166.1 |
| Equisetum pratense voucher AP451 ribulose-1,5-bisphosphate carboxylase/oxygenase large subunit (rbcL) gene, partial cds; chloroplast | 1092 | 1092 | 92% | 0.0 | 99.01% | HQ590083.1 |
| Equisetum arvense subsp. arvense isolate 26084 ribulose-1,5-bisphosphate carboxylase/oxygenase large subunit (rbcL) gene, partial cds; plastid | 1090 | 1090 | 92% | 0.0 | 99.17% | MH750155.1 |
| Equisetum xylochaetum chloroplast, complete genome | 1090 | 1090 | 100% | 0.0 | 96.65% | MW282958.1 |
| Equisetum hyemale chloroplast, complete genome | 1085 | 1085 | 100% | 0.0 | 96.49% | KC117177.1 |
| Equisetum arvense subsp. arvense isolate 40833 ribulose-1,5-bisphosphate carboxylase/oxygenase large subunit (rbcL) gene, partial cds; plastid | 1083 | 1083 | 91% | 0.0 | 99.17% | MH750157.1 |
| Equisetum scirpoides ribulose-1,5-bisphosphate carboxylase/oxygenase large subunit (rbcL) gene, partial cds; chloroplast gene for chloroplast product | 1083 | 1083 | 96% | 0.0 | 97.62% | AY226133.1 |
| Equisetum diffusum isolate 40804 ribulose-1,5-bisphosphate carboxylase/oxygenase large subunit (rbcL) gene, partial cds; plastid | 1079 | 1079 | 92% | 0.0 | 98.84% | MH750167.1 |
| Equisetum palustre chloroplast rbcL gene for ribulose-1,5-bisphosphate carboxylase/oxygenase large subunit, partial cds, specimen_voucher: TNS:776981 | 1075 | 1075 | 89% | 0.0 | 99.83% | AB574686.1 |
| Equisetum ramosissimum isolate BPTPS123 voucher LISE:96444 chloroplast, complete genome | 1074 | 1074 | 100% | 0.0 | 96.19% | ON641349.1 |

| Description | Max Score | Total Score | Query Cover | E value | Per. Ident | Accession |
|---|---|---|---|---|---|---|
| Equisetum ramosissimum isolate BPTPS056 voucher LISE:96334 chloroplast, complete genome | 1074 | 1074 | 100% | 0.0 | 96.19% | NC_062377.1 |
| Equisetum ramosissimum isolate BPTPS056 voucher LISE:96334 chloroplast, complete genome | 1074 | 1074 | 100% | 0.0 | 96.19% | OM691674.1 |
| Equisetum fluviatile ribulose-1,5-bisphosphate carboxylase/oxygenase large subunit (rbcL) gene, partial cds; chloroplast | 1074 | 1074 | 90% | 0.0 | 99.16% | DQ463101.1 |
| Equisetum arvense x Equisetum telmateia subsp. braunii isolate 40834 ribulose-1,5-bisphosphate carboxylase/oxygenase large subunit (rbcL) gene, partial cds; plastid | 1072 | 1072 | 90% | 0.0 | 99.16% | MH750161.1 |
| Equisetum arvense ribulose-1,5-bisphosphate carboxylase/oxygenase large subunit (rbcL) gene, partial cds; chloroplast | 1070 | 1070 | 91% | 0.0 | 98.99% | KM218345.1 |
| Equisetum hyemale ribulose-1,5-bisphosphate carboxylase/oxygenase large subunit (rbcL) gene, partial cds; chloroplast | 1070 | 1070 | 100% | 0.0 | 96.04% | DQ646001.1 |
| Equisetum sylvaticum chloroplast rbcL gene for ribulose-1,5-bisphosphate carboxylase/oxygenase large subunit, partial cds, specimen_voucher: TNS:776974 | 1064 | 1064 | 89% | 0.0 | 99.49% | AB574690.1 |
| Equisetum pratense chloroplast rbcL gene for ribulose-1,5-bisphosphate carboxylase/oxygenase large subunit, partial cds, specimen_voucher: TNS:1107843 | 1064 | 1064 | 89% | 0.0 | 99.49% | AB574687.1 |
| Equisetum scirpoides isolate 41089 ribulose-1,5-bisphosphate carboxylase/oxygenase large subunit (rbcL) gene, partial cds; plastid | 1061 | 1061 | 93% | 0.0 | 97.73% | MH750203.1 |
| Equisetum komarovii isolate 40830 ribulose-1,5-bisphosphate carboxylase/oxygenase large subunit (rbcL) gene, partial cds; plastid | 1061 | 1061 | 93% | 0.0 | 97.73% | MH750182.1 |
| Equisetum bogotense ribulose-1,5-bisphosphate carboxylase/oxygenase large subunit (rbcL) gene, partial cds; chloroplast gene for chloroplast product | 1059 | 1059 | 96% | 0.0 | 96.98% | AY226139.1 |
| Equisetum myriochaetum ribulose-1,5-bisphosphate carboxylase/oxygenase large subunit (rbcL) gene, partial cds; chloroplast gene for chloroplast product | 1055 | 1055 | 96% | 0.0 | 96.83% | AY226131.1 |
| Equisetum giganteum ribulose-1,5-bisphosphate carboxylase/oxygenase large subunit (rbcL) gene, partial cds; chloroplast gene for chloroplast product | 1055 | 1055 | 96% | 0.0 | 96.83% | AY226127.1 |
| Equisetum sp. Schuettpelz 1643 ribulose-1,5-bisphosphate carboxylase/oxygenase large subunit (rbcL) gene, partial cds; chloroplast | 1053 | 1053 | 96% | 0.0 | 96.83% | MW620286.1 |

| Description | Max Score | Total Score | Query Cover | E value | Per. Ident | Accession |
|---|---|---|---|---|---|---|
| Equisetum fluviatile chloroplast rbcL gene for ribulose-1,5-bisphosphate carboxylase/oxygenase large subunit, partial cds, specimen_voucher: TNS:776384 | 1053 | 1053 | 89% | 0.0 | 99.15% | AB574684.1 |
| Equisetum laevigatum ribulose-1,5-bisphosphate carboxylase/oxygenase large subunit (rbcL) gene, partial cds; chloroplast gene for chloroplast product | 1051 | 1051 | 95% | 0.0 | 96.82% | AY226130.1 |
| Equisetum scirpoides isolate 26090 ribulose-1,5-bisphosphate carboxylase/oxygenase large subunit (rbcL) gene, partial cds; plastid | 1050 | 1050 | 93% | 0.0 | 97.40% | MH750202.1 |
| Equisetum hyemale subsp. affine voucher OAC 94916 ribulose-1,5-bisphosphate carboxylase/oxygenase large subunit (rbcL) gene, partial cds; chloroplast | 1050 | 1050 | 96% | 0.0 | 96.67% | KF186500.1 |
| Equisetum scirpoides voucher AP248 ribulose-1,5-bisphosphate carboxylase/oxygenase large subunit (rbcL) gene, partial cds; chloroplast | 1050 | 1050 | 92% | 0.0 | 97.86% | HQ590084.1 |
| Equisetum hyemale subsp. affine ribulose-1,5-bisphosphate carboxylase/oxygenase large subunit (rbcL) gene, partial cds; chloroplast gene for chloroplast product | 1050 | 1050 | 96% | 0.0 | 96.67% | AY226128.1 |
| Equisetum giganteum isolate 40806 ribulose-1,5-bisphosphate carboxylase/oxygenase large subunit (rbcL) gene, partial cds; plastid | 1048 | 1048 | 95% | 0.0 | 96.81% | MH750172.1 |
| Equisetum giganteum isolate 26087 ribulose-1,5-bisphosphate carboxylase/oxygenase large subunit (rbcL) gene, partial cds; plastid | 1048 | 1048 | 95% | 0.0 | 96.81% | MH750170.1 |
| Equisetum ramosissimum voucher R825 ribulose-1,5-bisphosphate carboxylase/oxygenase large subunit (rbcl) gene, partial cds; chloroplast | 1048 | 1048 | 96% | 0.0 | 96.38% | KY656709.1 |
| Equisetum arvense chloroplast rbcL gene for ribulose-1,5-bisphosphate carboxylase/oxygenase large subunit, partial cds, specimen_voucher: TNS:763618 | 1048 | 1048 | 89% | 0.0 | 98.97% | AB574683.1 |
| Equisetum x ferrissii ribulose-1,5-bisphosphate carboxylase/oxygenase large subunit (rbcL) gene, partial cds; chloroplast | 1048 | 1048 | 95% | 0.0 | 96.81% | EU352292.1 |
| Equisetum sylvaticum voucher TJD-502 ribulose-1,5-bisphosphate carboxylase/oxygenase large subunit (rbcL) gene, partial cds; chloroplast | 1046 | 1046 | 86% | 0.0 | 99.82% | KJ841305.1 |
| Equisetum x ferrissii ribulose-1,5-bisphosphate carboxylase large subunit (rbcL) gene, partial cds; chloroplast gene for chloroplast product | 1046 | 1046 | 95% | 0.0 | 96.81% | AF313579.1 |
| Equisetum scirpoides isolate 10933 ribulose-1,5-bisphosphate carboxylase/oxygenase large subunit (rbcL) gene, partial cds; plastid | 1040 | 1040 | 92% | 0.0 | 97.69% | MH750201.1 |

| Description | Max Score | Total Score | Query Cover | E value | Per. Ident | Accession |
|---|---|---|---|---|---|---|
| Equisetum ramosissimum subsp. debile voucher G.M. Plunkett 3642 (NYBG) ribulose-1,5-bisphosphate carboxylase/oxygenase large subunit (rbcL) gene, partial cds; chloroplast | 1038 | 1038 | 96% | 0.0 | 96.35% | MT657901.1 |
| Equisetum ramosissimum subsp. debile ribulose-1,5-bisphosphate carboxylase/oxygenase large subunit (rbcL) gene, partial cds; chloroplast gene for chloroplast product | 1038 | 1038 | 96% | 0.0 | 96.35% | AY226132.1 |
| Equisetum arvense voucher TJD-508 ribulose-1,5-bisphosphate carboxylase/oxygenase large subunit (rbcL) gene, partial cds; chloroplast | 1037 | 1037 | 86% | 0.0 | 99.47% | KJ841301.1 |
| Equisetum myriochaetum isolate 40815 ribulose-1,5-bisphosphate carboxylase/oxygenase large subunit (rbcL) gene, partial cds; plastid | 1035 | 1035 | 95% | 0.0 | 96.63% | MH750185.1 |
| Equisetum arvense voucher TJD-503 ribulose-1,5-bisphosphate carboxylase/oxygenase large subunit (rbcL) gene, partial cds; chloroplast | 1035 | 1035 | 86% | 0.0 | 99.47% | KJ841302.1 |
| Equisetum arvense voucher TJD-504 ribulose-1,5-bisphosphate carboxylase/oxygenase large subunit (rbcL) gene, partial cds; chloroplast | 1033 | 1033 | 86% | 0.0 | 99.47% | KJ841300.1 |
| Equisetum hyemale voucher AP344 (OAC) ribulose-1,5-bisphosphate carboxylase/oxygenase large subunit (rbcL) gene, partial cds; chloroplast | 1029 | 1029 | 94% | 0.0 | 96.76% | EU677107.1 |
| Equisetum xylochaetum isolate 40614 ribulose-1,5-bisphosphate carboxylase/oxygenase large subunit (rbcL) gene, partial cds; plastid | 1027 | 1027 | 93% | 0.0 | 96.75% | MH750228.1 |
| Equisetum myriochaetum isolate 41080 ribulose-1,5-bisphosphate carboxylase/oxygenase large subunit (rbcL) gene, partial cds; plastid | 1027 | 1027 | 93% | 0.0 | 96.75% | MH750190.1 |
| Equisetum myriochaetum isolate 40826 ribulose-1,5-bisphosphate carboxylase/oxygenase large subunit (rbcL) gene, partial cds; plastid | 1027 | 1027 | 93% | 0.0 | 96.75% | MH750188.1 |
| Equisetum sylvaticum plastid partial rbcL gene for ribulose-1,5-biphosphate carboxylase/oxygenase large subunit, specimen voucher H823764 | 1027 | 1027 | 85% | 0.0 | 99.82% | HE574610.1 |
| Equisetum x schaffneri isolate 40813 ribulose-1,5-bisphosphate carboxylase/oxygenase large subunit (rbcL) gene, partial cds; plastid | 1026 | 1026 | 93% | 0.0 | 96.75% | MH750224.1 |
| Commelina diffusa voucher Q555 ribulose-1,5-bisphosphate carboxylase/oxygenase large subunit (rbcL) gene, partial cds; chloroplast | 1026 | 1026 | 86% | 0.0 | 99.29% | MH714073.1 |
| Equisetum giganteum isolate 40811 ribulose-1,5-bisphosphate carboxylase/oxygenase large subunit (rbcL) gene, partial cds; plastid | 1024 | 1024 | 93% | 0.0 | 96.74% | MH750176.1 |

| Description | Max Score | Total Score | Query Cover | E value | Per. Ident | Accession |
|---|---|---|---|---|---|---|
| Equisetum hyemale subsp. hyemale isolate 41088 ribulose-1,5-bisphosphate carboxylase/oxygenase large subunit (rbcL) gene, partial cds; plastid | 1022 | 1022 | 93% | 0.0 | 96.59% | MH750181.1 |
| Equisetum palustre voucher Chase 11641 (K) ribulose-1,5-bisphosphate carboxylase/oxygenase large subunit (rbcL) gene, partial cds; chloroplast | 1022 | 1022 | 84% | 0.0 | 100.00% | GQ248601.1 |
| Equisetum variegatum ribulose-1,5-bisphosphate carboxylase/oxygenase large subunit (rbcL) gene, partial cds; chloroplast gene for chloroplast product | 1022 | 1022 | 96% | 0.0 | 95.88% | AY226134.1 |
| Equisetum bogotense isolate 40800 ribulose-1,5-bisphosphate carboxylase/oxygenase large subunit (rbcL) gene, partial cds; plastid | 1020 | 1020 | 92% | 0.0 | 96.88% | MH750162.1 |
| Equisetum palustre voucher Gillespie_8830b ribulose-1,5-bisphosphate carboxylase/oxygenase large subunit (rbcL) gene, partial cds; chloroplast | 1020 | 1020 | 84% | 0.0 | 100.00% | KC482730.1 |
| Equisetum sylvaticum voucher WAB_1079140180 ribulose-1,5-bisphosphate carboxylase/oxygenase large subunit (rbcL) gene, partial cds; chloroplast | 1016 | 1016 | 84% | 0.0 | 99.82% | KJ593395.1 |
| Equisetum pratense voucher WAB_0132469185 ribulose-1,5-bisphosphate carboxylase/oxygenase large subunit (rbcL) gene, partial cds; chloroplast | 1016 | 1016 | 84% | 0.0 | 99.82% | KJ593391.1 |
| Equisetum giganteum isolate 40807 ribulose-1,5-bisphosphate carboxylase/oxygenase large subunit (rbcL) gene, partial cds; plastid | 1014 | 1014 | 92% | 0.0 | 96.72% | MH750173.1 |
| Equisetum sylvaticum voucher Gillespie_8524 ribulose-1,5-bisphosphate carboxylase/oxygenase large subunit (rbcL) gene, partial cds; chloroplast | 1014 | 1014 | 84% | 0.0 | 99.82% | KC482739.1 |
| Equisetum trachyodon isolate 41092 ribulose-1,5-bisphosphate carboxylase/oxygenase large subunit (rbcL) gene, partial cds; plastid | 1011 | 1011 | 93% | 0.0 | 96.41% | MH750227.1 |
| Equisetum ramosissimum subsp. debile isolate 40829 ribulose-1,5-bisphosphate carboxylase/oxygenase large subunit (rbcL) gene, partial cds; plastid | 1011 | 1011 | 93% | 0.0 | 96.27% | MH750200.1 |
| Equisetum myriochaetum isolate 40936 ribulose-1,5-bisphosphate carboxylase/oxygenase large subunit (rbcL) gene, partial cds; plastid | 1011 | 1011 | 92% | 0.0 | 96.71% | MH750189.1 |
| Equisetum hyemale subsp. affine isolate 40831 ribulose-1,5-bisphosphate carboxylase/oxygenase large subunit (rbcL) gene, partial cds; plastid | 1009 | 1009 | 92% | 0.0 | 96.55% | MH750177.1 |

## Alignments

Equisetum palustre chloroplast, partial genome

Sequence ID: **MT984458.1** Length: 5501 Number of Matches: 1
Range 1: 2214 to 2869

| Score | Expect | Identities | Gaps | Strand | Frame |
|---|---|---|---|---|---|
| 1195 bits(647) | 0.0() | 653/656(99%) | 0/656(0%) | Plus/Plus | |

Features:

```
Query  1     TGTCACCACAAACAGAGACTAAAGCAGGTGTTGGATTTAAAGCTGGTGTTAAAGATTATC  60
             ||||||||||||| ||||| ||||||||||||||||||||||||||||||||||||||||
Sbjct  2214  TGTCACCACAAACGGAGACCAAAGCAGGTGTTGGATTTAAAGCTGGTGTTAAAGATTATC  2273

Query  61    GATTGACTTATTTTACTCCAGATTATGAAACCAAAGATACCGATATTTTAGCAGCATTCC  120
             ||||||||||||||||||||||||||||||||||||||||||||||||||||||||||||
Sbjct  2274  GATTGACTTATTTTACTCCAGATTATGAAACCAAAGATACCGATATTTTAGCAGCATTCC  2333

Query  121   GTATGACTCCTCAACCGGGAGTACCACCGGAAGAAGCAGGAGCAGCCGTAGCTGCTGAAT  180
             ||||||||||||||||||||||||||||||||||||||||||||||||||||||||||||
Sbjct  2334  GTATGACTCCTCAACCGGGAGTACCACCGGAAGAAGCAGGAGCAGCCGTAGCTGCTGAAT  2393

Query  181   CGTCCACGGGCACCTGGACTACCGTATGGACAGATGGACTTACTAGTCTTGATCGATATA  240
             ||||||||||||||||||||||||||||||||||||||||||||||||||||||||||||
Sbjct  2394  CGTCCACGGGCACCTGGACTACCGTATGGACAGATGGACTTACTAGTCTTGATCGATATA  2453

Query  241   AAGGTCGATGCTATAATATTGAACCTGTTGCTGGAGAAGATAATCAATTCATAGCTTATG  300
             ||||||||||||||||||||||||||||||||||||||||||||||||||||||||||||
Sbjct  2454  AAGGTCGATGCTATAATATTGAACCTGTTGCTGGAGAAGATAATCAATTCATAGCTTATG  2513

Query  301   TAGCCTATCCTTTAGATCTTTTTGAAGAAGGTTCTGTTACCAATCTTTTTACTTCAATTG  360
             ||||||||||||||||||||||||||||||||||||||||||||||||||||||||||||
Sbjct  2514  TAGCCTATCCTTTAGATCTTTTTGAAGAAGGTTCTGTTACCAATCTTTTTACTTCAATTG  2573

Query  361   TTGGTAATGTTTTCGGCTTCAAAGCTCTACGTGCTTTACGTTTAGAAGATTTAAGAATTC  420
             ||||||||||||||||||||||||||||||||||||||||||||||||||||||||||||
Sbjct  2574  TTGGTAATGTTTTCGGCTTCAAAGCTCTACGTGCTTTACGTTTAGAAGATTTAAGAATTC  2633

Query  421   CTCCTGCTTACTCCAAAACTTTTATAGGACCACCCCACGGTATCCAGGTTGAAAGAGATA  480
             ||||||||||||||||||||||||||||||||||||||||||||||||||||||||||||
Sbjct  2634  CTCCTGCTTACTCCAAAACTTTTATAGGACCACCCCACGGTATCCAGGTTGAAAGAGATA  2693

Query  481   AGTTAAACAAATATGGTCGTCCTTTATTAGGTTGTACAATTAAACCAAAATTGGGACTAT  540
             ||||||||||||||||||||||||||||||||||||||||||||||||||||||||||||
Sbjct  2694  AGTTAAACAAATATGGTCGTCCTTTATTAGGTTGTACAATTAAACCAAAATTGGGACTAT  2753

Query  541   CTGCTAAAAACTATGGTAGGGCTGTTTATGAATGTCTTCGTGGTGGACTTGATTTCACCA  600
             ||||||||||||||||||||||||||||||||||||||||||||||||||||||||||||
Sbjct  2754  CTGCTAAAAACTATGGTAGGGCTGTTTATGAATGTCTTCGTGGTGGACTTGATTTCACCA  2813

Query  601   AAGATGATGAGAATGTAAACTCTCAACCCTTTATGCGTTGGAGAGATCGATTCTTA  656
             ||||||||||||||||||||||||||||||||||||||||||||||||| ||||||
Sbjct  2814  AAGATGATGAGAATGTAAACTCTCAACCCTTTATGCGTTGGAGAGATCGTTTCTTA  2869
```

Equisetum arvense chloroplast, complete genome
Sequence ID: **GU191334.1** Length: 133309 Number of Matches: 1
Range 1: 27315 to 27970

| Score | Expect | Identities | Gaps | Strand | Frame |
|---|---|---|---|---|---|
| 1173 bits(635) | 0.0() | 649/656(99%) | 0/656(0%) | Plus/Minus | |

Features:

```
Query  1      TGTCACCACAAACAGAGACTAAAGCAGGTGTTGGATTTAAAGCTGGTGTTAAAGATTATC  60
              ||||||||||||| ||||| ||||||||||||||||||||||||||||||||||||||||
Sbjct  27970  TGTCACCACAAACGGAGACCAAAGCAGGTGTTGGATTTAAAGCTGGTGTTAAAGATTATC  27911

Query  61     GATTGACTTATTTTACTCCAGATTATGAAACCAAAGATACCGATATTTTAGCAGCATTCC  120
              ||||||||||||||||||||||||||||||||||||||||||||||||||||||||||||
Sbjct  27910  GATTGACTTATTTTACTCCAGATTATGAAACCAAAGATACCGATATTTTAGCAGCATTCC  27851

Query  121    GTATGACTCCTCAACCGGGAGTACCACCGGAAGAAGCAGGAGCAGCCGTAGCTGCTGAAT  180
              ||||||||||||||||||||||||||||||||||||||||||||||||||||||||||||
Sbjct  27850  GTATGACTCCTCAACCGGGAGTACCACCGGAAGAAGCAGGAGCAGCCGTAGCTGCTGAAT  27791

Query  181    CGTCCACGGGCACCTGGACTACCGTATGGACAGATGGACTTACTAGTCTTGATCGATATA  240
              ||||||||||||||||||||||||||||||||||||||||||||||||||||||||||||
Sbjct  27790  CGTCCACGGGCACCTGGACTACCGTATGGACAGATGGACTTACTAGTCTTGATCGATATA  27731

Query  241    AAGGTCGATGCTATAATATTGAACCTGTTGCTGGAGAAGATAATCAATTCATAGCTTATG  300
              ||||||||||||||||||||||||||||||||||||||||||||||||||||||||||||
Sbjct  27730  AAGGTCGATGCTATAATATTGAACCTGTTGCTGGAGAAGATAATCAATTCATAGCTTATG  27671

Query  301    TAGCCTATCCTTTAGATCTTTTTGAAGAAGGTTCTGTTACCAATCTTTTTACTTCAATTG  360
              ||||||| ||||||||||||||||||||||||||||||||||||||||||||||||||||
Sbjct  27670  TAGCCTACCCTTTAGATCTTTTTGAAGAAGGTTCTGTTACCAATCTTTTTACTTCAATTG  27611
```

```
Query  361    TTGGTAATGTTTTCGGCTTCAAAGCTCTACGTGCTTTACGTTTAGAAGATTTAAGAATTC  420
              ||||||||||||||||||||||||||||||||||||||||||||||||||||||||||||
Sbjct  27610  TTGGTAATGTTTTCGGCTTCAAAGCTCTACGTGCTTTACGTTTAGAAGATTTAAGAATTC  27551

Query  421    CTCCTGCTTACTCCAAAACTTTTATAGGACCACCCCACGGTATCCAGGTTGAAAGAGATA  480
              ||||||||||||||||||||||||||||||||||||||||||||||||||||||||||||
Sbjct  27550  CTCCTGCTTACTCCAAAACTTTTATAGGACCACCCCACGGTATCCAGGTTGAAAGAGATA  27491

Query  481    AGTTAAACAAATATGGTCGTCCTTTATTAGGTTGTACAATTAAACCAAAATTGGGACTAT  540
              ||||||||||||| ||||||||||||||||||||||||||||||||||||||||||||||
Sbjct  27490  AGTTAAACAAATACGGTCGTCCTTTATTAGGTTGTACAATTAAACCAAAATTGGGACTAT  27431

Query  541    CTGCTAAAAACTATGGTAGGGCTGTTTATGAATGTCTTCGTGGTGGACTTGATTTCACCA  600
              ||||||||||||||||||| |||||||||||||||||||||||||| |||||||||||||
Sbjct  27430  CTGCTAAAAACTATGGTAGAGCTGTTTATGAATGTCTTCGTGGTGGGCTTGATTTCACCA  27371

Query  601    AAGATGATGAGAATGTAAACTCTCAACCCTTTATGCGTTGGAGAGATCGATTCTTA  656
              ||||||||||||||||||||||||||||||||||||||||||||||||| ||||||
Sbjct  27370  AAGATGATGAGAATGTAAACTCTCAACCCTTTATGCGTTGGAGAGATCGTTTCTTA  27315
```

Equisetum arvense chloroplast ribulosebisphosphate carboxylase (rbcL) gene, complete cds

Sequence ID: **L11053.1** Length: 1587 Number of Matches: 1

Range 1: 74 to 729

| Score | Expect | Identities | Gaps | Strand | Frame |
|---|---|---|---|---|---|
| 1173 bits(635) | 0.0() | 649/656(99%) | 0/656(0%) | Plus/Plus | |

Features:

```
Query  1    TGTCACCACAAACAGAGACTAAAGCAGGTGTTGGATTTAAAGCTGGTGTTAAAGATTATC  60
            ||||||||||||| ||||| ||||||||||||||||||||||||||||||||||||||||
Sbjct  74   TGTCACCACAAACGGAGACCAAAGCAGGTGTTGGATTTAAAGCTGGTGTTAAAGATTATC  133

Query  61   GATTGACTTATTTTACTCCAGATTATGAAACCAAAGATACCGATATTTTAGCAGCATTCC  120
            ||||||||||||||||||||||||||||||||||||||||||||||||||||||||||||
Sbjct  134  GATTGACTTATTTTACTCCAGATTATGAAACCAAAGATACCGATATTTTAGCAGCATTCC  193

Query  121  GTATGACTCCTCAACCGGGAGTACCACCGGAAGAAGCAGGAGCAGCCGTAGCTGCTGAAT  180
            ||||||||||||||||||||||||||||||||||||||||||||||||||||||||||||
Sbjct  194  GTATGACTCCTCAACCGGGAGTACCACCGGAAGAAGCAGGAGCAGCCGTAGCTGCTGAAT  253

Query  181  CGTCCACGGGCACCTGGACTACCGTATGGACAGATGGACTTACTAGTCTTGATCGATATA  240
            ||||||||||||||||||||||||||||||||||||||||||||||||||||||||||||
Sbjct  254  CGTCCACGGGCACCTGGACTACCGTATGGACAGATGGACTTACTAGTCTTGATCGATATA  313

Query  241  AAGGTCGATGCTATAATATTGAACCTGTTGCTGGAGAAGATAATCAATTCATAGCTTATG  300
            ||||||||||||||||||||||||||||||||||||||||||||||||||||||||||||
Sbjct  314  AAGGTCGATGCTATAATATTGAACCTGTTGCTGGAGAAGATAATCAATTCATAGCTTATG  373

Query  301  TAGCCTATCCTTTAGATCTTTTTGAAGAAGGTTCTGTTACCAATCTTTTTACTTCAATTG  360
            ||||||| ||||||||||||||||||||||||||||||||||||||||||||||||||||
Sbjct  374  TAGCCTACCCTTTAGATCTTTTTGAAGAAGGTTCTGTTACCAATCTTTTTACTTCAATTG  433

Query  361  TTGGTAATGTTTTCGGCTTCAAAGCTCTACGTGCTTTACGTTTAGAAGATTTAAGAATTC  420
            ||||||||||||||||||||||||||||||||||||||||||||||||||||||||||||
Sbjct  434  TTGGTAATGTTTTCGGCTTCAAAGCTCTACGTGCTTTACGTTTAGAAGATTTAAGAATTC  493

Query  421  CTCCTGCTTACTCCAAAACTTTTATAGGACCACCCCACGGTATCCAGGTTGAAAGAGATA  480
            ||||||||||||||||||||||||||||||||||||||||||||||||||||||||||||
Sbjct  494  CTCCTGCTTACTCCAAAACTTTTATAGGACCACCCCACGGTATCCAGGTTGAAAGAGATA  553

Query  481  AGTTAAACAAATATGGTCGTCCTTTATTAGGTTGTACAATTAAACCAAAATTGGGACTAT  540
            ||||||||||||| ||||||||||||||||||||||||||||||||||||||||||||||
Sbjct  554  AGTTAAACAAATACGGTCGTCCTTTATTAGGTTGTACAATTAAACCAAAATTGGGACTAT  613

Query  541  CTGCTAAAAACTATGGTAGGGCTGTTTATGAATGTCTTCGTGGTGGACTTGATTTCACCA  600
            ||||||||||||||||||| |||||||||||||||||||||||||| |||||||||||||
Sbjct  614  CTGCTAAAAACTATGGTAGAGCTGTTTATGAATGTCTTCGTGGTGGGCTTGATTTCACCA  673

Query  601  AAGATGATGAGAATGTAAACTCTCAACCCTTTATGCGTTGGAGAGATCGATTCTTA  656
            ||||||||||||||||||||||||||||||||||||||||||||||||| ||||||
Sbjct  674  AAGATGATGAGAATGTAAACTCTCAACCCTTTATGCGTTGGAGAGATCGTTTCTTA  729
```

Equisetum arvense plastid, complete genome

Sequence ID: **JN968380.1** Length: 132726 Number of Matches: 1

Range 1: 64959 to 65614

| Score | Expect | Identities | Gaps | Strand | Frame |
|---|---|---|---|---|---|
| 1168 bits(632) | 0.0() | 648/656(99%) | 0/656(0%) | Plus/Plus | |

Features:

```
Query  1      TGTCACCACAAACAGAGACTAAAGCAGGTGTTGGATTTAAAGCTGGTGTTAAAGATTATC  60
              ||||||||||||| ||||| ||||||||||||||||||||||||||||||||||||||||
Sbjct  64959  TGTCACCACAAACGGAGACCAAAGCAGGTGTTGGATTTAAAGCTGGTGTTAAAGATTATC  65018

Query  61     GATTGACTTATTTTACTCCAGATTATGAAACCAAAGATACCGATATTTTAGCAGCATTCC  120
              ||||||||||||||||||||||||||||||||||||||||||||||||||||||||||||
Sbjct  65019  GATTGACTTATTTTACTCCAGATTATGAAACCAAAGATACCGATATTTTAGCAGCATTCC  65078

Query  121    GTATGACTCCTCAACCGGGAGTACCACCGGAAGAAGCAGGAGCAGCCGTAGCTGCTGAAT  180
              ||||||||||||||||||||||||||||||||||||||||||||||||||||||||||||
Sbjct  65079  GTATGACTCCTCAACCGGGAGTACCACCGGAAGAAGCAGGAGCAGCCGTAGCTGCTGAAT  65138

Query  181    CGTCCACGGGCACCTGGACTACCGTATGGACAGATGGACTTACTAGTCTTGATCGATATA  240
              ||||||||||||||||||||||||||||||||||||||||||||||||||||||||||||
Sbjct  65139  CGTCCACGGGCACCTGGACTACCGTATGGACAGATGGACTTACTAGTCTTGATCGATATA  65198

Query  241    AAGGTCGATGCTATAATATTGAACCTGTTGCTGGAGAAGATAATCAATTCATAGCTTATG  300
              ||||||||||||||||||||||||||||||||||||||||||||||||||||||||||||
Sbjct  65199  AAGGTCGATGCTATAATATTGAACCTGTTGCTGGAGAAGATAATCAATTCATAGCTTATG  65258

Query  301    TAGCCTATCCTTTAGATCTTTTTGAAGAAGGTTCTGTTACCAATCTTTTTACTTCAATTG  360
              ||||||| ||||||||||||||||||||||||||||||||||||||||||||||||||||
Sbjct  65259  TAGCCTACCCTTTAGATCTTTTTGAAGAAGGTTCTGTTACCAATCTTTTTACTTCAATTG  65318

Query  361    TTGGTAATGTTTTCGGCTTCAAAGCTCTACGTGCTTTACGTTTAGAAGATTTAAGAATTC  420
              |||||||||||||||| |||||||||||||||||||||||||||||||||||||||||||
Sbjct  65319  TTGGTAATGTTTTCGGATTCAAAGCTCTACGTGCTTTACGTTTAGAAGATTTAAGAATTC  65378

Query  421    CTCCTGCTTACTCCAAAACTTTTATAGGACCACCCCACGGTATCCAGGTTGAAAGAGATA  480
              ||||||||||||||||||||||||||||||||||||||||||||||||||||||||||||
Sbjct  65379  CTCCTGCTTACTCCAAAACTTTTATAGGACCACCCCACGGTATCCAGGTTGAAAGAGATA  65438

Query  481    AGTTAAACAAATATGGTCGTCCTTTATTAGGTTGTACAATTAAACCAAAATTGGGACTAT  540
              ||||||||||||| ||||||||||||||||||||||||||||||||||||||||||||||
Sbjct  65439  AGTTAAACAAATACGGTCGTCCTTTATTAGGTTGTACAATTAAACCAAAATTGGGACTAT  65498

Query  541    CTGCTAAAAACTATGGTAGGGCTGTTTATGAATGTCTTCGTGGTGGACTTGATTTCACCA  600
              ||||||||||||||||||| |||||||||||||||||||||||||| |||||||||||||
Sbjct  65499  CTGCTAAAAACTATGGTAGAGCTGTTTATGAATGTCTTCGTGGTGGGCTTGATTTCACCA  65558

Query  601    AAGATGATGAGAATGTAAACTCTCAACCCTTTATGCGTTGGAGAGATCGATTCTTA  656
              ||||||||||||||||||||||||||||||||||||||||||||||||| ||||||
Sbjct  65559  AAGATGATGAGAATGTAAACTCTCAACCCTTTATGCGTTGGAGAGATCGTTTCTTA  65614
```

Equisetum palustre ribulose-1,5-bisphosphate carboxylase/oxygenase large subunit (rbcL) gene, partial cds; chloroplast gene for chloroplast product

Sequence ID: **AY226138.1** Length: 1325 Number of Matches: 1

Range 1: 1 to 631

| Score | Expect | Identities | Gaps | Strand | Frame |
|---|---|---|---|---|---|
| 1160 bits(628) | 0.0() | 630/631(99%) | 0/631(0%) | Plus/Plus | |

Features:

```
Query  26   AGGTGTTGGATTTAAAGCTGGTGTTAAAGATTATCGATTGACTTATTTTACTCCAGATTA  85
            ||||||||||||||||||||||||||||||||||||||||||||||||||||||||||||
Sbjct  1    AGGTGTTGGATTTAAAGCTGGTGTTAAAGATTATCGATTGACTTATTTTACTCCAGATTA  60

Query  86   TGAAACCAAAGATACCGATATTTTAGCAGCATTCCGTATGACTCCTCAACCGGGAGTACC  145
            ||||||||||||||||||||||||||||||||||||||||||||||||||||||||||||
Sbjct  61   TGAAACCAAAGATACCGATATTTTAGCAGCATTCCGTATGACTCCTCAACCGGGAGTACC  120

Query  146  ACCGGAAGAAGCAGGAGCAGCCGTAGCTGCTGAATCGTCCACGGGCACCTGGACTACCGT  205
            ||||||||||||||||||||||||||||||||||||||||||||||||||||||||||||
Sbjct  121  ACCGGAAGAAGCAGGAGCAGCCGTAGCTGCTGAATCGTCCACGGGCACCTGGACTACCGT  180

Query  206  ATGGACAGATGGACTTACTAGTCTTGATCGATATAAAGGTCGATGCTATAATATTGAACC  265
            ||||||||||||||||||||||||||||||||||||||||||||||||||||||||||||
Sbjct  181  ATGGACAGATGGACTTACTAGTCTTGATCGATATAAAGGTCGATGCTATAATATTGAACC  240

Query  266  TGTTGCTGGAGAAGATAATCAATTCATAGCTTATGTAGCCTATCCTTTAGATCTTTTTGA  325
            ||||||||||||||||||||||||||||||||||||||||||||||||||||||||||||
Sbjct  241  TGTTGCTGGAGAAGATAATCAATTCATAGCTTATGTAGCCTATCCTTTAGATCTTTTTGA  300

Query  326  AGAAGGTTCTGTTACCAATCTTTTTACTTCAATTGTTGGTAATGTTTTCGGCTTCAAAGC  385
            ||||||||||||||||||||||||||||||||||||||||||||||||||||||||||||
Sbjct  301  AGAAGGTTCTGTTACCAATCTTTTTACTTCAATTGTTGGTAATGTTTTCGGCTTCAAAGC  360

Query  386  TCTACGTGCTTTACGTTTAGAAGATTTAAGAATTCCTCCTGCTTACTCCAAAACTTTTAT  445
            ||||||||||||||||||||||||||||||||||||||||||||||||||||||||||||
Sbjct  361  TCTACGTGCTTTACGTTTAGAAGATTTAAGAATTCCTCCTGCTTACTCCAAAACTTTTAT  420

Query  446  AGGACCACCCCACGGTATCCAGGTTGAAAGAGATAAGTTAAACAAATATGGTCGTCCTTT  505
            ||||||||||||||||||||||||||||||||||||||||||||||||||||||||||||
```

```
Sbjct  421  AGGACCACCCCACGGTATCCAGGTTGAAAGAGATAAGTTAAACAAATATGGTCGTCCTTT  480

Query  506  ATTAGGTTGTACAATTAAACCAAAATTGGGACTATCTGCTAAAAACTATGGTAGGGCTGT  565
            ||||||||||||||||||||||||||||||||||||||||||||||||||||||||||||
Sbjct  481  ATTAGGTTGTACAATTAAACCAAAATTGGGACTATCTGCTAAAAACTATGGTAGGGCTGT  540

Query  566  TTATGAATGTCTTCGTGGTGGACTTGATTTCACCAAAGATGATGAGAATGTAAACTCTCA  625
            ||||||||||||||||||||||||||||||||||||||||||||||||||||||||||||
Sbjct  541  TTATGAATGTCTTCGTGGTGGACTTGATTTCACCAAAGATGATGAGAATGTAAACTCTCA  600

Query  626  ACCCTTTATGCGTTGGAGAGATCGATTCTTA  656
            |||||||||||||||||||||||| ||||||
Sbjct  601  ACCCTTTATGCGTTGGAGAGATCGTTTCTTA  631
```


BLAST is a registered trademark of the National Library of Medicine

Support center Mailing list YouTube

- National Library Of Medicine
- National Institutes Of Health
- U.S. Department of Health & Human Services
- USA.gov

**NCBI**

*National Center for Biotechnology Information, U.S. National Library of Medicine 8600 Rockville Pike, Bethesda MD, 20894 USA*

Policies and Guidelines | Contact

HHS Vulnerability Disclosure
