# Supplementary material for: A Multi-Pronged Technique for Identifying Equisetum palustre and Equisetum arvense—Combining HPTLC, HPLC-ESI-MS/MS and Optimized DNA Barcoding Techniques
Source: Plants (Basel). 2022 Sep 28;11(19):2562. doi: 10.3390/plants11192562 (PMC9573248; doi:10.3390/plants11192562)
Supplement: Supplementary file 1 [file plants-11-02562-s001.zip › plants-1932149 supplementary/Horsetail Supplementary Figures/Figure S24 (P6, 82712220323-1).pdf]

BLAST Results

Questions/comments

Job title: Nucleotide Sequence

**RID**	J1CB9EUS016 (Expires on 09-15 00:35 am)		
**Query ID**	lcl|Query_49497	**Database Name**	nt
**Description**	None	**Description**	Nucleotide collection (nt)
**Molecule type**	dna	**Program**	BLASTN 2.13.0+
**Query Length**	665		

Graphic Summary

Distribution of the top 100 Blast Hits on 100 subject sequences

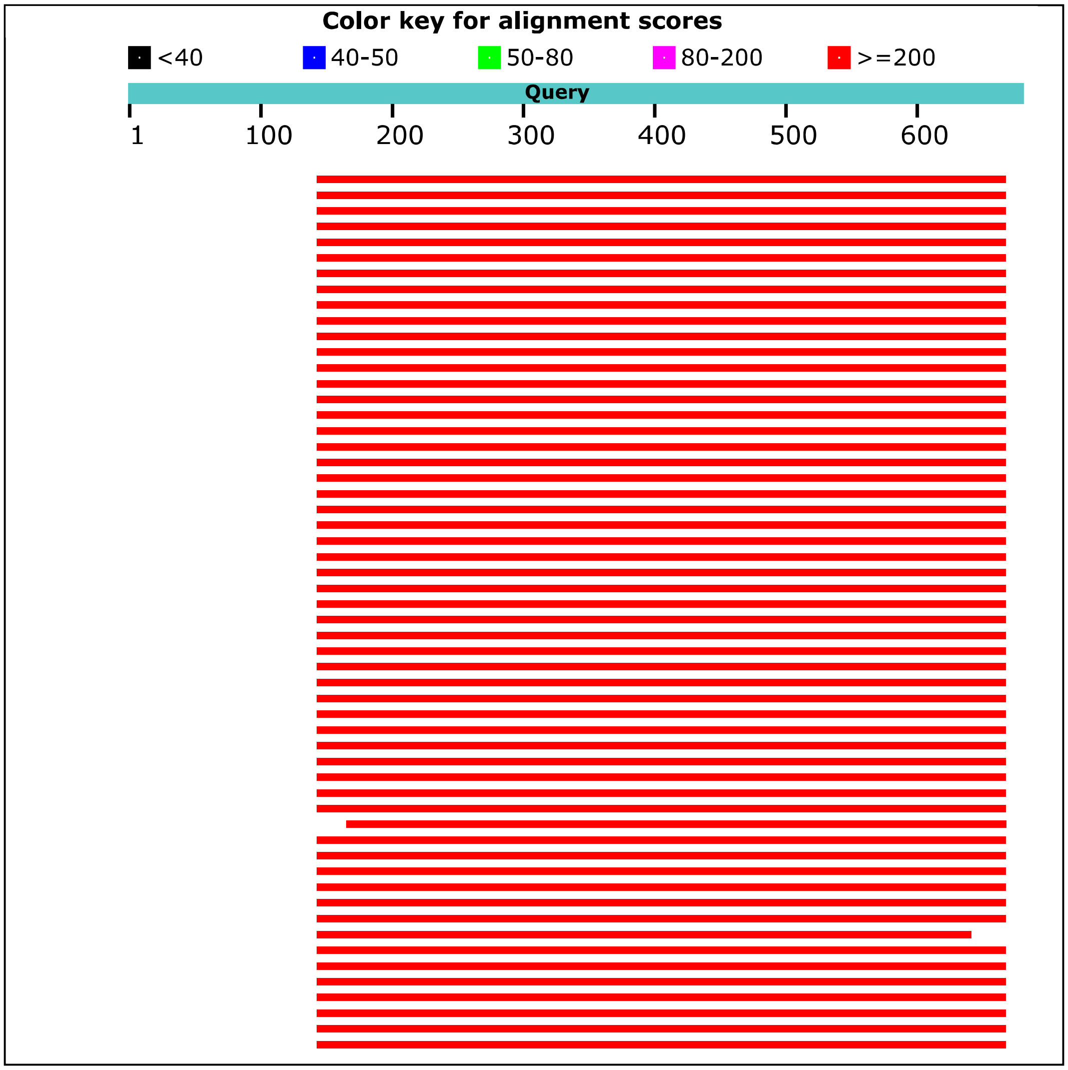

Descriptions

Sequences producing significant alignments:

Description	Max Score	Total Score	Query Cover	E value	Per. Ident	Accession
Equisetum palustre isolate 39349 ribulose-1,5-bisphosphate carboxylase/oxygenase large subunit (rbcL) gene, partial cds; plastid	900	900	78%	0.0	97.89%	MH750192.1
Equisetum palustre isolate 17671 ribulose-1,5-bisphosphate carboxylase/oxygenase large subunit (rbcL) gene, partial cds; plastid	900	900	78%	0.0	97.89%	MH750191.1
Equisetum palustre chloroplast, partial genome	900	900	78%	0.0	97.89%	MT984458.1
Equisetum palustre chloroplast rbcL gene for ribulose-1,5-bisphosphate carboxylase/oxygenase large subunit, partial cds, specimen_voucher: TNS:776981	900	900	78%	0.0	97.89%	AB574686.1
Equisetum palustre ribulose-1,5-bisphosphate carboxylase/oxygenase large subunit (rbcL) gene, partial cds; chloroplast gene for chloroplast product	900	900	78%	0.0	97.89%	AY226138.1
Equisetum sylvaticum isolate 41081 ribulose-1,5-bisphosphate carboxylase/oxygenase large subunit (rbcL) gene, partial cds; plastid	889	889	78%	0.0	97.51%	MH750205.1
Equisetum sylvaticum isolate 11640 ribulose-1,5-bisphosphate carboxylase/oxygenase large subunit (rbcL) gene, partial cds; plastid	889	889	78%	0.0	97.51%	MH750204.1
Equisetum pratense isolate 41087 ribulose-1,5-bisphosphate carboxylase/oxygenase large subunit (rbcL) gene, partial cds; plastid	889	889	78%	0.0	97.51%	MH750195.1
Equisetum pratense isolate 41086 ribulose-1,5-bisphosphate carboxylase/oxygenase large subunit (rbcL) gene, partial cds; plastid	889	889	78%	0.0	97.51%	MH750194.1
Equisetum pratense isolate 39348 ribulose-1,5-bisphosphate carboxylase/oxygenase large subunit (rbcL) gene, partial cds; plastid	889	889	78%	0.0	97.51%	MH750193.1
Equisetum sylvaticum chloroplast rbcL gene for ribulose-1,5-bisphosphate carboxylase/oxygenase large subunit, partial cds, specimen_voucher: TNS:776974	889	889	78%	0.0	97.51%	AB574690.1
Equisetum pratense chloroplast rbcL gene for ribulose-1,5-bisphosphate carboxylase/oxygenase large subunit, partial cds, specimen_voucher: TNS:1107843	889	889	78%	0.0	97.51%	AB574687.1
Equisetum pratense ribulose-1,5-bisphosphate carboxylase/oxygenase large subunit (rbcL) gene, partial cds; chloroplast gene for chloroplast product	889	889	78%	0.0	97.51%	AY226137.1

Description	Max Score	Total Score	Query Cover	E value	Per. Ident	Accession
Equisetum sylvaticum ribulose-1,5-bisphosphate carboxylase/oxygenase large subunit (rbcL) gene, partial cds; chloroplast gene for chloroplast product	889	889	78%	0.0	97.51%	AY226136.1
Equisetum x litorale isolate 41084 ribulose-1,5-bisphosphate carboxylase/oxygenase large subunit (rbcL) gene, partial cds; plastid	878	878	78%	0.0	97.13%	MH750222.1
Equisetum x fontqueri isolate 26093 ribulose-1,5-bisphosphate carboxylase/oxygenase large subunit (rbcL) gene, partial cds; plastid	878	878	78%	0.0	97.13%	MH750221.1
Equisetum x dycei isolate 26083 ribulose-1,5-bisphosphate carboxylase/oxygenase large subunit (rbcL) gene, partial cds; plastid	878	878	78%	0.0	97.13%	MH750220.1
Equisetum telmateia subsp. telmateia isolate 11642 ribulose-1,5-bisphosphate carboxylase/oxygenase large subunit (rbcL) gene, partial cds; plastid	878	878	78%	0.0	97.13%	MH750210.1
Equisetum telmateia subsp. braunii isolate 40836 ribulose-1,5-bisphosphate carboxylase/oxygenase large subunit (rbcL) gene, partial cds; plastid	878	878	78%	0.0	97.13%	MH750209.1
Equisetum telmateia subsp. braunii isolate 40832 ribulose-1,5-bisphosphate carboxylase/oxygenase large subunit (rbcL) gene, partial cds; plastid	878	878	78%	0.0	97.13%	MH750208.1
Equisetum telmateia subsp. braunii isolate 40828 ribulose-1,5-bisphosphate carboxylase/oxygenase large subunit (rbcL) gene, partial cds; plastid	878	878	78%	0.0	97.13%	MH750207.1
Equisetum telmateia subsp. braunii isolate 40817 ribulose-1,5-bisphosphate carboxylase/oxygenase large subunit (rbcL) gene, partial cds; plastid	878	878	78%	0.0	97.13%	MH750206.1
Equisetum fluviatile isolate 41076 ribulose-1,5-bisphosphate carboxylase/oxygenase large subunit (rbcL) gene, partial cds; plastid	878	878	78%	0.0	97.13%	MH750169.1
Equisetum fluviatile isolate 41075 ribulose-1,5-bisphosphate carboxylase/oxygenase large subunit (rbcL) gene, partial cds; plastid	878	878	78%	0.0	97.13%	MH750168.1
Equisetum diffusum isolate 40689 ribulose-1,5-bisphosphate carboxylase/oxygenase large subunit (rbcL) gene, partial cds; plastid	878	878	78%	0.0	97.13%	MH750166.1
Equisetum arvense x Equisetum telmateia subsp. braunii isolate 40834 ribulose-1,5-bisphosphate carboxylase/oxygenase large subunit (rbcL) gene, partial cds; plastid	878	878	78%	0.0	97.13%	MH750161.1
Equisetum arvense subsp. boreale isolate 41074 ribulose-1,5-bisphosphate carboxylase/oxygenase large subunit (rbcL) gene, partial cds; plastid	878	878	78%	0.0	97.13%	MH750160.1
Equisetum arvense subsp. boreale isolate 41073 ribulose-1,5-bisphosphate carboxylase/oxygenase large subunit (rbcL) gene, partial cds; plastid	878	878	78%	0.0	97.13%	MH750159.1

Description	Max Score	Total Score	Query Cover	E value	Per. Ident	Accession
Equisetum arvense subsp. arvense isolate 41071 ribulose-1,5-bisphosphate carboxylase/oxygenase large subunit (rbcL) gene, partial cds; plastid	878	878	78%	0.0	97.13%	MH750158.1
Equisetum arvense subsp. arvense isolate 40833 ribulose-1,5-bisphosphate carboxylase/oxygenase large subunit (rbcL) gene, partial cds; plastid	878	878	78%	0.0	97.13%	MH750157.1
Equisetum arvense subsp. arvense isolate 26085 ribulose-1,5-bisphosphate carboxylase/oxygenase large subunit (rbcL) gene, partial cds; plastid	878	878	78%	0.0	97.13%	MH750156.1
Equisetum arvense subsp. arvense isolate 26084 ribulose-1,5-bisphosphate carboxylase/oxygenase large subunit (rbcL) gene, partial cds; plastid	878	878	78%	0.0	97.13%	MH750155.1
Equisetum environmental sample isolate EDNA16-0043490 ribulose-1,5-bisphosphate carboxylase/oxygenase large subunit (rbcL) gene, partial cds; chloroplast	878	878	78%	0.0	97.13%	MT197555.1
Equisetum fluviatile chloroplast rbcL gene for ribulose-1,5-bisphosphate carboxylase/oxygenase large subunit, partial cds, specimen_voucher: TNS:776384	878	878	78%	0.0	97.13%	AB574684.1
Equisetum arvense chloroplast, complete genome	878	878	78%	0.0	97.13%	GU191334.1
Equisetum fluviatile ribulose-1,5-bisphosphate carboxylase/oxygenase large subunit (rbcL) gene, partial cds; chloroplast	878	878	78%	0.0	97.13%	DQ463101.1
Equisetum fluviatile ribulose-1,5-bisphosphate carboxylase/oxygenase large subunit (rbcL) gene, partial cds; chloroplast gene for chloroplast product	878	878	78%	0.0	97.13%	AY226142.1
Equisetum arvense ribulose-1,5-bisphosphate carboxylase/oxygenase large subunit (rbcL) gene, partial cds; chloroplast gene for chloroplast product	878	878	78%	0.0	97.13%	AY226140.1
Equisetum telmateia subsp. braunii ribulose-1,5-bisphosphate carboxylase/oxygenase large subunit (rbcL) gene, partial cds; chloroplast gene for chloroplast product	878	878	78%	0.0	97.13%	AY226135.1
Equisetum telmateia ribulose-1,5-bisphosphate carboxylase large subunit (rbcL) gene, partial cds; chloroplast gene for chloroplast product	878	878	78%	0.0	97.13%	AF313580.1
Equisetum arvense chloroplast ribulosebisphosphate carboxylase (rbcL) gene, complete cds	878	878	78%	0.0	97.13%	L11053.1
Equisetum telmateia subsp. telmateia isolate 41082 ribulose-1,5-bisphosphate carboxylase/oxygenase large subunit (rbcL) gene, partial cds; plastid	874	874	75%	0.0	98.40%	MH750211.1
Equisetum arvense ribulose-1,5-bisphosphate carboxylase/oxygenase large subunit (rbcL) gene, partial cds; chloroplast	872	872	78%	0.0	96.93%	KM218345.1

Description	Max Score	Total Score	Query Cover	E value	Per. Ident	Accession
Equisetum environmental sample isolate EDNA16-0043491b ribulose-1,5-bisphosphate carboxylase/oxygenase large subunit (rbcL) gene, partial cds; chloroplast	872	872	78%	0.0	96.93%	MT197548.1
Equisetum arvense plastid, complete genome	872	872	78%	0.0	96.93%	JN968380.1
Equisetum arvense chloroplast rbcL gene for ribulose-1,5-bisphosphate carboxylase/oxygenase large subunit, partial cds, specimen_voucher: TNS:763618	872	872	78%	0.0	96.93%	AB574683.1
Equisetum diffusum isolate 40804 ribulose-1,5-bisphosphate carboxylase/oxygenase large subunit (rbcL) gene, partial cds; plastid	867	867	78%	0.0	96.74%	MH750167.1
Equisetum diffusum ribulose-1,5-bisphosphate carboxylase/oxygenase large subunit (rbcL) gene, partial cds; chloroplast gene for chloroplast product	867	867	78%	0.0	96.74%	AY226141.1
Equisetum arvense voucher JAG288 (OAC) ribulose-1,5-bisphosphate carboxylase/oxygenase large subunit (rbcL) gene, partial cds; chloroplast	846	846	74%	0.0	97.58%	EU677106.1
Equisetum scirpoides isolate 41089 ribulose-1,5-bisphosphate carboxylase/oxygenase large subunit (rbcL) gene, partial cds; plastid	833	833	78%	0.0	95.59%	MH750203.1
Equisetum scirpoides isolate 26090 ribulose-1,5-bisphosphate carboxylase/oxygenase large subunit (rbcL) gene, partial cds; plastid	833	833	78%	0.0	95.59%	MH750202.1
Equisetum scirpoides isolate 10933 ribulose-1,5-bisphosphate carboxylase/oxygenase large subunit (rbcL) gene, partial cds; plastid	833	833	78%	0.0	95.59%	MH750201.1
Equisetum komarovii isolate 40830 ribulose-1,5-bisphosphate carboxylase/oxygenase large subunit (rbcL) gene, partial cds; plastid	833	833	78%	0.0	95.59%	MH750182.1
Equisetum scirpoides chloroplast rbcL gene for ribulose-1,5-bisphosphate carboxylase/oxygenase large subunit, partial cds, specimen_voucher: TNS:768168	833	833	78%	0.0	95.59%	AB574689.1
Equisetum scirpoides ribulose-1,5-bisphosphate carboxylase/oxygenase large subunit (rbcL) gene, partial cds; chloroplast gene for chloroplast product	833	833	78%	0.0	95.59%	AY226133.1
Equisetum x schaffneri isolate 40814 ribulose-1,5-bisphosphate carboxylase/oxygenase large subunit (rbcL) gene, partial cds; plastid	828	828	78%	0.0	95.40%	MH750225.1
Equisetum myriochaetum isolate 41080 ribulose-1,5-bisphosphate carboxylase/oxygenase large subunit (rbcL) gene, partial cds; plastid	828	828	78%	0.0	95.40%	MH750190.1

Description	Max Score	Total Score	Query Cover	E value	Per. Ident	Accession
Equisetum myriochaetum isolate 40936 ribulose-1,5-bisphosphate carboxylase/oxygenase large subunit (rbcL) gene, partial cds; plastid	828	828	78%	0.0	95.40%	MH750189.1
Equisetum myriochaetum isolate 40826 ribulose-1,5-bisphosphate carboxylase/oxygenase large subunit (rbcL) gene, partial cds; plastid	828	828	78%	0.0	95.40%	MH750188.1
Equisetum myriochaetum isolate 40825 ribulose-1,5-bisphosphate carboxylase/oxygenase large subunit (rbcL) gene, partial cds; plastid	828	828	78%	0.0	95.40%	MH750187.1
Equisetum myriochaetum isolate 40816 ribulose-1,5-bisphosphate carboxylase/oxygenase large subunit (rbcL) gene, partial cds; plastid	828	828	78%	0.0	95.40%	MH750186.1
Equisetum myriochaetum isolate 40815 ribulose-1,5-bisphosphate carboxylase/oxygenase large subunit (rbcL) gene, partial cds; plastid	828	828	78%	0.0	95.40%	MH750185.1
Equisetum myriochaetum isolate 26088 ribulose-1,5-bisphosphate carboxylase/oxygenase large subunit (rbcL) gene, partial cds; plastid	828	828	78%	0.0	95.40%	MH750184.1
Equisetum laevigatum isolate 40812 ribulose-1,5-bisphosphate carboxylase/oxygenase large subunit (rbcL) gene, partial cds; plastid	828	828	78%	0.0	95.40%	MH750183.1
Equisetum sp. Schuettpelz 1643 ribulose-1,5-bisphosphate carboxylase/oxygenase large subunit (rbcL) gene, partial cds; chloroplast	828	828	78%	0.0	95.40%	MW620286.1
Equisetum pratense voucher AP451 ribulose-1,5-bisphosphate carboxylase/oxygenase large subunit (rbcL) gene, partial cds; chloroplast	828	828	72%	0.0	97.53%	HQ590083.1
Equisetum x ferrissii ribulose-1,5-bisphosphate carboxylase/oxygenase large subunit (rbcL) gene, partial cds; chloroplast	828	828	78%	0.0	95.40%	EU352292.1
Equisetum arvense voucher AP400 (OAC) ribulose-1,5-bisphosphate carboxylase/oxygenase large subunit (rbcL) gene, partial cds; chloroplast	828	828	72%	0.0	97.53%	EU677105.1
Equisetum myriochaetum ribulose-1,5-bisphosphate carboxylase/oxygenase large subunit (rbcL) gene, partial cds; chloroplast gene for chloroplast product	828	828	78%	0.0	95.40%	AY226131.1
Equisetum laevigatum ribulose-1,5-bisphosphate carboxylase/oxygenase large subunit (rbcL) gene, partial cds; chloroplast gene for chloroplast product	828	828	78%	0.0	95.40%	AY226130.1
Equisetum x ferrissii ribulose-1,5-bisphosphate carboxylase large subunit (rbcL) gene, partial cds; chloroplast gene for chloroplast product	828	828	78%	0.0	95.40%	AF313579.1
Equisetum xylochaetum isolate 40614 ribulose-1,5-bisphosphate carboxylase/oxygenase large subunit (rbcL) gene, partial cds; plastid	822	822	78%	0.0	95.21%	MH750228.1

Description	Max Score	Total Score	Query Cover	E value	Per. Ident	Accession
Equisetum x schaffneri isolate 40824 ribulose-1,5-bisphosphate carboxylase/oxygenase large subunit (rbcL) gene, partial cds; plastid	822	822	78%	0.0	95.21%	MH750226.1
Equisetum x schaffneri isolate 40813 ribulose-1,5-bisphosphate carboxylase/oxygenase large subunit (rbcL) gene, partial cds; plastid	822	822	78%	0.0	95.21%	MH750224.1
Equisetum giganteum isolate 40811 ribulose-1,5-bisphosphate carboxylase/oxygenase large subunit (rbcL) gene, partial cds; plastid	822	822	78%	0.0	95.21%	MH750176.1
Equisetum giganteum isolate 40809 ribulose-1,5-bisphosphate carboxylase/oxygenase large subunit (rbcL) gene, partial cds; plastid	822	822	78%	0.0	95.21%	MH750175.1
Equisetum giganteum isolate 40807 ribulose-1,5-bisphosphate carboxylase/oxygenase large subunit (rbcL) gene, partial cds; plastid	822	822	78%	0.0	95.21%	MH750173.1
Equisetum giganteum isolate 40806 ribulose-1,5-bisphosphate carboxylase/oxygenase large subunit (rbcL) gene, partial cds; plastid	822	822	78%	0.0	95.21%	MH750172.1
Equisetum giganteum isolate 40805 ribulose-1,5-bisphosphate carboxylase/oxygenase large subunit (rbcL) gene, partial cds; plastid	822	822	78%	0.0	95.21%	MH750171.1
Equisetum giganteum isolate 26087 ribulose-1,5-bisphosphate carboxylase/oxygenase large subunit (rbcL) gene, partial cds; plastid	822	822	78%	0.0	95.21%	MH750170.1
Equisetum xylochaetum chloroplast, complete genome	822	822	78%	0.0	95.21%	MW282958.1
Equisetum giganteum ribulose-1,5-bisphosphate carboxylase/oxygenase large subunit (rbcL) gene, partial cds; chloroplast gene for chloroplast product	822	822	78%	0.0	95.21%	AY226127.1
Equisetum environmental sample IRbL.E3 ribulose-1,5-bisphosphate carboxylase/oxygenase large subunit (rbcL) gene, partial cds; plastid	821	821	69%	0.0	98.70%	JQ007379.1
Equisetum trachyodon isolate 41092 ribulose-1,5-bisphosphate carboxylase/oxygenase large subunit (rbcL) gene, partial cds; plastid	817	817	78%	0.0	95.02%	MH750227.1
Equisetum hyemale subsp. hyemale isolate 41088 ribulose-1,5-bisphosphate carboxylase/oxygenase large subunit (rbcL) gene, partial cds; plastid	817	817	78%	0.0	95.02%	MH750181.1
Equisetum hyemale subsp. hyemale isolate 23252 ribulose-1,5-bisphosphate carboxylase/oxygenase large subunit (rbcL) gene, partial cds; plastid	817	817	78%	0.0	95.02%	MH750180.1
Equisetum hyemale subsp. hyemale isolate 20201 ribulose-1,5-bisphosphate carboxylase/oxygenase large subunit (rbcL) gene, partial cds; plastid	817	817	78%	0.0	95.02%	MH750179.1

Description	Max Score	Total Score	Query Cover	E value	Per. Ident	Accession
Equisetum hyemale subsp. affine isolate 41501 ribulose-1,5-bisphosphate carboxylase/oxygenase large subunit (rbcL) gene, partial cds; plastid	817	817	78%	0.0	95.02%	MH750178.1
Equisetum hyemale subsp. affine isolate 40831 ribulose-1,5-bisphosphate carboxylase/oxygenase large subunit (rbcL) gene, partial cds; plastid	817	817	78%	0.0	95.02%	MH750177.1
Equisetum hyemale subsp. affine voucher OAC 94916 ribulose-1,5-bisphosphate carboxylase/oxygenase large subunit (rbcL) gene, partial cds; chloroplast	817	817	78%	0.0	95.02%	KF186500.1
Equisetum hyemale chloroplast, complete genome	817	817	78%	0.0	95.02%	KC117177.1
Equisetum hyemale chloroplast rbcL gene for ribulose-1,5-bisphosphate carboxylase/oxygenase large subunit, partial cds, specimen_voucher: TNS:765859	817	817	78%	0.0	95.02%	AB574685.1
Equisetum hyemale subsp. affine ribulose-1,5-bisphosphate carboxylase/oxygenase large subunit (rbcL) gene, partial cds; chloroplast gene for chloroplast product	817	817	78%	0.0	95.02%	AY226128.1
Equisetum giganteum isolate 40808 ribulose-1,5-bisphosphate carboxylase/oxygenase large subunit (rbcL) gene, partial cds; plastid	811	811	78%	0.0	94.83%	MH750174.1
Equisetum variegatum chloroplast rbcL gene for ribulose-1,5-bisphosphate carboxylase/oxygenase large subunit, partial cds, specimen_voucher: TNS:768164	811	811	78%	0.0	94.85%	AB574691.1
Equisetum ramosissimum subsp. debile isolate 40829 ribulose-1,5-bisphosphate carboxylase/oxygenase large subunit (rbcL) gene, partial cds; plastid	806	806	78%	0.0	94.64%	MH750200.1
Equisetum ramosissimum isolate BPTPS123 voucher LISE:96444 chloroplast, complete genome	806	806	78%	0.0	94.64%	ON641349.1
Equisetum ramosissimum isolate BPTPS056 voucher LISE:96334 chloroplast, complete genome	806	806	78%	0.0	94.64%	NC_062377.1
Equisetum ramosissimum isolate BPTPS056 voucher LISE:96334 chloroplast, complete genome	806	806	78%	0.0	94.64%	OM691674.1
Equisetum ramosissimum subsp. debile voucher G.M. Plunkett 3642 (NYBG) ribulose-1,5-bisphosphate carboxylase/oxygenase large subunit (rbcL) gene, partial cds; chloroplast	806	806	78%	0.0	94.64%	MT657901.1

Alignments

Equisetum palustre isolate 39349 ribulose-1,5-bisphosphate carboxylase/oxygenase large subunit (rbcL) gene, partial cds; plastid

Sequence ID: **MH750192.1** Length: 1298 Number of Matches: 1

Range 1: 97 to 615

Score	Expect	Identities	Gaps	Strand	Frame
900 bits(487)	0.0()	511/522(98%)	3/522(0%)	Plus/Plus	

Features:

```
Query  144   GAAGAAGAAGGAGCAGAGCTATTTGCTGAATCGTCCACGGGCACCTGGACTACCGTATGG  203
             ||||||| ||||||||   ||  |||||||||||||||||||||||||||||||||||||
Sbjct  97    GAAGAAGCAGGAGCAGCCGTAGCTGCTGAATCGTCCACGGGCACCTGGACTACCGTATGG  156

Query  204   ACAGATGGACTTACTAGTCTTGATCGATATAAAGGTCGATGCTATAATATTGAACCTGTT  263
             ||||||||||||||||||||||||||||||||||||||||||||||||||||||||||||
Sbjct  157   ACAGATGGACTTACTAGTCTTGATCGATATAAAGGTCGATGCTATAATATTGAACCTGTT  216

Query  264   GCTGGAGAAGATAATCAATTCATAGCTTATGTAGCCTATCCTTTAGATCTTTTTGAAGAA  323
             ||||||||||||||||||||||||||||||||||||||||||||||||||||||||||||
Sbjct  217   GCTGGAGAAGATAATCAATTCATAGCTTATGTAGCCTATCCTTTAGATCTTTTTGAAGAA  276

Query  324   GGTTCTGTTACCAATCTTTTTACTTCAATTGTTGGTAATGTTTTCGGCTTCAAAGCTCTA  383
             ||||||||||||||||||||||||||||||||||||||||||||||||||||||||||||
Sbjct  277   GGTTCTGTTACCAATCTTTTTACTTCAATTGTTGGTAATGTTTTCGGCTTCAAAGCTCTA  336

Query  384   CGTGCTTTACGTTTAGAAGATTTAAGAATTCCTCCTGCTTACTCCAAAACTTTTATAGGA  443
             ||||||||||||||||||||||||||||||||||||||||||||||||||||||||||||
Sbjct  337   CGTGCTTTACGTTTAGAAGATTTAAGAATTCCTCCTGCTTACTCCAAAACTTTTATAGGA  396

Query  444   CCACCCCACGGTATCCAGGTTGAAAGAGATAAGTTAAACAAATATGGTCGTCCTTTATTA  503
             ||||||||||||||||||||||||||||||||||||||||||||||||||||||||||||
Sbjct  397   CCACCCCACGGTATCCAGGTTGAAAGAGATAAGTTAAACAAATATGGTCGTCCTTTATTA  456

Query  504   GGTTGTACAATTAAACCAAAAATTGGGACTATCTGCTAAAAACTATGGTAGGGGCTGTTT  563
             ||||||||||||||||| |||||||||||||||||||||||||||||||| |||||||||
Sbjct  457   GGTTGTACAATTAAACC-AAAATTGGGACTATCTGCTAAAAACTATGGTA-GGGCTGTTT  514

Query  564   ATGAATGTCTTCGTGGTGGACTTGATTTCACCAAAGATGATGAGAATGTAAACTCTCAAC  623
             ||||||||||||||||||||||||||||||||||||||||||||||||||||||||||||
Sbjct  515   ATGAATGTCTTCGTGGTGGACTTGATTTCACCAAAGATGATGAGAATGTAAACTCTCAAC  574

Query  624   CCTTTATGCGTTGGAGAGATCGATTCTTATTTTGTAGCAGAA  665
             |||||||||||||||||||||| ||||||||  |||||||||
Sbjct  575   CCTTTATGCGTTGGAGAGATCGTTTCTTATTC-GTAGCAGAA  615
```

Equisetum palustre isolate 17671 ribulose-1,5-bisphosphate carboxylase/oxygenase large subunit (rbcL) gene, partial cds; plastid
Sequence ID: **MH750191.1** Length: 1292 Number of Matches: 1
Range 1: 98 to 616

Score	Expect	Identities	Gaps	Strand	Frame
900 bits(487)	0.0()	511/522(98%)	3/522(0%)	Plus/Plus	

Features:

```
Query  144   GAAGAAGAAGGAGCAGAGCTATTTGCTGAATCGTCCACGGGCACCTGGACTACCGTATGG  203
             ||||||| ||||||||   ||  |||||||||||||||||||||||||||||||||||||
Sbjct  98    GAAGAAGCAGGAGCAGCCGTAGCTGCTGAATCGTCCACGGGCACCTGGACTACCGTATGG  157

Query  204   ACAGATGGACTTACTAGTCTTGATCGATATAAAGGTCGATGCTATAATATTGAACCTGTT  263
             ||||||||||||||||||||||||||||||||||||||||||||||||||||||||||||
Sbjct  158   ACAGATGGACTTACTAGTCTTGATCGATATAAAGGTCGATGCTATAATATTGAACCTGTT  217

Query  264   GCTGGAGAAGATAATCAATTCATAGCTTATGTAGCCTATCCTTTAGATCTTTTTGAAGAA  323
             ||||||||||||||||||||||||||||||||||||||||||||||||||||||||||||
Sbjct  218   GCTGGAGAAGATAATCAATTCATAGCTTATGTAGCCTATCCTTTAGATCTTTTTGAAGAA  277

Query  324   GGTTCTGTTACCAATCTTTTTACTTCAATTGTTGGTAATGTTTTCGGCTTCAAAGCTCTA  383
             ||||||||||||||||||||||||||||||||||||||||||||||||||||||||||||
Sbjct  278   GGTTCTGTTACCAATCTTTTTACTTCAATTGTTGGTAATGTTTTCGGCTTCAAAGCTCTA  337

Query  384   CGTGCTTTACGTTTAGAAGATTTAAGAATTCCTCCTGCTTACTCCAAAACTTTTATAGGA  443
             ||||||||||||||||||||||||||||||||||||||||||||||||||||||||||||
Sbjct  338   CGTGCTTTACGTTTAGAAGATTTAAGAATTCCTCCTGCTTACTCCAAAACTTTTATAGGA  397

Query  444   CCACCCCACGGTATCCAGGTTGAAAGAGATAAGTTAAACAAATATGGTCGTCCTTTATTA  503
             ||||||||||||||||||||||||||||||||||||||||||||||||||||||||||||
Sbjct  398   CCACCCCACGGTATCCAGGTTGAAAGAGATAAGTTAAACAAATATGGTCGTCCTTTATTA  457

Query  504   GGTTGTACAATTAAACCAAAAATTGGGACTATCTGCTAAAAACTATGGTAGGGGCTGTTT  563
             ||||||||||||||||| |||||||||||||||||||||||||||||||| |||||||||
Sbjct  458   GGTTGTACAATTAAACC-AAAATTGGGACTATCTGCTAAAAACTATGGTA-GGGCTGTTT  515

Query  564   ATGAATGTCTTCGTGGTGGACTTGATTTCACCAAAGATGATGAGAATGTAAACTCTCAAC  623
             ||||||||||||||||||||||||||||||||||||||||||||||||||||||||||||
Sbjct  516   ATGAATGTCTTCGTGGTGGACTTGATTTCACCAAAGATGATGAGAATGTAAACTCTCAAC  575

Query  624   CCTTTATGCGTTGGAGAGATCGATTCTTATTTTGTAGCAGAA  665
             |||||||||||||||||||||| ||||||||  |||||||||
Sbjct  576   CCTTTATGCGTTGGAGAGATCGTTTCTTATTC-GTAGCAGAA  616
```

Equisetum palustre chloroplast, partial genome

Sequence ID: **MT984458.1** Length: 5501 Number of Matches: 1

Range 1: 2363 to 2881

Score	Expect	Identities	Gaps	Strand	Frame
900 bits(487)	0.0()	511/522(98%)	3/522(0%)	Plus/Plus	

Features:

```
Query  144    GAAGAAGAAGGAGCAGAGCTATTTGCTGAATCGTCCACGGGCACCTGGACTACCGTATGG  203
              ||||||| ||||||||   ||  |||||||||||||||||||||||||||||||||||||
Sbjct  2363   GAAGAAGCAGGAGCAGCCGTAGCTGCTGAATCGTCCACGGGCACCTGGACTACCGTATGG  2422

Query  204    ACAGATGGACTTACTAGTCTTGATCGATATAAAGGTCGATGCTATAATATTGAACCTGTT  263
              ||||||||||||||||||||||||||||||||||||||||||||||||||||||||||||
Sbjct  2423   ACAGATGGACTTACTAGTCTTGATCGATATAAAGGTCGATGCTATAATATTGAACCTGTT  2482

Query  264    GCTGGAGAAGATAATCAATTCATAGCTTATGTAGCCTATCCTTTAGATCTTTTTGAAGAA  323
              ||||||||||||||||||||||||||||||||||||||||||||||||||||||||||||
Sbjct  2483   GCTGGAGAAGATAATCAATTCATAGCTTATGTAGCCTATCCTTTAGATCTTTTTGAAGAA  2542

Query  324    GGTTCTGTTACCAATCTTTTTACTTCAATTGTTGGTAATGTTTTCGGCTTCAAAGCTCTA  383
              ||||||||||||||||||||||||||||||||||||||||||||||||||||||||||||
Sbjct  2543   GGTTCTGTTACCAATCTTTTTACTTCAATTGTTGGTAATGTTTTCGGCTTCAAAGCTCTA  2602

Query  384    CGTGCTTTACGTTTAGAAGATTTAAGAATTCCTCCTGCTTACTCCAAAACTTTTATAGGA  443
              ||||||||||||||||||||||||||||||||||||||||||||||||||||||||||||
Sbjct  2603   CGTGCTTTACGTTTAGAAGATTTAAGAATTCCTCCTGCTTACTCCAAAACTTTTATAGGA  2662

Query  444    CCACCCCACGGTATCCAGGTTGAAAGAGATAAGTTAAACAAATATGGTCGTCCTTTATTA  503
              ||||||||||||||||||||||||||||||||||||||||||||||||||||||||||||
Sbjct  2663   CCACCCCACGGTATCCAGGTTGAAAGAGATAAGTTAAACAAATATGGTCGTCCTTTATTA  2722

Query  504    GGTTGTACAATTAAACCAAAAATTGGGACTATCTGCTAAAAACTATGGTAGGGGCTGTTT  563
              ||||||||||||||||| |||||||||||||||||||||||||||||||| |||||||||
Sbjct  2723   GGTTGTACAATTAAACC-AAAATTGGGACTATCTGCTAAAAACTATGGTA-GGGCTGTTT  2780

Query  564    ATGAATGTCTTCGTGGTGGACTTGATTTCACCAAAGATGATGAGAATGTAAACTCTCAAC  623
              ||||||||||||||||||||||||||||||||||||||||||||||||||||||||||||
Sbjct  2781   ATGAATGTCTTCGTGGTGGACTTGATTTCACCAAAGATGATGAGAATGTAAACTCTCAAC  2840

Query  624    CCTTTATGCGTTGGAGAGATCGATTCTTATTTTGTAGCAGAA  665
              |||||||||||||||||||||| ||||||||  |||||||||
Sbjct  2841   CCTTTATGCGTTGGAGAGATCGTTTCTTATTC-GTAGCAGAA  2881
```

Equisetum palustre chloroplast rbcL gene for ribulose-1,5-bisphosphate carboxylase/oxygenase large subunit, partial cds, specimen_voucher: TNS:776981

Sequence ID: **AB574686.1** Length: 1205 Number of Matches: 1

Range 1: 79 to 597

Score	Expect	Identities	Gaps	Strand	Frame
900 bits(487)	0.0()	511/522(98%)	3/522(0%)	Plus/Plus	

Features:

```
Query  144  GAAGAAGAAGGAGCAGAGCTATTTGCTGAATCGTCCACGGGCACCTGGACTACCGTATGG  203
            ||||||| ||||||||   ||  |||||||||||||||||||||||||||||||||||||
Sbjct  79   GAAGAAGCAGGAGCAGCCGTAGCTGCTGAATCGTCCACGGGCACCTGGACTACCGTATGG  138

Query  204  ACAGATGGACTTACTAGTCTTGATCGATATAAAGGTCGATGCTATAATATTGAACCTGTT  263
            ||||||||||||||||||||||||||||||||||||||||||||||||||||||||||||
Sbjct  139  ACAGATGGACTTACTAGTCTTGATCGATATAAAGGTCGATGCTATAATATTGAACCTGTT  198

Query  264  GCTGGAGAAGATAATCAATTCATAGCTTATGTAGCCTATCCTTTAGATCTTTTTGAAGAA  323
            ||||||||||||||||||||||||||||||||||||||||||||||||||||||||||||
Sbjct  199  GCTGGAGAAGATAATCAATTCATAGCTTATGTAGCCTATCCTTTAGATCTTTTTGAAGAA  258

Query  324  GGTTCTGTTACCAATCTTTTTACTTCAATTGTTGGTAATGTTTTCGGCTTCAAAGCTCTA  383
            ||||||||||||||||||||||||||||||||||||||||||||||||||||||||||||
Sbjct  259  GGTTCTGTTACCAATCTTTTTACTTCAATTGTTGGTAATGTTTTCGGCTTCAAAGCTCTA  318

Query  384  CGTGCTTTACGTTTAGAAGATTTAAGAATTCCTCCTGCTTACTCCAAAACTTTTATAGGA  443
            ||||||||||||||||||||||||||||||||||||||||||||||||||||||||||||
Sbjct  319  CGTGCTTTACGTTTAGAAGATTTAAGAATTCCTCCTGCTTACTCCAAAACTTTTATAGGA  378

Query  444  CCACCCCACGGTATCCAGGTTGAAAGAGATAAGTTAAACAAATATGGTCGTCCTTTATTA  503
            ||||||||||||||||||||||||||||||||||||||||||||||||||||||||||||
Sbjct  379  CCACCCCACGGTATCCAGGTTGAAAGAGATAAGTTAAACAAATATGGTCGTCCTTTATTA  438
```

```
Query  504   GGTTGTACAATTAAACCAAAAATTGGGACTATCTGCTAAAAACTATGGTAGGGGCTGTTT  563
             ||||||||||||||||| |||||||||||||||||||||||||||||||| |||||||||
Sbjct  439   GGTTGTACAATTAAACC-AAAATTGGGACTATCTGCTAAAAACTATGGTA-GGGCTGTTT  496

Query  564   ATGAATGTCTTCGTGGTGGACTTGATTTCACCAAAGATGATGAGAATGTAAACTCTCAAC  623
             ||||||||||||||||||||||||||||||||||||||||||||||||||||||||||||
Sbjct  497   ATGAATGTCTTCGTGGTGGACTTGATTTCACCAAAGATGATGAGAATGTAAACTCTCAAC  556

Query  624   CCTTTATGCGTTGGAGAGATCGATTCTTATTTTGTAGCAGAA  665
             |||||||||||||||||||||| ||||||||  |||||||||
Sbjct  557   CCTTTATGCGTTGGAGAGATCGTTTCTTATTC-GTAGCAGAA  597
```

Equisetum palustre ribulose-1,5-bisphosphate carboxylase/oxygenase large subunit (rbcL) gene, partial cds; chloroplast gene for chloroplast product

Sequence ID: **AY226138.1** Length: 1325 Number of Matches: 1

Range 1: 125 to 643

Score	Expect	Identities	Gaps	Strand	Frame
900 bits(487)	0.0()	511/522(98%)	3/522(0%)	Plus/Plus	

Features:

```
Query  144   GAAGAAGAAGGAGCAGAGCTATTTGCTGAATCGTCCACGGGCACCTGGACTACCGTATGG  203
             ||||||| ||||||||   ||  |||||||||||||||||||||||||||||||||||||
Sbjct  125   GAAGAAGCAGGAGCAGCCGTAGCTGCTGAATCGTCCACGGGCACCTGGACTACCGTATGG  184

Query  204   ACAGATGGACTTACTAGTCTTGATCGATATAAAGGTCGATGCTATAATATTGAACCTGTT  263
             ||||||||||||||||||||||||||||||||||||||||||||||||||||||||||||
Sbjct  185   ACAGATGGACTTACTAGTCTTGATCGATATAAAGGTCGATGCTATAATATTGAACCTGTT  244

Query  264   GCTGGAGAAGATAATCAATTCATAGCTTATGTAGCCTATCCTTTAGATCTTTTTGAAGAA  323
             ||||||||||||||||||||||||||||||||||||||||||||||||||||||||||||
Sbjct  245   GCTGGAGAAGATAATCAATTCATAGCTTATGTAGCCTATCCTTTAGATCTTTTTGAAGAA  304

Query  324   GGTTCTGTTACCAATCTTTTTACTTCAATTGTTGGTAATGTTTTCGGCTTCAAAGCTCTA  383
             ||||||||||||||||||||||||||||||||||||||||||||||||||||||||||||
Sbjct  305   GGTTCTGTTACCAATCTTTTTACTTCAATTGTTGGTAATGTTTTCGGCTTCAAAGCTCTA  364

Query  384   CGTGCTTTACGTTTAGAAGATTTAAGAATTCCTCCTGCTTACTCCAAAACTTTTATAGGA  443
             ||||||||||||||||||||||||||||||||||||||||||||||||||||||||||||
Sbjct  365   CGTGCTTTACGTTTAGAAGATTTAAGAATTCCTCCTGCTTACTCCAAAACTTTTATAGGA  424

Query  444   CCACCCCACGGTATCCAGGTTGAAAGAGATAAGTTAAACAAATATGGTCGTCCTTTATTA  503
             ||||||||||||||||||||||||||||||||||||||||||||||||||||||||||||
Sbjct  425   CCACCCCACGGTATCCAGGTTGAAAGAGATAAGTTAAACAAATATGGTCGTCCTTTATTA  484

Query  504   GGTTGTACAATTAAACCAAAAATTGGGACTATCTGCTAAAAACTATGGTAGGGGCTGTTT  563
             ||||||||||||||||| |||||||||||||||||||||||||||||||| |||||||||
Sbjct  485   GGTTGTACAATTAAACC-AAAATTGGGACTATCTGCTAAAAACTATGGTA-GGGCTGTTT  542

Query  564   ATGAATGTCTTCGTGGTGGACTTGATTTCACCAAAGATGATGAGAATGTAAACTCTCAAC  623
             ||||||||||||||||||||||||||||||||||||||||||||||||||||||||||||
Sbjct  543   ATGAATGTCTTCGTGGTGGACTTGATTTCACCAAAGATGATGAGAATGTAAACTCTCAAC  602

Query  624   CCTTTATGCGTTGGAGAGATCGATTCTTATTTTGTAGCAGAA  665
             |||||||||||||||||||||| ||||||||  |||||||||
Sbjct  603   CCTTTATGCGTTGGAGAGATCGTTTCTTATTC-GTAGCAGAA  643
```


BLAST is a registered trademark of the National Library of Medicine

Support center Mailing list YouTube

- National Library Of Medicine
- National Institutes Of Health
- U.S. Department of Health & Human Services

- USA.gov

NCBI

National Center for Biotechnology Information, U.S. National Library of Medicine *8600 Rockville Pike, Bethesda MD, 20894 USA*
Policies and Guidelines | Contact
HHS Vulnerability Disclosure
