# Supplementary material for: A Multi-Pronged Technique for Identifying Equisetum palustre and Equisetum arvense—Combining HPTLC, HPLC-ESI-MS/MS and Optimized DNA Barcoding Techniques
Source: Plants (Basel). 2022 Sep 28;11(19):2562. doi: 10.3390/plants11192562 (PMC9573248; doi:10.3390/plants11192562)
Supplement: Supplementary file 1 [file plants-11-02562-s001.zip › plants-1932149 supplementary/Horsetail Supplementary Figures/Figure S25 (P8, 82714220323-1).pdf]

BLAST ® » blastn suite » RID-J1CF9BU4013

BLAST Results

Questions/comments

Job title: Nucleotide Sequence

**RID**	J1CF9BU4013 (Expires on 09-15 00:38 am)
**Query ID**	lcl|Query_57325
**Description**	None
**Molecule type**	dna
**Query Length**	670
**Database Name**	nt
**Description**	Nucleotide collection (nt)
**Program**	BLASTN 2.13.0+

Graphic Summary

Distribution of the top 100 Blast Hits on 100 subject sequences

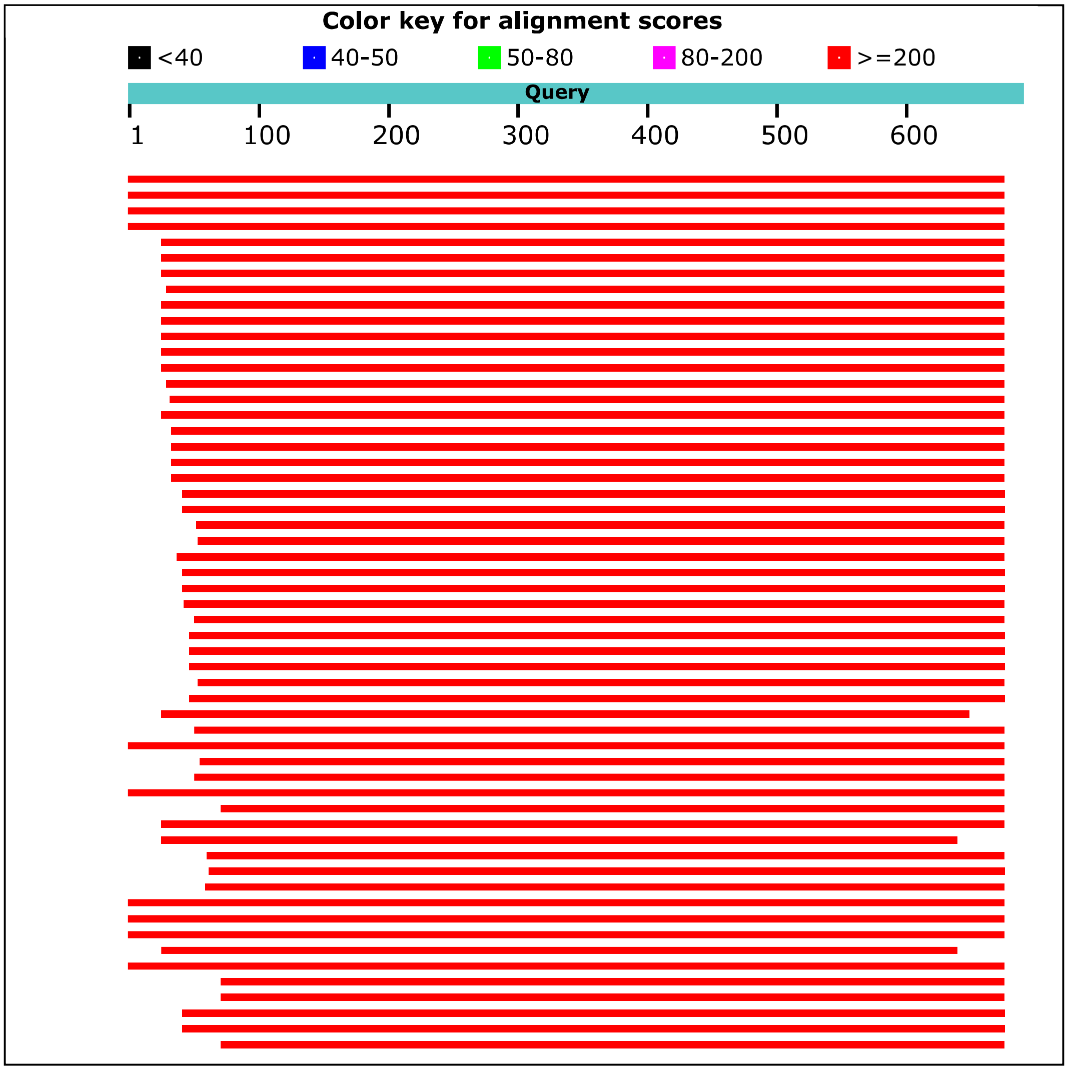

Descriptions

Sequences producing significant alignments:

Description	Max Score	Total Score	Query Cover	E value	Per. Ident	Accession
Equisetum palustre chloroplast, partial genome	1208	1208	99%	0.0	99.25%	MT984458.1
Equisetum arvense chloroplast, complete genome	1186	1186	99%	0.0	98.66%	GU191334.1
Equisetum arvense chloroplast ribulosebisphosphate carboxylase (rbcL) gene, complete cds	1186	1186	99%	0.0	98.66%	L11053.1
Equisetum arvense plastid, complete genome	1181	1181	99%	0.0	98.51%	JN968380.1
Equisetum palustre ribulose-1,5-bisphosphate carboxylase/oxygenase large subunit (rbcL) gene, partial cds; chloroplast gene for chloroplast product	1171	1171	95%	0.0	99.53%	AY226138.1
Equisetum pratense ribulose-1,5-bisphosphate carboxylase/oxygenase large subunit (rbcL) gene, partial cds; chloroplast gene for chloroplast product	1158	1158	95%	0.0	99.22%	AY226137.1
Equisetum sylvaticum ribulose-1,5-bisphosphate carboxylase/oxygenase large subunit (rbcL) gene, partial cds; chloroplast gene for chloroplast product	1158	1158	95%	0.0	99.22%	AY226136.1
Equisetum sylvaticum isolate 11640 ribulose-1,5-bisphosphate carboxylase/oxygenase large subunit (rbcL) gene, partial cds; plastid	1151	1151	95%	0.0	99.22%	MH750204.1
Equisetum environmental sample isolate EDNA16-0043490 ribulose-1,5-bisphosphate carboxylase/oxygenase large subunit (rbcL) gene, partial cds; chloroplast	1149	1149	95%	0.0	98.91%	MT197555.1
Equisetum fluviatile ribulose-1,5-bisphosphate carboxylase/oxygenase large subunit (rbcL) gene, partial cds; chloroplast gene for chloroplast product	1149	1149	95%	0.0	98.91%	AY226142.1
Equisetum arvense ribulose-1,5-bisphosphate carboxylase/oxygenase large subunit (rbcL) gene, partial cds; chloroplast gene for chloroplast product	1149	1149	95%	0.0	98.91%	AY226140.1
Equisetum telmateia subsp. braunii ribulose-1,5-bisphosphate carboxylase/oxygenase large subunit (rbcL) gene, partial cds; chloroplast gene for chloroplast product	1149	1149	95%	0.0	98.91%	AY226135.1
Equisetum environmental sample isolate EDNA16-0043491b ribulose-1,5-bisphosphate carboxylase/oxygenase large subunit (rbcL) gene, partial cds; chloroplast	1144	1144	95%	0.0	98.76%	MT197548.1
Equisetum telmateia subsp. telmateia isolate 11642 ribulose-1,5-bisphosphate carboxylase/oxygenase large subunit (rbcL) gene, partial cds; plastid	1142	1142	95%	0.0	98.91%	MH750210.1

Description	Max Score	Total Score	Query Cover	E value	Per. Ident	Accession
Equisetum arvense subsp. boreale isolate 41074 ribulose-1,5-bisphosphate carboxylase/oxygenase large subunit (rbcL) gene, partial cds; plastid	1138	1138	95%	0.0	98.90%	MH750160.1
Equisetum diffusum ribulose-1,5-bisphosphate carboxylase/oxygenase large subunit (rbcL) gene, partial cds; chloroplast gene for chloroplast product	1138	1138	95%	0.0	98.60%	AY226141.1
Equisetum x dycei isolate 26083 ribulose-1,5-bisphosphate carboxylase/oxygenase large subunit (rbcL) gene, partial cds; plastid	1136	1136	94%	0.0	98.90%	MH750220.1
Equisetum telmateia ribulose-1,5-bisphosphate carboxylase large subunit (rbcL) gene, partial cds; chloroplast gene for chloroplast product	1136	1136	94%	0.0	98.90%	AF313580.1
Equisetum fluviatile isolate 41076 ribulose-1,5-bisphosphate carboxylase/oxygenase large subunit (rbcL) gene, partial cds; plastid	1134	1134	94%	0.0	98.90%	MH750169.1
Equisetum fluviatile isolate 41075 ribulose-1,5-bisphosphate carboxylase/oxygenase large subunit (rbcL) gene, partial cds; plastid	1134	1134	94%	0.0	98.90%	MH750168.1
Equisetum sylvaticum isolate 41081 ribulose-1,5-bisphosphate carboxylase/oxygenase large subunit (rbcL) gene, partial cds; plastid	1131	1131	93%	0.0	99.20%	MH750205.1
Equisetum pratense isolate 41087 ribulose-1,5-bisphosphate carboxylase/oxygenase large subunit (rbcL) gene, partial cds; plastid	1131	1131	93%	0.0	99.20%	MH750195.1
Equisetum palustre isolate 17671 ribulose-1,5-bisphosphate carboxylase/oxygenase large subunit (rbcL) gene, partial cds; plastid	1129	1129	92%	0.0	99.68%	MH750191.1
Equisetum palustre isolate 39349 ribulose-1,5-bisphosphate carboxylase/oxygenase large subunit (rbcL) gene, partial cds; plastid	1127	1127	91%	0.0	99.68%	MH750192.1
Equisetum arvense subsp. boreale isolate 41073 ribulose-1,5-bisphosphate carboxylase/oxygenase large subunit (rbcL) gene, partial cds; plastid	1127	1127	94%	0.0	98.89%	MH750159.1
Equisetum telmateia subsp. braunii isolate 40828 ribulose-1,5-bisphosphate carboxylase/oxygenase large subunit (rbcL) gene, partial cds; plastid	1122	1122	93%	0.0	98.89%	MH750207.1
Equisetum arvense subsp. arvense isolate 41071 ribulose-1,5-bisphosphate carboxylase/oxygenase large subunit (rbcL) gene, partial cds; plastid	1120	1120	93%	0.0	98.89%	MH750158.1
Equisetum x fontqueri isolate 26093 ribulose-1,5-bisphosphate carboxylase/oxygenase large subunit (rbcL) gene, partial cds; plastid	1118	1118	93%	0.0	98.88%	MH750221.1
Equisetum pratense isolate 41086 ribulose-1,5-bisphosphate carboxylase/oxygenase large subunit (rbcL) gene, partial cds; plastid	1118	1118	92%	0.0	99.35%	MH750194.1

Description	Max Score	Total Score	Query Cover	E value	Per. Ident	Accession
Equisetum x litorale isolate 41084 ribulose-1,5-bisphosphate carboxylase/oxygenase large subunit (rbcL) gene, partial cds; plastid	1116	1116	92%	0.0	99.04%	MH750222.1
Equisetum telmateia subsp. braunii isolate 40832 ribulose-1,5-bisphosphate carboxylase/oxygenase large subunit (rbcL) gene, partial cds; plastid	1116	1116	92%	0.0	99.04%	MH750208.1
Equisetum arvense subsp. arvense isolate 26085 ribulose-1,5-bisphosphate carboxylase/oxygenase large subunit (rbcL) gene, partial cds; plastid	1116	1116	92%	0.0	99.04%	MH750156.1
Equisetum pratense isolate 39348 ribulose-1,5-bisphosphate carboxylase/oxygenase large subunit (rbcL) gene, partial cds; plastid	1114	1114	91%	0.0	99.35%	MH750193.1
Equisetum diffusum isolate 40689 ribulose-1,5-bisphosphate carboxylase/oxygenase large subunit (rbcL) gene, partial cds; plastid	1110	1110	92%	0.0	98.87%	MH750166.1
Equisetum arvense voucher JAG288 (OAC) ribulose-1,5-bisphosphate carboxylase/oxygenase large subunit (rbcL) gene, partial cds; chloroplast	1110	1110	91%	0.0	99.19%	EU677106.1
Equisetum arvense subsp. arvense isolate 26084 ribulose-1,5-bisphosphate carboxylase/oxygenase large subunit (rbcL) gene, partial cds; plastid	1109	1109	92%	0.0	99.03%	MH750155.1
Equisetum xylochaetum chloroplast, complete genome	1103	1103	99%	0.0	96.42%	MW282958.1
Equisetum arvense subsp. arvense isolate 40833 ribulose-1,5-bisphosphate carboxylase/oxygenase large subunit (rbcL) gene, partial cds; plastid	1101	1101	91%	0.0	99.02%	MH750157.1
Equisetum diffusum isolate 40804 ribulose-1,5-bisphosphate carboxylase/oxygenase large subunit (rbcL) gene, partial cds; plastid	1098	1098	92%	0.0	98.71%	MH750167.1
Equisetum hyemale chloroplast, complete genome	1098	1098	99%	0.0	96.27%	KC117177.1
Equisetum palustre chloroplast rbcL gene for ribulose-1,5-bisphosphate carboxylase/oxygenase large subunit, partial cds, specimen_voucher: TNS:776981	1094	1094	89%	0.0	99.67%	AB574686.1
Equisetum scirpoides ribulose-1,5-bisphosphate carboxylase/oxygenase large subunit (rbcL) gene, partial cds; chloroplast gene for chloroplast product	1094	1094	95%	0.0	97.36%	AY226133.1
Equisetum arvense voucher AP400 (OAC) ribulose-1,5-bisphosphate carboxylase/oxygenase large subunit (rbcL) gene, partial cds; chloroplast	1092	1092	90%	0.0	99.18%	EU677105.1
Equisetum fluviatile ribulose-1,5-bisphosphate carboxylase/oxygenase large subunit (rbcL) gene, partial cds; chloroplast	1092	1092	90%	0.0	99.01%	DQ463101.1

Description	Max Score	Total Score	Query Cover	E value	Per. Ident	Accession
Equisetum arvense x Equisetum telmateia subsp. braunii isolate 40834 ribulose-1,5-bisphosphate carboxylase/oxygenase large subunit (rbcL) gene, partial cds; plastid	1090	1090	90%	0.0	99.01%	MH750161.1
Equisetum arvense ribulose-1,5-bisphosphate carboxylase/oxygenase large subunit (rbcL) gene, partial cds; chloroplast	1088	1088	91%	0.0	98.85%	KM218345.1
Equisetum ramosissimum isolate BPTPS123 voucher LISE:96444 chloroplast, complete genome	1086	1086	99%	0.0	95.97%	ON641349.1
Equisetum ramosissimum isolate BPTPS056 voucher LISE:96334 chloroplast, complete genome	1086	1086	99%	0.0	95.97%	NC_062377.1
Equisetum ramosissimum isolate BPTPS056 voucher LISE:96334 chloroplast, complete genome	1086	1086	99%	0.0	95.97%	OM691674.1
Equisetum pratense voucher AP451 ribulose-1,5-bisphosphate carboxylase/oxygenase large subunit (rbcL) gene, partial cds; chloroplast	1085	1085	90%	0.0	98.85%	HQ590083.1
Equisetum hyemale ribulose-1,5-bisphosphate carboxylase/oxygenase large subunit (rbcL) gene, partial cds; chloroplast	1083	1083	99%	0.0	95.82%	DQ646001.1
Equisetum sylvaticum chloroplast rbcL gene for ribulose-1,5-bisphosphate carboxylase/oxygenase large subunit, partial cds, specimen_voucher: TNS:776974	1081	1081	89%	0.0	99.33%	AB574690.1
Equisetum pratense chloroplast rbcL gene for ribulose-1,5-bisphosphate carboxylase/oxygenase large subunit, partial cds, specimen_voucher: TNS:1107843	1081	1081	89%	0.0	99.33%	AB574687.1
Equisetum scirpoides isolate 41089 ribulose-1,5-bisphosphate carboxylase/oxygenase large subunit (rbcL) gene, partial cds; plastid	1072	1072	93%	0.0	97.46%	MH750203.1
Equisetum komarovii isolate 40830 ribulose-1,5-bisphosphate carboxylase/oxygenase large subunit (rbcL) gene, partial cds; plastid	1072	1072	93%	0.0	97.46%	MH750182.1
Equisetum fluviatile chloroplast rbcL gene for ribulose-1,5-bisphosphate carboxylase/oxygenase large subunit, partial cds, specimen_voucher: TNS:776384	1072	1072	89%	0.0	99.00%	AB574684.1
Equisetum bogotense ribulose-1,5-bisphosphate carboxylase/oxygenase large subunit (rbcL) gene, partial cds; chloroplast gene for chloroplast product	1068	1068	95%	0.0	96.73%	AY226139.1
Equisetum scirpoides isolate 26090 ribulose-1,5-bisphosphate carboxylase/oxygenase large subunit (rbcL) gene, partial cds; plastid	1066	1066	93%	0.0	97.30%	MH750202.1

Description	Max Score	Total Score	Query Cover	E value	Per. Ident	Accession
Equisetum arvense chloroplast rbcL gene for ribulose-1,5-bisphosphate carboxylase/oxygenase large subunit, partial cds, specimen_voucher: TNS:763618	1066	1066	89%	0.0	98.83%	AB574683.1
Equisetum myriochaetum ribulose-1,5-bisphosphate carboxylase/oxygenase large subunit (rbcL) gene, partial cds; chloroplast gene for chloroplast product	1066	1066	95%	0.0	96.58%	AY226131.1
Equisetum giganteum ribulose-1,5-bisphosphate carboxylase/oxygenase large subunit (rbcL) gene, partial cds; chloroplast gene for chloroplast product	1066	1066	95%	0.0	96.58%	AY226127.1
Equisetum sp. Schuettpelz 1643 ribulose-1,5-bisphosphate carboxylase/oxygenase large subunit (rbcL) gene, partial cds; chloroplast	1064	1064	95%	0.0	96.58%	MW620286.1
Equisetum laevigatum ribulose-1,5-bisphosphate carboxylase/oxygenase large subunit (rbcL) gene, partial cds; chloroplast gene for chloroplast product	1062	1062	95%	0.0	96.57%	AY226130.1
Equisetum hyemale subsp. affine voucher OAC 94916 ribulose-1,5-bisphosphate carboxylase/oxygenase large subunit (rbcL) gene, partial cds; chloroplast	1061	1061	95%	0.0	96.43%	KF186500.1
Equisetum hyemale subsp. affine ribulose-1,5-bisphosphate carboxylase/oxygenase large subunit (rbcL) gene, partial cds; chloroplast gene for chloroplast product	1061	1061	95%	0.0	96.43%	AY226128.1
Equisetum scirpoides isolate 10933 ribulose-1,5-bisphosphate carboxylase/oxygenase large subunit (rbcL) gene, partial cds; plastid	1059	1059	92%	0.0	97.57%	MH750201.1
Equisetum giganteum isolate 40806 ribulose-1,5-bisphosphate carboxylase/oxygenase large subunit (rbcL) gene, partial cds; plastid	1059	1059	95%	0.0	96.56%	MH750172.1
Equisetum giganteum isolate 26087 ribulose-1,5-bisphosphate carboxylase/oxygenase large subunit (rbcL) gene, partial cds; plastid	1059	1059	95%	0.0	96.56%	MH750170.1
Equisetum ramosissimum voucher R825 ribulose-1,5-bisphosphate carboxylase/oxygenase large subunit (rbcl) gene, partial cds; chloroplast	1059	1059	96%	0.0	96.15%	KY656709.1
Equisetum x ferrissii ribulose-1,5-bisphosphate carboxylase/oxygenase large subunit (rbcL) gene, partial cds; chloroplast	1059	1059	95%	0.0	96.56%	EU352292.1
Equisetum x ferrissii ribulose-1,5-bisphosphate carboxylase large subunit (rbcL) gene, partial cds; chloroplast gene for chloroplast product	1057	1057	95%	0.0	96.56%	AF313579.1
Equisetum ramosissimum subsp. debile voucher G.M. Plunkett 3642 (NYBG) ribulose-1,5-bisphosphate carboxylase/oxygenase large subunit (rbcL) gene, partial cds; chloroplast	1050	1050	95%	0.0	96.12%	MT657901.1

Description	Max Score	Total Score	Query Cover	E value	Per. Ident	Accession
Equisetum ramosissimum subsp. debile ribulose-1,5-bisphosphate carboxylase/oxygenase large subunit (rbcL) gene, partial cds; chloroplast gene for chloroplast product	1050	1050	95%	0.0	96.12%	AY226132.1
Equisetum myriochaetum isolate 40815 ribulose-1,5-bisphosphate carboxylase/oxygenase large subunit (rbcL) gene, partial cds; plastid	1048	1048	94%	0.0	96.39%	MH750185.1
Equisetum scirpoides voucher AP248 ribulose-1,5-bisphosphate carboxylase/oxygenase large subunit (rbcL) gene, partial cds; chloroplast	1042	1042	90%	0.0	97.69%	HQ590084.1
Equisetum xylochaetum isolate 40614 ribulose-1,5-bisphosphate carboxylase/oxygenase large subunit (rbcL) gene, partial cds; plastid	1038	1038	93%	0.0	96.50%	MH750228.1
Equisetum myriochaetum isolate 41080 ribulose-1,5-bisphosphate carboxylase/oxygenase large subunit (rbcL) gene, partial cds; plastid	1038	1038	93%	0.0	96.50%	MH750190.1
Equisetum myriochaetum isolate 40826 ribulose-1,5-bisphosphate carboxylase/oxygenase large subunit (rbcL) gene, partial cds; plastid	1038	1038	93%	0.0	96.50%	MH750188.1
Equisetum sylvaticum voucher TJD-502 ribulose-1,5-bisphosphate carboxylase/oxygenase large subunit (rbcL) gene, partial cds; chloroplast	1038	1038	84%	0.0	99.65%	KJ841305.1
Equisetum x schaffneri isolate 40813 ribulose-1,5-bisphosphate carboxylase/oxygenase large subunit (rbcL) gene, partial cds; plastid	1037	1037	93%	0.0	96.50%	MH750224.1
Equisetum bogotense isolate 40800 ribulose-1,5-bisphosphate carboxylase/oxygenase large subunit (rbcL) gene, partial cds; plastid	1037	1037	92%	0.0	96.78%	MH750162.1
Equisetum giganteum isolate 40811 ribulose-1,5-bisphosphate carboxylase/oxygenase large subunit (rbcL) gene, partial cds; plastid	1035	1035	93%	0.0	96.49%	MH750176.1
Equisetum hyemale subsp. hyemale isolate 41088 ribulose-1,5-bisphosphate carboxylase/oxygenase large subunit (rbcL) gene, partial cds; plastid	1033	1033	93%	0.0	96.34%	MH750181.1
Equisetum giganteum isolate 40807 ribulose-1,5-bisphosphate carboxylase/oxygenase large subunit (rbcL) gene, partial cds; plastid	1033	1033	92%	0.0	96.62%	MH750173.1
Equisetum variegatum ribulose-1,5-bisphosphate carboxylase/oxygenase large subunit (rbcL) gene, partial cds; chloroplast gene for chloroplast product	1033	1033	95%	0.0	95.65%	AY226134.1
Equisetum myriochaetum isolate 40936 ribulose-1,5-bisphosphate carboxylase/oxygenase large subunit (rbcL) gene, partial cds; plastid	1029	1029	92%	0.0	96.61%	MH750189.1
Equisetum arvense voucher TJD-508 ribulose-1,5-bisphosphate carboxylase/oxygenase large subunit (rbcL) gene, partial cds; chloroplast	1029	1029	84%	0.0	99.30%	KJ841301.1

Description	Max Score	Total Score	Query Cover	E value	Per. Ident	Accession
Equisetum hyemale subsp. affine isolate 40831 ribulose-1,5-bisphosphate carboxylase/oxygenase large subunit (rbcL) gene, partial cds; plastid	1027	1027	92%	0.0	96.46%	MH750177.1
Equisetum arvense voucher TJD-503 ribulose-1,5-bisphosphate carboxylase/oxygenase large subunit (rbcL) gene, partial cds; chloroplast	1027	1027	84%	0.0	99.30%	KJ841302.1
Equisetum arvense voucher TJD-504 ribulose-1,5-bisphosphate carboxylase/oxygenase large subunit (rbcL) gene, partial cds; chloroplast	1026	1026	84%	0.0	99.30%	KJ841300.1
Equisetum ramosissimum subsp. debile isolate 36802 ribulose-1,5-bisphosphate carboxylase/oxygenase large subunit (rbcL) gene, partial cds; plastid	1024	1024	93%	0.0	96.03%	MH750199.1
Equisetum trachyodon isolate 41092 ribulose-1,5-bisphosphate carboxylase/oxygenase large subunit (rbcL) gene, partial cds; plastid	1022	1022	93%	0.0	96.17%	MH750227.1
Equisetum ramosissimum subsp. debile isolate 40829 ribulose-1,5-bisphosphate carboxylase/oxygenase large subunit (rbcL) gene, partial cds; plastid	1022	1022	93%	0.0	96.03%	MH750200.1
Equisetum myriochaetum isolate 26088 ribulose-1,5-bisphosphate carboxylase/oxygenase large subunit (rbcL) gene, partial cds; plastid	1022	1022	91%	0.0	96.59%	MH750184.1
Equisetum scirpoides chloroplast rbcL gene for ribulose-1,5-bisphosphate carboxylase/oxygenase large subunit, partial cds, specimen_voucher: TNS:768168	1022	1022	89%	0.0	97.49%	AB574689.1
Equisetum hyemale voucher AP344 (OAC) ribulose-1,5-bisphosphate carboxylase/oxygenase large subunit (rbcL) gene, partial cds; chloroplast	1022	1022	91%	0.0	96.60%	EU677107.1
Equisetum laevigatum isolate 40812 ribulose-1,5-bisphosphate carboxylase/oxygenase large subunit (rbcL) gene, partial cds; plastid	1020	1020	91%	0.0	96.59%	MH750183.1
Equisetum giganteum isolate 40809 ribulose-1,5-bisphosphate carboxylase/oxygenase large subunit (rbcL) gene, partial cds; plastid	1020	1020	91%	0.0	96.59%	MH750175.1
Equisetum bogotense isolate 40801 ribulose-1,5-bisphosphate carboxylase/oxygenase large subunit (rbcL) gene, partial cds; plastid	1020	1020	91%	0.0	96.73%	MH750163.1
Equisetum sylvaticum plastid partial rbcL gene for ribulose-1,5-biphosphate carboxylase/oxygenase large subunit, specimen voucher H823764	1020	1020	83%	0.0	99.64%	HE574610.1

Alignments

Equisetum palustre chloroplast, partial genome

Sequence ID: **MT984458.1** Length: 5501 Number of Matches: 1
Range 1: 2213 to 2882

Score	Expect	Identities	Gaps	Strand	Frame
1208 bits(654)	0.0()	665/670(99%)	1/670(0%)	Plus/Plus	

Features:

```
Query  1     ATGTCACCACAAACAGAGACTAAAGCAGGTGTTGGATTTAAAGCTGG-GTTAAAGATTAT  59
             |||||||||||||| ||||| |||||||||||||||||||||||||| ||||||||||||
Sbjct  2213  ATGTCACCACAAACGGAGACCAAAGCAGGTGTTGGATTTAAAGCTGGTGTTAAAGATTAT  2272

Query  60    CGATTGACTTATTTTACTCCAGATTATGAAACCAAAGATACCGATATTTTAGCAGCATTC  119
             ||||||||||||||||||||||||||||||||||||||||||||||||||||||||||||
Sbjct  2273  CGATTGACTTATTTTACTCCAGATTATGAAACCAAAGATACCGATATTTTAGCAGCATTC  2332

Query  120   CGTATGACTCCTCAACCGGGAGTACCACCGGAAGAAGCAGGAGCAGCCGTAGCTGCTGAA  179
             ||||||||||||||||||||||||||||||||||||||||||||||||||||||||||||
Sbjct  2333  CGTATGACTCCTCAACCGGGAGTACCACCGGAAGAAGCAGGAGCAGCCGTAGCTGCTGAA  2392

Query  180   TCGTCCACGGGCACCTGGACTACCGTATGGACAGATGGACTTACTAGTCTTGATCGATAT  239
             ||||||||||||||||||||||||||||||||||||||||||||||||||||||||||||
Sbjct  2393  TCGTCCACGGGCACCTGGACTACCGTATGGACAGATGGACTTACTAGTCTTGATCGATAT  2452

Query  240   AAAGGTCGATGCTATAATATTGAACCTGTTGCTGGAGAAGATAATCAATTCATAGCTTAT  299
             ||||||||||||||||||||||||||||||||||||||||||||||||||||||||||||
Sbjct  2453  AAAGGTCGATGCTATAATATTGAACCTGTTGCTGGAGAAGATAATCAATTCATAGCTTAT  2512

Query  300   GTAGCCTATCCTTTAGATCTTTTTGAAGAAGGTTCTGTTACCAATCTTTTTACTTCAATT  359
             ||||||||||||||||||||||||||||||||||||||||||||||||||||||||||||
Sbjct  2513  GTAGCCTATCCTTTAGATCTTTTTGAAGAAGGTTCTGTTACCAATCTTTTTACTTCAATT  2572

Query  360   GTTGGTAATGTTTTCGGCTTCAAAGCTCTACGTGCTTTACGTTTAGAAGATTTAAGAATT  419
             ||||||||||||||||||||||||||||||||||||||||||||||||||||||||||||
Sbjct  2573  GTTGGTAATGTTTTCGGCTTCAAAGCTCTACGTGCTTTACGTTTAGAAGATTTAAGAATT  2632

Query  420   CCTCCTGCTTACTCCAAAACTTTTATAGGACCACCCCACGGTATCCAGGTTGAAAGAGAT  479
             ||||||||||||||||||||||||||||||||||||||||||||||||||||||||||||
Sbjct  2633  CCTCCTGCTTACTCCAAAACTTTTATAGGACCACCCCACGGTATCCAGGTTGAAAGAGAT  2692

Query  480   AAGTTAAACAAATATGGTCGTCCTTTATTAGGTTGTACAATTAAACCAAAATTGGGACTA  539
             ||||||||||||||||||||||||||||||||||||||||||||||||||||||||||||
Sbjct  2693  AAGTTAAACAAATATGGTCGTCCTTTATTAGGTTGTACAATTAAACCAAAATTGGGACTA  2752

Query  540   TCTGCTAAAAACTATGGTAGGGCTGTTTATGAATGTCTTCGTGGTGGACTTGATTTCACC  599
             ||||||||||||||||||||||||||||||||||||||||||||||||||||||||||||
Sbjct  2753  TCTGCTAAAAACTATGGTAGGGCTGTTTATGAATGTCTTCGTGGTGGACTTGATTTCACC  2812

Query  600   AAAGATGATGAGAATGTAAACTCTCAACCCTTTATGCGTTGGAGAGATCGATTCTTATTT  659
             |||||||||||||||||||||||||||||||||||||||||||||||||| ||||||||
Sbjct  2813  AAAGATGATGAGAATGTAAACTCTCAACCCTTTATGCGTTGGAGAGATCGTTTCTTATTC  2872

Query  660   GTAGCAGAAG  669
             ||||||||||
Sbjct  2873  GTAGCAGAAG  2882
```

Equisetum arvense chloroplast, complete genome

Sequence ID: **GU191334.1** Length: 133309 Number of Matches: 1

Range 1: 27302 to 27971

Score	Expect	Identities	Gaps	Strand	Frame
1186 bits(642)	0.0()	661/670(99%)	1/670(0%)	Plus/Minus	

Features:

```
Query  1      ATGTCACCACAAACAGAGACTAAAGCAGGTGTTGGATTTAAAGCTGG-GTTAAAGATTAT  59
              |||||||||||||| ||||| |||||||||||||||||||||||||| ||||||||||||
Sbjct  27971  ATGTCACCACAAACGGAGACCAAAGCAGGTGTTGGATTTAAAGCTGGTGTTAAAGATTAT  27912

Query  60     CGATTGACTTATTTTACTCCAGATTATGAAACCAAAGATACCGATATTTTAGCAGCATTC  119
              ||||||||||||||||||||||||||||||||||||||||||||||||||||||||||||
Sbjct  27911  CGATTGACTTATTTTACTCCAGATTATGAAACCAAAGATACCGATATTTTAGCAGCATTC  27852

Query  120    CGTATGACTCCTCAACCGGGAGTACCACCGGAAGAAGCAGGAGCAGCCGTAGCTGCTGAA  179
              ||||||||||||||||||||||||||||||||||||||||||||||||||||||||||||
Sbjct  27851  CGTATGACTCCTCAACCGGGAGTACCACCGGAAGAAGCAGGAGCAGCCGTAGCTGCTGAA  27792

Query  180    TCGTCCACGGGCACCTGGACTACCGTATGGACAGATGGACTTACTAGTCTTGATCGATAT  239
              ||||||||||||||||||||||||||||||||||||||||||||||||||||||||||||
Sbjct  27791  TCGTCCACGGGCACCTGGACTACCGTATGGACAGATGGACTTACTAGTCTTGATCGATAT  27732

Query  240    AAAGGTCGATGCTATAATATTGAACCTGTTGCTGGAGAAGATAATCAATTCATAGCTTAT  299
              ||||||||||||||||||||||||||||||||||||||||||||||||||||||||||||
Sbjct  27731  AAAGGTCGATGCTATAATATTGAACCTGTTGCTGGAGAAGATAATCAATTCATAGCTTAT  27672

Query  300    GTAGCCTATCCTTTAGATCTTTTTGAAGAAGGTTCTGTTACCAATCTTTTTACTTCAATT  359
              |||||||| |||||||||||||||||||||||||||||||||||||||||||||||||||
```

```
Sbjct  27671  GTAGCCTACCCTTTAGATCTTTTTGAAGAAGGTTCTGTTACCAATCTTTTTACTTCAATT  27612

Query  360    GTTGGTAATGTTTTCGGCTTCAAAGCTCTACGTGCTTTACGTTTAGAAGATTTAAGAATT  419
              ||||||||||||||||||||||||||||||||||||||||||||||||||||||||||||
Sbjct  27611  GTTGGTAATGTTTTCGGCTTCAAAGCTCTACGTGCTTTACGTTTAGAAGATTTAAGAATT  27552

Query  420    CCTCCTGCTTACTCCAAAACTTTTATAGGACCACCCCACGGTATCCAGGTTGAAAGAGAT  479
              ||||||||||||||||||||||||||||||||||||||||||||||||||||||||||||
Sbjct  27551  CCTCCTGCTTACTCCAAAACTTTTATAGGACCACCCCACGGTATCCAGGTTGAAAGAGAT  27492

Query  480    AAGTTAAACAAATATGGTCGTCCTTTATTAGGTTGTACAATTAAACCAAAATTGGGACTA  539
              |||||||||||||| |||||||||||||||||||||||||||||||||||||||||||||
Sbjct  27491  AAGTTAAACAAATACGGTCGTCCTTTATTAGGTTGTACAATTAAACCAAAATTGGGACTA  27432

Query  540    TCTGCTAAAAACTATGGTAGGGCTGTTTATGAATGTCTTCGTGGTGGACTTGATTTCACC  599
              |||||||||||||||||||| ||||||||||||||||||||||||| |||||||||||||
Sbjct  27431  TCTGCTAAAAACTATGGTAGAGCTGTTTATGAATGTCTTCGTGGTGGGCTTGATTTCACC  27372

Query  600    AAAGATGATGAGAATGTAAACTCTCAACCCTTTATGCGTTGGAGAGATCGATTCTTATTT  659
              |||||||||||||||||||||||||||||||||||||||||||||||||| ||||||||
Sbjct  27371  AAAGATGATGAGAATGTAAACTCTCAACCCTTTATGCGTTGGAGAGATCGTTTCTTATTC  27312

Query  660    GTAGCAGAAG  669
              ||||||||||
Sbjct  27311  GTAGCAGAAG  27302
```

Equisetum arvense chloroplast ribulosebisphosphate carboxylase (rbcL) gene, complete cds
Sequence ID: **L11053.1** Length: 1587 Number of Matches: 1
Range 1: 73 to 742

Score	Expect	Identities	Gaps	Strand	Frame
1186 bits(642)	0.0()	661/670(99%)	1/670(0%)	Plus/Plus	

Features:

```
Query  1    ATGTCACCACAAACAGAGACTAAAGCAGGTGTTGGATTTAAAGCTGG-GTTAAAGATTAT  59
            |||||||||||||| ||||| |||||||||||||||||||||||||| ||||||||||||
Sbjct  73   ATGTCACCACAAACGGAGACCAAAGCAGGTGTTGGATTTAAAGCTGGTGTTAAAGATTAT  132

Query  60   CGATTGACTTATTTTACTCCAGATTATGAAACCAAAGATACCGATATTTTAGCAGCATTC  119
            ||||||||||||||||||||||||||||||||||||||||||||||||||||||||||||
Sbjct  133  CGATTGACTTATTTTACTCCAGATTATGAAACCAAAGATACCGATATTTTAGCAGCATTC  192

Query  120  CGTATGACTCCTCAACCGGGAGTACCACCGGAAGAAGCAGGAGCAGCCGTAGCTGCTGAA  179
            ||||||||||||||||||||||||||||||||||||||||||||||||||||||||||||
Sbjct  193  CGTATGACTCCTCAACCGGGAGTACCACCGGAAGAAGCAGGAGCAGCCGTAGCTGCTGAA  252

Query  180  TCGTCCACGGGCACCTGGACTACCGTATGGACAGATGGACTTACTAGTCTTGATCGATAT  239
            ||||||||||||||||||||||||||||||||||||||||||||||||||||||||||||
Sbjct  253  TCGTCCACGGGCACCTGGACTACCGTATGGACAGATGGACTTACTAGTCTTGATCGATAT  312

Query  240  AAAGGTCGATGCTATAATATTGAACCTGTTGCTGGAGAAGATAATCAATTCATAGCTTAT  299
            ||||||||||||||||||||||||||||||||||||||||||||||||||||||||||||
Sbjct  313  AAAGGTCGATGCTATAATATTGAACCTGTTGCTGGAGAAGATAATCAATTCATAGCTTAT  372

Query  300  GTAGCCTATCCTTTAGATCTTTTTGAAGAAGGTTCTGTTACCAATCTTTTTACTTCAATT  359
            |||||||| |||||||||||||||||||||||||||||||||||||||||||||||||||
Sbjct  373  GTAGCCTACCCTTTAGATCTTTTTGAAGAAGGTTCTGTTACCAATCTTTTTACTTCAATT  432

Query  360  GTTGGTAATGTTTTCGGCTTCAAAGCTCTACGTGCTTTACGTTTAGAAGATTTAAGAATT  419
            ||||||||||||||||||||||||||||||||||||||||||||||||||||||||||||
Sbjct  433  GTTGGTAATGTTTTCGGCTTCAAAGCTCTACGTGCTTTACGTTTAGAAGATTTAAGAATT  492

Query  420  CCTCCTGCTTACTCCAAAACTTTTATAGGACCACCCCACGGTATCCAGGTTGAAAGAGAT  479
            ||||||||||||||||||||||||||||||||||||||||||||||||||||||||||||
Sbjct  493  CCTCCTGCTTACTCCAAAACTTTTATAGGACCACCCCACGGTATCCAGGTTGAAAGAGAT  552

Query  480  AAGTTAAACAAATATGGTCGTCCTTTATTAGGTTGTACAATTAAACCAAAATTGGGACTA  539
            |||||||||||||| |||||||||||||||||||||||||||||||||||||||||||||
Sbjct  553  AAGTTAAACAAATACGGTCGTCCTTTATTAGGTTGTACAATTAAACCAAAATTGGGACTA  612

Query  540  TCTGCTAAAAACTATGGTAGGGCTGTTTATGAATGTCTTCGTGGTGGACTTGATTTCACC  599
            |||||||||||||||||||| ||||||||||||||||||||||||| |||||||||||||
Sbjct  613  TCTGCTAAAAACTATGGTAGAGCTGTTTATGAATGTCTTCGTGGTGGGCTTGATTTCACC  672

Query  600  AAAGATGATGAGAATGTAAACTCTCAACCCTTTATGCGTTGGAGAGATCGATTCTTATTT  659
            |||||||||||||||||||||||||||||||||||||||||||||||||| ||||||||
Sbjct  673  AAAGATGATGAGAATGTAAACTCTCAACCCTTTATGCGTTGGAGAGATCGTTTCTTATTC  732

Query  660  GTAGCAGAAG  669
            ||||||||||
Sbjct  733  GTAGCAGAAG  742
```

Equisetum arvense plastid, complete genome

Sequence ID: **JN968380.1** Length: 132726 Number of Matches: 1

Range 1: 64958 to 65627

Score	Expect	Identities	Gaps	Strand	Frame
1181 bits(639)	0.0()	660/670(99%)	1/670(0%)	Plus/Plus	

Features:

```
Query  1      ATGTCACCACAAACAGAGACTAAAGCAGGTGTTGGATTTAAAGCTGG-GTTAAAGATTAT  59
              |||||||||||||| ||||| |||||||||||||||||||||||||| ||||||||||||
Sbjct  64958  ATGTCACCACAAACGGAGACCAAAGCAGGTGTTGGATTTAAAGCTGGTGTTAAAGATTAT  65017

Query  60     CGATTGACTTATTTTACTCCAGATTATGAAACCAAAGATACCGATATTTTAGCAGCATTC  119
              ||||||||||||||||||||||||||||||||||||||||||||||||||||||||||||
Sbjct  65018  CGATTGACTTATTTTACTCCAGATTATGAAACCAAAGATACCGATATTTTAGCAGCATTC  65077

Query  120    CGTATGACTCCTCAACCGGGAGTACCACCGGAAGAAGCAGGAGCAGCCGTAGCTGCTGAA  179
              ||||||||||||||||||||||||||||||||||||||||||||||||||||||||||||
Sbjct  65078  CGTATGACTCCTCAACCGGGAGTACCACCGGAAGAAGCAGGAGCAGCCGTAGCTGCTGAA  65137

Query  180    TCGTCCACGGGCACCTGGACTACCGTATGGACAGATGGACTTACTAGTCTTGATCGATAT  239
              ||||||||||||||||||||||||||||||||||||||||||||||||||||||||||||
Sbjct  65138  TCGTCCACGGGCACCTGGACTACCGTATGGACAGATGGACTTACTAGTCTTGATCGATAT  65197

Query  240    AAAGGTCGATGCTATAATATTGAACCTGTTGCTGGAGAAGATAATCAATTCATAGCTTAT  299
              ||||||||||||||||||||||||||||||||||||||||||||||||||||||||||||
Sbjct  65198  AAAGGTCGATGCTATAATATTGAACCTGTTGCTGGAGAAGATAATCAATTCATAGCTTAT  65257

Query  300    GTAGCCTATCCTTTAGATCTTTTTGAAGAAGGTTCTGTTACCAATCTTTTTACTTCAATT  359
              |||||||| |||||||||||||||||||||||||||||||||||||||||||||||||||
Sbjct  65258  GTAGCCTACCCTTTAGATCTTTTTGAAGAAGGTTCTGTTACCAATCTTTTTACTTCAATT  65317

Query  360    GTTGGTAATGTTTTCGGCTTCAAAGCTCTACGTGCTTTACGTTTAGAAGATTTAAGAATT  419
              ||||||||||||||||| ||||||||||||||||||||||||||||||||||||||||||
Sbjct  65318  GTTGGTAATGTTTTCGGATTCAAAGCTCTACGTGCTTTACGTTTAGAAGATTTAAGAATT  65377

Query  420    CCTCCTGCTTACTCCAAAACTTTTATAGGACCACCCCACGGTATCCAGGTTGAAAGAGAT  479
              ||||||||||||||||||||||||||||||||||||||||||||||||||||||||||||
Sbjct  65378  CCTCCTGCTTACTCCAAAACTTTTATAGGACCACCCCACGGTATCCAGGTTGAAAGAGAT  65437

Query  480    AAGTTAAACAAATATGGTCGTCCTTTATTAGGTTGTACAATTAAACCAAAATTGGGACTA  539
              |||||||||||||| |||||||||||||||||||||||||||||||||||||||||||||
Sbjct  65438  AAGTTAAACAAATACGGTCGTCCTTTATTAGGTTGTACAATTAAACCAAAATTGGGACTA  65497

Query  540    TCTGCTAAAAACTATGGTAGGGCTGTTTATGAATGTCTTCGTGGTGGACTTGATTTCACC  599
              |||||||||||||||||||| |||||||||||||||||||||||||| ||||||||||||
Sbjct  65498  TCTGCTAAAAACTATGGTAGAGCTGTTTATGAATGTCTTCGTGGTGGGCTTGATTTCACC  65557

Query  600    AAAGATGATGAGAATGTAAACTCTCAACCCTTTATGCGTTGGAGAGATCGATTCTTATTT  659
              |||||||||||||||||||||||||||||||||||||||||||||||||| |||||||| 
Sbjct  65558  AAAGATGATGAGAATGTAAACTCTCAACCCTTTATGCGTTGGAGAGATCGTTTCTTATTC  65617

Query  660    GTAGCAGAAG  669
              ||||||||||
Sbjct  65618  GTAGCAGAAG  65627
```

Equisetum palustre ribulose-1,5-bisphosphate carboxylase/oxygenase large subunit (rbcL) gene, partial cds; chloroplast gene for chloroplast product

Sequence ID: **AY226138.1** Length: 1325 Number of Matches: 1

Range 1: 1 to 644

Score	Expect	Identities	Gaps	Strand	Frame
1171 bits(634)	0.0()	641/644(99%)	1/644(0%)	Plus/Plus	

Features:

```
Query  27   AGGTGTTGGATTTAAAGCTGG-GTTAAAGATTATCGATTGACTTATTTTACTCCAGATTA  85
            ||||||||||||||||||||| ||||||||||||||||||||||||||||||||||||||
Sbjct  1    AGGTGTTGGATTTAAAGCTGGTGTTAAAGATTATCGATTGACTTATTTTACTCCAGATTA  60

Query  86   TGAAACCAAAGATACCGATATTTTAGCAGCATTCCGTATGACTCCTCAACCGGGAGTACC  145
            ||||||||||||||||||||||||||||||||||||||||||||||||||||||||||||
Sbjct  61   TGAAACCAAAGATACCGATATTTTAGCAGCATTCCGTATGACTCCTCAACCGGGAGTACC  120

Query  146  ACCGGAAGAAGCAGGAGCAGCCGTAGCTGCTGAATCGTCCACGGGCACCTGGACTACCGT  205
            ||||||||||||||||||||||||||||||||||||||||||||||||||||||||||||
Sbjct  121  ACCGGAAGAAGCAGGAGCAGCCGTAGCTGCTGAATCGTCCACGGGCACCTGGACTACCGT  180

Query  206  ATGGACAGATGGACTTACTAGTCTTGATCGATATAAAGGTCGATGCTATAATATTGAACC  265
            ||||||||||||||||||||||||||||||||||||||||||||||||||||||||||||
Sbjct  181  ATGGACAGATGGACTTACTAGTCTTGATCGATATAAAGGTCGATGCTATAATATTGAACC  240
```

```
Query  266   TGTTGCTGGAGAAGATAATCAATTCATAGCTTATGTAGCCTATCCTTTAGATCTTTTTGA  325
             ||||||||||||||||||||||||||||||||||||||||||||||||||||||||||||
Sbjct  241   TGTTGCTGGAGAAGATAATCAATTCATAGCTTATGTAGCCTATCCTTTAGATCTTTTTGA  300

Query  326   AGAAGGTTCTGTTACCAATCTTTTTACTTCAATTGTTGGTAATGTTTTCGGCTTCAAAGC  385
             ||||||||||||||||||||||||||||||||||||||||||||||||||||||||||||
Sbjct  301   AGAAGGTTCTGTTACCAATCTTTTTACTTCAATTGTTGGTAATGTTTTCGGCTTCAAAGC  360

Query  386   TCTACGTGCTTTACGTTTAGAAGATTTAAGAATTCCTCCTGCTTACTCCAAAACTTTTAT  445
             ||||||||||||||||||||||||||||||||||||||||||||||||||||||||||||
Sbjct  361   TCTACGTGCTTTACGTTTAGAAGATTTAAGAATTCCTCCTGCTTACTCCAAAACTTTTAT  420

Query  446   AGGACCACCCCACGGTATCCAGGTTGAAAGAGATAAGTTAAACAAATATGGTCGTCCTTT  505
             ||||||||||||||||||||||||||||||||||||||||||||||||||||||||||||
Sbjct  421   AGGACCACCCCACGGTATCCAGGTTGAAAGAGATAAGTTAAACAAATATGGTCGTCCTTT  480

Query  506   ATTAGGTTGTACAATTAAACCAAAATTGGGACTATCTGCTAAAAACTATGGTAGGGCTGT  565
             ||||||||||||||||||||||||||||||||||||||||||||||||||||||||||||
Sbjct  481   ATTAGGTTGTACAATTAAACCAAAATTGGGACTATCTGCTAAAAACTATGGTAGGGCTGT  540

Query  566   TTATGAATGTCTTCGTGGTGGACTTGATTTCACCAAAGATGATGAGAATGTAAACTCTCA  625
             ||||||||||||||||||||||||||||||||||||||||||||||||||||||||||||
Sbjct  541   TTATGAATGTCTTCGTGGTGGACTTGATTTCACCAAAGATGATGAGAATGTAAACTCTCA  600

Query  626   ACCCTTTATGCGTTGGAGAGATCGATTCTTATTTGTAGCAGAAG  669
             |||||||||||||||||||||||| |||||||| ||||||||||
Sbjct  601   ACCCTTTATGCGTTGGAGAGATCGTTTCTTATTCGTAGCAGAAG  644
```


BLAST is a registered trademark of the National Library of Medicine

Support center Mailing list YouTube

- National Library Of Medicine
- National Institutes Of Health
- U.S. Department of Health & Human Services
- USA.gov

NCBI

National Center for Biotechnology Information, U.S. National Library of Medicine 8600 Rockville Pike, Bethesda MD, 20894 USA

Policies and Guidelines | Contact

HHS Vulnerability Disclosure
