# Supplementary material for: A Multi-Pronged Technique for Identifying Equisetum palustre and Equisetum arvense—Combining HPTLC, HPLC-ESI-MS/MS and Optimized DNA Barcoding Techniques
Source: Plants (Basel). 2022 Sep 28;11(19):2562. doi: 10.3390/plants11192562 (PMC9573248; doi:10.3390/plants11192562)
Supplement: Supplementary file 1 [file plants-11-02562-s001.zip › plants-1932149 supplementary/Horsetail Supplementary Figures/Figure S26 (P9, 82715220323-1).pdf]

BLAST ® » blastn suite » RID-J1CJFKN8013

BLAST Results

Questions/comments

Job title: Nucleotide Sequence

**RID**	J1CJFKN8013 (Expires on 09-15 00:39 am)
**Query ID**	lcl|Query_45033
**Description**	None
**Molecule type**	dna
**Query Length**	601

**Database Name**	nt
**Description**	Nucleotide collection (nt)
**Program**	BLASTN 2.13.0+

Graphic Summary

Distribution of the top 100 Blast Hits on 100 subject sequences

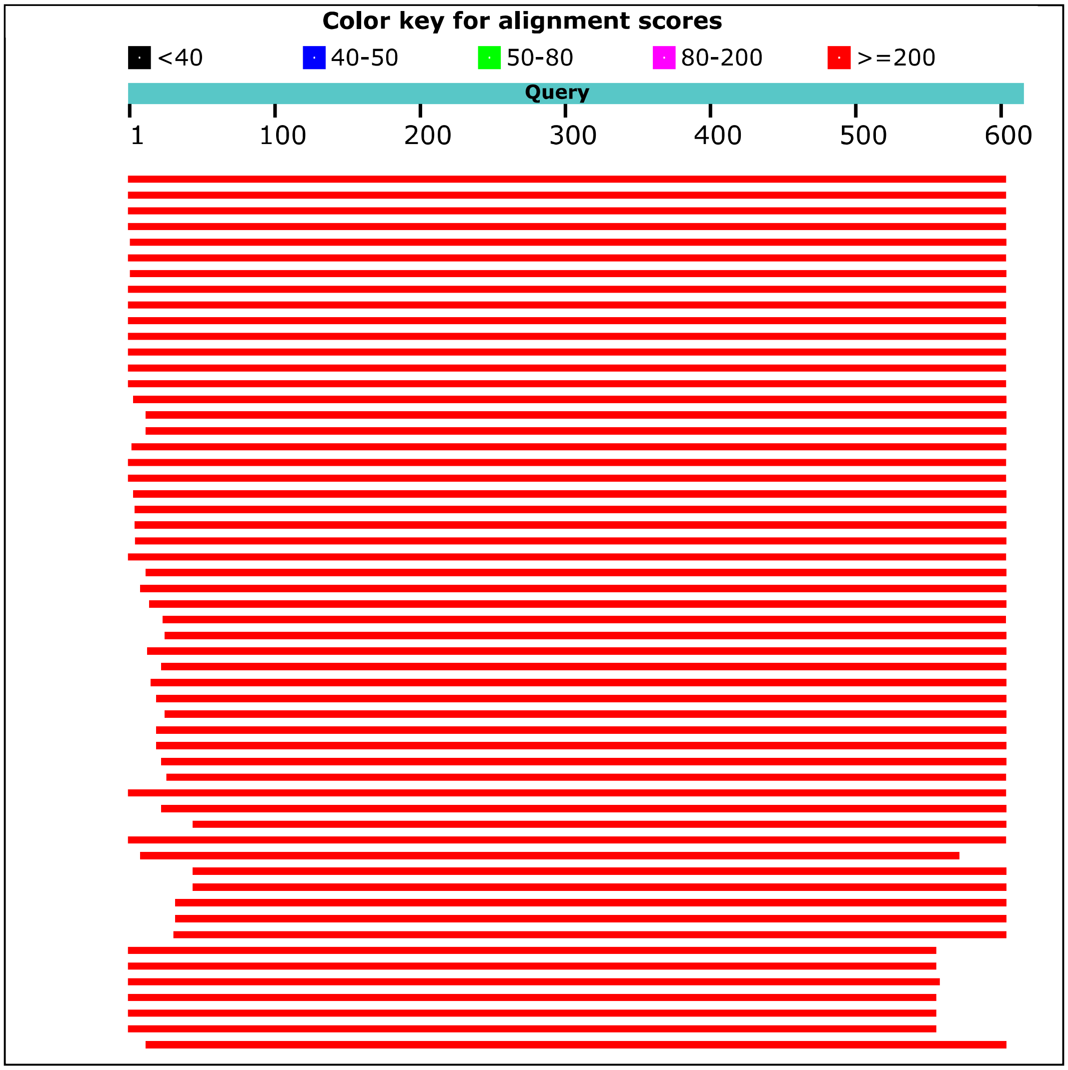

Descriptions

Sequences producing significant alignments:

Description	Max Score	Total Score	Query Cover	E value	Per. Ident	Accession
Equisetum palustre chloroplast, partial genome	1086	1086	100%	0.0	99.34%	MT984458.1
Equisetum palustre ribulose-1,5-bisphosphate carboxylase/oxygenase large subunit (rbcL) gene, partial cds; chloroplast gene for chloroplast product	1086	1086	100%	0.0	99.34%	AY226138.1
Equisetum pratense ribulose-1,5-bisphosphate carboxylase/oxygenase large subunit (rbcL) gene, partial cds; chloroplast gene for chloroplast product	1081	1081	100%	0.0	99.17%	AY226137.1
Equisetum sylvaticum ribulose-1,5-bisphosphate carboxylase/oxygenase large subunit (rbcL) gene, partial cds; chloroplast gene for chloroplast product	1081	1081	100%	0.0	99.17%	AY226136.1
Equisetum sylvaticum isolate 11640 ribulose-1,5-bisphosphate carboxylase/oxygenase large subunit (rbcL) gene, partial cds; plastid	1079	1079	99%	0.0	99.17%	MH750204.1
Equisetum telmateia subsp. braunii ribulose-1,5-bisphosphate carboxylase/oxygenase large subunit (rbcL) gene, partial cds; chloroplast gene for chloroplast product	1070	1070	100%	0.0	98.84%	AY226135.1
Equisetum telmateia subsp. telmateia isolate 11642 ribulose-1,5-bisphosphate carboxylase/oxygenase large subunit (rbcL) gene, partial cds; plastid	1068	1068	99%	0.0	98.84%	MH750210.1
Equisetum environmental sample isolate EDNA16-0043490 ribulose-1,5-bisphosphate carboxylase/oxygenase large subunit (rbcL) gene, partial cds; chloroplast	1064	1064	100%	0.0	98.67%	MT197555.1
Equisetum arvense chloroplast, complete genome	1064	1064	100%	0.0	98.67%	GU191334.1
Equisetum arvense voucher JAG288 (OAC) ribulose-1,5-bisphosphate carboxylase/oxygenase large subunit (rbcL) gene, partial cds; chloroplast	1064	1064	100%	0.0	98.67%	EU677106.1
Equisetum arvense voucher AP400 (OAC) ribulose-1,5-bisphosphate carboxylase/oxygenase large subunit (rbcL) gene, partial cds; chloroplast	1064	1064	100%	0.0	98.67%	EU677105.1
Equisetum fluviatile ribulose-1,5-bisphosphate carboxylase/oxygenase large subunit (rbcL) gene, partial cds; chloroplast gene for chloroplast product	1064	1064	100%	0.0	98.67%	AY226142.1
Equisetum arvense ribulose-1,5-bisphosphate carboxylase/oxygenase large subunit (rbcL) gene, partial cds; chloroplast gene for chloroplast product	1064	1064	100%	0.0	98.67%	AY226140.1
Equisetum arvense chloroplast ribulosebisphosphate carboxylase (rbcL) gene, complete cds	1064	1064	100%	0.0	98.67%	L11053.1

Description	Max Score	Total Score	Query Cover	E value	Per. Ident	Accession
Equisetum telmateia ribulose-1,5-bisphosphate carboxylase large subunit (rbcL) gene, partial cds; chloroplast gene for chloroplast product	1062	1062	99%	0.0	98.83%	AF313580.1
Equisetum sylvaticum isolate 41081 ribulose-1,5-bisphosphate carboxylase/oxygenase large subunit (rbcL) gene, partial cds; plastid	1059	1059	98%	0.0	99.15%	MH750205.1
Equisetum pratense isolate 41087 ribulose-1,5-bisphosphate carboxylase/oxygenase large subunit (rbcL) gene, partial cds; plastid	1059	1059	98%	0.0	99.15%	MH750195.1
Equisetum arvense subsp. boreale isolate 41074 ribulose-1,5-bisphosphate carboxylase/oxygenase large subunit (rbcL) gene, partial cds; plastid	1059	1059	99%	0.0	98.66%	MH750160.1
Equisetum environmental sample isolate EDNA16-0043491b ribulose-1,5-bisphosphate carboxylase/oxygenase large subunit (rbcL) gene, partial cds; chloroplast	1059	1059	100%	0.0	98.50%	MT197548.1
Equisetum arvense plastid, complete genome	1059	1059	100%	0.0	98.50%	JN968380.1
Equisetum x dycei isolate 26083 ribulose-1,5-bisphosphate carboxylase/oxygenase large subunit (rbcL) gene, partial cds; plastid	1057	1057	99%	0.0	98.66%	MH750220.1
Equisetum fluviatile isolate 41076 ribulose-1,5-bisphosphate carboxylase/oxygenase large subunit (rbcL) gene, partial cds; plastid	1055	1055	99%	0.0	98.66%	MH750169.1
Equisetum fluviatile isolate 41075 ribulose-1,5-bisphosphate carboxylase/oxygenase large subunit (rbcL) gene, partial cds; plastid	1055	1055	99%	0.0	98.66%	MH750168.1
Equisetum pratense voucher AP451 ribulose-1,5-bisphosphate carboxylase/oxygenase large subunit (rbcL) gene, partial cds; chloroplast	1055	1055	99%	0.0	98.66%	HQ590083.1
Equisetum diffusum ribulose-1,5-bisphosphate carboxylase/oxygenase large subunit (rbcL) gene, partial cds; chloroplast gene for chloroplast product	1053	1053	100%	0.0	98.34%	AY226141.1
Equisetum telmateia subsp. braunii isolate 40828 ribulose-1,5-bisphosphate carboxylase/oxygenase large subunit (rbcL) gene, partial cds; plastid	1048	1048	98%	0.0	98.81%	MH750207.1
Equisetum arvense subsp. boreale isolate 41073 ribulose-1,5-bisphosphate carboxylase/oxygenase large subunit (rbcL) gene, partial cds; plastid	1048	1048	98%	0.0	98.65%	MH750159.1
Equisetum x fontqueri isolate 26093 ribulose-1,5-bisphosphate carboxylase/oxygenase large subunit (rbcL) gene, partial cds; plastid	1044	1044	97%	0.0	98.81%	MH750221.1
Equisetum palustre isolate 17671 ribulose-1,5-bisphosphate carboxylase/oxygenase large subunit (rbcL) gene, partial cds; plastid	1042	1042	96%	0.0	99.31%	MH750191.1

Description	Max Score	Total Score	Query Cover	E value	Per. Ident	Accession
Equisetum palustre isolate 39349 ribulose-1,5-bisphosphate carboxylase/oxygenase large subunit (rbcL) gene, partial cds; plastid	1040	1040	95%	0.0	99.31%	MH750192.1
Equisetum arvense subsp. arvense isolate 41071 ribulose-1,5-bisphosphate carboxylase/oxygenase large subunit (rbcL) gene, partial cds; plastid	1040	1040	97%	0.0	98.64%	MH750158.1
Equisetum pratense isolate 41086 ribulose-1,5-bisphosphate carboxylase/oxygenase large subunit (rbcL) gene, partial cds; plastid	1038	1038	96%	0.0	99.14%	MH750194.1
Equisetum x litorale isolate 41084 ribulose-1,5-bisphosphate carboxylase/oxygenase large subunit (rbcL) gene, partial cds; plastid	1035	1035	97%	0.0	98.63%	MH750222.1
Equisetum telmateia subsp. braunii isolate 40832 ribulose-1,5-bisphosphate carboxylase/oxygenase large subunit (rbcL) gene, partial cds; plastid	1035	1035	96%	0.0	98.80%	MH750208.1
Equisetum pratense isolate 39348 ribulose-1,5-bisphosphate carboxylase/oxygenase large subunit (rbcL) gene, partial cds; plastid	1035	1035	95%	0.0	99.13%	MH750193.1
Equisetum arvense subsp. arvense isolate 26085 ribulose-1,5-bisphosphate carboxylase/oxygenase large subunit (rbcL) gene, partial cds; plastid	1029	1029	96%	0.0	98.63%	MH750156.1
Equisetum diffusum isolate 40689 ribulose-1,5-bisphosphate carboxylase/oxygenase large subunit (rbcL) gene, partial cds; plastid	1024	1024	96%	0.0	98.46%	MH750166.1
Equisetum arvense subsp. arvense isolate 26084 ribulose-1,5-bisphosphate carboxylase/oxygenase large subunit (rbcL) gene, partial cds; plastid	1022	1022	96%	0.0	98.62%	MH750155.1
Equisetum arvense subsp. arvense isolate 40833 ribulose-1,5-bisphosphate carboxylase/oxygenase large subunit (rbcL) gene, partial cds; plastid	1014	1014	95%	0.0	98.61%	MH750157.1
Equisetum scirpoides voucher AP248 ribulose-1,5-bisphosphate carboxylase/oxygenase large subunit (rbcL) gene, partial cds; chloroplast	1014	1014	100%	0.0	97.18%	HQ590084.1
Equisetum diffusum isolate 40804 ribulose-1,5-bisphosphate carboxylase/oxygenase large subunit (rbcL) gene, partial cds; plastid	1011	1011	96%	0.0	98.27%	MH750167.1
Equisetum palustre chloroplast rbcL gene for ribulose-1,5-bisphosphate carboxylase/oxygenase large subunit, partial cds, specimen_voucher: TNS:776981	1011	1011	92%	0.0	99.46%	AB574686.1
Equisetum scirpoides ribulose-1,5-bisphosphate carboxylase/oxygenase large subunit (rbcL) gene, partial cds; chloroplast gene for chloroplast product	1009	1009	100%	0.0	97.01%	AY226133.1
Equisetum sylvaticum plastid partial rbcL gene for ribulose-1,5-biphosphate carboxylase/oxygenase large subunit, specimen voucher H823764	1007	1007	93%	0.0	99.11%	HE574610.1

Description	Max Score	Total Score	Query Cover	E value	Per. Ident	Accession
Equisetum sylvaticum chloroplast rbcL gene for ribulose-1,5-bisphosphate carboxylase/oxygenase large subunit, partial cds, specimen_voucher: TNS:776974	1005	1005	92%	0.0	99.28%	AB574690.1
Equisetum pratense chloroplast rbcL gene for ribulose-1,5-bisphosphate carboxylase/oxygenase large subunit, partial cds, specimen_voucher: TNS:1107843	1005	1005	92%	0.0	99.28%	AB574687.1
Equisetum fluviatile ribulose-1,5-bisphosphate carboxylase/oxygenase large subunit (rbcL) gene, partial cds; chloroplast	1005	1005	94%	0.0	98.60%	DQ463101.1
Equisetum arvense x Equisetum telmateia subsp. braunii isolate 40834 ribulose-1,5-bisphosphate carboxylase/oxygenase large subunit (rbcL) gene, partial cds; plastid	1003	1003	94%	0.0	98.59%	MH750161.1
Equisetum arvense ribulose-1,5-bisphosphate carboxylase/oxygenase large subunit (rbcL) gene, partial cds; chloroplast	1002	1002	94%	0.0	98.42%	KM218345.1
Equisetum palustre voucher Gillespie_8830b ribulose-1,5-bisphosphate carboxylase/oxygenase large subunit (rbcL) gene, partial cds; chloroplast	1002	1002	92%	0.0	99.46%	KC482730.1
Equisetum palustre voucher Chase 11641 (K) ribulose-1,5-bisphosphate carboxylase/oxygenase large subunit (rbcL) gene, partial cds; chloroplast	1002	1002	92%	0.0	99.46%	GQ248601.1
Equisetum sylvaticum voucher TJD-502 ribulose-1,5-bisphosphate carboxylase/oxygenase large subunit (rbcL) gene, partial cds; chloroplast	1000	1000	92%	0.0	99.28%	KJ841305.1
Equisetum sylvaticum voucher WAB_1079140180 ribulose-1,5-bisphosphate carboxylase/oxygenase large subunit (rbcL) gene, partial cds; chloroplast	996	996	92%	0.0	99.28%	KJ593395.1
Equisetum pratense voucher WAB_0132469185 ribulose-1,5-bisphosphate carboxylase/oxygenase large subunit (rbcL) gene, partial cds; chloroplast	996	996	92%	0.0	99.28%	KJ593391.1
Equisetum sylvaticum voucher Gillespie_8524 ribulose-1,5-bisphosphate carboxylase/oxygenase large subunit (rbcL) gene, partial cds; chloroplast	996	996	92%	0.0	99.28%	KC482739.1
Equisetum scirpoides isolate 41089 ribulose-1,5-bisphosphate carboxylase/oxygenase large subunit (rbcL) gene, partial cds; plastid	992	992	98%	0.0	97.12%	MH750203.1
Equisetum komarovii isolate 40830 ribulose-1,5-bisphosphate carboxylase/oxygenase large subunit (rbcL) gene, partial cds; plastid	992	992	98%	0.0	97.12%	MH750182.1

Description	Max Score	Total Score	Query Cover	E value	Per. Ident	Accession
Equisetum telmateia voucher CCDB-18287-A07 ribulose-1,5-bisphosphate carboxylase/oxygenase large subunit (rbcL) gene, partial cds; chloroplast	990	990	92%	0.0	99.10%	MG215015.1
Equisetum arvense voucher TJD-503 ribulose-1,5-bisphosphate carboxylase/oxygenase large subunit (rbcL) gene, partial cds; chloroplast	989	989	92%	0.0	98.92%	KJ841302.1
Equisetum arvense voucher TJD-508 ribulose-1,5-bisphosphate carboxylase/oxygenase large subunit (rbcL) gene, partial cds; chloroplast	989	989	92%	0.0	98.92%	KJ841301.1
Equisetum arvense voucher TJD-504 ribulose-1,5-bisphosphate carboxylase/oxygenase large subunit (rbcL) gene, partial cds; chloroplast	989	989	92%	0.0	98.92%	KJ841300.1
Equisetum fluviatile chloroplast rbcL gene for ribulose-1,5-bisphosphate carboxylase/oxygenase large subunit, partial cds, specimen_voucher: TNS:776384	989	989	92%	0.0	98.75%	AB574684.1
Equisetum xylochaetum chloroplast, complete genome	987	987	100%	0.0	96.35%	MW282958.1
Equisetum sp. Schuettpelz 1643 ribulose-1,5-bisphosphate carboxylase/oxygenase large subunit (rbcL) gene, partial cds; chloroplast	987	987	100%	0.0	96.35%	MW620286.1
Equisetum arvense subsp. alpestre voucher Gillespie_10109_CAN ribulose-1,5-bisphosphate carboxylase/oxygenase large subunit (rbcL) gene, partial cds; chloroplast	987	987	92%	0.0	98.92%	KC482713.1
Equisetum bogotense ribulose-1,5-bisphosphate carboxylase/oxygenase large subunit (rbcL) gene, partial cds; chloroplast gene for chloroplast product	987	987	100%	0.0	96.35%	AY226139.1
Equisetum myriochaetum ribulose-1,5-bisphosphate carboxylase/oxygenase large subunit (rbcL) gene, partial cds; chloroplast gene for chloroplast product	987	987	100%	0.0	96.35%	AY226131.1
Equisetum laevigatum ribulose-1,5-bisphosphate carboxylase/oxygenase large subunit (rbcL) gene, partial cds; chloroplast gene for chloroplast product	987	987	100%	0.0	96.35%	AY226130.1
Equisetum giganteum ribulose-1,5-bisphosphate carboxylase/oxygenase large subunit (rbcL) gene, partial cds; chloroplast gene for chloroplast product	987	987	100%	0.0	96.35%	AY226127.1
Equisetum giganteum isolate 40806 ribulose-1,5-bisphosphate carboxylase/oxygenase large subunit (rbcL) gene, partial cds; plastid	985	985	99%	0.0	96.34%	MH750172.1
Equisetum giganteum isolate 26087 ribulose-1,5-bisphosphate carboxylase/oxygenase large subunit (rbcL) gene, partial cds; plastid	985	985	99%	0.0	96.34%	MH750170.1
Equisetum arvense voucher WAB_1079140221 ribulose-1,5-bisphosphate carboxylase/oxygenase large subunit (rbcL) gene, partial cds; chloroplast	985	985	92%	0.0	98.92%	KJ593388.1

Description	Max Score	Total Score	Query Cover	E value	Per. Ident	Accession
Equisetum fluviatile voucher 09PROBE-05547 ribulose-1,5-bisphosphate carboxylase/oxygenase large subunit (rbcL) gene, partial cds; chloroplast	985	985	92%	0.0	98.92%	JN965528.1
Equisetum x ferrissii ribulose-1,5-bisphosphate carboxylase/oxygenase large subunit (rbcL) gene, partial cds; chloroplast	985	985	99%	0.0	96.34%	EU352292.1
Equisetum arvense voucher G00199177 ribulose-1,5-bisphosphate carboxylase/oxygenase large subunit (rbcL) gene, partial cds; chloroplast	983	983	91%	0.0	98.91%	KJ204336.1
Equisetum arvense ribulose-1,5-bisphosphate carboxylase/oxygenase large subunit (rbcL) gene, partial cds; chloroplast	983	983	91%	0.0	98.91%	MZ359289.1
Equisetum arvense chloroplast rbcL gene for ribulose-1,5-bisphosphate carboxylase/oxygenase large subunit, partial cds, specimen_voucher: TNS:763618	983	983	92%	0.0	98.57%	AB574683.1
Equisetum x ferrissii ribulose-1,5-bisphosphate carboxylase large subunit (rbcL) gene, partial cds; chloroplast gene for chloroplast product	983	983	99%	0.0	96.33%	AF313579.1
Equisetum scirpoides isolate 26090 ribulose-1,5-bisphosphate carboxylase/oxygenase large subunit (rbcL) gene, partial cds; plastid	981	981	98%	0.0	96.78%	MH750202.1
Equisetum arvense voucher BIOUG24048-F01 ribulose-1,5-bisphosphate carboxylase/oxygenase large subunit (rbcL) gene, partial cds; chloroplast	981	981	91%	0.0	98.91%	KT695464.1
Equisetum hyemale subsp. affine voucher OAC 94916 ribulose-1,5-bisphosphate carboxylase/oxygenase large subunit (rbcL) gene, partial cds; chloroplast	981	981	100%	0.0	96.18%	KF186500.1
Equisetum hyemale chloroplast, complete genome	981	981	100%	0.0	96.18%	KC117177.1
Commelina diffusa voucher Q555 ribulose-1,5-bisphosphate carboxylase/oxygenase large subunit (rbcL) gene, partial cds; chloroplast	981	981	92%	0.0	98.74%	MH714073.1
Equisetum hyemale voucher AP344 (OAC) ribulose-1,5-bisphosphate carboxylase/oxygenase large subunit (rbcL) gene, partial cds; chloroplast	981	981	100%	0.0	96.18%	EU677107.1
Equisetum hyemale subsp. affine ribulose-1,5-bisphosphate carboxylase/oxygenase large subunit (rbcL) gene, partial cds; chloroplast gene for chloroplast product	981	981	100%	0.0	96.18%	AY226128.1
Equisetum palustre ribulose-1,5-bisphosphate carboxylase/oxygenase large subunit (rbcL) gene, partial cds; chloroplast	979	979	90%	0.0	99.45%	MK525785.1
Equisetum sylvaticum ribulose-1,5-bisphosphate carboxylase/oxygenase large subunit (rbcL) gene, partial cds; chloroplast	974	974	90%	0.0	99.26%	MK525787.1

Description	Max Score	Total Score	Query Cover	E value	Per. Ident	Accession
Equisetum pratense ribulose-1,5-bisphosphate carboxylase/oxygenase large subunit (rbcL) gene, partial cds; chloroplast	974	974	90%	0.0	99.26%	MK525786.1
Equisetum scirpoides isolate 10933 ribulose-1,5-bisphosphate carboxylase/oxygenase large subunit (rbcL) gene, partial cds; plastid	972	972	96%	0.0	97.06%	MH750201.1
Equisetum myriochaetum isolate 40815 ribulose-1,5-bisphosphate carboxylase/oxygenase large subunit (rbcL) gene, partial cds; plastid	972	972	99%	0.0	96.15%	MH750185.1
Equisetum ramosissimum voucher R825 ribulose-1,5-bisphosphate carboxylase/oxygenase large subunit (rbcl) gene, partial cds; chloroplast	970	970	100%	0.0	95.85%	KY656709.1
Equisetum sylvaticum voucher WAB_0132469351 ribulose-1,5-bisphosphate carboxylase/oxygenase large subunit (rbcL) gene, partial cds; chloroplast	970	970	89%	0.0	99.26%	KJ593397.1
Equisetum ramosissimum isolate BPTPS123 voucher LISE:96444 chloroplast, complete genome	970	970	100%	0.0	95.85%	ON641349.1
Equisetum ramosissimum isolate BPTPS056 voucher LISE:96334 chloroplast, complete genome	970	970	100%	0.0	95.85%	NC_062377.1
Equisetum ramosissimum isolate BPTPS056 voucher LISE:96334 chloroplast, complete genome	970	970	100%	0.0	95.85%	OM691674.1
Equisetum ramosissimum subsp. debile voucher G.M. Plunkett 3642 (NYBG) ribulose-1,5-bisphosphate carboxylase/oxygenase large subunit (rbcL) gene, partial cds; chloroplast	970	970	100%	0.0	95.85%	MT657901.1
Equisetum hyemale ribulose-1,5-bisphosphate carboxylase/oxygenase large subunit (rbcL) gene, partial cds; chloroplast	970	970	100%	0.0	95.85%	DQ646001.1
Equisetum ramosissimum subsp. debile ribulose-1,5-bisphosphate carboxylase/oxygenase large subunit (rbcL) gene, partial cds; chloroplast gene for chloroplast product	970	970	100%	0.0	95.85%	AY226132.1
Equisetum xylochaetum isolate 40614 ribulose-1,5-bisphosphate carboxylase/oxygenase large subunit (rbcL) gene, partial cds; plastid	965	965	98%	0.0	96.27%	MH750228.1
Equisetum myriochaetum isolate 41080 ribulose-1,5-bisphosphate carboxylase/oxygenase large subunit (rbcL) gene, partial cds; plastid	965	965	98%	0.0	96.27%	MH750190.1

Alignments

Equisetum palustre chloroplast, partial genome

Sequence ID: **MT984458.1** Length: 5501 Number of Matches: 1

Range 1: 2242 to 2840

Score	Expect	Identities	Gaps	Strand	Frame
1086 bits(588)	0.0()	598/602(99%)	4/602(0%)	Plus/Plus	

Features:

```
Query  1     TGTTGGATTTAAAGCTGGTGTTAAAGATTATCGATTGAACTTATTTTACTCCAGATTATG  60
             ||||||||||||||||||||||||||||||||||||| ||||||||||||||||||||||
Sbjct  2242  TGTTGGATTTAAAGCTGGTGTTAAAGATTATCGATTG-ACTTATTTTACTCCAGATTATG  2300

Query  61    AAACCAAAGATACCGATATTTTAGCAGCATTCCGTATGACTCCTCAACCGGGAGTACCAC  120
             ||||||||||||||||||||||||||||||||||||||||||||||||||||||||||||
Sbjct  2301  AAACCAAAGATACCGATATTTTAGCAGCATTCCGTATGACTCCTCAACCGGGAGTACCAC  2360

Query  121   CGGAAGAAGCAGGAGCAGCCGTAGCTGCTGAATCGTCCACGGGCACCTGGACTACCGTAT  180
             ||||||||||||||||||||||||||||||||||||||||||||||||||||||||||||
Sbjct  2361  CGGAAGAAGCAGGAGCAGCCGTAGCTGCTGAATCGTCCACGGGCACCTGGACTACCGTAT  2420

Query  181   GGACAGATGGACTTACTAGTCTTGATCGATATAAAGGTCGATGCTATAATATTGAACCTG  240
             ||||||||||||||||||||||||||||||||||||||||||||||||||||||||||||
Sbjct  2421  GGACAGATGGACTTACTAGTCTTGATCGATATAAAGGTCGATGCTATAATATTGAACCTG  2480

Query  241   TTGCTGGGAGAAGATAATCAATTCATAGCTTATGTAGCCTATCCTTTAGATCTTTTTGAA  300
             ||||| ||||||||||||||||||||||||||||||||||||||||||||||||||||||
Sbjct  2481  TTGCT-GGAGAAGATAATCAATTCATAGCTTATGTAGCCTATCCTTTAGATCTTTTTGAA  2539

Query  301   GAAGGTTCTGTTACCAATCTTTTTACTTCAATTGTTGGTAATGTTTTCGGCTTCAAAGCT  360
             ||||||||||||||||||||||||||||||||||||||||||||||||||||||||||||
Sbjct  2540  GAAGGTTCTGTTACCAATCTTTTTACTTCAATTGTTGGTAATGTTTTCGGCTTCAAAGCT  2599

Query  361   CTACGTGCTTTACGTTTAGAAGATTTAAGAATTCCTCCTGCTTACTCCAAAACTTTTATA  420
             ||||||||||||||||||||||||||||||||||||||||||||||||||||||||||||
Sbjct  2600  CTACGTGCTTTACGTTTAGAAGATTTAAGAATTCCTCCTGCTTACTCCAAAACTTTTATA  2659

Query  421   GGACCACCCCACGGTATCCAGGTTGAAAGAGATAAGTTAAAACAAATATGGTCGTCCTTT  480
             |||||||||||||||||||||||||||||||||||||| |||||||||||||||||||||
Sbjct  2660  GGACCACCCCACGGTATCCAGGTTGAAAGAGATAAGTT-AAACAAATATGGTCGTCCTTT  2718

Query  481   ATTAGGTTGTACAATTAAACCAAAATTGGGACTATCTGCTAAAAACTATGGTAGGGCTGT  540
             ||||||||||||||||||||||||||||||||||||||||||||||||||||||||||||
Sbjct  2719  ATTAGGTTGTACAATTAAACCAAAATTGGGACTATCTGCTAAAAACTATGGTAGGGCTGT  2778

Query  541   TTATGAATGTCTTCGTGG-GGACTTGATTTCACCAAAGATGATGAGAATGTAAACTCTCA  599
             |||||||||||||||||| |||||||||||||||||||||||||||||||||||||||||
Sbjct  2779  TTATGAATGTCTTCGTGGTGGACTTGATTTCACCAAAGATGATGAGAATGTAAACTCTCA  2838

Query  600   AC  601
             ||
Sbjct  2839  AC  2840
```

Equisetum palustre ribulose-1,5-bisphosphate carboxylase/oxygenase large subunit (rbcL) gene, partial cds; chloroplast gene for chloroplast product

Sequence ID: **AY226138.1** Length: 1325 Number of Matches: 1

Range 1: 4 to 602

Score	Expect	Identities	Gaps	Strand	Frame
1086 bits(588)	0.0()	598/602(99%)	4/602(0%)	Plus/Plus	

Features:

```
Query  1    TGTTGGATTTAAAGCTGGTGTTAAAGATTATCGATTGAACTTATTTTACTCCAGATTATG  60
            ||||||||||||||||||||||||||||||||||||| ||||||||||||||||||||||
Sbjct  4    TGTTGGATTTAAAGCTGGTGTTAAAGATTATCGATTG-ACTTATTTTACTCCAGATTATG  62

Query  61   AAACCAAAGATACCGATATTTTAGCAGCATTCCGTATGACTCCTCAACCGGGAGTACCAC  120
            ||||||||||||||||||||||||||||||||||||||||||||||||||||||||||||
Sbjct  63   AAACCAAAGATACCGATATTTTAGCAGCATTCCGTATGACTCCTCAACCGGGAGTACCAC  122

Query  121  CGGAAGAAGCAGGAGCAGCCGTAGCTGCTGAATCGTCCACGGGCACCTGGACTACCGTAT  180
            ||||||||||||||||||||||||||||||||||||||||||||||||||||||||||||
Sbjct  123  CGGAAGAAGCAGGAGCAGCCGTAGCTGCTGAATCGTCCACGGGCACCTGGACTACCGTAT  182

Query  181  GGACAGATGGACTTACTAGTCTTGATCGATATAAAGGTCGATGCTATAATATTGAACCTG  240
            ||||||||||||||||||||||||||||||||||||||||||||||||||||||||||||
Sbjct  183  GGACAGATGGACTTACTAGTCTTGATCGATATAAAGGTCGATGCTATAATATTGAACCTG  242

Query  241  TTGCTGGGAGAAGATAATCAATTCATAGCTTATGTAGCCTATCCTTTAGATCTTTTTGAA  300
            ||||| ||||||||||||||||||||||||||||||||||||||||||||||||||||||
Sbjct  243  TTGCT-GGAGAAGATAATCAATTCATAGCTTATGTAGCCTATCCTTTAGATCTTTTTGAA  301

Query  301  GAAGGTTCTGTTACCAATCTTTTTACTTCAATTGTTGGTAATGTTTTCGGCTTCAAAGCT  360
            ||||||||||||||||||||||||||||||||||||||||||||||||||||||||||||
Sbjct  302  GAAGGTTCTGTTACCAATCTTTTTACTTCAATTGTTGGTAATGTTTTCGGCTTCAAAGCT  361

Query  361  CTACGTGCTTTACGTTTAGAAGATTTAAGAATTCCTCCTGCTTACTCCAAAACTTTTATA  420
```

```
          ||||||||||||||||||||||||||||||||||||||||||||||||||||||||||||
Sbjct  362  CTACGTGCTTTACGTTTAGAAGATTTAAGAATTCCTCCTGCTTACTCCAAAACTTTTATA  421

Query  421  GGACCACCCCACGGTATCCAGGTTGAAAGAGATAAGTTAAAACAAATATGGTCGTCCTTT  480
            |||||||||||||||||||||||||||||||||||||| |||||||||||||||||||||
Sbjct  422  GGACCACCCCACGGTATCCAGGTTGAAAGAGATAAGTT-AAACAAATATGGTCGTCCTTT  480

Query  481  ATTAGGTTGTACAATTAAACCAAAATTGGGACTATCTGCTAAAAACTATGGTAGGGCTGT  540
            ||||||||||||||||||||||||||||||||||||||||||||||||||||||||||||
Sbjct  481  ATTAGGTTGTACAATTAAACCAAAATTGGGACTATCTGCTAAAAACTATGGTAGGGCTGT  540

Query  541  TTATGAATGTCTTCGTGG-GGACTTGATTTCACCAAAGATGATGAGAATGTAAACTCTCA  599
            |||||||||||||||||| |||||||||||||||||||||||||||||||||||||||||
Sbjct  541  TTATGAATGTCTTCGTGGTGGACTTGATTTCACCAAAGATGATGAGAATGTAAACTCTCA  600

Query  600  AC  601
            ||
Sbjct  601  AC  602
```

Equisetum pratense ribulose-1,5-bisphosphate carboxylase/oxygenase large subunit (rbcL) gene, partial cds; chloroplast gene for chloroplast product

Sequence ID: **AY226137.1** Length: 1325 Number of Matches: 1

Range 1: 4 to 602

Score	Expect	Identities	Gaps	Strand	Frame
1081 bits(585)	0.0()	597/602(99%)	4/602(0%)	Plus/Plus	

Features:

```
Query  1    TGTTGGATTTAAAGCTGGTGTTAAAGATTATCGATTGAACTTATTTTACTCCAGATTATG  60
            ||||||||||||||||||||||||||||||||||||| ||||||||||||||||||||||
Sbjct  4    TGTTGGATTTAAAGCTGGTGTTAAAGATTATCGATTG-ACTTATTTTACTCCAGATTATG  62

Query  61   AAACCAAAGATACCGATATTTTAGCAGCATTCCGTATGACTCCTCAACCGGGAGTACCAC  120
            ||||||||||||||||||||||||||||||||||||||||||||||||||||||||||||
Sbjct  63   AAACCAAAGATACCGATATTTTAGCAGCATTCCGTATGACTCCTCAACCGGGAGTACCAC  122

Query  121  CGGAAGAAGCAGGAGCAGCCGTAGCTGCTGAATCGTCCACGGGCACCTGGACTACCGTAT  180
            ||||||||||||||||||||||||||||||||||||||||||||||||||||||||||||
Sbjct  123  CGGAAGAAGCAGGAGCAGCCGTAGCTGCTGAATCGTCCACGGGCACCTGGACTACCGTAT  182

Query  181  GGACAGATGGACTTACTAGTCTTGATCGATATAAAGGTCGATGCTATAATATTGAACCTG  240
            ||||||||||||||||||||||||||||||||||||||||||||||||||||||||||||
Sbjct  183  GGACAGATGGACTTACTAGTCTTGATCGATATAAAGGTCGATGCTATAATATTGAACCTG  242

Query  241  TTGCTGGGAGAAGATAATCAATTCATAGCTTATGTAGCCTATCCTTTAGATCTTTTTGAA  300
            ||||| ||||||||||||||||||||||||||||||||||||||||||||||||||||||
Sbjct  243  TTGCT-GGAGAAGATAATCAATTCATAGCTTATGTAGCCTATCCTTTAGATCTTTTTGAA  301

Query  301  GAAGGTTCTGTTACCAATCTTTTTACTTCAATTGTTGGTAATGTTTTCGGCTTCAAAGCT  360
            ||||||||||||||||| ||||||||||||||||||||||||||||||||||||||||||
Sbjct  302  GAAGGTTCTGTTACCAACCTTTTTACTTCAATTGTTGGTAATGTTTTCGGCTTCAAAGCT  361

Query  361  CTACGTGCTTTACGTTTAGAAGATTTAAGAATTCCTCCTGCTTACTCCAAAACTTTTATA  420
            ||||||||||||||||||||||||||||||||||||||||||||||||||||||||||||
Sbjct  362  CTACGTGCTTTACGTTTAGAAGATTTAAGAATTCCTCCTGCTTACTCCAAAACTTTTATA  421

Query  421  GGACCACCCCACGGTATCCAGGTTGAAAGAGATAAGTTAAAACAAATATGGTCGTCCTTT  480
            |||||||||||||||||||||||||||||||||||||| |||||||||||||||||||||
Sbjct  422  GGACCACCCCACGGTATCCAGGTTGAAAGAGATAAGTT-AAACAAATATGGTCGTCCTTT  480

Query  481  ATTAGGTTGTACAATTAAACCAAAATTGGGACTATCTGCTAAAAACTATGGTAGGGCTGT  540
            ||||||||||||||||||||||||||||||||||||||||||||||||||||||||||||
Sbjct  481  ATTAGGTTGTACAATTAAACCAAAATTGGGACTATCTGCTAAAAACTATGGTAGGGCTGT  540

Query  541  TTATGAATGTCTTCGTGG-GGACTTGATTTCACCAAAGATGATGAGAATGTAAACTCTCA  599
            |||||||||||||||||| |||||||||||||||||||||||||||||||||||||||||
Sbjct  541  TTATGAATGTCTTCGTGGTGGACTTGATTTCACCAAAGATGATGAGAATGTAAACTCTCA  600

Query  600  AC  601
            ||
Sbjct  601  AC  602
```

Equisetum sylvaticum ribulose-1,5-bisphosphate carboxylase/oxygenase large subunit (rbcL) gene, partial cds; chloroplast gene for chloroplast product

Sequence ID: **AY226136.1** Length: 1324 Number of Matches: 1

Range 1: 4 to 602

Score	Expect	Identities	Gaps	Strand	Frame

1081 bits(585)	0.0()	597/602(99%)	4/602(0%)	Plus/Plus	

Features:

```
Query  1    TGTTGGATTTAAAGCTGGTGTTAAAGATTATCGATTGAACTTATTTTACTCCAGATTATG  60
            ||||||||||||||||||||||||||||||||||||| ||||||||||||||||||||||
Sbjct  4    TGTTGGATTTAAAGCTGGTGTTAAAGATTATCGATTG-ACTTATTTTACTCCAGATTATG  62

Query  61   AAACCAAAGATACCGATATTTTAGCAGCATTCCGTATGACTCCTCAACCGGGAGTACCAC  120
            ||||||||||||||||||||||||||||||||||||||||||||||||||||||||||||
Sbjct  63   AAACCAAAGATACCGATATTTTAGCAGCATTCCGTATGACTCCTCAACCGGGAGTACCAC  122

Query  121  CGGAAGAAGCAGGAGCAGCCGTAGCTGCTGAATCGTCCACGGGCACCTGGACTACCGTAT  180
            ||||||||||||||||||||||||||||||||||||||||||||||||||||||||||||
Sbjct  123  CGGAAGAAGCAGGAGCAGCCGTAGCTGCTGAATCGTCCACGGGCACCTGGACTACCGTAT  182

Query  181  GGACAGATGGACTTACTAGTCTTGATCGATATAAAGGTCGATGCTATAATATTGAACCTG  240
            ||||||||||||||||||||||||||||||||||||||||||||||||||||||||||||
Sbjct  183  GGACAGATGGACTTACTAGTCTTGATCGATATAAAGGTCGATGCTATAATATTGAACCTG  242

Query  241  TTGCTGGGAGAAGATAATCAATTCATAGCTTATGTAGCCTATCCTTTAGATCTTTTTGAA  300
            ||||| ||||||||||||||||||||||||||||||||||||||||||||||||||||||
Sbjct  243  TTGCT-GGAGAAGATAATCAATTCATAGCTTATGTAGCCTATCCTTTAGATCTTTTTGAA  301

Query  301  GAAGGTTCTGTTACCAATCTTTTTACTTCAATTGTTGGTAATGTTTTCGGCTTCAAAGCT  360
            ||||||||||||||||||||||||||||||||||||||||||||||||||||||||||||
Sbjct  302  GAAGGTTCTGTTACCAATCTTTTTACTTCAATTGTTGGTAATGTTTTCGGCTTCAAAGCT  361

Query  361  CTACGTGCTTTACGTTTAGAAGATTTAAGAATTCCTCCTGCTTACTCCAAAACTTTTATA  420
            |||||||||||||||||||||||||||||||||||||||||||| |||||||||||||||
Sbjct  362  CTACGTGCTTTACGTTTAGAAGATTTAAGAATTCCTCCTGCTTATTCCAAAACTTTTATA  421

Query  421  GGACCACCCCACGGTATCCAGGTTGAAAGAGATAAGTTAAAACAAATATGGTCGTCCTTT  480
            |||||||||||||||||||||||||||||||||||||| |||||||||||||||||||||
Sbjct  422  GGACCACCCCACGGTATCCAGGTTGAAAGAGATAAGTT-AAACAAATATGGTCGTCCTTT  480

Query  481  ATTAGGTTGTACAATTAAACCAAAATTGGGACTATCTGCTAAAAACTATGGTAGGGCTGT  540
            ||||||||||||||||||||||||||||||||||||||||||||||||||||||||||||
Sbjct  481  ATTAGGTTGTACAATTAAACCAAAATTGGGACTATCTGCTAAAAACTATGGTAGGGCTGT  540

Query  541  TTATGAATGTCTTCGTGG-GGACTTGATTTCACCAAAGATGATGAGAATGTAAACTCTCA  599
            |||||||||||||||||| |||||||||||||||||||||||||||||||||||||||||
Sbjct  541  TTATGAATGTCTTCGTGGTGGACTTGATTTCACCAAAGATGATGAGAATGTAAACTCTCA  600

Query  600  AC  601
            ||
Sbjct  601  AC  602
```

Equisetum sylvaticum isolate 11640 ribulose-1,5-bisphosphate carboxylase/oxygenase large subunit (rbcL) gene, partial cds; plastid

Sequence ID: **MH750204.1** Length: 1307 Number of Matches: 1

Range 1: 1 to 598

**Score**	**Expect**	**Identities**	**Gaps**	**Strand**	**Frame**
1079 bits(584)	0.0()	596/601(99%)	4/601(0%)	Plus/Plus	

Features:

```
Query  2    GTTGGATTTAAAGCTGGTGTTAAAGATTATCGATTGAACTTATTTTACTCCAGATTATGA  61
            |||||||||||||||||||||||||||||||||||| |||||||||||||||||||||||
Sbjct  1    GTTGGATTTAAAGCTGGTGTTAAAGATTATCGATTG-ACTTATTTTACTCCAGATTATGA  59

Query  62   AACCAAAGATACCGATATTTTAGCAGCATTCCGTATGACTCCTCAACCGGGAGTACCACC  121
            ||||||||||||||||||||||||||||||||||||||||||||||||||||||||||||
Sbjct  60   AACCAAAGATACCGATATTTTAGCAGCATTCCGTATGACTCCTCAACCGGGAGTACCACC  119

Query  122  GGAAGAAGCAGGAGCAGCCGTAGCTGCTGAATCGTCCACGGGCACCTGGACTACCGTATG  181
            ||||||||||||||||||||||||||||||||||||||||||||||||||||||||||||
Sbjct  120  GGAAGAAGCAGGAGCAGCCGTAGCTGCTGAATCGTCCACGGGCACCTGGACTACCGTATG  179

Query  182  GACAGATGGACTTACTAGTCTTGATCGATATAAAGGTCGATGCTATAATATTGAACCTGT  241
            ||||||||||||||||||||||||||||||||||||||||||||||||||||||||||||
Sbjct  180  GACAGATGGACTTACTAGTCTTGATCGATATAAAGGTCGATGCTATAATATTGAACCTGT  239

Query  242  TGCTGGGAGAAGATAATCAATTCATAGCTTATGTAGCCTATCCTTTAGATCTTTTTGAAG  301
            |||| |||||||||||||||||||||||||||||||||||||||||||||||||||||||
Sbjct  240  TGCT-GGAGAAGATAATCAATTCATAGCTTATGTAGCCTATCCTTTAGATCTTTTTGAAG  298

Query  302  AAGGTTCTGTTACCAATCTTTTTACTTCAATTGTTGGTAATGTTTTCGGCTTCAAAGCTC  361
            ||||||||||||||||||||||||||||||||||||||||||||||||||||||||||||
Sbjct  299  AAGGTTCTGTTACCAATCTTTTTACTTCAATTGTTGGTAATGTTTTCGGCTTCAAAGCTC  358

Query  362  TACGTGCTTTACGTTTAGAAGATTTAAGAATTCCTCCTGCTTACTCCAAAACTTTTATAG  421
            ||||||||||||||||||||||||||||||||||||||||||| ||||||||||||||||
Sbjct  359  TACGTGCTTTACGTTTAGAAGATTTAAGAATTCCTCCTGCTTATTCCAAAACTTTTATAG  418

Query  422  GACCACCCCACGGTATCCAGGTTGAAAGAGATAAGTTAAAACAAATATGGTCGTCCTTTA  481
            ||||||||||||||||||||||||||||||||||||| ||||||||||||||||||||||
```

```
Sbjct  419  GACCACCCCACGGTATCCAGGTTGAAAGAGATAAGTT-AAACAAATATGGTCGTCCTTTA  477

Query  482  TTAGGTTGTACAATTAAACCAAAATTGGGACTATCTGCTAAAAACTATGGTAGGGCTGTT  541
            ||||||||||||||||||||||||||||||||||||||||||||||||||||||||||||
Sbjct  478  TTAGGTTGTACAATTAAACCAAAATTGGGACTATCTGCTAAAAACTATGGTAGGGCTGTT  537

Query  542  TATGAATGTCTTCGTGG-GGACTTGATTTCACCAAAGATGATGAGAATGTAAACTCTCAA  600
            ||||||||||||||||| ||||||||||||||||||||||||||||||||||||||||||
Sbjct  538  TATGAATGTCTTCGTGGTGGACTTGATTTCACCAAAGATGATGAGAATGTAAACTCTCAA  597

Query  601  C  601
            |
Sbjct  598  C  598
```


BLAST is a registered trademark of the National Library of Medicine

Support center Mailing list YouTube

- National Library Of Medicine
- National Institutes Of Health
- U.S. Department of Health & Human Services
- USA.gov

NCBI

National Center for Biotechnology Information, U.S. National Library of Medicine 8600 Rockville Pike, Bethesda MD, 20894 USA

Policies and Guidelines | Contact

HHS Vulnerability Disclosure
