# Supplementary material for: A Multi-Pronged Technique for Identifying Equisetum palustre and Equisetum arvense—Combining HPTLC, HPLC-ESI-MS/MS and Optimized DNA Barcoding Techniques
Source: Plants (Basel). 2022 Sep 28;11(19):2562. doi: 10.3390/plants11192562 (PMC9573248; doi:10.3390/plants11192562)
Supplement: Supplementary file 1 [file plants-11-02562-s001.zip › plants-1932149 supplementary/Horsetail Supplementary Figures/Figure S27 (P10, 82716220323-1).pdf]

BLAST Results

Questions/comments

Job title: Nucleotide Sequence

**RID**	J1CNFFP3013 (Expires on 09-15 00:41 am)
**Query ID**	lcl|Query_19957
**Description**	None
**Molecule type**	dna
**Query Length**	667
**Database Name**	nt
**Description**	Nucleotide collection (nt)
**Program**	BLASTN 2.13.0+

Graphic Summary

Distribution of the top 100 Blast Hits on 100 subject sequences

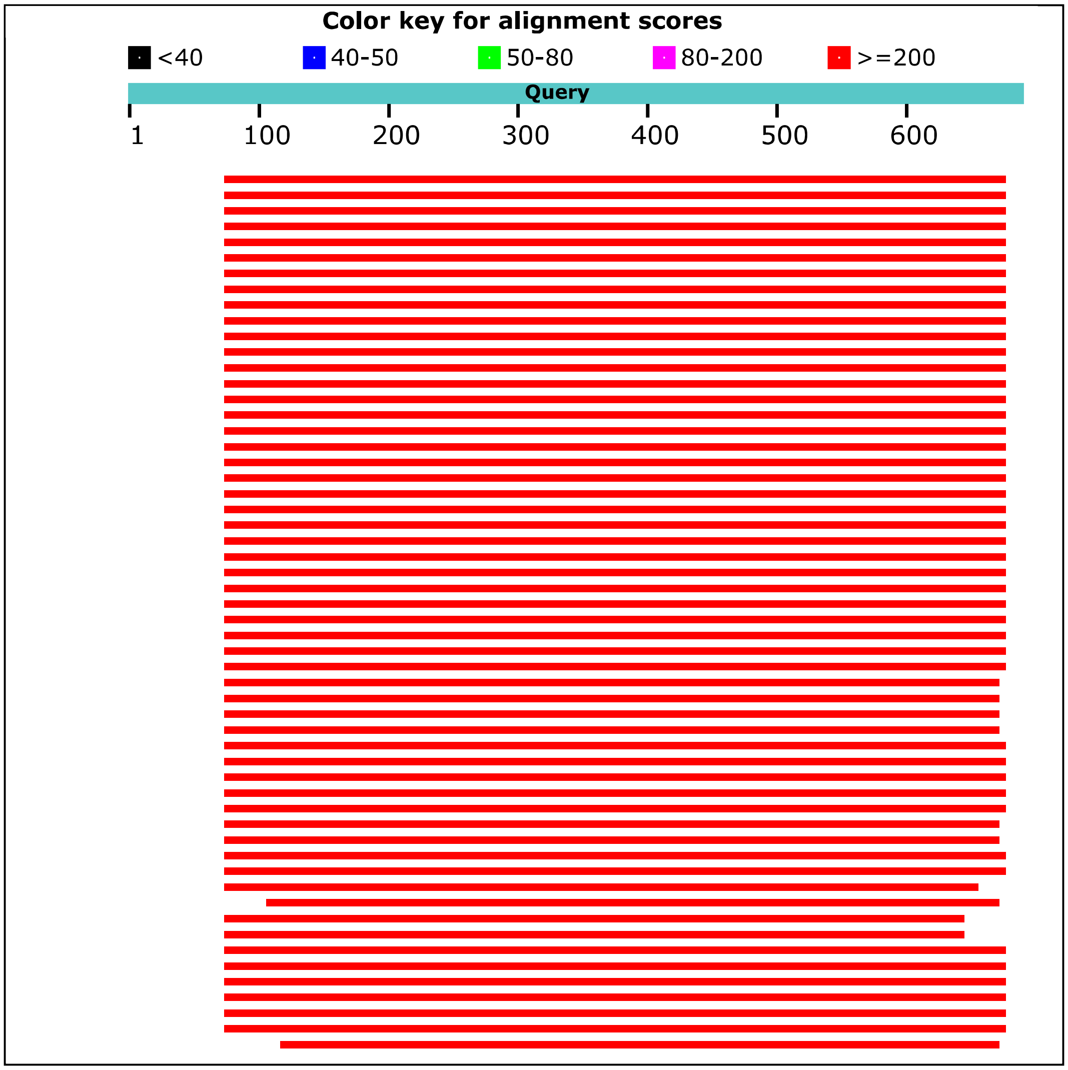

Descriptions

Sequences producing significant alignments:

Description	Max Score	Total Score	Query Cover	E value	Per. Ident	Accession
Equisetum palustre isolate 39349 ribulose-1,5-bisphosphate carboxylase/oxygenase large subunit (rbcL) gene, partial cds; plastid	1007	1007	89%	0.0	97.31%	MH750192.1
Equisetum palustre isolate 17671 ribulose-1,5-bisphosphate carboxylase/oxygenase large subunit (rbcL) gene, partial cds; plastid	1007	1007	89%	0.0	97.31%	MH750191.1
Equisetum palustre chloroplast, partial genome	1007	1007	89%	0.0	97.31%	MT984458.1
Equisetum palustre chloroplast rbcL gene for ribulose-1,5-bisphosphate carboxylase/oxygenase large subunit, partial cds, specimen_voucher: TNS:776981	1007	1007	89%	0.0	97.31%	AB574686.1
Equisetum palustre ribulose-1,5-bisphosphate carboxylase/oxygenase large subunit (rbcL) gene, partial cds; chloroplast gene for chloroplast product	1007	1007	89%	0.0	97.31%	AY226138.1
Equisetum sylvaticum isolate 41081 ribulose-1,5-bisphosphate carboxylase/oxygenase large subunit (rbcL) gene, partial cds; plastid	996	996	89%	0.0	96.97%	MH750205.1
Equisetum sylvaticum isolate 11640 ribulose-1,5-bisphosphate carboxylase/oxygenase large subunit (rbcL) gene, partial cds; plastid	996	996	89%	0.0	96.97%	MH750204.1
Equisetum pratense isolate 41087 ribulose-1,5-bisphosphate carboxylase/oxygenase large subunit (rbcL) gene, partial cds; plastid	996	996	89%	0.0	96.97%	MH750195.1
Equisetum pratense isolate 41086 ribulose-1,5-bisphosphate carboxylase/oxygenase large subunit (rbcL) gene, partial cds; plastid	996	996	89%	0.0	96.97%	MH750194.1
Equisetum pratense isolate 39348 ribulose-1,5-bisphosphate carboxylase/oxygenase large subunit (rbcL) gene, partial cds; plastid	996	996	89%	0.0	96.97%	MH750193.1
Equisetum sylvaticum chloroplast rbcL gene for ribulose-1,5-bisphosphate carboxylase/oxygenase large subunit, partial cds, specimen_voucher: TNS:776974	996	996	89%	0.0	96.97%	AB574690.1
Equisetum pratense chloroplast rbcL gene for ribulose-1,5-bisphosphate carboxylase/oxygenase large subunit, partial cds, specimen_voucher: TNS:1107843	996	996	89%	0.0	96.97%	AB574687.1
Equisetum pratense ribulose-1,5-bisphosphate carboxylase/oxygenase large subunit (rbcL) gene, partial cds; chloroplast gene for chloroplast product	996	996	89%	0.0	96.97%	AY226137.1

Description	Max Score	Total Score	Query Cover	E value	Per. Ident	Accession
Equisetum sylvaticum ribulose-1,5-bisphosphate carboxylase/oxygenase large subunit (rbcL) gene, partial cds; chloroplast gene for chloroplast product	996	996	89%	0.0	96.97%	AY226136.1
Equisetum x litorale isolate 41084 ribulose-1,5-bisphosphate carboxylase/oxygenase large subunit (rbcL) gene, partial cds; plastid	990	990	89%	0.0	96.81%	MH750222.1
Equisetum x dycei isolate 26083 ribulose-1,5-bisphosphate carboxylase/oxygenase large subunit (rbcL) gene, partial cds; plastid	990	990	89%	0.0	96.81%	MH750220.1
Equisetum fluviatile isolate 41076 ribulose-1,5-bisphosphate carboxylase/oxygenase large subunit (rbcL) gene, partial cds; plastid	990	990	89%	0.0	96.81%	MH750169.1
Equisetum fluviatile isolate 41075 ribulose-1,5-bisphosphate carboxylase/oxygenase large subunit (rbcL) gene, partial cds; plastid	990	990	89%	0.0	96.81%	MH750168.1
Equisetum arvense x Equisetum telmateia subsp. braunii isolate 40834 ribulose-1,5-bisphosphate carboxylase/oxygenase large subunit (rbcL) gene, partial cds; plastid	990	990	89%	0.0	96.81%	MH750161.1
Equisetum arvense subsp. boreale isolate 41074 ribulose-1,5-bisphosphate carboxylase/oxygenase large subunit (rbcL) gene, partial cds; plastid	990	990	89%	0.0	96.81%	MH750160.1
Equisetum arvense subsp. boreale isolate 41073 ribulose-1,5-bisphosphate carboxylase/oxygenase large subunit (rbcL) gene, partial cds; plastid	990	990	89%	0.0	96.81%	MH750159.1
Equisetum arvense subsp. arvense isolate 41071 ribulose-1,5-bisphosphate carboxylase/oxygenase large subunit (rbcL) gene, partial cds; plastid	990	990	89%	0.0	96.81%	MH750158.1
Equisetum arvense subsp. arvense isolate 40833 ribulose-1,5-bisphosphate carboxylase/oxygenase large subunit (rbcL) gene, partial cds; plastid	990	990	89%	0.0	96.81%	MH750157.1
Equisetum arvense subsp. arvense isolate 26085 ribulose-1,5-bisphosphate carboxylase/oxygenase large subunit (rbcL) gene, partial cds; plastid	990	990	89%	0.0	96.81%	MH750156.1
Equisetum arvense subsp. arvense isolate 26084 ribulose-1,5-bisphosphate carboxylase/oxygenase large subunit (rbcL) gene, partial cds; plastid	990	990	89%	0.0	96.81%	MH750155.1
Equisetum environmental sample isolate EDNA16-0043490 ribulose-1,5-bisphosphate carboxylase/oxygenase large subunit (rbcL) gene, partial cds; chloroplast	990	990	89%	0.0	96.81%	MT197555.1
Equisetum fluviatile chloroplast rbcL gene for ribulose-1,5-bisphosphate carboxylase/oxygenase large subunit, partial cds, specimen_voucher: TNS:776384	990	990	89%	0.0	96.81%	AB574684.1
Equisetum arvense chloroplast, complete genome	990	990	89%	0.0	96.81%	GU191334.1

Description	Max Score	Total Score	Query Cover	E value	Per. Ident	Accession
Equisetum fluviatile ribulose-1,5-bisphosphate carboxylase/oxygenase large subunit (rbcL) gene, partial cds; chloroplast	990	990	89%	0.0	96.81%	DQ463101.1
Equisetum fluviatile ribulose-1,5-bisphosphate carboxylase/oxygenase large subunit (rbcL) gene, partial cds; chloroplast gene for chloroplast product	990	990	89%	0.0	96.81%	AY226142.1
Equisetum arvense ribulose-1,5-bisphosphate carboxylase/oxygenase large subunit (rbcL) gene, partial cds; chloroplast gene for chloroplast product	990	990	89%	0.0	96.81%	AY226140.1
Equisetum arvense chloroplast ribulosebisphosphate carboxylase (rbcL) gene, complete cds	990	990	89%	0.0	96.81%	L11053.1
Equisetum x fontqueri isolate 26093 ribulose-1,5-bisphosphate carboxylase/oxygenase large subunit (rbcL) gene, partial cds; plastid	985	985	88%	0.0	96.94%	MH750221.1
Equisetum telmateia subsp. telmateia isolate 11642 ribulose-1,5-bisphosphate carboxylase/oxygenase large subunit (rbcL) gene, partial cds; plastid	985	985	88%	0.0	96.94%	MH750210.1
Equisetum telmateia subsp. braunii isolate 40832 ribulose-1,5-bisphosphate carboxylase/oxygenase large subunit (rbcL) gene, partial cds; plastid	985	985	88%	0.0	96.94%	MH750208.1
Equisetum telmateia subsp. braunii isolate 40828 ribulose-1,5-bisphosphate carboxylase/oxygenase large subunit (rbcL) gene, partial cds; plastid	985	985	88%	0.0	96.94%	MH750207.1
Equisetum diffusum isolate 40689 ribulose-1,5-bisphosphate carboxylase/oxygenase large subunit (rbcL) gene, partial cds; plastid	985	985	89%	0.0	96.64%	MH750166.1
Equisetum arvense ribulose-1,5-bisphosphate carboxylase/oxygenase large subunit (rbcL) gene, partial cds; chloroplast	985	985	89%	0.0	96.64%	KM218345.1
Equisetum environmental sample isolate EDNA16-0043491b ribulose-1,5-bisphosphate carboxylase/oxygenase large subunit (rbcL) gene, partial cds; chloroplast	985	985	89%	0.0	96.64%	MT197548.1
Equisetum arvense plastid, complete genome	985	985	89%	0.0	96.64%	JN968380.1
Equisetum arvense chloroplast rbcL gene for ribulose-1,5-bisphosphate carboxylase/oxygenase large subunit, partial cds, specimen_voucher: TNS:763618	985	985	89%	0.0	96.64%	AB574683.1
Equisetum telmateia subsp. braunii ribulose-1,5-bisphosphate carboxylase/oxygenase large subunit (rbcL) gene, partial cds; chloroplast gene for chloroplast product	985	985	88%	0.0	96.94%	AY226135.1
Equisetum telmateia ribulose-1,5-bisphosphate carboxylase large subunit (rbcL) gene, partial cds; chloroplast gene for chloroplast product	985	985	88%	0.0	96.94%	AF313580.1

Description	Max Score	Total Score	Query Cover	E value	Per. Ident	Accession
Equisetum diffusum isolate 40804 ribulose-1,5-bisphosphate carboxylase/oxygenase large subunit (rbcL) gene, partial cds; plastid	979	979	89%	0.0	96.47%	MH750167.1
Equisetum diffusum ribulose-1,5-bisphosphate carboxylase/oxygenase large subunit (rbcL) gene, partial cds; chloroplast gene for chloroplast product	979	979	89%	0.0	96.47%	AY226141.1
Equisetum arvense voucher JAG288 (OAC) ribulose-1,5-bisphosphate carboxylase/oxygenase large subunit (rbcL) gene, partial cds; chloroplast	961	961	85%	0.0	97.03%	EU677106.1
Equisetum telmateia subsp. braunii isolate 40836 ribulose-1,5-bisphosphate carboxylase/oxygenase large subunit (rbcL) gene, partial cds; plastid	942	942	83%	0.0	97.31%	MH750209.1
Equisetum pratense voucher AP451 ribulose-1,5-bisphosphate carboxylase/oxygenase large subunit (rbcL) gene, partial cds; chloroplast	942	942	84%	0.0	96.98%	HQ590083.1
Equisetum arvense voucher AP400 (OAC) ribulose-1,5-bisphosphate carboxylase/oxygenase large subunit (rbcL) gene, partial cds; chloroplast	942	942	84%	0.0	96.98%	EU677105.1
Equisetum scirpoides isolate 41089 ribulose-1,5-bisphosphate carboxylase/oxygenase large subunit (rbcL) gene, partial cds; plastid	941	941	89%	0.0	95.29%	MH750203.1
Equisetum scirpoides isolate 26090 ribulose-1,5-bisphosphate carboxylase/oxygenase large subunit (rbcL) gene, partial cds; plastid	941	941	89%	0.0	95.29%	MH750202.1
Equisetum scirpoides isolate 10933 ribulose-1,5-bisphosphate carboxylase/oxygenase large subunit (rbcL) gene, partial cds; plastid	941	941	89%	0.0	95.29%	MH750201.1
Equisetum komarovii isolate 40830 ribulose-1,5-bisphosphate carboxylase/oxygenase large subunit (rbcL) gene, partial cds; plastid	941	941	89%	0.0	95.29%	MH750182.1
Equisetum scirpoides chloroplast rbcL gene for ribulose-1,5-bisphosphate carboxylase/oxygenase large subunit, partial cds, specimen_voucher: TNS:768168	941	941	89%	0.0	95.29%	AB574689.1
Equisetum scirpoides ribulose-1,5-bisphosphate carboxylase/oxygenase large subunit (rbcL) gene, partial cds; chloroplast gene for chloroplast product	935	935	89%	0.0	95.13%	AY226133.1
Equisetum telmateia subsp. braunii isolate 40817 ribulose-1,5-bisphosphate carboxylase/oxygenase large subunit (rbcL) gene, partial cds; plastid	928	928	81%	0.0	97.44%	MH750206.1
Equisetum bogotense isolate 40801 ribulose-1,5-bisphosphate carboxylase/oxygenase large subunit (rbcL) gene, partial cds; plastid	913	913	89%	0.0	94.45%	MH750163.1
Equisetum bogotense isolate 40800 ribulose-1,5-bisphosphate carboxylase/oxygenase large subunit (rbcL) gene, partial cds; plastid	913	913	89%	0.0	94.45%	MH750162.1

Description	Max Score	Total Score	Query Cover	E value	Per. Ident	Accession
Equisetum bogotense ribulose-1,5-bisphosphate carboxylase/oxygenase large subunit (rbcL) gene, partial cds; chloroplast gene for chloroplast product	913	913	89%	0.0	94.45%	AY226139.1
Equisetum bogotense isolate 40802 ribulose-1,5-bisphosphate carboxylase/oxygenase large subunit (rbcL) gene, partial cds; plastid	909	909	88%	0.0	94.44%	MH750164.1
Equisetum xylochaetum isolate 40614 ribulose-1,5-bisphosphate carboxylase/oxygenase large subunit (rbcL) gene, partial cds; plastid	902	902	89%	0.0	94.12%	MH750228.1
Equisetum x schaffneri isolate 40813 ribulose-1,5-bisphosphate carboxylase/oxygenase large subunit (rbcL) gene, partial cds; plastid	902	902	89%	0.0	94.12%	MH750224.1
Equisetum myriochaetum isolate 41080 ribulose-1,5-bisphosphate carboxylase/oxygenase large subunit (rbcL) gene, partial cds; plastid	902	902	89%	0.0	94.12%	MH750190.1
Equisetum myriochaetum isolate 40936 ribulose-1,5-bisphosphate carboxylase/oxygenase large subunit (rbcL) gene, partial cds; plastid	902	902	89%	0.0	94.12%	MH750189.1
Equisetum myriochaetum isolate 40826 ribulose-1,5-bisphosphate carboxylase/oxygenase large subunit (rbcL) gene, partial cds; plastid	902	902	89%	0.0	94.12%	MH750188.1
Equisetum myriochaetum isolate 40816 ribulose-1,5-bisphosphate carboxylase/oxygenase large subunit (rbcL) gene, partial cds; plastid	902	902	89%	0.0	94.12%	MH750186.1
Equisetum myriochaetum isolate 40815 ribulose-1,5-bisphosphate carboxylase/oxygenase large subunit (rbcL) gene, partial cds; plastid	902	902	89%	0.0	94.12%	MH750185.1
Equisetum myriochaetum isolate 26088 ribulose-1,5-bisphosphate carboxylase/oxygenase large subunit (rbcL) gene, partial cds; plastid	902	902	89%	0.0	94.12%	MH750184.1
Equisetum laevigatum isolate 40812 ribulose-1,5-bisphosphate carboxylase/oxygenase large subunit (rbcL) gene, partial cds; plastid	902	902	89%	0.0	94.12%	MH750183.1
Equisetum giganteum isolate 40811 ribulose-1,5-bisphosphate carboxylase/oxygenase large subunit (rbcL) gene, partial cds; plastid	902	902	89%	0.0	94.12%	MH750176.1
Equisetum giganteum isolate 40809 ribulose-1,5-bisphosphate carboxylase/oxygenase large subunit (rbcL) gene, partial cds; plastid	902	902	89%	0.0	94.12%	MH750175.1
Equisetum giganteum isolate 40807 ribulose-1,5-bisphosphate carboxylase/oxygenase large subunit (rbcL) gene, partial cds; plastid	902	902	89%	0.0	94.12%	MH750173.1
Equisetum giganteum isolate 40806 ribulose-1,5-bisphosphate carboxylase/oxygenase large subunit (rbcL) gene, partial cds; plastid	902	902	89%	0.0	94.12%	MH750172.1

Description	Max Score	Total Score	Query Cover	E value	Per. Ident	Accession
Equisetum giganteum isolate 26087 ribulose-1,5-bisphosphate carboxylase/oxygenase large subunit (rbcL) gene, partial cds; plastid	902	902	89%	0.0	94.12%	MH750170.1
Equisetum xylochaetum chloroplast, complete genome	902	902	89%	0.0	94.12%	MW282958.1
Equisetum sp. Schuettpelz 1643 ribulose-1,5-bisphosphate carboxylase/oxygenase large subunit (rbcL) gene, partial cds; chloroplast	902	902	89%	0.0	94.12%	MW620286.1
Equisetum x ferrissii ribulose-1,5-bisphosphate carboxylase/oxygenase large subunit (rbcL) gene, partial cds; chloroplast	902	902	89%	0.0	94.12%	EU352292.1
Equisetum myriochaetum ribulose-1,5-bisphosphate carboxylase/oxygenase large subunit (rbcL) gene, partial cds; chloroplast gene for chloroplast product	902	902	89%	0.0	94.12%	AY226131.1
Equisetum laevigatum ribulose-1,5-bisphosphate carboxylase/oxygenase large subunit (rbcL) gene, partial cds; chloroplast gene for chloroplast product	902	902	89%	0.0	94.12%	AY226130.1
Equisetum giganteum ribulose-1,5-bisphosphate carboxylase/oxygenase large subunit (rbcL) gene, partial cds; chloroplast gene for chloroplast product	902	902	89%	0.0	94.12%	AY226127.1
Equisetum x ferrissii ribulose-1,5-bisphosphate carboxylase large subunit (rbcL) gene, partial cds; chloroplast gene for chloroplast product	902	902	89%	0.0	94.12%	AF313579.1
Equisetum hyemale subsp. hyemale isolate 41088 ribulose-1,5-bisphosphate carboxylase/oxygenase large subunit (rbcL) gene, partial cds; plastid	896	896	89%	0.0	93.95%	MH750181.1
Equisetum hyemale subsp. hyemale isolate 23252 ribulose-1,5-bisphosphate carboxylase/oxygenase large subunit (rbcL) gene, partial cds; plastid	896	896	89%	0.0	93.95%	MH750180.1
Equisetum hyemale subsp. hyemale isolate 20201 ribulose-1,5-bisphosphate carboxylase/oxygenase large subunit (rbcL) gene, partial cds; plastid	896	896	89%	0.0	93.95%	MH750179.1
Equisetum hyemale subsp. affine isolate 40831 ribulose-1,5-bisphosphate carboxylase/oxygenase large subunit (rbcL) gene, partial cds; plastid	896	896	89%	0.0	93.95%	MH750177.1
Equisetum hyemale subsp. affine voucher OAC 94916 ribulose-1,5-bisphosphate carboxylase/oxygenase large subunit (rbcL) gene, partial cds; chloroplast	896	896	89%	0.0	93.95%	KF186500.1
Equisetum hyemale chloroplast, complete genome	896	896	89%	0.0	93.95%	KC117177.1
Equisetum hyemale chloroplast rbcL gene for ribulose-1,5-bisphosphate carboxylase/oxygenase large subunit, partial cds, specimen_voucher: TNS:765859	896	896	89%	0.0	93.95%	AB574685.1

Description	Max Score	Total Score	Query Cover	E value	Per. Ident	Accession
Equisetum hyemale ribulose-1,5-bisphosphate carboxylase/oxygenase large subunit (rbcL) gene, partial cds; chloroplast	896	896	89%	0.0	93.95%	DQ646001.1
Equisetum hyemale subsp. affine ribulose-1,5-bisphosphate carboxylase/oxygenase large subunit (rbcL) gene, partial cds; chloroplast gene for chloroplast product	896	896	89%	0.0	93.95%	AY226128.1
Equisetum sylvaticum plastid partial rbcL gene for ribulose-1,5-biphosphate carboxylase/oxygenase large subunit, specimen voucher H823764	894	894	78%	0.0	97.52%	HE574610.1
Equisetum scirpoides voucher AP248 ribulose-1,5-bisphosphate carboxylase/oxygenase large subunit (rbcL) gene, partial cds; chloroplast	893	893	84%	0.0	95.38%	HQ590084.1
Equisetum trachyodon isolate 41092 ribulose-1,5-bisphosphate carboxylase/oxygenase large subunit (rbcL) gene, partial cds; plastid	891	891	89%	0.0	93.78%	MH750227.1
Equisetum variegatum chloroplast rbcL gene for ribulose-1,5-bisphosphate carboxylase/oxygenase large subunit, partial cds, specimen_voucher: TNS:768164	891	891	89%	0.0	93.78%	AB574691.1
Equisetum ramosissimum subsp. debile isolate 40829 ribulose-1,5-bisphosphate carboxylase/oxygenase large subunit (rbcL) gene, partial cds; plastid	885	885	89%	0.0	93.61%	MH750200.1
Equisetum ramosissimum subsp. debile isolate 36802 ribulose-1,5-bisphosphate carboxylase/oxygenase large subunit (rbcL) gene, partial cds; plastid	885	885	89%	0.0	93.61%	MH750199.1
Equisetum ramosissimum isolate BPTPS123 voucher LISE:96444 chloroplast, complete genome	885	885	89%	0.0	93.61%	ON641349.1
Equisetum ramosissimum isolate BPTPS056 voucher LISE:96334 chloroplast, complete genome	885	885	89%	0.0	93.61%	NC_062377.1
Equisetum ramosissimum isolate BPTPS056 voucher LISE:96334 chloroplast, complete genome	885	885	89%	0.0	93.61%	OM691674.1
Equisetum ramosissimum subsp. debile voucher G.M. Plunkett 3642 (NYBG) ribulose-1,5-bisphosphate carboxylase/oxygenase large subunit (rbcL) gene, partial cds; chloroplast	885	885	89%	0.0	93.61%	MT657901.1

Alignments

Equisetum palustre isolate 39349 ribulose-1,5-bisphosphate carboxylase/oxygenase large subunit (rbcL) gene, partial cds; plastid

Sequence ID: **MH750192.1** Length: 1298 Number of Matches: 1

Range 1: 20 to 611

Score	Expect	Identities	Gaps	Strand	Frame
1007 bits(545)	0.0()	579/595(97%)	4/595(0%)	Plus/Plus	

Features:

```
Query  74   TTACTCCAGATTATGAAATCATAGATACCGAGATTTTAGCAGTATTCCGGATGACTCCTC  133
            |||||||||||||||||| || ||||||||| |||||||||| |||||| ||||||||||
Sbjct  20   TTACTCCAGATTATGAAACCAAAGATACCGATATTTTAGCAGCATTCCGTATGACTCCTC  79

Query  134  AACCGGGAGTCCCACCGGAAGAAGAAGGAGCAGCCGTAGTTCCTGAATCGTCCACGGGCA  193
            |||||||||| ||||||||||||| |||||||||||||| | ||||||||||||||||||
Sbjct  80   AACCGGGAGTACCACCGGAAGAAGCAGGAGCAGCCGTAGCTGCTGAATCGTCCACGGGCA  139

Query  194  CCTGGACTACCGTATGGACAGATGGACTTACTAGTCTTGATCGATATAAAGGTCGATGCT  253
            ||||||||||||||||||||||||||||||||||||||||||||||||||||||||||||
Sbjct  140  CCTGGACTACCGTATGGACAGATGGACTTACTAGTCTTGATCGATATAAAGGTCGATGCT  199

Query  254  ATAATATTGAACCTGTTGCTGGAGAAGATAATCAATTCATAGCTTATGTAGCCTATCCTT  313
            ||||||||||||||||||||||||||||||||||||||||||||||||||||||||||||
Sbjct  200  ATAATATTGAACCTGTTGCTGGAGAAGATAATCAATTCATAGCTTATGTAGCCTATCCTT  259

Query  314  TAGATCTTTTTGAAGAAGGTTCTGTTACCAATCTTTTTACTTCAATTGTTGGTAATGTTT  373
            ||||||||||||||||||||||||||||||||||||||||||||||||||||||||||||
Sbjct  260  TAGATCTTTTTGAAGAAGGTTCTGTTACCAATCTTTTTACTTCAATTGTTGGTAATGTTT  319

Query  374  TCGGCTTCAAAGCTCTACGTGCTTTACGTTTAGAAGATTTAAGAATTCCTCCTGCTTACT  433
            ||||||||||||||||||||||||||||||||||||||||||||||||||||||||||||
Sbjct  320  TCGGCTTCAAAGCTCTACGTGCTTTACGTTTAGAAGATTTAAGAATTCCTCCTGCTTACT  379

Query  434  CCAAAACTTTTATAGGACCACCCCACGG-ATCCAGGTTGAAAGAGATAAGTTAAACAAAT  492
            |||||||||||||||||||||||||||| |||||||||||||||||||||||||||||||
Sbjct  380  CCAAAACTTTTATAGGACCACCCCACGGTATCCAGGTTGAAAGAGATAAGTTAAACAAAT  439

Query  493  ATGGTCGTCCTTTATTAGGTTGTACAATTAAACCAAAATTGGGACTATCTGCTAAAAACT  552
            ||||||||||||||||||||||||||||||||||||||||||||||||||||||||||||
Sbjct  440  ATGGTCGTCCTTTATTAGGTTGTACAATTAAACCAAAATTGGGACTATCTGCTAAAAACT  499

Query  553  ATGGTAGGGCTGTTTATGAATGTCTTCGTGGGGGGACTTGATTTCACCAAAGATGATGAG  612
            |||||||||||||||||||||||||||||||  |||||||||||||||||||||||||||
Sbjct  500  ATGGTAGGGCTGTTTATGAATGTCTTCGTGGT-GGACTTGATTTCACCAAAGATGATGAG  558

Query  613  AATGGTAAACTCTCAACCCCTTTATGCGTTGGAGAGATCGATTCTTATTTGTAGC  667
            ||| |||||||||||| ||||||||||||||||||||||| |||||||| |||||
Sbjct  559  AAT-GTAAACTCTCAA-CCCTTTATGCGTTGGAGAGATCGTTTCTTATTCGTAGC  611
```

Equisetum palustre isolate 17671 ribulose-1,5-bisphosphate carboxylase/oxygenase large subunit (rbcL) gene, partial cds; plastid
Sequence ID: **MH750191.1** Length: 1292 Number of Matches: 1
Range 1: 21 to 612

Score	Expect	Identities	Gaps	Strand	Frame
1007 bits(545)	0.0()	579/595(97%)	4/595(0%)	Plus/Plus	

Features:

```
Query  74   TTACTCCAGATTATGAAATCATAGATACCGAGATTTTAGCAGTATTCCGGATGACTCCTC  133
            |||||||||||||||||| || ||||||||| |||||||||| |||||| ||||||||||
Sbjct  21   TTACTCCAGATTATGAAACCAAAGATACCGATATTTTAGCAGCATTCCGTATGACTCCTC  80

Query  134  AACCGGGAGTCCCACCGGAAGAAGAAGGAGCAGCCGTAGTTCCTGAATCGTCCACGGGCA  193
            |||||||||| ||||||||||||| |||||||||||||| | ||||||||||||||||||
Sbjct  81   AACCGGGAGTACCACCGGAAGAAGCAGGAGCAGCCGTAGCTGCTGAATCGTCCACGGGCA  140

Query  194  CCTGGACTACCGTATGGACAGATGGACTTACTAGTCTTGATCGATATAAAGGTCGATGCT  253
            ||||||||||||||||||||||||||||||||||||||||||||||||||||||||||||
Sbjct  141  CCTGGACTACCGTATGGACAGATGGACTTACTAGTCTTGATCGATATAAAGGTCGATGCT  200

Query  254  ATAATATTGAACCTGTTGCTGGAGAAGATAATCAATTCATAGCTTATGTAGCCTATCCTT  313
            ||||||||||||||||||||||||||||||||||||||||||||||||||||||||||||
Sbjct  201  ATAATATTGAACCTGTTGCTGGAGAAGATAATCAATTCATAGCTTATGTAGCCTATCCTT  260

Query  314  TAGATCTTTTTGAAGAAGGTTCTGTTACCAATCTTTTTACTTCAATTGTTGGTAATGTTT  373
            ||||||||||||||||||||||||||||||||||||||||||||||||||||||||||||
Sbjct  261  TAGATCTTTTTGAAGAAGGTTCTGTTACCAATCTTTTTACTTCAATTGTTGGTAATGTTT  320

Query  374  TCGGCTTCAAAGCTCTACGTGCTTTACGTTTAGAAGATTTAAGAATTCCTCCTGCTTACT  433
            ||||||||||||||||||||||||||||||||||||||||||||||||||||||||||||
Sbjct  321  TCGGCTTCAAAGCTCTACGTGCTTTACGTTTAGAAGATTTAAGAATTCCTCCTGCTTACT  380

Query  434  CCAAAACTTTTATAGGACCACCCCACGG-ATCCAGGTTGAAAGAGATAAGTTAAACAAAT  492
            |||||||||||||||||||||||||||| |||||||||||||||||||||||||||||||
Sbjct  381  CCAAAACTTTTATAGGACCACCCCACGGTATCCAGGTTGAAAGAGATAAGTTAAACAAAT  440

Query  493  ATGGTCGTCCTTTATTAGGTTGTACAATTAAACCAAAATTGGGACTATCTGCTAAAAACT  552
            ||||||||||||||||||||||||||||||||||||||||||||||||||||||||||||
Sbjct  441  ATGGTCGTCCTTTATTAGGTTGTACAATTAAACCAAAATTGGGACTATCTGCTAAAAACT  500

Query  553  ATGGTAGGGCTGTTTATGAATGTCTTCGTGGGGGGACTTGATTTCACCAAAGATGATGAG  612
            |||||||||||||||||||||||||||||||  |||||||||||||||||||||||||||
Sbjct  501  ATGGTAGGGCTGTTTATGAATGTCTTCGTGGT-GGACTTGATTTCACCAAAGATGATGAG  559

Query  613  AATGGTAAACTCTCAACCCCTTTATGCGTTGGAGAGATCGATTCTTATTTGTAGC  667
            ||| |||||||||||| ||||||||||||||||||||||| |||||||| |||||
```

```
Sbjct  560    AAT-GTAAACTCTCAA-CCCTTTATGCGTTGGAGAGATCGTTTCTTATTCGTAGC  612
```

Equisetum palustre chloroplast, partial genome

Sequence ID: **MT984458.1** Length: 5501 Number of Matches: 1

Range 1: 2286 to 2877

Score	Expect	Identities	Gaps	Strand	Frame
1007 bits(545)	0.0()	579/595(97%)	4/595(0%)	Plus/Plus	

Features:

```
Query  74     TTACTCCAGATTATGAAATCATAGATACCGAGATTTTAGCAGTATTCCGGATGACTCCTC  133
              |||||||||||||||||| || ||||||||| |||||||||| |||||| ||||||||||
Sbjct  2286   TTACTCCAGATTATGAAACCAAAGATACCGATATTTTAGCAGCATTCCGTATGACTCCTC  2345

Query  134    AACCGGGAGTCCCACCGGAAGAAGAAGGAGCAGCCGTAGTTCCTGAATCGTCCACGGGCA  193
              |||||||||| ||||||||||||| |||||||||||||| | ||||||||||||||||||
Sbjct  2346   AACCGGGAGTACCACCGGAAGAAGCAGGAGCAGCCGTAGCTGCTGAATCGTCCACGGGCA  2405

Query  194    CCTGGACTACCGTATGGACAGATGGACTTACTAGTCTTGATCGATATAAAGGTCGATGCT  253
              ||||||||||||||||||||||||||||||||||||||||||||||||||||||||||||
Sbjct  2406   CCTGGACTACCGTATGGACAGATGGACTTACTAGTCTTGATCGATATAAAGGTCGATGCT  2465

Query  254    ATAATATTGAACCTGTTGCTGGAGAAGATAATCAATTCATAGCTTATGTAGCCTATCCTT  313
              ||||||||||||||||||||||||||||||||||||||||||||||||||||||||||||
Sbjct  2466   ATAATATTGAACCTGTTGCTGGAGAAGATAATCAATTCATAGCTTATGTAGCCTATCCTT  2525

Query  314    TAGATCTTTTTGAAGAAGGTTCTGTTACCAATCTTTTTACTTCAATTGTTGGTAATGTTT  373
              ||||||||||||||||||||||||||||||||||||||||||||||||||||||||||||
Sbjct  2526   TAGATCTTTTTGAAGAAGGTTCTGTTACCAATCTTTTTACTTCAATTGTTGGTAATGTTT  2585

Query  374    TCGGCTTCAAAGCTCTACGTGCTTTACGTTTAGAAGATTTAAGAATTCCTCCTGCTTACT  433
              ||||||||||||||||||||||||||||||||||||||||||||||||||||||||||||
Sbjct  2586   TCGGCTTCAAAGCTCTACGTGCTTTACGTTTAGAAGATTTAAGAATTCCTCCTGCTTACT  2645

Query  434    CCAAAACTTTTATAGGACCACCCCACGG-ATCCAGGTTGAAAGAGATAAGTTAAACAAAT  492
              |||||||||||||||||||||||||||| |||||||||||||||||||||||||||||||
Sbjct  2646   CCAAAACTTTTATAGGACCACCCCACGGTATCCAGGTTGAAAGAGATAAGTTAAACAAAT  2705

Query  493    ATGGTCGTCCTTTATTAGGTTGTACAATTAAACCAAAATTGGGACTATCTGCTAAAAACT  552
              ||||||||||||||||||||||||||||||||||||||||||||||||||||||||||||
Sbjct  2706   ATGGTCGTCCTTTATTAGGTTGTACAATTAAACCAAAATTGGGACTATCTGCTAAAAACT  2765

Query  553    ATGGTAGGGCTGTTTATGAATGTCTTCGTGGGGGGACTTGATTTCACCAAAGATGATGAG  612
              |||||||||||||||||||||||||||||||  |||||||||||||||||||||||||||
Sbjct  2766   ATGGTAGGGCTGTTTATGAATGTCTTCGTGGT-GGACTTGATTTCACCAAAGATGATGAG  2824

Query  613    AATGGTAAACTCTCAACCCCTTTATGCGTTGGAGAGATCGATTCTTATTTGTAGC  667
              ||| |||||||||||| ||||||||||||||||||||||| |||||||| |||||
Sbjct  2825   AAT-GTAAACTCTCAA-CCCTTTATGCGTTGGAGAGATCGTTTCTTATTCGTAGC  2877
```

Equisetum palustre chloroplast rbcL gene for ribulose-1,5-bisphosphate carboxylase/oxygenase large subunit, partial cds, specimen_voucher: TNS:776981

Sequence ID: **AB574686.1** Length: 1205 Number of Matches: 1

Range 1: 2 to 593

Score	Expect	Identities	Gaps	Strand	Frame
1007 bits(545)	0.0()	579/595(97%)	4/595(0%)	Plus/Plus	

Features:

```
Query  74     TTACTCCAGATTATGAAATCATAGATACCGAGATTTTAGCAGTATTCCGGATGACTCCTC  133
              |||||||||||||||||| || ||||||||| |||||||||| |||||| ||||||||||
Sbjct  2      TTACTCCAGATTATGAAACCAAAGATACCGATATTTTAGCAGCATTCCGTATGACTCCTC  61

Query  134    AACCGGGAGTCCCACCGGAAGAAGAAGGAGCAGCCGTAGTTCCTGAATCGTCCACGGGCA  193
              |||||||||| ||||||||||||| |||||||||||||| | ||||||||||||||||||
Sbjct  62     AACCGGGAGTACCACCGGAAGAAGCAGGAGCAGCCGTAGCTGCTGAATCGTCCACGGGCA  121

Query  194    CCTGGACTACCGTATGGACAGATGGACTTACTAGTCTTGATCGATATAAAGGTCGATGCT  253
              ||||||||||||||||||||||||||||||||||||||||||||||||||||||||||||
Sbjct  122    CCTGGACTACCGTATGGACAGATGGACTTACTAGTCTTGATCGATATAAAGGTCGATGCT  181

Query  254    ATAATATTGAACCTGTTGCTGGAGAAGATAATCAATTCATAGCTTATGTAGCCTATCCTT  313
              ||||||||||||||||||||||||||||||||||||||||||||||||||||||||||||
Sbjct  182    ATAATATTGAACCTGTTGCTGGAGAAGATAATCAATTCATAGCTTATGTAGCCTATCCTT  241

Query  314    TAGATCTTTTTGAAGAAGGTTCTGTTACCAATCTTTTTACTTCAATTGTTGGTAATGTTT  373
              ||||||||||||||||||||||||||||||||||||||||||||||||||||||||||||
Sbjct  242    TAGATCTTTTTGAAGAAGGTTCTGTTACCAATCTTTTTACTTCAATTGTTGGTAATGTTT  301
```

```
Query  374   TCGGCTTCAAAGCTCTACGTGCTTTACGTTTAGAAGATTTAAGAATTCCTCCTGCTTACT  433
             ||||||||||||||||||||||||||||||||||||||||||||||||||||||||||||
Sbjct  302   TCGGCTTCAAAGCTCTACGTGCTTTACGTTTAGAAGATTTAAGAATTCCTCCTGCTTACT  361

Query  434   CCAAAACTTTTATAGGACCACCCCACGG-ATCCAGGTTGAAAGAGATAAGTTAAACAAAT  492
             |||||||||||||||||||||||||||| |||||||||||||||||||||||||||||||
Sbjct  362   CCAAAACTTTTATAGGACCACCCCACGGTATCCAGGTTGAAAGAGATAAGTTAAACAAAT  421

Query  493   ATGGTCGTCCTTTATTAGGTTGTACAATTAAACCAAAATTGGGACTATCTGCTAAAAACT  552
             ||||||||||||||||||||||||||||||||||||||||||||||||||||||||||||
Sbjct  422   ATGGTCGTCCTTTATTAGGTTGTACAATTAAACCAAAATTGGGACTATCTGCTAAAAACT  481

Query  553   ATGGTAGGGCTGTTTATGAATGTCTTCGTGGGGGGACTTGATTTCACCAAAGATGATGAG  612
             |||||||||||||||||||||||||||||||  |||||||||||||||||||||||||||
Sbjct  482   ATGGTAGGGCTGTTTATGAATGTCTTCGTGGT-GGACTTGATTTCACCAAAGATGATGAG  540

Query  613   AATGGTAAACTCTCAACCCCTTTATGCGTTGGAGAGATCGATTCTTATTTGTAGC  667
             ||| |||||||||||| ||||||||||||||||||||||| |||||||| |||||
Sbjct  541   AAT-GTAAACTCTCAA-CCCTTTATGCGTTGGAGAGATCGTTTCTTATTCGTAGC  593
```

Equisetum palustre ribulose-1,5-bisphosphate carboxylase/oxygenase large subunit (rbcL) gene, partial cds; chloroplast gene for chloroplast product

Sequence ID: **AY226138.1** Length: 1325 Number of Matches: 1

Range 1: 48 to 639

Score	Expect	Identities	Gaps	Strand	Frame
1007 bits(545)	0.0()	579/595(97%)	4/595(0%)	Plus/Plus	

Features:

```
Query  74    TTACTCCAGATTATGAAATCATAGATACCGAGATTTTAGCAGTATTCCGGATGACTCCTC  133
             |||||||||||||||||| || ||||||||| |||||||||| |||||| ||||||||||
Sbjct  48    TTACTCCAGATTATGAAACCAAAGATACCGATATTTTAGCAGCATTCCGTATGACTCCTC  107

Query  134   AACCGGGAGTCCCACCGGAAGAAGAAGGAGCAGCCGTAGTTCCTGAATCGTCCACGGGCA  193
             |||||||||| ||||||||||||| |||||||||||||| | ||||||||||||||||||
Sbjct  108   AACCGGGAGTACCACCGGAAGAAGCAGGAGCAGCCGTAGCTGCTGAATCGTCCACGGGCA  167

Query  194   CCTGGACTACCGTATGGACAGATGGACTTACTAGTCTTGATCGATATAAAGGTCGATGCT  253
             ||||||||||||||||||||||||||||||||||||||||||||||||||||||||||||
Sbjct  168   CCTGGACTACCGTATGGACAGATGGACTTACTAGTCTTGATCGATATAAAGGTCGATGCT  227

Query  254   ATAATATTGAACCTGTTGCTGGAGAAGATAATCAATTCATAGCTTATGTAGCCTATCCTT  313
             ||||||||||||||||||||||||||||||||||||||||||||||||||||||||||||
Sbjct  228   ATAATATTGAACCTGTTGCTGGAGAAGATAATCAATTCATAGCTTATGTAGCCTATCCTT  287

Query  314   TAGATCTTTTTGAAGAAGGTTCTGTTACCAATCTTTTTACTTCAATTGTTGGTAATGTTT  373
             ||||||||||||||||||||||||||||||||||||||||||||||||||||||||||||
Sbjct  288   TAGATCTTTTTGAAGAAGGTTCTGTTACCAATCTTTTTACTTCAATTGTTGGTAATGTTT  347

Query  374   TCGGCTTCAAAGCTCTACGTGCTTTACGTTTAGAAGATTTAAGAATTCCTCCTGCTTACT  433
             ||||||||||||||||||||||||||||||||||||||||||||||||||||||||||||
Sbjct  348   TCGGCTTCAAAGCTCTACGTGCTTTACGTTTAGAAGATTTAAGAATTCCTCCTGCTTACT  407

Query  434   CCAAAACTTTTATAGGACCACCCCACGG-ATCCAGGTTGAAAGAGATAAGTTAAACAAAT  492
             |||||||||||||||||||||||||||| |||||||||||||||||||||||||||||||
Sbjct  408   CCAAAACTTTTATAGGACCACCCCACGGTATCCAGGTTGAAAGAGATAAGTTAAACAAAT  467

Query  493   ATGGTCGTCCTTTATTAGGTTGTACAATTAAACCAAAATTGGGACTATCTGCTAAAAACT  552
             ||||||||||||||||||||||||||||||||||||||||||||||||||||||||||||
Sbjct  468   ATGGTCGTCCTTTATTAGGTTGTACAATTAAACCAAAATTGGGACTATCTGCTAAAAACT  527

Query  553   ATGGTAGGGCTGTTTATGAATGTCTTCGTGGGGGGACTTGATTTCACCAAAGATGATGAG  612
             |||||||||||||||||||||||||||||||  |||||||||||||||||||||||||||
Sbjct  528   ATGGTAGGGCTGTTTATGAATGTCTTCGTGGT-GGACTTGATTTCACCAAAGATGATGAG  586

Query  613   AATGGTAAACTCTCAACCCCTTTATGCGTTGGAGAGATCGATTCTTATTTGTAGC  667
             ||| |||||||||||| ||||||||||||||||||||||| |||||||| |||||
Sbjct  587   AAT-GTAAACTCTCAA-CCCTTTATGCGTTGGAGAGATCGTTTCTTATTCGTAGC  639
```

BLAST is a registered trademark of the National Library of Medicine

Support center Mailing list YouTube

- National Library Of Medicine
- National Institutes Of Health
- U.S. Department of Health & Human Services
- USA.gov

NCBI

National Center for Biotechnology Information, U.S. National Library of Medicine 8600 Rockville Pike, Bethesda MD, 20894 USA

Policies and Guidelines | Contact

HHS Vulnerability Disclosure
