# Supplementary material for: A Multi-Pronged Technique for Identifying Equisetum palustre and Equisetum arvense—Combining HPTLC, HPLC-ESI-MS/MS and Optimized DNA Barcoding Techniques
Source: Plants (Basel). 2022 Sep 28;11(19):2562. doi: 10.3390/plants11192562 (PMC9573248; doi:10.3390/plants11192562)
Supplement: Supplementary file 1 [file plants-11-02562-s001.zip › plants-1932149 supplementary/Horsetail Supplementary Figures/Figure S28 (A2, 74323210721-1).pdf]

BLAST Results

Questions/comments

Job title: Nucleotide Sequence

**RID**	J1CSNGE3013 (Expires on 09-15 00:43 am)
**Query ID**	lcl|Query_8089
**Description**	None
**Molecule type**	dna
**Query Length**	677
**Database Name**	nt
**Description**	Nucleotide collection (nt)
**Program**	BLASTN 2.13.0+

Graphic Summary

Distribution of the top 100 Blast Hits on 100 subject sequences

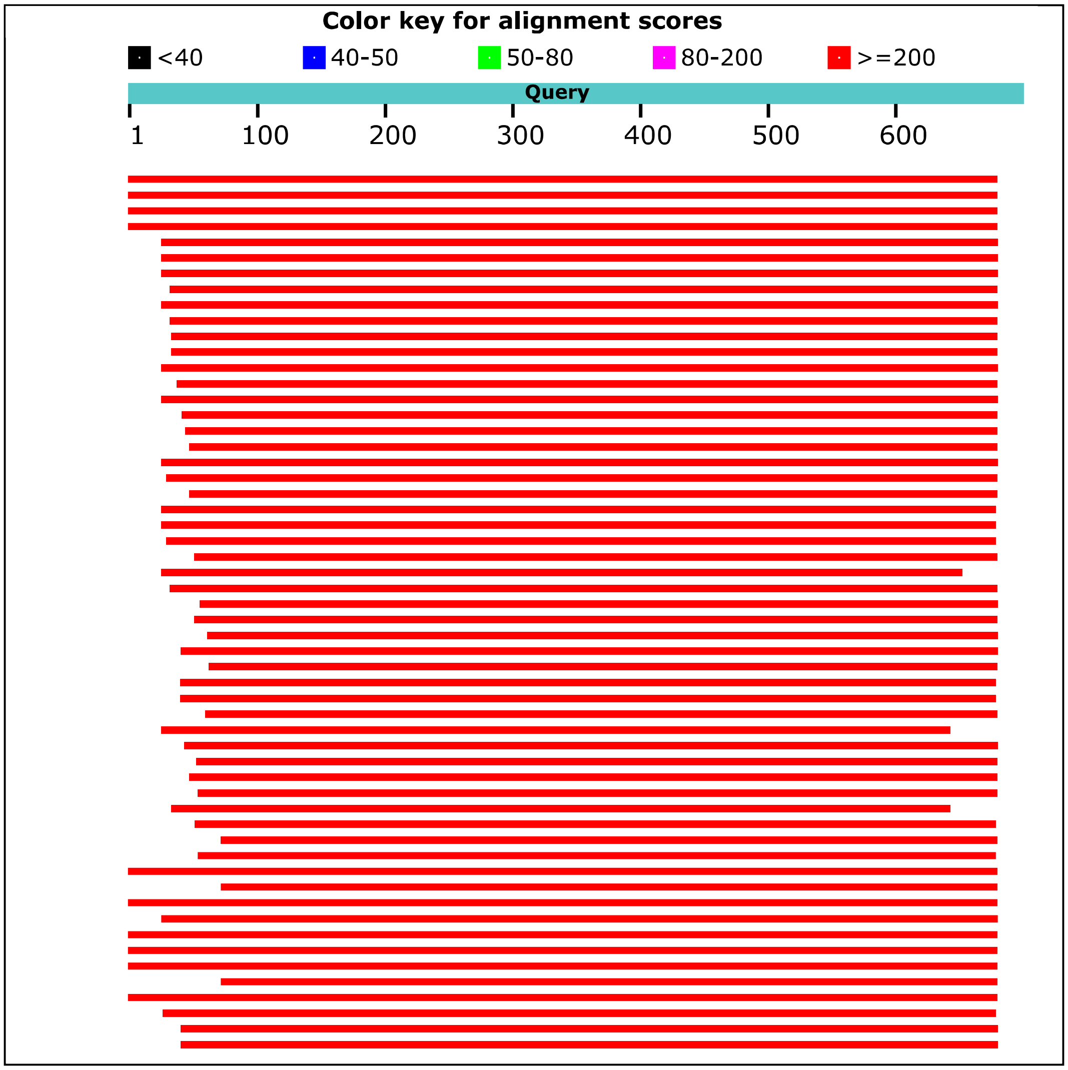

Descriptions

Sequences producing significant alignments:

Description	Max Score	Total Score	Query Cover	E value	Per. Ident	Accession
Equisetum arvense chloroplast, complete genome	1205	1205	98%	0.0	99.10%	GU191334.1
Equisetum arvense chloroplast ribulosebisphosphate carboxylase (rbcL) gene, complete cds	1205	1205	98%	0.0	99.10%	L11053.1
Equisetum arvense plastid, complete genome	1199	1199	98%	0.0	98.96%	JN968380.1
Equisetum palustre chloroplast, partial genome	1182	1182	98%	0.0	98.51%	MT984458.1
Equisetum environmental sample isolate EDNA16-0043490 ribulose-1,5-bisphosphate carboxylase/oxygenase large subunit (rbcL) gene, partial cds; chloroplast	1168	1168	95%	0.0	99.38%	MT197555.1
Equisetum fluviatile ribulose-1,5-bisphosphate carboxylase/oxygenase large subunit (rbcL) gene, partial cds; chloroplast gene for chloroplast product	1168	1168	95%	0.0	99.38%	AY226142.1
Equisetum arvense ribulose-1,5-bisphosphate carboxylase/oxygenase large subunit (rbcL) gene, partial cds; chloroplast gene for chloroplast product	1168	1168	95%	0.0	99.38%	AY226140.1
Equisetum arvense subsp. boreale isolate 41074 ribulose-1,5-bisphosphate carboxylase/oxygenase large subunit (rbcL) gene, partial cds; plastid	1162	1162	94%	0.0	99.53%	MH750160.1
Equisetum environmental sample isolate EDNA16-0043491b ribulose-1,5-bisphosphate carboxylase/oxygenase large subunit (rbcL) gene, partial cds; chloroplast	1162	1162	95%	0.0	99.22%	MT197548.1
Equisetum x dycei isolate 26083 ribulose-1,5-bisphosphate carboxylase/oxygenase large subunit (rbcL) gene, partial cds; plastid	1160	1160	94%	0.0	99.53%	MH750220.1
Equisetum fluviatile isolate 41076 ribulose-1,5-bisphosphate carboxylase/oxygenase large subunit (rbcL) gene, partial cds; plastid	1158	1158	93%	0.0	99.53%	MH750169.1
Equisetum fluviatile isolate 41075 ribulose-1,5-bisphosphate carboxylase/oxygenase large subunit (rbcL) gene, partial cds; plastid	1158	1158	93%	0.0	99.53%	MH750168.1
Equisetum diffusum ribulose-1,5-bisphosphate carboxylase/oxygenase large subunit (rbcL) gene, partial cds; chloroplast gene for chloroplast product	1157	1157	95%	0.0	99.07%	AY226141.1
Equisetum arvense subsp. boreale isolate 41073 ribulose-1,5-bisphosphate carboxylase/oxygenase large subunit (rbcL) gene, partial cds; plastid	1151	1151	93%	0.0	99.53%	MH750159.1

Description	Max Score	Total Score	Query Cover	E value	Per. Ident	Accession
Equisetum palustre ribulose-1,5-bisphosphate carboxylase/oxygenase large subunit (rbcL) gene, partial cds; chloroplast gene for chloroplast product	1146	1146	95%	0.0	98.76%	AY226138.1
Equisetum arvense subsp. arvense isolate 41071 ribulose-1,5-bisphosphate carboxylase/oxygenase large subunit (rbcL) gene, partial cds; plastid	1144	1144	92%	0.0	99.52%	MH750158.1
Equisetum x litorale isolate 41084 ribulose-1,5-bisphosphate carboxylase/oxygenase large subunit (rbcL) gene, partial cds; plastid	1138	1138	92%	0.0	99.52%	MH750222.1
Equisetum arvense subsp. arvense isolate 26085 ribulose-1,5-bisphosphate carboxylase/oxygenase large subunit (rbcL) gene, partial cds; plastid	1138	1138	91%	0.0	99.68%	MH750156.1
Equisetum telmateia subsp. braunii ribulose-1,5-bisphosphate carboxylase/oxygenase large subunit (rbcL) gene, partial cds; chloroplast gene for chloroplast product	1134	1134	95%	0.0	98.45%	AY226135.1
Equisetum telmateia subsp. telmateia isolate 11642 ribulose-1,5-bisphosphate carboxylase/oxygenase large subunit (rbcL) gene, partial cds; plastid	1133	1133	94%	0.0	98.59%	MH750210.1
Equisetum diffusum isolate 40689 ribulose-1,5-bisphosphate carboxylase/oxygenase large subunit (rbcL) gene, partial cds; plastid	1133	1133	91%	0.0	99.52%	MH750166.1
Equisetum pratense ribulose-1,5-bisphosphate carboxylase/oxygenase large subunit (rbcL) gene, partial cds; chloroplast gene for chloroplast product	1133	1133	94%	0.0	98.44%	AY226137.1
Equisetum sylvaticum ribulose-1,5-bisphosphate carboxylase/oxygenase large subunit (rbcL) gene, partial cds; chloroplast gene for chloroplast product	1133	1133	94%	0.0	98.44%	AY226136.1
Equisetum sylvaticum isolate 11640 ribulose-1,5-bisphosphate carboxylase/oxygenase large subunit (rbcL) gene, partial cds; plastid	1131	1131	94%	0.0	98.59%	MH750204.1
Equisetum arvense subsp. arvense isolate 26084 ribulose-1,5-bisphosphate carboxylase/oxygenase large subunit (rbcL) gene, partial cds; plastid	1131	1131	91%	0.0	99.68%	MH750155.1
Equisetum arvense voucher JAG288 (OAC) ribulose-1,5-bisphosphate carboxylase/oxygenase large subunit (rbcL) gene, partial cds; chloroplast	1129	1129	91%	0.0	99.68%	EU677106.1
Equisetum telmateia ribulose-1,5-bisphosphate carboxylase large subunit (rbcL) gene, partial cds; chloroplast gene for chloroplast product	1127	1127	94%	0.0	98.59%	AF313580.1
Equisetum arvense subsp. arvense isolate 40833 ribulose-1,5-bisphosphate carboxylase/oxygenase large subunit (rbcL) gene, partial cds; plastid	1123	1123	90%	0.0	99.67%	MH750157.1
Equisetum diffusum isolate 40804 ribulose-1,5-bisphosphate carboxylase/oxygenase large subunit (rbcL) gene, partial cds; plastid	1120	1120	91%	0.0	99.35%	MH750167.1

Description	Max Score	Total Score	Query Cover	E value	Per. Ident	Accession
Equisetum fluviatile ribulose-1,5-bisphosphate carboxylase/oxygenase large subunit (rbcL) gene, partial cds; chloroplast	1114	1114	89%	0.0	99.67%	DQ463101.1
Equisetum telmateia subsp. braunii isolate 40828 ribulose-1,5-bisphosphate carboxylase/oxygenase large subunit (rbcL) gene, partial cds; plastid	1112	1112	92%	0.0	98.57%	MH750207.1
Equisetum arvense x Equisetum telmateia subsp. braunii isolate 40834 ribulose-1,5-bisphosphate carboxylase/oxygenase large subunit (rbcL) gene, partial cds; plastid	1112	1112	89%	0.0	99.67%	MH750161.1
Equisetum sylvaticum isolate 41081 ribulose-1,5-bisphosphate carboxylase/oxygenase large subunit (rbcL) gene, partial cds; plastid	1110	1110	92%	0.0	98.57%	MH750205.1
Equisetum pratense isolate 41087 ribulose-1,5-bisphosphate carboxylase/oxygenase large subunit (rbcL) gene, partial cds; plastid	1110	1110	92%	0.0	98.57%	MH750195.1
Equisetum arvense ribulose-1,5-bisphosphate carboxylase/oxygenase large subunit (rbcL) gene, partial cds; chloroplast	1110	1110	90%	0.0	99.51%	KM218345.1
Equisetum arvense voucher AP400 (OAC) ribulose-1,5-bisphosphate carboxylase/oxygenase large subunit (rbcL) gene, partial cds; chloroplast	1110	1110	89%	0.0	99.67%	EU677105.1
Equisetum x fontqueri isolate 26093 ribulose-1,5-bisphosphate carboxylase/oxygenase large subunit (rbcL) gene, partial cds; plastid	1109	1109	92%	0.0	98.56%	MH750221.1
Equisetum palustre isolate 17671 ribulose-1,5-bisphosphate carboxylase/oxygenase large subunit (rbcL) gene, partial cds; plastid	1107	1107	91%	0.0	99.03%	MH750191.1
Equisetum telmateia subsp. braunii isolate 40832 ribulose-1,5-bisphosphate carboxylase/oxygenase large subunit (rbcL) gene, partial cds; plastid	1105	1105	91%	0.0	98.71%	MH750208.1
Equisetum palustre isolate 39349 ribulose-1,5-bisphosphate carboxylase/oxygenase large subunit (rbcL) gene, partial cds; plastid	1105	1105	90%	0.0	99.03%	MH750192.1
Equisetum pratense voucher AP451 ribulose-1,5-bisphosphate carboxylase/oxygenase large subunit (rbcL) gene, partial cds; chloroplast	1101	1101	88%	0.0	99.83%	HQ590083.1
Equisetum pratense isolate 41086 ribulose-1,5-bisphosphate carboxylase/oxygenase large subunit (rbcL) gene, partial cds; plastid	1096	1096	91%	0.0	98.70%	MH750194.1
Equisetum fluviatile chloroplast rbcL gene for ribulose-1,5-bisphosphate carboxylase/oxygenase large subunit, partial cds, specimen_voucher: TNS:776384	1094	1094	88%	0.0	99.67%	AB574684.1

Description	Max Score	Total Score	Query Cover	E value	Per. Ident	Accession
Equisetum pratense isolate 39348 ribulose-1,5-bisphosphate carboxylase/oxygenase large subunit (rbcL) gene, partial cds; plastid	1092	1092	90%	0.0	98.70%	MH750193.1
Equisetum xylochaetum chloroplast, complete genome	1088	1088	98%	0.0	95.97%	MW282958.1
Equisetum arvense chloroplast rbcL gene for ribulose-1,5-bisphosphate carboxylase/oxygenase large subunit, partial cds, specimen_voucher: TNS:763618	1088	1088	88%	0.0	99.50%	AB574683.1
Equisetum hyemale chloroplast, complete genome	1083	1083	98%	0.0	95.82%	KC117177.1
Equisetum scirpoides ribulose-1,5-bisphosphate carboxylase/oxygenase large subunit (rbcL) gene, partial cds; chloroplast gene for chloroplast product	1079	1079	95%	0.0	96.89%	AY226133.1
Equisetum ramosissimum isolate BPTPS123 voucher LISE:96444 chloroplast, complete genome	1072	1072	98%	0.0	95.52%	ON641349.1
Equisetum ramosissimum isolate BPTPS056 voucher LISE:96334 chloroplast, complete genome	1072	1072	98%	0.0	95.52%	NC_062377.1
Equisetum ramosissimum isolate BPTPS056 voucher LISE:96334 chloroplast, complete genome	1072	1072	98%	0.0	95.52%	OM691674.1
Equisetum palustre chloroplast rbcL gene for ribulose-1,5-bisphosphate carboxylase/oxygenase large subunit, partial cds, specimen_voucher: TNS:776981	1072	1072	88%	0.0	99.00%	AB574686.1
Equisetum hyemale ribulose-1,5-bisphosphate carboxylase/oxygenase large subunit (rbcL) gene, partial cds; chloroplast	1068	1068	98%	0.0	95.37%	DQ646001.1
Equisetum bogotense ribulose-1,5-bisphosphate carboxylase/oxygenase large subunit (rbcL) gene, partial cds; chloroplast gene for chloroplast product	1064	1064	94%	0.0	96.57%	AY226139.1
Equisetum scirpoides isolate 41089 ribulose-1,5-bisphosphate carboxylase/oxygenase large subunit (rbcL) gene, partial cds; plastid	1062	1062	92%	0.0	97.14%	MH750203.1
Equisetum scirpoides isolate 26090 ribulose-1,5-bisphosphate carboxylase/oxygenase large subunit (rbcL) gene, partial cds; plastid	1062	1062	92%	0.0	97.14%	MH750202.1
Equisetum komarovii isolate 40830 ribulose-1,5-bisphosphate carboxylase/oxygenase large subunit (rbcL) gene, partial cds; plastid	1062	1062	92%	0.0	97.14%	MH750182.1
Equisetum sylvaticum chloroplast rbcL gene for ribulose-1,5-bisphosphate carboxylase/oxygenase large subunit, partial cds, specimen_voucher: TNS:776974	1059	1059	88%	0.0	98.66%	AB574690.1

Description	Max Score	Total Score	Query Cover	E value	Per. Ident	Accession
Equisetum pratense chloroplast rbcL gene for ribulose-1,5-bisphosphate carboxylase/oxygenase large subunit, partial cds, specimen_voucher: TNS:1107843	1059	1059	88%	0.0	98.66%	AB574687.1
Equisetum giganteum isolate 40806 ribulose-1,5-bisphosphate carboxylase/oxygenase large subunit (rbcL) gene, partial cds; plastid	1051	1051	94%	0.0	96.26%	MH750172.1
Equisetum myriochaetum ribulose-1,5-bisphosphate carboxylase/oxygenase large subunit (rbcL) gene, partial cds; chloroplast gene for chloroplast product	1051	1051	95%	0.0	96.12%	AY226131.1
Equisetum giganteum ribulose-1,5-bisphosphate carboxylase/oxygenase large subunit (rbcL) gene, partial cds; chloroplast gene for chloroplast product	1051	1051	95%	0.0	96.12%	AY226127.1
Equisetum giganteum isolate 26087 ribulose-1,5-bisphosphate carboxylase/oxygenase large subunit (rbcL) gene, partial cds; plastid	1050	1050	94%	0.0	96.25%	MH750170.1
Equisetum sp. Schuettpelz 1643 ribulose-1,5-bisphosphate carboxylase/oxygenase large subunit (rbcL) gene, partial cds; chloroplast	1050	1050	94%	0.0	96.11%	MW620286.1
Equisetum x ferrissii ribulose-1,5-bisphosphate carboxylase/oxygenase large subunit (rbcL) gene, partial cds; chloroplast	1050	1050	94%	0.0	96.25%	EU352292.1
Equisetum laevigatum ribulose-1,5-bisphosphate carboxylase/oxygenase large subunit (rbcL) gene, partial cds; chloroplast gene for chloroplast product	1050	1050	94%	0.0	96.25%	AY226130.1
Equisetum scirpoides isolate 10933 ribulose-1,5-bisphosphate carboxylase/oxygenase large subunit (rbcL) gene, partial cds; plastid	1048	1048	91%	0.0	97.25%	MH750201.1
Equisetum x ferrissii ribulose-1,5-bisphosphate carboxylase large subunit (rbcL) gene, partial cds; chloroplast gene for chloroplast product	1048	1048	94%	0.0	96.24%	AF313579.1
Equisetum hyemale subsp. affine voucher OAC 94916 ribulose-1,5-bisphosphate carboxylase/oxygenase large subunit (rbcL) gene, partial cds; chloroplast	1046	1046	95%	0.0	95.96%	KF186500.1
Equisetum hyemale subsp. affine ribulose-1,5-bisphosphate carboxylase/oxygenase large subunit (rbcL) gene, partial cds; chloroplast gene for chloroplast product	1046	1046	95%	0.0	95.96%	AY226128.1
Equisetum ramosissimum voucher R825 ribulose-1,5-bisphosphate carboxylase/oxygenase large subunit (rbcl) gene, partial cds; chloroplast	1044	1044	95%	0.0	95.69%	KY656709.1
Equisetum arvense voucher TJD-508 ribulose-1,5-bisphosphate carboxylase/oxygenase large subunit (rbcL) gene, partial cds; chloroplast	1042	1042	84%	0.0	99.65%	KJ841301.1

Description	Max Score	Total Score	Query Cover	E value	Per. Ident	Accession
Equisetum arvense voucher TJD-503 ribulose-1,5-bisphosphate carboxylase/oxygenase large subunit (rbcL) gene, partial cds; chloroplast	1040	1040	84%	0.0	99.65%	KJ841302.1
Equisetum arvense voucher TJD-504 ribulose-1,5-bisphosphate carboxylase/oxygenase large subunit (rbcL) gene, partial cds; chloroplast	1038	1038	83%	0.0	99.65%	KJ841300.1
Equisetum myriochaetum isolate 40815 ribulose-1,5-bisphosphate carboxylase/oxygenase large subunit (rbcL) gene, partial cds; plastid	1037	1037	94%	0.0	96.08%	MH750185.1
Equisetum bogotense isolate 40800 ribulose-1,5-bisphosphate carboxylase/oxygenase large subunit (rbcL) gene, partial cds; plastid	1037	1037	91%	0.0	96.78%	MH750162.1
Equisetum ramosissimum subsp. debile voucher G.M. Plunkett 3642 (NYBG) ribulose-1,5-bisphosphate carboxylase/oxygenase large subunit (rbcL) gene, partial cds; chloroplast	1035	1035	95%	0.0	95.65%	MT657901.1
Equisetum ramosissimum subsp. debile ribulose-1,5-bisphosphate carboxylase/oxygenase large subunit (rbcL) gene, partial cds; chloroplast gene for chloroplast product	1035	1035	95%	0.0	95.65%	AY226132.1
Commelina diffusa voucher Q555 ribulose-1,5-bisphosphate carboxylase/oxygenase large subunit (rbcL) gene, partial cds; chloroplast	1031	1031	83%	0.0	99.47%	MH714073.1
Equisetum xylochaetum isolate 40614 ribulose-1,5-bisphosphate carboxylase/oxygenase large subunit (rbcL) gene, partial cds; plastid	1029	1029	92%	0.0	96.18%	MH750228.1
Equisetum myriochaetum isolate 41080 ribulose-1,5-bisphosphate carboxylase/oxygenase large subunit (rbcL) gene, partial cds; plastid	1029	1029	92%	0.0	96.18%	MH750190.1
Equisetum myriochaetum isolate 40826 ribulose-1,5-bisphosphate carboxylase/oxygenase large subunit (rbcL) gene, partial cds; plastid	1029	1029	92%	0.0	96.18%	MH750188.1
Equisetum x schaffneri isolate 40813 ribulose-1,5-bisphosphate carboxylase/oxygenase large subunit (rbcL) gene, partial cds; plastid	1027	1027	92%	0.0	96.18%	MH750224.1
Equisetum scirpoides voucher AP248 ribulose-1,5-bisphosphate carboxylase/oxygenase large subunit (rbcL) gene, partial cds; chloroplast	1027	1027	89%	0.0	97.20%	HQ590084.1
Equisetum giganteum isolate 40811 ribulose-1,5-bisphosphate carboxylase/oxygenase large subunit (rbcL) gene, partial cds; plastid	1026	1026	92%	0.0	96.17%	MH750176.1
Equisetum hyemale subsp. hyemale isolate 41088 ribulose-1,5-bisphosphate carboxylase/oxygenase large subunit (rbcL) gene, partial cds; plastid	1024	1024	92%	0.0	96.03%	MH750181.1

Description	Max Score	Total Score	Query Cover	E value	Per. Ident	Accession
Equisetum giganteum isolate 40807 ribulose-1,5-bisphosphate carboxylase/oxygenase large subunit (rbcL) gene, partial cds; plastid	1022	1022	91%	0.0	96.30%	MH750173.1
Equisetum ramosissimum subsp. debile isolate 36802 ribulose-1,5-bisphosphate carboxylase/oxygenase large subunit (rbcL) gene, partial cds; plastid	1020	1020	93%	0.0	95.87%	MH750199.1
Equisetum bogotense isolate 40801 ribulose-1,5-bisphosphate carboxylase/oxygenase large subunit (rbcL) gene, partial cds; plastid	1020	1020	90%	0.0	96.73%	MH750163.1
Equisetum myriochaetum isolate 40936 ribulose-1,5-bisphosphate carboxylase/oxygenase large subunit (rbcL) gene, partial cds; plastid	1018	1018	91%	0.0	96.29%	MH750189.1
Equisetum sylvaticum voucher TJD-502 ribulose-1,5-bisphosphate carboxylase/oxygenase large subunit (rbcL) gene, partial cds; chloroplast	1018	1018	84%	0.0	98.95%	KJ841305.1
Equisetum variegatum ribulose-1,5-bisphosphate carboxylase/oxygenase large subunit (rbcL) gene, partial cds; chloroplast gene for chloroplast product	1018	1018	95%	0.0	95.19%	AY226134.1
Equisetum hyemale subsp. affine isolate 40831 ribulose-1,5-bisphosphate carboxylase/oxygenase large subunit (rbcL) gene, partial cds; plastid	1016	1016	91%	0.0	96.14%	MH750177.1
Equisetum trachyodon isolate 41092 ribulose-1,5-bisphosphate carboxylase/oxygenase large subunit (rbcL) gene, partial cds; plastid	1013	1013	92%	0.0	95.85%	MH750227.1
Equisetum ramosissimum subsp. debile isolate 40829 ribulose-1,5-bisphosphate carboxylase/oxygenase large subunit (rbcL) gene, partial cds; plastid	1013	1013	92%	0.0	95.71%	MH750200.1
Equisetum myriochaetum isolate 26088 ribulose-1,5-bisphosphate carboxylase/oxygenase large subunit (rbcL) gene, partial cds; plastid	1011	1011	90%	0.0	96.27%	MH750184.1
Equisetum arvense voucher WAB_1079140221 ribulose-1,5-bisphosphate carboxylase/oxygenase large subunit (rbcL) gene, partial cds; chloroplast	1011	1011	81%	0.0	99.64%	KJ593388.1
Equisetum arvense subsp. alpestre voucher Gillespie_10109_CAN ribulose-1,5-bisphosphate carboxylase/oxygenase large subunit (rbcL) gene, partial cds; chloroplast	1011	1011	81%	0.0	99.64%	KC482713.1
Equisetum scirpoides chloroplast rbcL gene for ribulose-1,5-bisphosphate carboxylase/oxygenase large subunit, partial cds, specimen_voucher: TNS:768168	1011	1011	88%	0.0	97.16%	AB574689.1
Equisetum arvense ribulose-1,5-bisphosphate carboxylase/oxygenase large subunit (rbcL) gene, partial cds; chloroplast	1009	1009	81%	0.0	99.64%	MZ359289.1

Alignments

Equisetum arvense chloroplast, complete genome

Sequence ID: **GU191334.1** Length: 133309 Number of Matches: 1

Range 1: 27302 to 27971

Score	Expect	Identities	Gaps	Strand	Frame
1205 bits(652)	0.0()	664/670(99%)	0/670(0%)	Plus/Minus	

Features:

```
Query  1      ATGTCACCACAAACAGAGACTAAAGCAGGGGTTGGATTTAAAGCTGGGGTTAAAGATTAT  60
              |||||||||||||| ||||| |||||||| ||||||||||||||||| ||||||||||||
Sbjct  27971  ATGTCACCACAAACGGAGACCAAAGCAGGTGTTGGATTTAAAGCTGGTGTTAAAGATTAT  27912

Query  61     CGATTGACTTATTTTACTCCAGATTATGAAACCAAAGATACCGATATTTTAGCAGCATTC  120
              ||||||||||||||||||||||||||||||||||||||||||||||||||||||||||||
Sbjct  27911  CGATTGACTTATTTTACTCCAGATTATGAAACCAAAGATACCGATATTTTAGCAGCATTC  27852

Query  121    CGTATGACTCCTCAACCGGGAGTACCACCGGAAGAAGCAGGAGCAGCCGTAGCTGCTGAA  180
              ||||||||||||||||||||||||||||||||||||||||||||||||||||||||||||
Sbjct  27851  CGTATGACTCCTCAACCGGGAGTACCACCGGAAGAAGCAGGAGCAGCCGTAGCTGCTGAA  27792

Query  181    TCGTCCACGGGCACCTGGACTACCGTATGGACAGATGGACTTACTAGTCTTGATCGATAT  240
              ||||||||||||||||||||||||||||||||||||||||||||||||||||||||||||
Sbjct  27791  TCGTCCACGGGCACCTGGACTACCGTATGGACAGATGGACTTACTAGTCTTGATCGATAT  27732

Query  241    AAAGGTCGATGCTATAATATTGAACCTGTTGCTGGAGAAGATAATCAATTCATAGCTTAT  300
              ||||||||||||||||||||||||||||||||||||||||||||||||||||||||||||
Sbjct  27731  AAAGGTCGATGCTATAATATTGAACCTGTTGCTGGAGAAGATAATCAATTCATAGCTTAT  27672

Query  301    GTAGCCTACCCTTTAGATCTTTTTGAAGAAGGTTCTGTTACCAATCTTTTTACTTCAATT  360
              ||||||||||||||||||||||||||||||||||||||||||||||||||||||||||||
Sbjct  27671  GTAGCCTACCCTTTAGATCTTTTTGAAGAAGGTTCTGTTACCAATCTTTTTACTTCAATT  27612

Query  361    GTTGGTAATGTTTTCGGCTTCAAAGCTCTACGTGCTTTACGTTTAGAAGATTTAAGAATT  420
              ||||||||||||||||||||||||||||||||||||||||||||||||||||||||||||
Sbjct  27611  GTTGGTAATGTTTTCGGCTTCAAAGCTCTACGTGCTTTACGTTTAGAAGATTTAAGAATT  27552

Query  421    CCTCCTGCTTACTCCAAAACTTTTATAGGACCACCCCACGGTATCCAGGTTGAAAGAGAT  480
              ||||||||||||||||||||||||||||||||||||||||||||||||||||||||||||
Sbjct  27551  CCTCCTGCTTACTCCAAAACTTTTATAGGACCACCCCACGGTATCCAGGTTGAAAGAGAT  27492

Query  481    AAGTTAAACAAATACGGTCGTCCTTTATTAGGTTGTACAATTAAACCAAAATTGGGACTA  540
              ||||||||||||||||||||||||||||||||||||||||||||||||||||||||||||
Sbjct  27491  AAGTTAAACAAATACGGTCGTCCTTTATTAGGTTGTACAATTAAACCAAAATTGGGACTA  27432

Query  541    TCTGCTAAAAACTATGGTAGAGCTGTTTATGAATGTCTTCGTGGTGGGCTTGATTTCACC  600
              ||||||||||||||||||||||||||||||||||||||||||||||||||||||||||||
Sbjct  27431  TCTGCTAAAAACTATGGTAGAGCTGTTTATGAATGTCTTCGTGGTGGGCTTGATTTCACC  27372

Query  601    AAAGATGATGAGAATGTAAACTCTCAACCCTTTATGCGTTGGAGAGATCGATTCTTATTT  660
              |||||||||||||||||||||||||||||||||||||||||||||||||| |||||||| 
Sbjct  27371  AAAGATGATGAGAATGTAAACTCTCAACCCTTTATGCGTTGGAGAGATCGTTTCTTATTC  27312

Query  661    GTAGCAGAAG  670
              ||||||||||
Sbjct  27311  GTAGCAGAAG  27302
```

Equisetum arvense chloroplast ribulosebisphosphate carboxylase (rbcL) gene, complete cds

Sequence ID: **L11053.1** Length: 1587 Number of Matches: 1

Range 1: 73 to 742

Score	Expect	Identities	Gaps	Strand	Frame
1205 bits(652)	0.0()	664/670(99%)	0/670(0%)	Plus/Plus	

Features:

```
Query  1    ATGTCACCACAAACAGAGACTAAAGCAGGGGTTGGATTTAAAGCTGGGGTTAAAGATTAT  60
            |||||||||||||| ||||| |||||||| ||||||||||||||||| ||||||||||||
Sbjct  73   ATGTCACCACAAACGGAGACCAAAGCAGGTGTTGGATTTAAAGCTGGTGTTAAAGATTAT  132

Query  61   CGATTGACTTATTTTACTCCAGATTATGAAACCAAAGATACCGATATTTTAGCAGCATTC  120
            ||||||||||||||||||||||||||||||||||||||||||||||||||||||||||||
Sbjct  133  CGATTGACTTATTTTACTCCAGATTATGAAACCAAAGATACCGATATTTTAGCAGCATTC  192

Query  121  CGTATGACTCCTCAACCGGGAGTACCACCGGAAGAAGCAGGAGCAGCCGTAGCTGCTGAA  180
            ||||||||||||||||||||||||||||||||||||||||||||||||||||||||||||
Sbjct  193  CGTATGACTCCTCAACCGGGAGTACCACCGGAAGAAGCAGGAGCAGCCGTAGCTGCTGAA  252

Query  181  TCGTCCACGGGCACCTGGACTACCGTATGGACAGATGGACTTACTAGTCTTGATCGATAT  240
            ............................................................
```

```
                ||||||||||||||||||||||||||||||||||||||||||||||||||||||||||||
Sbjct  253      TCGTCCACGGGCACCTGGACTACCGTATGGACAGATGGACTTACTAGTCTTGATCGATAT  312

Query  241      AAAGGTCGATGCTATAATATTGAACCTGTTGCTGGAGAAGATAATCAATTCATAGCTTAT  300
                ||||||||||||||||||||||||||||||||||||||||||||||||||||||||||||
Sbjct  313      AAAGGTCGATGCTATAATATTGAACCTGTTGCTGGAGAAGATAATCAATTCATAGCTTAT  372

Query  301      GTAGCCTACCCTTTAGATCTTTTTGAAGAAGGTTCTGTTACCAATCTTTTTACTTCAATT  360
                ||||||||||||||||||||||||||||||||||||||||||||||||||||||||||||
Sbjct  373      GTAGCCTACCCTTTAGATCTTTTTGAAGAAGGTTCTGTTACCAATCTTTTTACTTCAATT  432

Query  361      GTTGGTAATGTTTTCGGCTTCAAAGCTCTACGTGCTTTACGTTTAGAAGATTTAAGAATT  420
                ||||||||||||||||||||||||||||||||||||||||||||||||||||||||||||
Sbjct  433      GTTGGTAATGTTTTCGGCTTCAAAGCTCTACGTGCTTTACGTTTAGAAGATTTAAGAATT  492

Query  421      CCTCCTGCTTACTCCAAAACTTTTATAGGACCACCCCACGGTATCCAGGTTGAAAGAGAT  480
                ||||||||||||||||||||||||||||||||||||||||||||||||||||||||||||
Sbjct  493      CCTCCTGCTTACTCCAAAACTTTTATAGGACCACCCCACGGTATCCAGGTTGAAAGAGAT  552

Query  481      AAGTTAAACAAATACGGTCGTCCTTTATTAGGTTGTACAATTAAACCAAAATTGGGACTA  540
                ||||||||||||||||||||||||||||||||||||||||||||||||||||||||||||
Sbjct  553      AAGTTAAACAAATACGGTCGTCCTTTATTAGGTTGTACAATTAAACCAAAATTGGGACTA  612

Query  541      TCTGCTAAAAACTATGGTAGAGCTGTTTATGAATGTCTTCGTGGTGGGCTTGATTTCACC  600
                ||||||||||||||||||||||||||||||||||||||||||||||||||||||||||||
Sbjct  613      TCTGCTAAAAACTATGGTAGAGCTGTTTATGAATGTCTTCGTGGTGGGCTTGATTTCACC  672

Query  601      AAAGATGATGAGAATGTAAACTCTCAACCCTTTATGCGTTGGAGAGATCGATTCTTATTT  660
                |||||||||||||||||||||||||||||||||||||||||||||||||| ||||||||
Sbjct  673      AAAGATGATGAGAATGTAAACTCTCAACCCTTTATGCGTTGGAGAGATCGTTTCTTATTC  732

Query  661      GTAGCAGAAG  670
                ||||||||||
Sbjct  733      GTAGCAGAAG  742
```

Equisetum arvense plastid, complete genome

Sequence ID: **JN968380.1** Length: 132726 Number of Matches: 1

Range 1: 64958 to 65627

**Score**	**Expect**	**Identities**	**Gaps**	**Strand**	**Frame**
1199 bits(649)	0.0()	663/670(99%)	0/670(0%)	Plus/Plus	

Features:

```
Query  1        ATGTCACCACAAACAGAGACTAAAGCAGGGGTTGGATTTAAAGCTGGGGTTAAAGATTAT  60
                |||||||||||||| ||||| |||||||| ||||||||||||||||| ||||||||||||
Sbjct  64958    ATGTCACCACAAACGGAGACCAAAGCAGGTGTTGGATTTAAAGCTGGTGTTAAAGATTAT  65017

Query  61       CGATTGACTTATTTTACTCCAGATTATGAAACCAAAGATACCGATATTTTAGCAGCATTC  120
                ||||||||||||||||||||||||||||||||||||||||||||||||||||||||||||
Sbjct  65018    CGATTGACTTATTTTACTCCAGATTATGAAACCAAAGATACCGATATTTTAGCAGCATTC  65077

Query  121      CGTATGACTCCTCAACCGGGAGTACCACCGGAAGAAGCAGGAGCAGCCGTAGCTGCTGAA  180
                ||||||||||||||||||||||||||||||||||||||||||||||||||||||||||||
Sbjct  65078    CGTATGACTCCTCAACCGGGAGTACCACCGGAAGAAGCAGGAGCAGCCGTAGCTGCTGAA  65137

Query  181      TCGTCCACGGGCACCTGGACTACCGTATGGACAGATGGACTTACTAGTCTTGATCGATAT  240
                ||||||||||||||||||||||||||||||||||||||||||||||||||||||||||||
Sbjct  65138    TCGTCCACGGGCACCTGGACTACCGTATGGACAGATGGACTTACTAGTCTTGATCGATAT  65197

Query  241      AAAGGTCGATGCTATAATATTGAACCTGTTGCTGGAGAAGATAATCAATTCATAGCTTAT  300
                ||||||||||||||||||||||||||||||||||||||||||||||||||||||||||||
Sbjct  65198    AAAGGTCGATGCTATAATATTGAACCTGTTGCTGGAGAAGATAATCAATTCATAGCTTAT  65257

Query  301      GTAGCCTACCCTTTAGATCTTTTTGAAGAAGGTTCTGTTACCAATCTTTTTACTTCAATT  360
                ||||||||||||||||||||||||||||||||||||||||||||||||||||||||||||
Sbjct  65258    GTAGCCTACCCTTTAGATCTTTTTGAAGAAGGTTCTGTTACCAATCTTTTTACTTCAATT  65317

Query  361      GTTGGTAATGTTTTCGGCTTCAAAGCTCTACGTGCTTTACGTTTAGAAGATTTAAGAATT  420
                ||||||||||||||||| ||||||||||||||||||||||||||||||||||||||||||
Sbjct  65318    GTTGGTAATGTTTTCGGATTCAAAGCTCTACGTGCTTTACGTTTAGAAGATTTAAGAATT  65377

Query  421      CCTCCTGCTTACTCCAAAACTTTTATAGGACCACCCCACGGTATCCAGGTTGAAAGAGAT  480
                ||||||||||||||||||||||||||||||||||||||||||||||||||||||||||||
Sbjct  65378    CCTCCTGCTTACTCCAAAACTTTTATAGGACCACCCCACGGTATCCAGGTTGAAAGAGAT  65437

Query  481      AAGTTAAACAAATACGGTCGTCCTTTATTAGGTTGTACAATTAAACCAAAATTGGGACTA  540
                ||||||||||||||||||||||||||||||||||||||||||||||||||||||||||||
Sbjct  65438    AAGTTAAACAAATACGGTCGTCCTTTATTAGGTTGTACAATTAAACCAAAATTGGGACTA  65497

Query  541      TCTGCTAAAAACTATGGTAGAGCTGTTTATGAATGTCTTCGTGGTGGGCTTGATTTCACC  600
                ||||||||||||||||||||||||||||||||||||||||||||||||||||||||||||
Sbjct  65498    TCTGCTAAAAACTATGGTAGAGCTGTTTATGAATGTCTTCGTGGTGGGCTTGATTTCACC  65557

Query  601      AAAGATGATGAGAATGTAAACTCTCAACCCTTTATGCGTTGGAGAGATCGATTCTTATTT  660
                |||||||||||||||||||||||||||||||||||||||||||||||||| ||||||||
Sbjct  65558    AAAGATGATGAGAATGTAAACTCTCAACCCTTTATGCGTTGGAGAGATCGTTTCTTATTC  65617
```

```
Query  661    GTAGCAGAAG  670
              ||||||||||
Sbjct  65618  GTAGCAGAAG  65627
```

Equisetum palustre chloroplast, partial genome

Sequence ID: **MT984458.1** Length: 5501 Number of Matches: 1

Range 1: 2213 to 2882

Score	Expect	Identities	Gaps	Strand	Frame
1182 bits(640)	0.0()	660/670(99%)	0/670(0%)	Plus/Plus	

Features:

```
Query  1     ATGTCACCACAAACAGAGACTAAAGCAGGGGTTGGATTTAAAGCTGGGGTTAAAGATTAT  60
             |||||||||||||| ||||| |||||||| ||||||||||||||||| ||||||||||||
Sbjct  2213  ATGTCACCACAAACGGAGACCAAAGCAGGTGTTGGATTTAAAGCTGGTGTTAAAGATTAT  2272

Query  61    CGATTGACTTATTTTACTCCAGATTATGAAACCAAAGATACCGATATTTTAGCAGCATTC  120
             ||||||||||||||||||||||||||||||||||||||||||||||||||||||||||||
Sbjct  2273  CGATTGACTTATTTTACTCCAGATTATGAAACCAAAGATACCGATATTTTAGCAGCATTC  2332

Query  121   CGTATGACTCCTCAACCGGGAGTACCACCGGAAGAAGCAGGAGCAGCCGTAGCTGCTGAA  180
             ||||||||||||||||||||||||||||||||||||||||||||||||||||||||||||
Sbjct  2333  CGTATGACTCCTCAACCGGGAGTACCACCGGAAGAAGCAGGAGCAGCCGTAGCTGCTGAA  2392

Query  181   TCGTCCACGGGCACCTGGACTACCGTATGGACAGATGGACTTACTAGTCTTGATCGATAT  240
             ||||||||||||||||||||||||||||||||||||||||||||||||||||||||||||
Sbjct  2393  TCGTCCACGGGCACCTGGACTACCGTATGGACAGATGGACTTACTAGTCTTGATCGATAT  2452

Query  241   AAAGGTCGATGCTATAATATTGAACCTGTTGCTGGAGAAGATAATCAATTCATAGCTTAT  300
             ||||||||||||||||||||||||||||||||||||||||||||||||||||||||||||
Sbjct  2453  AAAGGTCGATGCTATAATATTGAACCTGTTGCTGGAGAAGATAATCAATTCATAGCTTAT  2512

Query  301   GTAGCCTACCCTTTAGATCTTTTTGAAGAAGGTTCTGTTACCAATCTTTTTACTTCAATT  360
             |||||||| |||||||||||||||||||||||||||||||||||||||||||||||||||
Sbjct  2513  GTAGCCTATCCTTTAGATCTTTTTGAAGAAGGTTCTGTTACCAATCTTTTTACTTCAATT  2572

Query  361   GTTGGTAATGTTTTCGGCTTCAAAGCTCTACGTGCTTTACGTTTAGAAGATTTAAGAATT  420
             ||||||||||||||||||||||||||||||||||||||||||||||||||||||||||||
Sbjct  2573  GTTGGTAATGTTTTCGGCTTCAAAGCTCTACGTGCTTTACGTTTAGAAGATTTAAGAATT  2632

Query  421   CCTCCTGCTTACTCCAAAACTTTTATAGGACCACCCCACGGTATCCAGGTTGAAAGAGAT  480
             ||||||||||||||||||||||||||||||||||||||||||||||||||||||||||||
Sbjct  2633  CCTCCTGCTTACTCCAAAACTTTTATAGGACCACCCCACGGTATCCAGGTTGAAAGAGAT  2692

Query  481   AAGTTAAACAAATACGGTCGTCCTTTATTAGGTTGTACAATTAAACCAAAATTGGGACTA  540
             |||||||||||||| |||||||||||||||||||||||||||||||||||||||||||||
Sbjct  2693  AAGTTAAACAAATATGGTCGTCCTTTATTAGGTTGTACAATTAAACCAAAATTGGGACTA  2752

Query  541   TCTGCTAAAAACTATGGTAGAGCTGTTTATGAATGTCTTCGTGGTGGGCTTGATTTCACC  600
             |||||||||||||||||||| |||||||||||||||||||||||||| ||||||||||||
Sbjct  2753  TCTGCTAAAAACTATGGTAGGGCTGTTTATGAATGTCTTCGTGGTGGACTTGATTTCACC  2812

Query  601   AAAGATGATGAGAATGTAAACTCTCAACCCTTTATGCGTTGGAGAGATCGATTCTTATTT  660
             |||||||||||||||||||||||||||||||||||||||||||||||||| |||||||| 
Sbjct  2813  AAAGATGATGAGAATGTAAACTCTCAACCCTTTATGCGTTGGAGAGATCGTTTCTTATTC  2872

Query  661   GTAGCAGAAG  670
             ||||||||||
Sbjct  2873  GTAGCAGAAG  2882
```

Equisetum environmental sample isolate EDNA16-0043490 ribulose-1,5-bisphosphate carboxylase/oxygenase large subunit (rbcL) gene, partial cds; chloroplast

Sequence ID: **MT197555.1** Length: 697 Number of Matches: 1

Range 1: 1 to 644

Score	Expect	Identities	Gaps	Strand	Frame
1168 bits(632)	0.0()	640/644(99%)	0/644(0%)	Plus/Plus	

Features:

```
Query  27   AGGGGTTGGATTTAAAGCTGGGGTTAAAGATTATCGATTGACTTATTTTACTCCAGATTA  86
            ||| ||||||||||||||||| ||||||||||||||||||||||||||||||||||||||
Sbjct  1    AGGTGTTGGATTTAAAGCTGGTGTTAAAGATTATCGATTGACTTATTTTACTCCAGATTA  60

Query  87   TGAAACCAAAGATACCGATATTTTAGCAGCATTCCGTATGACTCCTCAACCGGGAGTACC  146
            ||||||||||||||||||||||||||||||||||||||||||||||||||||||||||||
Sbjct  61   TGAAACCAAAGATACCGATATTTTAGCAGCATTCCGTATGACTCCTCAACCGGGAGTACC  120
```

```
Query  147   ACCGGAAGAAGCAGGAGCAGCCGTAGCTGCTGAATCGTCCACGGGCACCTGGACTACCGT  206
             ||||||||||||||||||||||||||||||||||||||||||||||||||||||||||||
Sbjct  121   ACCGGAAGAAGCAGGAGCAGCCGTAGCTGCTGAATCGTCCACGGGCACCTGGACTACCGT  180

Query  207   ATGGACAGATGGACTTACTAGTCTTGATCGATATAAAGGTCGATGCTATAATATTGAACC  266
             ||||||||||||||||||||||||||||||||||||||||||||||||||||||||||||
Sbjct  181   ATGGACAGATGGACTTACTAGTCTTGATCGATATAAAGGTCGATGCTATAATATTGAACC  240

Query  267   TGTTGCTGGAGAAGATAATCAATTCATAGCTTATGTAGCCTACCCTTTAGATCTTTTTGA  326
             ||||||||||||||||||||||||||||||||||||||||||||||||||||||||||||
Sbjct  241   TGTTGCTGGAGAAGATAATCAATTCATAGCTTATGTAGCCTACCCTTTAGATCTTTTTGA  300

Query  327   AGAAGGTTCTGTTACCAATCTTTTTACTTCAATTGTTGGTAATGTTTTCGGCTTCAAAGC  386
             ||||||||||||||||||||||||||||||||||||||||||||||||||||||||||||
Sbjct  301   AGAAGGTTCTGTTACCAATCTTTTTACTTCAATTGTTGGTAATGTTTTCGGCTTCAAAGC  360

Query  387   TCTACGTGCTTTACGTTTAGAAGATTTAAGAATTCCTCCTGCTTACTCCAAAACTTTTAT  446
             ||||||||||||||||||||||||||||||||||||||||||||||||||||||||||||
Sbjct  361   TCTACGTGCTTTACGTTTAGAAGATTTAAGAATTCCTCCTGCTTACTCCAAAACTTTTAT  420

Query  447   AGGACCACCCCACGGTATCCAGGTTGAAAGAGATAAGTTAAACAAATACGGTCGTCCTTT  506
             ||||||||||||||||||||||||||||||||||||||||||||||||||||||||||||
Sbjct  421   AGGACCACCCCACGGTATCCAGGTTGAAAGAGATAAGTTAAACAAATACGGTCGTCCTTT  480

Query  507   ATTAGGTTGTACAATTAAACCAAAATTGGGACTATCTGCTAAAAACTATGGTAGAGCTGT  566
             ||||||||||||||||||||||||||||||||||||||||||||||||||||||||||||
Sbjct  481   ATTAGGTTGTACAATTAAACCAAAATTGGGACTATCTGCTAAAAACTATGGTAGAGCTGT  540

Query  567   TTATGAATGTCTTCGTGGTGGGCTTGATTTCACCAAAGATGATGAGAATGTAAACTCTCA  626
             ||||||||||||||||||||||||||||||||||||||||||||||||||||||||||||
Sbjct  541   TTATGAATGTCTTCGTGGTGGGCTTGATTTCACCAAAGATGATGAGAATGTAAACTCTCA  600

Query  627   ACCCTTTATGCGTTGGAGAGATCGATTCTTATTTGTAGCAGAAG  670
             |||||||||||||||||||||||| |||||||| ||||||||||
Sbjct  601   ACCCTTTATGCGTTGGAGAGATCGTTTCTTATTCGTAGCAGAAG  644
```


BLAST is a registered trademark of the National Library of Medicine

Support center Mailing list YouTube

- National Library Of Medicine
- National Institutes Of Health
- U.S. Department of Health & Human Services
- USA.gov

NCBI

National Center for Biotechnology Information, U.S. National Library of Medicine 8600 Rockville Pike, Bethesda MD, 20894 USA

Policies and Guidelines | Contact

HHS Vulnerability Disclosure
