# Supplementary material for: A Multi-Pronged Technique for Identifying Equisetum palustre and Equisetum arvense—Combining HPTLC, HPLC-ESI-MS/MS and Optimized DNA Barcoding Techniques
Source: Plants (Basel). 2022 Sep 28;11(19):2562. doi: 10.3390/plants11192562 (PMC9573248; doi:10.3390/plants11192562)
Supplement: Supplementary file 1 [file plants-11-02562-s001.zip › plants-1932149 supplementary/Horsetail Supplementary Figures/Figure S29 (A6, 82704220323-1).pdf]

BLAST Results

Questions/comments

Job title: Nucleotide Sequence

**RID**	J1CWKHW7016 (Expires on 09-15 00:45 am)		
**Query ID**	lcl|Query_63351	**Database Name**	nt
**Description**	None	**Description**	Nucleotide collection (nt)
**Molecule type**	dna	**Program**	BLASTN 2.13.0+
**Query Length**	665		

Graphic Summary

Distribution of the top 100 Blast Hits on 100 subject sequences

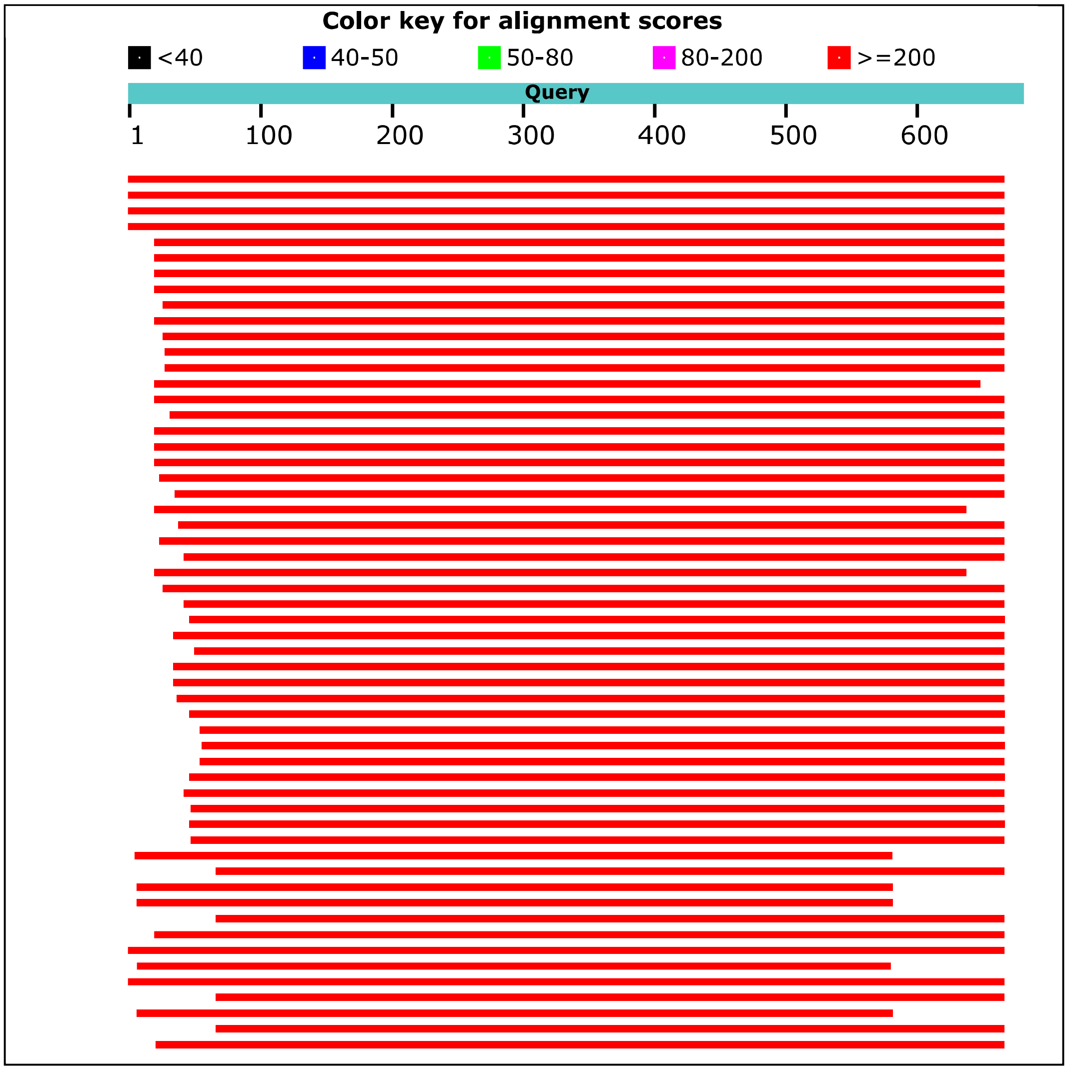

Descriptions

Sequences producing significant alignments:

Description	Max Score	Total Score	Query Cover	E value	Per. Ident	Accession
Equisetum arvense chloroplast, complete genome	1138	1138	99%	0.0	97.89%	GU191334.1
Equisetum arvense chloroplast ribulosebisphosphate carboxylase (rbcL) gene, complete cds	1138	1138	99%	0.0	97.89%	L11053.1
Equisetum arvense plastid, complete genome	1133	1133	99%	0.0	97.74%	JN968380.1
Equisetum palustre chloroplast, partial genome	1122	1122	99%	0.0	97.44%	MT984458.1
Equisetum environmental sample isolate EDNA16-0043490 ribulose-1,5-bisphosphate carboxylase/oxygenase large subunit (rbcL) gene, partial cds; chloroplast	1112	1112	96%	0.0	98.14%	MT197555.1
Equisetum fluviatile ribulose-1,5-bisphosphate carboxylase/oxygenase large subunit (rbcL) gene, partial cds; chloroplast gene for chloroplast product	1112	1112	96%	0.0	98.14%	AY226142.1
Equisetum arvense ribulose-1,5-bisphosphate carboxylase/oxygenase large subunit (rbcL) gene, partial cds; chloroplast gene for chloroplast product	1112	1112	96%	0.0	98.14%	AY226140.1
Equisetum environmental sample isolate EDNA16-0043491b ribulose-1,5-bisphosphate carboxylase/oxygenase large subunit (rbcL) gene, partial cds; chloroplast	1107	1107	96%	0.0	97.98%	MT197548.1
Equisetum arvense subsp. boreale isolate 41074 ribulose-1,5-bisphosphate carboxylase/oxygenase large subunit (rbcL) gene, partial cds; plastid	1101	1101	95%	0.0	98.12%	MH750160.1
Equisetum diffusum ribulose-1,5-bisphosphate carboxylase/oxygenase large subunit (rbcL) gene, partial cds; chloroplast gene for chloroplast product	1101	1101	96%	0.0	97.83%	AY226141.1
Equisetum x dycei isolate 26083 ribulose-1,5-bisphosphate carboxylase/oxygenase large subunit (rbcL) gene, partial cds; plastid	1099	1099	95%	0.0	98.12%	MH750220.1
Equisetum fluviatile isolate 41076 ribulose-1,5-bisphosphate carboxylase/oxygenase large subunit (rbcL) gene, partial cds; plastid	1098	1098	95%	0.0	98.11%	MH750169.1
Equisetum fluviatile isolate 41075 ribulose-1,5-bisphosphate carboxylase/oxygenase large subunit (rbcL) gene, partial cds; plastid	1098	1098	95%	0.0	98.11%	MH750168.1
Equisetum arvense voucher JAG288 (OAC) ribulose-1,5-bisphosphate carboxylase/oxygenase large subunit (rbcL) gene, partial cds; chloroplast	1098	1098	93%	0.0	98.56%	EU677106.1

Description	Max Score	Total Score	Query Cover	E value	Per. Ident	Accession
Equisetum palustre ribulose-1,5-bisphosphate carboxylase/oxygenase large subunit (rbcL) gene, partial cds; chloroplast gene for chloroplast product	1096	1096	96%	0.0	97.67%	AY226138.1
Equisetum arvense subsp. boreale isolate 41073 ribulose-1,5-bisphosphate carboxylase/oxygenase large subunit (rbcL) gene, partial cds; plastid	1090	1090	95%	0.0	98.10%	MH750159.1
Equisetum sylvaticum ribulose-1,5-bisphosphate carboxylase/oxygenase large subunit (rbcL) gene, partial cds; chloroplast gene for chloroplast product	1090	1090	96%	0.0	97.52%	AY226136.1
Equisetum pratense ribulose-1,5-bisphosphate carboxylase/oxygenase large subunit (rbcL) gene, partial cds; chloroplast gene for chloroplast product	1085	1085	96%	0.0	97.36%	AY226137.1
Equisetum telmateia subsp. braunii ribulose-1,5-bisphosphate carboxylase/oxygenase large subunit (rbcL) gene, partial cds; chloroplast gene for chloroplast product	1085	1085	96%	0.0	97.36%	AY226135.1
Equisetum sylvaticum isolate 11640 ribulose-1,5-bisphosphate carboxylase/oxygenase large subunit (rbcL) gene, partial cds; plastid	1083	1083	96%	0.0	97.50%	MH750204.1
Equisetum arvense subsp. arvense isolate 41071 ribulose-1,5-bisphosphate carboxylase/oxygenase large subunit (rbcL) gene, partial cds; plastid	1083	1083	94%	0.0	98.09%	MH750158.1
Equisetum arvense voucher AP400 (OAC) ribulose-1,5-bisphosphate carboxylase/oxygenase large subunit (rbcL) gene, partial cds; chloroplast	1079	1079	92%	0.0	98.54%	EU677105.1
Equisetum x litorale isolate 41084 ribulose-1,5-bisphosphate carboxylase/oxygenase large subunit (rbcL) gene, partial cds; plastid	1077	1077	93%	0.0	98.08%	MH750222.1
Equisetum telmateia subsp. telmateia isolate 11642 ribulose-1,5-bisphosphate carboxylase/oxygenase large subunit (rbcL) gene, partial cds; plastid	1077	1077	96%	0.0	97.34%	MH750210.1
Equisetum arvense subsp. arvense isolate 26085 ribulose-1,5-bisphosphate carboxylase/oxygenase large subunit (rbcL) gene, partial cds; plastid	1072	1072	93%	0.0	98.07%	MH750156.1
Equisetum pratense voucher AP451 ribulose-1,5-bisphosphate carboxylase/oxygenase large subunit (rbcL) gene, partial cds; chloroplast	1072	1072	92%	0.0	98.21%	HQ590083.1
Equisetum telmateia ribulose-1,5-bisphosphate carboxylase large subunit (rbcL) gene, partial cds; chloroplast gene for chloroplast product	1072	1072	95%	0.0	97.33%	AF313580.1
Equisetum diffusum isolate 40689 ribulose-1,5-bisphosphate carboxylase/oxygenase large subunit (rbcL) gene, partial cds; plastid	1066	1066	93%	0.0	97.91%	MH750166.1
Equisetum arvense subsp. arvense isolate 26084 ribulose-1,5-bisphosphate carboxylase/oxygenase large subunit (rbcL) gene, partial cds; plastid	1064	1064	92%	0.0	98.06%	MH750155.1

Description	Max Score	Total Score	Query Cover	E value	Per. Ident	Accession
Equisetum sylvaticum isolate 41081 ribulose-1,5-bisphosphate carboxylase/oxygenase large subunit (rbcL) gene, partial cds; plastid	1062	1062	94%	0.0	97.46%	MH750205.1
Equisetum arvense subsp. arvense isolate 40833 ribulose-1,5-bisphosphate carboxylase/oxygenase large subunit (rbcL) gene, partial cds; plastid	1061	1061	92%	0.0	98.21%	MH750157.1
Equisetum telmateia subsp. braunii isolate 40828 ribulose-1,5-bisphosphate carboxylase/oxygenase large subunit (rbcL) gene, partial cds; plastid	1057	1057	94%	0.0	97.30%	MH750207.1
Equisetum pratense isolate 41087 ribulose-1,5-bisphosphate carboxylase/oxygenase large subunit (rbcL) gene, partial cds; plastid	1057	1057	94%	0.0	97.30%	MH750195.1
Equisetum x fontqueri isolate 26093 ribulose-1,5-bisphosphate carboxylase/oxygenase large subunit (rbcL) gene, partial cds; plastid	1053	1053	94%	0.0	97.29%	MH750221.1
Equisetum diffusum isolate 40804 ribulose-1,5-bisphosphate carboxylase/oxygenase large subunit (rbcL) gene, partial cds; plastid	1053	1053	92%	0.0	97.73%	MH750167.1
Equisetum fluviatile ribulose-1,5-bisphosphate carboxylase/oxygenase large subunit (rbcL) gene, partial cds; chloroplast	1053	1053	91%	0.0	98.19%	DQ463101.1
Equisetum arvense x Equisetum telmateia subsp. braunii isolate 40834 ribulose-1,5-bisphosphate carboxylase/oxygenase large subunit (rbcL) gene, partial cds; plastid	1051	1051	91%	0.0	98.19%	MH750161.1
Equisetum arvense ribulose-1,5-bisphosphate carboxylase/oxygenase large subunit (rbcL) gene, partial cds; chloroplast	1050	1050	91%	0.0	98.03%	KM218345.1
Equisetum palustre isolate 17671 ribulose-1,5-bisphosphate carboxylase/oxygenase large subunit (rbcL) gene, partial cds; plastid	1046	1046	92%	0.0	97.57%	MH750191.1
Equisetum telmateia subsp. braunii isolate 40832 ribulose-1,5-bisphosphate carboxylase/oxygenase large subunit (rbcL) gene, partial cds; plastid	1044	1044	93%	0.0	97.27%	MH750208.1
Equisetum palustre isolate 39349 ribulose-1,5-bisphosphate carboxylase/oxygenase large subunit (rbcL) gene, partial cds; plastid	1044	1044	92%	0.0	97.56%	MH750192.1
Equisetum pratense isolate 41086 ribulose-1,5-bisphosphate carboxylase/oxygenase large subunit (rbcL) gene, partial cds; plastid	1037	1037	92%	0.0	97.25%	MH750194.1
Equisetum pratense isolate 39348 ribulose-1,5-bisphosphate carboxylase/oxygenase large subunit (rbcL) gene, partial cds; plastid	1033	1033	92%	0.0	97.24%	MH750193.1
Equisetum arvense voucher TJD-508 ribulose-1,5-bisphosphate carboxylase/oxygenase large subunit (rbcL) gene, partial cds; chloroplast	1033	1033	86%	0.0	99.30%	KJ841301.1

Description	Max Score	Total Score	Query Cover	E value	Per. Ident	Accession
Equisetum fluviatile chloroplast rbcL gene for ribulose-1,5-bisphosphate carboxylase/oxygenase large subunit, partial cds, specimen_voucher: TNS:776384	1033	1033	89%	0.0	98.16%	AB574684.1
Equisetum arvense voucher TJD-503 ribulose-1,5-bisphosphate carboxylase/oxygenase large subunit (rbcL) gene, partial cds; chloroplast	1031	1031	86%	0.0	99.30%	KJ841302.1
Equisetum arvense voucher TJD-504 ribulose-1,5-bisphosphate carboxylase/oxygenase large subunit (rbcL) gene, partial cds; chloroplast	1029	1029	85%	0.0	99.30%	KJ841300.1
Equisetum arvense chloroplast rbcL gene for ribulose-1,5-bisphosphate carboxylase/oxygenase large subunit, partial cds, specimen_voucher: TNS:763618	1027	1027	89%	0.0	97.99%	AB574683.1
Equisetum scirpoides ribulose-1,5-bisphosphate carboxylase/oxygenase large subunit (rbcL) gene, partial cds; chloroplast gene for chloroplast product	1024	1024	96%	0.0	95.65%	AY226133.1
Equisetum xylochaetum chloroplast, complete genome	1022	1022	99%	0.0	94.73%	MW282958.1
Commelina diffusa voucher Q555 ribulose-1,5-bisphosphate carboxylase/oxygenase large subunit (rbcL) gene, partial cds; chloroplast	1022	1022	85%	0.0	99.12%	MH714073.1
Equisetum hyemale chloroplast, complete genome	1016	1016	99%	0.0	94.58%	KC117177.1
Equisetum palustre chloroplast rbcL gene for ribulose-1,5-bisphosphate carboxylase/oxygenase large subunit, partial cds, specimen_voucher: TNS:776981	1016	1016	89%	0.0	97.66%	AB574686.1
Equisetum sylvaticum voucher TJD-502 ribulose-1,5-bisphosphate carboxylase/oxygenase large subunit (rbcL) gene, partial cds; chloroplast	1014	1014	86%	0.0	98.78%	KJ841305.1
Equisetum sylvaticum chloroplast rbcL gene for ribulose-1,5-bisphosphate carboxylase/oxygenase large subunit, partial cds, specimen_voucher: TNS:776974	1011	1011	89%	0.0	97.49%	AB574690.1
Equisetum bogotense ribulose-1,5-bisphosphate carboxylase/oxygenase large subunit (rbcL) gene, partial cds; chloroplast gene for chloroplast product	1011	1011	96%	0.0	95.33%	AY226139.1
Equisetum ramosissimum isolate BPTPS123 voucher LISE:96444 chloroplast, complete genome	1005	1005	99%	0.0	94.28%	ON641349.1
Equisetum ramosissimum isolate BPTPS056 voucher LISE:96334 chloroplast, complete genome	1005	1005	99%	0.0	94.28%	NC_062377.1
Equisetum ramosissimum isolate BPTPS056 voucher LISE:96334 chloroplast, complete genome	1005	1005	99%	0.0	94.28%	OM691674.1

Description	Max Score	Total Score	Query Cover	E value	Per. Ident	Accession
Equisetum pratense chloroplast rbcL gene for ribulose-1,5-bisphosphate carboxylase/oxygenase large subunit, partial cds, specimen_voucher: TNS:1107843	1005	1005	89%	0.0	97.32%	AB574687.1
Equisetum scirpoides isolate 41089 ribulose-1,5-bisphosphate carboxylase/oxygenase large subunit (rbcL) gene, partial cds; plastid	1002	1002	94%	0.0	95.71%	MH750203.1
Equisetum komarovii isolate 40830 ribulose-1,5-bisphosphate carboxylase/oxygenase large subunit (rbcL) gene, partial cds; plastid	1002	1002	94%	0.0	95.71%	MH750182.1
Equisetum arvense voucher WAB_1079140221 ribulose-1,5-bisphosphate carboxylase/oxygenase large subunit (rbcL) gene, partial cds; chloroplast	1002	1002	83%	0.0	99.28%	KJ593388.1
Equisetum arvense subsp. alpestre voucher Gillespie_10109_CAN ribulose-1,5-bisphosphate carboxylase/oxygenase large subunit (rbcL) gene, partial cds; chloroplast	1002	1002	83%	0.0	99.28%	KC482713.1
Equisetum hyemale ribulose-1,5-bisphosphate carboxylase/oxygenase large subunit (rbcL) gene, partial cds; chloroplast	1002	1002	99%	0.0	94.13%	DQ646001.1
Equisetum arvense ribulose-1,5-bisphosphate carboxylase/oxygenase large subunit (rbcL) gene, partial cds; chloroplast	1000	1000	83%	0.0	99.28%	MZ359289.1
Equisetum fluviatile voucher 09PROBE-05547 ribulose-1,5-bisphosphate carboxylase/oxygenase large subunit (rbcL) gene, partial cds; chloroplast	1000	1000	83%	0.0	99.28%	JN965528.1
Equisetum arvense voucher BIOUG24048-F01 ribulose-1,5-bisphosphate carboxylase/oxygenase large subunit (rbcL) gene, partial cds; chloroplast	998	998	83%	0.0	99.28%	KT695464.1
Equisetum scirpoides voucher AP248 ribulose-1,5-bisphosphate carboxylase/oxygenase large subunit (rbcL) gene, partial cds; chloroplast	996	996	92%	0.0	96.10%	HQ590084.1
Equisetum myriochaetum ribulose-1,5-bisphosphate carboxylase/oxygenase large subunit (rbcL) gene, partial cds; chloroplast gene for chloroplast product	996	996	96%	0.0	94.88%	AY226131.1
Equisetum giganteum ribulose-1,5-bisphosphate carboxylase/oxygenase large subunit (rbcL) gene, partial cds; chloroplast gene for chloroplast product	996	996	96%	0.0	94.88%	AY226127.1
Equisetum arvense voucher G00199177 ribulose-1,5-bisphosphate carboxylase/oxygenase large subunit (rbcL) gene, partial cds; chloroplast	994	994	83%	0.0	99.28%	KJ204336.1
Equisetum sp. Schuettpelz 1643 ribulose-1,5-bisphosphate carboxylase/oxygenase large subunit (rbcL) gene, partial cds; chloroplast	994	994	96%	0.0	94.87%	MW620286.1

Description	Max Score	Total Score	Query Cover	E value	Per. Ident	Accession
Equisetum sylvaticum plastid partial rbcL gene for ribulose-1,5-biphosphate carboxylase/oxygenase large subunit, specimen voucher H823764	992	992	84%	0.0	98.58%	HE574610.1
Equisetum laevigatum ribulose-1,5-bisphosphate carboxylase/oxygenase large subunit (rbcL) gene, partial cds; chloroplast gene for chloroplast product	992	992	96%	0.0	94.86%	AY226130.1
Equisetum scirpoides isolate 26090 ribulose-1,5-bisphosphate carboxylase/oxygenase large subunit (rbcL) gene, partial cds; plastid	990	990	94%	0.0	95.39%	MH750202.1
Equisetum hyemale subsp. affine voucher OAC 94916 ribulose-1,5-bisphosphate carboxylase/oxygenase large subunit (rbcL) gene, partial cds; chloroplast	990	990	96%	0.0	94.72%	KF186500.1
Equisetum palustre voucher Chase 11641 (K) ribulose-1,5-bisphosphate carboxylase/oxygenase large subunit (rbcL) gene, partial cds; chloroplast	990	990	83%	0.0	98.92%	GQ248601.1
Equisetum hyemale subsp. affine ribulose-1,5-bisphosphate carboxylase/oxygenase large subunit (rbcL) gene, partial cds; chloroplast gene for chloroplast product	990	990	96%	0.0	94.72%	AY226128.1
Equisetum giganteum isolate 40806 ribulose-1,5-bisphosphate carboxylase/oxygenase large subunit (rbcL) gene, partial cds; plastid	989	989	96%	0.0	94.84%	MH750172.1
Equisetum giganteum isolate 26087 ribulose-1,5-bisphosphate carboxylase/oxygenase large subunit (rbcL) gene, partial cds; plastid	989	989	96%	0.0	94.84%	MH750170.1
Equisetum ramosissimum voucher R825 ribulose-1,5-bisphosphate carboxylase/oxygenase large subunit (rbcl) gene, partial cds; chloroplast	989	989	97%	0.0	94.45%	KY656709.1
Equisetum palustre voucher Gillespie_8830b ribulose-1,5-bisphosphate carboxylase/oxygenase large subunit (rbcL) gene, partial cds; chloroplast	989	989	83%	0.0	98.92%	KC482730.1
Equisetum x ferrissii ribulose-1,5-bisphosphate carboxylase/oxygenase large subunit (rbcL) gene, partial cds; chloroplast	989	989	96%	0.0	94.84%	EU352292.1
Equisetum x ferrissii ribulose-1,5-bisphosphate carboxylase large subunit (rbcL) gene, partial cds; chloroplast gene for chloroplast product	987	987	96%	0.0	94.84%	AF313579.1
Equisetum sylvaticum voucher WAB_1079140180 ribulose-1,5-bisphosphate carboxylase/oxygenase large subunit (rbcL) gene, partial cds; chloroplast	985	985	83%	0.0	98.74%	KJ593395.1
Equisetum pratense voucher WAB_0132469185 ribulose-1,5-bisphosphate carboxylase/oxygenase large subunit (rbcL) gene, partial cds; chloroplast	985	985	83%	0.0	98.74%	KJ593391.1

Description	Max Score	Total Score	Query Cover	E value	Per. Ident	Accession
Equisetum palustre ribulose-1,5-bisphosphate carboxylase/oxygenase large subunit (rbcL) gene, partial cds; chloroplast	983	983	82%	0.0	99.09%	MK525785.1
Equisetum telmateia voucher CCDB-18287-A07 ribulose-1,5-bisphosphate carboxylase/oxygenase large subunit (rbcL) gene, partial cds; chloroplast	983	983	83%	0.0	98.74%	MG215015.1
Equisetum sylvaticum voucher Gillespie_8524 ribulose-1,5-bisphosphate carboxylase/oxygenase large subunit (rbcL) gene, partial cds; chloroplast	983	983	83%	0.0	98.74%	KC482739.1
Equisetum scirpoides isolate 10933 ribulose-1,5-bisphosphate carboxylase/oxygenase large subunit (rbcL) gene, partial cds; plastid	981	981	92%	0.0	95.63%	MH750201.1
Equisetum ramosissimum subsp. debile voucher G.M. Plunkett 3642 (NYBG) ribulose-1,5-bisphosphate carboxylase/oxygenase large subunit (rbcL) gene, partial cds; chloroplast	979	979	96%	0.0	94.41%	MT657901.1
Equisetum ramosissimum subsp. debile ribulose-1,5-bisphosphate carboxylase/oxygenase large subunit (rbcL) gene, partial cds; chloroplast gene for chloroplast product	979	979	96%	0.0	94.41%	AY226132.1
Equisetum myriochaetum isolate 40815 ribulose-1,5-bisphosphate carboxylase/oxygenase large subunit (rbcL) gene, partial cds; plastid	977	977	95%	0.0	94.66%	MH750185.1
Equisetum sylvaticum ribulose-1,5-bisphosphate carboxylase/oxygenase large subunit (rbcL) gene, partial cds; chloroplast	977	977	82%	0.0	98.91%	MK525787.1
Equisetum pratense ribulose-1,5-bisphosphate carboxylase/oxygenase large subunit (rbcL) gene, partial cds; chloroplast	977	977	82%	0.0	98.91%	MK525786.1
Equisetum hyemale voucher AP344 (OAC) ribulose-1,5-bisphosphate carboxylase/oxygenase large subunit (rbcL) gene, partial cds; chloroplast	976	976	93%	0.0	95.04%	EU677107.1
Equisetum bogotense isolate 40800 ribulose-1,5-bisphosphate carboxylase/oxygenase large subunit (rbcL) gene, partial cds; plastid	972	972	93%	0.0	95.18%	MH750162.1
Equisetum xylochaetum isolate 40614 ribulose-1,5-bisphosphate carboxylase/oxygenase large subunit (rbcL) gene, partial cds; plastid	968	968	94%	0.0	94.75%	MH750228.1
Equisetum myriochaetum isolate 41080 ribulose-1,5-bisphosphate carboxylase/oxygenase large subunit (rbcL) gene, partial cds; plastid	968	968	94%	0.0	94.75%	MH750190.1

Alignments

Equisetum arvense chloroplast, complete genome

Sequence ID: **GU191334.1** Length: 133309 Number of Matches: 1
Range 1: 27313 to 27965

Score	Expect	Identities	Gaps	Strand	Frame
1138 bits(616)	0.0()	650/664(98%)	11/664(1%)	Plus/Minus	

Features:

```
Query  1      CCACAAACAGAGACTAAAGCAGGTGTTGGATTTAAAGCTGGTGTTAAAGAATATCGATTG  60
              |||||||| ||||| ||||||||||||||||||||||||||||||||||| |||||||||
Sbjct  27965  CCACAAACGGAGACCAAAGCAGGTGTTGGATTTAAAGCTGGTGTTAAAGATTATCGATTG  27906

Query  61     ACTTATTTTACTCCAGATTATGAAACCAAAGATACCGATATTTTAGCAGCATTCCGTATG  120
              ||||||||||||||||||||||||||||||||||||||||||||||||||||||||||||
Sbjct  27905  ACTTATTTTACTCCAGATTATGAAACCAAAGATACCGATATTTTAGCAGCATTCCGTATG  27846

Query  121    ACTCCTCAACCGGGAGTACCACCGGAAGAAGCAGGAGCAGCCGTAGCTGCTGAATCGTCC  180
              ||||||||||||||||||||||||||||||||||||||||||||||||||||||||||||
Sbjct  27845  ACTCCTCAACCGGGAGTACCACCGGAAGAAGCAGGAGCAGCCGTAGCTGCTGAATCGTCC  27786

Query  181    ACGGGCACCTGGACTACCGTATGGACAGATGGACTTACTAGTCTTGATCGATATAAAGGT  240
              ||||||||||||||||||||||||||||||||||||||||||||||||||||||||||||
Sbjct  27785  ACGGGCACCTGGACTACCGTATGGACAGATGGACTTACTAGTCTTGATCGATATAAAGGT  27726

Query  241    CGATGCTATAATATTGAACCTGTTGCTGGAGAAGATAATCAATTCATAGCTTATGTAGCC  300
              ||||||||||||||||||||||||||||||||||||||||||||||||||||||||||||
Sbjct  27725  CGATGCTATAATATTGAACCTGTTGCTGGAGAAGATAATCAATTCATAGCTTATGTAGCC  27666

Query  301    TACCCTTTAGATCTTTTTGAAGAAGGTTCTGTTACCAATCTTTTTACTTCAATTGTTGGT  360
              ||||||||||||||||||||||||||||||||||||||||||||||||||||||||||||
Sbjct  27665  TACCCTTTAGATCTTTTTGAAGAAGGTTCTGTTACCAATCTTTTTACTTCAATTGTTGGT  27606

Query  361    AATGTTTTCGGCTTCAAAGCTCTACGTGCTTTACGTTTAGAAGATTTAAGAATTCCTCCT  420
              ||||||||||||||||||||||||||||||||||||||||||||||||||||||||||||
Sbjct  27605  AATGTTTTCGGCTTCAAAGCTCTACGTGCTTTACGTTTAGAAGATTTAAGAATTCCTCCT  27546

Query  421    GCTTACTCCAAAACTTTTATAGGACCACCCCACGGTATCCAGGTTGAAAGAGATAAGTTA  480
              ||||||||||||||||||||||||||||||||||||||||||||||||||||||||||||
Sbjct  27545  GCTTACTCCAAAACTTTTATAGGACCACCCCACGGTATCCAGGTTGAAAGAGATAAGTTA  27486

Query  481    AACAAATACGGTCGTCCTTTATTAGGTTTGTACAATTAAACCAAAATTGGGACTATCTGC  540
              |||||||||||||||||||||||||||| |||||||||||||||||||||||||||||||
Sbjct  27485  AACAAATACGGTCGTCCTTTATTAGGTT-GTACAATTAAACCAAAATTGGGACTATCTGC  27427

Query  541    TAAAAACTATGGTAGGAGCTGTTTTATGAATGTCTTCGTGGTGGGCTTGATTTTCACCAA  600
              ||||||||||||||| ||||| ||||||||||||||||||||||||||||||| ||||||
Sbjct  27426  TAAAAACTATGGTAG-AGCTG-TTTATGAATGTCTTCGTGGTGGGCTTGATTT-CACCAA  27370

Query  601    AAGATGATGAGAAATGTAAAACTCTCAACCCCTTTATGCGTTGGAAGAGATCCGATTTCT  660
              | ||||||||||| |||||| ||||||| |||||||||||||||| |||||| | |||||
Sbjct  27369  A-GATGATGAGAA-TGTAAA-CTCTCAA-CCCTTTATGCGTTGGA-GAGATC-G-TTTCT  27317

Query  661    TATT  664
              ||||
Sbjct  27316  TATT  27313
```

Equisetum arvense chloroplast ribulosebisphosphate carboxylase (rbcL) gene, complete cds
Sequence ID: **L11053.1** Length: 1587 Number of Matches: 1
Range 1: 79 to 731

Score	Expect	Identities	Gaps	Strand	Frame
1138 bits(616)	0.0()	650/664(98%)	11/664(1%)	Plus/Plus	

Features:

```
Query  1    CCACAAACAGAGACTAAAGCAGGTGTTGGATTTAAAGCTGGTGTTAAAGAATATCGATTG  60
            |||||||| ||||| ||||||||||||||||||||||||||||||||||| |||||||||
Sbjct  79   CCACAAACGGAGACCAAAGCAGGTGTTGGATTTAAAGCTGGTGTTAAAGATTATCGATTG  138

Query  61   ACTTATTTTACTCCAGATTATGAAACCAAAGATACCGATATTTTAGCAGCATTCCGTATG  120
            ||||||||||||||||||||||||||||||||||||||||||||||||||||||||||||
Sbjct  139  ACTTATTTTACTCCAGATTATGAAACCAAAGATACCGATATTTTAGCAGCATTCCGTATG  198

Query  121  ACTCCTCAACCGGGAGTACCACCGGAAGAAGCAGGAGCAGCCGTAGCTGCTGAATCGTCC  180
            ||||||||||||||||||||||||||||||||||||||||||||||||||||||||||||
Sbjct  199  ACTCCTCAACCGGGAGTACCACCGGAAGAAGCAGGAGCAGCCGTAGCTGCTGAATCGTCC  258

Query  181  ACGGGCACCTGGACTACCGTATGGACAGATGGACTTACTAGTCTTGATCGATATAAAGGT  240
            ||||||||||||||||||||||||||||||||||||||||||||||||||||||||||||
Sbjct  259  ACGGGCACCTGGACTACCGTATGGACAGATGGACTTACTAGTCTTGATCGATATAAAGGT  318

Query  241  CGATGCTATAATATTGAACCTGTTGCTGGAGAAGATAATCAATTCATAGCTTATGTAGCC  300
            ||||||||||||||||||||||||||||||||||||||||||||||||||||||||||||
Sbjct  319  CGATGCTATAATATTGAACCTGTTGCTGGAGAAGATAATCAATTCATAGCTTATGTAGCC  378
```

```
Query  301   TACCCTTTAGATCTTTTTGAAGAAGGTTCTGTTACCAATCTTTTTACTTCAATTGTTGGT  360
             ||||||||||||||||||||||||||||||||||||||||||||||||||||||||||||
Sbjct  379   TACCCTTTAGATCTTTTTGAAGAAGGTTCTGTTACCAATCTTTTTACTTCAATTGTTGGT  438

Query  361   AATGTTTTCGGCTTCAAAGCTCTACGTGCTTTACGTTTAGAAGATTTAAGAATTCCTCCT  420
             ||||||||||||||||||||||||||||||||||||||||||||||||||||||||||||
Sbjct  439   AATGTTTTCGGCTTCAAAGCTCTACGTGCTTTACGTTTAGAAGATTTAAGAATTCCTCCT  498

Query  421   GCTTACTCCAAAACTTTTATAGGACCACCCCACGGTATCCAGGTTGAAAGAGATAAGTTA  480
             ||||||||||||||||||||||||||||||||||||||||||||||||||||||||||||
Sbjct  499   GCTTACTCCAAAACTTTTATAGGACCACCCCACGGTATCCAGGTTGAAAGAGATAAGTTA  558

Query  481   AACAAATACGGTCGTCCTTTATTAGGTTTGTACAATTAAACCAAAATTGGGACTATCTGC  540
             |||||||||||||||||||||||||| |||||||||||||||||||||||||||||||||
Sbjct  559   AACAAATACGGTCGTCCTTTATTAGG-TTGTACAATTAAACCAAAATTGGGACTATCTGC  617

Query  541   TAAAAACTATGGTAGGAGCTGTTTTATGAATGTCTTCGTGGTGGGCTTGATTTTCACCAA  600
             ||||||||||||||| ||||| ||||||||||||||||||||||||||||||| ||||||
Sbjct  618   TAAAAACTATGGTAG-AGCTG-TTTATGAATGTCTTCGTGGTGGGCTTGATTT-CACCAA  674

Query  601   AAGATGATGAGAAATGTAAAACTCTCAACCCCTTTATGCGTTGGAAGAGATCCGATTTCT  660
             | ||||||||||| |||||| ||||||| |||||||||||||||| |||||| | |||||
Sbjct  675   A-GATGATGAGAA-TGTAAA-CTCTCAA-CCCTTTATGCGTTGGA-GAGATC-G-TTTCT  727

Query  661   TATT  664
             ||||
Sbjct  728   TATT  731
```

Equisetum arvense plastid, complete genome

Sequence ID: **JN968380.1** Length: 132726 Number of Matches: 1

Range 1: 64964 to 65616

Score	Expect	Identities	Gaps	Strand	Frame
1133 bits(613)	0.0()	649/664(98%)	11/664(1%)	Plus/Plus	

Features:

```
Query  1       CCACAAACAGAGACTAAAGCAGGTGTTGGATTTAAAGCTGGTGTTAAAGAATATCGATTG  60
               |||||||| ||||| ||||||||||||||||||||||||||||||||||| |||||||||
Sbjct  64964   CCACAAACGGAGACCAAAGCAGGTGTTGGATTTAAAGCTGGTGTTAAAGATTATCGATTG  65023

Query  61      ACTTATTTTACTCCAGATTATGAAACCAAAGATACCGATATTTTAGCAGCATTCCGTATG  120
               ||||||||||||||||||||||||||||||||||||||||||||||||||||||||||||
Sbjct  65024   ACTTATTTTACTCCAGATTATGAAACCAAAGATACCGATATTTTAGCAGCATTCCGTATG  65083

Query  121     ACTCCTCAACCGGGAGTACCACCGGAAGAAGCAGGAGCAGCCGTAGCTGCTGAATCGTCC  180
               ||||||||||||||||||||||||||||||||||||||||||||||||||||||||||||
Sbjct  65084   ACTCCTCAACCGGGAGTACCACCGGAAGAAGCAGGAGCAGCCGTAGCTGCTGAATCGTCC  65143

Query  181     ACGGGCACCTGGACTACCGTATGGACAGATGGACTTACTAGTCTTGATCGATATAAAGGT  240
               ||||||||||||||||||||||||||||||||||||||||||||||||||||||||||||
Sbjct  65144   ACGGGCACCTGGACTACCGTATGGACAGATGGACTTACTAGTCTTGATCGATATAAAGGT  65203

Query  241     CGATGCTATAATATTGAACCTGTTGCTGGAGAAGATAATCAATTCATAGCTTATGTAGCC  300
               ||||||||||||||||||||||||||||||||||||||||||||||||||||||||||||
Sbjct  65204   CGATGCTATAATATTGAACCTGTTGCTGGAGAAGATAATCAATTCATAGCTTATGTAGCC  65263

Query  301     TACCCTTTAGATCTTTTTGAAGAAGGTTCTGTTACCAATCTTTTTACTTCAATTGTTGGT  360
               ||||||||||||||||||||||||||||||||||||||||||||||||||||||||||||
Sbjct  65264   TACCCTTTAGATCTTTTTGAAGAAGGTTCTGTTACCAATCTTTTTACTTCAATTGTTGGT  65323

Query  361     AATGTTTTCGGCTTCAAAGCTCTACGTGCTTTACGTTTAGAAGATTTAAGAATTCCTCCT  420
               ||||||||||| ||||||||||||||||||||||||||||||||||||||||||||||||
Sbjct  65324   AATGTTTTCGGATTCAAAGCTCTACGTGCTTTACGTTTAGAAGATTTAAGAATTCCTCCT  65383

Query  421     GCTTACTCCAAAACTTTTATAGGACCACCCCACGGTATCCAGGTTGAAAGAGATAAGTTA  480
               ||||||||||||||||||||||||||||||||||||||||||||||||||||||||||||
Sbjct  65384   GCTTACTCCAAAACTTTTATAGGACCACCCCACGGTATCCAGGTTGAAAGAGATAAGTTA  65443

Query  481     AACAAATACGGTCGTCCTTTATTAGGTTTGTACAATTAAACCAAAATTGGGACTATCTGC  540
               |||||||||||||||||||||||||| |||||||||||||||||||||||||||||||||
Sbjct  65444   AACAAATACGGTCGTCCTTTATTAGG-TTGTACAATTAAACCAAAATTGGGACTATCTGC  65502

Query  541     TAAAAACTATGGTAGGAGCTGTTTTATGAATGTCTTCGTGGTGGGCTTGATTTTCACCAA  600
               ||||||||||||||| ||||| ||||||||||||||||||||||||||||||| ||||||
Sbjct  65503   TAAAAACTATGGTAG-AGCTG-TTTATGAATGTCTTCGTGGTGGGCTTGATTT-CACCAA  65559

Query  601     AAGATGATGAGAAATGTAAAACTCTCAACCCCTTTATGCGTTGGAAGAGATCCGATTTCT  660
               | ||||||||||| |||||| ||||||| |||||||||||||||| |||||| | |||||
Sbjct  65560   A-GATGATGAGAA-TGTAAA-CTCTCAA-CCCTTTATGCGTTGGA-GAGATC-G-TTTCT  65612

Query  661     TATT  664
               ||||
Sbjct  65613   TATT  65616
```

Equisetum palustre chloroplast, partial genome

Sequence ID: **MT984458.1** Length: 5501 Number of Matches: 1
Range 1: 2219 to 2871

**Score**	**Expect**	**Identities**	**Gaps**	**Strand**	**Frame**
1122 bits(607)	0.0()	647/664(97%)	11/664(1%)	Plus/Plus	

Features:

```
Query  1     CCACAAACAGAGACTAAAGCAGGTGTTGGATTTAAAGCTGGTGTTAAAGAATATCGATTG  60
             |||||||| ||||| ||||||||||||||||||||||||||||||||||| |||||||||
Sbjct  2219  CCACAAACGGAGACCAAAGCAGGTGTTGGATTTAAAGCTGGTGTTAAAGATTATCGATTG  2278

Query  61    ACTTATTTTACTCCAGATTATGAAACCAAAGATACCGATATTTTAGCAGCATTCCGTATG  120
             ||||||||||||||||||||||||||||||||||||||||||||||||||||||||||||
Sbjct  2279  ACTTATTTTACTCCAGATTATGAAACCAAAGATACCGATATTTTAGCAGCATTCCGTATG  2338

Query  121   ACTCCTCAACCGGGAGTACCACCGGAAGAAGCAGGAGCAGCCGTAGCTGCTGAATCGTCC  180
             ||||||||||||||||||||||||||||||||||||||||||||||||||||||||||||
Sbjct  2339  ACTCCTCAACCGGGAGTACCACCGGAAGAAGCAGGAGCAGCCGTAGCTGCTGAATCGTCC  2398

Query  181   ACGGGCACCTGGACTACCGTATGGACAGATGGACTTACTAGTCTTGATCGATATAAAGGT  240
             ||||||||||||||||||||||||||||||||||||||||||||||||||||||||||||
Sbjct  2399  ACGGGCACCTGGACTACCGTATGGACAGATGGACTTACTAGTCTTGATCGATATAAAGGT  2458

Query  241   CGATGCTATAATATTGAACCTGTTGCTGGAGAAGATAATCAATTCATAGCTTATGTAGCC  300
             ||||||||||||||||||||||||||||||||||||||||||||||||||||||||||||
Sbjct  2459  CGATGCTATAATATTGAACCTGTTGCTGGAGAAGATAATCAATTCATAGCTTATGTAGCC  2518

Query  301   TACCCTTTAGATCTTTTTGAAGAAGGTTCTGTTACCAATCTTTTTACTTCAATTGTTGGT  360
             || |||||||||||||||||||||||||||||||||||||||||||||||||||||||||
Sbjct  2519  TATCCTTTAGATCTTTTTGAAGAAGGTTCTGTTACCAATCTTTTTACTTCAATTGTTGGT  2578

Query  361   AATGTTTTCGGCTTCAAAGCTCTACGTGCTTTACGTTTAGAAGATTTAAGAATTCCTCCT  420
             ||||||||||||||||||||||||||||||||||||||||||||||||||||||||||||
Sbjct  2579  AATGTTTTCGGCTTCAAAGCTCTACGTGCTTTACGTTTAGAAGATTTAAGAATTCCTCCT  2638

Query  421   GCTTACTCCAAAACTTTTATAGGACCACCCCACGGTATCCAGGTTGAAAGAGATAAGTTA  480
             ||||||||||||||||||||||||||||||||||||||||||||||||||||||||||||
Sbjct  2639  GCTTACTCCAAAACTTTTATAGGACCACCCCACGGTATCCAGGTTGAAAGAGATAAGTTA  2698

Query  481   AACAAATACGGTCGTCCTTTATTAGGTTTGTACAATTAAACCAAAATTGGGACTATCTGC  540
             |||||||| ||||||||||||||||| |||||||||||||||||||||||||||||||||
Sbjct  2699  AACAAATATGGTCGTCCTTTATTAGG-TTGTACAATTAAACCAAAATTGGGACTATCTGC  2757

Query  541   TAAAAACTATGGTAGGAGCTGTTTTATGAATGTCTTCGTGGTGGGCTTGATTTTCACCAA  600
             |||||||||||||||| |||| |||||||||||||||||||||| |||||||| ||||||
Sbjct  2758  TAAAAACTATGGTAGG-GCTG-TTTATGAATGTCTTCGTGGTGGACTTGATTT-CACCAA  2814

Query  601   AAGATGATGAGAAATGTAAAACTCTCAACCCCTTTATGCGTTGGAAGAGATCCGATTTCT  660
             | ||||||||||| |||||| ||||||| |||||||||||||||| |||||| | |||||
Sbjct  2815  A-GATGATGAGAA-TGTAAA-CTCTCAA-CCCTTTATGCGTTGGA-GAGATC-G-TTTCT  2867

Query  661   TATT  664
             ||||
Sbjct  2868  TATT  2871
```

Equisetum environmental sample isolate EDNA16-0043490 ribulose-1,5-bisphosphate carboxylase/oxygenase large subunit (rbcL) gene, partial cds; chloroplast

Sequence ID: **MT197555.1** Length: 697 Number of Matches: 1
Range 1: 1 to 633

**Score**	**Expect**	**Identities**	**Gaps**	**Strand**	**Frame**
1112 bits(602)	0.0()	632/644(98%)	11/644(1%)	Plus/Plus	

Features:

```
Query  21   AGGTGTTGGATTTAAAGCTGGTGTTAAAGAATATCGATTGACTTATTTTACTCCAGATTA  80
            |||||||||||||||||||||||||||||| |||||||||||||||||||||||||||||
Sbjct  1    AGGTGTTGGATTTAAAGCTGGTGTTAAAGATTATCGATTGACTTATTTTACTCCAGATTA  60

Query  81   TGAAACCAAAGATACCGATATTTTAGCAGCATTCCGTATGACTCCTCAACCGGGAGTACC  140
            ||||||||||||||||||||||||||||||||||||||||||||||||||||||||||||
Sbjct  61   TGAAACCAAAGATACCGATATTTTAGCAGCATTCCGTATGACTCCTCAACCGGGAGTACC  120

Query  141  ACCGGAAGAAGCAGGAGCAGCCGTAGCTGCTGAATCGTCCACGGGCACCTGGACTACCGT  200
            ||||||||||||||||||||||||||||||||||||||||||||||||||||||||||||
Sbjct  121  ACCGGAAGAAGCAGGAGCAGCCGTAGCTGCTGAATCGTCCACGGGCACCTGGACTACCGT  180

Query  201  ATGGACAGATGGACTTACTAGTCTTGATCGATATAAAGGTCGATGCTATAATATTGAACC  260
            ||||||||||||||||||||||||||||||||||||||||||||||||||||||||||||
Sbjct  181  ATGGACAGATGGACTTACTAGTCTTGATCGATATAAAGGTCGATGCTATAATATTGAACC  240
```

```
Query  261   TGTTGCTGGAGAAGATAATCAATTCATAGCTTATGTAGCCTACCCTTTAGATCTTTTTGA  320
             ||||||||||||||||||||||||||||||||||||||||||||||||||||||||||||
Sbjct  241   TGTTGCTGGAGAAGATAATCAATTCATAGCTTATGTAGCCTACCCTTTAGATCTTTTTGA  300

Query  321   AGAAGGTTCTGTTACCAATCTTTTTACTTCAATTGTTGGTAATGTTTTCGGCTTCAAAGC  380
             ||||||||||||||||||||||||||||||||||||||||||||||||||||||||||||
Sbjct  301   AGAAGGTTCTGTTACCAATCTTTTTACTTCAATTGTTGGTAATGTTTTCGGCTTCAAAGC  360

Query  381   TCTACGTGCTTTACGTTTAGAAGATTTAAGAATTCCTCCTGCTTACTCCAAAACTTTTAT  440
             ||||||||||||||||||||||||||||||||||||||||||||||||||||||||||||
Sbjct  361   TCTACGTGCTTTACGTTTAGAAGATTTAAGAATTCCTCCTGCTTACTCCAAAACTTTTAT  420

Query  441   AGGACCACCCCACGGTATCCAGGTTGAAAGAGATAAGTTAAACAAATACGGTCGTCCTTT  500
             ||||||||||||||||||||||||||||||||||||||||||||||||||||||||||||
Sbjct  421   AGGACCACCCCACGGTATCCAGGTTGAAAGAGATAAGTTAAACAAATACGGTCGTCCTTT  480

Query  501   ATTAGGTTTGTACAATTAAACCAAAATTGGGACTATCTGCTAAAAACTATGGTAGGAGCT  560
             |||||| |||||||||||||||||||||||||||||||||||||||||||||||| ||||
Sbjct  481   ATTAGG-TTGTACAATTAAACCAAAATTGGGACTATCTGCTAAAAACTATGGTAG-AGCT  538

Query  561   GTTTTATGAATGTCTTCGTGGTGGGCTTGATTTTCACCAAAAGATGATGAGAAATGTAAA  620
             | ||||||||||||||||||||||||||||||| ||||||| ||||||||||| ||||||
Sbjct  539   G-TTTATGAATGTCTTCGTGGTGGGCTTGATTT-CACCAAA-GATGATGAGAA-TGTAAA  594

Query  621   ACTCTCAACCCCTTTATGCGTTGGAAGAGATCCGATTTCTTATT  664
              ||||||| |||||||||||||||| |||||| | |||||||||
Sbjct  595   -CTCTCAA-CCCTTTATGCGTTGGA-GAGATC-G-TTTCTTATT  633
```


BLAST is a registered trademark of the National Library of Medicine

Support center Mailing list YouTube

- National Library Of Medicine
- National Institutes Of Health
- U.S. Department of Health & Human Services
- USA.gov

NCBI

National Center for Biotechnology Information, U.S. National Library of Medicine 8600 Rockville Pike, Bethesda MD, 20894 USA

Policies and Guidelines | Contact

HHS Vulnerability Disclosure
