# Supplementary material for: A Multi-Pronged Technique for Identifying Equisetum palustre and Equisetum arvense—Combining HPTLC, HPLC-ESI-MS/MS and Optimized DNA Barcoding Techniques
Source: Plants (Basel). 2022 Sep 28;11(19):2562. doi: 10.3390/plants11192562 (PMC9573248; doi:10.3390/plants11192562)
Supplement: Supplementary file 1 [file plants-11-02562-s001.zip › plants-1932149 supplementary/Horsetail Supplementary Figures/Figure S30 (A7, 82708220323-1).pdf]

BLAST Results

Questions/comments

Job title: Nucleotide Sequence

**RID**	J1CZS9FT016 (Expires on 09-15 00:46 am)
**Query ID**	lcl|Query_30347
**Description**	None
**Molecule type**	dna
**Query Length**	653
**Database Name**	nt
**Description**	Nucleotide collection (nt)
**Program**	BLASTN 2.13.0+

Graphic Summary

Distribution of the top 100 Blast Hits on 100 subject sequences

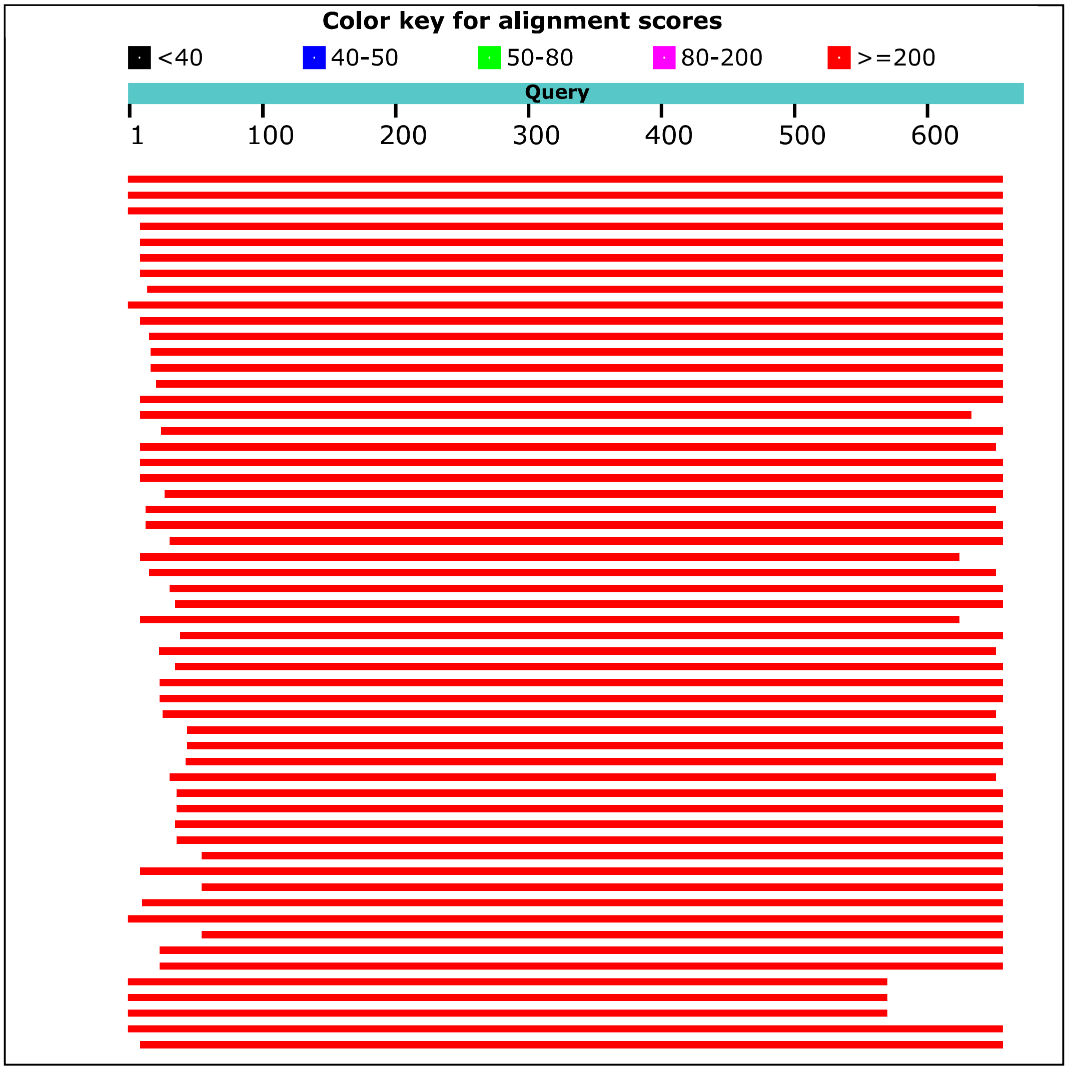

Descriptions

Sequences producing significant alignments:

Description	Max Score	Total Score	Query Cover	E value	Per. Ident	Accession
Equisetum arvense chloroplast, complete genome	1164	1164	99%	0.0	99.08%	GU191334.1
Equisetum arvense chloroplast ribulosebisphosphate carboxylase (rbcL) gene, complete cds	1164	1164	99%	0.0	99.08%	L11053.1
Equisetum arvense plastid, complete genome	1158	1158	99%	0.0	98.92%	JN968380.1
Equisetum environmental sample isolate EDNA16-0043490 ribulose-1,5-bisphosphate carboxylase/oxygenase large subunit (rbcL) gene, partial cds; chloroplast	1153	1153	98%	0.0	99.22%	MT197555.1
Equisetum fluviatile ribulose-1,5-bisphosphate carboxylase/oxygenase large subunit (rbcL) gene, partial cds; chloroplast gene for chloroplast product	1153	1153	98%	0.0	99.22%	AY226142.1
Equisetum arvense ribulose-1,5-bisphosphate carboxylase/oxygenase large subunit (rbcL) gene, partial cds; chloroplast gene for chloroplast product	1153	1153	98%	0.0	99.22%	AY226140.1
Equisetum environmental sample isolate EDNA16-0043491b ribulose-1,5-bisphosphate carboxylase/oxygenase large subunit (rbcL) gene, partial cds; chloroplast	1147	1147	98%	0.0	99.06%	MT197548.1
Equisetum arvense subsp. boreale isolate 41074 ribulose-1,5-bisphosphate carboxylase/oxygenase large subunit (rbcL) gene, partial cds; plastid	1142	1142	97%	0.0	99.21%	MH750160.1
Equisetum palustre chloroplast, partial genome	1142	1142	99%	0.0	98.46%	MT984458.1
Equisetum diffusum ribulose-1,5-bisphosphate carboxylase/oxygenase large subunit (rbcL) gene, partial cds; chloroplast gene for chloroplast product	1142	1142	98%	0.0	98.91%	AY226141.1
Equisetum x dycei isolate 26083 ribulose-1,5-bisphosphate carboxylase/oxygenase large subunit (rbcL) gene, partial cds; plastid	1140	1140	97%	0.0	99.21%	MH750220.1
Equisetum fluviatile isolate 41076 ribulose-1,5-bisphosphate carboxylase/oxygenase large subunit (rbcL) gene, partial cds; plastid	1138	1138	96%	0.0	99.21%	MH750169.1
Equisetum fluviatile isolate 41075 ribulose-1,5-bisphosphate carboxylase/oxygenase large subunit (rbcL) gene, partial cds; plastid	1138	1138	96%	0.0	99.21%	MH750168.1
Equisetum arvense subsp. boreale isolate 41073 ribulose-1,5-bisphosphate carboxylase/oxygenase large subunit (rbcL) gene, partial cds; plastid	1131	1131	96%	0.0	99.21%	MH750159.1

Description	Max Score	Total Score	Query Cover	E value	Per. Ident	Accession
Equisetum palustre ribulose-1,5-bisphosphate carboxylase/oxygenase large subunit (rbcL) gene, partial cds; chloroplast gene for chloroplast product	1131	1131	98%	0.0	98.60%	AY226138.1
Equisetum arvense voucher JAG288 (OAC) ribulose-1,5-bisphosphate carboxylase/oxygenase large subunit (rbcL) gene, partial cds; chloroplast	1129	1129	94%	0.0	99.68%	EU677106.1
Equisetum arvense subsp. arvense isolate 41071 ribulose-1,5-bisphosphate carboxylase/oxygenase large subunit (rbcL) gene, partial cds; plastid	1123	1123	95%	0.0	99.20%	MH750158.1
Equisetum telmateia subsp. braunii ribulose-1,5-bisphosphate carboxylase/oxygenase large subunit (rbcL) gene, partial cds; chloroplast gene for chloroplast product	1122	1122	97%	0.0	98.58%	AY226135.1
Equisetum pratense ribulose-1,5-bisphosphate carboxylase/oxygenase large subunit (rbcL) gene, partial cds; chloroplast gene for chloroplast product	1120	1120	98%	0.0	98.28%	AY226137.1
Equisetum sylvaticum ribulose-1,5-bisphosphate carboxylase/oxygenase large subunit (rbcL) gene, partial cds; chloroplast gene for chloroplast product	1120	1120	98%	0.0	98.28%	AY226136.1
Equisetum x litorale isolate 41084 ribulose-1,5-bisphosphate carboxylase/oxygenase large subunit (rbcL) gene, partial cds; plastid	1118	1118	95%	0.0	99.20%	MH750222.1
Equisetum telmateia subsp. telmateia isolate 11642 ribulose-1,5-bisphosphate carboxylase/oxygenase large subunit (rbcL) gene, partial cds; plastid	1114	1114	96%	0.0	98.58%	MH750210.1
Equisetum sylvaticum isolate 11640 ribulose-1,5-bisphosphate carboxylase/oxygenase large subunit (rbcL) gene, partial cds; plastid	1112	1112	97%	0.0	98.27%	MH750204.1
Equisetum arvense subsp. arvense isolate 26085 ribulose-1,5-bisphosphate carboxylase/oxygenase large subunit (rbcL) gene, partial cds; plastid	1112	1112	94%	0.0	99.19%	MH750156.1
Equisetum arvense voucher AP400 (OAC) ribulose-1,5-bisphosphate carboxylase/oxygenase large subunit (rbcL) gene, partial cds; chloroplast	1112	1112	93%	0.0	99.67%	EU677105.1
Equisetum telmateia ribulose-1,5-bisphosphate carboxylase large subunit (rbcL) gene, partial cds; chloroplast gene for chloroplast product	1109	1109	96%	0.0	98.57%	AF313580.1
Equisetum diffusum isolate 40689 ribulose-1,5-bisphosphate carboxylase/oxygenase large subunit (rbcL) gene, partial cds; plastid	1107	1107	94%	0.0	99.03%	MH750166.1
Equisetum arvense subsp. arvense isolate 26084 ribulose-1,5-bisphosphate carboxylase/oxygenase large subunit (rbcL) gene, partial cds; plastid	1105	1105	94%	0.0	99.19%	MH750155.1
Equisetum pratense voucher AP451 ribulose-1,5-bisphosphate carboxylase/oxygenase large subunit (rbcL) gene, partial cds; chloroplast	1105	1105	93%	0.0	99.34%	HQ590083.1

Description	Max Score	Total Score	Query Cover	E value	Per. Ident	Accession
Equisetum arvense subsp. arvense isolate 40833 ribulose-1,5-bisphosphate carboxylase/oxygenase large subunit (rbcL) gene, partial cds; plastid	1098	1098	93%	0.0	99.18%	MH750157.1
Equisetum telmateia subsp. braunii isolate 40828 ribulose-1,5-bisphosphate carboxylase/oxygenase large subunit (rbcL) gene, partial cds; plastid	1094	1094	95%	0.0	98.55%	MH750207.1
Equisetum diffusum isolate 40804 ribulose-1,5-bisphosphate carboxylase/oxygenase large subunit (rbcL) gene, partial cds; plastid	1094	1094	94%	0.0	98.86%	MH750167.1
Equisetum sylvaticum isolate 41081 ribulose-1,5-bisphosphate carboxylase/oxygenase large subunit (rbcL) gene, partial cds; plastid	1092	1092	95%	0.0	98.24%	MH750205.1
Equisetum pratense isolate 41087 ribulose-1,5-bisphosphate carboxylase/oxygenase large subunit (rbcL) gene, partial cds; plastid	1092	1092	95%	0.0	98.24%	MH750195.1
Equisetum x fontqueri isolate 26093 ribulose-1,5-bisphosphate carboxylase/oxygenase large subunit (rbcL) gene, partial cds; plastid	1090	1090	94%	0.0	98.55%	MH750221.1
Equisetum fluviatile ribulose-1,5-bisphosphate carboxylase/oxygenase large subunit (rbcL) gene, partial cds; chloroplast	1088	1088	92%	0.0	99.17%	DQ463101.1
Equisetum arvense x Equisetum telmateia subsp. braunii isolate 40834 ribulose-1,5-bisphosphate carboxylase/oxygenase large subunit (rbcL) gene, partial cds; plastid	1086	1086	92%	0.0	99.17%	MH750161.1
Equisetum arvense ribulose-1,5-bisphosphate carboxylase/oxygenase large subunit (rbcL) gene, partial cds; chloroplast	1085	1085	92%	0.0	99.01%	KM218345.1
Equisetum telmateia subsp. braunii isolate 40832 ribulose-1,5-bisphosphate carboxylase/oxygenase large subunit (rbcL) gene, partial cds; plastid	1081	1081	94%	0.0	98.53%	MH750208.1
Equisetum palustre isolate 17671 ribulose-1,5-bisphosphate carboxylase/oxygenase large subunit (rbcL) gene, partial cds; plastid	1081	1081	94%	0.0	98.53%	MH750191.1
Equisetum palustre isolate 39349 ribulose-1,5-bisphosphate carboxylase/oxygenase large subunit (rbcL) gene, partial cds; plastid	1079	1079	93%	0.0	98.53%	MH750192.1
Equisetum pratense isolate 41086 ribulose-1,5-bisphosphate carboxylase/oxygenase large subunit (rbcL) gene, partial cds; plastid	1072	1072	94%	0.0	98.21%	MH750194.1
Equisetum pratense isolate 39348 ribulose-1,5-bisphosphate carboxylase/oxygenase large subunit (rbcL) gene, partial cds; plastid	1068	1068	93%	0.0	98.21%	MH750193.1

Description	Max Score	Total Score	Query Cover	E value	Per. Ident	Accession
Equisetum fluviatile chloroplast rbcL gene for ribulose-1,5-bisphosphate carboxylase/oxygenase large subunit, partial cds, specimen_voucher: TNS:776384	1068	1068	91%	0.0	99.16%	AB574684.1
Equisetum scirpoides ribulose-1,5-bisphosphate carboxylase/oxygenase large subunit (rbcL) gene, partial cds; chloroplast gene for chloroplast product	1064	1064	98%	0.0	96.72%	AY226133.1
Equisetum arvense chloroplast rbcL gene for ribulose-1,5-bisphosphate carboxylase/oxygenase large subunit, partial cds, specimen_voucher: TNS:763618	1062	1062	91%	0.0	98.99%	AB574683.1
Equisetum bogotense ribulose-1,5-bisphosphate carboxylase/oxygenase large subunit (rbcL) gene, partial cds; chloroplast gene for chloroplast product	1051	1051	98%	0.0	96.41%	AY226139.1
Equisetum xylochaetum chloroplast, complete genome	1048	1048	99%	0.0	95.85%	MW282958.1
Equisetum palustre chloroplast rbcL gene for ribulose-1,5-bisphosphate carboxylase/oxygenase large subunit, partial cds, specimen_voucher: TNS:776981	1046	1046	91%	0.0	98.49%	AB574686.1
Equisetum scirpoides isolate 41089 ribulose-1,5-bisphosphate carboxylase/oxygenase large subunit (rbcL) gene, partial cds; plastid	1042	1042	95%	0.0	96.81%	MH750203.1
Equisetum komarovii isolate 40830 ribulose-1,5-bisphosphate carboxylase/oxygenase large subunit (rbcL) gene, partial cds; plastid	1042	1042	95%	0.0	96.81%	MH750182.1
Equisetum arvense voucher TJD-503 ribulose-1,5-bisphosphate carboxylase/oxygenase large subunit (rbcL) gene, partial cds; chloroplast	1042	1042	86%	0.0	100.00%	KJ841302.1
Equisetum arvense voucher TJD-508 ribulose-1,5-bisphosphate carboxylase/oxygenase large subunit (rbcL) gene, partial cds; chloroplast	1042	1042	86%	0.0	100.00%	KJ841301.1
Equisetum arvense voucher TJD-504 ribulose-1,5-bisphosphate carboxylase/oxygenase large subunit (rbcL) gene, partial cds; chloroplast	1042	1042	86%	0.0	100.00%	KJ841300.1
Equisetum hyemale chloroplast, complete genome	1042	1042	99%	0.0	95.69%	KC117177.1
Equisetum myriochaetum ribulose-1,5-bisphosphate carboxylase/oxygenase large subunit (rbcL) gene, partial cds; chloroplast gene for chloroplast product	1037	1037	98%	0.0	95.94%	AY226131.1
Equisetum giganteum ribulose-1,5-bisphosphate carboxylase/oxygenase large subunit (rbcL) gene, partial cds; chloroplast gene for chloroplast product	1037	1037	98%	0.0	95.94%	AY226127.1
Equisetum sp. Schuettpelz 1643 ribulose-1,5-bisphosphate carboxylase/oxygenase large subunit (rbcL) gene, partial cds; chloroplast	1035	1035	98%	0.0	95.94%	MW620286.1

Description	Max Score	Total Score	Query Cover	E value	Per. Ident	Accession
Commelina diffusa voucher Q555 ribulose-1,5-bisphosphate carboxylase/oxygenase large subunit (rbcL) gene, partial cds; chloroplast	1035	1035	86%	0.0	99.82%	MH714073.1
Equisetum sylvaticum chloroplast rbcL gene for ribulose-1,5-bisphosphate carboxylase/oxygenase large subunit, partial cds, specimen_voucher: TNS:776974	1035	1035	91%	0.0	98.15%	AB574690.1
Equisetum pratense chloroplast rbcL gene for ribulose-1,5-bisphosphate carboxylase/oxygenase large subunit, partial cds, specimen_voucher: TNS:1107843	1035	1035	91%	0.0	98.15%	AB574687.1
Equisetum laevigatum ribulose-1,5-bisphosphate carboxylase/oxygenase large subunit (rbcL) gene, partial cds; chloroplast gene for chloroplast product	1033	1033	97%	0.0	95.93%	AY226130.1
Equisetum scirpoides isolate 26090 ribulose-1,5-bisphosphate carboxylase/oxygenase large subunit (rbcL) gene, partial cds; plastid	1031	1031	95%	0.0	96.49%	MH750202.1
Equisetum ramosissimum isolate BPTPS123 voucher LISE:96444 chloroplast, complete genome	1031	1031	99%	0.0	95.38%	ON641349.1
Equisetum ramosissimum isolate BPTPS056 voucher LISE:96334 chloroplast, complete genome	1031	1031	99%	0.0	95.38%	NC_062377.1
Equisetum ramosissimum isolate BPTPS056 voucher LISE:96334 chloroplast, complete genome	1031	1031	99%	0.0	95.38%	OM691674.1
Equisetum hyemale subsp. affine voucher OAC 94916 ribulose-1,5-bisphosphate carboxylase/oxygenase large subunit (rbcL) gene, partial cds; chloroplast	1031	1031	98%	0.0	95.79%	KF186500.1
Equisetum hyemale subsp. affine ribulose-1,5-bisphosphate carboxylase/oxygenase large subunit (rbcL) gene, partial cds; chloroplast gene for chloroplast product	1031	1031	98%	0.0	95.79%	AY226128.1
Equisetum giganteum isolate 40806 ribulose-1,5-bisphosphate carboxylase/oxygenase large subunit (rbcL) gene, partial cds; plastid	1029	1029	97%	0.0	95.92%	MH750172.1
Equisetum giganteum isolate 26087 ribulose-1,5-bisphosphate carboxylase/oxygenase large subunit (rbcL) gene, partial cds; plastid	1029	1029	97%	0.0	95.92%	MH750170.1
Equisetum ramosissimum voucher R825 ribulose-1,5-bisphosphate carboxylase/oxygenase large subunit (rbcl) gene, partial cds; chloroplast	1029	1029	98%	0.0	95.51%	KY656709.1
Equisetum scirpoides voucher AP248 ribulose-1,5-bisphosphate carboxylase/oxygenase large subunit (rbcL) gene, partial cds; chloroplast	1029	1029	93%	0.0	97.21%	HQ590084.1
Equisetum x ferrissii ribulose-1,5-bisphosphate carboxylase/oxygenase large subunit (rbcL) gene, partial cds; chloroplast	1029	1029	97%	0.0	95.92%	EU352292.1

Description	Max Score	Total Score	Query Cover	E value	Per. Ident	Accession
Equisetum hyemale ribulose-1,5-bisphosphate carboxylase/oxygenase large subunit (rbcL) gene, partial cds; chloroplast	1029	1029	99%	0.0	95.38%	DQ646001.1
Equisetum x ferrissii ribulose-1,5-bisphosphate carboxylase large subunit (rbcL) gene, partial cds; chloroplast gene for chloroplast product	1027	1027	97%	0.0	95.91%	AF313579.1
Equisetum scirpoides isolate 10933 ribulose-1,5-bisphosphate carboxylase/oxygenase large subunit (rbcL) gene, partial cds; plastid	1022	1022	94%	0.0	96.75%	MH750201.1
Equisetum arvense voucher WAB_1079140221 ribulose-1,5-bisphosphate carboxylase/oxygenase large subunit (rbcL) gene, partial cds; chloroplast	1022	1022	84%	0.0	100.00%	KJ593388.1
Equisetum arvense subsp. alpestre voucher Gillespie_10109_CAN ribulose-1,5-bisphosphate carboxylase/oxygenase large subunit (rbcL) gene, partial cds; chloroplast	1022	1022	84%	0.0	100.00%	KC482713.1
Equisetum sylvaticum voucher TJD-502 ribulose-1,5-bisphosphate carboxylase/oxygenase large subunit (rbcL) gene, partial cds; chloroplast	1020	1020	86%	0.0	99.29%	KJ841305.1
Equisetum arvense ribulose-1,5-bisphosphate carboxylase/oxygenase large subunit (rbcL) gene, partial cds; chloroplast	1020	1020	84%	0.0	100.00%	MZ359289.1
Equisetum ramosissimum subsp. debile voucher G.M. Plunkett 3642 (NYBG) ribulose-1,5-bisphosphate carboxylase/oxygenase large subunit (rbcL) gene, partial cds; chloroplast	1020	1020	98%	0.0	95.48%	MT657901.1
Equisetum fluviatile voucher 09PROBE-05547 ribulose-1,5-bisphosphate carboxylase/oxygenase large subunit (rbcL) gene, partial cds; chloroplast	1020	1020	84%	0.0	100.00%	JN965528.1
Equisetum ramosissimum subsp. debile ribulose-1,5-bisphosphate carboxylase/oxygenase large subunit (rbcL) gene, partial cds; chloroplast gene for chloroplast product	1020	1020	98%	0.0	95.48%	AY226132.1
Equisetum myriochaetum isolate 40815 ribulose-1,5-bisphosphate carboxylase/oxygenase large subunit (rbcL) gene, partial cds; plastid	1018	1018	97%	0.0	95.74%	MH750185.1
Equisetum arvense voucher BIOUG24048-F01 ribulose-1,5-bisphosphate carboxylase/oxygenase large subunit (rbcL) gene, partial cds; chloroplast	1018	1018	84%	0.0	100.00%	KT695464.1
Equisetum arvense voucher G00199177 ribulose-1,5-bisphosphate carboxylase/oxygenase large subunit (rbcL) gene, partial cds; chloroplast	1014	1014	84%	0.0	100.00%	KJ204336.1
Equisetum bogotense isolate 40800 ribulose-1,5-bisphosphate carboxylase/oxygenase large subunit (rbcL) gene, partial cds; plastid	1013	1013	94%	0.0	96.28%	MH750162.1

Description	Max Score	Total Score	Query Cover	E value	Per. Ident	Accession
Equisetum xylochaetum isolate 40614 ribulose-1,5-bisphosphate carboxylase/oxygenase large subunit (rbcL) gene, partial cds; plastid	1009	1009	95%	0.0	95.85%	MH750228.1
Equisetum myriochaetum isolate 41080 ribulose-1,5-bisphosphate carboxylase/oxygenase large subunit (rbcL) gene, partial cds; plastid	1009	1009	95%	0.0	95.85%	MH750190.1
Equisetum myriochaetum isolate 40826 ribulose-1,5-bisphosphate carboxylase/oxygenase large subunit (rbcL) gene, partial cds; plastid	1009	1009	95%	0.0	95.85%	MH750188.1
Equisetum x schaffneri isolate 40813 ribulose-1,5-bisphosphate carboxylase/oxygenase large subunit (rbcL) gene, partial cds; plastid	1007	1007	95%	0.0	95.84%	MH750224.1
Equisetum hyemale voucher AP344 (OAC) ribulose-1,5-bisphosphate carboxylase/oxygenase large subunit (rbcL) gene, partial cds; chloroplast	1007	1007	94%	0.0	96.12%	EU677107.1
Equisetum giganteum isolate 40811 ribulose-1,5-bisphosphate carboxylase/oxygenase large subunit (rbcL) gene, partial cds; plastid	1005	1005	95%	0.0	95.83%	MH750176.1
Equisetum palustre voucher Chase 11641 (K) ribulose-1,5-bisphosphate carboxylase/oxygenase large subunit (rbcL) gene, partial cds; chloroplast	1005	1005	84%	0.0	99.46%	GQ248601.1
Equisetum hyemale subsp. hyemale isolate 41088 ribulose-1,5-bisphosphate carboxylase/oxygenase large subunit (rbcL) gene, partial cds; plastid	1003	1003	95%	0.0	95.69%	MH750181.1
Equisetum telmateia voucher CCDB-18287-A07 ribulose-1,5-bisphosphate carboxylase/oxygenase large subunit (rbcL) gene, partial cds; chloroplast	1003	1003	84%	0.0	99.46%	MG215015.1
Equisetum palustre voucher Gillespie_8830b ribulose-1,5-bisphosphate carboxylase/oxygenase large subunit (rbcL) gene, partial cds; chloroplast	1003	1003	84%	0.0	99.46%	KC482730.1
Equisetum variegatum ribulose-1,5-bisphosphate carboxylase/oxygenase large subunit (rbcL) gene, partial cds; chloroplast gene for chloroplast product	1003	1003	98%	0.0	95.01%	AY226134.1
Equisetum sylvaticum voucher WAB_1079140180 ribulose-1,5-bisphosphate carboxylase/oxygenase large subunit (rbcL) gene, partial cds; chloroplast	1000	1000	84%	0.0	99.28%	KJ593395.1
Equisetum pratense voucher WAB_0132469185 ribulose-1,5-bisphosphate carboxylase/oxygenase large subunit (rbcL) gene, partial cds; chloroplast	1000	1000	84%	0.0	99.28%	KJ593391.1

Alignments

Equisetum arvense chloroplast, complete genome

Sequence ID: **GU191334.1** Length: 133309 Number of Matches: 1
Range 1: 27308 to 27954

Score	Expect	Identities	Gaps	Strand	Frame
1164 bits(630)	0.0()	644/650(99%)	3/650(0%)	Plus/Minus	

Features:

```
Query  1      GACTAAAGCAGGTGTTGGATTTAAAGCTGGTGTTAAAGATTATCGATTGACTTATTTTAC  60
              ||| ||||||||||||||||||||||||||||||||||||||||||||||||||||||||
Sbjct  27954  GACCAAAGCAGGTGTTGGATTTAAAGCTGGTGTTAAAGATTATCGATTGACTTATTTTAC  27895

Query  61     TCCAGATTATGAAACCAAAGATACCGATATTTTAGCAGCATTCCGTATGACTCCTCAACC  120
              ||||||||||||||||||||||||||||||||||||||||||||||||||||||||||||
Sbjct  27894  TCCAGATTATGAAACCAAAGATACCGATATTTTAGCAGCATTCCGTATGACTCCTCAACC  27835

Query  121    GGGAGTACCACCGGAAGAAGCAGGAGCAGCCGTAGCTGCTGAATCGTCCACGGGCACCTG  180
              ||||||||||||||||||||||||||||||||||||||||||||||||||||||||||||
Sbjct  27834  GGGAGTACCACCGGAAGAAGCAGGAGCAGCCGTAGCTGCTGAATCGTCCACGGGCACCTG  27775

Query  181    GACTACCGTATGGACAGATGGACTTACTAGTCTTGATCGATATAAAGGTCGATGCTATAA  240
              ||||||||||||||||||||||||||||||||||||||||||||||||||||||||||||
Sbjct  27774  GACTACCGTATGGACAGATGGACTTACTAGTCTTGATCGATATAAAGGTCGATGCTATAA  27715

Query  241    TATTGAACCTGTTGCTGGAGAAGATAATCAATTCATAGCTTATGTAGCCTACCCTTTAGA  300
              ||||||||||||||||||||||||||||||||||||||||||||||||||||||||||||
Sbjct  27714  TATTGAACCTGTTGCTGGAGAAGATAATCAATTCATAGCTTATGTAGCCTACCCTTTAGA  27655

Query  301    TCTTTTTGAAGAAGGTTCTGTTACCAATCTTTTTACTTCAATTGTTGGTAATGTTTTCGG  360
              ||||||||||||||||||||||||||||||||||||||||||||||||||||||||||||
Sbjct  27654  TCTTTTTGAAGAAGGTTCTGTTACCAATCTTTTTACTTCAATTGTTGGTAATGTTTTCGG  27595

Query  361    CTTCAAAGCTCTACGTGCTTTACGTTTAGAAGATTTAAGAATTCCTCCTGCTTACTCCAA  420
              ||||||||||||||||||||||||||||||||||||||||||||||||||||||||||||
Sbjct  27594  CTTCAAAGCTCTACGTGCTTTACGTTTAGAAGATTTAAGAATTCCTCCTGCTTACTCCAA  27535

Query  421    AACTTTTATAGGACCACCCCACGGTATCCAGGTTGAAAGAGATAAGTTAAACAAATACGG  480
              ||||||||||||||||||||||||||||||||||||||||||||||||||||||||||||
Sbjct  27534  AACTTTTATAGGACCACCCCACGGTATCCAGGTTGAAAGAGATAAGTTAAACAAATACGG  27475

Query  481    TCGTCCTTTATTAGGTTGTACAATTAAACCAAAATTGGGACTATCTGCTAAAAACTATGG  540
              ||||||||||||||||||||||||||||||||||||||||||||||||||||||||||||
Sbjct  27474  TCGTCCTTTATTAGGTTGTACAATTAAACCAAAATTGGGACTATCTGCTAAAAACTATGG  27415

Query  541    TAGAGCTGTTTATGAATGTCTTCGTGGTGGGGCTTGATTTCACCAAAGATGATGAGAATG  600
              ||||||||||||||||||||||||||||||| ||||||||||||||||||||||||||||
Sbjct  27414  TAGAGCTGTTTATGAATGTCTTCGTGGTGGG-CTTGATTTCACCAAAGATGATGAGAATG  27356

Query  601    TAAACTCTCAAACCCTTTATGCGTTGGGAGAGATCGATTCTTATTTGTAG  650
              ||||||||||| ||||||||||||||| |||||||| |||||||| ||||
Sbjct  27355  TAAACTCTCAA-CCCTTTATGCGTTGG-AGAGATCGTTTCTTATTCGTAG  27308
```

Equisetum arvense chloroplast ribulosebisphosphate carboxylase (rbcL) gene, complete cds
Sequence ID: **L11053.1** Length: 1587 Number of Matches: 1
Range 1: 90 to 736

Score	Expect	Identities	Gaps	Strand	Frame
1164 bits(630)	0.0()	644/650(99%)	3/650(0%)	Plus/Plus	

Features:

```
Query  1    GACTAAAGCAGGTGTTGGATTTAAAGCTGGTGTTAAAGATTATCGATTGACTTATTTTAC  60
            ||| ||||||||||||||||||||||||||||||||||||||||||||||||||||||||
Sbjct  90   GACCAAAGCAGGTGTTGGATTTAAAGCTGGTGTTAAAGATTATCGATTGACTTATTTTAC  149

Query  61   TCCAGATTATGAAACCAAAGATACCGATATTTTAGCAGCATTCCGTATGACTCCTCAACC  120
            ||||||||||||||||||||||||||||||||||||||||||||||||||||||||||||
Sbjct  150  TCCAGATTATGAAACCAAAGATACCGATATTTTAGCAGCATTCCGTATGACTCCTCAACC  209

Query  121  GGGAGTACCACCGGAAGAAGCAGGAGCAGCCGTAGCTGCTGAATCGTCCACGGGCACCTG  180
            ||||||||||||||||||||||||||||||||||||||||||||||||||||||||||||
Sbjct  210  GGGAGTACCACCGGAAGAAGCAGGAGCAGCCGTAGCTGCTGAATCGTCCACGGGCACCTG  269

Query  181  GACTACCGTATGGACAGATGGACTTACTAGTCTTGATCGATATAAAGGTCGATGCTATAA  240
            ||||||||||||||||||||||||||||||||||||||||||||||||||||||||||||
Sbjct  270  GACTACCGTATGGACAGATGGACTTACTAGTCTTGATCGATATAAAGGTCGATGCTATAA  329

Query  241  TATTGAACCTGTTGCTGGAGAAGATAATCAATTCATAGCTTATGTAGCCTACCCTTTAGA  300
            ||||||||||||||||||||||||||||||||||||||||||||||||||||||||||||
Sbjct  330  TATTGAACCTGTTGCTGGAGAAGATAATCAATTCATAGCTTATGTAGCCTACCCTTTAGA  389

Query  301  TCTTTTTGAAGAAGGTTCTGTTACCAATCTTTTTACTTCAATTGTTGGTAATGTTTTCGG  360
            ||||||||||||||||||||||||||||||||||||||||||||||||||||||||||||
Sbjct  390  TCTTTTTGAAGAAGGTTCTGTTACCAATCTTTTTACTTCAATTGTTGGTAATGTTTTCGG  449
```

```
Query  361   CTTCAAAGCTCTACGTGCTTTACGTTTAGAAGATTTAAGAATTCCTCCTGCTTACTCCAA  420
             ||||||||||||||||||||||||||||||||||||||||||||||||||||||||||||
Sbjct  450   CTTCAAAGCTCTACGTGCTTTACGTTTAGAAGATTTAAGAATTCCTCCTGCTTACTCCAA  509

Query  421   AACTTTTATAGGACCACCCCACGGTATCCAGGTTGAAAGAGATAAGTTAAACAAATACGG  480
             ||||||||||||||||||||||||||||||||||||||||||||||||||||||||||||
Sbjct  510   AACTTTTATAGGACCACCCCACGGTATCCAGGTTGAAAGAGATAAGTTAAACAAATACGG  569

Query  481   TCGTCCTTTATTAGGTTGTACAATTAAACCAAAATTGGGACTATCTGCTAAAAACTATGG  540
             ||||||||||||||||||||||||||||||||||||||||||||||||||||||||||||
Sbjct  570   TCGTCCTTTATTAGGTTGTACAATTAAACCAAAATTGGGACTATCTGCTAAAAACTATGG  629

Query  541   TAGAGCTGTTTATGAATGTCTTCGTGGTGGGGCTTGATTTCACCAAAGATGATGAGAATG  600
             |||||||||||||||||||||||||||| |||||||||||||||||||||||||||||||
Sbjct  630   TAGAGCTGTTTATGAATGTCTTCGTGGT-GGGCTTGATTTCACCAAAGATGATGAGAATG  688

Query  601   TAAACTCTCAAACCCTTTATGCGTTGGGAGAGATCGATTCTTATTTGTAG  650
             ||||||||| ||||||||||||||| |||||||||| |||||||| ||||
Sbjct  689   TAAACTCTC-AACCCTTTATGCGTT-GGAGAGATCGTTTCTTATTCGTAG  736
```

Equisetum arvense plastid, complete genome

Sequence ID: **JN968380.1** Length: 132726 Number of Matches: 1
Range 1: 64975 to 65621

Score	Expect	Identities	Gaps	Strand	Frame
1158 bits(627)	0.0()	643/650(99%)	3/650(0%)	Plus/Plus	

Features:

```
Query  1      GACTAAAGCAGGTGTTGGATTTAAAGCTGGTGTTAAAGATTATCGATTGACTTATTTTAC  60
              ||| ||||||||||||||||||||||||||||||||||||||||||||||||||||||||
Sbjct  64975  GACCAAAGCAGGTGTTGGATTTAAAGCTGGTGTTAAAGATTATCGATTGACTTATTTTAC  65034

Query  61     TCCAGATTATGAAACCAAAGATACCGATATTTTAGCAGCATTCCGTATGACTCCTCAACC  120
              ||||||||||||||||||||||||||||||||||||||||||||||||||||||||||||
Sbjct  65035  TCCAGATTATGAAACCAAAGATACCGATATTTTAGCAGCATTCCGTATGACTCCTCAACC  65094

Query  121    GGGAGTACCACCGGAAGAAGCAGGAGCAGCCGTAGCTGCTGAATCGTCCACGGGCACCTG  180
              ||||||||||||||||||||||||||||||||||||||||||||||||||||||||||||
Sbjct  65095  GGGAGTACCACCGGAAGAAGCAGGAGCAGCCGTAGCTGCTGAATCGTCCACGGGCACCTG  65154

Query  181    GACTACCGTATGGACAGATGGACTTACTAGTCTTGATCGATATAAAGGTCGATGCTATAA  240
              ||||||||||||||||||||||||||||||||||||||||||||||||||||||||||||
Sbjct  65155  GACTACCGTATGGACAGATGGACTTACTAGTCTTGATCGATATAAAGGTCGATGCTATAA  65214

Query  241    TATTGAACCTGTTGCTGGAGAAGATAATCAATTCATAGCTTATGTAGCCTACCCTTTAGA  300
              ||||||||||||||||||||||||||||||||||||||||||||||||||||||||||||
Sbjct  65215  TATTGAACCTGTTGCTGGAGAAGATAATCAATTCATAGCTTATGTAGCCTACCCTTTAGA  65274

Query  301    TCTTTTTGAAGAAGGTTCTGTTACCAATCTTTTTACTTCAATTGTTGGTAATGTTTTCGG  360
              ||||||||||||||||||||||||||||||||||||||||||||||||||||||||||||
Sbjct  65275  TCTTTTTGAAGAAGGTTCTGTTACCAATCTTTTTACTTCAATTGTTGGTAATGTTTTCGG  65334

Query  361    CTTCAAAGCTCTACGTGCTTTACGTTTAGAAGATTTAAGAATTCCTCCTGCTTACTCCAA  420
               |||||||||||||||||||||||||||||||||||||||||||||||||||||||||||
Sbjct  65335  ATTCAAAGCTCTACGTGCTTTACGTTTAGAAGATTTAAGAATTCCTCCTGCTTACTCCAA  65394

Query  421    AACTTTTATAGGACCACCCCACGGTATCCAGGTTGAAAGAGATAAGTTAAACAAATACGG  480
              ||||||||||||||||||||||||||||||||||||||||||||||||||||||||||||
Sbjct  65395  AACTTTTATAGGACCACCCCACGGTATCCAGGTTGAAAGAGATAAGTTAAACAAATACGG  65454

Query  481    TCGTCCTTTATTAGGTTGTACAATTAAACCAAAATTGGGACTATCTGCTAAAAACTATGG  540
              ||||||||||||||||||||||||||||||||||||||||||||||||||||||||||||
Sbjct  65455  TCGTCCTTTATTAGGTTGTACAATTAAACCAAAATTGGGACTATCTGCTAAAAACTATGG  65514

Query  541    TAGAGCTGTTTATGAATGTCTTCGTGGTGGGGCTTGATTTCACCAAAGATGATGAGAATG  600
              |||||||||||||||||||||||||||| |||||||||||||||||||||||||||||||
Sbjct  65515  TAGAGCTGTTTATGAATGTCTTCGTGGT-GGGCTTGATTTCACCAAAGATGATGAGAATG  65573

Query  601    TAAACTCTCAAACCCTTTATGCGTTGGGAGAGATCGATTCTTATTTGTAG  650
              ||||||||| ||||||||||||||| |||||||||| |||||||| ||||
Sbjct  65574  TAAACTCTC-AACCCTTTATGCGTT-GGAGAGATCGTTTCTTATTCGTAG  65621
```

Equisetum environmental sample isolate EDNA16-0043490 ribulose-1,5-bisphosphate carboxylase/oxygenase large subunit (rbcL) gene, partial cds; chloroplast

Sequence ID: **MT197555.1** Length: 697 Number of Matches: 1
Range 1: 1 to 638

Score	Expect	Identities	Gaps	Strand	Frame

1153 bits(624)	0.0()	636/641(99%)	3/641(0%)	Plus/Plus

Features:

```
Query  10   AGGTGTTGGATTTAAAGCTGGTGTTAAAGATTATCGATTGACTTATTTTACTCCAGATTA  69
            ||||||||||||||||||||||||||||||||||||||||||||||||||||||||||||
Sbjct  1    AGGTGTTGGATTTAAAGCTGGTGTTAAAGATTATCGATTGACTTATTTTACTCCAGATTA  60

Query  70   TGAAACCAAAGATACCGATATTTTAGCAGCATTCCGTATGACTCCTCAACCGGGAGTACC  129
            ||||||||||||||||||||||||||||||||||||||||||||||||||||||||||||
Sbjct  61   TGAAACCAAAGATACCGATATTTTAGCAGCATTCCGTATGACTCCTCAACCGGGAGTACC  120

Query  130  ACCGGAAGAAGCAGGAGCAGCCGTAGCTGCTGAATCGTCCACGGGCACCTGGACTACCGT  189
            ||||||||||||||||||||||||||||||||||||||||||||||||||||||||||||
Sbjct  121  ACCGGAAGAAGCAGGAGCAGCCGTAGCTGCTGAATCGTCCACGGGCACCTGGACTACCGT  180

Query  190  ATGGACAGATGGACTTACTAGTCTTGATCGATATAAAGGTCGATGCTATAATATTGAACC  249
            ||||||||||||||||||||||||||||||||||||||||||||||||||||||||||||
Sbjct  181  ATGGACAGATGGACTTACTAGTCTTGATCGATATAAAGGTCGATGCTATAATATTGAACC  240

Query  250  TGTTGCTGGAGAAGATAATCAATTCATAGCTTATGTAGCCTACCCTTTAGATCTTTTTGA  309
            ||||||||||||||||||||||||||||||||||||||||||||||||||||||||||||
Sbjct  241  TGTTGCTGGAGAAGATAATCAATTCATAGCTTATGTAGCCTACCCTTTAGATCTTTTTGA  300

Query  310  AGAAGGTTCTGTTACCAATCTTTTTACTTCAATTGTTGGTAATGTTTTCGGCTTCAAAGC  369
            ||||||||||||||||||||||||||||||||||||||||||||||||||||||||||||
Sbjct  301  AGAAGGTTCTGTTACCAATCTTTTTACTTCAATTGTTGGTAATGTTTTCGGCTTCAAAGC  360

Query  370  TCTACGTGCTTTACGTTTAGAAGATTTAAGAATTCCTCCTGCTTACTCCAAAACTTTTAT  429
            ||||||||||||||||||||||||||||||||||||||||||||||||||||||||||||
Sbjct  361  TCTACGTGCTTTACGTTTAGAAGATTTAAGAATTCCTCCTGCTTACTCCAAAACTTTTAT  420

Query  430  AGGACCACCCCACGGTATCCAGGTTGAAAGAGATAAGTTAAACAAATACGGTCGTCCTTT  489
            ||||||||||||||||||||||||||||||||||||||||||||||||||||||||||||
Sbjct  421  AGGACCACCCCACGGTATCCAGGTTGAAAGAGATAAGTTAAACAAATACGGTCGTCCTTT  480

Query  490  ATTAGGTTGTACAATTAAACCAAAATTGGGACTATCTGCTAAAAACTATGGTAGAGCTGT  549
            ||||||||||||||||||||||||||||||||||||||||||||||||||||||||||||
Sbjct  481  ATTAGGTTGTACAATTAAACCAAAATTGGGACTATCTGCTAAAAACTATGGTAGAGCTGT  540

Query  550  TTATGAATGTCTTCGTGGTGGGGCTTGATTTCACCAAAGATGATGAGAATGTAAACTCTC  609
            ||||||||||||||||||| ||||||||||||||||||||||||||||||||||||||||
Sbjct  541  TTATGAATGTCTTCGTGGT-GGGCTTGATTTCACCAAAGATGATGAGAATGTAAACTCTC  599

Query  610  AAACCCTTTATGCGTTGGGAGAGATCGATTCTTATTTGTAG  650
             ||||||||||||||| |||||||||| |||||||| ||||
Sbjct  600  -AACCCTTTATGCGTT-GGAGAGATCGTTTCTTATTCGTAG  638
```

Equisetum fluviatile ribulose-1,5-bisphosphate carboxylase/oxygenase large subunit (rbcL) gene, partial cds; chloroplast gene for chloroplast product

Sequence ID: **AY226142.1** Length: 1325 Number of Matches: 1

Range 1: 1 to 638

Score	Expect	Identities	Gaps	Strand	Frame
1153 bits(624)	0.0()	636/641(99%)	3/641(0%)	Plus/Plus	

Features:

```
Query  10   AGGTGTTGGATTTAAAGCTGGTGTTAAAGATTATCGATTGACTTATTTTACTCCAGATTA  69
            ||||||||||||||||||||||||||||||||||||||||||||||||||||||||||||
Sbjct  1    AGGTGTTGGATTTAAAGCTGGTGTTAAAGATTATCGATTGACTTATTTTACTCCAGATTA  60

Query  70   TGAAACCAAAGATACCGATATTTTAGCAGCATTCCGTATGACTCCTCAACCGGGAGTACC  129
            ||||||||||||||||||||||||||||||||||||||||||||||||||||||||||||
Sbjct  61   TGAAACCAAAGATACCGATATTTTAGCAGCATTCCGTATGACTCCTCAACCGGGAGTACC  120

Query  130  ACCGGAAGAAGCAGGAGCAGCCGTAGCTGCTGAATCGTCCACGGGCACCTGGACTACCGT  189
            ||||||||||||||||||||||||||||||||||||||||||||||||||||||||||||
Sbjct  121  ACCGGAAGAAGCAGGAGCAGCCGTAGCTGCTGAATCGTCCACGGGCACCTGGACTACCGT  180

Query  190  ATGGACAGATGGACTTACTAGTCTTGATCGATATAAAGGTCGATGCTATAATATTGAACC  249
            ||||||||||||||||||||||||||||||||||||||||||||||||||||||||||||
Sbjct  181  ATGGACAGATGGACTTACTAGTCTTGATCGATATAAAGGTCGATGCTATAATATTGAACC  240

Query  250  TGTTGCTGGAGAAGATAATCAATTCATAGCTTATGTAGCCTACCCTTTAGATCTTTTTGA  309
            ||||||||||||||||||||||||||||||||||||||||||||||||||||||||||||
Sbjct  241  TGTTGCTGGAGAAGATAATCAATTCATAGCTTATGTAGCCTACCCTTTAGATCTTTTTGA  300

Query  310  AGAAGGTTCTGTTACCAATCTTTTTACTTCAATTGTTGGTAATGTTTTCGGCTTCAAAGC  369
            ||||||||||||||||||||||||||||||||||||||||||||||||||||||||||||
Sbjct  301  AGAAGGTTCTGTTACCAATCTTTTTACTTCAATTGTTGGTAATGTTTTCGGCTTCAAAGC  360

Query  370  TCTACGTGCTTTACGTTTAGAAGATTTAAGAATTCCTCCTGCTTACTCCAAAACTTTTAT  429
            ||||||||||||||||||||||||||||||||||||||||||||||||||||||||||||
Sbjct  361  TCTACGTGCTTTACGTTTAGAAGATTTAAGAATTCCTCCTGCTTACTCCAAAACTTTTAT  420
```

```
Query  430  AGGACCACCCCACGGTATCCAGGTTGAAAGAGATAAGTTAAACAAATACGGTCGTCCTTT  489
            ||||||||||||||||||||||||||||||||||||||||||||||||||||||||||||
Sbjct  421  AGGACCACCCCACGGTATCCAGGTTGAAAGAGATAAGTTAAACAAATACGGTCGTCCTTT  480

Query  490  ATTAGGTTGTACAATTAAACCAAAATTGGGACTATCTGCTAAAAACTATGGTAGAGCTGT  549
            ||||||||||||||||||||||||||||||||||||||||||||||||||||||||||||
Sbjct  481  ATTAGGTTGTACAATTAAACCAAAATTGGGACTATCTGCTAAAAACTATGGTAGAGCTGT  540

Query  550  TTATGAATGTCTTCGTGGTGGGGCTTGATTTCACCAAAGATGATGAGAATGTAAACTCTC  609
            ||||||||||||||||||| ||||||||||||||||||||||||||||||||||||||||
Sbjct  541  TTATGAATGTCTTCGTGGT-GGGCTTGATTTCACCAAAGATGATGAGAATGTAAACTCTC  599

Query  610  AAACCCTTTATGCGTTGGGAGAGATCGATTCTTATTTGTAG  650
             ||||||||||||||| |||||||||| |||||||| ||||
Sbjct  600  -AACCCTTTATGCGTT-GGAGAGATCGTTTCTTATTCGTAG  638
```


BLAST is a registered trademark of the National Library of Medicine

Support center Mailing list YouTube

- National Library Of Medicine
- National Institutes Of Health
- U.S. Department of Health & Human Services
- USA.gov

NCBI

National Center for Biotechnology Information, U.S. National Library of Medicine 8600 Rockville Pike, Bethesda MD, 20894 USA

Policies and Guidelines | Contact

HHS Vulnerability Disclosure
