# Supplementary material for: A Multi-Pronged Technique for Identifying Equisetum palustre and Equisetum arvense—Combining HPTLC, HPLC-ESI-MS/MS and Optimized DNA Barcoding Techniques
Source: Plants (Basel). 2022 Sep 28;11(19):2562. doi: 10.3390/plants11192562 (PMC9573248; doi:10.3390/plants11192562)
Supplement: Supplementary file 1 [file plants-11-02562-s001.zip › plants-1932149 supplementary/Horsetail Supplementary Figures/Figure S31 (A8, 83919220502-1).pdf]

BLAST ® » blastn suite » RID-J1D3GR71016

BLAST Results

Questions/comments

Job title: Nucleotide Sequence

**RID**	J1D3GR71016 (Expires on 09-15 00:48 am)		
**Query ID**	lcl|Query_39287	**Database Name**	nt
**Description**	None	**Description**	Nucleotide collection (nt)
**Molecule type**	dna	**Program**	BLASTN 2.13.0+
**Query Length**	656		

Graphic Summary

Distribution of the top 100 Blast Hits on 100 subject sequences

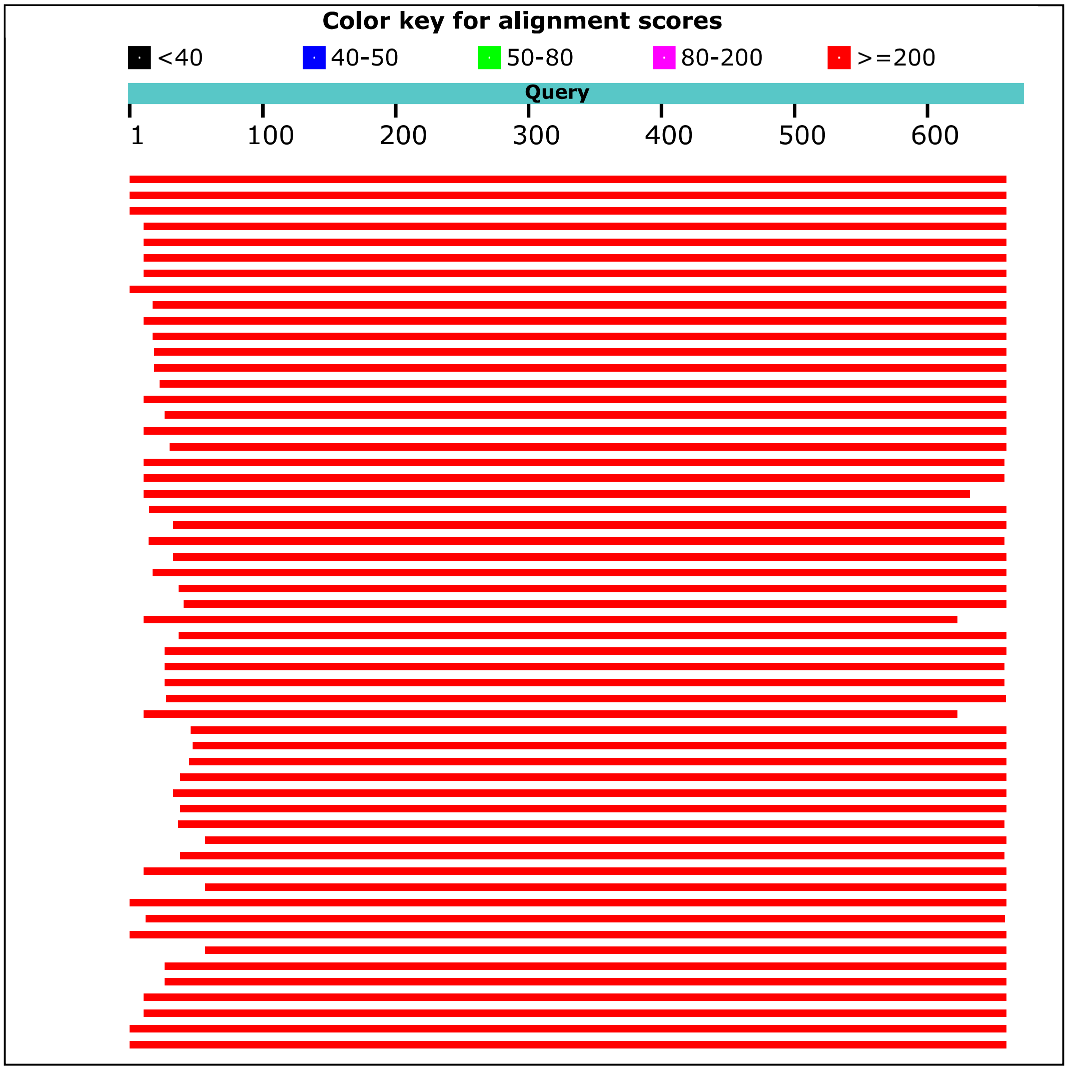

Descriptions

Sequences producing significant alignments:

Description	Max Score	Total Score	Query Cover	E value	Per. Ident	Accession
Equisetum arvense chloroplast, complete genome	1194	1194	99%	0.0	99.54%	GU191334.1
Equisetum arvense chloroplast ribulosebisphosphate carboxylase (rbcL) gene, complete cds	1194	1194	99%	0.0	99.54%	L11053.1
Equisetum arvense plastid, complete genome	1188	1188	99%	0.0	99.39%	JN968380.1
Equisetum environmental sample isolate EDNA16-0043490 ribulose-1,5-bisphosphate carboxylase/oxygenase large subunit (rbcL) gene, partial cds; chloroplast	1179	1179	98%	0.0	99.69%	MT197555.1
Equisetum fluviatile ribulose-1,5-bisphosphate carboxylase/oxygenase large subunit (rbcL) gene, partial cds; chloroplast gene for chloroplast product	1179	1179	98%	0.0	99.69%	AY226142.1
Equisetum arvense ribulose-1,5-bisphosphate carboxylase/oxygenase large subunit (rbcL) gene, partial cds; chloroplast gene for chloroplast product	1179	1179	98%	0.0	99.69%	AY226140.1
Equisetum environmental sample isolate EDNA16-0043491b ribulose-1,5-bisphosphate carboxylase/oxygenase large subunit (rbcL) gene, partial cds; chloroplast	1173	1173	98%	0.0	99.53%	MT197548.1
Equisetum palustre chloroplast, partial genome	1171	1171	99%	0.0	98.93%	MT984458.1
Equisetum arvense subsp. boreale isolate 41074 ribulose-1,5-bisphosphate carboxylase/oxygenase large subunit (rbcL) gene, partial cds; plastid	1168	1168	97%	0.0	99.69%	MH750160.1
Equisetum diffusum ribulose-1,5-bisphosphate carboxylase/oxygenase large subunit (rbcL) gene, partial cds; chloroplast gene for chloroplast product	1168	1168	98%	0.0	99.38%	AY226141.1
Equisetum x dycei isolate 26083 ribulose-1,5-bisphosphate carboxylase/oxygenase large subunit (rbcL) gene, partial cds; plastid	1166	1166	97%	0.0	99.69%	MH750220.1
Equisetum fluviatile isolate 41076 ribulose-1,5-bisphosphate carboxylase/oxygenase large subunit (rbcL) gene, partial cds; plastid	1164	1164	96%	0.0	99.69%	MH750169.1
Equisetum fluviatile isolate 41075 ribulose-1,5-bisphosphate carboxylase/oxygenase large subunit (rbcL) gene, partial cds; plastid	1164	1164	96%	0.0	99.69%	MH750168.1
Equisetum arvense subsp. boreale isolate 41073 ribulose-1,5-bisphosphate carboxylase/oxygenase large subunit (rbcL) gene, partial cds; plastid	1157	1157	96%	0.0	99.68%	MH750159.1

Description	Max Score	Total Score	Query Cover	E value	Per. Ident	Accession
Equisetum palustre ribulose-1,5-bisphosphate carboxylase/oxygenase large subunit (rbcL) gene, partial cds; chloroplast gene for chloroplast product	1157	1157	98%	0.0	99.07%	AY226138.1
Equisetum arvense subsp. arvense isolate 41071 ribulose-1,5-bisphosphate carboxylase/oxygenase large subunit (rbcL) gene, partial cds; plastid	1149	1149	95%	0.0	99.68%	MH750158.1
Equisetum telmateia subsp. braunii ribulose-1,5-bisphosphate carboxylase/oxygenase large subunit (rbcL) gene, partial cds; chloroplast gene for chloroplast product	1146	1146	98%	0.0	98.76%	AY226135.1
Equisetum x litorale isolate 41084 ribulose-1,5-bisphosphate carboxylase/oxygenase large subunit (rbcL) gene, partial cds; plastid	1144	1144	95%	0.0	99.68%	MH750222.1
Equisetum pratense ribulose-1,5-bisphosphate carboxylase/oxygenase large subunit (rbcL) gene, partial cds; chloroplast gene for chloroplast product	1144	1144	98%	0.0	98.76%	AY226137.1
Equisetum sylvaticum ribulose-1,5-bisphosphate carboxylase/oxygenase large subunit (rbcL) gene, partial cds; chloroplast gene for chloroplast product	1144	1144	98%	0.0	98.76%	AY226136.1
Equisetum arvense voucher JAG288 (OAC) ribulose-1,5-bisphosphate carboxylase/oxygenase large subunit (rbcL) gene, partial cds; chloroplast	1140	1140	94%	0.0	100.00%	EU677106.1
Equisetum telmateia subsp. telmateia isolate 11642 ribulose-1,5-bisphosphate carboxylase/oxygenase large subunit (rbcL) gene, partial cds; plastid	1138	1138	97%	0.0	98.75%	MH750210.1
Equisetum arvense subsp. arvense isolate 26085 ribulose-1,5-bisphosphate carboxylase/oxygenase large subunit (rbcL) gene, partial cds; plastid	1138	1138	94%	0.0	99.68%	MH750156.1
Equisetum sylvaticum isolate 11640 ribulose-1,5-bisphosphate carboxylase/oxygenase large subunit (rbcL) gene, partial cds; plastid	1136	1136	97%	0.0	98.75%	MH750204.1
Equisetum diffusum isolate 40689 ribulose-1,5-bisphosphate carboxylase/oxygenase large subunit (rbcL) gene, partial cds; plastid	1133	1133	94%	0.0	99.52%	MH750166.1
Equisetum telmateia ribulose-1,5-bisphosphate carboxylase large subunit (rbcL) gene, partial cds; chloroplast gene for chloroplast product	1133	1133	97%	0.0	98.74%	AF313580.1
Equisetum arvense subsp. arvense isolate 26084 ribulose-1,5-bisphosphate carboxylase/oxygenase large subunit (rbcL) gene, partial cds; plastid	1131	1131	94%	0.0	99.68%	MH750155.1
Equisetum arvense subsp. arvense isolate 40833 ribulose-1,5-bisphosphate carboxylase/oxygenase large subunit (rbcL) gene, partial cds; plastid	1123	1123	93%	0.0	99.67%	MH750157.1
Equisetum arvense voucher AP400 (OAC) ribulose-1,5-bisphosphate carboxylase/oxygenase large subunit (rbcL) gene, partial cds; chloroplast	1122	1122	92%	0.0	100.00%	EU677105.1

Description	Max Score	Total Score	Query Cover	E value	Per. Ident	Accession
Equisetum diffusum isolate 40804 ribulose-1,5-bisphosphate carboxylase/oxygenase large subunit (rbcL) gene, partial cds; plastid	1120	1120	94%	0.0	99.35%	MH750167.1
Equisetum telmateia subsp. braunii isolate 40828 ribulose-1,5-bisphosphate carboxylase/oxygenase large subunit (rbcL) gene, partial cds; plastid	1118	1118	95%	0.0	98.73%	MH750207.1
Equisetum sylvaticum isolate 41081 ribulose-1,5-bisphosphate carboxylase/oxygenase large subunit (rbcL) gene, partial cds; plastid	1116	1116	95%	0.0	98.73%	MH750205.1
Equisetum pratense isolate 41087 ribulose-1,5-bisphosphate carboxylase/oxygenase large subunit (rbcL) gene, partial cds; plastid	1116	1116	95%	0.0	98.73%	MH750195.1
Equisetum x fontqueri isolate 26093 ribulose-1,5-bisphosphate carboxylase/oxygenase large subunit (rbcL) gene, partial cds; plastid	1114	1114	95%	0.0	98.72%	MH750221.1
Equisetum pratense voucher AP451 ribulose-1,5-bisphosphate carboxylase/oxygenase large subunit (rbcL) gene, partial cds; chloroplast	1114	1114	92%	0.0	99.67%	HQ590083.1
Equisetum fluviatile ribulose-1,5-bisphosphate carboxylase/oxygenase large subunit (rbcL) gene, partial cds; chloroplast	1114	1114	92%	0.0	99.67%	DQ463101.1
Equisetum arvense x Equisetum telmateia subsp. braunii isolate 40834 ribulose-1,5-bisphosphate carboxylase/oxygenase large subunit (rbcL) gene, partial cds; plastid	1112	1112	92%	0.0	99.67%	MH750161.1
Equisetum arvense ribulose-1,5-bisphosphate carboxylase/oxygenase large subunit (rbcL) gene, partial cds; chloroplast	1110	1110	92%	0.0	99.51%	KM218345.1
Equisetum palustre isolate 17671 ribulose-1,5-bisphosphate carboxylase/oxygenase large subunit (rbcL) gene, partial cds; plastid	1107	1107	94%	0.0	99.03%	MH750191.1
Equisetum telmateia subsp. braunii isolate 40832 ribulose-1,5-bisphosphate carboxylase/oxygenase large subunit (rbcL) gene, partial cds; plastid	1105	1105	94%	0.0	98.71%	MH750208.1
Equisetum palustre isolate 39349 ribulose-1,5-bisphosphate carboxylase/oxygenase large subunit (rbcL) gene, partial cds; plastid	1105	1105	93%	0.0	99.03%	MH750192.1
Equisetum pratense isolate 41086 ribulose-1,5-bisphosphate carboxylase/oxygenase large subunit (rbcL) gene, partial cds; plastid	1096	1096	94%	0.0	98.70%	MH750194.1
Equisetum fluviatile chloroplast rbcL gene for ribulose-1,5-bisphosphate carboxylase/oxygenase large subunit, partial cds, specimen_voucher: TNS:776384	1094	1094	91%	0.0	99.67%	AB574684.1

Description	Max Score	Total Score	Query Cover	E value	Per. Ident	Accession
Equisetum pratense isolate 39348 ribulose-1,5-bisphosphate carboxylase/oxygenase large subunit (rbcL) gene, partial cds; plastid	1092	1092	93%	0.0	98.70%	MH750193.1
Equisetum scirpoides ribulose-1,5-bisphosphate carboxylase/oxygenase large subunit (rbcL) gene, partial cds; chloroplast gene for chloroplast product	1090	1090	98%	0.0	97.20%	AY226133.1
Equisetum arvense chloroplast rbcL gene for ribulose-1,5-bisphosphate carboxylase/oxygenase large subunit, partial cds, specimen_voucher: TNS:763618	1088	1088	91%	0.0	99.50%	AB574683.1
Equisetum xylochaetum chloroplast, complete genome	1077	1077	99%	0.0	96.34%	MW282958.1
Equisetum bogotense ribulose-1,5-bisphosphate carboxylase/oxygenase large subunit (rbcL) gene, partial cds; chloroplast gene for chloroplast product	1075	1075	97%	0.0	96.88%	AY226139.1
Equisetum hyemale chloroplast, complete genome	1072	1072	99%	0.0	96.18%	KC117177.1
Equisetum palustre chloroplast rbcL gene for ribulose-1,5-bisphosphate carboxylase/oxygenase large subunit, partial cds, specimen_voucher: TNS:776981	1072	1072	91%	0.0	99.00%	AB574686.1
Equisetum scirpoides isolate 41089 ribulose-1,5-bisphosphate carboxylase/oxygenase large subunit (rbcL) gene, partial cds; plastid	1068	1068	95%	0.0	97.30%	MH750203.1
Equisetum komarovii isolate 40830 ribulose-1,5-bisphosphate carboxylase/oxygenase large subunit (rbcL) gene, partial cds; plastid	1068	1068	95%	0.0	97.30%	MH750182.1
Equisetum myriochaetum ribulose-1,5-bisphosphate carboxylase/oxygenase large subunit (rbcL) gene, partial cds; chloroplast gene for chloroplast product	1062	1062	98%	0.0	96.43%	AY226131.1
Equisetum giganteum ribulose-1,5-bisphosphate carboxylase/oxygenase large subunit (rbcL) gene, partial cds; chloroplast gene for chloroplast product	1062	1062	98%	0.0	96.43%	AY226127.1
Equisetum ramosissimum isolate BPTPS123 voucher LISE:96444 chloroplast, complete genome	1061	1061	99%	0.0	95.88%	ON641349.1
Equisetum ramosissimum isolate BPTPS056 voucher LISE:96334 chloroplast, complete genome	1061	1061	99%	0.0	95.88%	NC_062377.1
Equisetum ramosissimum isolate BPTPS056 voucher LISE:96334 chloroplast, complete genome	1061	1061	99%	0.0	95.88%	OM691674.1
Equisetum sp. Schuettpelz 1643 ribulose-1,5-bisphosphate carboxylase/oxygenase large subunit (rbcL) gene, partial cds; chloroplast	1061	1061	98%	0.0	96.42%	MW620286.1
Equisetum sylvaticum chloroplast rbcL gene for ribulose-1,5-bisphosphate carboxylase/oxygenase large subunit, partial cds, specimen_voucher: TNS:776974	1059	1059	91%	0.0	98.66%	AB574690.1

Description	Max Score	Total Score	Query Cover	E value	Per. Ident	Accession
Equisetum pratense chloroplast rbcL gene for ribulose-1,5-bisphosphate carboxylase/oxygenase large subunit, partial cds, specimen_voucher: TNS:1107843	1059	1059	91%	0.0	98.66%	AB574687.1
Equisetum laevigatum ribulose-1,5-bisphosphate carboxylase/oxygenase large subunit (rbcL) gene, partial cds; chloroplast gene for chloroplast product	1059	1059	97%	0.0	96.42%	AY226130.1
Equisetum scirpoides isolate 26090 ribulose-1,5-bisphosphate carboxylase/oxygenase large subunit (rbcL) gene, partial cds; plastid	1057	1057	95%	0.0	96.98%	MH750202.1
Equisetum hyemale subsp. affine voucher OAC 94916 ribulose-1,5-bisphosphate carboxylase/oxygenase large subunit (rbcL) gene, partial cds; chloroplast	1057	1057	98%	0.0	96.27%	KF186500.1
Equisetum hyemale subsp. affine ribulose-1,5-bisphosphate carboxylase/oxygenase large subunit (rbcL) gene, partial cds; chloroplast gene for chloroplast product	1057	1057	98%	0.0	96.27%	AY226128.1
Equisetum giganteum isolate 40806 ribulose-1,5-bisphosphate carboxylase/oxygenase large subunit (rbcL) gene, partial cds; plastid	1055	1055	97%	0.0	96.41%	MH750172.1
Equisetum giganteum isolate 26087 ribulose-1,5-bisphosphate carboxylase/oxygenase large subunit (rbcL) gene, partial cds; plastid	1055	1055	97%	0.0	96.41%	MH750170.1
Equisetum ramosissimum voucher R825 ribulose-1,5-bisphosphate carboxylase/oxygenase large subunit (rbcl) gene, partial cds; chloroplast	1055	1055	98%	0.0	95.99%	KY656709.1
Equisetum x ferrissii ribulose-1,5-bisphosphate carboxylase/oxygenase large subunit (rbcL) gene, partial cds; chloroplast	1055	1055	97%	0.0	96.41%	EU352292.1
Equisetum hyemale ribulose-1,5-bisphosphate carboxylase/oxygenase large subunit (rbcL) gene, partial cds; chloroplast	1055	1055	99%	0.0	95.73%	DQ646001.1
Equisetum x ferrissii ribulose-1,5-bisphosphate carboxylase large subunit (rbcL) gene, partial cds; chloroplast gene for chloroplast product	1053	1053	97%	0.0	96.40%	AF313579.1
Equisetum scirpoides isolate 10933 ribulose-1,5-bisphosphate carboxylase/oxygenase large subunit (rbcL) gene, partial cds; plastid	1048	1048	94%	0.0	97.25%	MH750201.1
Equisetum arvense voucher TJD-503 ribulose-1,5-bisphosphate carboxylase/oxygenase large subunit (rbcL) gene, partial cds; chloroplast	1048	1048	86%	0.0	100.00%	KJ841302.1
Equisetum arvense voucher TJD-508 ribulose-1,5-bisphosphate carboxylase/oxygenase large subunit (rbcL) gene, partial cds; chloroplast	1048	1048	86%	0.0	100.00%	KJ841301.1

Description	Max Score	Total Score	Query Cover	E value	Per. Ident	Accession
Equisetum arvense voucher TJD-504 ribulose-1,5-bisphosphate carboxylase/oxygenase large subunit (rbcL) gene, partial cds; chloroplast	1048	1048	86%	0.0	100.00%	KJ841300.1
Equisetum ramosissimum subsp. debile voucher G.M. Plunkett 3642 (NYBG) ribulose-1,5-bisphosphate carboxylase/oxygenase large subunit (rbcL) gene, partial cds; chloroplast	1046	1046	98%	0.0	95.96%	MT657901.1
Equisetum ramosissimum subsp. debile ribulose-1,5-bisphosphate carboxylase/oxygenase large subunit (rbcL) gene, partial cds; chloroplast gene for chloroplast product	1046	1046	98%	0.0	95.96%	AY226132.1
Equisetum myriochaetum isolate 40815 ribulose-1,5-bisphosphate carboxylase/oxygenase large subunit (rbcL) gene, partial cds; plastid	1042	1042	97%	0.0	96.23%	MH750185.1
Commelina diffusa voucher Q555 ribulose-1,5-bisphosphate carboxylase/oxygenase large subunit (rbcL) gene, partial cds; chloroplast	1040	1040	86%	0.0	99.82%	MH714073.1
Equisetum scirpoides voucher AP248 ribulose-1,5-bisphosphate carboxylase/oxygenase large subunit (rbcL) gene, partial cds; chloroplast	1038	1038	92%	0.0	97.53%	HQ590084.1
Equisetum bogotense isolate 40800 ribulose-1,5-bisphosphate carboxylase/oxygenase large subunit (rbcL) gene, partial cds; plastid	1037	1037	94%	0.0	96.78%	MH750162.1
Equisetum xylochaetum isolate 40614 ribulose-1,5-bisphosphate carboxylase/oxygenase large subunit (rbcL) gene, partial cds; plastid	1035	1035	95%	0.0	96.34%	MH750228.1
Equisetum myriochaetum isolate 41080 ribulose-1,5-bisphosphate carboxylase/oxygenase large subunit (rbcL) gene, partial cds; plastid	1035	1035	95%	0.0	96.34%	MH750190.1
Equisetum myriochaetum isolate 40826 ribulose-1,5-bisphosphate carboxylase/oxygenase large subunit (rbcL) gene, partial cds; plastid	1035	1035	95%	0.0	96.34%	MH750188.1
Equisetum x schaffneri isolate 40813 ribulose-1,5-bisphosphate carboxylase/oxygenase large subunit (rbcL) gene, partial cds; plastid	1033	1033	95%	0.0	96.34%	MH750224.1
Equisetum giganteum isolate 40811 ribulose-1,5-bisphosphate carboxylase/oxygenase large subunit (rbcL) gene, partial cds; plastid	1031	1031	95%	0.0	96.33%	MH750176.1
Equisetum hyemale subsp. hyemale isolate 41088 ribulose-1,5-bisphosphate carboxylase/oxygenase large subunit (rbcL) gene, partial cds; plastid	1029	1029	95%	0.0	96.18%	MH750181.1
Equisetum variegatum ribulose-1,5-bisphosphate carboxylase/oxygenase large subunit (rbcL) gene, partial cds; chloroplast gene for chloroplast product	1029	1029	98%	0.0	95.50%	AY226134.1

Description	Max Score	Total Score	Query Cover	E value	Per. Ident	Accession
Equisetum sylvaticum voucher TJD-502 ribulose-1,5-bisphosphate carboxylase/oxygenase large subunit (rbcL) gene, partial cds; chloroplast	1026	1026	86%	0.0	99.29%	KJ841305.1
Equisetum giganteum isolate 40807 ribulose-1,5-bisphosphate carboxylase/oxygenase large subunit (rbcL) gene, partial cds; plastid	1022	1022	94%	0.0	96.30%	MH750173.1
Equisetum arvense voucher WAB_1079140221 ribulose-1,5-bisphosphate carboxylase/oxygenase large subunit (rbcL) gene, partial cds; chloroplast	1022	1022	84%	0.0	100.00%	KJ593388.1
Equisetum arvense subsp. alpestre voucher Gillespie_10109_CAN ribulose-1,5-bisphosphate carboxylase/oxygenase large subunit (rbcL) gene, partial cds; chloroplast	1022	1022	84%	0.0	100.00%	KC482713.1
Equisetum bogotense isolate 40801 ribulose-1,5-bisphosphate carboxylase/oxygenase large subunit (rbcL) gene, partial cds; plastid	1020	1020	93%	0.0	96.73%	MH750163.1
Equisetum arvense ribulose-1,5-bisphosphate carboxylase/oxygenase large subunit (rbcL) gene, partial cds; chloroplast	1020	1020	84%	0.0	100.00%	MZ359289.1
Equisetum fluviatile voucher 09PROBE-05547 ribulose-1,5-bisphosphate carboxylase/oxygenase large subunit (rbcL) gene, partial cds; chloroplast	1020	1020	84%	0.0	100.00%	JN965528.1
Equisetum trachyodon isolate 41092 ribulose-1,5-bisphosphate carboxylase/oxygenase large subunit (rbcL) gene, partial cds; plastid	1018	1018	95%	0.0	96.01%	MH750227.1
Equisetum ramosissimum subsp. debile isolate 40829 ribulose-1,5-bisphosphate carboxylase/oxygenase large subunit (rbcL) gene, partial cds; plastid	1018	1018	95%	0.0	95.87%	MH750200.1
Equisetum myriochaetum isolate 40936 ribulose-1,5-bisphosphate carboxylase/oxygenase large subunit (rbcL) gene, partial cds; plastid	1018	1018	94%	0.0	96.29%	MH750189.1
Equisetum arvense voucher BIOUG24048-F01 ribulose-1,5-bisphosphate carboxylase/oxygenase large subunit (rbcL) gene, partial cds; chloroplast	1018	1018	83%	0.0	100.00%	KT695464.1
Equisetum hyemale voucher AP344 (OAC) ribulose-1,5-bisphosphate carboxylase/oxygenase large subunit (rbcL) gene, partial cds; chloroplast	1018	1018	94%	0.0	96.43%	EU677107.1
Equisetum hyemale subsp. affine isolate 40831 ribulose-1,5-bisphosphate carboxylase/oxygenase large subunit (rbcL) gene, partial cds; plastid	1016	1016	94%	0.0	96.14%	MH750177.1

Alignments

Equisetum arvense chloroplast, complete genome

Sequence ID: **GU191334.1** Length: 133309 Number of Matches: 1
Range 1: 27302 to 27956

Score	Expect	Identities	Gaps	Strand	Frame
1194 bits(646)	0.0()	652/655(99%)	0/655(0%)	Plus/Minus	

Features:

```
Query  2      GAGACTAAAGCAGGTGTTGGATTTAAAGCTGGTGTTAAAGATTATCGATTGACTTATTTT  61
              ||||| ||||||||||||||||||||||||||||||||||||||||||||||||||||||
Sbjct  27956  GAGACCAAAGCAGGTGTTGGATTTAAAGCTGGTGTTAAAGATTATCGATTGACTTATTTT  27897

Query  62     ACTCCAGATTATGAAACCAAAGATACCGATATTTTAGCAGCATTCCGTATGACTCCTCAA  121
              ||||||||||||||||||||||||||||||||||||||||||||||||||||||||||||
Sbjct  27896  ACTCCAGATTATGAAACCAAAGATACCGATATTTTAGCAGCATTCCGTATGACTCCTCAA  27837

Query  122    CCGGGAGTACCACCGGAAGAAGCAGGAGCAGCCGTAGCTGCTGAATCGTCCACGGGCACC  181
              ||||||||||||||||||||||||||||||||||||||||||||||||||||||||||||
Sbjct  27836  CCGGGAGTACCACCGGAAGAAGCAGGAGCAGCCGTAGCTGCTGAATCGTCCACGGGCACC  27777

Query  182    TGGACTACCGTATGGACAGATGGACTTACTAGTCTTGATCGATATAAAGGTCGATGCTAT  241
              ||||||||||||||||||||||||||||||||||||||||||||||||||||||||||||
Sbjct  27776  TGGACTACCGTATGGACAGATGGACTTACTAGTCTTGATCGATATAAAGGTCGATGCTAT  27717

Query  242    AATATTGAACCTGTTGCTGGAGAAGATAATCAATTCATAGCTTATGTAGCCTACCCTTTA  301
              ||||||||||||||||||||||||||||||||||||||||||||||||||||||||||||
Sbjct  27716  AATATTGAACCTGTTGCTGGAGAAGATAATCAATTCATAGCTTATGTAGCCTACCCTTTA  27657

Query  302    GATCTTTTTGAAGAAGGTTCTGTTACCAATCTTTTTACTTCAATTGTTGGTAATGTTTTC  361
              ||||||||||||||||||||||||||||||||||||||||||||||||||||||||||||
Sbjct  27656  GATCTTTTTGAAGAAGGTTCTGTTACCAATCTTTTTACTTCAATTGTTGGTAATGTTTTC  27597

Query  362    GGCTTCAAAGCTCTACGTGCTTTACGTTTAGAAGATTTAAGAATTCCTCCTGCTTACTCC  421
              ||||||||||||||||||||||||||||||||||||||||||||||||||||||||||||
Sbjct  27596  GGCTTCAAAGCTCTACGTGCTTTACGTTTAGAAGATTTAAGAATTCCTCCTGCTTACTCC  27537

Query  422    AAAACTTTTATAGGACCACCCCACGGTATCCAGGTTGAAAGAGATAAGTTAAACAAATAC  481
              ||||||||||||||||||||||||||||||||||||||||||||||||||||||||||||
Sbjct  27536  AAAACTTTTATAGGACCACCCCACGGTATCCAGGTTGAAAGAGATAAGTTAAACAAATAC  27477

Query  482    GGTCGTCCTTTATTAGGTTGTACAATTAAACCAAAATTGGGACTATCTGCTAAAAACTAT  541
              ||||||||||||||||||||||||||||||||||||||||||||||||||||||||||||
Sbjct  27476  GGTCGTCCTTTATTAGGTTGTACAATTAAACCAAAATTGGGACTATCTGCTAAAAACTAT  27417

Query  542    GGTAGAGCTGTTTATGAATGTCTTCGTGGTGGGCTTGATTTCACCAAAGATGATGAGAAT  601
              ||||||||||||||||||||||||||||||||||||||||||||||||||||||||||||
Sbjct  27416  GGTAGAGCTGTTTATGAATGTCTTCGTGGTGGGCTTGATTTCACCAAAGATGATGAGAAT  27357

Query  602    GTAAACTCTCAACCCTTTATGCGTTGGAGAGATCGATTCTTATTTGTAGCAGAAG  656
              ||||||||||||||||||||||||||||||||||| |||||||| ||||||||||
Sbjct  27356  GTAAACTCTCAACCCTTTATGCGTTGGAGAGATCGTTTCTTATTCGTAGCAGAAG  27302
```

Equisetum arvense chloroplast ribulosebisphosphate carboxylase (rbcL) gene, complete cds
Sequence ID: **L11053.1** Length: 1587 Number of Matches: 1
Range 1: 88 to 742

Score	Expect	Identities	Gaps	Strand	Frame
1194 bits(646)	0.0()	652/655(99%)	0/655(0%)	Plus/Plus	

Features:

```
Query  2    GAGACTAAAGCAGGTGTTGGATTTAAAGCTGGTGTTAAAGATTATCGATTGACTTATTTT  61
            ||||| ||||||||||||||||||||||||||||||||||||||||||||||||||||||
Sbjct  88   GAGACCAAAGCAGGTGTTGGATTTAAAGCTGGTGTTAAAGATTATCGATTGACTTATTTT  147

Query  62   ACTCCAGATTATGAAACCAAAGATACCGATATTTTAGCAGCATTCCGTATGACTCCTCAA  121
            ||||||||||||||||||||||||||||||||||||||||||||||||||||||||||||
Sbjct  148  ACTCCAGATTATGAAACCAAAGATACCGATATTTTAGCAGCATTCCGTATGACTCCTCAA  207

Query  122  CCGGGAGTACCACCGGAAGAAGCAGGAGCAGCCGTAGCTGCTGAATCGTCCACGGGCACC  181
            ||||||||||||||||||||||||||||||||||||||||||||||||||||||||||||
Sbjct  208  CCGGGAGTACCACCGGAAGAAGCAGGAGCAGCCGTAGCTGCTGAATCGTCCACGGGCACC  267

Query  182  TGGACTACCGTATGGACAGATGGACTTACTAGTCTTGATCGATATAAAGGTCGATGCTAT  241
            ||||||||||||||||||||||||||||||||||||||||||||||||||||||||||||
Sbjct  268  TGGACTACCGTATGGACAGATGGACTTACTAGTCTTGATCGATATAAAGGTCGATGCTAT  327

Query  242  AATATTGAACCTGTTGCTGGAGAAGATAATCAATTCATAGCTTATGTAGCCTACCCTTTA  301
            ||||||||||||||||||||||||||||||||||||||||||||||||||||||||||||
Sbjct  328  AATATTGAACCTGTTGCTGGAGAAGATAATCAATTCATAGCTTATGTAGCCTACCCTTTA  387

Query  302  GATCTTTTTGAAGAAGGTTCTGTTACCAATCTTTTTACTTCAATTGTTGGTAATGTTTTC  361
            ||||||||||||||||||||||||||||||||||||||||||||||||||||||||||||
Sbjct  388  GATCTTTTTGAAGAAGGTTCTGTTACCAATCTTTTTACTTCAATTGTTGGTAATGTTTTC  447
```

```
Query  362    GGCTTCAAAGCTCTACGTGCTTTACGTTTAGAAGATTTAAGAATTCCTCCTGCTTACTCC  421
              ||||||||||||||||||||||||||||||||||||||||||||||||||||||||||||
Sbjct  448    GGCTTCAAAGCTCTACGTGCTTTACGTTTAGAAGATTTAAGAATTCCTCCTGCTTACTCC  507

Query  422    AAAACTTTTATAGGACCACCCCACGGTATCCAGGTTGAAAGAGATAAGTTAAACAAATAC  481
              ||||||||||||||||||||||||||||||||||||||||||||||||||||||||||||
Sbjct  508    AAAACTTTTATAGGACCACCCCACGGTATCCAGGTTGAAAGAGATAAGTTAAACAAATAC  567

Query  482    GGTCGTCCTTTATTAGGTTGTACAATTAAACCAAAATTGGGACTATCTGCTAAAAACTAT  541
              ||||||||||||||||||||||||||||||||||||||||||||||||||||||||||||
Sbjct  568    GGTCGTCCTTTATTAGGTTGTACAATTAAACCAAAATTGGGACTATCTGCTAAAAACTAT  627

Query  542    GGTAGAGCTGTTTATGAATGTCTTCGTGGTGGGCTTGATTTCACCAAAGATGATGAGAAT  601
              ||||||||||||||||||||||||||||||||||||||||||||||||||||||||||||
Sbjct  628    GGTAGAGCTGTTTATGAATGTCTTCGTGGTGGGCTTGATTTCACCAAAGATGATGAGAAT  687

Query  602    GTAAACTCTCAACCCTTTATGCGTTGGAGAGATCGATTCTTATTTGTAGCAGAAG  656
              ||||||||||||||||||||||||||||||||||| |||||||| ||||||||||
Sbjct  688    GTAAACTCTCAACCCTTTATGCGTTGGAGAGATCGTTTCTTATTCGTAGCAGAAG  742
```

Equisetum arvense plastid, complete genome

Sequence ID: **JN968380.1** Length: 132726 Number of Matches: 1

Range 1: 64973 to 65627

**Score**	**Expect**	**Identities**	**Gaps**	**Strand**	**Frame**
1188 bits(643)	0.0()	651/655(99%)	0/655(0%)	Plus/Plus	

Features:

```
Query  2      GAGACTAAAGCAGGTGTTGGATTTAAAGCTGGTGTTAAAGATTATCGATTGACTTATTTT  61
              ||||| ||||||||||||||||||||||||||||||||||||||||||||||||||||||
Sbjct  64973  GAGACCAAAGCAGGTGTTGGATTTAAAGCTGGTGTTAAAGATTATCGATTGACTTATTTT  65032

Query  62     ACTCCAGATTATGAAACCAAAGATACCGATATTTTAGCAGCATTCCGTATGACTCCTCAA  121
              ||||||||||||||||||||||||||||||||||||||||||||||||||||||||||||
Sbjct  65033  ACTCCAGATTATGAAACCAAAGATACCGATATTTTAGCAGCATTCCGTATGACTCCTCAA  65092

Query  122    CCGGGAGTACCACCGGAAGAAGCAGGAGCAGCCGTAGCTGCTGAATCGTCCACGGGCACC  181
              ||||||||||||||||||||||||||||||||||||||||||||||||||||||||||||
Sbjct  65093  CCGGGAGTACCACCGGAAGAAGCAGGAGCAGCCGTAGCTGCTGAATCGTCCACGGGCACC  65152

Query  182    TGGACTACCGTATGGACAGATGGACTTACTAGTCTTGATCGATATAAAGGTCGATGCTAT  241
              ||||||||||||||||||||||||||||||||||||||||||||||||||||||||||||
Sbjct  65153  TGGACTACCGTATGGACAGATGGACTTACTAGTCTTGATCGATATAAAGGTCGATGCTAT  65212

Query  242    AATATTGAACCTGTTGCTGGAGAAGATAATCAATTCATAGCTTATGTAGCCTACCCTTTA  301
              ||||||||||||||||||||||||||||||||||||||||||||||||||||||||||||
Sbjct  65213  AATATTGAACCTGTTGCTGGAGAAGATAATCAATTCATAGCTTATGTAGCCTACCCTTTA  65272

Query  302    GATCTTTTTGAAGAAGGTTCTGTTACCAATCTTTTTACTTCAATTGTTGGTAATGTTTTC  361
              ||||||||||||||||||||||||||||||||||||||||||||||||||||||||||||
Sbjct  65273  GATCTTTTTGAAGAAGGTTCTGTTACCAATCTTTTTACTTCAATTGTTGGTAATGTTTTC  65332

Query  362    GGCTTCAAAGCTCTACGTGCTTTACGTTTAGAAGATTTAAGAATTCCTCCTGCTTACTCC  421
              || |||||||||||||||||||||||||||||||||||||||||||||||||||||||||
Sbjct  65333  GGATTCAAAGCTCTACGTGCTTTACGTTTAGAAGATTTAAGAATTCCTCCTGCTTACTCC  65392

Query  422    AAAACTTTTATAGGACCACCCCACGGTATCCAGGTTGAAAGAGATAAGTTAAACAAATAC  481
              ||||||||||||||||||||||||||||||||||||||||||||||||||||||||||||
Sbjct  65393  AAAACTTTTATAGGACCACCCCACGGTATCCAGGTTGAAAGAGATAAGTTAAACAAATAC  65452

Query  482    GGTCGTCCTTTATTAGGTTGTACAATTAAACCAAAATTGGGACTATCTGCTAAAAACTAT  541
              ||||||||||||||||||||||||||||||||||||||||||||||||||||||||||||
Sbjct  65453  GGTCGTCCTTTATTAGGTTGTACAATTAAACCAAAATTGGGACTATCTGCTAAAAACTAT  65512

Query  542    GGTAGAGCTGTTTATGAATGTCTTCGTGGTGGGCTTGATTTCACCAAAGATGATGAGAAT  601
              ||||||||||||||||||||||||||||||||||||||||||||||||||||||||||||
Sbjct  65513  GGTAGAGCTGTTTATGAATGTCTTCGTGGTGGGCTTGATTTCACCAAAGATGATGAGAAT  65572

Query  602    GTAAACTCTCAACCCTTTATGCGTTGGAGAGATCGATTCTTATTTGTAGCAGAAG  656
              ||||||||||||||||||||||||||||||||||| |||||||| ||||||||||
Sbjct  65573  GTAAACTCTCAACCCTTTATGCGTTGGAGAGATCGTTTCTTATTCGTAGCAGAAG  65627
```

Equisetum environmental sample isolate EDNA16-0043490 ribulose-1,5-bisphosphate carboxylase/oxygenase large subunit (rbcL) gene, partial cds; chloroplast

Sequence ID: **MT197555.1** Length: 697 Number of Matches: 1

Range 1: 1 to 644

**Score**	**Expect**	**Identities**	**Gaps**	**Strand**	**Frame**

1179 bits(638) 0.0() 642/644(99%) 0/644(0%) Plus/Plus

Features:

```
Query  13   AGGTGTTGGATTTAAAGCTGGTGTTAAAGATTATCGATTGACTTATTTTACTCCAGATTA  72
            ||||||||||||||||||||||||||||||||||||||||||||||||||||||||||||
Sbjct  1    AGGTGTTGGATTTAAAGCTGGTGTTAAAGATTATCGATTGACTTATTTTACTCCAGATTA  60

Query  73   TGAAACCAAAGATACCGATATTTTAGCAGCATTCCGTATGACTCCTCAACCGGGAGTACC  132
            ||||||||||||||||||||||||||||||||||||||||||||||||||||||||||||
Sbjct  61   TGAAACCAAAGATACCGATATTTTAGCAGCATTCCGTATGACTCCTCAACCGGGAGTACC  120

Query  133  ACCGGAAGAAGCAGGAGCAGCCGTAGCTGCTGAATCGTCCACGGGCACCTGGACTACCGT  192
            ||||||||||||||||||||||||||||||||||||||||||||||||||||||||||||
Sbjct  121  ACCGGAAGAAGCAGGAGCAGCCGTAGCTGCTGAATCGTCCACGGGCACCTGGACTACCGT  180

Query  193  ATGGACAGATGGACTTACTAGTCTTGATCGATATAAAGGTCGATGCTATAATATTGAACC  252
            ||||||||||||||||||||||||||||||||||||||||||||||||||||||||||||
Sbjct  181  ATGGACAGATGGACTTACTAGTCTTGATCGATATAAAGGTCGATGCTATAATATTGAACC  240

Query  253  TGTTGCTGGAGAAGATAATCAATTCATAGCTTATGTAGCCTACCCTTTAGATCTTTTTGA  312
            ||||||||||||||||||||||||||||||||||||||||||||||||||||||||||||
Sbjct  241  TGTTGCTGGAGAAGATAATCAATTCATAGCTTATGTAGCCTACCCTTTAGATCTTTTTGA  300

Query  313  AGAAGGTTCTGTTACCAATCTTTTTACTTCAATTGTTGGTAATGTTTTCGGCTTCAAAGC  372
            ||||||||||||||||||||||||||||||||||||||||||||||||||||||||||||
Sbjct  301  AGAAGGTTCTGTTACCAATCTTTTTACTTCAATTGTTGGTAATGTTTTCGGCTTCAAAGC  360

Query  373  TCTACGTGCTTTACGTTTAGAAGATTTAAGAATTCCTCCTGCTTACTCCAAAACTTTTAT  432
            ||||||||||||||||||||||||||||||||||||||||||||||||||||||||||||
Sbjct  361  TCTACGTGCTTTACGTTTAGAAGATTTAAGAATTCCTCCTGCTTACTCCAAAACTTTTAT  420

Query  433  AGGACCACCCCACGGTATCCAGGTTGAAAGAGATAAGTTAAACAAATACGGTCGTCCTTT  492
            ||||||||||||||||||||||||||||||||||||||||||||||||||||||||||||
Sbjct  421  AGGACCACCCCACGGTATCCAGGTTGAAAGAGATAAGTTAAACAAATACGGTCGTCCTTT  480

Query  493  ATTAGGTTGTACAATTAAACCAAAATTGGGACTATCTGCTAAAAACTATGGTAGAGCTGT  552
            ||||||||||||||||||||||||||||||||||||||||||||||||||||||||||||
Sbjct  481  ATTAGGTTGTACAATTAAACCAAAATTGGGACTATCTGCTAAAAACTATGGTAGAGCTGT  540

Query  553  TTATGAATGTCTTCGTGGTGGGCTTGATTTCACCAAAGATGATGAGAATGTAAACTCTCA  612
            ||||||||||||||||||||||||||||||||||||||||||||||||||||||||||||
Sbjct  541  TTATGAATGTCTTCGTGGTGGGCTTGATTTCACCAAAGATGATGAGAATGTAAACTCTCA  600

Query  613  ACCCTTTATGCGTTGGAGAGATCGATTCTTATTTGTAGCAGAAG  656
            |||||||||||||||||||||||| |||||||| ||||||||||
Sbjct  601  ACCCTTTATGCGTTGGAGAGATCGTTTCTTATTCGTAGCAGAAG  644
```

Equisetum fluviatile ribulose-1,5-bisphosphate carboxylase/oxygenase large subunit (rbcL) gene, partial cds; chloroplast gene for chloroplast product

Sequence ID: **AY226142.1** Length: 1325 Number of Matches: 1

Range 1: 1 to 644

Score	Expect	Identities	Gaps	Strand	Frame
1179 bits(638)	0.0()	642/644(99%)	0/644(0%)	Plus/Plus	

Features:

```
Query  13   AGGTGTTGGATTTAAAGCTGGTGTTAAAGATTATCGATTGACTTATTTTACTCCAGATTA  72
            ||||||||||||||||||||||||||||||||||||||||||||||||||||||||||||
Sbjct  1    AGGTGTTGGATTTAAAGCTGGTGTTAAAGATTATCGATTGACTTATTTTACTCCAGATTA  60

Query  73   TGAAACCAAAGATACCGATATTTTAGCAGCATTCCGTATGACTCCTCAACCGGGAGTACC  132
            ||||||||||||||||||||||||||||||||||||||||||||||||||||||||||||
Sbjct  61   TGAAACCAAAGATACCGATATTTTAGCAGCATTCCGTATGACTCCTCAACCGGGAGTACC  120

Query  133  ACCGGAAGAAGCAGGAGCAGCCGTAGCTGCTGAATCGTCCACGGGCACCTGGACTACCGT  192
            ||||||||||||||||||||||||||||||||||||||||||||||||||||||||||||
Sbjct  121  ACCGGAAGAAGCAGGAGCAGCCGTAGCTGCTGAATCGTCCACGGGCACCTGGACTACCGT  180

Query  193  ATGGACAGATGGACTTACTAGTCTTGATCGATATAAAGGTCGATGCTATAATATTGAACC  252
            ||||||||||||||||||||||||||||||||||||||||||||||||||||||||||||
Sbjct  181  ATGGACAGATGGACTTACTAGTCTTGATCGATATAAAGGTCGATGCTATAATATTGAACC  240

Query  253  TGTTGCTGGAGAAGATAATCAATTCATAGCTTATGTAGCCTACCCTTTAGATCTTTTTGA  312
            ||||||||||||||||||||||||||||||||||||||||||||||||||||||||||||
Sbjct  241  TGTTGCTGGAGAAGATAATCAATTCATAGCTTATGTAGCCTACCCTTTAGATCTTTTTGA  300

Query  313  AGAAGGTTCTGTTACCAATCTTTTTACTTCAATTGTTGGTAATGTTTTCGGCTTCAAAGC  372
            ||||||||||||||||||||||||||||||||||||||||||||||||||||||||||||
Sbjct  301  AGAAGGTTCTGTTACCAATCTTTTTACTTCAATTGTTGGTAATGTTTTCGGCTTCAAAGC  360

Query  373  TCTACGTGCTTTACGTTTAGAAGATTTAAGAATTCCTCCTGCTTACTCCAAAACTTTTAT  432
            ||||||||||||||||||||||||||||||||||||||||||||||||||||||||||||
Sbjct  361  TCTACGTGCTTTACGTTTAGAAGATTTAAGAATTCCTCCTGCTTACTCCAAAACTTTTAT  420
```

```
Query  433   AGGACCACCCCACGGTATCCAGGTTGAAAGAGATAAGTTAAACAAATACGGTCGTCCTTT  492
             ||||||||||||||||||||||||||||||||||||||||||||||||||||||||||||
Sbjct  421   AGGACCACCCCACGGTATCCAGGTTGAAAGAGATAAGTTAAACAAATACGGTCGTCCTTT  480

Query  493   ATTAGGTTGTACAATTAAACCAAAATTGGGACTATCTGCTAAAAACTATGGTAGAGCTGT  552
             ||||||||||||||||||||||||||||||||||||||||||||||||||||||||||||
Sbjct  481   ATTAGGTTGTACAATTAAACCAAAATTGGGACTATCTGCTAAAAACTATGGTAGAGCTGT  540

Query  553   TTATGAATGTCTTCGTGGTGGGCTTGATTTCACCAAAGATGATGAGAATGTAAACTCTCA  612
             ||||||||||||||||||||||||||||||||||||||||||||||||||||||||||||
Sbjct  541   TTATGAATGTCTTCGTGGTGGGCTTGATTTCACCAAAGATGATGAGAATGTAAACTCTCA  600

Query  613   ACCCTTTATGCGTTGGAGAGATCGATTCTTATTTGTAGCAGAAG  656
             |||||||||||||||||||||||| |||||||| ||||||||||
Sbjct  601   ACCCTTTATGCGTTGGAGAGATCGTTTCTTATTCGTAGCAGAAG  644
```


BLAST is a registered trademark of the National Library of Medicine

Support center Mailing list YouTube

- National Library Of Medicine
- National Institutes Of Health
- U.S. Department of Health & Human Services
- USA.gov

NCBI

National Center for Biotechnology Information, U.S. National Library of Medicine 8600 Rockville Pike, Bethesda MD, 20894 USA

Policies and Guidelines | Contact

HHS Vulnerability Disclosure
