# Supplementary material for: A Multi-Pronged Technique for Identifying Equisetum palustre and Equisetum arvense—Combining HPTLC, HPLC-ESI-MS/MS and Optimized DNA Barcoding Techniques
Source: Plants (Basel). 2022 Sep 28;11(19):2562. doi: 10.3390/plants11192562 (PMC9573248; doi:10.3390/plants11192562)
Supplement: Supplementary file 1 [file plants-11-02562-s001.zip › plants-1932149 supplementary/Horsetail Supplementary Figures/Figure S33 (A4, 82702220323-1).pdf]

BLAST Results

Questions/comments

Job title: Nucleotide Sequence

**RID**	J1DBCX25013 (Expires on 09-15 00:53 am)		
**Query ID**	lcl|Query_3131	**Database Name**	nt
**Description**	None	**Description**	Nucleotide collection (nt)
**Molecule type**	dna	**Program**	BLASTN 2.13.0+
**Query Length**	540		

Graphic Summary

Distribution of the top 100 Blast Hits on 100 subject sequences

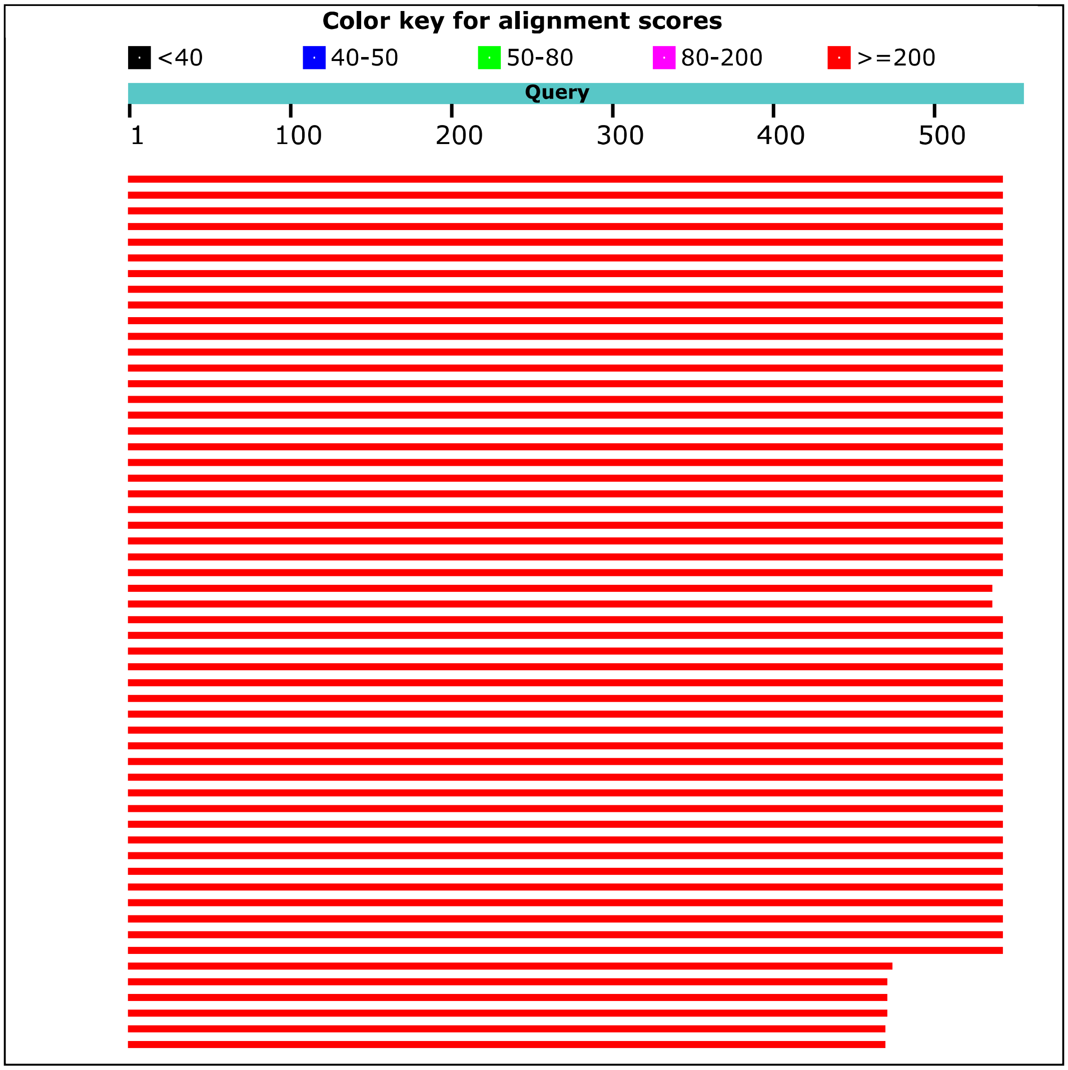

Descriptions

Sequences producing significant alignments:

Description	Max Score	Total Score	Query Cover	E value	Per. Ident	Accession
Equisetum x litorale isolate 41084 ribulose-1,5-bisphosphate carboxylase/oxygenase large subunit (rbcL) gene, partial cds; plastid	852	852	99%	0.0	95.91%	MH750222.1
Equisetum x dycei isolate 26083 ribulose-1,5-bisphosphate carboxylase/oxygenase large subunit (rbcL) gene, partial cds; plastid	852	852	99%	0.0	95.91%	MH750220.1
Equisetum fluviatile isolate 41076 ribulose-1,5-bisphosphate carboxylase/oxygenase large subunit (rbcL) gene, partial cds; plastid	852	852	99%	0.0	95.91%	MH750169.1
Equisetum fluviatile isolate 41075 ribulose-1,5-bisphosphate carboxylase/oxygenase large subunit (rbcL) gene, partial cds; plastid	852	852	99%	0.0	95.91%	MH750168.1
Equisetum arvense x Equisetum telmateia subsp. braunii isolate 40834 ribulose-1,5-bisphosphate carboxylase/oxygenase large subunit (rbcL) gene, partial cds; plastid	852	852	99%	0.0	95.91%	MH750161.1
Equisetum arvense subsp. boreale isolate 41074 ribulose-1,5-bisphosphate carboxylase/oxygenase large subunit (rbcL) gene, partial cds; plastid	852	852	99%	0.0	95.91%	MH750160.1
Equisetum arvense subsp. boreale isolate 41073 ribulose-1,5-bisphosphate carboxylase/oxygenase large subunit (rbcL) gene, partial cds; plastid	852	852	99%	0.0	95.91%	MH750159.1
Equisetum arvense subsp. arvense isolate 41071 ribulose-1,5-bisphosphate carboxylase/oxygenase large subunit (rbcL) gene, partial cds; plastid	852	852	99%	0.0	95.91%	MH750158.1
Equisetum arvense subsp. arvense isolate 40833 ribulose-1,5-bisphosphate carboxylase/oxygenase large subunit (rbcL) gene, partial cds; plastid	852	852	99%	0.0	95.91%	MH750157.1
Equisetum arvense subsp. arvense isolate 26085 ribulose-1,5-bisphosphate carboxylase/oxygenase large subunit (rbcL) gene, partial cds; plastid	852	852	99%	0.0	95.91%	MH750156.1
Equisetum arvense subsp. arvense isolate 26084 ribulose-1,5-bisphosphate carboxylase/oxygenase large subunit (rbcL) gene, partial cds; plastid	852	852	99%	0.0	95.91%	MH750155.1
Equisetum environmental sample isolate EDNA16-0043490 ribulose-1,5-bisphosphate carboxylase/oxygenase large subunit (rbcL) gene, partial cds; chloroplast	852	852	99%	0.0	95.91%	MT197555.1
Equisetum fluviatile chloroplast rbcL gene for ribulose-1,5-bisphosphate carboxylase/oxygenase large subunit, partial cds, specimen_voucher: TNS:776384	852	852	99%	0.0	95.91%	AB574684.1

Description	Max Score	Total Score	Query Cover	E value	Per. Ident	Accession
Equisetum arvense chloroplast, complete genome	852	852	99%	0.0	95.91%	GU191334.1
Equisetum arvense voucher JAG288 (OAC) ribulose-1,5-bisphosphate carboxylase/oxygenase large subunit (rbcL) gene, partial cds; chloroplast	852	852	99%	0.0	95.91%	EU677106.1
Equisetum fluviatile ribulose-1,5-bisphosphate carboxylase/oxygenase large subunit (rbcL) gene, partial cds; chloroplast	852	852	99%	0.0	95.91%	DQ463101.1
Equisetum fluviatile ribulose-1,5-bisphosphate carboxylase/oxygenase large subunit (rbcL) gene, partial cds; chloroplast gene for chloroplast product	852	852	99%	0.0	95.91%	AY226142.1
Equisetum arvense ribulose-1,5-bisphosphate carboxylase/oxygenase large subunit (rbcL) gene, partial cds; chloroplast gene for chloroplast product	852	852	99%	0.0	95.91%	AY226140.1
Equisetum arvense chloroplast ribulosebisphosphate carboxylase (rbcL) gene, complete cds	852	852	99%	0.0	95.91%	L11053.1
Equisetum diffusum isolate 40689 ribulose-1,5-bisphosphate carboxylase/oxygenase large subunit (rbcL) gene, partial cds; plastid	846	846	99%	0.0	95.72%	MH750166.1
Equisetum arvense ribulose-1,5-bisphosphate carboxylase/oxygenase large subunit (rbcL) gene, partial cds; chloroplast	846	846	99%	0.0	95.72%	KM218345.1
Equisetum environmental sample isolate EDNA16-0043491b ribulose-1,5-bisphosphate carboxylase/oxygenase large subunit (rbcL) gene, partial cds; chloroplast	846	846	99%	0.0	95.72%	MT197548.1
Equisetum arvense plastid, complete genome	846	846	99%	0.0	95.72%	JN968380.1
Equisetum arvense chloroplast rbcL gene for ribulose-1,5-bisphosphate carboxylase/oxygenase large subunit, partial cds, specimen_voucher: TNS:763618	846	846	99%	0.0	95.72%	AB574683.1
Equisetum diffusum isolate 40804 ribulose-1,5-bisphosphate carboxylase/oxygenase large subunit (rbcL) gene, partial cds; plastid	841	841	99%	0.0	95.54%	MH750167.1
Equisetum diffusum ribulose-1,5-bisphosphate carboxylase/oxygenase large subunit (rbcL) gene, partial cds; chloroplast gene for chloroplast product	841	841	99%	0.0	95.54%	AY226141.1
Equisetum pratense voucher AP451 ribulose-1,5-bisphosphate carboxylase/oxygenase large subunit (rbcL) gene, partial cds; chloroplast	839	839	98%	0.0	95.86%	HQ590083.1
Equisetum arvense voucher AP400 (OAC) ribulose-1,5-bisphosphate carboxylase/oxygenase large subunit (rbcL) gene, partial cds; chloroplast	839	839	98%	0.0	95.86%	EU677105.1

Description	Max Score	Total Score	Query Cover	E value	Per. Ident	Accession
Equisetum palustre isolate 39349 ribulose-1,5-bisphosphate carboxylase/oxygenase large subunit (rbcL) gene, partial cds; plastid	830	830	99%	0.0	95.17%	MH750192.1
Equisetum palustre isolate 17671 ribulose-1,5-bisphosphate carboxylase/oxygenase large subunit (rbcL) gene, partial cds; plastid	830	830	99%	0.0	95.17%	MH750191.1
Equisetum palustre chloroplast, partial genome	830	830	99%	0.0	95.17%	MT984458.1
Equisetum palustre chloroplast rbcL gene for ribulose-1,5-bisphosphate carboxylase/oxygenase large subunit, partial cds, specimen_voucher: TNS:776981	830	830	99%	0.0	95.17%	AB574686.1
Equisetum palustre ribulose-1,5-bisphosphate carboxylase/oxygenase large subunit (rbcL) gene, partial cds; chloroplast gene for chloroplast product	830	830	99%	0.0	95.17%	AY226138.1
Equisetum x fontqueri isolate 26093 ribulose-1,5-bisphosphate carboxylase/oxygenase large subunit (rbcL) gene, partial cds; plastid	824	824	99%	0.0	94.98%	MH750221.1
Equisetum telmateia subsp. telmateia isolate 11642 ribulose-1,5-bisphosphate carboxylase/oxygenase large subunit (rbcL) gene, partial cds; plastid	824	824	99%	0.0	94.98%	MH750210.1
Equisetum telmateia subsp. braunii isolate 40836 ribulose-1,5-bisphosphate carboxylase/oxygenase large subunit (rbcL) gene, partial cds; plastid	824	824	99%	0.0	94.98%	MH750209.1
Equisetum telmateia subsp. braunii isolate 40832 ribulose-1,5-bisphosphate carboxylase/oxygenase large subunit (rbcL) gene, partial cds; plastid	824	824	99%	0.0	94.98%	MH750208.1
Equisetum telmateia subsp. braunii isolate 40828 ribulose-1,5-bisphosphate carboxylase/oxygenase large subunit (rbcL) gene, partial cds; plastid	824	824	99%	0.0	94.98%	MH750207.1
Equisetum telmateia subsp. braunii isolate 40817 ribulose-1,5-bisphosphate carboxylase/oxygenase large subunit (rbcL) gene, partial cds; plastid	824	824	99%	0.0	94.98%	MH750206.1
Equisetum sylvaticum isolate 41081 ribulose-1,5-bisphosphate carboxylase/oxygenase large subunit (rbcL) gene, partial cds; plastid	824	824	99%	0.0	94.98%	MH750205.1
Equisetum sylvaticum isolate 11640 ribulose-1,5-bisphosphate carboxylase/oxygenase large subunit (rbcL) gene, partial cds; plastid	824	824	99%	0.0	94.98%	MH750204.1
Equisetum sylvaticum chloroplast rbcL gene for ribulose-1,5-bisphosphate carboxylase/oxygenase large subunit, partial cds, specimen_voucher: TNS:776974	824	824	99%	0.0	94.98%	AB574690.1
Equisetum sylvaticum ribulose-1,5-bisphosphate carboxylase/oxygenase large subunit (rbcL) gene, partial cds; chloroplast gene for chloroplast product	824	824	99%	0.0	94.98%	AY226136.1

Description	Max Score	Total Score	Query Cover	E value	Per. Ident	Accession
Equisetum telmateia subsp. braunii ribulose-1,5-bisphosphate carboxylase/oxygenase large subunit (rbcL) gene, partial cds; chloroplast gene for chloroplast product	824	824	99%	0.0	94.98%	AY226135.1
Equisetum telmateia ribulose-1,5-bisphosphate carboxylase large subunit (rbcL) gene, partial cds; chloroplast gene for chloroplast product	824	824	99%	0.0	94.98%	AF313580.1
Equisetum pratense isolate 41087 ribulose-1,5-bisphosphate carboxylase/oxygenase large subunit (rbcL) gene, partial cds; plastid	819	819	99%	0.0	94.80%	MH750195.1
Equisetum pratense isolate 41086 ribulose-1,5-bisphosphate carboxylase/oxygenase large subunit (rbcL) gene, partial cds; plastid	819	819	99%	0.0	94.80%	MH750194.1
Equisetum pratense isolate 39348 ribulose-1,5-bisphosphate carboxylase/oxygenase large subunit (rbcL) gene, partial cds; plastid	819	819	99%	0.0	94.80%	MH750193.1
Equisetum pratense chloroplast rbcL gene for ribulose-1,5-bisphosphate carboxylase/oxygenase large subunit, partial cds, specimen_voucher: TNS:1107843	819	819	99%	0.0	94.80%	AB574687.1
Equisetum pratense ribulose-1,5-bisphosphate carboxylase/oxygenase large subunit (rbcL) gene, partial cds; chloroplast gene for chloroplast product	819	819	99%	0.0	94.80%	AY226137.1
Equisetum arvense isolate YYZW2016DF32-3 ribulose-1,5-bisphosphate carboxylase/oxygenase large subunit (rbcL) gene, partial cds; chloroplast	798	798	87%	0.0	97.66%	KY014938.1
Equisetum arvense voucher TJD-503 ribulose-1,5-bisphosphate carboxylase/oxygenase large subunit (rbcL) gene, partial cds; chloroplast	798	798	86%	0.0	97.86%	KJ841302.1
Equisetum arvense voucher TJD-508 ribulose-1,5-bisphosphate carboxylase/oxygenase large subunit (rbcL) gene, partial cds; chloroplast	798	798	86%	0.0	97.86%	KJ841301.1
Equisetum arvense voucher TJD-504 ribulose-1,5-bisphosphate carboxylase/oxygenase large subunit (rbcL) gene, partial cds; chloroplast	798	798	86%	0.0	97.86%	KJ841300.1
Equisetum arvense subsp. alpestre voucher Gillespie_10109_CAN ribulose-1,5-bisphosphate carboxylase/oxygenase large subunit (rbcL) gene, partial cds; chloroplast	797	797	86%	0.0	97.85%	KC482713.1
Equisetum arvense voucher WAB_1079140221 ribulose-1,5-bisphosphate carboxylase/oxygenase large subunit (rbcL) gene, partial cds; chloroplast	795	795	86%	0.0	97.85%	KJ593388.1
Equisetum arvense voucher G00199177 ribulose-1,5-bisphosphate carboxylase/oxygenase large subunit (rbcL) gene, partial cds; chloroplast	795	795	86%	0.0	97.85%	KJ204336.1

Description	Max Score	Total Score	Query Cover	E value	Per. Ident	Accession
Equisetum fluviatile voucher 09PROBE-05547 ribulose-1,5-bisphosphate carboxylase/oxygenase large subunit (rbcL) gene, partial cds; chloroplast	795	795	86%	0.0	97.85%	JN965528.1
Equisetum sylvaticum plastid partial rbcL gene for ribulose-1,5-biphosphate carboxylase/oxygenase large subunit, specimen voucher H823764	795	795	89%	0.0	96.69%	HE574610.1
Equisetum arvense isolate YYZW2016DF32-1 ribulose-1,5-bisphosphate carboxylase/oxygenase large subunit (rbcL) gene, partial cds; chloroplast	793	793	86%	0.0	97.64%	KY014936.1
Equisetum arvense ribulose-1,5-bisphosphate carboxylase/oxygenase large subunit (rbcL) gene, partial cds; chloroplast	793	793	85%	0.0	97.84%	MZ359289.1
Equisetum arvense voucher BIOUG24048-F01 ribulose-1,5-bisphosphate carboxylase/oxygenase large subunit (rbcL) gene, partial cds; chloroplast	791	791	85%	0.0	97.84%	KT695464.1
Commelina diffusa voucher Q555 ribulose-1,5-bisphosphate carboxylase/oxygenase large subunit (rbcL) gene, partial cds; chloroplast	791	791	86%	0.0	97.64%	MH714073.1
Equisetum sylvaticum isolate YYZW2016JC33-1 ribulose-1,5-bisphosphate carboxylase/oxygenase large subunit (rbcL) gene, partial cds; chloroplast	782	782	87%	0.0	96.83%	KY014946.1
Equisetum telmateia voucher CCDB-18287-A07 ribulose-1,5-bisphosphate carboxylase/oxygenase large subunit (rbcL) gene, partial cds; chloroplast	778	778	86%	0.0	97.20%	MG215015.1
Equisetum palustre voucher Gillespie_8830b ribulose-1,5-bisphosphate carboxylase/oxygenase large subunit (rbcL) gene, partial cds; chloroplast	778	778	86%	0.0	97.20%	KC482730.1
Equisetum palustre voucher Chase 11641 (K) ribulose-1,5-bisphosphate carboxylase/oxygenase large subunit (rbcL) gene, partial cds; chloroplast	778	778	86%	0.0	97.20%	GQ248601.1
Equisetum sylvaticum voucher TJD-502 ribulose-1,5-bisphosphate carboxylase/oxygenase large subunit (rbcL) gene, partial cds; chloroplast	776	776	86%	0.0	97.00%	KJ841305.1
Equisetum palustre ribulose-1,5-bisphosphate carboxylase/oxygenase large subunit (rbcL) gene, partial cds; chloroplast	776	776	85%	0.0	97.20%	MZ359290.1
Equisetum sylvaticum voucher WAB_0132469351 ribulose-1,5-bisphosphate carboxylase/oxygenase large subunit (rbcL) gene, partial cds; chloroplast	773	773	86%	0.0	96.99%	KJ593397.1

Description	Max Score	Total Score	Query Cover	E value	Per. Ident	Accession
Equisetum sylvaticum voucher WAB_1079140180 ribulose-1,5-bisphosphate carboxylase/oxygenase large subunit (rbcL) gene, partial cds; chloroplast	773	773	86%	0.0	96.99%	KJ593395.1
Equisetum pratense voucher WAB_0132469185 ribulose-1,5-bisphosphate carboxylase/oxygenase large subunit (rbcL) gene, partial cds; chloroplast	773	773	86%	0.0	96.99%	KJ593391.1
Equisetum sylvaticum voucher Gillespie_8524 ribulose-1,5-bisphosphate carboxylase/oxygenase large subunit (rbcL) gene, partial cds; chloroplast	773	773	86%	0.0	96.99%	KC482739.1
Equisetum scirpoides isolate 41089 ribulose-1,5-bisphosphate carboxylase/oxygenase large subunit (rbcL) gene, partial cds; plastid	769	769	99%	0.0	93.12%	MH750203.1
Equisetum scirpoides isolate 26090 ribulose-1,5-bisphosphate carboxylase/oxygenase large subunit (rbcL) gene, partial cds; plastid	769	769	99%	0.0	93.12%	MH750202.1
Equisetum scirpoides isolate 10933 ribulose-1,5-bisphosphate carboxylase/oxygenase large subunit (rbcL) gene, partial cds; plastid	769	769	99%	0.0	93.12%	MH750201.1
Equisetum komarovii isolate 40830 ribulose-1,5-bisphosphate carboxylase/oxygenase large subunit (rbcL) gene, partial cds; plastid	769	769	99%	0.0	93.12%	MH750182.1
Equisetum scirpoides chloroplast rbcL gene for ribulose-1,5-bisphosphate carboxylase/oxygenase large subunit, partial cds, specimen_voucher: TNS:768168	769	769	99%	0.0	93.12%	AB574689.1
Equisetum scirpoides ribulose-1,5-bisphosphate carboxylase/oxygenase large subunit (rbcL) gene, partial cds; chloroplast gene for chloroplast product	769	769	99%	0.0	93.12%	AY226133.1
Equisetum telmateia subsp. telmateia isolate 41082 ribulose-1,5-bisphosphate carboxylase/oxygenase large subunit (rbcL) gene, partial cds; plastid	765	765	93%	0.0	94.66%	MH750211.1
Equisetum bogotense isolate 40827 ribulose-1,5-bisphosphate carboxylase/oxygenase large subunit (rbcL) gene, partial cds; plastid	758	758	99%	0.0	92.75%	MH750165.1
Equisetum bogotense isolate 40802 ribulose-1,5-bisphosphate carboxylase/oxygenase large subunit (rbcL) gene, partial cds; plastid	758	758	99%	0.0	92.75%	MH750164.1
Equisetum bogotense isolate 40801 ribulose-1,5-bisphosphate carboxylase/oxygenase large subunit (rbcL) gene, partial cds; plastid	758	758	99%	0.0	92.75%	MH750163.1
Equisetum bogotense isolate 40800 ribulose-1,5-bisphosphate carboxylase/oxygenase large subunit (rbcL) gene, partial cds; plastid	758	758	99%	0.0	92.75%	MH750162.1

Description	Max Score	Total Score	Query Cover	E value	Per. Ident	Accession
Equisetum bogotense ribulose-1,5-bisphosphate carboxylase/oxygenase large subunit (rbcL) gene, partial cds; chloroplast gene for chloroplast product	758	758	99%	0.0	92.75%	AY226139.1
Equisetum palustre ribulose-1,5-bisphosphate carboxylase/oxygenase large subunit (rbcL) gene, partial cds; chloroplast	756	756	83%	0.0	97.13%	MK525785.1
Equisetum scirpoides voucher AP248 ribulose-1,5-bisphosphate carboxylase/oxygenase large subunit (rbcL) gene, partial cds; chloroplast	756	756	98%	0.0	93.03%	HQ590084.1
Equisetum sylvaticum ribulose-1,5-bisphosphate carboxylase/oxygenase large subunit (rbcL) gene, partial cds; chloroplast	750	750	83%	0.0	96.91%	MK525787.1
Equisetum pratense ribulose-1,5-bisphosphate carboxylase/oxygenase large subunit (rbcL) gene, partial cds; chloroplast	750	750	83%	0.0	96.91%	MK525786.1
Equisetum environmental sample IRbL.E3 ribulose-1,5-bisphosphate carboxylase/oxygenase large subunit (rbcL) gene, partial cds; plastid	749	749	89%	0.0	95.44%	JQ007379.1
Equisetum x schaffneri isolate 40814 ribulose-1,5-bisphosphate carboxylase/oxygenase large subunit (rbcL) gene, partial cds; plastid	747	747	99%	0.0	92.38%	MH750225.1
Equisetum myriochaetum isolate 41080 ribulose-1,5-bisphosphate carboxylase/oxygenase large subunit (rbcL) gene, partial cds; plastid	747	747	99%	0.0	92.38%	MH750190.1
Equisetum myriochaetum isolate 40936 ribulose-1,5-bisphosphate carboxylase/oxygenase large subunit (rbcL) gene, partial cds; plastid	747	747	99%	0.0	92.38%	MH750189.1
Equisetum myriochaetum isolate 40826 ribulose-1,5-bisphosphate carboxylase/oxygenase large subunit (rbcL) gene, partial cds; plastid	747	747	99%	0.0	92.38%	MH750188.1
Equisetum myriochaetum isolate 40825 ribulose-1,5-bisphosphate carboxylase/oxygenase large subunit (rbcL) gene, partial cds; plastid	747	747	99%	0.0	92.38%	MH750187.1
Equisetum myriochaetum isolate 40816 ribulose-1,5-bisphosphate carboxylase/oxygenase large subunit (rbcL) gene, partial cds; plastid	747	747	99%	0.0	92.38%	MH750186.1
Equisetum myriochaetum isolate 40815 ribulose-1,5-bisphosphate carboxylase/oxygenase large subunit (rbcL) gene, partial cds; plastid	747	747	99%	0.0	92.38%	MH750185.1
Equisetum myriochaetum isolate 26088 ribulose-1,5-bisphosphate carboxylase/oxygenase large subunit (rbcL) gene, partial cds; plastid	747	747	99%	0.0	92.38%	MH750184.1
Equisetum laevigatum isolate 40812 ribulose-1,5-bisphosphate carboxylase/oxygenase large subunit (rbcL) gene, partial cds; plastid	747	747	99%	0.0	92.38%	MH750183.1

Description	Max Score	Total Score	Query Cover	E value	Per. Ident	Accession
Equisetum sp. Schuettpelz 1643 ribulose-1,5-bisphosphate carboxylase/oxygenase large subunit (rbcL) gene, partial cds; chloroplast	747	747	99%	0.0	92.38%	MW620286.1

Alignments

Equisetum x litorale isolate 41084 ribulose-1,5-bisphosphate carboxylase/oxygenase large subunit (rbcL) gene, partial cds; plastid
Sequence ID: **MH750222.1** Length: 1292 Number of Matches: 1
Range 1: 80 to 595

Score	Expect	Identities	Gaps	Strand	Frame
852 bits(461)	0.0()	516/538(96%)	22/538(4%)	Plus/Plus	

Features:

```
Query  1    TGACTCCTCAACCGGGAGTACCACCGGAAGAAGCAGGAGCAGCCGTAGCTGCTGAATCGT  60
            ||||||||||||||||||||||||||||||||||||||||||||||||||||||||||||
Sbjct  80   TGACTCCTCAACCGGGAGTACCACCGGAAGAAGCAGGAGCAGCCGTAGCTGCTGAATCGT  139

Query  61   CCACGGGCACCTGGACTACCGTATGGACAGATGGACTTACTAGTCTTGATCGATATAAAG  120
            ||||||||||||||||||||||||||||||||||||||||||||||||||||||||||||
Sbjct  140  CCACGGGCACCTGGACTACCGTATGGACAGATGGACTTACTAGTCTTGATCGATATAAAG  199

Query  121  GTCGATGCTATAATATTGAACCTGTTGCTGGAGAAGATAATCAATTCATAGCTTATGTAG  180
            ||||||||||||||||||||||||||||||||||||||||||||||||||||||||||||
Sbjct  200  GTCGATGCTATAATATTGAACCTGTTGCTGGAGAAGATAATCAATTCATAGCTTATGTAG  259

Query  181  CCTACCCTTTAGATCTTTTTGAAGAAGGTTCTGTTACCAATCTTTTTACTTCAATTGTTG  240
            ||||||||||||||||||||||||||||||||||||||||||||||||||||||||||||
Sbjct  260  CCTACCCTTTAGATCTTTTTGAAGAAGGTTCTGTTACCAATCTTTTTACTTCAATTGTTG  319

Query  241  GTAATGTTTTCGGCTTCAAAGCTCTACGTGCTTTACGTTTAGAAGATTTAAGAATTCCTC  300
            ||||||||||||||||||||||||||||||||||||||||||||||||||||||||||||
Sbjct  320  GTAATGTTTTCGGCTTCAAAGCTCTACGTGCTTTACGTTTAGAAGATTTAAGAATTCCTC  379

Query  301  CTGCTTACTCCAAAACTTTTATAGGGGAACCCCAcccccccAACGGTATCCAGGGTTGAA  360
            |||||||||||||||||||||||||  | ||  |||||   | ||||||||| |||||||
Sbjct  380  CTGCTTACTCCAAAACTTTTATAGG--A-CC--ACCCC---A-CGGTATCCA-GGTTGAA  429

Query  361  AGAGATAAGTTAAACAAATACGGTCGTCCTTTATTAGGTTGTACAATTAAACCAAAATTG  420
            ||||||||||||||||||||||||||||||||||||||||||||||||||||||||||||
Sbjct  430  AGAGATAAGTTAAACAAATACGGTCGTCCTTTATTAGGTTGTACAATTAAACCAAAATTG  489

Query  421  GGACTATCTGCTAAAAACTATGGTAGAGCTGTTTATGAATGTCTTCGTGGTGGGCTTGAA  480
            ||||||||||||||||||||||||||||||||||||||||||||||||||||||||||| 
Sbjct  490  GGACTATCTGCTAAAAACTATGGTAGAGCTGTTTATGAATGTCTTCGTGGTGGGCTTGA-  548

Query  481  TTTTTCAACCCAAAAAGGAATGAATGAAGAAATGTAAACTCTCAACCCTTTATGCGTT  538
            |||  || || |||  | | ||| ||| | ||||||||||||||||||||||||||||
Sbjct  549  TTT--CA-CC-AAA--G-A-TGA-TGA-G-AATGTAAACTCTCAACCCTTTATGCGTT  595
```

Equisetum x dycei isolate 26083 ribulose-1,5-bisphosphate carboxylase/oxygenase large subunit (rbcL) gene, partial cds; plastid
Sequence ID: **MH750220.1** Length: 1312 Number of Matches: 1
Range 1: 92 to 607

Score	Expect	Identities	Gaps	Strand	Frame
852 bits(461)	0.0()	516/538(96%)	22/538(4%)	Plus/Plus	

Features:

```
Query  1    TGACTCCTCAACCGGGAGTACCACCGGAAGAAGCAGGAGCAGCCGTAGCTGCTGAATCGT  60
            ||||||||||||||||||||||||||||||||||||||||||||||||||||||||||||
Sbjct  92   TGACTCCTCAACCGGGAGTACCACCGGAAGAAGCAGGAGCAGCCGTAGCTGCTGAATCGT  151

Query  61   CCACGGGCACCTGGACTACCGTATGGACAGATGGACTTACTAGTCTTGATCGATATAAAG  120
            ||||||||||||||||||||||||||||||||||||||||||||||||||||||||||||
Sbjct  152  CCACGGGCACCTGGACTACCGTATGGACAGATGGACTTACTAGTCTTGATCGATATAAAG  211

Query  121  GTCGATGCTATAATATTGAACCTGTTGCTGGAGAAGATAATCAATTCATAGCTTATGTAG  180
            ||||||||||||||||||||||||||||||||||||||||||||||||||||||||||||
Sbjct  212  GTCGATGCTATAATATTGAACCTGTTGCTGGAGAAGATAATCAATTCATAGCTTATGTAG  271
```

```
Query  181   CCTACCCTTTAGATCTTTTTGAAGAAGGTTCTGTTACCAATCTTTTTACTTCAATTGTTG  240
             ||||||||||||||||||||||||||||||||||||||||||||||||||||||||||||
Sbjct  272   CCTACCCTTTAGATCTTTTTGAAGAAGGTTCTGTTACCAATCTTTTTACTTCAATTGTTG  331

Query  241   GTAATGTTTTCGGCTTCAAAGCTCTACGTGCTTTACGTTTAGAAGATTTAAGAATTCCTC  300
             ||||||||||||||||||||||||||||||||||||||||||||||||||||||||||||
Sbjct  332   GTAATGTTTTCGGCTTCAAAGCTCTACGTGCTTTACGTTTAGAAGATTTAAGAATTCCTC  391

Query  301   CTGCTTACTCCAAAACTTTTATAGGGGAACCCCAcccccccAACGGTATCCAGGGTTGAA  360
             |||||||||||||||||||||||||  | ||  |||||   | ||||||||| |||||||
Sbjct  392   CTGCTTACTCCAAAACTTTTATAGG--A-CC--ACCCC---A-CGGTATCCA-GGTTGAA  441

Query  361   AGAGATAAGTTAAACAAATACGGTCGTCCTTTATTAGGTTGTACAATTAAACCAAAATTG  420
             ||||||||||||||||||||||||||||||||||||||||||||||||||||||||||||
Sbjct  442   AGAGATAAGTTAAACAAATACGGTCGTCCTTTATTAGGTTGTACAATTAAACCAAAATTG  501

Query  421   GGACTATCTGCTAAAAACTATGGTAGAGCTGTTTATGAATGTCTTCGTGGTGGGCTTGAA  480
             ||||||||||||||||||||||||||||||||||||||||||||||||||||||||||| 
Sbjct  502   GGACTATCTGCTAAAAACTATGGTAGAGCTGTTTATGAATGTCTTCGTGGTGGGCTTGA-  560

Query  481   TTTTTCAACCCAAAAAGGAATGAATGAAGAAATGTAAACTCTCAACCCTTTATGCGTT  538
             |||  || || |||  | | ||| ||| | ||||||||||||||||||||||||||||
Sbjct  561   TTT--CA-CC-AAA--G-A-TGA-TGA-G-AATGTAAACTCTCAACCCTTTATGCGTT  607
```

Equisetum fluviatile isolate 41076 ribulose-1,5-bisphosphate carboxylase/oxygenase large subunit (rbcL) gene, partial cds; plastid
Sequence ID: **MH750169.1** Length: 1308 Number of Matches: 1
Range 1: 91 to 606

Score	Expect	Identities	Gaps	Strand	Frame
852 bits(461)	0.0()	516/538(96%)	22/538(4%)	Plus/Plus	

Features:

```
Query  1     TGACTCCTCAACCGGGAGTACCACCGGAAGAAGCAGGAGCAGCCGTAGCTGCTGAATCGT  60
             ||||||||||||||||||||||||||||||||||||||||||||||||||||||||||||
Sbjct  91    TGACTCCTCAACCGGGAGTACCACCGGAAGAAGCAGGAGCAGCCGTAGCTGCTGAATCGT  150

Query  61    CCACGGGCACCTGGACTACCGTATGGACAGATGGACTTACTAGTCTTGATCGATATAAAG  120
             ||||||||||||||||||||||||||||||||||||||||||||||||||||||||||||
Sbjct  151   CCACGGGCACCTGGACTACCGTATGGACAGATGGACTTACTAGTCTTGATCGATATAAAG  210

Query  121   GTCGATGCTATAATATTGAACCTGTTGCTGGAGAAGATAATCAATTCATAGCTTATGTAG  180
             ||||||||||||||||||||||||||||||||||||||||||||||||||||||||||||
Sbjct  211   GTCGATGCTATAATATTGAACCTGTTGCTGGAGAAGATAATCAATTCATAGCTTATGTAG  270

Query  181   CCTACCCTTTAGATCTTTTTGAAGAAGGTTCTGTTACCAATCTTTTTACTTCAATTGTTG  240
             ||||||||||||||||||||||||||||||||||||||||||||||||||||||||||||
Sbjct  271   CCTACCCTTTAGATCTTTTTGAAGAAGGTTCTGTTACCAATCTTTTTACTTCAATTGTTG  330

Query  241   GTAATGTTTTCGGCTTCAAAGCTCTACGTGCTTTACGTTTAGAAGATTTAAGAATTCCTC  300
             ||||||||||||||||||||||||||||||||||||||||||||||||||||||||||||
Sbjct  331   GTAATGTTTTCGGCTTCAAAGCTCTACGTGCTTTACGTTTAGAAGATTTAAGAATTCCTC  390

Query  301   CTGCTTACTCCAAAACTTTTATAGGGGAACCCCAcccccccAACGGTATCCAGGGTTGAA  360
             |||||||||||||||||||||||||  | ||  |||||   | ||||||||| |||||||
Sbjct  391   CTGCTTACTCCAAAACTTTTATAGG--A-CC--ACCCC---A-CGGTATCCA-GGTTGAA  440

Query  361   AGAGATAAGTTAAACAAATACGGTCGTCCTTTATTAGGTTGTACAATTAAACCAAAATTG  420
             ||||||||||||||||||||||||||||||||||||||||||||||||||||||||||||
Sbjct  441   AGAGATAAGTTAAACAAATACGGTCGTCCTTTATTAGGTTGTACAATTAAACCAAAATTG  500

Query  421   GGACTATCTGCTAAAAACTATGGTAGAGCTGTTTATGAATGTCTTCGTGGTGGGCTTGAA  480
             ||||||||||||||||||||||||||||||||||||||||||||||||||||||||||| 
Sbjct  501   GGACTATCTGCTAAAAACTATGGTAGAGCTGTTTATGAATGTCTTCGTGGTGGGCTTGA-  559

Query  481   TTTTTCAACCCAAAAAGGAATGAATGAAGAAATGTAAACTCTCAACCCTTTATGCGTT  538
             |||  || || |||  | | ||| ||| | ||||||||||||||||||||||||||||
Sbjct  560   TTT--CA-CC-AAA--G-A-TGA-TGA-G-AATGTAAACTCTCAACCCTTTATGCGTT  606
```

Equisetum fluviatile isolate 41075 ribulose-1,5-bisphosphate carboxylase/oxygenase large subunit (rbcL) gene, partial cds; plastid
Sequence ID: **MH750168.1** Length: 1311 Number of Matches: 1
Range 1: 91 to 606

Score	Expect	Identities	Gaps	Strand	Frame
852 bits(461)	0.0()	516/538(96%)	22/538(4%)	Plus/Plus	

Features:

```
Query  1     TGACTCCTCAACCGGGAGTACCACCGGAAGAAGCAGGAGCAGCCGTAGCTGCTGAATCGT  60
             ||||||||||||||||||||||||||||||||||||||||||||||||||||||||||||
```

```
Sbjct  91    TGACTCCTCAACCGGGAGTACCACCGGAAGAAGCAGGAGCAGCCGTAGCTGCTGAATCGT  150

Query  61    CCACGGGCACCTGGACTACCGTATGGACAGATGGACTTACTAGTCTTGATCGATATAAAG  120
             ||||||||||||||||||||||||||||||||||||||||||||||||||||||||||||
Sbjct  151   CCACGGGCACCTGGACTACCGTATGGACAGATGGACTTACTAGTCTTGATCGATATAAAG  210

Query  121   GTCGATGCTATAATATTGAACCTGTTGCTGGAGAAGATAATCAATTCATAGCTTATGTAG  180
             ||||||||||||||||||||||||||||||||||||||||||||||||||||||||||||
Sbjct  211   GTCGATGCTATAATATTGAACCTGTTGCTGGAGAAGATAATCAATTCATAGCTTATGTAG  270

Query  181   CCTACCCTTTAGATCTTTTTGAAGAAGGTTCTGTTACCAATCTTTTTACTTCAATTGTTG  240
             ||||||||||||||||||||||||||||||||||||||||||||||||||||||||||||
Sbjct  271   CCTACCCTTTAGATCTTTTTGAAGAAGGTTCTGTTACCAATCTTTTTACTTCAATTGTTG  330

Query  241   GTAATGTTTTCGGCTTCAAAGCTCTACGTGCTTTACGTTTAGAAGATTTAAGAATTCCTC  300
             ||||||||||||||||||||||||||||||||||||||||||||||||||||||||||||
Sbjct  331   GTAATGTTTTCGGCTTCAAAGCTCTACGTGCTTTACGTTTAGAAGATTTAAGAATTCCTC  390

Query  301   CTGCTTACTCCAAAACTTTTATAGGGGAACCCCAcccccccAACGGTATCCAGGGTTGAA  360
             |||||||||||||||||||||||||  | ||  |||||   | ||||||||| |||||||
Sbjct  391   CTGCTTACTCCAAAACTTTTATAGG--A-CC--ACCCC---A-CGGTATCCA-GGTTGAA  440

Query  361   AGAGATAAGTTAAACAAATACGGTCGTCCTTTATTAGGTTGTACAATTAAACCAAAATTG  420
             ||||||||||||||||||||||||||||||||||||||||||||||||||||||||||||
Sbjct  441   AGAGATAAGTTAAACAAATACGGTCGTCCTTTATTAGGTTGTACAATTAAACCAAAATTG  500

Query  421   GGACTATCTGCTAAAAACTATGGTAGAGCTGTTTATGAATGTCTTCGTGGTGGGCTTGAA  480
             ||||||||||||||||||||||||||||||||||||||||||||||||||||||||||| 
Sbjct  501   GGACTATCTGCTAAAAACTATGGTAGAGCTGTTTATGAATGTCTTCGTGGTGGGCTTGA-  559

Query  481   TTTTTCAACCCAAAAAGGAATGAATGAAGAAATGTAAACTCTCAACCCTTTATGCGTT  538
             |||  || || |||  | | ||| ||| | ||||||||||||||||||||||||||||
Sbjct  560   TTT--CA-CC-AAA--G-A-TGA-TGA-G-AATGTAAACTCTCAACCCTTTATGCGTT  606
```

Equisetum arvense x Equisetum telmateia subsp. braunii isolate 40834 ribulose-1,5-bisphosphate carboxylase/oxygenase large subunit (rbcL) gene, partial cds; plastid

Sequence ID: **MH750161.1** Length: 1290 Number of Matches: 1

Range 1: 63 to 578

Score	Expect	Identities	Gaps	Strand	Frame
852 bits(461)	0.0()	516/538(96%)	22/538(4%)	Plus/Plus	

Features:

```
Query  1     TGACTCCTCAACCGGGAGTACCACCGGAAGAAGCAGGAGCAGCCGTAGCTGCTGAATCGT  60
             ||||||||||||||||||||||||||||||||||||||||||||||||||||||||||||
Sbjct  63    TGACTCCTCAACCGGGAGTACCACCGGAAGAAGCAGGAGCAGCCGTAGCTGCTGAATCGT  122

Query  61    CCACGGGCACCTGGACTACCGTATGGACAGATGGACTTACTAGTCTTGATCGATATAAAG  120
             ||||||||||||||||||||||||||||||||||||||||||||||||||||||||||||
Sbjct  123   CCACGGGCACCTGGACTACCGTATGGACAGATGGACTTACTAGTCTTGATCGATATAAAG  182

Query  121   GTCGATGCTATAATATTGAACCTGTTGCTGGAGAAGATAATCAATTCATAGCTTATGTAG  180
             ||||||||||||||||||||||||||||||||||||||||||||||||||||||||||||
Sbjct  183   GTCGATGCTATAATATTGAACCTGTTGCTGGAGAAGATAATCAATTCATAGCTTATGTAG  242

Query  181   CCTACCCTTTAGATCTTTTTGAAGAAGGTTCTGTTACCAATCTTTTTACTTCAATTGTTG  240
             ||||||||||||||||||||||||||||||||||||||||||||||||||||||||||||
Sbjct  243   CCTACCCTTTAGATCTTTTTGAAGAAGGTTCTGTTACCAATCTTTTTACTTCAATTGTTG  302

Query  241   GTAATGTTTTCGGCTTCAAAGCTCTACGTGCTTTACGTTTAGAAGATTTAAGAATTCCTC  300
             ||||||||||||||||||||||||||||||||||||||||||||||||||||||||||||
Sbjct  303   GTAATGTTTTCGGCTTCAAAGCTCTACGTGCTTTACGTTTAGAAGATTTAAGAATTCCTC  362

Query  301   CTGCTTACTCCAAAACTTTTATAGGGGAACCCCAcccccccAACGGTATCCAGGGTTGAA  360
             |||||||||||||||||||||||||  | ||  |||||   | ||||||||| |||||||
Sbjct  363   CTGCTTACTCCAAAACTTTTATAGG--A-CC--ACCCC---A-CGGTATCCA-GGTTGAA  412

Query  361   AGAGATAAGTTAAACAAATACGGTCGTCCTTTATTAGGTTGTACAATTAAACCAAAATTG  420
             ||||||||||||||||||||||||||||||||||||||||||||||||||||||||||||
Sbjct  413   AGAGATAAGTTAAACAAATACGGTCGTCCTTTATTAGGTTGTACAATTAAACCAAAATTG  472

Query  421   GGACTATCTGCTAAAAACTATGGTAGAGCTGTTTATGAATGTCTTCGTGGTGGGCTTGAA  480
             ||||||||||||||||||||||||||||||||||||||||||||||||||||||||||| 
Sbjct  473   GGACTATCTGCTAAAAACTATGGTAGAGCTGTTTATGAATGTCTTCGTGGTGGGCTTGA-  531

Query  481   TTTTTCAACCCAAAAAGGAATGAATGAAGAAATGTAAACTCTCAACCCTTTATGCGTT  538
             |||  || || |||  | | ||| ||| | ||||||||||||||||||||||||||||
Sbjct  532   TTT--CA-CC-AAA--G-A-TGA-TGA-G-AATGTAAACTCTCAACCCTTTATGCGTT  578
```

BLAST is a registered trademark of the National Library of Medicine

Support center Mailing list YouTube

- National Library Of Medicine
- National Institutes Of Health
- U.S. Department of Health & Human Services
- USA.gov

NCBI
National Center for Biotechnology Information, U.S. National Library of Medicine 8600 Rockville Pike, Bethesda MD, 20894 USA
Policies and Guidelines | Contact
HHS Vulnerability Disclosure
