# Supplementary material for: A Multi-Pronged Technique for Identifying Equisetum palustre and Equisetum arvense—Combining HPTLC, HPLC-ESI-MS/MS and Optimized DNA Barcoding Techniques
Source: Plants (Basel). 2022 Sep 28;11(19):2562. doi: 10.3390/plants11192562 (PMC9573248; doi:10.3390/plants11192562)
Supplement: Supplementary file 1 [file plants-11-02562-s001.zip › plants-1932149 supplementary/Horsetail Supplementary Figures/Figure S4.pdf]

BLAST Results

Questions/comments

Job title: MW040322.1

**RID**	FD5BHM6M013 (Expires on 08-14 02:01 am)
**Query ID**	lcl|Query_54821
**Description**	None
**Molecule type**	dna
**Query Length**	335
**Database Name**	nt
**Description**	Nucleotide collection (nt)
**Program**	BLASTN 2.13.0+

Graphic Summary

Distribution of the top 108 Blast Hits on 100 subject sequences

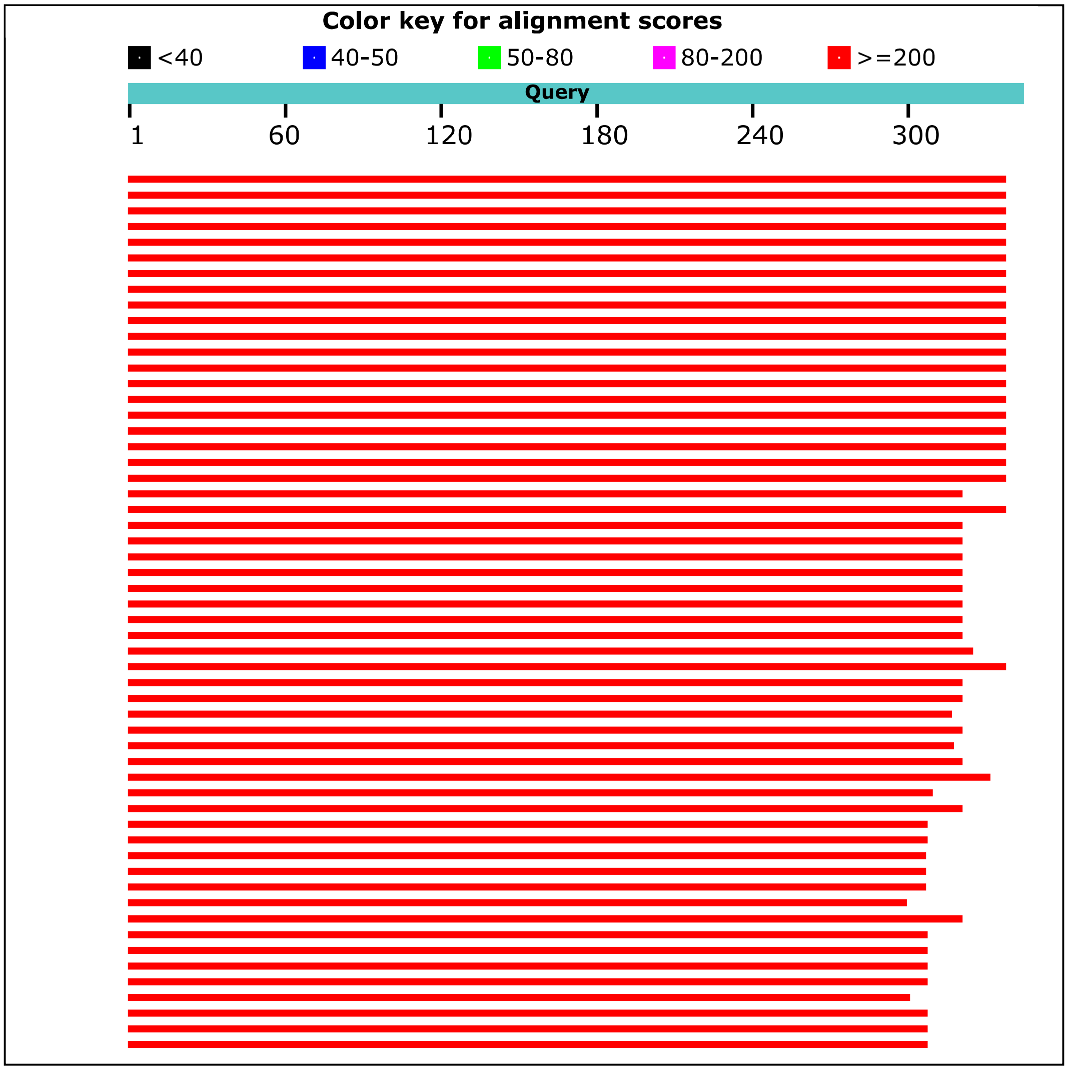

Descriptions

Sequences producing significant alignments:

Description	Max Score	Total Score	Query Cover	E value	Per. Ident	Accession
uncultured fungus genomic DNA sequence contains ITS1	608	608	100%	1e-169	99.10%	OW836430.1
Equisetum arvense voucher MN06 5.8S ribosomal RNA gene, partial sequence; internal transcribed spacer 2, complete sequence; and large subunit ribosomal RNA gene, partial sequence	608	608	100%	1e-169	99.10%	OL304250.1
Chloroidium sp. FACHB-2422 small subunit ribosomal RNA gene, partial sequence; internal transcribed spacer 1, 5.8S ribosomal RNA gene, and internal transcribed spacer 2, complete sequence; and large subunit ribosomal RNA gene, partial sequence	608	608	100%	1e-169	99.10%	MT006211.1
Equisetum arvense voucher 34008 5.8S ribosomal RNA gene, partial sequence; internal transcribed spacer 2, complete sequence; and large subunit ribosomal RNA gene, partial sequence	608	608	100%	1e-169	100.00%	MW040322.1
Chloroidium saccharophilum genomic DNA containing 18S rRNA gene, ITS1, 5.8S rRNA gene, ITS2, 28S rRNA gene, culture collection CCAP 211/31	603	603	100%	7e-168	98.81%	FR865664.1
Equisetum arvense voucher Equisetum_arvense_ITS.fasta_2 5.8S ribosomal RNA gene, partial sequence; internal transcribed spacer 2, complete sequence; and large subunit ribosomal RNA gene, partial sequence	601	601	100%	3e-167	98.51%	MW040323.1
Chloroidium saccharophilum strain FACHB-1796 18S ribosomal RNA gene, partial sequence; internal transcribed spacer 1, 5.8S ribosomal RNA gene, and internal transcribed spacer 2, complete sequence; and 28S ribosomal RNA gene, partial sequence	597	597	100%	3e-166	98.51%	KX024689.1
Equisetum arvense voucher MN05 5.8S ribosomal RNA gene, partial sequence; internal transcribed spacer 2, complete sequence; and large subunit ribosomal RNA gene, partial sequence	597	597	100%	3e-166	98.51%	OL304249.1
uncultured Pezizaceae genomic DNA sequence contains ITS1	597	597	100%	3e-166	98.51%	OU496020.1
Equisetum arvense voucher MN01 5.8S ribosomal RNA gene, partial sequence; internal transcribed spacer 2, complete sequence; and large subunit ribosomal RNA gene, partial sequence	593	593	100%	4e-165	98.51%	OL304248.1
Equisetum arvense voucher Equisetum_arvense_ITS.fasta_1 5.8S ribosomal RNA gene, partial sequence; internal transcribed spacer 2, complete sequence; and large subunit ribosomal RNA gene, partial sequence	593	593	100%	4e-165	97.91%	MW040324.1

Description	Max Score	Total Score	Query Cover	E value	Per. Ident	Accession
Unclassified Viridiplantae clone 1537 5.8S ribosomal RNA gene, partial sequence; internal transcribed spacer 2, complete sequence; and large subunit ribosomal RNA gene, partial sequence	592	592	100%	2e-164	98.21%	MF570051.1
Chloroidium ellipsoideum isolate FG2/4.5E 18S ribosomal RNA gene, partial sequence; internal transcribed spacer 1, 5.8S ribosomal RNA gene, and internal transcribed spacer 2, complete sequence; and 26S ribosomal RNA gene, partial sequence	592	592	100%	2e-164	98.21%	KM020071.1
Chloroidium saccharophilum isolate FG2/1.3 18S ribosomal RNA gene, partial sequence; internal transcribed spacer 1, 5.8S ribosomal RNA gene, and internal transcribed spacer 2, complete sequence; and 26S ribosomal RNA gene, partial sequence	592	592	100%	2e-164	98.21%	KM020070.1
Chloroidium saccharophilum var. saccharophilum strain UTEX247 18S ribosomal RNA gene, partial sequence; internal transcribed spacer 1, 5.8S ribosomal RNA gene, and internal transcribed spacer 2, complete sequence; and 28S ribosomal RNA gene, partial sequence	592	592	100%	2e-164	98.21%	KJ676130.1
Chloroidium saccharophilum strain OD-1-1-C small subunit ribosomal RNA gene, partial sequence; internal transcribed spacer 1, 5.8S ribosomal RNA gene, and internal transcribed spacer 2, complete sequence; and large subunit ribosomal RNA gene, partial sequence	592	592	100%	2e-164	98.21%	MT991539.1
Chloroidium ellipsoideum genomic DNA containing 18S rRNA gene, ITS1, 5.8S rRNA gene, ITS2, 28S rRNA gene, culture collection CCAP 211/40	592	592	100%	2e-164	98.21%	FR865669.1
Chloroidium saccharophilum genomic DNA containing 18S rRNA gene, ITS1, 5.8S rRNA gene, ITS2, 28S rRNA gene, culture collection CCAP 211/32	592	592	100%	2e-164	98.21%	FR865665.1
Chloroidium saccharophilum var. saccharophilum strain UTEX2469 18S ribosomal RNA gene, partial sequence; internal transcribed spacer 1, 5.8S ribosomal RNA gene, and internal transcribed spacer 2, complete sequence; and 28S ribosomal RNA gene, partial sequence	580	580	100%	3e-161	97.61%	KJ676131.1
Chloroidium sp. FACHB-2420 small subunit ribosomal RNA gene, partial sequence; internal transcribed spacer 1, 5.8S ribosomal RNA gene, and internal transcribed spacer 2, complete sequence; and large subunit ribosomal RNA gene, partial sequence	580	580	100%	3e-161	97.61%	MT006209.1
Chloroidium saccharophilum strain ISBAL-007 18S ribosomal RNA gene, internal transcribed spacer 1, 5.8S ribosomal RNA gene, internal transcribed spacer 2, and 28S ribosomal RNA gene, region	577	577	94%	4e-160	99.06%	MH551492.1

Description	Max Score	Total Score	Query Cover	E value	Per. Ident	Accession
'Chlorella' ellipsoidea strain Ce 18S ribosomal RNA gene, partial sequence; internal transcribed spacer 1, 5.8S ribosomal RNA gene, and internal transcribed spacer 2, complete sequence; and 26S ribosomal RNA gene, partial sequence	575	575	100%	2e-159	97.31%	EU038292.1
Chlorella saccharophila partial 18S rRNA gene, ITS1, 5.8S rRNA, ITS2 and partial 28S rRNA gene, strain SAG 2120	566	566	94%	9e-157	98.43%	FM946008.1
Chlorella saccharophila partial 18S rRNA gene, ITS1, 5.8S rRNA, ITS2 and partial 28S rRNA gene, strain CCAP 211/42	566	566	94%	9e-157	98.43%	FM946004.1
Chlorella saccharophila partial 18S rRNA gene, ITS1, 5.8S rRNA, ITS2 and partial 28S rRNA gene, strain CCAP 211/36	566	566	94%	9e-157	98.43%	FM946003.1
Chloroidium saccharophilum strain CCAP 211/27 18S ribosomal RNA gene, internal transcribed spacer 1, 5.8S ribosomal RNA gene, internal transcribed spacer 2, and 28S ribosomal RNA gene, region	560	560	94%	4e-155	98.11%	MH551491.1
Chloroidium saccharophilum strain CCAP 211/40 small subunit ribosomal RNA gene, partial sequence; internal transcribed spacer 1 and 5.8S ribosomal RNA gene, complete sequence; and internal transcribed spacer 2, partial sequence	560	560	94%	4e-155	98.11%	MH551490.1
Chlorella saccharophila partial 18S rRNA gene, ITS1, 5.8S rRNA, ITS2 and partial 28S rRNA gene, strain SAG 2197	560	560	94%	4e-155	98.11%	FM946010.1
Chlorella saccharophila partial 18S rRNA gene, ITS1, 5.8S rRNA, ITS2 and partial 28S rRNA gene, strain SAG 211-1c	560	560	94%	4e-155	98.11%	FM946007.1
Chlorella saccharophila partial 18S rRNA gene, ITS1, 5.8S rRNA, ITS2 and partial 28S rRNA gene, strain CCAP 211/32	560	560	94%	4e-155	98.11%	FM946001.1
Chlorella sp. UTEX 2068 18S ribosomal RNA gene, partial sequence; internal transcribed spacer 1, 5.8S ribosomal RNA gene, and internal transcribed spacer 2, complete sequence; and 28S ribosomal RNA gene, partial sequence	556	556	96%	5e-154	97.52%	KJ676132.1
Chloroidium saccharophilum genomic DNA containing 18S rRNA gene, ITS1, 5.8S rRNA gene, ITS2, 28S rRNA gene, culture collection CCAP 211/58	556	556	100%	5e-154	96.42%	FR865677.1
Chloroidium saccharophilum strain FaII/B-1b small subunit ribosomal RNA gene, partial sequence; internal transcribed spacer 1 and 5.8S ribosomal RNA gene, complete sequence; and internal transcribed spacer 2, partial sequence	555	555	94%	2e-153	97.80%	MH551489.1
Chloroidium saccharophilum strain SAG 2055 small subunit ribosomal RNA gene, partial sequence; internal transcribed spacer 1 and 5.8S ribosomal RNA gene, complete sequence; and internal transcribed spacer 2, partial sequence	555	555	94%	2e-153	97.80%	MH551488.1

Description	Max Score	Total Score	Query Cover	E value	Per. Ident	Accession
Chloroidium saccharophilum strain KMMCC 192 18S ribosomal RNA gene, internal transcribed spacer 1, 5.8S ribosomal RNA gene, internal transcribed spacer 2, and 28S ribosomal RNA gene, region	553	553	93%	7e-153	98.09%	JQ315766.1
Chlorella saccharophila partial 18S rRNA gene, ITS1, 5.8S rRNA, ITS2 and partial 28S rRNA gene, strain SAG 211-1b	553	553	94%	7e-153	97.80%	FM946005.1
Uncultured alga genes for 5.8S rRNA, ITS2, 28S rRNA, partial and complete sequence, note: OTU163	549	549	94%	9e-152	97.78%	LC381753.1
Chlorella saccharophila partial 18S rRNA gene, ITS1, 5.8S rRNA, ITS2 and partial 28S rRNA gene, strain SAG 211-9a	549	549	94%	9e-152	97.48%	FM946000.1
Chloroidium saccharophilum strain KMMCC 158 18S ribosomal RNA gene, internal transcribed spacer 1, 5.8S ribosomal RNA gene, internal transcribed spacer 2, and 28S ribosomal RNA gene, region	538	538	98%	2e-148	95.47%	JQ315771.1
Chloroidium saccharophilum strain KMMCC 195 18S ribosomal RNA gene, internal transcribed spacer 1, 5.8S ribosomal RNA gene, internal transcribed spacer 2, and 28S ribosomal RNA gene, region	534	534	91%	3e-147	97.72%	JQ315769.1
Chloroidium saccharophilum strain CCAP 264/2 small subunit ribosomal RNA gene, partial sequence; internal transcribed spacer 1 and 5.8S ribosomal RNA gene, complete sequence; and internal transcribed spacer 2, partial sequence	531	531	94%	3e-146	96.54%	MH551493.1
Uncultured Chloroidium isolate A207 internal transcribed spacer 1, partial sequence; 5.8S ribosomal RNA gene, complete sequence; and internal transcribed spacer 2, partial sequence	531	531	91%	3e-146	97.70%	KP314644.1
Uncultured Chloroidium isolate A103 internal transcribed spacer 1, partial sequence; 5.8S ribosomal RNA gene, complete sequence; and internal transcribed spacer 2, partial sequence	531	531	91%	3e-146	97.70%	KP314563.1
Chloroidium saccharophilum strain St6 small subunit ribosomal RNA gene, partial sequence; internal transcribed spacer 1 and 5.8S ribosomal RNA gene, complete sequence; and internal transcribed spacer 2, partial sequence	529	529	90%	1e-145	97.70%	OM472016.1
Chloroidium saccharophilum strain ACSSI 340 small subunit ribosomal RNA gene, partial sequence; internal transcribed spacer 1 and 5.8S ribosomal RNA gene, complete sequence; and internal transcribed spacer 2, partial sequence	529	529	90%	1e-145	97.70%	MT827198.1
Chloroidium saccharophilum strain ACSSI 339 small subunit ribosomal RNA gene, partial sequence; internal transcribed spacer 1 and 5.8S ribosomal RNA gene, complete sequence; and internal transcribed spacer 2, partial sequence	529	529	90%	1e-145	97.70%	MT827197.1

Description	Max Score	Total Score	Query Cover	E value	Per. Ident	Accession
Chloroidium saccharophilum strain CCM-UDEC 143 18S ribosomal RNA gene, partial sequence; and internal transcribed spacer 1, 5S ribosomal RNA gene, and internal transcribed spacer 2, complete sequence	527	527	88%	4e-145	98.32%	KC517116.1
Chloroidium saccharophilum strain CCAP 211/58 small subunit ribosomal RNA gene, partial sequence; internal transcribed spacer 1 and 5.8S ribosomal RNA gene, complete sequence; and internal transcribed spacer 2, partial sequence	525	525	94%	2e-144	96.23%	MH551494.1
Uncultured Chloroidium isolate A214 internal transcribed spacer 1, partial sequence; 5.8S ribosomal RNA gene, complete sequence; and internal transcribed spacer 2, partial sequence	525	525	91%	2e-144	97.38%	KP314651.1
Uncultured Chloroidium isolate A032 internal transcribed spacer 1, partial sequence; 5.8S ribosomal RNA gene, complete sequence; and internal transcribed spacer 2, partial sequence	525	525	91%	2e-144	97.38%	KP314515.1
Uncultured Chloroidium isolate A029 internal transcribed spacer 1, partial sequence; 5.8S ribosomal RNA gene, complete sequence; and internal transcribed spacer 2, partial sequence	525	525	91%	2e-144	97.38%	KP314513.1
Uncultured Chloroidium isolate A011 internal transcribed spacer 1, partial sequence; 5.8S ribosomal RNA gene, complete sequence; and internal transcribed spacer 2, partial sequence	525	525	91%	2e-144	97.38%	KP314497.1
Chloroidium saccharophilum genomic DNA sequence contains 5.8S rRNA gene, ITS2, strain Chlorella saccharophila RNY	523	523	88%	6e-144	97.99%	LR215745.1
Uncultured Chloroidium isolate A023 internal transcribed spacer 1, partial sequence; 5.8S ribosomal RNA gene, complete sequence; and internal transcribed spacer 2, partial sequence	518	518	91%	3e-142	97.05%	KP314508.1
Uncultured Chloroidium isolate A141 internal transcribed spacer 1, partial sequence; 5.8S ribosomal RNA gene, complete sequence; and internal transcribed spacer 2, partial sequence	512	512	91%	1e-140	96.72%	KP314596.1
Uncultured Chloroidium isolate A217 internal transcribed spacer 1, partial sequence; 5.8S ribosomal RNA gene, complete sequence; and internal transcribed spacer 2, partial sequence	507	507	91%	6e-139	96.39%	KP314654.1
'Chlorella' saccharophila voucher UTEX 247 internal transcribed spacer 1, partial sequence; 5.8S ribosomal RNA gene, complete sequence; and internal transcribed spacer 2, partial sequence	499	499	91%	9e-137	95.48%	EF120783.1

Description	Max Score	Total Score	Query Cover	E value	Per. Ident	Accession
Chloroidium saccharophilum voucher Herbarium Tuerk51488 internal transcribed spacer 1, partial sequence; 5.8S ribosomal RNA gene, complete sequence; and internal transcribed spacer 2, partial sequence	497	497	84%	3e-136	97.89%	KF907674.1
Chloroidium saccharophilum voucher Herbarium Tuerk51481 internal transcribed spacer 1, partial sequence; 5.8S ribosomal RNA gene, complete sequence; and internal transcribed spacer 2, partial sequence	497	497	84%	3e-136	97.89%	KF907673.1
Chloroidium saccharophilum voucher Herbarium Tuerk51492 internal transcribed spacer 1, partial sequence; 5.8S ribosomal RNA gene, complete sequence; and internal transcribed spacer 2, partial sequence	492	492	84%	2e-134	97.54%	KF907675.1
Chloroidium saccharophilum voucher Herbarium Tuerk51480 internal transcribed spacer 1, partial sequence; 5.8S ribosomal RNA gene, complete sequence; and internal transcribed spacer 2, partial sequence	492	492	84%	2e-134	97.54%	KF907672.1
'Chlorella' saccharophila voucher UTEX 2911 internal transcribed spacer 1, partial sequence; 5.8S ribosomal RNA gene, complete sequence; and internal transcribed spacer 2, partial sequence	472	472	81%	2e-128	97.45%	EF120782.1
Chloroidium sp. S4MWC-51 internal transcribed spacer 1, 5.8S ribosomal RNA gene, and internal transcribed spacer 2, complete sequence	462	462	79%	1e-125	97.74%	MK005103.1
Uncultured Chloroidium isolate A026 internal transcribed spacer 1, partial sequence; 5.8S ribosomal RNA gene, complete sequence; and internal transcribed spacer 2, partial sequence	425	425	91%	2e-114	91.50%	KP314510.1
'Chlorella' saccharophila voucher UTEX 2469 internal transcribed spacer 1, partial sequence; 5.8S ribosomal RNA gene, complete sequence; and internal transcribed spacer 2, partial sequence	411	411	70%	5e-110	97.47%	EF120784.1
Chloroidium saccharophilum strain KMMCC 192 18S ribosomal RNA gene, internal transcribed spacer 1, 5.8S ribosomal RNA gene, internal transcribed spacer 2, and 28S ribosomal RNA gene, region	355	355	58%	2e-93	98.98%	JQ315767.1
Uncultured alga isolate ASV_573_A 5.8S ribosomal RNA gene, partial sequence; internal transcribed spacer 2, complete sequence; and large subunit ribosomal RNA gene, partial sequence	270	270	100%	8e-68	81.28%	ON119385.1
Chloroidium angustoellipsoideum voucher VancurovaA356 internal transcribed spacer 1, partial sequence; 5.8S ribosomal RNA gene and internal transcribed spacer 2, complete sequence; and large subunit ribosomal RNA gene, partial sequence	246	246	100%	1e-60	81.21%	MH415249.1

Description	Max Score	Total Score	Query Cover	E value	Per. Ident	Accession
Chloroidium viscosum strain MG-2 small subunit ribosomal RNA gene, partial sequence; internal transcribed spacer 1 and 5.8S ribosomal RNA gene, complete sequence; and internal transcribed spacer 2, partial sequence	241	241	94%	6e-59	80.35%	MH551496.1
Chloroidium lichenum strain BII/C-1 small subunit ribosomal RNA gene, partial sequence; internal transcribed spacer 1 and 5.8S ribosomal RNA gene, complete sequence; and internal transcribed spacer 2, partial sequence	237	237	77%	8e-58	83.64%	MH551507.1
Chloroidium lichenum strain MG-4 small subunit ribosomal RNA gene, partial sequence; internal transcribed spacer 1 and 5.8S ribosomal RNA gene, complete sequence; and internal transcribed spacer 2, partial sequence	237	237	77%	8e-58	83.64%	MH551506.1
Chloroidium lichenum strain BII/B-9 small subunit ribosomal RNA gene, partial sequence; internal transcribed spacer 1 and 5.8S ribosomal RNA gene, complete sequence; and internal transcribed spacer 2, partial sequence	237	237	77%	8e-58	83.64%	MH551505.1
Uncultured Chlorella clone CM-E-A25 internal transcribed spacer 1, partial sequence; 5.8S ribosomal RNA gene, complete sequence; and internal transcribed spacer 2, partial sequence	237	237	77%	8e-58	83.64%	KJ489091.1
Chlorella angustoellipsoidea partial 18S rRNA gene, ITS1, 5.8S rRNA, ITS2 and partial 28S rRNA gene, strain SAG 2115	237	237	77%	8e-58	83.64%	FM946019.1
Chlorella angustoellipsoidea partial 18S rRNA gene, ITS1, 5.8S rRNA, ITS2 and partial 28S rRNA gene, strain SAG 2144	237	237	77%	8e-58	83.64%	FM946020.1
Uncultured alga genes for 5.8S rRNA, ITS2, 28S rRNA, partial and complete sequence, note: OTU71	235	235	53%	3e-57	90.16%	LC381752.1
uncultured fungus genomic DNA sequence contains ITS1	235	235	100%	3e-57	79.50%	OW836154.1
Chloroidium lichenum strain SAG 2293 18S ribosomal RNA gene, internal transcribed spacer 1, 5.8S ribosomal RNA gene, internal transcribed spacer 2, and 28S ribosomal RNA gene, region	231	231	67%	4e-56	85.17%	MH551510.1
Chloroidium lichenum strain SAG 2294 18S ribosomal RNA gene, internal transcribed spacer 1, 5.8S ribosomal RNA gene, internal transcribed spacer 2, and 28S ribosomal RNA gene, region	231	231	67%	4e-56	85.17%	MH551509.1
Chloroidium sp. LV-2018 voucher VancurovaA318 small subunit ribosomal RNA gene, partial sequence; internal transcribed spacer 1 and 5.8S ribosomal RNA gene, complete sequence; and internal transcribed spacer 2, partial sequence	231	231	67%	4e-56	85.17%	MH415228.1

Description	Max Score	Total Score	Query Cover	E value	Per. Ident	Accession
Chloroidium lichenum strain SAG 2141 18S ribosomal RNA gene, internal transcribed spacer 1, 5.8S ribosomal RNA gene, internal transcribed spacer 2, and 28S ribosomal RNA gene, region	230	230	53%	1e-55	90.00%	MH551512.1
Chloroidium sp. LV-2018 voucher VancurovaA503 small subunit ribosomal RNA gene, partial sequence; internal transcribed spacer 1, 5.8S ribosomal RNA gene, and internal transcribed spacer 2, complete sequence; and large subunit ribosomal RNA gene, partial sequence	230	303	65%	1e-55	90.00%	MH415295.1
Chloroidium angustoellipsoideum voucher VancurovaA421.2 internal transcribed spacer 1, partial sequence; 5.8S ribosomal RNA gene and internal transcribed spacer 2, complete sequence; and large subunit ribosomal RNA gene, partial sequence	230	312	90%	1e-55	83.27%	MH415277.1
Chloroidium orientale strain VCA-45 (Ce2E) small subunit ribosomal RNA gene, partial sequence; internal transcribed spacer 1, 5.8S ribosomal RNA gene, and internal transcribed spacer 2, complete sequence; and large subunit ribosomal RNA gene, partial sequence	228	228	53%	5e-55	89.94%	MZ558750.1
Chloroidium sp. LV-2018 voucher VancurovaO29 small subunit ribosomal RNA gene, partial sequence; internal transcribed spacer 1, 5.8S ribosomal RNA gene, and internal transcribed spacer 2, complete sequence; and large subunit ribosomal RNA gene, partial sequence	226	303	90%	2e-54	83.27%	MH415404.1
Chloroidium ellipsoideum voucher RidkaNZ23 internal transcribed spacer 1, partial sequence; 5.8S ribosomal RNA gene, complete sequence; and internal transcribed spacer 2, partial sequence	226	226	53%	2e-54	89.94%	MH415207.1
Chloroidium lichenum voucher A114 internal transcribed spacer 1, partial sequence; 5.8S ribosomal RNA gene, complete sequence; and internal transcribed spacer 2, partial sequence	224	224	67%	6e-54	84.68%	OL625124.1
Chloroidium lichenum strain CCAP 211/33 18S ribosomal RNA gene, internal transcribed spacer 1, 5.8S ribosomal RNA gene, internal transcribed spacer 2, and 28S ribosomal RNA gene, region	222	222	53%	2e-53	89.39%	MH551518.1
Chloroidium lichenum strain QII/C-5b small subunit ribosomal RNA gene, partial sequence; internal transcribed spacer 1 and 5.8S ribosomal RNA gene, complete sequence; and internal transcribed spacer 2, partial sequence	222	222	67%	2e-53	84.26%	MH551508.1
Chloroidium sp. Ru-6-6 small subunit ribosomal RNA gene, partial sequence; internal transcribed spacer 1, 5.8S ribosomal RNA gene, and internal transcribed spacer 2, complete sequence; and large subunit ribosomal RNA gene, partial sequence	222	299	67%	2e-53	89.39%	MH703747.1

Description	Max Score	Total Score	Query Cover	E value	Per. Ident	Accession
Chloroidium sp. SY-1-2-B small subunit ribosomal RNA gene, partial sequence; internal transcribed spacer 1, 5.8S ribosomal RNA gene, and internal transcribed spacer 2, complete sequence; and large subunit ribosomal RNA gene, partial sequence	222	222	53%	2e-53	89.39%	MT991543.1
Chloroidium sp. NN-2-O small subunit ribosomal RNA gene, partial sequence; internal transcribed spacer 1, 5.8S ribosomal RNA gene, and internal transcribed spacer 2, complete sequence; and large subunit ribosomal RNA gene, partial sequence	222	299	67%	2e-53	89.39%	MT991540.1
Chloroidium ellipsoideum genomic DNA containing 18S rRNA gene, ITS1, 5.8S rRNA gene, ITS2, 28S rRNA gene, culture collection CCAP 211/33	222	299	67%	2e-53	89.39%	FR865666.1
Chloroidium lichenum strain SAG 2295 small subunit ribosomal RNA gene, partial sequence; internal transcribed spacer 1 and 5.8S ribosomal RNA gene, complete sequence; and internal transcribed spacer 2, partial sequence	220	220	75%	8e-53	82.89%	MH551517.1
Chloroidium sp. LV-2018 voucher VancurovaO27 internal transcribed spacer 1, partial sequence; 5.8S ribosomal RNA gene and internal transcribed spacer 2, complete sequence; and large subunit ribosomal RNA gene, partial sequence	220	303	67%	8e-53	89.39%	MH415402.1
Chloroidium ellipsoideum voucher Z7 internal transcribed spacer 1, partial sequence; 5.8S ribosomal RNA gene, complete sequence; and internal transcribed spacer 2, partial sequence	215	215	53%	4e-51	88.83%	OL625378.1
Chloroidium ellipsoideum voucher A689 internal transcribed spacer 1, partial sequence; 5.8S ribosomal RNA gene, complete sequence; and internal transcribed spacer 2, partial sequence	215	215	53%	4e-51	88.83%	OL625330.1
Chloroidium lichenum voucher A67_6 internal transcribed spacer 1, partial sequence; 5.8S ribosomal RNA gene, complete sequence; and internal transcribed spacer 2, partial sequence	215	215	53%	4e-51	88.83%	OL625329.1
Chloroidium lichenum voucher A67_5 internal transcribed spacer 1, partial sequence; 5.8S ribosomal RNA gene and internal transcribed spacer 2, complete sequence; and large subunit ribosomal RNA gene, partial sequence	215	292	68%	4e-51	88.83%	OL625328.1
Chloroidium ellipsoideum voucher A67_4 internal transcribed spacer 1, partial sequence; 5.8S ribosomal RNA gene, complete sequence; and internal transcribed spacer 2, partial sequence	215	215	53%	4e-51	88.83%	OL625327.1

Alignments

uncultured fungus genomic DNA sequence contains ITS1

Sequence ID: **OW836430.1** Length: 358 Number of Matches: 1
Range 1: 24 to 358

Score	Expect	Identities	Gaps	Strand	Frame
608 bits(329)	1e-169()	332/335(99%)	0/335(0%)	Plus/Plus	

Features:

```
Query  1    TGCAGAATTCCGTGAATCATCGAGTCTTTGAACGCATATTGCGCNCGAGGCTTCGGTCAA  60
            |||||||||||||||||||||||||||||||||||||||||||| |||||||||||||||
Sbjct  24   TGCAGAATTCCGTGAATCATCGAGTCTTTGAACGCATATTGCGCCCGAGGCTTCGGTCAA  83

Query  61   GGGCATGTCTGCCTCAGCGTCGGTTGACCCCTCTCGTCCAATTCCCTCGTGGAAGCAGAC  120
            ||||||||||||||||||||||||||||||||||||||||||||||||||||||||||||
Sbjct  84   GGGCATGTCTGCCTCAGCGTCGGTTGACCCCTCTCGTCCAATTCCCTCGTGGAAGCAGAC  143

Query  121  GGACCTGGCAGTCCCGCTGGCGACAGCGGGTCTGCTGCAGAACAGACGACGAGCCAGCCG  180
            ||||||||||||||||||||||||||||||||||||||||||||||||||||||||||||
Sbjct  144  GGACCTGGCAGTCCCGCTGGCGACAGCGGGTCTGCTGCAGAACAGACGACGAGCCAGCCG  203

Query  181  CAGCGCCGCTCGATTGGNAGGTTTTCTCCGGAAGACATGCCGTCGAGNAGCCTGGGGACC  240
            ||||||||||||||||| |||||||||||||||||||||||||||| |||||||||||||
Sbjct  204  CAGCGCCGCTCGATTGGTAGGTTTTCTCCGGAAGACATGCCGTCGAGTAGCCTGGGGACC  263

Query  241  AACTGCCCGTCCTACCAACGACGGGGGAGCGCGCAAGCCATCCCCCTGACTCTCATTTCG  300
            ||||||||||||||||||||||||||||||||||||||||||||||||||||||||||||
Sbjct  264  AACTGCCCGTCCTACCAACGACGGGGGAGCGCGCAAGCCATCCCCCTGACTCTCATTTCG  323

Query  301  ACCTGAGTTCAGGCAAGACCACCCGCTCAACTTAA  335
            |||||||||||||||||||||||||||||||||||
Sbjct  324  ACCTGAGTTCAGGCAAGACCACCCGCTCAACTTAA  358
```

Equisetum arvense voucher MN06 5.8S ribosomal RNA gene, partial sequence; internal transcribed spacer 2, complete sequence; and large subunit ribosomal RNA gene, partial sequence
Sequence ID: **OL304250.1** Length: 335 Number of Matches: 1
Range 1: 1 to 335

Score	Expect	Identities	Gaps	Strand	Frame
608 bits(329)	1e-169()	332/335(99%)	0/335(0%)	Plus/Plus	

Features:

```
Query  1    TGCAGAATTCCGTGAATCATCGAGTCTTTGAACGCATATTGCGCNCGAGGCTTCGGTCAA  60
            |||||||||||||||||||||||||||||||||||||||||||| |||||||||||||||
Sbjct  1    TGCAGAATTCCGTGAATCATCGAGTCTTTGAACGCATATTGCGCCCGAGGCTTCGGTCAA  60

Query  61   GGGCATGTCTGCCTCAGCGTCGGTTGACCCCTCTCGTCCAATTCCCTCGTGGAAGCAGAC  120
            ||||||||||||||||||||||||||||||||||||||||||||||||||||||||||||
Sbjct  61   GGGCATGTCTGCCTCAGCGTCGGTTGACCCCTCTCGTCCAATTCCCTCGTGGAAGCAGAC  120

Query  121  GGACCTGGCAGTCCCGCTGGCGACAGCGGGTCTGCTGCAGAACAGACGACGAGCCAGCCG  180
            ||||||||||||||||||||||||||||||||||||||||||||||||||||||||||||
Sbjct  121  GGACCTGGCAGTCCCGCTGGCGACAGCGGGTCTGCTGCAGAACAGACGACGAGCCAGCCG  180

Query  181  CAGCGCCGCTCGATTGGNAGGTTTTCTCCGGAAGACATGCCGTCGAGNAGCCTGGGGACC  240
            ||||||||||||||||| |||||||||||||||||||||||||||| |||||||||||||
Sbjct  181  CAGCGCCGCTCGATTGGAAGGTTTTCTCCGGAAGACATGCCGTCGAGTAGCCTGGGGACC  240

Query  241  AACTGCCCGTCCTACCAACGACGGGGGAGCGCGCAAGCCATCCCCCTGACTCTCATTTCG  300
            ||||||||||||||||||||||||||||||||||||||||||||||||||||||||||||
Sbjct  241  AACTGCCCGTCCTACCAACGACGGGGGAGCGCGCAAGCCATCCCCCTGACTCTCATTTCG  300

Query  301  ACCTGAGTTCAGGCAAGACCACCCGCTCAACTTAA  335
            |||||||||||||||||||||||||||||||||||
Sbjct  301  ACCTGAGTTCAGGCAAGACCACCCGCTCAACTTAA  335
```

Chloroidium sp. FACHB-2422 small subunit ribosomal RNA gene, partial sequence; internal transcribed spacer 1, 5.8S ribosomal RNA gene, and internal transcribed spacer 2, complete sequence; and large subunit ribosomal RNA gene, partial sequence
Sequence ID: **MT006211.1** Length: 3787 Number of Matches: 1
Range 1: 2888 to 3222

Score	Expect	Identities	Gaps	Strand	Frame
608 bits(329)	1e-169()	332/335(99%)	0/335(0%)	Plus/Plus	

Features:

```
Query  1     TGCAGAATTCCGTGAATCATCGAGTCTTTGAACGCATATTGCGCNCGAGGCTTCGGTCAA  60
             |||||||||||||||||||||||||||||||||||||||||||| |||||||||||||||
Sbjct  2888  TGCAGAATTCCGTGAATCATCGAGTCTTTGAACGCATATTGCGCCCGAGGCTTCGGTCAA  2947

Query  61    GGGCATGTCTGCCTCAGCGTCGGTTGACCCCTCTCGTCCAATTCCCTCGTGGAAGCAGAC  120
             ||||||||||||||||||||||||||||||||||||||||||||||||||||||||||||
Sbjct  2948  GGGCATGTCTGCCTCAGCGTCGGTTGACCCCTCTCGTCCAATTCCCTCGTGGAAGCAGAC  3007

Query  121   GGACCTGGCAGTCCCGCTGGCGACAGCGGGTCTGCTGCAGAACAGACGACGAGCCAGCCG  180
             ||||||||||||||||||||||||||||||||||||||||||||||||||||||||||||
Sbjct  3008  GGACCTGGCAGTCCCGCTGGCGACAGCGGGTCTGCTGCAGAACAGACGACGAGCCAGCCG  3067

Query  181   CAGCGCCGCTCGATTGGNAGGTTTTCTCCGGAAGACATGCCGTCGAGNAGCCTGGGGACC  240
             ||||||||||||||||| |||||||||||||||||||||||||||||| |||||||||||||
Sbjct  3068  CAGCGCCGCTCGATTGGTAGGTTTTCTCCGGAAGACATGCCGTCGAGTAGCCTGGGGACC  3127

Query  241   AACTGCCCGTCCTACCAACGACGGGGGAGCGCGCAAGCCATCCCCCTGACTCTCATTTCG  300
             ||||||||||||||||||||||||||||||||||||||||||||||||||||||||||||
Sbjct  3128  AACTGCCCGTCCTACCAACGACGGGGGAGCGCGCAAGCCATCCCCCTGACTCTCATTTCG  3187

Query  301   ACCTGAGTTCAGGCAAGACCACCCGCTCAACTTAA  335
             |||||||||||||||||||||||||||||||||||
Sbjct  3188  ACCTGAGTTCAGGCAAGACCACCCGCTCAACTTAA  3222
```

Equisetum arvense voucher 34008 5.8S ribosomal RNA gene, partial sequence; internal transcribed spacer 2, complete sequence; and large subunit ribosomal RNA gene, partial sequence

Sequence ID: **MW040322.1** Length: 335 Number of Matches: 1
Range 1: 1 to 335

Score	Expect	Identities	Gaps	Strand	Frame
608 bits(329)	1e-169()	335/335(100%)	0/335(0%)	Plus/Plus	

Features:

```
Query  1     TGCAGAATTCCGTGAATCATCGAGTCTTTGAACGCATATTGCGCNCGAGGCTTCGGTCAA  60
             ||||||||||||||||||||||||||||||||||||||||||||||||||||||||||||
Sbjct  1     TGCAGAATTCCGTGAATCATCGAGTCTTTGAACGCATATTGCGCNCGAGGCTTCGGTCAA  60

Query  61    GGGCATGTCTGCCTCAGCGTCGGTTGACCCCTCTCGTCCAATTCCCTCGTGGAAGCAGAC  120
             ||||||||||||||||||||||||||||||||||||||||||||||||||||||||||||
Sbjct  61    GGGCATGTCTGCCTCAGCGTCGGTTGACCCCTCTCGTCCAATTCCCTCGTGGAAGCAGAC  120

Query  121   GGACCTGGCAGTCCCGCTGGCGACAGCGGGTCTGCTGCAGAACAGACGACGAGCCAGCCG  180
             ||||||||||||||||||||||||||||||||||||||||||||||||||||||||||||
Sbjct  121   GGACCTGGCAGTCCCGCTGGCGACAGCGGGTCTGCTGCAGAACAGACGACGAGCCAGCCG  180

Query  181   CAGCGCCGCTCGATTGGNAGGTTTTCTCCGGAAGACATGCCGTCGAGNAGCCTGGGGACC  240
             ||||||||||||||||||||||||||||||||||||||||||||||||||||||||||||
Sbjct  181   CAGCGCCGCTCGATTGGNAGGTTTTCTCCGGAAGACATGCCGTCGAGNAGCCTGGGGACC  240

Query  241   AACTGCCCGTCCTACCAACGACGGGGGAGCGCGCAAGCCATCCCCCTGACTCTCATTTCG  300
             ||||||||||||||||||||||||||||||||||||||||||||||||||||||||||||
Sbjct  241   AACTGCCCGTCCTACCAACGACGGGGGAGCGCGCAAGCCATCCCCCTGACTCTCATTTCG  300

Query  301   ACCTGAGTTCAGGCAAGACCACCCGCTCAACTTAA  335
             |||||||||||||||||||||||||||||||||||
Sbjct  301   ACCTGAGTTCAGGCAAGACCACCCGCTCAACTTAA  335
```

Chloroidium saccharophilum genomic DNA containing 18S rRNA gene, ITS1, 5.8S rRNA gene, ITS2, 28S rRNA gene, culture collection CCAP 211/31

Sequence ID: **FR865664.1** Length: 2988 Number of Matches: 1
Range 1: 2460 to 2794

Score	Expect	Identities	Gaps	Strand	Frame
603 bits(326)	7e-168()	331/335(99%)	0/335(0%)	Plus/Plus	

Features:

```
Query  1     TGCAGAATTCCGTGAATCATCGAGTCTTTGAACGCATATTGCGCNCGAGGCTTCGGTCAA  60
             |||||||||||||||||||||||||||||||||||||||||||| |||||||||||||||
Sbjct  2460  TGCAGAATTCCGTGAATCATCGAGTCTTTGAACGCATATTGCGCCCGAGGCTTCGGTCAA  2519

Query  61    GGGCATGTCTGCCTCAGCGTCGGTTGACCCCTCTCGTCCAATTCCCTCGTGGAAGCAGAC  120
             ||||||||||||||||||||||||||||||||||||||||||||||||||||||||||||
Sbjct  2520  GGGCATGTCTGCCTCAGCGTCGGTTGACCCCTCTCGTCCAATTCCCTCGTGGAAGCAGAC  2579

Query  121   GGACCTGGCAGTCCCGCTGGCGACAGCGGGTCTGCTGCAGAACAGACGACGAGCCAGCCG  180
             ||||||||||||||||||||||||||||||||||||||||||||||||||||||||||||
Sbjct  2580  GGACCTGGCAGTCCCGCTGGCGACAGCGGGTCTGCTGCAGAACAGACGACGAGCCAGCCG  2639
```

```
Query  181   CAGCGCCGCTCGATTGGNAGGTTTTCTCCGGAAGACATGCCGTCGAGNAGCCTGGGGACC  240
             ||||||||||||||||| ||||||||||||||||||||||||||||| ||||||||||||
Sbjct  2640  CAGCGCCGCTCGATTGGTAGGTTTTCTCCGGAAGACATGCCGTCGAGTAGCCTGGGGACC  2699

Query  241   AACTGCCCGTCCTACCAACGACGGGGGAGCGCGCAAGCCATCCCCCTGACTCTCATTTCG  300
             |||||||||||||||||||||||||||||||||||||| |||||||||||||||||||||
Sbjct  2700  AACTGCCCGTCCTACCAACGACGGGGGAGCGCGCAAGCTATCCCCCTGACTCTCATTTCG  2759

Query  301   ACCTGAGTTCAGGCAAGACCACCCGCTCAACTTAA  335
             |||||||||||||||||||||||||||||||||||
Sbjct  2760  ACCTGAGTTCAGGCAAGACCACCCGCTCAACTTAA  2794
```

BLAST is a registered trademark of the National Library of Medicine
