# Supplementary material for: A Multi-Pronged Technique for Identifying Equisetum palustre and Equisetum arvense—Combining HPTLC, HPLC-ESI-MS/MS and Optimized DNA Barcoding Techniques
Source: Plants (Basel). 2022 Sep 28;11(19):2562. doi: 10.3390/plants11192562 (PMC9573248; doi:10.3390/plants11192562)
Supplement: Supplementary file 1 [file plants-11-02562-s001.zip › plants-1932149 supplementary/Horsetail Supplementary Figures/Figure S5.pdf]

BLAST Results

Questions/comments

Job title: MW040323.1

**RID**	FD5DU9B3016 (Expires on 08-14 02:02 am)		
**Query ID**	lcl|Query_61545	**Database Name**	nt
**Description**	None	**Description**	Nucleotide collection (nt)
**Molecule type**	dna	**Program**	BLASTN 2.13.0+
**Query Length**	335		

Graphic Summary

Distribution of the top 108 Blast Hits on 100 subject sequences

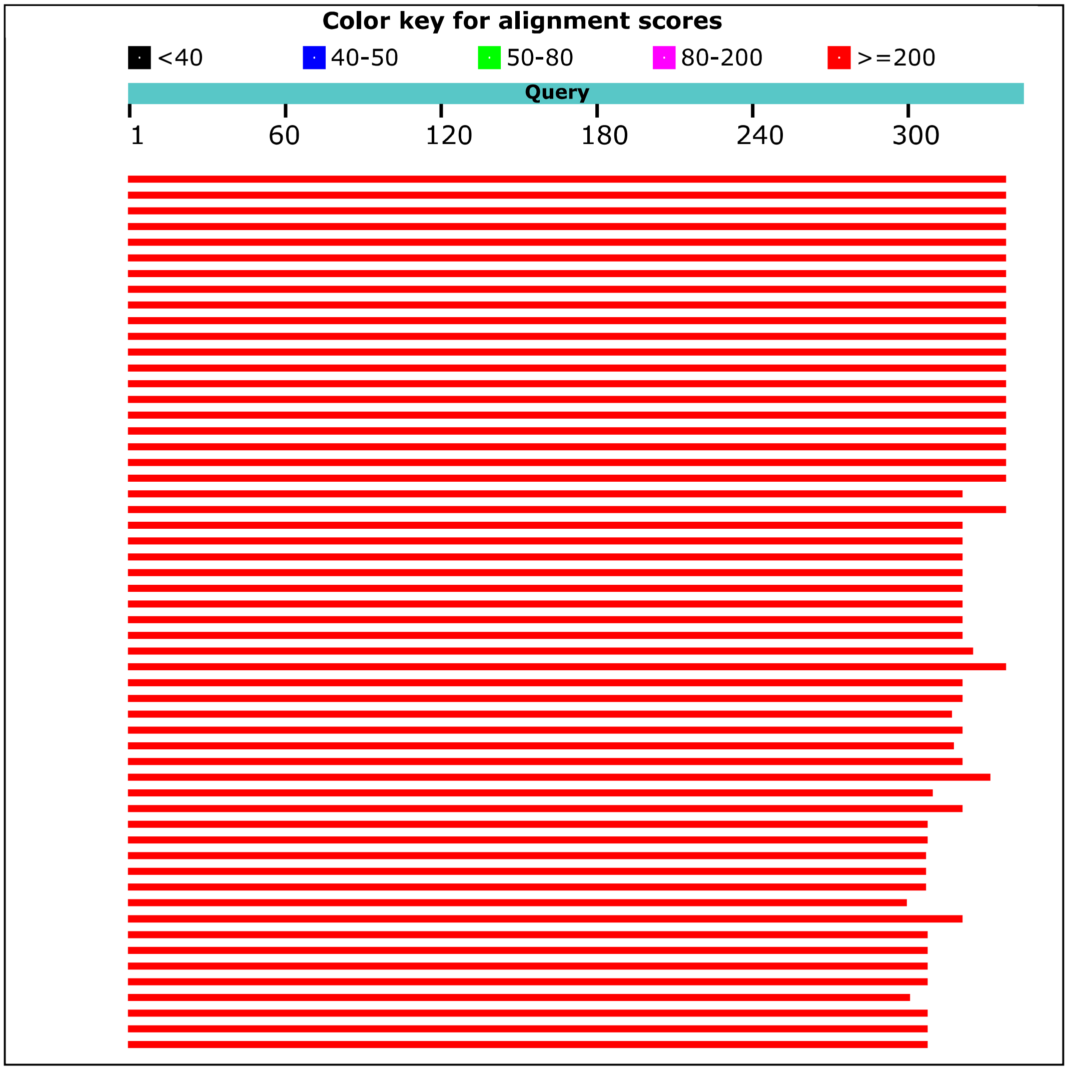

Descriptions

Sequences producing significant alignments:

Description	Max Score	Total Score	Query Cover	E value	Per. Ident	Accession
uncultured fungus genomic DNA sequence contains ITS1	612	612	100%	1e-170	99.40%	OW836430.1
Chloroidium saccharophilum genomic DNA containing 18S rRNA gene, ITS1, 5.8S rRNA gene, ITS2, 28S rRNA gene, culture collection CCAP 211/31	612	612	100%	1e-170	99.40%	FR865664.1
Chloroidium sp. FACHB-2422 small subunit ribosomal RNA gene, partial sequence; internal transcribed spacer 1, 5.8S ribosomal RNA gene, and internal transcribed spacer 2, complete sequence; and large subunit ribosomal RNA gene, partial sequence	612	612	100%	1e-170	99.40%	MT006211.1
Equisetum arvense voucher Equisetum_arvense_ITS.fasta_2 5.8S ribosomal RNA gene, partial sequence; internal transcribed spacer 2, complete sequence; and large subunit ribosomal RNA gene, partial sequence	612	612	100%	1e-170	100.00%	MW040323.1
Equisetum arvense voucher MN06 5.8S ribosomal RNA gene, partial sequence; internal transcribed spacer 2, complete sequence; and large subunit ribosomal RNA gene, partial sequence	606	606	100%	5e-169	99.10%	OL304250.1
Chloroidium saccharophilum strain FACHB-1796 18S ribosomal RNA gene, partial sequence; internal transcribed spacer 1, 5.8S ribosomal RNA gene, and internal transcribed spacer 2, complete sequence; and 28S ribosomal RNA gene, partial sequence	601	601	100%	3e-167	98.81%	KX024689.1
Equisetum arvense voucher MN05 5.8S ribosomal RNA gene, partial sequence; internal transcribed spacer 2, complete sequence; and large subunit ribosomal RNA gene, partial sequence	601	601	100%	3e-167	98.81%	OL304249.1
uncultured Pezizaceae genomic DNA sequence contains ITS1	601	601	100%	3e-167	98.81%	OU496020.1
Equisetum arvense voucher 34008 5.8S ribosomal RNA gene, partial sequence; internal transcribed spacer 2, complete sequence; and large subunit ribosomal RNA gene, partial sequence	601	601	100%	3e-167	98.51%	MW040322.1
Equisetum arvense voucher Equisetum_arvense_ITS.fasta_1 5.8S ribosomal RNA gene, partial sequence; internal transcribed spacer 2, complete sequence; and large subunit ribosomal RNA gene, partial sequence	597	597	100%	3e-166	98.21%	MW040324.1
Unclassified Viridiplantae clone 1537 5.8S ribosomal RNA gene, partial sequence; internal transcribed spacer 2, complete sequence; and large subunit ribosomal RNA gene, partial sequence	595	595	100%	1e-165	98.51%	MF570051.1

Description	Max Score	Total Score	Query Cover	E value	Per. Ident	Accession
Chloroidium ellipsoideum isolate FG2/4.5E 18S ribosomal RNA gene, partial sequence; internal transcribed spacer 1, 5.8S ribosomal RNA gene, and internal transcribed spacer 2, complete sequence; and 26S ribosomal RNA gene, partial sequence	595	595	100%	1e-165	98.51%	KM020071.1
Chloroidium saccharophilum isolate FG2/1.3 18S ribosomal RNA gene, partial sequence; internal transcribed spacer 1, 5.8S ribosomal RNA gene, and internal transcribed spacer 2, complete sequence; and 26S ribosomal RNA gene, partial sequence	595	595	100%	1e-165	98.51%	KM020070.1
Chloroidium saccharophilum var. saccharophilum strain UTEX247 18S ribosomal RNA gene, partial sequence; internal transcribed spacer 1, 5.8S ribosomal RNA gene, and internal transcribed spacer 2, complete sequence; and 28S ribosomal RNA gene, partial sequence	595	595	100%	1e-165	98.51%	KJ676130.1
Chloroidium saccharophilum strain OD-1-1-C small subunit ribosomal RNA gene, partial sequence; internal transcribed spacer 1, 5.8S ribosomal RNA gene, and internal transcribed spacer 2, complete sequence; and large subunit ribosomal RNA gene, partial sequence	595	595	100%	1e-165	98.51%	MT991539.1
Chloroidium ellipsoideum genomic DNA containing 18S rRNA gene, ITS1, 5.8S rRNA gene, ITS2, 28S rRNA gene, culture collection CCAP 211/40	595	595	100%	1e-165	98.51%	FR865669.1
Chloroidium saccharophilum genomic DNA containing 18S rRNA gene, ITS1, 5.8S rRNA gene, ITS2, 28S rRNA gene, culture collection CCAP 211/32	595	595	100%	1e-165	98.51%	FR865665.1
Equisetum arvense voucher MN01 5.8S ribosomal RNA gene, partial sequence; internal transcribed spacer 2, complete sequence; and large subunit ribosomal RNA gene, partial sequence	593	593	100%	4e-165	98.51%	OL304248.1
Chloroidium saccharophilum var. saccharophilum strain UTEX2469 18S ribosomal RNA gene, partial sequence; internal transcribed spacer 1, 5.8S ribosomal RNA gene, and internal transcribed spacer 2, complete sequence; and 28S ribosomal RNA gene, partial sequence	584	584	100%	3e-162	97.91%	KJ676131.1
Chloroidium sp. FACHB-2420 small subunit ribosomal RNA gene, partial sequence; internal transcribed spacer 1, 5.8S ribosomal RNA gene, and internal transcribed spacer 2, complete sequence; and large subunit ribosomal RNA gene, partial sequence	584	584	100%	3e-162	97.91%	MT006209.1
Chloroidium saccharophilum strain ISBAL-007 18S ribosomal RNA gene, internal transcribed spacer 1, 5.8S ribosomal RNA gene, internal transcribed spacer 2, and 28S ribosomal RNA gene, region	580	580	94%	3e-161	99.37%	MH551492.1

Description	Max Score	Total Score	Query Cover	E value	Per. Ident	Accession
'Chlorella' ellipsoidea strain Ce 18S ribosomal RNA gene, partial sequence; internal transcribed spacer 1, 5.8S ribosomal RNA gene, and internal transcribed spacer 2, complete sequence; and 26S ribosomal RNA gene, partial sequence	579	579	100%	1e-160	97.61%	EU038292.1
Chlorella saccharophila partial 18S rRNA gene, ITS1, 5.8S rRNA, ITS2 and partial 28S rRNA gene, strain SAG 2120	569	569	94%	7e-158	98.74%	FM946008.1
Chlorella saccharophila partial 18S rRNA gene, ITS1, 5.8S rRNA, ITS2 and partial 28S rRNA gene, strain CCAP 211/42	569	569	94%	7e-158	98.74%	FM946004.1
Chlorella saccharophila partial 18S rRNA gene, ITS1, 5.8S rRNA, ITS2 and partial 28S rRNA gene, strain CCAP 211/36	569	569	94%	7e-158	98.74%	FM946003.1
Chloroidium saccharophilum strain CCAP 211/27 18S ribosomal RNA gene, internal transcribed spacer 1, 5.8S ribosomal RNA gene, internal transcribed spacer 2, and 28S ribosomal RNA gene, region	564	564	94%	3e-156	98.43%	MH551491.1
Chloroidium saccharophilum strain CCAP 211/40 small subunit ribosomal RNA gene, partial sequence; internal transcribed spacer 1 and 5.8S ribosomal RNA gene, complete sequence; and internal transcribed spacer 2, partial sequence	564	564	94%	3e-156	98.43%	MH551490.1
Chlorella saccharophila partial 18S rRNA gene, ITS1, 5.8S rRNA, ITS2 and partial 28S rRNA gene, strain SAG 2197	564	564	94%	3e-156	98.43%	FM946010.1
Chlorella saccharophila partial 18S rRNA gene, ITS1, 5.8S rRNA, ITS2 and partial 28S rRNA gene, strain SAG 211-1c	564	564	94%	3e-156	98.43%	FM946007.1
Chlorella saccharophila partial 18S rRNA gene, ITS1, 5.8S rRNA, ITS2 and partial 28S rRNA gene, strain CCAP 211/32	564	564	94%	3e-156	98.43%	FM946001.1
Chlorella sp. UTEX 2068 18S ribosomal RNA gene, partial sequence; internal transcribed spacer 1, 5.8S ribosomal RNA gene, and internal transcribed spacer 2, complete sequence; and 28S ribosomal RNA gene, partial sequence	560	560	96%	4e-155	97.83%	KJ676132.1
Chloroidium saccharophilum genomic DNA containing 18S rRNA gene, ITS1, 5.8S rRNA gene, ITS2, 28S rRNA gene, culture collection CCAP 211/58	560	560	100%	4e-155	96.72%	FR865677.1
Chloroidium saccharophilum strain FaII/B-1b small subunit ribosomal RNA gene, partial sequence; internal transcribed spacer 1 and 5.8S ribosomal RNA gene, complete sequence; and internal transcribed spacer 2, partial sequence	558	558	94%	2e-154	98.11%	MH551489.1
Chloroidium saccharophilum strain SAG 2055 small subunit ribosomal RNA gene, partial sequence; internal transcribed spacer 1 and 5.8S ribosomal RNA gene, complete sequence; and internal transcribed spacer 2, partial sequence	558	558	94%	2e-154	98.11%	MH551488.1

Description	Max Score	Total Score	Query Cover	E value	Per. Ident	Accession
Chloroidium saccharophilum strain KMMCC 192 18S ribosomal RNA gene, internal transcribed spacer 1, 5.8S ribosomal RNA gene, internal transcribed spacer 2, and 28S ribosomal RNA gene, region	556	556	93%	5e-154	98.41%	JQ315766.1
Chlorella saccharophila partial 18S rRNA gene, ITS1, 5.8S rRNA, ITS2 and partial 28S rRNA gene, strain SAG 211-1b	556	556	94%	5e-154	98.11%	FM946005.1
Uncultured alga genes for 5.8S rRNA, ITS2, 28S rRNA, partial and complete sequence, note: OTU163	553	553	94%	7e-153	98.10%	LC381753.1
Chlorella saccharophila partial 18S rRNA gene, ITS1, 5.8S rRNA, ITS2 and partial 28S rRNA gene, strain SAG 211-9a	553	553	94%	7e-153	97.80%	FM946000.1
Chloroidium saccharophilum strain KMMCC 158 18S ribosomal RNA gene, internal transcribed spacer 1, 5.8S ribosomal RNA gene, internal transcribed spacer 2, and 28S ribosomal RNA gene, region	545	545	98%	1e-150	96.37%	JQ315771.1
Chloroidium saccharophilum strain KMMCC 195 18S ribosomal RNA gene, internal transcribed spacer 1, 5.8S ribosomal RNA gene, internal transcribed spacer 2, and 28S ribosomal RNA gene, region	538	538	91%	2e-148	98.05%	JQ315769.1
Chloroidium saccharophilum strain CCAP 264/2 small subunit ribosomal RNA gene, partial sequence; internal transcribed spacer 1 and 5.8S ribosomal RNA gene, complete sequence; and internal transcribed spacer 2, partial sequence	534	534	94%	3e-147	96.86%	MH551493.1
Uncultured Chloroidium isolate A207 internal transcribed spacer 1, partial sequence; 5.8S ribosomal RNA gene, complete sequence; and internal transcribed spacer 2, partial sequence	534	534	91%	3e-147	98.03%	KP314644.1
Uncultured Chloroidium isolate A103 internal transcribed spacer 1, partial sequence; 5.8S ribosomal RNA gene, complete sequence; and internal transcribed spacer 2, partial sequence	534	534	91%	3e-147	98.03%	KP314563.1
Chloroidium saccharophilum strain St6 small subunit ribosomal RNA gene, partial sequence; internal transcribed spacer 1 and 5.8S ribosomal RNA gene, complete sequence; and internal transcribed spacer 2, partial sequence	532	532	90%	9e-147	98.03%	OM472016.1
Chloroidium saccharophilum strain ACSSI 340 small subunit ribosomal RNA gene, partial sequence; internal transcribed spacer 1 and 5.8S ribosomal RNA gene, complete sequence; and internal transcribed spacer 2, partial sequence	532	532	90%	9e-147	98.03%	MT827198.1
Chloroidium saccharophilum strain ACSSI 339 small subunit ribosomal RNA gene, partial sequence; internal transcribed spacer 1 and 5.8S ribosomal RNA gene, complete sequence; and internal transcribed spacer 2, partial sequence	532	532	90%	9e-147	98.03%	MT827197.1

Description	Max Score	Total Score	Query Cover	E value	Per. Ident	Accession
Chloroidium saccharophilum strain CCM-UDEC 143 18S ribosomal RNA gene, partial sequence; and internal transcribed spacer 1, 5S ribosomal RNA gene, and internal transcribed spacer 2, complete sequence	531	531	88%	3e-146	98.65%	KC517116.1
Chloroidium saccharophilum strain CCAP 211/58 small subunit ribosomal RNA gene, partial sequence; internal transcribed spacer 1 and 5.8S ribosomal RNA gene, complete sequence; and internal transcribed spacer 2, partial sequence	529	529	94%	1e-145	96.54%	MH551494.1
Uncultured Chloroidium isolate A214 internal transcribed spacer 1, partial sequence; 5.8S ribosomal RNA gene, complete sequence; and internal transcribed spacer 2, partial sequence	529	529	91%	1e-145	97.70%	KP314651.1
Uncultured Chloroidium isolate A032 internal transcribed spacer 1, partial sequence; 5.8S ribosomal RNA gene, complete sequence; and internal transcribed spacer 2, partial sequence	529	529	91%	1e-145	97.70%	KP314515.1
Uncultured Chloroidium isolate A029 internal transcribed spacer 1, partial sequence; 5.8S ribosomal RNA gene, complete sequence; and internal transcribed spacer 2, partial sequence	529	529	91%	1e-145	97.70%	KP314513.1
Uncultured Chloroidium isolate A011 internal transcribed spacer 1, partial sequence; 5.8S ribosomal RNA gene, complete sequence; and internal transcribed spacer 2, partial sequence	529	529	91%	1e-145	97.70%	KP314497.1
Chloroidium saccharophilum genomic DNA sequence contains 5.8S rRNA gene, ITS2, strain Chlorella saccharophila RNY	527	527	88%	4e-145	98.33%	LR215745.1
Uncultured Chloroidium isolate A023 internal transcribed spacer 1, partial sequence; 5.8S ribosomal RNA gene, complete sequence; and internal transcribed spacer 2, partial sequence	521	521	91%	2e-143	97.38%	KP314508.1
Uncultured Chloroidium isolate A141 internal transcribed spacer 1, partial sequence; 5.8S ribosomal RNA gene, complete sequence; and internal transcribed spacer 2, partial sequence	516	516	91%	9e-142	97.05%	KP314596.1
Uncultured Chloroidium isolate A217 internal transcribed spacer 1, partial sequence; 5.8S ribosomal RNA gene, complete sequence; and internal transcribed spacer 2, partial sequence	510	510	91%	4e-140	96.72%	KP314654.1
'Chlorella' saccharophila voucher UTEX 247 internal transcribed spacer 1, partial sequence; 5.8S ribosomal RNA gene, complete sequence; and internal transcribed spacer 2, partial sequence	503	503	91%	7e-138	95.81%	EF120783.1

Description	Max Score	Total Score	Query Cover	E value	Per. Ident	Accession
Chloroidium saccharophilum voucher Herbarium Tuerk51488 internal transcribed spacer 1, partial sequence; 5.8S ribosomal RNA gene, complete sequence; and internal transcribed spacer 2, partial sequence	501	501	84%	3e-137	98.24%	KF907674.1
Chloroidium saccharophilum voucher Herbarium Tuerk51481 internal transcribed spacer 1, partial sequence; 5.8S ribosomal RNA gene, complete sequence; and internal transcribed spacer 2, partial sequence	501	501	84%	3e-137	98.24%	KF907673.1
Chloroidium saccharophilum voucher Herbarium Tuerk51492 internal transcribed spacer 1, partial sequence; 5.8S ribosomal RNA gene, complete sequence; and internal transcribed spacer 2, partial sequence	496	496	84%	1e-135	97.89%	KF907675.1
Chloroidium saccharophilum voucher Herbarium Tuerk51480 internal transcribed spacer 1, partial sequence; 5.8S ribosomal RNA gene, complete sequence; and internal transcribed spacer 2, partial sequence	496	496	84%	1e-135	97.89%	KF907672.1
'Chlorella' saccharophila voucher UTEX 2911 internal transcribed spacer 1, partial sequence; 5.8S ribosomal RNA gene, complete sequence; and internal transcribed spacer 2, partial sequence	479	479	81%	1e-130	98.17%	EF120782.1
Chloroidium sp. S4MWC-51 internal transcribed spacer 1, 5.8S ribosomal RNA gene, and internal transcribed spacer 2, complete sequence	470	470	79%	7e-128	98.49%	MK005103.1
Uncultured Chloroidium isolate A026 internal transcribed spacer 1, partial sequence; 5.8S ribosomal RNA gene, complete sequence; and internal transcribed spacer 2, partial sequence	435	435	91%	3e-117	92.16%	KP314510.1
'Chlorella' saccharophila voucher UTEX 2469 internal transcribed spacer 1, partial sequence; 5.8S ribosomal RNA gene, complete sequence; and internal transcribed spacer 2, partial sequence	418	418	70%	3e-112	98.31%	EF120784.1
Chloroidium saccharophilum strain KMMCC 192 18S ribosomal RNA gene, internal transcribed spacer 1, 5.8S ribosomal RNA gene, internal transcribed spacer 2, and 28S ribosomal RNA gene, region	359	359	59%	2e-94	98.99%	JQ315767.1
Uncultured alga isolate ASV_573_A 5.8S ribosomal RNA gene, partial sequence; internal transcribed spacer 2, complete sequence; and large subunit ribosomal RNA gene, partial sequence	276	276	100%	2e-69	81.67%	ON119385.1
Chloroidium viscosum strain SAG 56.87 18S ribosomal RNA gene, internal transcribed spacer 1, 5.8S ribosomal RNA gene, internal transcribed spacer 2, and 28S ribosomal RNA gene, region	255	255	94%	2e-63	81.34%	MH551495.1

Description	Max Score	Total Score	Query Cover	E value	Per. Ident	Accession
Chloroidium angustoellipsoideum voucher VancurovaA356 internal transcribed spacer 1, partial sequence; 5.8S ribosomal RNA gene and internal transcribed spacer 2, complete sequence; and large subunit ribosomal RNA gene, partial sequence	254	254	100%	8e-63	81.79%	MH415249.1
uncultured fungus genomic DNA sequence contains ITS1	250	250	100%	1e-61	80.28%	OW836154.1
Chloroidium lichenum strain BII/C-1 small subunit ribosomal RNA gene, partial sequence; internal transcribed spacer 1 and 5.8S ribosomal RNA gene, complete sequence; and internal transcribed spacer 2, partial sequence	244	244	77%	5e-60	84.39%	MH551507.1
Chloroidium lichenum strain MG-4 small subunit ribosomal RNA gene, partial sequence; internal transcribed spacer 1 and 5.8S ribosomal RNA gene, complete sequence; and internal transcribed spacer 2, partial sequence	244	244	77%	5e-60	84.39%	MH551506.1
Chloroidium lichenum strain BII/B-9 small subunit ribosomal RNA gene, partial sequence; internal transcribed spacer 1 and 5.8S ribosomal RNA gene, complete sequence; and internal transcribed spacer 2, partial sequence	244	244	77%	5e-60	84.39%	MH551505.1
Chloroidium viscosum strain MG-2 small subunit ribosomal RNA gene, partial sequence; internal transcribed spacer 1 and 5.8S ribosomal RNA gene, complete sequence; and internal transcribed spacer 2, partial sequence	244	244	94%	5e-60	80.76%	MH551496.1
Uncultured Chlorella clone CM-E-A25 internal transcribed spacer 1, partial sequence; 5.8S ribosomal RNA gene, complete sequence; and internal transcribed spacer 2, partial sequence	244	244	77%	5e-60	84.39%	KJ489091.1
Chlorella angustoellipsoidea partial 18S rRNA gene, ITS1, 5.8S rRNA, ITS2 and partial 28S rRNA gene, strain SAG 2115	244	244	77%	5e-60	84.39%	FM946019.1
Chlorella angustoellipsoidea partial 18S rRNA gene, ITS1, 5.8S rRNA, ITS2 and partial 28S rRNA gene, strain SAG 2144	244	244	77%	5e-60	84.39%	FM946020.1
Chloroidium lichenum strain SAG 2293 18S ribosomal RNA gene, internal transcribed spacer 1, 5.8S ribosomal RNA gene, internal transcribed spacer 2, and 28S ribosomal RNA gene, region	241	241	67%	6e-59	86.02%	MH551510.1
Chloroidium lichenum strain SAG 2294 18S ribosomal RNA gene, internal transcribed spacer 1, 5.8S ribosomal RNA gene, internal transcribed spacer 2, and 28S ribosomal RNA gene, region	241	241	67%	6e-59	86.02%	MH551509.1

Description	Max Score	Total Score	Query Cover	E value	Per. Ident	Accession
Chloroidium sp. LV-2018 voucher VancurovaA318 small subunit ribosomal RNA gene, partial sequence; internal transcribed spacer 1 and 5.8S ribosomal RNA gene, complete sequence; and internal transcribed spacer 2, partial sequence	241	241	67%	6e-59	86.02%	MH415228.1
Uncultured alga genes for 5.8S rRNA, ITS2, 28S rRNA, partial and complete sequence, note: OTU71	241	241	53%	6e-59	90.71%	LC381752.1
Chloroidium lichenum strain SAG 2141 18S ribosomal RNA gene, internal transcribed spacer 1, 5.8S ribosomal RNA gene, internal transcribed spacer 2, and 28S ribosomal RNA gene, region	239	239	76%	2e-58	83.96%	MH551512.1
Chloroidium sp. LV-2018 voucher VancurovaA503 small subunit ribosomal RNA gene, partial sequence; internal transcribed spacer 1, 5.8S ribosomal RNA gene, and internal transcribed spacer 2, complete sequence; and large subunit ribosomal RNA gene, partial sequence	239	312	88%	2e-58	83.76%	MH415295.1
Chloroidium angustoellipsoideum voucher VancurovaA421.2 internal transcribed spacer 1, partial sequence; 5.8S ribosomal RNA gene and internal transcribed spacer 2, complete sequence; and large subunit ribosomal RNA gene, partial sequence	237	319	90%	8e-58	84.01%	MH415277.1
Chloroidium orientale strain VCA-45 (Ce2E) small subunit ribosomal RNA gene, partial sequence; internal transcribed spacer 1, 5.8S ribosomal RNA gene, and internal transcribed spacer 2, complete sequence; and large subunit ribosomal RNA gene, partial sequence	237	237	94%	8e-58	80.59%	MZ558750.1
Chloroidium sp. LV-2018 voucher VancurovaO29 small subunit ribosomal RNA gene, partial sequence; internal transcribed spacer 1, 5.8S ribosomal RNA gene, and internal transcribed spacer 2, complete sequence; and large subunit ribosomal RNA gene, partial sequence	233	310	89%	1e-56	84.03%	MH415404.1
Chloroidium lichenum voucher A114 internal transcribed spacer 1, partial sequence; 5.8S ribosomal RNA gene, complete sequence; and internal transcribed spacer 2, partial sequence	233	233	67%	1e-56	85.53%	OL625124.1
Chloroidium lichenum strain QII/C-5b small subunit ribosomal RNA gene, partial sequence; internal transcribed spacer 1 and 5.8S ribosomal RNA gene, complete sequence; and internal transcribed spacer 2, partial sequence	231	231	67%	4e-56	85.11%	MH551508.1
Chloroidium ellipsoideum voucher RidkaNZ23 internal transcribed spacer 1, partial sequence; 5.8S ribosomal RNA gene, complete sequence; and internal transcribed spacer 2, partial sequence	231	231	53%	4e-56	90.50%	MH415207.1

Description	Max Score	Total Score	Query Cover	E value	Per. Ident	Accession
Chloroidium lichenum strain CCAP 211/33 18S ribosomal RNA gene, internal transcribed spacer 1, 5.8S ribosomal RNA gene, internal transcribed spacer 2, and 28S ribosomal RNA gene, region	230	230	76%	1e-55	83.27%	MH551518.1
Chloroidium sp. Ru-6-6 small subunit ribosomal RNA gene, partial sequence; internal transcribed spacer 1, 5.8S ribosomal RNA gene, and internal transcribed spacer 2, complete sequence; and large subunit ribosomal RNA gene, partial sequence	230	306	90%	1e-55	83.27%	MH703747.1
Chloroidium sp. SY-1-2-B small subunit ribosomal RNA gene, partial sequence; internal transcribed spacer 1, 5.8S ribosomal RNA gene, and internal transcribed spacer 2, complete sequence; and large subunit ribosomal RNA gene, partial sequence	230	230	76%	1e-55	83.27%	MT991543.1
Chloroidium sp. NN-2-O small subunit ribosomal RNA gene, partial sequence; internal transcribed spacer 1, 5.8S ribosomal RNA gene, and internal transcribed spacer 2, complete sequence; and large subunit ribosomal RNA gene, partial sequence	230	306	90%	1e-55	83.27%	MT991540.1
Chloroidium ellipsoideum genomic DNA containing 18S rRNA gene, ITS1, 5.8S rRNA gene, ITS2, 28S rRNA gene, culture collection CCAP 211/33	230	306	90%	1e-55	83.27%	FR865666.1
Chloroidium lichenum strain SAG 2295 small subunit ribosomal RNA gene, partial sequence; internal transcribed spacer 1 and 5.8S ribosomal RNA gene, complete sequence; and internal transcribed spacer 2, partial sequence	228	228	75%	5e-55	83.65%	MH551517.1
Chloroidium sp. LV-2018 voucher VancurovaO27 internal transcribed spacer 1, partial sequence; 5.8S ribosomal RNA gene and internal transcribed spacer 2, complete sequence; and large subunit ribosomal RNA gene, partial sequence	228	310	89%	5e-55	83.65%	MH415402.1
Chloroidium lichenum voucher A67_6 internal transcribed spacer 1, partial sequence; 5.8S ribosomal RNA gene, complete sequence; and internal transcribed spacer 2, partial sequence	222	222	75%	2e-53	83.33%	OL625329.1
Chloroidium lichenum voucher A67_5 internal transcribed spacer 1, partial sequence; 5.8S ribosomal RNA gene and internal transcribed spacer 2, complete sequence; and large subunit ribosomal RNA gene, partial sequence	222	299	89%	2e-53	83.33%	OL625328.1
Chloroidium lichenum voucher A67_3 internal transcribed spacer 1, partial sequence; 5.8S ribosomal RNA gene, complete sequence; and internal transcribed spacer 2, partial sequence	222	222	75%	2e-53	83.33%	OL625326.1

Description	Max Score	Total Score	Query Cover	E value	Per. Ident	Accession
Chloroidium lichenum voucher A65_1 internal transcribed spacer 1, partial sequence; 5.8S ribosomal RNA gene, complete sequence; and internal transcribed spacer 2, partial sequence	222	222	75%	2e-53	83.33%	OL625324.1

Alignments

uncultured fungus genomic DNA sequence contains ITS1

Sequence ID: **OW836430.1** Length: 358 Number of Matches: 1

Range 1: 24 to 358

Score	Expect	Identities	Gaps	Strand	Frame
612 bits(331)	1e-170()	333/335(99%)	0/335(0%)	Plus/Plus	

Features:

```
Query  1    TGCAGAATTCCGTGAATCATCGAGTCTTTGAACGCATATTGCGCCCGAGGCTTCGGTCNA  60
            |||||||||||||||||||||||||||||||||||||||||||||||||||||||||| |
Sbjct  24   TGCAGAATTCCGTGAATCATCGAGTCTTTGAACGCATATTGCGCCCGAGGCTTCGGTCAA  83

Query  61   GGGCATGTCTGCCTCAGCGTCGGTTGACCCCTCTCGTCCAATTCCCTCGTGGAAGCAGAC  120
            ||||||||||||||||||||||||||||||||||||||||||||||||||||||||||||
Sbjct  84   GGGCATGTCTGCCTCAGCGTCGGTTGACCCCTCTCGTCCAATTCCCTCGTGGAAGCAGAC  143

Query  121  GGACCTGGCAGTCCCGCTGGCGACAGCGGGTCTGCTGCAGAACAGACGACGAGCCAGCCG  180
            ||||||||||||||||||||||||||||||||||||||||||||||||||||||||||||
Sbjct  144  GGACCTGGCAGTCCCGCTGGCGACAGCGGGTCTGCTGCAGAACAGACGACGAGCCAGCCG  203

Query  181  CAGCGCCGCTCGATTGGTAGGTTTTCTCCGGAAGACATGCCGTCGAGTAGCCTGGGGACC  240
            ||||||||||||||||||||||||||||||||||||||||||||||||||||||||||||
Sbjct  204  CAGCGCCGCTCGATTGGTAGGTTTTCTCCGGAAGACATGCCGTCGAGTAGCCTGGGGACC  263

Query  241  AACTGCCCGTCCTACCAACGACGGGGGAGCGCGCAAGCNATCCCCCTGACTCTCATTTCG  300
            |||||||||||||||||||||||||||||||||||||| |||||||||||||||||||||
Sbjct  264  AACTGCCCGTCCTACCAACGACGGGGGAGCGCGCAAGCCATCCCCCTGACTCTCATTTCG  323

Query  301  ACCTGAGTTCAGGCAAGACCACCCGCTCAACTTAA  335
            |||||||||||||||||||||||||||||||||||
Sbjct  324  ACCTGAGTTCAGGCAAGACCACCCGCTCAACTTAA  358
```

Chloroidium saccharophilum genomic DNA containing 18S rRNA gene, ITS1, 5.8S rRNA gene, ITS2, 28S rRNA gene, culture collection CCAP 211/31

Sequence ID: **FR865664.1** Length: 2988 Number of Matches: 1

Range 1: 2460 to 2794

Score	Expect	Identities	Gaps	Strand	Frame
612 bits(331)	1e-170()	333/335(99%)	0/335(0%)	Plus/Plus	

Features:

```
Query  1     TGCAGAATTCCGTGAATCATCGAGTCTTTGAACGCATATTGCGCCCGAGGCTTCGGTCNA  60
             |||||||||||||||||||||||||||||||||||||||||||||||||||||||||| |
Sbjct  2460  TGCAGAATTCCGTGAATCATCGAGTCTTTGAACGCATATTGCGCCCGAGGCTTCGGTCAA  2519

Query  61    GGGCATGTCTGCCTCAGCGTCGGTTGACCCCTCTCGTCCAATTCCCTCGTGGAAGCAGAC  120
             ||||||||||||||||||||||||||||||||||||||||||||||||||||||||||||
Sbjct  2520  GGGCATGTCTGCCTCAGCGTCGGTTGACCCCTCTCGTCCAATTCCCTCGTGGAAGCAGAC  2579

Query  121   GGACCTGGCAGTCCCGCTGGCGACAGCGGGTCTGCTGCAGAACAGACGACGAGCCAGCCG  180
             ||||||||||||||||||||||||||||||||||||||||||||||||||||||||||||
Sbjct  2580  GGACCTGGCAGTCCCGCTGGCGACAGCGGGTCTGCTGCAGAACAGACGACGAGCCAGCCG  2639

Query  181   CAGCGCCGCTCGATTGGTAGGTTTTCTCCGGAAGACATGCCGTCGAGTAGCCTGGGGACC  240
             ||||||||||||||||||||||||||||||||||||||||||||||||||||||||||||
Sbjct  2640  CAGCGCCGCTCGATTGGTAGGTTTTCTCCGGAAGACATGCCGTCGAGTAGCCTGGGGACC  2699

Query  241   AACTGCCCGTCCTACCAACGACGGGGGAGCGCGCAAGCNATCCCCCTGACTCTCATTTCG  300
             |||||||||||||||||||||||||||||||||||||| |||||||||||||||||||||
Sbjct  2700  AACTGCCCGTCCTACCAACGACGGGGGAGCGCGCAAGCTATCCCCCTGACTCTCATTTCG  2759

Query  301   ACCTGAGTTCAGGCAAGACCACCCGCTCAACTTAA  335
```

```
             |||||||||||||||||||||||||||||||||||
Sbjct  2760  ACCTGAGTTCAGGCAAGACCACCCGCTCAACTTAA  2794
```

Chloroidium sp. FACHB-2422 small subunit ribosomal RNA gene, partial sequence; internal transcribed spacer 1, 5.8S ribosomal RNA gene, and internal transcribed spacer 2, complete sequence; and large subunit ribosomal RNA gene, partial sequence

Sequence ID: **MT006211.1** Length: 3787 Number of Matches: 1
Range 1: 2888 to 3222

Score	Expect	Identities	Gaps	Strand	Frame
612 bits(331)	1e-170()	333/335(99%)	0/335(0%)	Plus/Plus	

Features:

```
Query  1     TGCAGAATTCCGTGAATCATCGAGTCTTTGAACGCATATTGCGCCCGAGGCTTCGGTCNA  60
             |||||||||||||||||||||||||||||||||||||||||||||||||||||||||| |
Sbjct  2888  TGCAGAATTCCGTGAATCATCGAGTCTTTGAACGCATATTGCGCCCGAGGCTTCGGTCAA  2947

Query  61    GGGCATGTCTGCCTCAGCGTCGGTTGACCCCTCTCGTCCAATTCCCTCGTGGAAGCAGAC  120
             ||||||||||||||||||||||||||||||||||||||||||||||||||||||||||||
Sbjct  2948  GGGCATGTCTGCCTCAGCGTCGGTTGACCCCTCTCGTCCAATTCCCTCGTGGAAGCAGAC  3007

Query  121   GGACCTGGCAGTCCCGCTGGCGACAGCGGGTCTGCTGCAGAACAGACGACGAGCCAGCCG  180
             ||||||||||||||||||||||||||||||||||||||||||||||||||||||||||||
Sbjct  3008  GGACCTGGCAGTCCCGCTGGCGACAGCGGGTCTGCTGCAGAACAGACGACGAGCCAGCCG  3067

Query  181   CAGCGCCGCTCGATTGGTAGGTTTTCTCCGGAAGACATGCCGTCGAGTAGCCTGGGGACC  240
             ||||||||||||||||||||||||||||||||||||||||||||||||||||||||||||
Sbjct  3068  CAGCGCCGCTCGATTGGTAGGTTTTCTCCGGAAGACATGCCGTCGAGTAGCCTGGGGACC  3127

Query  241   AACTGCCCGTCCTACCAACGACGGGGGAGCGCGCAAGCNATCCCCCTGACTCTCATTTCG  300
             |||||||||||||||||||||||||||||||||||||| |||||||||||||||||||||
Sbjct  3128  AACTGCCCGTCCTACCAACGACGGGGGAGCGCGCAAGCCATCCCCCTGACTCTCATTTCG  3187

Query  301   ACCTGAGTTCAGGCAAGACCACCCGCTCAACTTAA  335
             |||||||||||||||||||||||||||||||||||
Sbjct  3188  ACCTGAGTTCAGGCAAGACCACCCGCTCAACTTAA  3222
```

Equisetum arvense voucher Equisetum_arvense_ITS.fasta_2 5.8S ribosomal RNA gene, partial sequence; internal transcribed spacer 2, complete sequence; and large subunit ribosomal RNA gene, partial sequence

Sequence ID: **MW040323.1** Length: 335 Number of Matches: 1
Range 1: 1 to 335

Score	Expect	Identities	Gaps	Strand	Frame
612 bits(331)	1e-170()	335/335(100%)	0/335(0%)	Plus/Plus	

Features:

```
Query  1    TGCAGAATTCCGTGAATCATCGAGTCTTTGAACGCATATTGCGCCCGAGGCTTCGGTCNA  60
            ||||||||||||||||||||||||||||||||||||||||||||||||||||||||||||
Sbjct  1    TGCAGAATTCCGTGAATCATCGAGTCTTTGAACGCATATTGCGCCCGAGGCTTCGGTCNA  60

Query  61   GGGCATGTCTGCCTCAGCGTCGGTTGACCCCTCTCGTCCAATTCCCTCGTGGAAGCAGAC  120
            ||||||||||||||||||||||||||||||||||||||||||||||||||||||||||||
Sbjct  61   GGGCATGTCTGCCTCAGCGTCGGTTGACCCCTCTCGTCCAATTCCCTCGTGGAAGCAGAC  120

Query  121  GGACCTGGCAGTCCCGCTGGCGACAGCGGGTCTGCTGCAGAACAGACGACGAGCCAGCCG  180
            ||||||||||||||||||||||||||||||||||||||||||||||||||||||||||||
Sbjct  121  GGACCTGGCAGTCCCGCTGGCGACAGCGGGTCTGCTGCAGAACAGACGACGAGCCAGCCG  180

Query  181  CAGCGCCGCTCGATTGGTAGGTTTTCTCCGGAAGACATGCCGTCGAGTAGCCTGGGGACC  240
            ||||||||||||||||||||||||||||||||||||||||||||||||||||||||||||
Sbjct  181  CAGCGCCGCTCGATTGGTAGGTTTTCTCCGGAAGACATGCCGTCGAGTAGCCTGGGGACC  240

Query  241  AACTGCCCGTCCTACCAACGACGGGGGAGCGCGCAAGCNATCCCCCTGACTCTCATTTCG  300
            ||||||||||||||||||||||||||||||||||||||||||||||||||||||||||||
Sbjct  241  AACTGCCCGTCCTACCAACGACGGGGGAGCGCGCAAGCNATCCCCCTGACTCTCATTTCG  300

Query  301  ACCTGAGTTCAGGCAAGACCACCCGCTCAACTTAA  335
            |||||||||||||||||||||||||||||||||||
Sbjct  301  ACCTGAGTTCAGGCAAGACCACCCGCTCAACTTAA  335
```

Equisetum arvense voucher MN06 5.8S ribosomal RNA gene, partial sequence; internal transcribed spacer 2, complete sequence; and large subunit ribosomal RNA gene, partial sequence

Sequence ID: **OL304250.1** Length: 335 Number of Matches: 1

Range 1: 1 to 335

Score	Expect	Identities	Gaps	Strand	Frame
606 bits(328)	5e-169()	332/335(99%)	0/335(0%)	Plus/Plus	

Features:

```
Query  1    TGCAGAATTCCGTGAATCATCGAGTCTTTGAACGCATATTGCGCCCGAGGCTTCGGTCNA  60
            |||||||||||||||||||||||||||||||||||||||||||||||||||||||||| |
Sbjct  1    TGCAGAATTCCGTGAATCATCGAGTCTTTGAACGCATATTGCGCCCGAGGCTTCGGTCAA  60

Query  61   GGGCATGTCTGCCTCAGCGTCGGTTGACCCCTCTCGTCCAATTCCCTCGTGGAAGCAGAC  120
            ||||||||||||||||||||||||||||||||||||||||||||||||||||||||||||
Sbjct  61   GGGCATGTCTGCCTCAGCGTCGGTTGACCCCTCTCGTCCAATTCCCTCGTGGAAGCAGAC  120

Query  121  GGACCTGGCAGTCCCGCTGGCGACAGCGGGTCTGCTGCAGAACAGACGACGAGCCAGCCG  180
            ||||||||||||||||||||||||||||||||||||||||||||||||||||||||||||
Sbjct  121  GGACCTGGCAGTCCCGCTGGCGACAGCGGGTCTGCTGCAGAACAGACGACGAGCCAGCCG  180

Query  181  CAGCGCCGCTCGATTGGTAGGTTTTCTCCGGAAGACATGCCGTCGAGTAGCCTGGGGACC  240
            ||||||||||||||||| ||||||||||||||||||||||||||||||||||||||||||
Sbjct  181  CAGCGCCGCTCGATTGGAAGGTTTTCTCCGGAAGACATGCCGTCGAGTAGCCTGGGGACC  240

Query  241  AACTGCCCGTCCTACCAACGACGGGGGAGCGCGCAAGCNATCCCCCTGACTCTCATTTCG  300
            |||||||||||||||||||||||||||||||||||||| |||||||||||||||||||||
Sbjct  241  AACTGCCCGTCCTACCAACGACGGGGGAGCGCGCAAGCCATCCCCCTGACTCTCATTTCG  300

Query  301  ACCTGAGTTCAGGCAAGACCACCCGCTCAACTTAA  335
            |||||||||||||||||||||||||||||||||||
Sbjct  301  ACCTGAGTTCAGGCAAGACCACCCGCTCAACTTAA  335
```

BLAST is a registered trademark of the National Library of Medicine

Support center Mailing list YouTube

- National Library Of Medicine
- National Institutes Of Health
- U.S. Department of Health & Human Services
- USA.gov

NCBI

National Center for Biotechnology Information, U.S. National Library of Medicine 8600 Rockville Pike, Bethesda MD, 20894 USA
Policies and Guidelines | Contact
HHS Vulnerability Disclosure
