# Supplementary material for: A Multi-Pronged Technique for Identifying Equisetum palustre and Equisetum arvense—Combining HPTLC, HPLC-ESI-MS/MS and Optimized DNA Barcoding Techniques
Source: Plants (Basel). 2022 Sep 28;11(19):2562. doi: 10.3390/plants11192562 (PMC9573248; doi:10.3390/plants11192562)
Supplement: Supplementary file 1 [file plants-11-02562-s001.zip › plants-1932149 supplementary/Horsetail Supplementary Figures/Figure S6.pdf]

BLAST ® » blastn suite » RID-FD5FRBPD016

BLAST Results

Questions/comments

Job title: MW040324.1

**RID**	FD5FRBPD016 (Expires on 08-14 02:03 am)		
**Query ID**	lcl|Query_26413	**Database Name**	nt
**Description**	None	**Description**	Nucleotide collection (nt)
**Molecule type**	dna	**Program**	BLASTN 2.13.0+
**Query Length**	335		

Graphic Summary

Distribution of the top 100 Blast Hits on 100 subject sequences

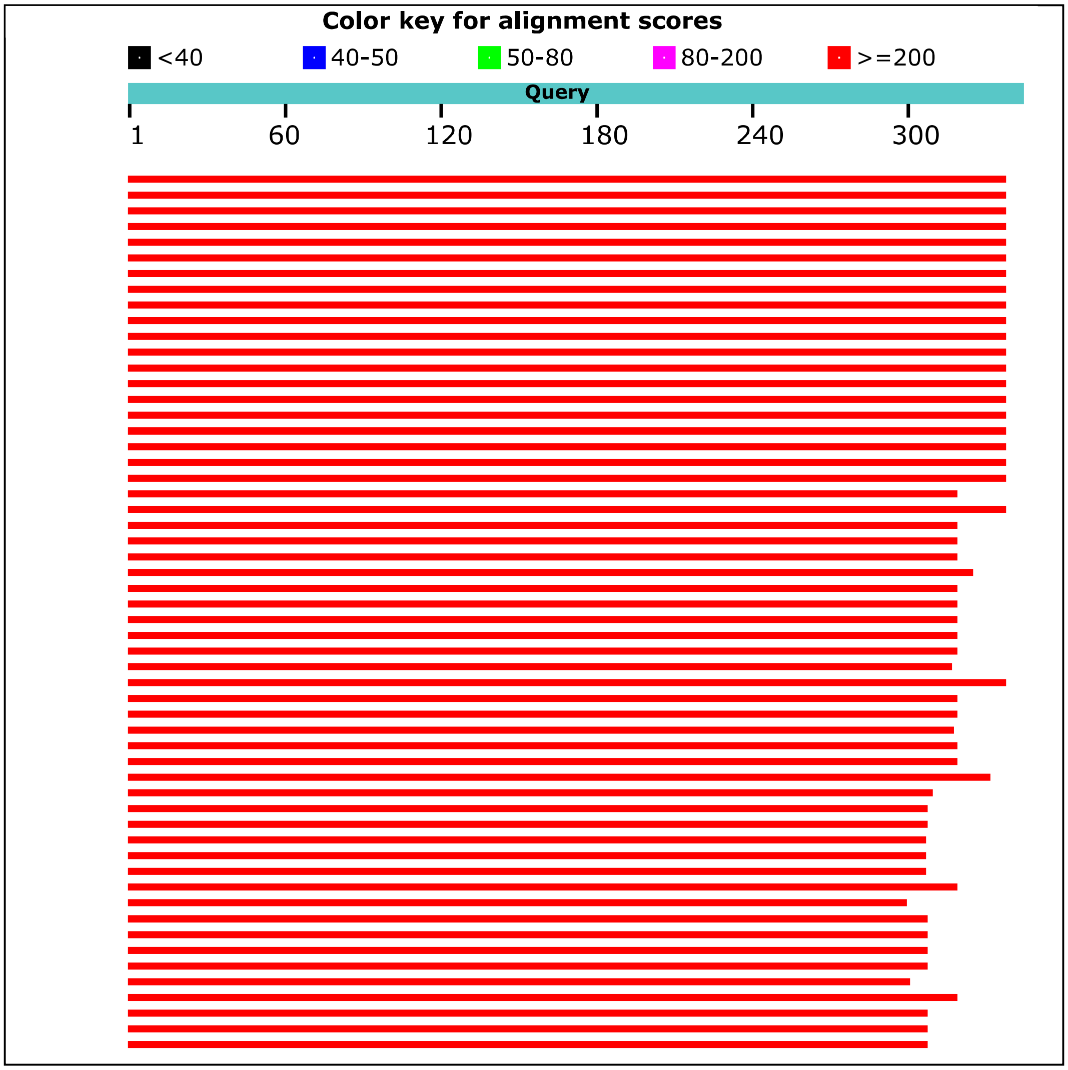

Descriptions

Sequences producing significant alignments:

Description	Max Score	Total Score	Query Cover	E value	Per. Ident	Accession
uncultured fungus genomic DNA sequence contains ITS1	604	604	100%	2e-168	98.81%	OW836430.1
Chloroidium sp. FACHB-2422 small subunit ribosomal RNA gene, partial sequence; internal transcribed spacer 1, 5.8S ribosomal RNA gene, and internal transcribed spacer 2, complete sequence; and large subunit ribosomal RNA gene, partial sequence	604	604	100%	2e-168	98.81%	MT006211.1
Equisetum arvense voucher Equisetum_arvense_ITS.fasta_1 5.8S ribosomal RNA gene, partial sequence; internal transcribed spacer 2, complete sequence; and large subunit ribosomal RNA gene, partial sequence	604	604	100%	2e-168	100.00%	MW040324.1
Equisetum arvense voucher MN06 5.8S ribosomal RNA gene, partial sequence; internal transcribed spacer 2, complete sequence; and large subunit ribosomal RNA gene, partial sequence	599	599	100%	9e-167	98.51%	OL304250.1
Equisetum arvense voucher MN05 5.8S ribosomal RNA gene, partial sequence; internal transcribed spacer 2, complete sequence; and large subunit ribosomal RNA gene, partial sequence	599	599	100%	9e-167	98.51%	OL304249.1
Chloroidium saccharophilum genomic DNA containing 18S rRNA gene, ITS1, 5.8S rRNA gene, ITS2, 28S rRNA gene, culture collection CCAP 211/31	599	599	100%	9e-167	98.51%	FR865664.1
Equisetum arvense voucher Equisetum_arvense_ITS.fasta_2 5.8S ribosomal RNA gene, partial sequence; internal transcribed spacer 2, complete sequence; and large subunit ribosomal RNA gene, partial sequence	597	597	100%	3e-166	98.21%	MW040323.1
Chloroidium saccharophilum strain FACHB-1796 18S ribosomal RNA gene, partial sequence; internal transcribed spacer 1, 5.8S ribosomal RNA gene, and internal transcribed spacer 2, complete sequence; and 28S ribosomal RNA gene, partial sequence	593	593	100%	4e-165	98.21%	KX024689.1
uncultured Pezizaceae genomic DNA sequence contains ITS1	593	593	100%	4e-165	98.21%	OU496020.1
Equisetum arvense voucher 34008 5.8S ribosomal RNA gene, partial sequence; internal transcribed spacer 2, complete sequence; and large subunit ribosomal RNA gene, partial sequence	593	593	100%	4e-165	97.91%	MW040322.1
Equisetum arvense voucher MN01 5.8S ribosomal RNA gene, partial sequence; internal transcribed spacer 2, complete sequence; and large subunit ribosomal RNA gene, partial sequence	590	590	100%	5e-164	98.21%	OL304248.1

Description	Max Score	Total Score	Query Cover	E value	Per. Ident	Accession
Unclassified Viridiplantae clone 1537 5.8S ribosomal RNA gene, partial sequence; internal transcribed spacer 2, complete sequence; and large subunit ribosomal RNA gene, partial sequence	588	588	100%	2e-163	97.91%	MF570051.1
Chloroidium ellipsoideum isolate FG2/4.5E 18S ribosomal RNA gene, partial sequence; internal transcribed spacer 1, 5.8S ribosomal RNA gene, and internal transcribed spacer 2, complete sequence; and 26S ribosomal RNA gene, partial sequence	588	588	100%	2e-163	97.91%	KM020071.1
Chloroidium saccharophilum isolate FG2/1.3 18S ribosomal RNA gene, partial sequence; internal transcribed spacer 1, 5.8S ribosomal RNA gene, and internal transcribed spacer 2, complete sequence; and 26S ribosomal RNA gene, partial sequence	588	588	100%	2e-163	97.91%	KM020070.1
Chloroidium saccharophilum var. saccharophilum strain UTEX247 18S ribosomal RNA gene, partial sequence; internal transcribed spacer 1, 5.8S ribosomal RNA gene, and internal transcribed spacer 2, complete sequence; and 28S ribosomal RNA gene, partial sequence	588	588	100%	2e-163	97.91%	KJ676130.1
Chloroidium saccharophilum strain OD-1-1-C small subunit ribosomal RNA gene, partial sequence; internal transcribed spacer 1, 5.8S ribosomal RNA gene, and internal transcribed spacer 2, complete sequence; and large subunit ribosomal RNA gene, partial sequence	588	588	100%	2e-163	97.91%	MT991539.1
Chloroidium ellipsoideum genomic DNA containing 18S rRNA gene, ITS1, 5.8S rRNA gene, ITS2, 28S rRNA gene, culture collection CCAP 211/40	588	588	100%	2e-163	97.91%	FR865669.1
Chloroidium saccharophilum genomic DNA containing 18S rRNA gene, ITS1, 5.8S rRNA gene, ITS2, 28S rRNA gene, culture collection CCAP 211/32	588	588	100%	2e-163	97.91%	FR865665.1
Chloroidium saccharophilum var. saccharophilum strain UTEX2469 18S ribosomal RNA gene, partial sequence; internal transcribed spacer 1, 5.8S ribosomal RNA gene, and internal transcribed spacer 2, complete sequence; and 28S ribosomal RNA gene, partial sequence	577	577	100%	4e-160	97.31%	KJ676131.1
Chloroidium sp. FACHB-2420 small subunit ribosomal RNA gene, partial sequence; internal transcribed spacer 1, 5.8S ribosomal RNA gene, and internal transcribed spacer 2, complete sequence; and large subunit ribosomal RNA gene, partial sequence	577	577	100%	4e-160	97.31%	MT006209.1
Chloroidium saccharophilum strain ISBAL-007 18S ribosomal RNA gene, internal transcribed spacer 1, 5.8S ribosomal RNA gene, internal transcribed spacer 2, and 28S ribosomal RNA gene, region	573	573	94%	5e-159	99.05%	MH551492.1

Description	Max Score	Total Score	Query Cover	E value	Per. Ident	Accession
'Chlorella' ellipsoidea strain Ce 18S ribosomal RNA gene, partial sequence; internal transcribed spacer 1, 5.8S ribosomal RNA gene, and internal transcribed spacer 2, complete sequence; and 26S ribosomal RNA gene, partial sequence	571	571	100%	2e-158	97.01%	EU038292.1
Chlorella saccharophila partial 18S rRNA gene, ITS1, 5.8S rRNA, ITS2 and partial 28S rRNA gene, strain SAG 2120	562	562	94%	1e-155	98.42%	FM946008.1
Chlorella saccharophila partial 18S rRNA gene, ITS1, 5.8S rRNA, ITS2 and partial 28S rRNA gene, strain CCAP 211/42	562	562	94%	1e-155	98.42%	FM946004.1
Chlorella saccharophila partial 18S rRNA gene, ITS1, 5.8S rRNA, ITS2 and partial 28S rRNA gene, strain CCAP 211/36	562	562	94%	1e-155	98.42%	FM946003.1
Chlorella sp. UTEX 2068 18S ribosomal RNA gene, partial sequence; internal transcribed spacer 1, 5.8S ribosomal RNA gene, and internal transcribed spacer 2, complete sequence; and 28S ribosomal RNA gene, partial sequence	558	558	96%	2e-154	97.52%	KJ676132.1
Chloroidium saccharophilum strain CCAP 211/27 18S ribosomal RNA gene, internal transcribed spacer 1, 5.8S ribosomal RNA gene, internal transcribed spacer 2, and 28S ribosomal RNA gene, region	556	556	94%	5e-154	98.10%	MH551491.1
Chloroidium saccharophilum strain CCAP 211/40 small subunit ribosomal RNA gene, partial sequence; internal transcribed spacer 1 and 5.8S ribosomal RNA gene, complete sequence; and internal transcribed spacer 2, partial sequence	556	556	94%	5e-154	98.10%	MH551490.1
Chlorella saccharophila partial 18S rRNA gene, ITS1, 5.8S rRNA, ITS2 and partial 28S rRNA gene, strain SAG 2197	556	556	94%	5e-154	98.10%	FM946010.1
Chlorella saccharophila partial 18S rRNA gene, ITS1, 5.8S rRNA, ITS2 and partial 28S rRNA gene, strain SAG 211-1c	556	556	94%	5e-154	98.10%	FM946007.1
Chlorella saccharophila partial 18S rRNA gene, ITS1, 5.8S rRNA, ITS2 and partial 28S rRNA gene, strain CCAP 211/32	556	556	94%	5e-154	98.10%	FM946001.1
Chloroidium saccharophilum strain KMMCC 192 18S ribosomal RNA gene, internal transcribed spacer 1, 5.8S ribosomal RNA gene, internal transcribed spacer 2, and 28S ribosomal RNA gene, region	553	553	93%	7e-153	98.09%	JQ315766.1
Chloroidium saccharophilum genomic DNA containing 18S rRNA gene, ITS1, 5.8S rRNA gene, ITS2, 28S rRNA gene, culture collection CCAP 211/58	553	553	100%	7e-153	96.12%	FR865677.1
Chloroidium saccharophilum strain FalI/B-1b small subunit ribosomal RNA gene, partial sequence; internal transcribed spacer 1 and 5.8S ribosomal RNA gene, complete sequence; and internal transcribed spacer 2, partial sequence	551	551	94%	3e-152	97.78%	MH551489.1

Description	Max Score	Total Score	Query Cover	E value	Per. Ident	Accession
Chloroidium saccharophilum strain SAG 2055 small subunit ribosomal RNA gene, partial sequence; internal transcribed spacer 1 and 5.8S ribosomal RNA gene, complete sequence; and internal transcribed spacer 2, partial sequence	551	551	94%	3e-152	97.78%	MH551488.1
Uncultured alga genes for 5.8S rRNA, ITS2, 28S rRNA, partial and complete sequence, note: OTU163	549	549	94%	9e-152	97.78%	LC381753.1
Chlorella saccharophila partial 18S rRNA gene, ITS1, 5.8S rRNA, ITS2 and partial 28S rRNA gene, strain SAG 211-1b	549	549	94%	9e-152	97.78%	FM946005.1
Chlorella saccharophila partial 18S rRNA gene, ITS1, 5.8S rRNA, ITS2 and partial 28S rRNA gene, strain SAG 211-9a	545	545	94%	1e-150	97.47%	FM946000.1
Chloroidium saccharophilum strain KMMCC 158 18S ribosomal RNA gene, internal transcribed spacer 1, 5.8S ribosomal RNA gene, internal transcribed spacer 2, and 28S ribosomal RNA gene, region	540	540	98%	6e-149	95.47%	JQ315771.1
Chloroidium saccharophilum strain KMMCC 195 18S ribosomal RNA gene, internal transcribed spacer 1, 5.8S ribosomal RNA gene, internal transcribed spacer 2, and 28S ribosomal RNA gene, region	534	534	91%	3e-147	97.72%	JQ315769.1
Uncultured Chloroidium isolate A207 internal transcribed spacer 1, partial sequence; 5.8S ribosomal RNA gene, complete sequence; and internal transcribed spacer 2, partial sequence	531	531	91%	3e-146	97.70%	KP314644.1
Uncultured Chloroidium isolate A103 internal transcribed spacer 1, partial sequence; 5.8S ribosomal RNA gene, complete sequence; and internal transcribed spacer 2, partial sequence	531	531	91%	3e-146	97.70%	KP314563.1
Chloroidium saccharophilum strain St6 small subunit ribosomal RNA gene, partial sequence; internal transcribed spacer 1 and 5.8S ribosomal RNA gene, complete sequence; and internal transcribed spacer 2, partial sequence	529	529	90%	1e-145	97.70%	OM472016.1
Chloroidium saccharophilum strain ACSSI 340 small subunit ribosomal RNA gene, partial sequence; internal transcribed spacer 1 and 5.8S ribosomal RNA gene, complete sequence; and internal transcribed spacer 2, partial sequence	529	529	90%	1e-145	97.70%	MT827198.1
Chloroidium saccharophilum strain ACSSI 339 small subunit ribosomal RNA gene, partial sequence; internal transcribed spacer 1 and 5.8S ribosomal RNA gene, complete sequence; and internal transcribed spacer 2, partial sequence	529	529	90%	1e-145	97.70%	MT827197.1
Chloroidium saccharophilum strain CCAP 264/2 small subunit ribosomal RNA gene, partial sequence; internal transcribed spacer 1 and 5.8S ribosomal RNA gene, complete sequence; and internal transcribed spacer 2, partial sequence	527	527	94%	4e-145	96.52%	MH551493.1

Description	Max Score	Total Score	Query Cover	E value	Per. Ident	Accession
Chloroidium saccharophilum strain CCM-UDEC 143 18S ribosomal RNA gene, partial sequence; and internal transcribed spacer 1, 5S ribosomal RNA gene, and internal transcribed spacer 2, complete sequence	527	527	88%	4e-145	98.32%	KC517116.1
Uncultured Chloroidium isolate A214 internal transcribed spacer 1, partial sequence; 5.8S ribosomal RNA gene, complete sequence; and internal transcribed spacer 2, partial sequence	525	525	91%	2e-144	97.38%	KP314651.1
Uncultured Chloroidium isolate A032 internal transcribed spacer 1, partial sequence; 5.8S ribosomal RNA gene, complete sequence; and internal transcribed spacer 2, partial sequence	525	525	91%	2e-144	97.38%	KP314515.1
Uncultured Chloroidium isolate A029 internal transcribed spacer 1, partial sequence; 5.8S ribosomal RNA gene, complete sequence; and internal transcribed spacer 2, partial sequence	525	525	91%	2e-144	97.38%	KP314513.1
Uncultured Chloroidium isolate A011 internal transcribed spacer 1, partial sequence; 5.8S ribosomal RNA gene, complete sequence; and internal transcribed spacer 2, partial sequence	525	525	91%	2e-144	97.38%	KP314497.1
Chloroidium saccharophilum genomic DNA sequence contains 5.8S rRNA gene, ITS2, strain Chlorella saccharophila RNY	523	523	88%	6e-144	97.99%	LR215745.1
Chloroidium saccharophilum strain CCAP 211/58 small subunit ribosomal RNA gene, partial sequence; internal transcribed spacer 1 and 5.8S ribosomal RNA gene, complete sequence; and internal transcribed spacer 2, partial sequence	521	521	94%	2e-143	96.20%	MH551494.1
Uncultured Chloroidium isolate A023 internal transcribed spacer 1, partial sequence; 5.8S ribosomal RNA gene, complete sequence; and internal transcribed spacer 2, partial sequence	518	518	91%	3e-142	97.05%	KP314508.1
Uncultured Chloroidium isolate A141 internal transcribed spacer 1, partial sequence; 5.8S ribosomal RNA gene, complete sequence; and internal transcribed spacer 2, partial sequence	512	512	91%	1e-140	96.72%	KP314596.1
Uncultured Chloroidium isolate A217 internal transcribed spacer 1, partial sequence; 5.8S ribosomal RNA gene, complete sequence; and internal transcribed spacer 2, partial sequence	507	507	91%	6e-139	96.39%	KP314654.1
'Chlorella' saccharophila voucher UTEX 247 internal transcribed spacer 1, partial sequence; 5.8S ribosomal RNA gene, complete sequence; and internal transcribed spacer 2, partial sequence	499	499	91%	9e-137	95.48%	EF120783.1

Description	Max Score	Total Score	Query Cover	E value	Per. Ident	Accession
Chloroidium saccharophilum voucher Herbarium Tuerk51488 internal transcribed spacer 1, partial sequence; 5.8S ribosomal RNA gene, complete sequence; and internal transcribed spacer 2, partial sequence	497	497	84%	3e-136	97.89%	KF907674.1
Chloroidium saccharophilum voucher Herbarium Tuerk51481 internal transcribed spacer 1, partial sequence; 5.8S ribosomal RNA gene, complete sequence; and internal transcribed spacer 2, partial sequence	497	497	84%	3e-136	97.89%	KF907673.1
Chloroidium saccharophilum voucher Herbarium Tuerk51492 internal transcribed spacer 1, partial sequence; 5.8S ribosomal RNA gene, complete sequence; and internal transcribed spacer 2, partial sequence	492	492	84%	2e-134	97.54%	KF907675.1
Chloroidium saccharophilum voucher Herbarium Tuerk51480 internal transcribed spacer 1, partial sequence; 5.8S ribosomal RNA gene, complete sequence; and internal transcribed spacer 2, partial sequence	492	492	84%	2e-134	97.54%	KF907672.1
'Chlorella' saccharophila voucher UTEX 2911 internal transcribed spacer 1, partial sequence; 5.8S ribosomal RNA gene, complete sequence; and internal transcribed spacer 2, partial sequence	472	472	81%	2e-128	97.45%	EF120782.1
Chloroidium sp. S4MWC-51 internal transcribed spacer 1, 5.8S ribosomal RNA gene, and internal transcribed spacer 2, complete sequence	462	462	79%	1e-125	97.74%	MK005103.1
Uncultured Chloroidium isolate A026 internal transcribed spacer 1, partial sequence; 5.8S ribosomal RNA gene, complete sequence; and internal transcribed spacer 2, partial sequence	425	425	91%	2e-114	91.50%	KP314510.1
'Chlorella' saccharophila voucher UTEX 2469 internal transcribed spacer 1, partial sequence; 5.8S ribosomal RNA gene, complete sequence; and internal transcribed spacer 2, partial sequence	411	411	70%	5e-110	97.47%	EF120784.1
Chloroidium saccharophilum strain KMMCC 192 18S ribosomal RNA gene, internal transcribed spacer 1, 5.8S ribosomal RNA gene, internal transcribed spacer 2, and 28S ribosomal RNA gene, region	351	351	59%	3e-92	97.99%	JQ315767.1
Uncultured alga isolate ASV_573_A 5.8S ribosomal RNA gene, partial sequence; internal transcribed spacer 2, complete sequence; and large subunit ribosomal RNA gene, partial sequence	274	274	100%	6e-69	81.28%	ON119385.1
Chloroidium viscosum strain SAG 56.87 18S ribosomal RNA gene, internal transcribed spacer 1, 5.8S ribosomal RNA gene, internal transcribed spacer 2, and 28S ribosomal RNA gene, region	254	254	94%	8e-63	81.12%	MH551495.1

Description	Max Score	Total Score	Query Cover	E value	Per. Ident	Accession
Chloroidium viscosum strain MG-2 small subunit ribosomal RNA gene, partial sequence; internal transcribed spacer 1 and 5.8S ribosomal RNA gene, complete sequence; and internal transcribed spacer 2, partial sequence	243	243	94%	2e-59	80.53%	MH551496.1
Chloroidium angustoellipsoideum voucher VancurovaA356 internal transcribed spacer 1, partial sequence; 5.8S ribosomal RNA gene and internal transcribed spacer 2, complete sequence; and large subunit ribosomal RNA gene, partial sequence	241	241	100%	6e-59	80.92%	MH415249.1
uncultured fungus genomic DNA sequence contains ITS1	237	237	100%	8e-58	79.50%	OW836154.1
Chloroidium lichenum strain BII/C-1 small subunit ribosomal RNA gene, partial sequence; internal transcribed spacer 1 and 5.8S ribosomal RNA gene, complete sequence; and internal transcribed spacer 2, partial sequence	235	235	77%	3e-57	83.64%	MH551507.1
Chloroidium lichenum strain MG-4 small subunit ribosomal RNA gene, partial sequence; internal transcribed spacer 1 and 5.8S ribosomal RNA gene, complete sequence; and internal transcribed spacer 2, partial sequence	235	235	77%	3e-57	83.64%	MH551506.1
Chloroidium lichenum strain BII/B-9 small subunit ribosomal RNA gene, partial sequence; internal transcribed spacer 1 and 5.8S ribosomal RNA gene, complete sequence; and internal transcribed spacer 2, partial sequence	235	235	77%	3e-57	83.64%	MH551505.1
Uncultured Chlorella clone CM-E-A25 internal transcribed spacer 1, partial sequence; 5.8S ribosomal RNA gene, complete sequence; and internal transcribed spacer 2, partial sequence	235	235	77%	3e-57	83.64%	KJ489091.1
Chlorella angustoellipsoidea partial 18S rRNA gene, ITS1, 5.8S rRNA, ITS2 and partial 28S rRNA gene, strain SAG 2115	235	235	77%	3e-57	83.64%	FM946019.1
Chlorella angustoellipsoidea partial 18S rRNA gene, ITS1, 5.8S rRNA, ITS2 and partial 28S rRNA gene, strain SAG 2144	235	235	77%	3e-57	83.64%	FM946020.1
Chloroidium lichenum strain SAG 2293 18S ribosomal RNA gene, internal transcribed spacer 1, 5.8S ribosomal RNA gene, internal transcribed spacer 2, and 28S ribosomal RNA gene, region	233	233	67%	1e-56	85.17%	MH551510.1
Chloroidium lichenum strain SAG 2294 18S ribosomal RNA gene, internal transcribed spacer 1, 5.8S ribosomal RNA gene, internal transcribed spacer 2, and 28S ribosomal RNA gene, region	233	233	67%	1e-56	85.17%	MH551509.1

Description	Max Score	Total Score	Query Cover	E value	Per. Ident	Accession
Chloroidium sp. LV-2018 voucher VancurovaA318 small subunit ribosomal RNA gene, partial sequence; internal transcribed spacer 1 and 5.8S ribosomal RNA gene, complete sequence; and internal transcribed spacer 2, partial sequence	233	233	67%	1e-56	85.17%	MH415228.1
Chloroidium lichenum strain SAG 2141 18S ribosomal RNA gene, internal transcribed spacer 1, 5.8S ribosomal RNA gene, internal transcribed spacer 2, and 28S ribosomal RNA gene, region	231	231	76%	4e-56	83.21%	MH551512.1
Chloroidium sp. LV-2018 voucher VancurovaA503 small subunit ribosomal RNA gene, partial sequence; internal transcribed spacer 1, 5.8S ribosomal RNA gene, and internal transcribed spacer 2, complete sequence; and large subunit ribosomal RNA gene, partial sequence	231	231	77%	4e-56	83.03%	MH415295.1
Uncultured alga genes for 5.8S rRNA, ITS2, 28S rRNA, partial and complete sequence, note: OTU71	230	230	51%	1e-55	90.29%	LC381752.1
Chloroidium orientale strain VCA-45 (Ce2E) small subunit ribosomal RNA gene, partial sequence; internal transcribed spacer 1, 5.8S ribosomal RNA gene, and internal transcribed spacer 2, complete sequence; and large subunit ribosomal RNA gene, partial sequence	230	230	94%	1e-55	80.18%	MZ558750.1
Chloroidium angustoellipsoideum voucher VancurovaA421.2 internal transcribed spacer 1, partial sequence; 5.8S ribosomal RNA gene and internal transcribed spacer 2, complete sequence; and large subunit ribosomal RNA gene, partial sequence	228	228	76%	5e-55	83.27%	MH415277.1
Chloroidium sp. LV-2018 voucher VancurovaO29 small subunit ribosomal RNA gene, partial sequence; internal transcribed spacer 1, 5.8S ribosomal RNA gene, and internal transcribed spacer 2, complete sequence; and large subunit ribosomal RNA gene, partial sequence	224	224	75%	6e-54	83.27%	MH415404.1
Chloroidium lichenum voucher A114 internal transcribed spacer 1, partial sequence; 5.8S ribosomal RNA gene, complete sequence; and internal transcribed spacer 2, partial sequence	224	224	67%	6e-54	84.68%	OL625124.1
Chloroidium lichenum strain QII/C-5b small subunit ribosomal RNA gene, partial sequence; internal transcribed spacer 1 and 5.8S ribosomal RNA gene, complete sequence; and internal transcribed spacer 2, partial sequence	222	222	67%	2e-53	84.39%	MH551508.1
Chloroidium ellipsoideum voucher RidkaNZ23 internal transcribed spacer 1, partial sequence; 5.8S ribosomal RNA gene, complete sequence; and internal transcribed spacer 2, partial sequence	222	222	51%	2e-53	90.64%	MH415207.1

Description	Max Score	Total Score	Query Cover	E value	Per. Ident	Accession
Chloroidium lichenum strain CCAP 211/33 18S ribosomal RNA gene, internal transcribed spacer 1, 5.8S ribosomal RNA gene, internal transcribed spacer 2, and 28S ribosomal RNA gene, region	220	220	51%	8e-53	90.06%	MH551518.1
Chloroidium sp. Ru-6-6 small subunit ribosomal RNA gene, partial sequence; internal transcribed spacer 1, 5.8S ribosomal RNA gene, and internal transcribed spacer 2, complete sequence; and large subunit ribosomal RNA gene, partial sequence	220	220	51%	8e-53	90.06%	MH703747.1
Chloroidium sp. SY-1-2-B small subunit ribosomal RNA gene, partial sequence; internal transcribed spacer 1, 5.8S ribosomal RNA gene, and internal transcribed spacer 2, complete sequence; and large subunit ribosomal RNA gene, partial sequence	220	220	51%	8e-53	90.06%	MT991543.1
Chloroidium sp. NN-2-O small subunit ribosomal RNA gene, partial sequence; internal transcribed spacer 1, 5.8S ribosomal RNA gene, and internal transcribed spacer 2, complete sequence; and large subunit ribosomal RNA gene, partial sequence	220	220	51%	8e-53	90.06%	MT991540.1
Chloroidium ellipsoideum genomic DNA containing 18S rRNA gene, ITS1, 5.8S rRNA gene, ITS2, 28S rRNA gene, culture collection CCAP 211/33	220	220	51%	8e-53	90.06%	FR865666.1
Chloroidium lichenum strain SAG 2295 small subunit ribosomal RNA gene, partial sequence; internal transcribed spacer 1 and 5.8S ribosomal RNA gene, complete sequence; and internal transcribed spacer 2, partial sequence	219	219	75%	3e-52	82.89%	MH551517.1
Chloroidium sp. LV-2018 voucher VancurovaO27 internal transcribed spacer 1, partial sequence; 5.8S ribosomal RNA gene and internal transcribed spacer 2, complete sequence; and large subunit ribosomal RNA gene, partial sequence	219	219	75%	3e-52	82.89%	MH415402.1
Magnoliophyta sp. YCL-2017 voucher VCL-GB12 5.8S ribosomal RNA gene, partial sequence; internal transcribed spacer 2, complete sequence; and large subunit ribosomal RNA gene, partial sequence	215	215	50%	4e-51	89.88%	KY080678.1
Chloroidium lichenum voucher A67_6 internal transcribed spacer 1, partial sequence; 5.8S ribosomal RNA gene, complete sequence; and internal transcribed spacer 2, partial sequence	213	213	75%	1e-50	82.58%	OL625329.1
Chloroidium ellipsoideum voucher Z7 internal transcribed spacer 1, partial sequence; 5.8S ribosomal RNA gene, complete sequence; and internal transcribed spacer 2, partial sequence	211	211	51%	5e-50	89.47%	OL625378.1

Description	Max Score	Total Score	Query Cover	E value	Per. Ident	Accession
Chloroidium ellipsoideum voucher A689 internal transcribed spacer 1, partial sequence; 5.8S ribosomal RNA gene, complete sequence; and internal transcribed spacer 2, partial sequence	211	211	51%	5e-50	89.47%	OL625330.1

Alignments

uncultured fungus genomic DNA sequence contains ITS1

Sequence ID: **OW836430.1** Length: 358 Number of Matches: 1

Range 1: 24 to 358

Score	Expect	Identities	Gaps	Strand	Frame
604 bits(327)	2e-168()	331/335(99%)	0/335(0%)	Plus/Plus	

Features:

```
Query  1    TGCAGAATTCCGTGAATCATCGAGTCTTTGAACGCATATTGCGCCCGAGGCTTCGGTCAA  60
            ||||||||||||||||||||||||||||||||||||||||||||||||||||||||||||
Sbjct  24   TGCAGAATTCCGTGAATCATCGAGTCTTTGAACGCATATTGCGCCCGAGGCTTCGGTCAA  83

Query  61   GGGCATGTCTGCCTCAGCGTCGGTTGACCCCTCTCGTCCAATTCCCTCGNGGAAGCAGAC  120
            ||||||||||||||||||||||||||||||||||||||||||||||||| ||||||||||
Sbjct  84   GGGCATGTCTGCCTCAGCGTCGGTTGACCCCTCTCGTCCAATTCCCTCGTGGAAGCAGAC  143

Query  121  GGACCTGGNAGTCCCGCTGGCGACAGCGGGTCTGCTGCAGAACAGACGACGANCCAGCCG  180
            |||||||| ||||||||||||||||||||||||||||||||||||||||||| |||||||
Sbjct  144  GGACCTGGCAGTCCCGCTGGCGACAGCGGGTCTGCTGCAGAACAGACGACGAGCCAGCCG  203

Query  181  CAGCGCCGCTCGATTGGTAGGTTTTCTCCGGAAGACATGCCGTCGAGTAGCCTGGGGACC  240
            ||||||||||||||||||||||||||||||||||||||||||||||||||||||||||||
Sbjct  204  CAGCGCCGCTCGATTGGTAGGTTTTCTCCGGAAGACATGCCGTCGAGTAGCCTGGGGACC  263

Query  241  AACTGCCCGTCCTACCAACGACGGGGGAGCGCGCAAGCCATCCCCCTGACTCTCATTTCG  300
            ||||||||||||||||||||||||||||||||||||||||||||||||||||||||||||
Sbjct  264  AACTGCCCGTCCTACCAACGACGGGGGAGCGCGCAAGCCATCCCCCTGACTCTCATTTCG  323

Query  301  ACCTGAGTTCAGGCAANACCACCCGCTCAACTTAA  335
            |||||||||||||||| ||||||||||||||||||
Sbjct  324  ACCTGAGTTCAGGCAAGACCACCCGCTCAACTTAA  358
```

Chloroidium sp. FACHB-2422 small subunit ribosomal RNA gene, partial sequence; internal transcribed spacer 1, 5.8S ribosomal RNA gene, and internal transcribed spacer 2, complete sequence; and large subunit ribosomal RNA gene, partial sequence

Sequence ID: **MT006211.1** Length: 3787 Number of Matches: 1

Range 1: 2888 to 3222

Score	Expect	Identities	Gaps	Strand	Frame
604 bits(327)	2e-168()	331/335(99%)	0/335(0%)	Plus/Plus	

Features:

```
Query  1     TGCAGAATTCCGTGAATCATCGAGTCTTTGAACGCATATTGCGCCCGAGGCTTCGGTCAA  60
             ||||||||||||||||||||||||||||||||||||||||||||||||||||||||||||
Sbjct  2888  TGCAGAATTCCGTGAATCATCGAGTCTTTGAACGCATATTGCGCCCGAGGCTTCGGTCAA  2947

Query  61    GGGCATGTCTGCCTCAGCGTCGGTTGACCCCTCTCGTCCAATTCCCTCGNGGAAGCAGAC  120
             ||||||||||||||||||||||||||||||||||||||||||||||||| ||||||||||
Sbjct  2948  GGGCATGTCTGCCTCAGCGTCGGTTGACCCCTCTCGTCCAATTCCCTCGTGGAAGCAGAC  3007

Query  121   GGACCTGGNAGTCCCGCTGGCGACAGCGGGTCTGCTGCAGAACAGACGACGANCCAGCCG  180
             |||||||| ||||||||||||||||||||||||||||||||||||||||||| |||||||
Sbjct  3008  GGACCTGGCAGTCCCGCTGGCGACAGCGGGTCTGCTGCAGAACAGACGACGAGCCAGCCG  3067

Query  181   CAGCGCCGCTCGATTGGTAGGTTTTCTCCGGAAGACATGCCGTCGAGTAGCCTGGGGACC  240
             ||||||||||||||||||||||||||||||||||||||||||||||||||||||||||||
Sbjct  3068  CAGCGCCGCTCGATTGGTAGGTTTTCTCCGGAAGACATGCCGTCGAGTAGCCTGGGGACC  3127

Query  241   AACTGCCCGTCCTACCAACGACGGGGGAGCGCGCAAGCCATCCCCCTGACTCTCATTTCG  300
             ||||||||||||||||||||||||||||||||||||||||||||||||||||||||||||
Sbjct  3128  AACTGCCCGTCCTACCAACGACGGGGGAGCGCGCAAGCCATCCCCCTGACTCTCATTTCG  3187

Query  301   ACCTGAGTTCAGGCAANACCACCCGCTCAACTTAA  335
```

```
                ||||||||||||||||| ||||||||||||||||||
Sbjct  3188  ACCTGAGTTCAGGCAAGACCACCCGCTCAACTTAA  3222
```

Equisetum arvense voucher Equisetum_arvense_ITS.fasta_1 5.8S ribosomal RNA gene, partial sequence; internal transcribed spacer 2, complete sequence; and large subunit ribosomal RNA gene, partial sequence

Sequence ID: **MW040324.1** Length: 335 Number of Matches: 1
Range 1: 1 to 335

Score	Expect	Identities	Gaps	Strand	Frame
604 bits(327)	2e-168()	335/335(100%)	0/335(0%)	Plus/Plus	

Features:

```
Query  1    TGCAGAATTCCGTGAATCATCGAGTCTTTGAACGCATATTGCGCCCGAGGCTTCGGTCAA  60
            ||||||||||||||||||||||||||||||||||||||||||||||||||||||||||||
Sbjct  1    TGCAGAATTCCGTGAATCATCGAGTCTTTGAACGCATATTGCGCCCGAGGCTTCGGTCAA  60

Query  61   GGGCATGTCTGCCTCAGCGTCGGTTGACCCCTCTCGTCCAATTCCCTCGNGGAAGCAGAC  120
            ||||||||||||||||||||||||||||||||||||||||||||||||||||||||||||
Sbjct  61   GGGCATGTCTGCCTCAGCGTCGGTTGACCCCTCTCGTCCAATTCCCTCGNGGAAGCAGAC  120

Query  121  GGACCTGGNAGTCCCGCTGGCGACAGCGGGTCTGCTGCAGAACAGACGACGANCCAGCCG  180
            ||||||||||||||||||||||||||||||||||||||||||||||||||||||||||||
Sbjct  121  GGACCTGGNAGTCCCGCTGGCGACAGCGGGTCTGCTGCAGAACAGACGACGANCCAGCCG  180

Query  181  CAGCGCCGCTCGATTGGTAGGTTTTCTCCGGAAGACATGCCGTCGAGTAGCCTGGGGACC  240
            ||||||||||||||||||||||||||||||||||||||||||||||||||||||||||||
Sbjct  181  CAGCGCCGCTCGATTGGTAGGTTTTCTCCGGAAGACATGCCGTCGAGTAGCCTGGGGACC  240

Query  241  AACTGCCCGTCCTACCAACGACGGGGGAGCGCGCAAGCCATCCCCCTGACTCTCATTTCG  300
            ||||||||||||||||||||||||||||||||||||||||||||||||||||||||||||
Sbjct  241  AACTGCCCGTCCTACCAACGACGGGGGAGCGCGCAAGCCATCCCCCTGACTCTCATTTCG  300

Query  301  ACCTGAGTTCAGGCAANACCACCCGCTCAACTTAA  335
            |||||||||||||||||||||||||||||||||||
Sbjct  301  ACCTGAGTTCAGGCAANACCACCCGCTCAACTTAA  335
```

Equisetum arvense voucher MN06 5.8S ribosomal RNA gene, partial sequence; internal transcribed spacer 2, complete sequence; and large subunit ribosomal RNA gene, partial sequence

Sequence ID: **OL304250.1** Length: 335 Number of Matches: 1
Range 1: 1 to 335

Score	Expect	Identities	Gaps	Strand	Frame
599 bits(324)	9e-167()	330/335(99%)	0/335(0%)	Plus/Plus	

Features:

```
Query  1    TGCAGAATTCCGTGAATCATCGAGTCTTTGAACGCATATTGCGCCCGAGGCTTCGGTCAA  60
            ||||||||||||||||||||||||||||||||||||||||||||||||||||||||||||
Sbjct  1    TGCAGAATTCCGTGAATCATCGAGTCTTTGAACGCATATTGCGCCCGAGGCTTCGGTCAA  60

Query  61   GGGCATGTCTGCCTCAGCGTCGGTTGACCCCTCTCGTCCAATTCCCTCGNGGAAGCAGAC  120
            ||||||||||||||||||||||||||||||||||||||||||||||||| ||||||||||
Sbjct  61   GGGCATGTCTGCCTCAGCGTCGGTTGACCCCTCTCGTCCAATTCCCTCGTGGAAGCAGAC  120

Query  121  GGACCTGGNAGTCCCGCTGGCGACAGCGGGTCTGCTGCAGAACAGACGACGANCCAGCCG  180
            |||||||| ||||||||||||||||||||||||||||||||||||||||||| |||||||
Sbjct  121  GGACCTGGCAGTCCCGCTGGCGACAGCGGGTCTGCTGCAGAACAGACGACGAGCCAGCCG  180

Query  181  CAGCGCCGCTCGATTGGTAGGTTTTCTCCGGAAGACATGCCGTCGAGTAGCCTGGGGACC  240
            ||||||||||||||||| ||||||||||||||||||||||||||||||||||||||||||
Sbjct  181  CAGCGCCGCTCGATTGGAAGGTTTTCTCCGGAAGACATGCCGTCGAGTAGCCTGGGGACC  240

Query  241  AACTGCCCGTCCTACCAACGACGGGGGAGCGCGCAAGCCATCCCCCTGACTCTCATTTCG  300
            ||||||||||||||||||||||||||||||||||||||||||||||||||||||||||||
Sbjct  241  AACTGCCCGTCCTACCAACGACGGGGGAGCGCGCAAGCCATCCCCCTGACTCTCATTTCG  300

Query  301  ACCTGAGTTCAGGCAANACCACCCGCTCAACTTAA  335
            |||||||||||||||| ||||||||||||||||||
Sbjct  301  ACCTGAGTTCAGGCAAGACCACCCGCTCAACTTAA  335
```

Equisetum arvense voucher MN05 5.8S ribosomal RNA gene, partial sequence; internal transcribed spacer 2, complete sequence; and large subunit ribosomal RNA gene, partial sequence

Sequence ID: **OL304249.1** Length: 335 Number of Matches: 1

Range 1: 1 to 335

Score	Expect	Identities	Gaps	Strand	Frame
599 bits(324)	9e-167()	330/335(99%)	0/335(0%)	Plus/Plus	

Features:

```
Query  1    TGCAGAATTCCGTGAATCATCGAGTCTTTGAACGCATATTGCGCCCGAGGCTTCGGTCAA  60
            ||||||||||||||||||||||||||||||||||||||||||||| ||||||||||||||
Sbjct  1    TGCAGAATTCCGTGAATCATCGAGTCTTTGAACGCATATTGCGCCGGAGGCTTCGGTCAA  60

Query  61   GGGCATGTCTGCCTCAGCGTCGGTTGACCCCTCTCGTCCAATTCCCTCGNGGAAGCAGAC  120
            ||||||||||||||||||||||||||||||||||||||||||||||||| ||||||||||
Sbjct  61   GGGCATGTCTGCCTCAGCGTCGGTTGACCCCTCTCGTCCAATTCCCTCGAGGAAGCAGAC  120

Query  121  GGACCTGGNAGTCCCGCTGGCGACAGCGGGTCTGCTGCAGAACAGACGACGANCCAGCCG  180
            |||||||| ||||||||||||||||||||||||||||||||||||||||||| |||||||
Sbjct  121  GGACCTGGCAGTCCCGCTGGCGACAGCGGGTCTGCTGCAGAACAGACGACGAGCCAGCCG  180

Query  181  CAGCGCCGCTCGATTGGTAGGTTTTCTCCGGAAGACATGCCGTCGAGTAGCCTGGGGACC  240
            ||||||||||||||||||||||||||||||||||||||||||||||||||||||||||||
Sbjct  181  CAGCGCCGCTCGATTGGTAGGTTTTCTCCGGAAGACATGCCGTCGAGTAGCCTGGGGACC  240

Query  241  AACTGCCCGTCCTACCAACGACGGGGGAGCGCGCAAGCCATCCCCCTGACTCTCATTTCG  300
            ||||||||||||||||||||||||||||||||||||||||||||||||||||||||||||
Sbjct  241  AACTGCCCGTCCTACCAACGACGGGGGAGCGCGCAAGCCATCCCCCTGACTCTCATTTCG  300

Query  301  ACCTGAGTTCAGGCAANACCACCCGCTCAACTTAA  335
            |||||||||||||||| ||||||||||||||||||
Sbjct  301  ACCTGAGTTCAGGCAAGACCACCCGCTCAACTTAA  335
```


BLAST is a registered trademark of the National Library of Medicine

Support center Mailing list You Tube YouTube

- NATIONAL LIBRARY OF MEDICINE National Library Of Medicine
- NIH National Institutes Of Health
- U.S. Department of Health & Human Services
- USA.gov Government Made Easy USA.gov

NCBI

National Center for Biotechnology Information, U.S. National Library of Medicine 8600 Rockville Pike, Bethesda MD, 20894 USA

Policies and Guidelines | Contact

HHS Vulnerability Disclosure
