# Supplementary material for: A Multi-Pronged Technique for Identifying Equisetum palustre and Equisetum arvense—Combining HPTLC, HPLC-ESI-MS/MS and Optimized DNA Barcoding Techniques
Source: Plants (Basel). 2022 Sep 28;11(19):2562. doi: 10.3390/plants11192562 (PMC9573248; doi:10.3390/plants11192562)
Supplement: Supplementary file 1 [file plants-11-02562-s001.zip › plants-1932149 supplementary/Horsetail Supplementary Figures/Figure S7.pdf]

BLAST Results

Questions/comments

Job title: Y11470.1

**RID**	FD5HP52R01R (Expires on 08-14 02:04 am)		
**Query ID**	lcl|Query_341209	**Database Name**	nt
**Description**	None	**Description**	Nucleotide collection (nt)
**Molecule type**	dna	**Program**	BLASTN 2.13.0+
**Query Length**	159		

Graphic Summary

Distribution of the top 1 Blast Hits on 1 subject sequences

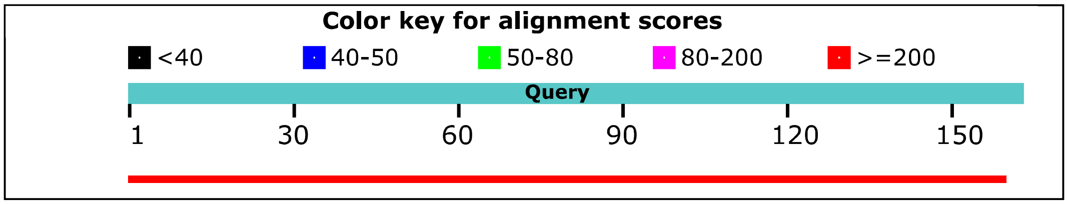

Descriptions

Sequences producing significant alignments:

Description	Max Score	Total Score	Query Cover	E value	Per. Ident	Accession
E.arvense ribosomal DNA, internal transcribed spacer 1	294	294	100%	2e-75	100.00%	Y11470.1

Alignments

E.arvense ribosomal DNA, internal transcribed spacer 1

Sequence ID: **Y11470.1** Length: 159 Number of Matches: 1

Range 1: 1 to 159

Score	Expect	Identities	Gaps	Strand	Frame
294 bits(159)	2e-75()	159/159(100%)	0/159(0%)	Plus/Plus	

Features:

```
Query  1    CCACGCATCCGTGCAAACCCTGCAAATTGTGGACATCCCCCAAGGAGGCGACTTCAATAC  60
            ||||||||||||||||||||||||||||||||||||||||||||||||||||||||||||
Sbjct  1    CCACGCATCCGTGCAAACCCTGCAAATTGTGGACATCCCCCAAGGAGGCGACTTCAATAC  60

Query  61   CCTCCTGCGCCTGTCCGTCCGACATCCAGGGGTCAACTTCTGCCCTCCGATGGAGCACTC  120
            ||||||||||||||||||||||||||||||||||||||||||||||||||||||||||||
Sbjct  61   CCTCCTGCGCCTGTCCGTCCGACATCCAGGGGTCAACTTCTGCCCTCCGATGGAGCACTC  120

Query  121  TTCAATGCGCTGAGACTCCCACATAGGAATATGAAATCA  159
            |||||||||||||||||||||||||||||||||||||||
Sbjct  121  TTCAATGCGCTGAGACTCCCACATAGGAATATGAAATCA  159
```


BLAST is a registered trademark of the National Library of Medicine

Support center Mailing list YouTube

- National Library Of Medicine
- National Institutes Of Health
- U.S. Department of Health & Human Services
- USA.gov

NCBI

National Center for Biotechnology Information, U.S. National Library of Medicine 8600 Rockville Pike, Bethesda MD, 20894 USA

Policies and Guidelines | Contact

HHS Vulnerability Disclosure
