# Supplementary material for: A Multi-Pronged Technique for Identifying Equisetum palustre and Equisetum arvense—Combining HPTLC, HPLC-ESI-MS/MS and Optimized DNA Barcoding Techniques
Source: Plants (Basel). 2022 Sep 28;11(19):2562. doi: 10.3390/plants11192562 (PMC9573248; doi:10.3390/plants11192562)
Supplement: Supplementary file 1 [file plants-11-02562-s001.zip › plants-1932149 supplementary/Horsetail Supplementary Figures/Figure S8.pdf]

BLAST Results

Questions/comments

Job title: Y11471.1

**RID**	FD5KCJW301R (Expires on 08-14 02:05 am)
**Query ID**	lcl|Query_169805
**Description**	None
**Molecule type**	dna
**Query Length**	227
**Database Name**	nt
**Description**	Nucleotide collection (nt)
**Program**	BLASTN 2.13.0+

Graphic Summary

Distribution of the top 1 Blast Hits on 1 subject sequences

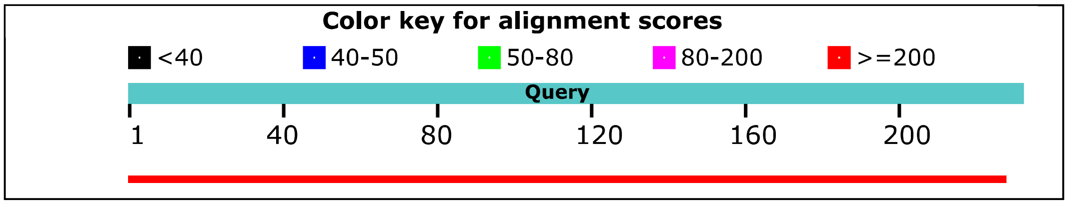

Descriptions

Sequences producing significant alignments:

Description	Max Score	Total Score	Query Cover	E value	Per. Ident	Accession
E.arvense ribosomal DNA, internal transcribed spacer 2	420	420	100%	5e-113	100.00%	Y11471.1

Alignments

E.arvense ribosomal DNA, internal transcribed spacer 2
Sequence ID: **Y11471.1** Length: 227 Number of Matches: 1
Range 1: 1 to 227

Score	Expect	Identities	Gaps	Strand	Frame
420 bits(227)	5e-113()	227/227(100%)	0/227(0%)	Plus/Plus	

Features:

```
Query  1    TACAATCTATTGCGCCTCCCGTAGAGGGAGCGACAACGTGGTCGTCGCAACTCCTCCAGA  60
            ||||||||||||||||||||||||||||||||||||||||||||||||||||||||||||
Sbjct  1    TACAATCTATTGCGCCTCCCGTAGAGGGAGCGACAACGTGGTCGTCGCAACTCCTCCAGA  60

Query  61   GGCACGGTTAGTTGAAATACATCGACGACACTGAGCGGCAAGACGAGTTGTAGGGGTGCT  120
            ||||||||||||||||||||||||||||||||||||||||||||||||||||||||||||
Sbjct  61   GGCACGGTTAGTTGAAATACATCGACGACACTGAGCGGCAAGACGAGTTGTAGGGGTGCT  120

Query  121  CCTTCACCCGCCTTGAGCTCGCGGGGGCTTACAGTGGTCACAGTCATCCGCCTCAGTGCG  180
            ||||||||||||||||||||||||||||||||||||||||||||||||||||||||||||
Sbjct  121  CCTTCACCCGCCTTGAGCTCGCGGGGGCTTACAGTGGTCACAGTCATCCGCCTCAGTGCG  180

Query  181  AAGCTAGAGAGGGCTTTAGGGTCGACCTTGTGCGCTCCCACCTACTC  227
            |||||||||||||||||||||||||||||||||||||||||||||||
Sbjct  181  AAGCTAGAGAGGGCTTTAGGGTCGACCTTGTGCGCTCCCACCTACTC  227
```

BLAST is a registered trademark of the National Library of Medicine

Support center Mailing list YouTube

- National Library Of Medicine
- National Institutes Of Health
- U.S. Department of Health & Human Services
- USA.gov

NCBI
National Center for Biotechnology Information, U.S. National Library of Medicine 8600 Rockville Pike, Bethesda MD, 20894 USA
Policies and Guidelines | Contact
HHS Vulnerability Disclosure
