# Supplementary material for: A Multi-Pronged Technique for Identifying Equisetum palustre and Equisetum arvense—Combining HPTLC, HPLC-ESI-MS/MS and Optimized DNA Barcoding Techniques
Source: Plants (Basel). 2022 Sep 28;11(19):2562. doi: 10.3390/plants11192562 (PMC9573248; doi:10.3390/plants11192562)
Supplement: Supplementary file 1 [file plants-11-02562-s001.zip › plants-1932149 supplementary/Horsetail Supplementary Figures/Figure S9.pdf]

BLAST Results

Questions/comments

Job title: DQ377154.1

**RID**	FD4G8GB201R (Expires on 08-14 01:46 am)
**Query ID**	lcl|Query_50773
**Description**	None
**Molecule type**	dna
**Query Length**	200
**Database Name**	nt
**Description**	Nucleotide collection (nt)
**Program**	BLASTN 2.13.0+

Graphic Summary

Distribution of the top 6 Blast Hits on 5 subject sequences

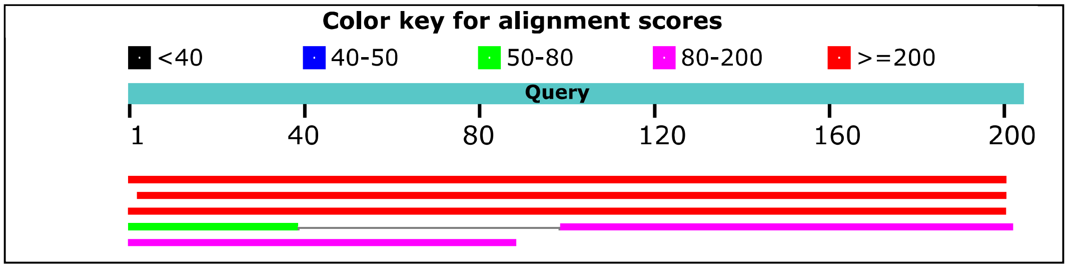

Descriptions

Sequences producing significant alignments:

Description	Max Score	Total Score	Query Cover	E value	Per. Ident	Accession
Equisetum telmateia subsp. telmateia internal transcribed spacer 1, partial sequence	370	370	100%	4e-98	100.00%	DQ377154.1
Equisetum environmental sample clone EDNA16-0043490 internal transcribed spacer 1, partial sequence; 5.8S ribosomal RNA gene and internal transcribed spacer 2, complete sequence; and large subunit ribosomal RNA gene, partial sequence	339	339	99%	1e-88	97.47%	MT198943.1
Equisetum pratense internal transcribed spacer 1, partial sequence	276	276	100%	9e-70	91.58%	DQ377152.1
Equisetum sylvaticum 18S ribosomal RNA gene, partial sequence; internal transcribed spacer 1, complete sequence; and 5.8S ribosomal RNA gene, partial sequence	171	242	70%	5e-38	96.12%	EU328342.1
Equisetum telmateia isolate DM233 small subunit ribosomal RNA gene, internal transcribed spacer 1, 5.8S ribosomal RNA gene, internal transcribed spacer 2, and large subunit ribosomal RNA gene, region	163	163	44%	8e-36	100.00%	MT784095.1

Alignments

Equisetum telmateia subsp. telmateia internal transcribed spacer 1, partial sequence

Sequence ID: **DQ377154.1** Length: 200 Number of Matches: 1

Range 1: 1 to 200

Score	Expect	Identities	Gaps	Strand	Frame
370 bits(200)	4e-98()	200/200(100%)	0/200(0%)	Plus/Plus	

Features:

```
Query  1    CACATCCGTGCAAACCCTGCGAACTGTGGACGTCCCCCAAgggggcggcggctccgatgc  60
            ||||||||||||||||||||||||||||||||||||||||||||||||||||||||||||
Sbjct  1    CACATCCGTGCAAACCCTGCGAACTGTGGACGTCCCCCAAGGGGGCGGCGGCTCCGATGC  60

Query  61   cctctcgcgccctcgcgggcgcgggcgcggcgcggcgccccgtcccctcctcccggccgc  120
            ||||||||||||||||||||||||||||||||||||||||||||||||||||||||||||
Sbjct  61   CCTCTCGCGCCCTCGCGGGCGCGGGCGCGGCGCGGCGCCCCGTCCCCTCCTCCCGGCCGC  120

Query  121  tccggcgtccgggggggTCGAACCTTCCGCCCCTCCGGGGGCGCGGAGCACCCTTAGAAC  180
            ||||||||||||||||||||||||||||||||||||||||||||||||||||||||||||
Sbjct  121  TCCGGCGTCCGGGGGGGTCGAACCTTCCGCCCCTCCGGGGGCGCGGAGCACCCTTAGAAC  180

Query  181  CCTAAACCGTCTGAGACTCC  200
            ||||||||||||||||||||
Sbjct  181  CCTAAACCGTCTGAGACTCC  200
```

Equisetum environmental sample clone EDNA16-0043490 internal transcribed spacer 1, partial sequence; 5.8S ribosomal RNA gene and internal transcribed spacer 2, complete sequence; and large subunit ribosomal RNA gene, partial sequence

Sequence ID: **MT198943.1** Length: 739 Number of Matches: 1

Range 1: 1 to 198

Score	Expect	Identities	Gaps	Strand	Frame
339 bits(183)	1e-88()	193/198(97%)	0/198(0%)	Plus/Plus	

Features:

```
Query  3    CATCCGTGCAAACCCTGCGAACTGTGGACGTCCCCCAAgggggcggcggctccgatgccc  62
            |||||||||||||||||||||||||||||||||||||||||||||||||||||| |||||
Sbjct  1    CATCCGTGCAAACCCTGCGAACTGTGGACGTCCCCCAAGGGGGCGGCGGCTCCGTTGCCC  60

Query  63   tctcgcgccctcgcgggcgcgggcgcggcgcggcgccccgtcccctcctcccggccgctc  122
            || |||||||||||||||||||||||||||||||||||||||||||||||||||||||||
Sbjct  61   TCCCGCGCCCTCGCGGGCGCGGGCGCGGCGCGGCGCCCCGTCCCCTCCTCCCGGCCGCTC  120

Query  123  cggcgtccgggggggTCGAACCTTCCGCCCCTCCGGGGGCGCGGAGCACCCTTAGAACCC  182
            ||||||||||||||||||||||||||||||||||||||||||||||||| ||| |||| |
Sbjct  121  CGGCGTCCGGGGGGGTCGAACCTTCCGCCCCTCCGGGGGCGCGGAGCACTCTTCGAACTC  180

Query  183  TAAACCGTCTGAGACTCC  200
            ||||||||||||||||||
Sbjct  181  TAAACCGTCTGAGACTCC  198
```

Equisetum pratense internal transcribed spacer 1, partial sequence

Sequence ID: **DQ377152.1** Length: 252 Number of Matches: 1

Range 1: 7 to 207

Score	Expect	Identities	Gaps	Strand	Frame
276 bits(149)	9e-70()	185/202(92%)	3/202(1%)	Plus/Plus	

Features:

```
Query  1    CACATCCGTGCAAACCCTGCGAACTGTGGACGTCCCCCAAgggggcggcggctccgatgc  60
            |||||| |||| |||||||||||||||||||||||||||||||||||||| |||| ||||
Sbjct  7    CACATCTGTGC-AACCCTGCGAACTGTGGACGTCCCCCAAGGGGGCGGCGACTCCAATGC  65

Query  61   cctctcgcgccctcgcgggc-gcgggcgcggcgcggcgccccgtcccctcctcccggccg  119
            ||||  |||||||||||||  |||||||| | | ||||||||||||||||||| | ||||
Sbjct  66   CCTCCTGCGCCCTCGCGGGAGGCGGGCGCTGTGTGGCGCCCCGTCCCCTCCTCTCAGCCG  125

Query  120  ctccggcgtccgggggggTCGAACCTTCCGCCCCTCCGGGGGCGCGGAGCACCCTTA-GA  178
            |||||||||||||||||||||||||||||||||||||||||||||| ||||| |||  ||
Sbjct  126  CTCCGGCGTCCGGGGGGGTCGAACCTTCCGCCCCTCCGGGGGCGCGAAGCACTCTTTCGA  185

Query  179  ACCCTAAACCGTCTGAGACTCC  200
            ||||||||||||||||||||||
Sbjct  186  ACCCTAAACCGTCTGAGACTCC  207
```

Equisetum sylvaticum 18S ribosomal RNA gene, partial sequence; internal transcribed spacer 1, complete sequence; and 5.8S ribosomal RNA gene, partial sequence

Sequence ID: **EU328342.1** Length: 230 Number of Matches: 2

Range 1: 63 to 165

Score	Expect	Identities	Gaps	Strand	Frame
171 bits(92)	5e-38()	99/103(96%)	0/103(0%)	Plus/Plus	

Features:

```
Query  98   ccccgtcccctcctcccggccgctccggcgtccgggggggTCGAACCTTCCGCCCCTCCG  157
            ||||||||||||||||||||||||||||||||||||||||||||||||||||||||||||
Sbjct  63   CCCCGTCCCCTCCTCCCGGCCGCTCCGGCGTCCGGGGGGGTCGAACCTTCCGCCCCTCCG  122

Query  158  GGGGCGCGGAGCACCCTTAGAACCCTAAACCGTCTGAGACTCC  200
            |||||||||||||||||| |||||   ||||||||||||||||
Sbjct  123  GGGGCGCGGAGCACCCTTCGAACCGNGAACCGTCTGAGACTCC  165
```

Range 2: 28 to 65

Score	Expect	Identities	Gaps	Strand	Frame
71.3 bits(38)	5e-08()	38/38(100%)	0/38(0%)	Plus/Plus	

Features:

```
Query  1    CACATCCGTGCAAACCCTGCGAACTGTGGACGTCCCCC  38
            ||||||||||||||||||||||||||||||||||||||
Sbjct  28   CACATCCGTGCAAACCCTGCGAACTGTGGACGTCCCCC  65
```

Equisetum telmateia isolate DM233 small subunit ribosomal RNA gene, internal transcribed spacer 1, 5.8S ribosomal RNA gene, internal transcribed spacer 2, and large subunit ribosomal RNA gene, region

Sequence ID: **MT784095.1** Length: 1902 Number of Matches: 1

Range 1: 1815 to 1902

Score	Expect	Identities	Gaps	Strand	Frame
163 bits(88)	8e-36()	88/88(100%)	0/88(0%)	Plus/Plus	

Features:

```
Query  1     CACATCCGTGCAAACCCTGCGAACTGTGGACGTCCCCCAAgggggcggcggctccgatgc  60
             ||||||||||||||||||||||||||||||||||||||||||||||||||||||||||||
Sbjct  1815  CACATCCGTGCAAACCCTGCGAACTGTGGACGTCCCCCAAGGGGGCGGCGGCTCCGATGC  1874

Query  61    cctctcgcgccctcgcgggcgcgggcgc  88
             ||||||||||||||||||||||||||||
Sbjct  1875  CCTCTCGCGCCCTCGCGGGCGCGGGCGC  1902
```

BLAST is a registered trademark of the National Library of Medicine

Support center Mailing list YouTube

- National Library Of Medicine
- National Institutes Of Health
- U.S. Department of Health & Human Services
- USA.gov

NCBI

National Center for Biotechnology Information, U.S. National Library of Medicine 8600 Rockville Pike, Bethesda MD, 20894 USA

Policies and Guidelines | Contact

HHS Vulnerability Disclosure
