# Supplementary material for: A Multi-Pronged Technique for Identifying Equisetum palustre and Equisetum arvense—Combining HPTLC, HPLC-ESI-MS/MS and Optimized DNA Barcoding Techniques
Source: Plants (Basel). 2022 Sep 28;11(19):2562. doi: 10.3390/plants11192562 (PMC9573248; doi:10.3390/plants11192562)
Supplement: Supplementary file 1 [file plants-11-02562-s001.zip › plants-1932149 supplementary/Supplementary Materials Figure captions.pdf]

Supplementary Materials

Figure S1. BLAST query results for the sequence contained in GenBank accession OL304248.1. The only *Equisetum* accessions matched by this sequence were OL304248.1 (itself), OL304249.1, OL304250.1, MW040322.1, MW040323.1, and MW040324.1. The remainder of the matches were green algae or fungus.

Figure S2. BLAST query results for the sequence contained in GenBank accession OL304249.1. The only *Equisetum* accessions matched by this sequence are OL304248.1, OL304249.1 (itself), OL304250.1, MW040322.1, MW040323.1, and MW040324.1. The remainder of the matches are green algae or fungus.

Figure S3. BLAST query results for the sequence contained in GenBank accession OL304250.1. The only *Equisetum* accessions matched by this sequence were OL304248.1, OL304249.1, OL304250.1 (itself), MW040322.1, MW040323.1, and MW040324.1. The remainder of the matches were green algae or fungus.

Figure S4. BLAST query results for the sequence contained in GenBank accession MW040322.1. The only *Equisetum* accessions matched by this sequence were OL304248.1, OL304249.1, OL304250.1, MW040322.1 (itself), MW040323.1, and MW040324.1. The remainder of the matches were green algae or fungus.

Figure S5. BLAST query results for the sequence contained in GenBank accession MW040323.1. The only *Equisetum* accessions matched by this sequence were OL304248.1, OL304249.1, OL304250.1, MW040322.1, MW040323.1 (itself), and MW040324.1. The remainder of the matches were green algae or fungus.

Figure S6. BLAST query results for the sequence contained in GenBank accession MW040324.1. The only *Equisetum* accessions matched by this sequence were OL304248.1, OL304249.1, OL304250.1, MW040322.1, MW040323.1, and MW040324.1 (itself). The remainder of the matches were green algae or fungus.

Figure S7. BLAST query results for the sequence contained in GenBank accession Y11470.1. The only match for this sequence was itself.

Figure S8. BLAST query results for the sequence contained in GenBank accession Y11471.1. The only match for this sequence was itself.

Figure S9. BLAST query results for the sequence contained in GenBank accession DQ377154.1. This sequence matched accessions belonging to several *Equisetum* species. No matches were from a different genus.

Figure S10. BLAST query results for the sequence contained in GenBank accession DQ377155.1. This sequence matched accessions belonging to several *Equisetum* species, which dominated the top matches.

Figure S11. BLAST query results for the sequence contained in GenBank accession DQ377156.1. This sequence matched accessions belonging to several *Equisetum* species. No matches were from a different genus.

Figure S12. BLAST query results for the sequence contained in GenBank accession DQ377157.1. This sequence matched accessions belonging to several *Equisetum* species. No matches were from a different genus.

Figure S13. BLAST query results for the sequence contained in GenBank accession AF448794.1. This sequence matched accessions belonging to several *Equisetum* species (including those contributed by this study), which dominated the top matches.

Figure S14. BLAST query results for the sequence contained in GenBank accession KT960212.1. The only *Equisetum* accession matched by this sequence was itself. All other matches were green algae.

Figure S15. BLAST query results for the sequence contained in GenBank accession EU328339.1. This sequence matched accessions belonging to several *Equisetum* species, which dominated the top matches.

Figure S16. BLAST query results for the sequence contained in GenBank accession MT198943.1. This sequence matched accessions belonging to several *Equisetum* species (including those contributed by this study), which dominated the top matches.

Figure S17. Alignment of the *Equisetum arvense* ITS sequences downloaded from GenBank and the ITS-p3u4 sequences obtained from vouchered plants. Sequences are denoted by their corresponding GenBank accession codes (ending in ".1") or our sample IDs (A3 to A9). Dashes (-) indicate indel areas. The color scheme is based on the BLOSUM62 scores for each nucleotide position, where: cyan indicates BLOSUM62 score >=3, grey indicates BLOSUM62 score >=0.2; white indicates BLOSUM62 score <0.2 [30]. Higher BLOSUM62 scores indicate a higher probability of nucleotide matching. Sequences of accessions Y11470.1 and Y11471.1 are each unique. Sequences from accessions MW040322.1, MW040323.1, MW040324.1, OL304248.1, OL304249.1, and OL304250.1 align well with each

other, but not with other sequences. Sequences of A3 to A9 are consistent with each other, but not with those from GenBank. This alignment indicates that our sequences are distinct from the existing GenBank sequences.

Figure S18. Alignment of the *Equisetum arvense* and *Equisetum palustre* ITS-p3u4 sequences obtained from the vouchered plants. Sequences are denoted by our sample IDs (A3 to A9, P3 to P10). Dashes (-) indicate indel areas. The color scheme is based on BLOSUM62 scores for each nucleotide position, where: cyan indicates BLOSUM62 score >=3, grey indicates BLOSUM62 score >=0.2; white indicates BLOSUM62 score <0.2 [30]. Higher BLOSUM62 scores indicate higher probability of nucleotide matching. All of the *E. palustre* samples produced longer ITS-p3u4 sequences than their *E. arvense* counterparts. However, for the entirety of the length of *E. arvense* sequences, the two species were largely the same; several nucleotide mismatches could be seen among the well-aligned region, as denoted by white spots among the cyan. This alignment shows distinguishing differences between *E. arvense* and *E. palustre* in terms of the ITS marker, as sequenced by the "p3u4" primer pair. The said marker, in turn, is a powerful DNA barcoding target to use for the identification of these *Equisetum* species.

Figure S19. BLAST query results for sample P1 (78075211104-1), *rbcL* marker. The query sequence was obtained using the *rbcL*-a primer pair. Numerous accessions matched the query sequence with an E-value of "0.0". Among these, the best match can be interpreted as the accession that produced the highest alignment score, indicating the highest amount of sequence similarity. In this case, the best match was *Equisetum palustre*.

Figure S20. BLAST query results for sample P2 (75890210901-1), *rbcL* marker. The query sequence was obtained using the *rbcL*-a primer pair. Numerous accessions matched the query sequence with an E-value of "0.0". Among these, the best match can be interpreted as the accession that produced the highest alignment score, indicating the highest amount of sequence similarity. In this case, the best match was *Equisetum palustre*.

Figure S21. BLAST query results for sample P3 (82709220323-1), *rbcL* marker. The query sequence was obtained using the *rbcL*-a primer pair. Numerous accessions matched the query sequence with an E-value of "0.0". Among these, the best match can be interpreted as the accession that produced the highest alignment score, indicating the highest amount of sequence similarity. In this case, the best match was *Equisetum palustre*.

Figure S22. BLAST query results for sample P4 (82710220323-1), *rbcL* marker. The query sequence was obtained using the *rbcL*-a primer pair. Numerous accessions matched the query sequence with an E-value of "0.0". Among these, the best match can be interpreted as the accession that produced the highest alignment score, indicating the highest amount of sequence similarity. In this case, the best match was *Equisetum palustre*.

Figure S23. BLAST query results for sample P5 (82711220323-1), *rbcL* marker. The query sequence was obtained using the *rbcL*-a primer pair. Numerous accessions matched the query sequence with an E-value of "0.0". Among these, the best match can be interpreted as the accession that produced the highest alignment score, indicating the highest amount of sequence similarity. In this case, the best match was *Equisetum palustre*.

Figure S24. BLAST query results for sample P6 (82712220323-1), *rbcL* marker. The query sequence was obtained using the *rbcL*-a primer pair. Numerous accessions matched the query sequence with an E-value of "0.0". Among these, the best match can be interpreted as the accession that produced the highest alignment score, indicating the highest amount of sequence similarity. In this case, five accessions produced the highest alignment score, all of which were *Equisetum palustre*.

Figure S25. BLAST query results for sample P8 (82714220323-1), *rbcL* marker. The query sequence was obtained using the *rbcL*-a primer pair. Numerous accessions matched the query sequence with an E-value of "0.0". Among these, the best match can be interpreted as the accession that produced the highest alignment score, indicating the highest amount of sequence similarity. In this case, the best match was *Equisetum palustre*.

Figure S26. BLAST query results for sample P9 (82715220323-1), *rbcL* marker. The query sequence was obtained using the *rbcL*-a primer pair. Numerous accessions matched the query sequence with an E-value of "0.0". Among these, the best match can be interpreted as the accession that produced the highest alignment score, indicating the highest amount of sequence similarity. In this case, two accessions produced the highest alignment score, both of which were *Equisetum palustre*.

Figure S27. BLAST query results for sample P10 (82716220323-1), *rbcL* marker. The query sequence was obtained using the *rbcL*-a primer pair. Numerous accessions matched the query sequence with an E-value of "0.0". Among these, the best match can be interpreted as the accession that produced the highest alignment score, indicating the

highest amount of sequence similarity. In this case, five accessions produced the highest alignment score, all of which were *Equisetum palustre*.

Figure S28. BLAST query results for sample A2 (74323210721-1), *rbcL* marker. The query sequence was obtained using the *rbcL*-a primer pair. Numerous accessions matched the query sequence with an E-value of "0.0". Among these, the best match can be interpreted as the accession that produced the highest alignment score, indicating the highest amount of sequence similarity. In this case, two accessions produced the highest alignment score, both of which were *Equisetum arvense*.

Figure S29. BLAST query results for sample A6 (82704220323-1), *rbcL* marker. The query sequence was obtained using the *rbcL*-a primer pair. Numerous accessions matched the query sequence with an E-value of "0.0". Among these, the best match can be interpreted as the accession that produced the highest alignment score, indicating the highest amount of sequence similarity. In this case, two accessions produced the highest alignment score, both of which were *Equisetum arvense*.

Figure S30. BLAST query results for sample A7 (82708220323-1), *rbcL* marker. The query sequence was obtained using the *rbcL*-a primer pair. Numerous accessions matched the query sequence with an E-value of "0.0". Among these, the best match can be interpreted as the accession that produced the highest alignment score, indicating the highest amount of sequence similarity. In this case, two accessions produced the highest alignment score, both of which were *Equisetum arvense*.

Figure S31. BLAST query results for sample A8 (83919220502-1), *rbcL* marker. The query sequence was obtained using the *rbcL*-a primer pair. Numerous accessions matched the query sequence with an E-value of "0.0". Among these, the best match can be interpreted as the accession that produced the highest alignment score, indicating the highest amount of sequence similarity. In this case, two accessions produced the highest alignment score, both of which were *Equisetum arvense*.

Figure S32. BLAST query results for sample A9 (84087220504-1), *rbcL* marker. The query sequence was obtained using the *rbcL*-a primer pair. Numerous accessions matched the query sequence with an E-value of "0.0". Among these, the best match can be interpreted as the accession that produced the highest alignment score, indicating the highest amount of sequence similarity. In this case, two accessions produced the highest alignment score, both of which were *Equisetum arvense*.

Figure S33. BLAST query results for sample A4 (82702220323-1), *rbcL* marker. The query sequence was obtained using the *rbcL*-a primer pair. Numerous accessions matched the query sequence with an E-value of "0.0". Among these, the best match can be interpreted as the accession that produced the highest alignment score, indicating the highest amount of sequence similarity. However, in this case, numerous accessions derived from different *Equisetum* species produced the highest alignment score. Consequently, when the *rbcL* marker was used, an exact species cannot be determined for this sample and it was only identified to the genus level.

References

1. Edgar, Robert. Belvu Alignment Coloring [Internet]. Available online: https://www.drive5.com/muscle/manual/belvu_color.html (accessed on 29 July 2022).
